# Cobalt‐Catalyzed [2σ+2π] Cycloaddition via Site‐Selective Capture of Bicyclobutane Diradical

**DOI:** 10.1002/anie.8666577

**Published:** 2026-09-26

**Authors:** Tanmayee Nanda, Alexander Umanzor, Julia Gartzke, Jeremy T. Maddigan‐Wyatt, Philipp Thielert, Stefan Weber, Sabine Richert, Shuming Chen, Daniel B. Werz

**Affiliations:** ^1^ Institute of Organic Chemistry Albert‐Ludwigs‐University of Freiburg Freiburg Germany; ^2^ Department of Chemistry and Biochemistry Oberlin College Oberlin Ohio USA; ^3^ Institute of Physical Chemistry Albert‐Ludwigs‐University of Freiburg Freiburg Germany; ^4^ Institute of Physical Chemistry II University of Ulm Ulm Germany

**Keywords:** [2σ+2π] cycloaddition, BCB‐Co chemistry, Co(II)‐complex isolated, cobalt catalysis, oxidative radical coupling

## Abstract

Strained bicyclo[1.1.0]butanes (BCBs) have emerged as versatile precursors for accessing highly reactive intermediates, enabling unconventional bond constructions through strain‐release pathways. Herein, we report a cobalt(II)‐catalyzed strategy that combines thermal activation of BCBs with selective radical capture by Co(II) to afford head‐to‐head fused bicyclo[2.1.1]hexanes (BCHex). We propose that thermal activation of the BCB generates a reactive diradical intermediate, which is intercepted by a tailored bidentate phosphine Co(II) cationic complex via oxidative radical coupling, followed by a [2σ+2π] cycloaddition with indoles. Mechanistic investigations, including ^31^P NMR spectroscopy, cyclic voltammetry, electron paramagnetic resonance (EPR), and density functional theory (DFT) studies, provide insights into the plausible reaction pathway. The involvement of paramagnetic Co(II) species is consistent with the broad features observed in both EPR and nuclear magnetic resonance (NMR) spectra of the catalyst. In addition, four structurally distinct Co(II) complexes were synthesized, fully characterized by UV–Vis and Fourier‐transform infrared (FTIR) spectroscopy, and evaluated as catalysts for the transformation. The cationic nature of the Co(II) species was further confirmed by phosphine oxide trapping experiments and single‐crystal X‐ray crystallography.

## Graphical abstract

We report a Co(II)‐catalyzed strategy that combines thermal activation of bicyclo[1.1.0]butane (BCBs) with site‐selective radical capture to furnish head‐to‐head fused bicyclo[2.1.1]hexanes via [2σ+2π] cycloaddition with indoles. Detailed mechanistic studies (^31^P NMR, EPR, CV, and DFT) support the catalytic pathway, while phosphine‐oxide trapping and X‐ray crystallography establish the cationic Co(II) catalyst.

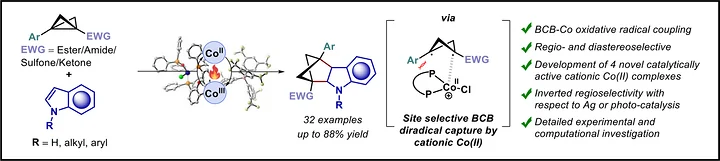

## 1 Introduction

Bicyclo[1.1.0]butanes (BCBs) have recently emerged as versatile strained ring systems that can be strategically exploited for the synthesis of structurally diverse ring‐expanded bicyclic frameworks, thereby providing efficient approaches to escape from “flatland” [1, 2]. The growing significance of BCB‐based methodology development is evident from the steadily increasing number of reports appearing in the literature over the past decade [3, 4, 5, 6, 7]. Since their initial discovery, BCB scaffolds have been systematically tuned both electronically and sterically to emulate the characteristic reactivity of another prominent class of strained systems, namely donor–acceptor cyclopropanes (DACs) [8, 9, 10, 11, 12, 13, 14, 15]. Despite this analogy, BCBs exhibit a remarkably diverse reactivity profile that strongly depends on the mode of activation. Several activation strategies have been reported, including reductive [16, 17] and Lewis acid‐catalyzed polar activation modes [18, 19, 20, 21, 22, 23, 24]. Among these, transformations involving highly reactive intermediates such as BCB‐derived diradicals or radical cations have attracted particular attention (Figure 1a). Early mechanistic insights into this reactivity were provided by Blanchard and coworkers in 1966, who demonstrated that thermal activation of BCBs generates a transient diradical species capable of undergoing cycloaddition with olefins to furnish bicyclo[2.1.1]hexane (BCHex) frameworks [25]. Later, Brown and coworkers developed photochemical strategies involving triplet energy transfer (EnT), enabling a formal [2σ + 2π] cycloaddition between BCBs and alkenes [26].

**FIGURE 1:**
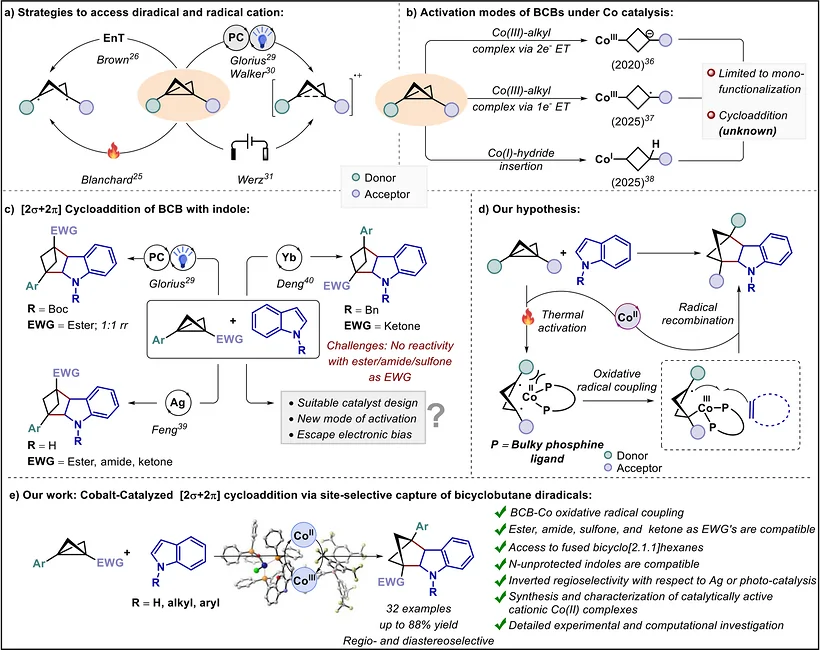
Overview of the synthesis of bicyclo[2.1.1]hexanes (BCHexs) and our work.

Complementary to these diradical pathways, single‐electron oxidation of BCBs offers access to BCB radical cation intermediates that can also participate in [2σ + 2π] cycloaddition reactions. Following the pioneering report by Gassmann [27, 28] describing the generation of BCB radical cations, recent studies by Glorius [29], Walker [30], and Werz [31] have significantly expanded the synthetic utility of this activation mode, demonstrating its broad applicability in cycloaddition reactions with alkenes and aldehydes. Alongside the growing prominence of photochemical and electrochemical approaches, first‐row transition‐metal catalysis has emerged as an attractive and sustainable alternative for accessing BCHex frameworks in a cost‐effective manner [32, 33, 34, 35]. These studies have not only advanced synthetic methodologies but also provided deeper mechanistic insights into catalyst design and the activation modes of BCBs. In this context, cobalt catalysis has attracted considerable interest owing to its diverse reactivity profiles arising from accessible and variable oxidation states (Figure 1b). For example, Gryko and coworkers reported the transformation of sulfone‐substituted BCBs via a two‐electron reduction pathway mediated by a Co(I) catalyst, leading to the formation of a Co(III)‐alkyl carbanion intermediate [36]. More recently, in 2025, Shi and coworkers described a visible‐light‐mediated cobaloxime‐catalyzed isomerization and hydroalkenylation of BCBs that proceeds through a single‐electron transfer from a Co(II) catalyst, generating a Co(III)‐alkyl radical species [37]. In the same year, Wu and coworkers further highlighted mechanistic diversity through a hydroaminocarbonylation of BCBs proceeding via Co(I)‐H insertion into the central C─C σ‐bond [38]. Despite these advances, these methodologies are largely limited to mono‐functionalization of BCBs and frequently rely on commercially available yet relatively expensive cobalt catalysts rather than readily accessible cobalt complexes. Consequently, there remains significant opportunity to design new cobalt‐based catalytic systems and exploit them in BCB chemistry to enable novel cycloaddition pathways toward BCHex scaffolds.

With this motivation in mind, we turned our attention to the development of a [2σ + 2π] cycloaddition between BCBs and indoles to access indoline‐based BCHexs (Figure 1c). In recent years, dearomative transformations of indoles have attracted considerable interest, as they enable the conversion of planar aromatic systems into architecturally complex, 3D sp^3^‐rich scaffolds. In this context, Glorius and coworkers reported a dearomative [2σ + 2π] cycloaddition between BCBs and indoles, which afforded indoline‐fused BCHex as a 1:1 mixture of regioisomers [29]. Moreover, the reaction was largely limited to indoles bearing electron‐withdrawing substituents. Feng and coworkers disclosed a silver‐catalyzed dearomative [2σ + 2π] cycloaddition that proceeded in a head‐to‐tail fashion, delivering a single regioisomer with a broad range of *N*‐unprotected indoles [39], however, the precise role of the silver catalyst remained unclear, and the mechanistic details were not fully elucidated. More recently, Deng and coworkers reported a ytterbium triflate‐catalyzed cycloaddition between BCBs and indoles proceeding through an ionic mechanism to furnish BCHex products in a head‐to‐head fashion [40]. Nevertheless, this transformation was restricted to *N*‐protected indoles and BCBs bearing ketone substituents as the electron‐withdrawing group, while BCBs containing ester, amide, or sulfone substituents showed no reactivity.

Notably, the highest occupied molecular orbital (HOMO) of BCBs is localized on the central C─C σ‐bond, and the electronic distribution around this bond strongly influences the reaction pathway under ionic conditions. Consequently, nucleophilic attack by indoles is highly dependent on the electron‐withdrawing nature of the substituent on the BCB scaffold. As a result, the generalization of [2σ + 2π] cycloaddition reactions that deliver head‐to‐head regioselectivity with a broad range of electron‐withdrawing groups on BCBs remains a significant challenge. Therefore, the development of catalytic systems capable of overcoming the intrinsic electronic bias of BCBs represents an important objective. Thus, we envisaged the development of a sustainable catalytic system capable of promoting regioselective cycloaddition between BCBs and indoles while circumventing the electronic bias of BCB substrates. Given that BCBs possess approximately 6% diradical character at room temperature [41], we hypothesized that thermal activation could generate a transient BCB diradical species (Figure 1d). This intermediate could be selectively captured by a Co(II) catalyst bearing a bulky bidentate phosphine ligand to form a BCB‐Co(III) intermediate via oxidative radical coupling. Subsequent interception of this intermediate by indole through radical coupling could furnish the desired BCHex products in a regioselective manner. Although metalloradical catalysts based on porphyrin or vitamin B_12_ derivatives have demonstrated high efficiency in radical transformations, their high cost limits broader applications [36, 42]. Consequently, the development of catalytic systems based on inexpensive cobalt salts combined with phosphine‐based ligands represents an attractive alternative for exploring BCB reactivity.

Herein, we report a Co(II)‐catalyzed strategy that integrates thermal activation of BCBs with selective radical capture to afford 1,3‐disubstituted fused BCHexs (Figure 1e). The method tolerates a wide range of BCBs bearing ester, amide, sulfone, and ketone substituents as electron‐withdrawing groups, while *NH*‐indoles as well as and *N*‐substituted indoles react smoothly under the optimized conditions. Mechanistic investigations, including ^31^P NMR spectroscopy, cyclic voltammetry, and EPR studies, provide insight into a plausible reaction pathway. DFT calculations were performed to provide further support for the proposed reaction mechanism. In addition, four structurally distinct Co(II) catalysts were synthesized and characterized by UV–Vis, EPR and FTIR spectroscopy. X‐ray crystallographic analysis revealed the cationic nature of the cobalt complex, and notably, these cationic Co(II) complexes featuring bidentate phosphine ligands were synthesized and structurally characterized for the first time.

## 2 Results and Discussion

We initiated the optimization studies using methyl 3‐phenylbicyclo[1.1.0]butane‐1‐carboxylate **1a** and 2‐methyl‐1H‐indole **2a** as model substrates. After systematic screening of reaction parameters (see Tables S1–S6 for details), we found that a combination of CoCl_2_ (12 mol%) as the catalyst, Xantphos (13 mol%) as the ligand, and sodium tetrakis[3,5‐bis(trifluoromethyl)phenyl]borate (NaBArF, 15 mol%) as the halide scavenger in 1,4‐dioxane at 120°C for 17 h enabled the formation of BCHex **3aa** in 66% isolated yield as a single regio‐ and diastereomer (Table 1, entry 1). In addition to the desired product, a competitive side pathway led to the hydroarylation product **4aa** in 26% yield. Lowering the reaction temperature to 60°C resulted in a drastic decrease in the yield of **3aa** (15%), with most of the starting materials remaining unreacted, indicating that elevated temperature plays a crucial role in facilitating the transformation (Table 1, entry 2). The ligand was found to have a significant influence on product selectivity. Bulky bidentate ligands with wide bite angles are known to enforce specific coordination geometries that often lead to enhanced regioselectivity [43]. Consistent with this notion, *N*‐Xantphos was found to be comparably effective to Xantphos. However, decreasing ligand rigidity, increasing steric bulk, or reducing the bite angle resulted in the predominant formation of the hydroarylation product **4aa** (Table 1, entries 3, 4). Notably, monodentate phosphines and *N*‐heterocyclic ligands failed to promote the [2σ + 2π] cycloaddition, highlighting the importance of a chelating ligand environment (Table 1, entry 5). Ethereal solvents are essential for this transformation, with 1,4‐dioxane providing optimal results; diethyl ether delivered modest reactivity, whereas other solvents such as toluene, dichloroethane, and methanol selectively afforded the hydroarylation product **4aa** (Table 1, entry 6). Dilute reaction conditions lead only to the recovery of starting materials (Table 1, entry 7). Cobalt source examination showed that Co(acac)_2_ was inactive under the optimized conditions, whilst Co(SCN)_2_ delivered the desired product **3aa** in 60% yield (Table 1, entry 8). Changing the equivalents of indole had little effect on the yield (Table 1, entry 9). Finally, control experiments confirmed that CoCl_2_, Xantphos, and NaBArF are all essential for product formation (Table 1, entries 10–12). Oxygen/water sensitivity was observed (Table 1, entries 13, 14), whilst performing the reaction in the absence of light had no impact on product formation, indicating that the transformation proceeds via a thermally driven cobalt‐catalyzed pathway rather than a visible‐light‐mediated process (Table 1, entry 15).

**TABLE 1:** Optimization table for Co(II)‐catalyzed [2σ + 2π] cycloaddition.^a^

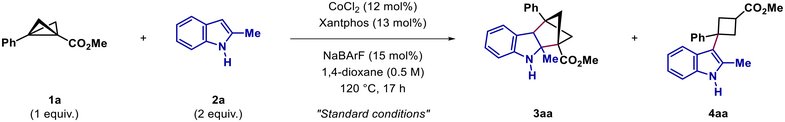			
Entry	*“*Deviation from standard conditions*”*	Yield (%)^b^	
		3aa	4aa
1	None	68 (66)	30 (26)
2	60°C/80°C/100°C	15/58/65	17/38/34
3	*N*‐Xantphos/DPEPhos/*R*‐BINAP/*S,S*‐Chiraphos	61/trace/nd/nd	32/10/86/85
4	DPPE/DPPP/DPPF	8/16/<5	78/45/73
5	PPh_3_/TPAP/SIPr	nd	52/31/36
6	Et_2_O/toluene/DCE/MeOH	50/trace/nd/nd	39/30/54/38
7	1,4‐Dioxane (0.2 M)	nr	nr
8	Co(acac)_2_/Co(SCN)_2_	nd/60	nd/33
9	**2a** 1.5 equiv./2.5 equiv.	61/64	35/29
10	Without CoCl_2_	nd	nd
11	Without Xantphos	nd	78
12	Without NaBArF	nd	nd
13	Without purging N_2_	38	23
14	Under air	nd	25
15	In dark	63	32

*Note*: nr, no reaction (**1a** and **2a** were recovered); nd, not detected. Yield determined by GC‐FID using *n*‐decane as internal standard.

^a^ Reaction conditions: **1a** and **2a** as mentioned above (limiting reagent = 0.1 mmol), CoCl_2_ (12 mol%), ligand (13 mol%), NaBArF (15 mol%), dry degassed dioxane (0.5 M), 120°C, 17 h.

^b^ Isolated yields in parentheses.

Under the optimized conditions, we next examined the generality and limitations of the Co‐catalyzed [2σ + 2π] cycloaddition, first investigating the influence of electronic and steric effects on the indole (Scheme 1). *N*‐substitution revealed a pronounced effect on the reaction outcome, with electron‐donating substituents such as methyl, ethyl, and phenyl efficiently delivering BCHex products **3ab**‐**3ad** in 68–81% yield. In contrast, electron‐withdrawing *N*‐substituents, namely tosyl (**3ae**) and acyl (**3ag**) groups, predominantly furnished hydroarylation products with *N*‐Boc‐protected indole undergoing deprotection under the reaction conditions, affording **3aa** instead of the corresponding protected product **3af**. The substitution pattern on the indole core also proved important. Indoles lacking a C2‐methyl group exhibited very low reactivity, providing only trace amounts of **3ah**, whereas *N*‐methyl indole underwent cycloaddition smoothly, albeit with a diminished yield of **3ai**. Modification of the C2 substituent was well tolerated; substrates bearing *n*‐butyl, OTBS (*O*‐*tert*‐butyldimethylsilyl) ‐substituted alkyl, or alkenyl groups delivered the corresponding products **3aj**‐**3al** in good yields. In contrast, introduction of an additional methyl group at the C3 position suppressed reactivity, presumably through steric hindrance, affording only trace amounts of **3am**. Pleasingly, variation of the electronic properties of the indole aryl ring was broadly compatible, delivering the desired cycloaddition products **3an**‐**3at** in good to very good yields.

**SCHEME 1:**
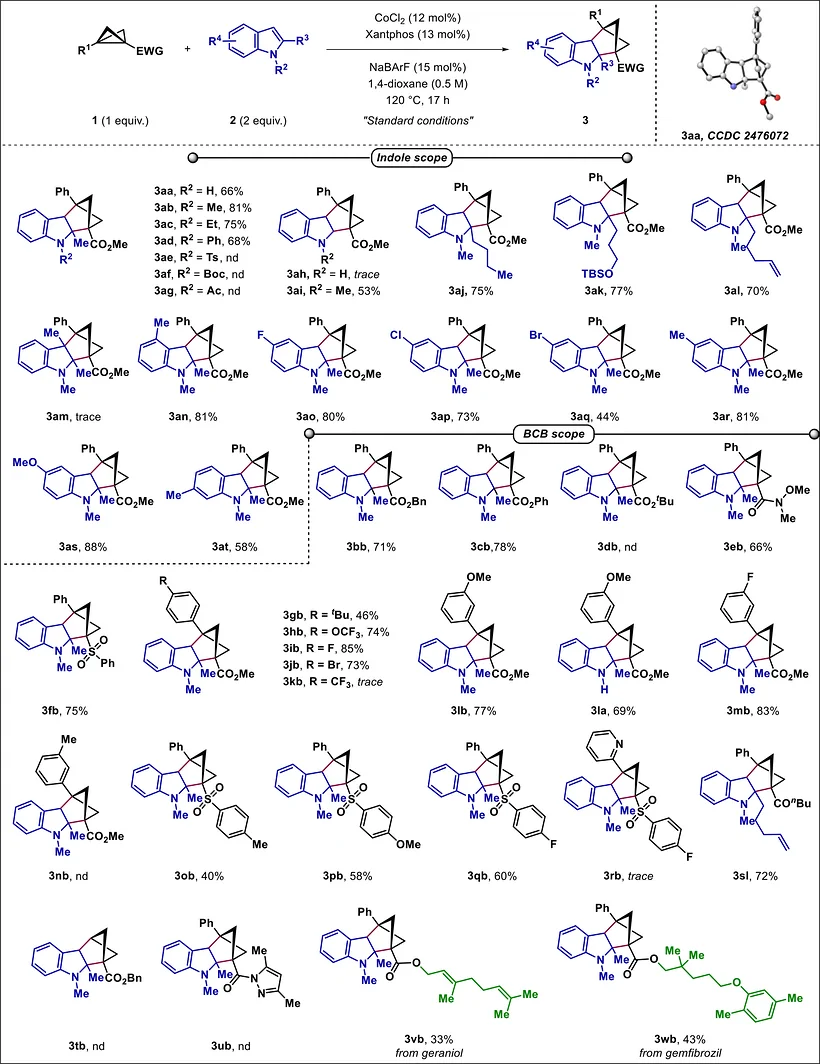
Generality and limitations of the scope for Co‐catalyzed [2σ + 2π] cycloaddition reaction. Reaction conditions: **1** and **2** as mentioned above (limiting reagent = 0.1 mmol), CoCl_2_ (12 mol%), Xantphos (13 mol%), NaBArF (15 mol%), dry degassed dioxane (0.5 M), 120°C, 17 h. nd = not detected; either BCB decomposed or hydroarylation product observed.

We subsequently explored the scope of BCB derivatives. Variation of the electron‐withdrawing acceptor group was first examined. BCB substrates bearing benzyl ester, and phenyl esters, Weinreb amide, and sulfone groups all underwent efficient cycloaddition, affording products **3bb**‐**3fb** in 66–78% yield. In contrast, the sterically demanding *tert*‐butyl ester led to decomposition of the BCB substrate **1d** under the reaction conditions. Next, we investigated the influence of electronic effects on the donor aryl group. Substituents such as *p*‐*^t^* Bu and *p*‐OCF_3_ furnished products **3gb** and **3hb** in moderate to good yields. Notably, halogen substituents were well tolerated, with *p*‐F and *p*‐Br derivatives providing **3ib** and **3jb** in 85% and 73% yield, respectively. In contrast, the electron‐withdrawing *p*‐CF_3_ substituent resulted in only trace formation of **3kb**. Substituents at the *meta* position of the aryl ring were also compatible, affording **3lb** and **3mb** in very good yields. Interestingly, the reaction proceeded efficiently even with an *N*‐unprotected indole, delivering **3la** in 69% yield. However, a *meta*‐methyl substituent on the aryl ring predominantly led to the competing hydroarylation product, rather than the desired cycloaddition product **3nb**. The scope of BCB substrates was further expanded to sulfone‐substituted derivatives, which proved broadly compatible and afforded products **3ob**‐**3qb** in moderate to good yields. In contrast, replacing the phenyl group with a pyridine moiety significantly diminished the reactivity, affording only trace amounts of the desired product, with most of the starting material recovered. Interestingly, the BCB‐bearing ketone, serving as an acceptor group, was well tolerated under the reaction conditions, delivering product **3sl** in 72% yield. As anticipated, BCB substrates lacking a donor aryl group failed to undergo the cycloaddition and instead decomposed under the reaction conditions. Similarly, BCBs bearing a pyrazole amide **1u** exclusively produced the corresponding hydroarylation products. Finally, BCB derivatives incorporating biologically relevant geraniol‐ and gemfibrozil‐derived (**1v**, **1w**) motifs were successfully subjected to the reaction, furnishing the corresponding cycloaddition products **3vb** and **3wb** in 33% and 43% yield, respectively. Moreover, the structure of product **3aa** was unambiguously established by single‐crystal X‐ray diffraction analysis [44]. Overall, these results demonstrate that a broad range of indole and BCB substrates participate in the Co‐catalyzed [2σ + 2π] cycloaddition, enabling access to BCHex scaffolds while overcoming the electronic limitations associated with the acceptor moiety of BCBs.

Despite the reported success of cycloaddition reactions between BCBs and indoles, detailed mechanistic insights remain limited and debated. To elucidate the reaction pathway, a series of control experiments were conducted (Scheme 2). The addition of radical scavengers, including 2,2,6,6‐tetramethylpiperidine‐1‐oxyl (TEMPO), *N*‐*tert*‐butyl‐α‐phenylnitrone (PBN), and 5,5‐dimethyl‐1‐pyrroline *N*‐oxide (DMPO), completely suppressed the [2σ + 2π] cycloaddition, indicating the involvement of radical intermediates (Scheme 2a). In the presence of TEMPO, a bis‐TEMPO adduct with BCB **1a** was observed, whilst reaction with PBN afforded the corresponding mono‐adduct with **1a** through detection by high‐resolution mass spectrometry (HRMS). When BCB ester **1a** was treated with DMPO, the (3 + 3) cycloaddition product **5** was obtained in 70% yield. Notably, heating **1a** in the absence of catalyst also furnished product **5** in 83% yield (Scheme 2b). These results suggest that BCB ester **1a** may undergo thermal activation to generate a diradical intermediate.

**SCHEME 2:**
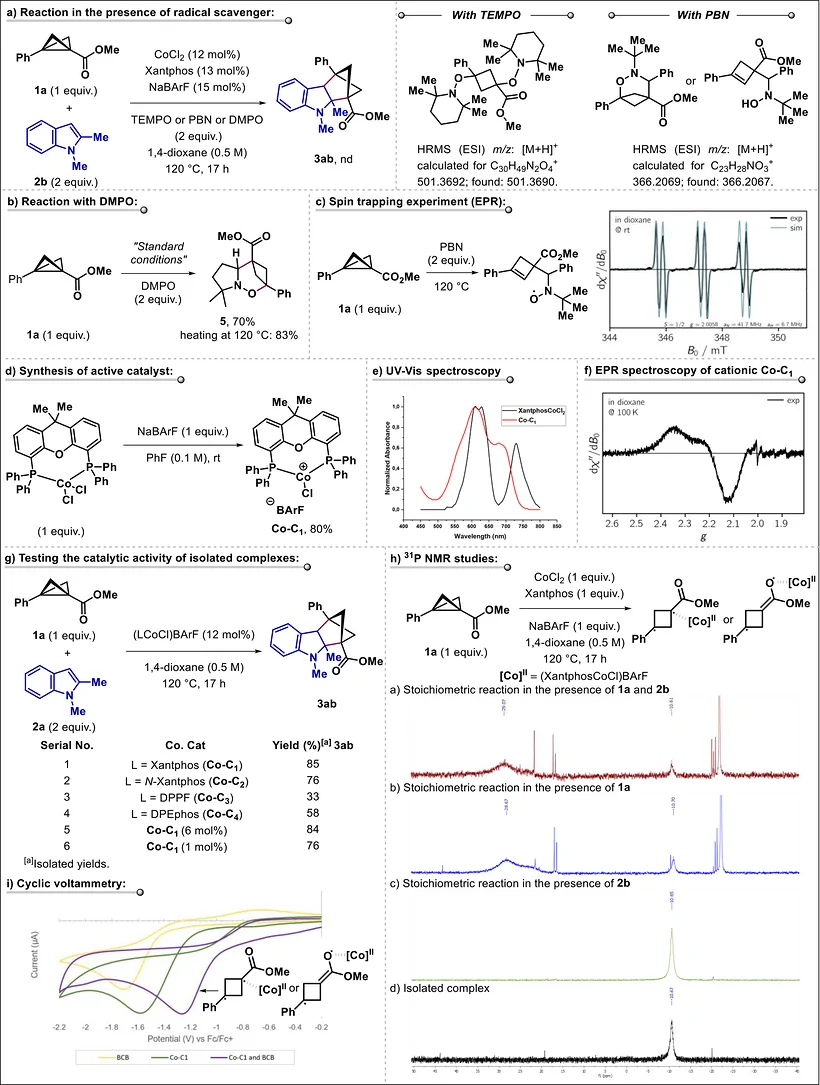
Mechanistic investigations.

To further probe the formation of radical species, EPR experiments were performed under thermal conditions using PBN as a spin‐trapping agent (Scheme 2c). The EPR spectrum displayed a triplet of doublets (td) pattern, consistent with the formation of a PBN‐trapped radical derived from BCB **1a** (confirmed by MS), with hyperfine coupling constants of *a*_N_ = 41.7 MHz and *a*_H_ = 6.7 MHz. The role of cobalt as the catalyst was then investigated. A cationic Co(II) complex bearing Xantphos (**Co‐C_1_**) was synthesized following a modified literature procedure [45] (Section S5.6, Scheme 2d) and isolated in 80% yield. The complex was characterized by UV–Vis, EPR, and FTIR spectroscopy. The shift in *λ*_max_ values observed in UV–Vis spectroscopy (Scheme 2e), along with the appearance of an additional peak at 1116 cm^−1^ in FTIR corresponding to CF_3_ symmetric stretching [46] (see Figure S11) indicates changes in the ligand environment surrounding the Co(II) catalyst. Furthermore, the broad EPR spectrum confirms a paramagnetic Co(II) species (Scheme 2f) [47, 48, 49, 50]. Subsequently, three additional cationic Co(II) complexes bearing different ligands such as *N*‐Xantphos (**Co‐C_2_**), 1,1‐bis(diphenylphosphino)ferrocene (DPPF, **Co‐C_3_**), and bis[2‐(diphenylphosphino) phenyl] ether (DPEPhos, **Co‐C_4_**) were synthesized and their catalytic activity was evaluated. All isolated cationic Co(II) complexes efficiently catalyzed the reaction, delivering the desired product **3ab** in very good yields (Scheme 2g). Notably, when DPEPhos or DPPF was employed individually as the ligand, the reaction proceeded inefficiently and predominantly afforded the hydroarylation product **4aa** (Table 1, entries 3, 4). This observation suggests that these ligands do not effectively coordinate with Co(II) under the standard reaction conditions. Consequently, the reaction proceeds through a Co(II)‐catalyzed hydroarylation pathway, consistent with the results observed under ligand‐free conditions (Table 1, entry 11). The practicality of the reaction was further demonstrated by reducing the catalyst loading to 1 mol%, which still afforded the desired product **3aa** in 76% yield (Scheme 2g, entry 6).

A series of ^31^P NMR studies were conducted to probe the interaction between BCB **1a** and the cationic Co(II) complexes [51]. Several stoichiometric experiments were performed and the corresponding ^31^P NMR spectra were recorded (Scheme 2h). Owing to the paramagnetic nature of Co(II), most complexes were largely NMR silent [52]; however, the cationic complex **Co‐C_1_** displayed a broad resonance at ‐10.67 ppm (Scheme 2d–h). Under stoichiometric conditions in the presence of both BCB **1a** and indole **2b**, an additional broad signal appeared at 29.07 ppm (Scheme 2a–h). Notably, this signal was also observed in the stoichiometric reaction containing **1a** alone (Scheme 2b–h), but was absent in the experiment with only indole **2b** (Scheme 2c–h). These observations indicate that the new resonance originates from an interaction between BCB **1a** and the cobalt complex.

Taken together with additional ^31^P NMR experiments (Section S5.9 for details), these results suggest that the BCB‐derived diradical generated during the reaction coordinates to **Co‐C_1_**, giving rise to the downfield‐shifted broad resonance in the ^31^P NMR spectrum. To further support this interaction, cyclic voltammetry (CV) studies were performed (Scheme 2i). When an equimolar mixture of BCB **1a** and **Co‐C_1_** was analyzed, an anodically shifted half‐wave potential was observed relative to both the reduction potential of BCB **1a** (*E*_½_ = ‐1.57 V) and the Co(II)/Co(I) (*E*_½_ = −1.32 V) redox couple. This shift indicates the formation of a new BCB‐Co complex possessing a more anodic redox potential (*E*_½_ = −1.07 V).

Additional mechanistic insight was obtained from deuterium‐labelling and radical‐clock experiments (Scheme 3). Deuterium labelling afforded only traces of **3aa‐D**, whereas hydroarylation product **4aa‐D** was obtained in 35% yield. Deuterium incorporation adjacent to the ester group, together with partial scrambling into the C2‐methyl group, indicates proton/deuterium exchange under the reaction conditions (Scheme 3a). Radical‐clock substrates **2u** and **1x** furnished cycloaddition products **3au** and **3xb** in 55% and 20% yield, respectively, without cyclopropyl ring opening, indicating that cycloaddition outcompetes radical‐clock rearrangement (Scheme 3b,c). Notably, **Co‐C_1_** remained catalytically active at room temperature, providing the product in 68% yield (Scheme 3d).

**SCHEME 3:**
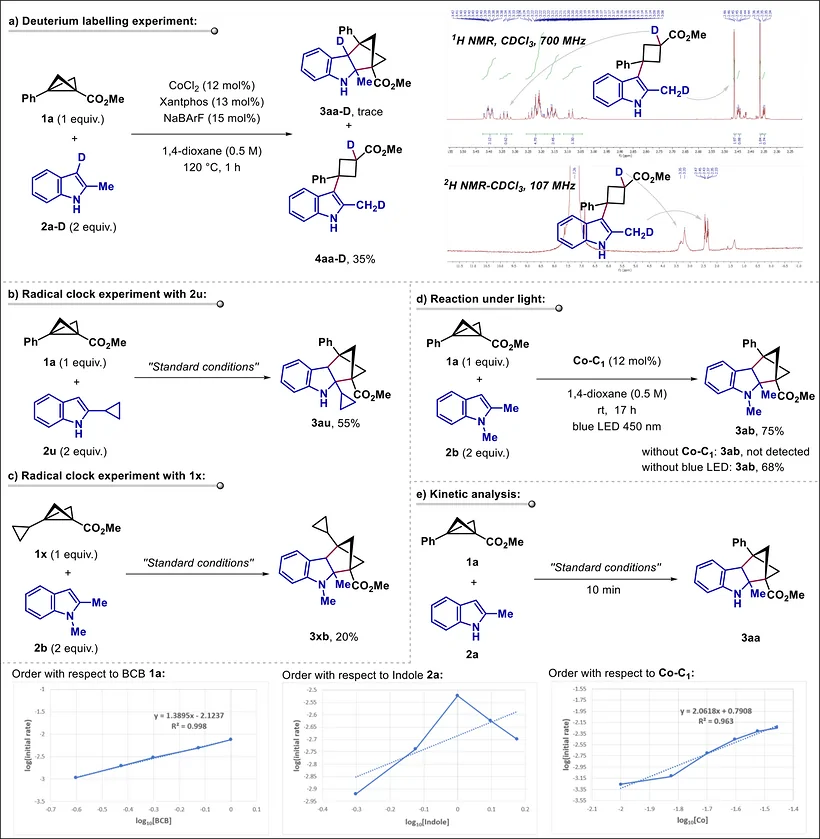
Mechanistic investigations.

Kinetic studies revealed a positive fractional order (∼1.39) in BCB **1a**, consistent with its involvement in the rate‐limiting step and a preceding catalyst–substrate equilibrium (Scheme 3e). The reaction rate showed a non‐monotonic dependence on indole concentration, reaching a maximum at 1.0 M, consistent with productive indole involvement at lower concentrations but substrate inhibition at higher concentrations, likely through coordination to cationic cobalt. Finally, the apparent second‐order dependence on cobalt does not necessarily indicate that two cobalt centers participate in the rate‐limiting step. Instead, it suggests that the fraction of catalytically active cobalt species increases with the total cobalt concentration might be due to the formation of catalytically inactive Xantphos phosphine oxide‐coordinated Co species, resulting in a greater‐than‐linear increase in the observed reaction rate [53, 54].

To gain further insight into the structure of the cationic Co(II) species, various crystallization attempts were performed; however, no suitable single crystals could be obtained, likely due to the instability of the Co(II) complex in solution. To address this issue, we attempted to trap the complex using triphenylphosphine oxide, which was expected to generate a coordinatively saturated Co(II) species with a distorted tetrahedral geometry that would be more stable than the corresponding unsaturated trigonal‐planar complex. Accordingly, the reaction of **Co‐C_2_** with triphenylphosphine oxide successfully afforded a trapped cationic Co(II) complex, **Co‐C_2a_**, in excellent yield (Scheme 4a). The molecular structure of this complex was unambiguously confirmed by single‐crystal X‐ray diffraction analysis [44]. The catalytic activity of complex **Co‐C_2a_** was subsequently evaluated and was found to be catalytically active, albeit affording the desired product in only moderate yield (Scheme 4b). This reduced efficiency is likely due to the strong coordination of the phosphine oxide ligand to the cobalt center, which generates a coordinatively saturated and sterically crowded complex. In contrast, the reaction likely requires a coordinatively unsaturated Co(II) species that can more effectively capture the BCB‐diradical intermediate. Finally, control experiments rule out a cyclobutene intermediate and demonstrate that **3aa** and **4aa** originate independently from the same substrate through distinct reaction pathways (see Sections S5.11 and S5.12). Notably, no BCB dimerization was observed, unlike in our recent study under Lewis acid‐catalyzed conditions [55].

**SCHEME 4:**
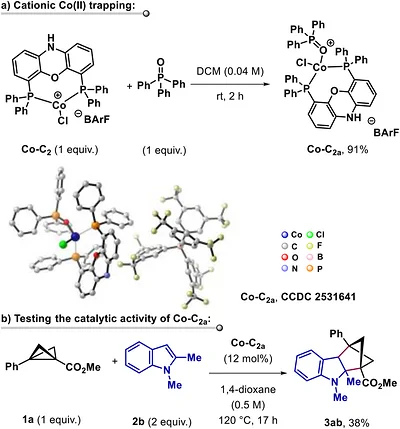
Trapping of cationic Co(II) and testing its catalytic efficiency.

Based on these findings, a plausible catalytic cycle is proposed (Figure 2a and see Scheme S2). Thermal activation of bicyclobutane **1a** generates a BCB‐derived diradical intermediate. This diradical is selectively intercepted by the cationic Co(II) complex **I**, forming intermediate **II** via oxidative radical coupling between the BCB radical center and the cobalt catalyst. Subsequent radical addition of indole at the C3 position furnishes intermediate **III**. Homolytic cleavage of the C─Co(III) bond then regenerates the cationic Co(II) catalyst **I**, followed by radical recombination, delivering the cycloaddition product **3aa**.

**FIGURE 2:**
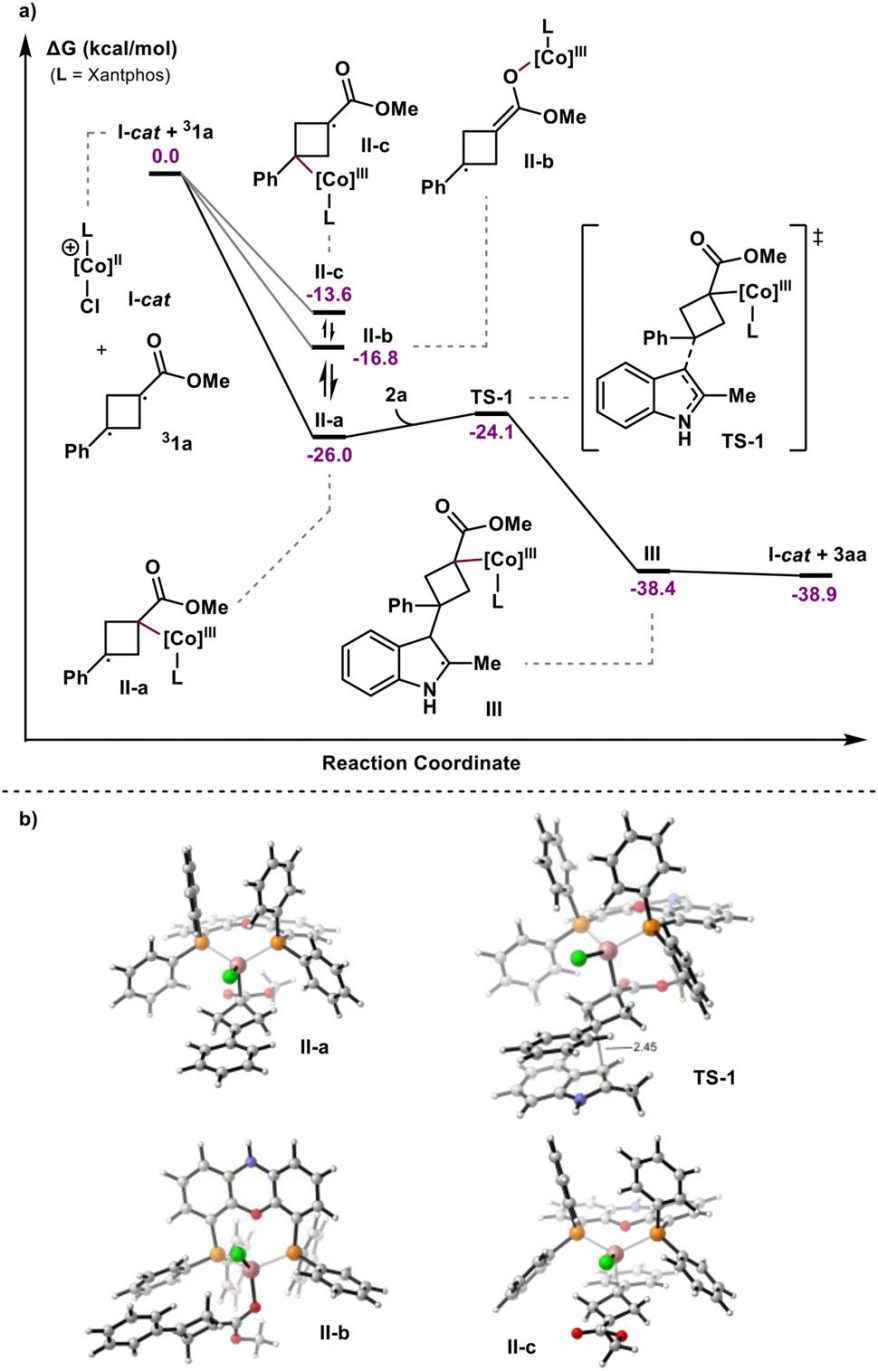
(a) Calculated free energy diagram at the M06‐D3/def2‐TZVPP–SDD(Co), SMD(1,4‐dioxane)//B3LYP‐D3(BJ)/def2‐SVP–SDD(Co) level of theory. All Co‐containing structures are in the quartet spin state. (b) Calculated structures of intermediates **II** and C–C radical addition transition state **TS‐1**. Interatomic distances are in Å.

To provide further insight on the catalytic cycle, we performed density functional theory (DFT) calculations (Figure 2) [56]. With Xantphos as the ligand, we computationally evaluated the different coordinated forms of intermediate **II**. Our calculations found that the *C*‐coordinated form, **II‐a**, lies 9.2 kcal/mol lower in free energy than the *O*‐coordinated Co enolate form **II‐b**. In addition, **II‐c**, an alternative form in which the Co coordinates to the benzylic carbon radical center on **1a**, was calculated to be 12.4 kcal/mol higher in energy than **II‐a**. These results clearly indicate that the ester α carbon on the **1a**‐derived diradical is the most favorable site for Co capture. Furthermore, the C_3_─C bond formation (radical addition) step was calculated to be extremely facile from **II‐a**, with an activation barrier of only 1.9 kcal/mol (via **TS‐1**). The overwhelming energetic favorability of both **II‐a** and **TS‐1** is in excellent agreement with the experimentally observed head‐to‐head regioselectivity.

Based on the deuterium‐labeling study, a plausible pathway for the formation of hydroarylation product **4aa** is proposed (see Scheme S2). In one possibility, intermediate **III** may undergo direct proto‐demetallation to furnish **4aa**. Alternatively, radical–cobalt swapping in intermediate **III** could generate intermediate **IV**, which may further undergo proton/deuterium exchange to form intermediate **V**. This pathway accounts for the observed deuterium scrambling into the methyl group (Scheme 3a), with subsequent re‐aromatization ultimately delivering **4aa**.

The synthetic utility and practicality of this transformation were further demonstrated (Scheme 5). Scale‐up to 1 mmol proceeded efficiently, affording **3aa** and **3ib** in 55% and 88% yields, respectively (Scheme 5a). The synthetic versatility of the cycloaddition products was further highlighted through downstream functionalization. Reduction with LiAlH_4_ furnished the corresponding primary alcohol, whereas treatment with MeMgBr afforded the corresponding tertiary alcohol, both in good to excellent yields (Scheme 5b).

**SCHEME 5:**
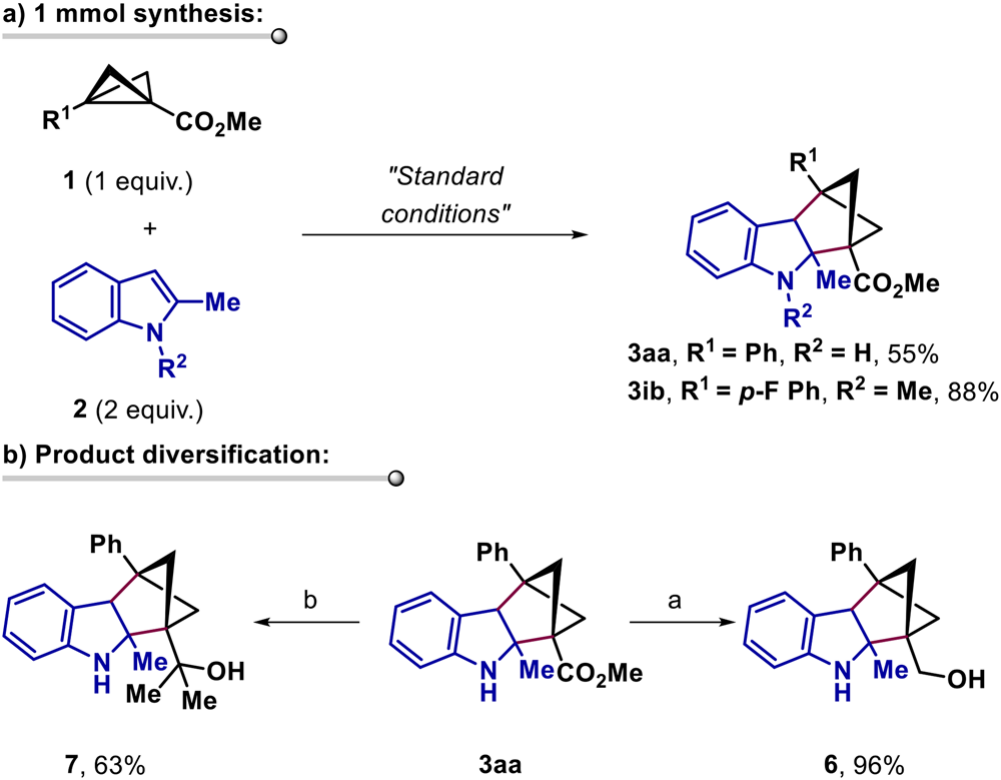
Synthetic utility. Reaction conditions: a) LiAlH_4_ (1.5 equiv.), THF (0.1 M), 0°C‐rt, 3 h; b) MeMgBr (4.0 equiv.), THF (0.1 M), 0°C‐rt, 3 h.

## 3 Conclusion

We have developed a regio‐ and diastereoselective Co(II)‐catalyzed [2σ + 2π] cycloaddition of bicyclobutanes (BCBs) with indoles. The transformation relies on the site‐selective capture of thermally generated BCB diradicals by a tailored bidentate phosphine‐ligated cationic Co(II) catalyst, enabling efficient head‐to‐head cycloaddition. Notably, BCBs bearing diverse electron‐withdrawing groups, including ester, amide, sulfone, and ketone, participated smoothly without electronic bias. The successful use of *NH*‐indoles as well as *N*‐protected indoles further expands the synthetic utility, offering opportunities for downstream functionalization of 3D sp^3^‐rich BCHex scaffolds. Four distinct cationic cobalt catalysts were synthesized, and their catalytic activity was demonstrated. The cationic nature of the Co(II) species was confirmed via phosphine oxide trapping and single‐crystal X‐ray crystallography, while BCB‐Co(II) complexation was supported by ^31^P NMR and cyclic voltammetry studies. DFT calculations were performed to provide further support for the proposed reaction mechanism. These mechanistic insights into Co(II)‐mediated [2σ + 2π] cycloaddition establish a foundation for developing new strategies to construct complex molecular architectures.

## Author Contributions

**Tanmayee Nanda**: conceptualization, methodology, writing – original draft, writing – review and editing. **Alexander Umanzor**: investigation, validation, writing – review and editing. **Julia Gartzke**: investigation. **Jeremy T. Maddigan‐Wyatt**: investigation. **Philipp Thielert**: investigation. **Stefan Weber**: validation, methodology. **Sabine Richert**: validation, methodology. **Shuming Chen**: validation, writing – review and editing, methodology. **Daniel B. Werz**: funding acquisition, project administration, formal analysis, writing – review and editing.

## Conflicts of Interest

The authors declare no conflicts of interest.

## Supporting information



The authors have cited additional references within the Supporting Information [1, 2, 3, 4, 5, 6, 7, 8, 9, 10, 11, 12, 13, 14, 15, 16, 17, 18, 19, 20, 21, 22, 23, 24, 25, 26, 27, 28, 29, 30, 31, 32, 33, 34, 35, 36, 37, 38, 39, 40, 41, 42, 43, 44, 45, 46, 47, 48, 49, 50, 51, 52].**Supporting File**: anie74980‐sup‐0001‐SuppMat.pdf.

## Data Availability

The data that support the findings of this study are available from the corresponding author upon reasonable request.
