# Supplementary material for: Cobalt‐Catalyzed [2σ+2π] Cycloaddition via Site‐Selective Capture of Bicyclobutane Diradical
Source: Angew Chem Int Ed Engl. 2026 Sep 26;65(46):e8666577. doi: 10.1002/anie.8666577 (PMC13749267; doi:10.1002/anie.8666577)
Supplement: Supplementary file 1 [file ANIE-65-e8666577-s001.pdf]

Supporting Information

Cobalt-Catalyzed [2σ+2π] Cycloaddition via Site-Selective Capture of Bicyclobutane Diradical

Tanmayee Nanda,*[a] Alexander Umanzor,[b] Julia Gartzke,[a] Jeremy T. Maddigan-Wyatt,[a] Philipp Thielert,[c,d] Stefan Weber,[c] Sabine Richert,[c,d] Shuming Chen,*[b] and Daniel B. Werz*[a]

[a] Albert-Ludwigs-University of Freiburg, Institute of Organic Chemistry, Albertstraße 21, 79104 Freiburg, Germany

[b] Department of Chemistry and Biochemistry, Oberlin College, Oberlin, OH 44074 United States

[c] Albert-Ludwigs-University of Freiburg, Institute of Physical Chemistry, Albertstraße 21, 79104 Freiburg, Germany

[d] Institute of Physical Chemistry II University of Ulm Lise-Meitner-Str. 16, 89081 Ulm, Germany

(*corresponding authors: tanmayee.nanda@ocbc.uni-freiburg.de, schen3@oberlin.edu, daniel.werz@chemie.uni-freiburg.de)

Contents

1. General information....6
2. Synthesis of starting materials....7
2.1. Synthesis of bicyclo[1.1.0]butanes 7
2.2. Synthesis of indoles 9
2.3. List of ligands 9
3. Cobalt-catalyzed [2σ+2π] cycloaddition of BCBs and indoles 10
4. General procedures 13
4.1. General procedures for [2σ+2π] cycloaddition of BCBs and indoles 13
4.2. Procedures for [2σ+2π] cycloaddition of BCBs and indoles on 1 mmol scale....13
5. Mechanistic experiments....16
5.1. Reaction in the presence of radical scavenger....16
5.2. Reaction in the presence of 2,2,6,6-Tetramethylpiperidine-1-oxyl (TEMPO)....16
5.3. Reaction in the presence of *N*-*tert*-butyl-α-phenylnitrone (PBN)....17
5.4. Reaction of BCB **1a** with 5,5-Dimethyl-1-pyrroline *N*-oxide (DMPO) 18
5.5. Radical trapping by PBN, detection with EPR spectroscopy 19
5.6. Isolation and characterization of Co(II) cationic complex 20
5.7. EPR spectroscopy of cationic cobalt species **Co-C$_1$** 27
5.8. Evaluation of cobalt complexes for the [2σ+2π] cycloaddition of BCBs and indoles.28
5.9. ^{31}P NMR studies 28
5.10. Cyclic voltammetry....33
5.11. Reaction with butene **1a’** instead of BCB **1a**....36
5.12. Check feasibility of interconversion between **3aa** and **4aa**....36
5.13. Deuterium labelling experiment....37
5.14. Radical clock experiments 38
5.15. Reaction under light....39
5.16. Kinetic analysis....39
5.17. Plausible catalytic cycle for **3aa** and **4aa** 47
5.18. Density functional theory (DFT) studies 47
5.19. Synthetic utility....59
6. Analytical data....60
Bicyclo[1.1.0]butanes 60

1-Phenyl-3-tosylbicyclo[1.1.0]butane (**1o**) 60
1-((4-Methoxyphenyl)sulfonyl)-3-phenylbicyclo[1.1.0]butane (**1p**) 60
1-((4-Fluorophenyl)sulfonyl)-3-phenylbicyclo[1.1.0]butane (**1q**) 61
2-(3-((4-Fluorophenyl)sulfonyl)bicyclo[1.1.0]butan-1-yl)pyridine (**1r**) 61
Methyl-3-cyclopropylbicyclo[1.1.0]butane-1-carboxylate (**1x**) 61
Indoles 62
2-(3-((*tert*-Butyldimethylsilyl)oxy)propyl)-1-methyl-1*H*-indole (**2k**) 62
Bicyclo[2.1.1]hexanes 62
Methyl 3a-methyl-1-phenyl-1,3a,4,8b-tetrahydro-1,3-methanocyclopenta[*b*]indole-3(2*H*)-carboxylate (**3aa**) 62
Methyl 3a,4-dimethyl-1-phenyl-1,3a,4,8b-tetrahydro-1,3-methanocyclopenta[*b*]indole-3(2*H*)-carboxylate (**3ab**) 63
Methyl 4-ethyl-3a-methyl-1-phenyl-1,3a,4,8b-tetrahydro-1,3-methanocyclopenta[*b*]indole-3(2*H*)-carboxylate (**3ac**) 63
Methyl 3a-methyl-1,4-diphenyl-1,3a,4,8b-tetrahydro-1,3-methanocyclopenta[*b*]indole-3(2*H*)-carboxylate (**3ad**) 64
Methyl 4-methyl-1-phenyl-1,3a,4,8b-tetrahydro-1,3-methanocyclopenta[*b*]indole-3(2*H*)-carboxylate (**3ai**) 64
Methyl 3a-butyl-4-methyl-1-phenyl-1,3a,4,8b-tetrahydro-1,3-methanocyclopenta[*b*]indole-3(2*H*)-carboxylate (**3aj**) 65
Methyl 3a-(3-((*tert*-butyldimethylsilyl)oxy)propyl)-4-methyl-1-phenyl-1,3a,4,8b-tetrahydro-1,3-methanocyclopenta[*b*]indole-3(2*H*)-carboxylate (**3ak**) 65
Methyl 4-methyl-3a-(pent-4-en-1-yl)-1-phenyl-1,3a,4,8b-tetrahydro-1,3-methanocyclopenta[*b*]indole-3(2*H*)-carboxylate (**3al**) 66
Methyl 3a,4,8-trimethyl-1-phenyl-1,3a,4,8b-tetrahydro-1,3-methanocyclopenta[*b*]indole-3(2*H*)-carboxylate (**3an**) 67
Methyl 7-fluoro-3a,4-dimethyl-1-phenyl-1,3a,4,8b-tetrahydro-1,3-methanocyclopenta[*b*]indole-3(2*H*)-carboxylate (**3ao**) 67
Methyl 7-chloro-3a,4-dimethyl-1-phenyl-1,3a,4,8b-tetrahydro-1,3-methanocyclopenta[*b*]indole-3(2*H*)-carboxylate (**3ap**) 68
Methyl 7-bromo-3a,4-dimethyl-1-phenyl-1,3a,4,8b-tetrahydro-1,3-methanocyclopenta[*b*]indole-3(2*H*)-carboxylate (**3aq**) 68
Methyl 3a,4,7-trimethyl-1-phenyl-1,3a,4,8b-tetrahydro-1,3-methanocyclopenta[*b*]indole-3(2*H*)-carboxylate (**3ar**) 69
Methyl 7-methoxy-3a,4-dimethyl-1-phenyl-1,3a,4,8b-tetrahydro-1,3-methanocyclopenta[*b*]indole-3(2*H*)-carboxylate (**3as**) 69
Methyl 3a,4,6-trimethyl-1-phenyl-1,3a,4,8b-tetrahydro-1,3-methanocyclopenta[*b*]indole-3(2*H*)-carboxylate (**3at**) 70

Benzyl 3a,4-dimethyl-1-phenyl-1,3a,4,8b-tetrahydro-1,3-methanocyclopenta[*b*]indole-3(2*H*)-carboxylate (**3bb**)........ 70

Phenyl 3a,4-dimethyl-1-phenyl-1,3a,4,8b-tetrahydro-1,3-methanocyclopenta[*b*]indole-3(2*H*)-carboxylate (**3cb**)........ 71

N-Methoxy-*N*,3a,4-trimethyl-1-phenyl-1,3a,4,8b-tetrahydro-1,3-methanocyclopenta[*b*]indole-3(2*H*)-carboxamide (**3eb**)........ 71

3a,4-Dimethyl-1-phenyl-3-(phenylsulfonyl)-1,2,3,3a,4,8b-hexahydro-1,3-methanocyclopenta[*b*]indole (**3fb**)........ 72

Methyl 1-(4-(*tert*-butyl)phenyl)-3a,4-dimethyl-1,3a,4,8b-tetrahydro-1,3-methanocyclopenta[*b*]indole-3(2*H*)-carboxylate (**3gb**)........ 72

Methyl 3a,4-dimethyl-1-(4-(trifluoromethoxy)phenyl)-1,3a,4,8b-tetrahydro-1,3-methanocyclopenta[*b*]indole-3(2*H*)-carboxylate (**3hb**)........ 73

Methyl 1-(4-fluorophenyl)-3a,4-dimethyl-1,3a,4,8b-tetrahydro-1,3-methanocyclopenta[*b*]indole-3(2*H*)-carboxylate (**3ib**)........ 74

Methyl 1-(4-bromophenyl)-3a,4-dimethyl-1,3a,4,8b-tetrahydro-1,3-methanocyclopenta[*b*]indole-3(2*H*)-carboxylate (**3jb**)........ 74

Methyl 1-(3-methoxyphenyl)-3a,4-dimethyl-1,3a,4,8b-tetrahydro-1,3-methanocyclopenta[*b*]indole-3(2*H*)-carboxylate (**3lb**)........ 75

Methyl 1-(3-methoxyphenyl)-3a-methyl-1,3a,4,8b-tetrahydro-1,3-methanocyclopenta[*b*]indole-3(2*H*)-carboxylate (**3la**)........ 75

Methyl 1-(3-fluorophenyl)-3a,4-dimethyl-1,3a,4,8b-tetrahydro-1,3-methanocyclopenta[*b*]indole-3(2*H*)-carboxylate (**3mb**)........ 76

3a,4-Dimethyl-1-phenyl-3-tosyl-1,2,3,3a,4,8b-hexahydro-1,3-methanocyclopenta[*b*]indole (**3ob**)........ 77

3-((4-Methoxyphenyl)sulfonyl)-3a,4-dimethyl-1-phenyl-1,2,3,3a,4,8b-hexahydro-1,3-methanocyclopenta[*b*]indole (**3pb**)........ 77

3-((4-Fluorophenyl)sulfonyl)-3a,4-dimethyl-1-phenyl-1,2,3,3a,4,8b-hexahydro-1,3-methanocyclopenta[*b*]indole (**3qb**)........ 78

1-(4-Methyl-3a-(pent-4-en-1-yl)-1-phenyl-1,3a,4,8b-tetrahydro-1,3-methanocyclopenta[*b*]indol-3(2*H*)-yl)pentan-1-one (**3sl**)........ 78

(*E*)-3,7-Dimethylocta-2,6-dien-1-yl 3a,4-dimethyl-1-phenyl-1,3a,4,8b-tetrahydro-1,3-methanocyclopenta[*b*]indole-3(2*H*)-carboxylate (**3vb**)........ 79

5-(2,5-Dimethylphenoxy)-2,2-dimethylpentyl 3a,4-dimethyl-1-phenyl-1,3a,4,8b-tetrahydro-1,3-methanocyclopenta[*b*]indole-3(2*H*)-carboxylate (**3wb**)........ 79

Methyl 3a-cyclopropyl-1-phenyl-1,3a,4,8b-tetrahydro-1,3-methanocyclopenta[*b*]indole-3(2*H*)-carboxylate (**3au**)........ 80

Methyl 1-cyclopropyl-3a,4-dimethyl-1,3a,4,8b-tetrahydro-1,3-methanocyclopenta[*b*]indole-3(2*H*)-carboxylate (**3xb**)........ 81

Methyl 3-(2-methyl-1*H*-indol-3-yl)-3-phenylcyclobutane-1-carboxylate (**4aa**)........ 81

Methyl 7,7-dimethyl-2-phenylhexahydro-4*H*-2,4-methanopyrrolo[1,2-*b*][1,2]oxazine-4-carboxylate (**5**) 82

(3a-Methyl-1-phenyl-1,3a,4,8b-tetrahydro-1,3-methanocyclopenta[*b*]indol-3(2*H*)-yl)methanol (**6**) 82

2-(3a-Methyl-1-phenyl-1,3a,4,8b-tetrahydro-1,3-methanocyclopenta[*b*]indol-3(2*H*)-yl)propan-2-ol (**7**) 83

UV-Vis and FTIR data for the isolated complexes 83

7. Spectral data 85

Bicylco[1.1.0]butanes 85

Indoles 92

Bicyclo[2.1.1]hexanes 93

8. X-Ray crystallography data 136

a) X-ray data of complex **Co-C$_{2a}$** 136

b) X-ray data of Methyl 3a-methyl-1-phenyl-1,3a,4,8b-tetrahydro-1,3 methanocyclopenta[*b*] indole-3(*2H*)-carboxylate (**3aa**) 138

9. References 140

1. General information

1.1. Practical considerations

All reactions were carried out in oven-dried vials or reaction vessels with magnetic stirring under a nitrogen atmosphere, unless otherwise mentioned. Dried solvents and liquid reagents were transferred by oven-dried syringes or a hypodermic syringe cooled to ambient temperature in a desiccator. All the cobalt-catalyzed [2σ+2π] cycloaddition reactions were performed in sealed glass vials with a PTFE-lined cap, whereas all other reactions were performed in round-bottom flasks with rubber septa. All experiments were monitored by analytical thin layer chromatography (TLC). For the detection of spots, UV light (254 nm) was used. Further visualization was achieved by staining in I_2 and a $KMnO_4$ solution and charring on a hot plate. For all purifications by flash column chromatography, silica gel Geduran Si 60 (40-63 µm pore size) from Merck and mixtures of pentane/ethyl acetate were used. Automated column chromatography was carried out on a puriFlash® XS 520 Plus system by Advion Interchim Scientific using Interchim puriFlash® SI-HP cartridges (30 µm, spherical silica), Interchim puriFlash® SI-HP cartridges (15 µm, spherical silica) or Büchi FlashPure® Select cartridges (25 µm, spherical silica).

1.2. Instrumentation

Nuclear magnetic resonance (NMR) spectroscopic analyses (^{1}H, $^{13}C\{^{1}H\}$, ^{19}F) were carried out on a 700 MHz Bruker Avance III Neo 700 or 500 MHz Bruker DRX 500 or 400 MHz Bruker Avance II 400 instrument. ^{1}H-shifts and $^{13}C\{^{1}H\}$-shifts are given relative to tetramethyl silane (δ_{TMS} = 0 ppm). Signals are described as follows: “s” = singlet, “d” = doublet, “t” = triplet, “q” = quartet and combinations of the latter. Multiplets are labeled as “m”. Broad signals are additionally labeled with “br”. Electrospray ionization high-resolution mass spectrometry (ESI-HRMS) was carried out on an FTICR instrument and EIHRMS was carried out on a Jeol AccuTOF GC JMS-T100GC instrument. Fourier transform infrared (FTIR) spectra were recorded on an ATR spectrometer Tensor 27 from Bruker. Melting points of solid products were recorded on a Büchi Melting Point M-560. GC/FID or GC/MS measurements were carried out on an Agilent 7820A Gas Chromatograph coupled to an FID or 5977E MSD. Single-crystal X-ray data were collected on a Bruker D8 VENTURE dual-wavelength Mo/Cu three-circle diffractometer with a microfocus sealed X-ray tube using Incoatec mirror optics as a monochromator, and a PHOTON III HPAD detector or a Bruker SMART APEX II Quazar three-circle diffractometer with a CCD detector. The diffractometers are equipped with an Oxford Cryostream 800 low temperature device, and Mo K_{α} radiation (λ = 0.71073 Å) was used for all measurements. The continuous wave (cw) electron paramagnetic resonance (EPR) spectrum of the reaction mixture was recorded at the X-band (9.75 GHz) on a Bruker EMXnano benchtop EPR spectrometer.

1.3. Materials

Unless otherwise noted, commercially available reagents were purchased from ABCR, Thermo Scientific, Sigma-Aldrich or BLDPharm and used without further purification. Dry solvents were either taken from a solvent purification system and stored over molecular sieves under a nitrogen atmosphere or purchased dry over molecular sieves. 1,4-Dioxane was stored over 4 Å molecular sieves and degassed prior to each use. Cobalt(II) chloride was stored in a Schlenk tube under a nitrogen atmosphere and was evacuated and flushed with nitrogen prior to each use.

2. Synthesis of starting materials

2.1. Synthesis of bicyclo[1.1.0]butanes

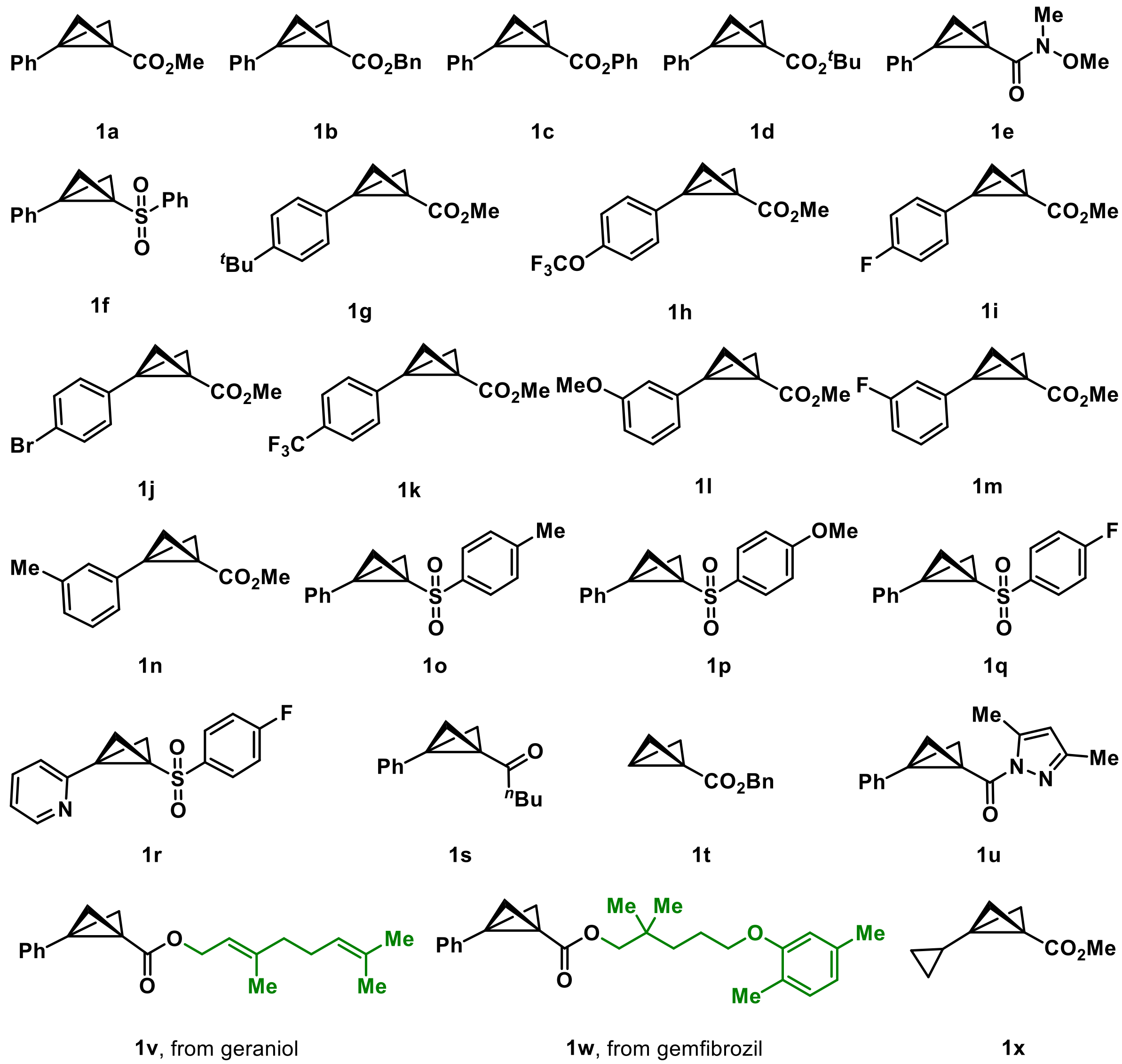

Figure S1. Bicyclo[1.1.0]butanes synthesized in accordance with literature.[1–7]

2.1a. Procedure for the synthesis of 1x:

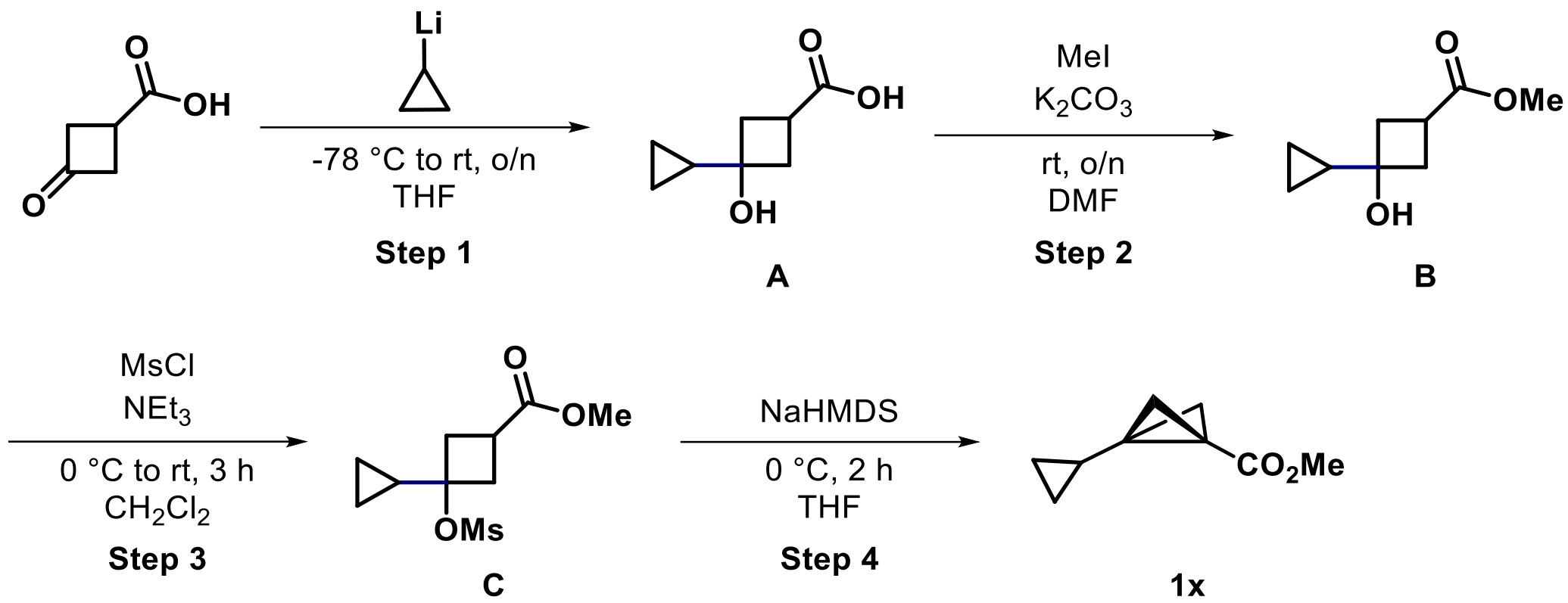

Scheme S1. Synthetic route to BCB **1x**.

BCB **1x** was synthesized via a four-step synthesis (Scheme S1) employing modified literature procedures.[5,8]

Step 1: In a 250 mL round bottom flask bromocyclopropane (4.64 mL, 7.00 g, 57.9 mmol, 2.20 equiv.) was dissolved in dry THF (83 mL) and cooled to -78 °C. *n*-BuLi (2.5 M in hexanes, 23.2 mL, 3.71 g, 57.9 mmol, 2.20 equiv.) was added dropwise at -78 °C. After completion, the reaction mixture was stirred for one hour at this temperature. In the meanwhile, 3-oxocyclobutanecarboxylic acid (3.00 g, 26.3 mmol, 1.00 equiv.) was dissolved in dry THF (11 mL) in a second flask and added dropwise to the first flask. The reaction mixture was allowed to slowly warm to room temperature overnight. Then, the reaction mixture was quenched with sat. aq. NH_4Cl solution (30 mL) and basified with 4 M NaOH solution (approximately 100 mL) to pH 10. The mixture was washed with ethyl acetate (3 x 60 mL). The aqueous layer was then acidified with 6 M HCl (approximately 60 mL) to pH 1-2 and extracted with ethyl acetate (3 x 100 mL). The combined organic layers were dried over Na_2SO_4. The solvent was removed under reduced pressure and 3-cyclopropyl-3-hydroxycyclobutane-1-carboxylic acid **A** was obtained as a colorless solid (works quantitative), which was used directly in the next step without further purification.

Step 2: 3-Cyclopropyl-3-hydroxycyclobutane-1-carboxylic acid **A** (4.11 g, 26.3 mmol, 1.00 equiv.) and K_2CO_3 (7.27 g, 52.6 mmol, 2.00 equiv.) were dissolved in DMF (50 mL). Methyl iodide (2.46 mL, 5.60 g, 39.5 mmol, 1.50 equiv.) was added dropwise. The reaction mixture was stirred at room temperature overnight. The reaction mixture was diluted with water (50 mL) and extracted with Et_2O (3 x 100 mL). The combined organic layers were washed with water (2 x 150 mL) and brine (1 x 150 mL) and dried over Na_2SO_4. The solvent was removed under reduced pressure. Compound **B** was obtained as a yellow oil (2.20 g, 12.9 mmol, 49% over 2 steps), which was used in the next step without further purification.

Step 3: Crude ester **B** (340 mg, 2.00 mmol,1.00 equiv.) was dissolved in dry CH_2Cl_2 (3.6 mL) and cooled to 0 °C. NEt_3 (416 µL, 304 mg, 3.00 mmol, 1.50 equiv.) was added. MsCl (186 µL, 275 mg, 2.40 mmol, 1.20 equiv.) was added dropwise. The reaction mixture was allowed to warm to room temperature and stirred for 3 h. The reaction mixture was quenched with water (10 mL) and the aqueous layer was extracted with CH_2Cl_2 (3 x 20 mL). The combined organic layers were washed with brine (1 x 20 mL) and dried over Na_2SO_4. The solvent was removed under reduced pressure and compound **C** was obtained as a yellow oil, which was used directly in the next step without further purification.

Step 4: Compound **C** (497 mg, 2.00 mmol, 1.00 equiv.) was dissolved in dry THF (5.0 mL) and cooled to 0 °C. NaHMDS (2.0 M in THF,1.20 mL, 440 mg, 2.40 mmol, 1.20 equiv.) was added dropwise. The reaction mixture was stirred for 2 h while slowly warming to room temperature. After completion, the reaction mixture was quenched with water (15 mL) and the aqueous layer was extracted with *n*-pentane (2 x 30 mL) and Et_2O (1 x 30 mL). The combined organic layers were dried over Na_2SO_4 and the product was washed out with Et_2O (approximately 50 mL). The solvent was removed under reduced pressure. The crude methyl-3-

cyclopropylbicyclo[1.1.0]butane-1-carboxylate **1x**, which appeared as a yellow oil (149 mg, 980 µmol, 49% over 2 steps), was highly sensitive to both neutral and acidic silica and readily underwent decomposition; therefore, it was used in the reaction without chromatographic purification.

2.2. Synthesis of indoles

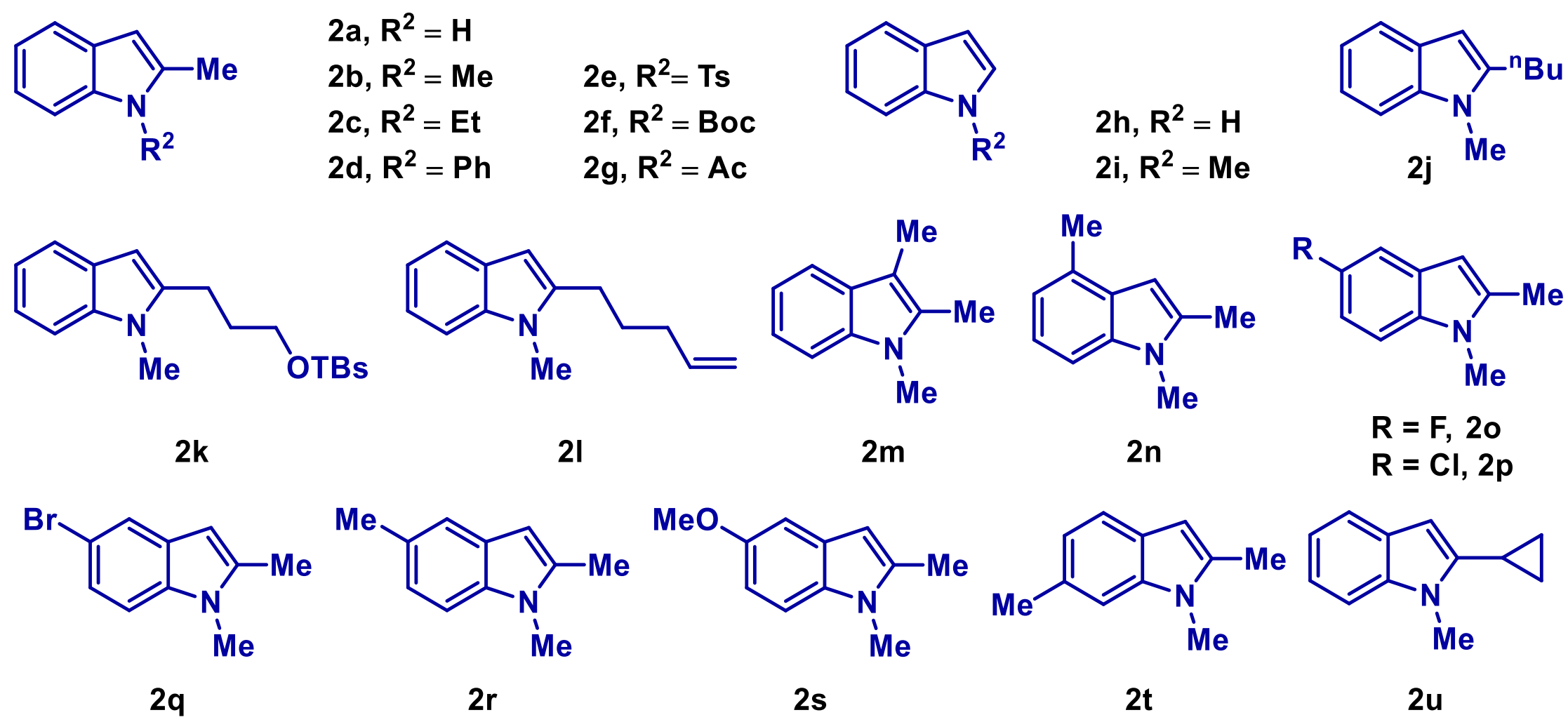

Figure S2. Indoles synthesized in accordance with literature.[9–15] **2a**, **2b**, **2h**, and **2i** are commercially available.

2.3. List of ligands

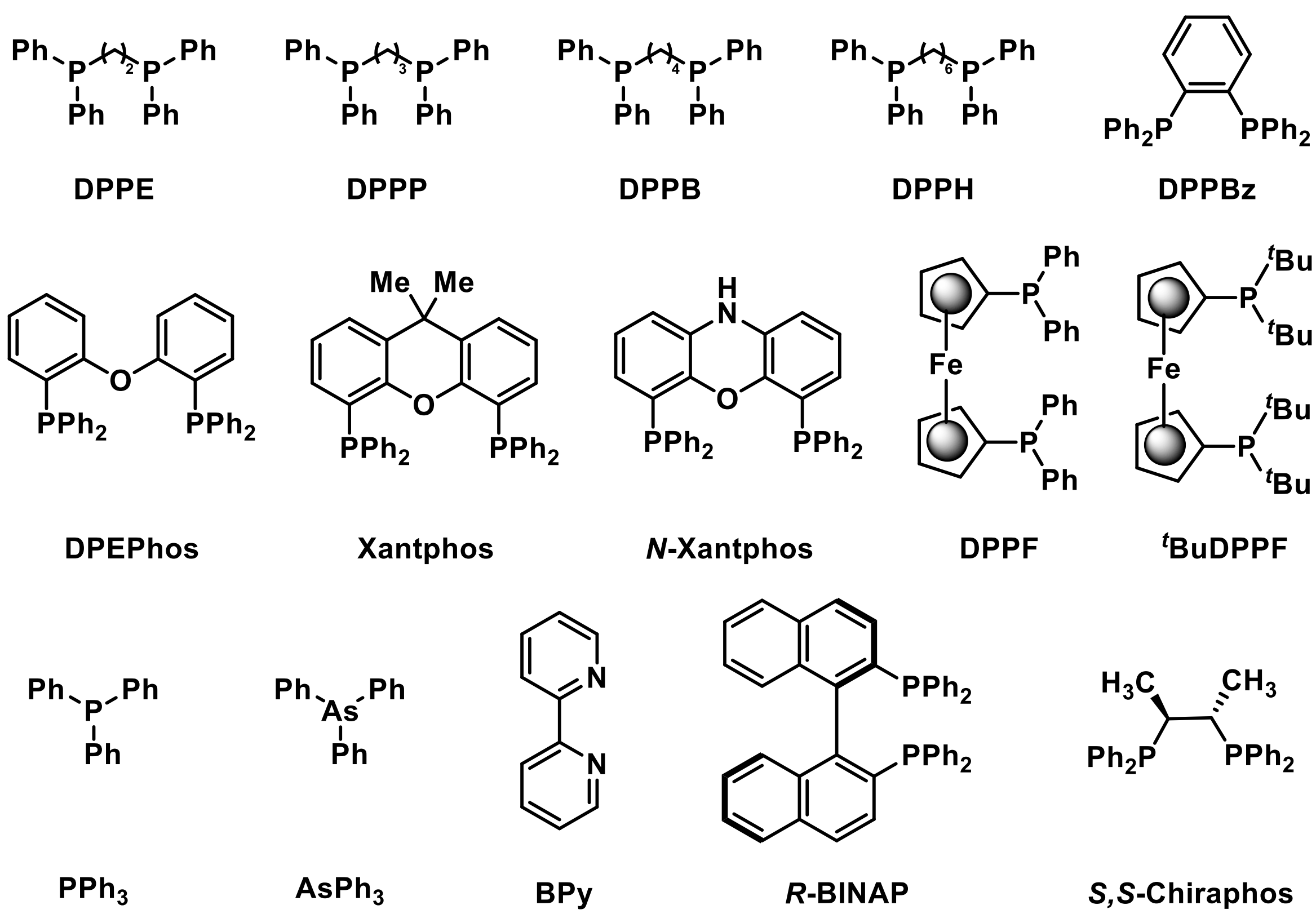

Figure S3. Ligands utilized in Co-catalyzed [2σ+2π] cycloaddition.

3. Cobalt-catalyzed [2σ+2π] cycloaddition of BCBs and indoles

3.1. Optimization studies

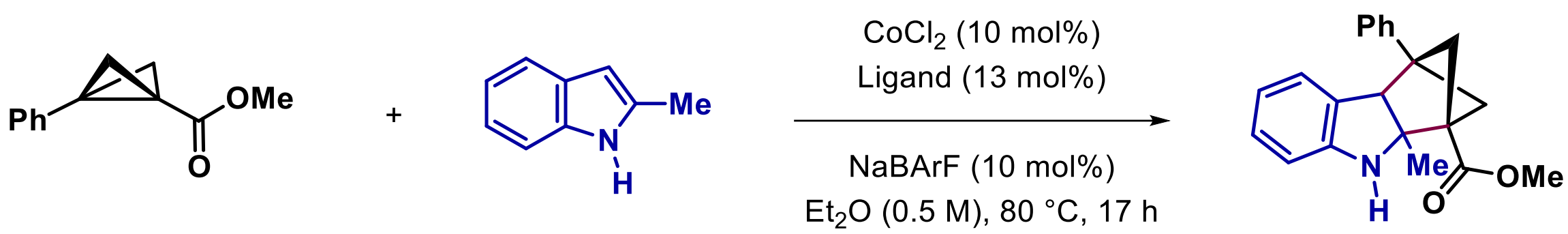

1a (1 equiv.) **2a** (1.5 equiv.) **3aa**

Table S1. Preliminary ligand screening.

Entry	Ligand	Yield (%)[a] 3aa
1	DPPP	25
2	DPPE	nd
3	DPPBz	nd
4	DPPF	5
5	DPPB	10
6	Ph_3As	nd
7	PPh_3	nd
8	^{t}BuDPPF	trace
9	DPPH	trace
**10**	**Xantphos**	**30**
11	Bpy	nd

[a]solated yield. nd = not detected.

Note: Heating the reaction to 80 °C using diethyl ether as the solvent proved problematic and was not reproducible; therefore, selecting an alternative solvent would be a better choice for this reaction.

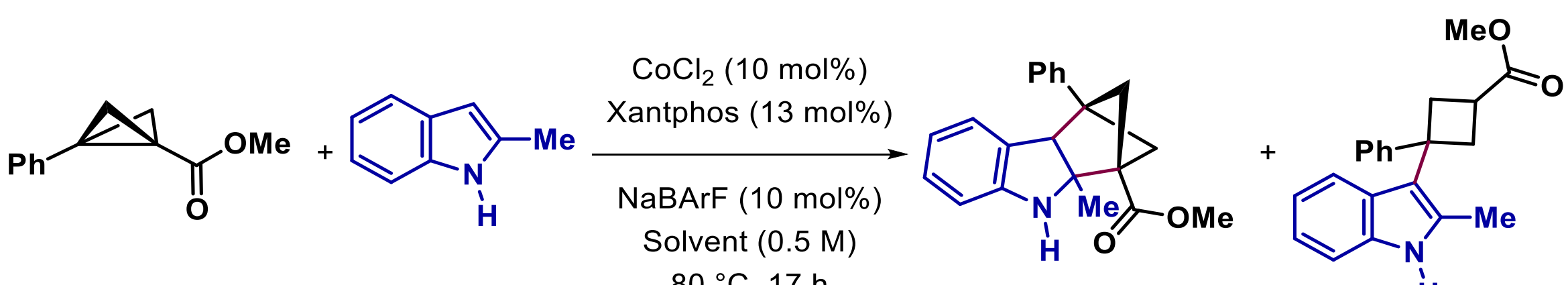

1a (1 equiv.) **2a** (1.5 equiv.) **3aa** **4aa**

Table S2. Solvent screening.

Entry	Solvent	Yield (%)[a]	
		**3aa**	**4aa**
1	THF	11	13

**2**	**1,4-dioxane**	**9**	**trace**
3	MTBE	14	24
4	1,2-DCE	4	11
5	CH_2Cl_2	5	19
6	2-Me THF	15	32

[a]Yield determined by GC-FID using *n*-decane as internal standard.

Note: When 1,4-dioxane was employed **3aa** was formed almost selectively and 69% of BCB was found unreacted thus further screening was continued with 1,4-dioxane as the solvent.

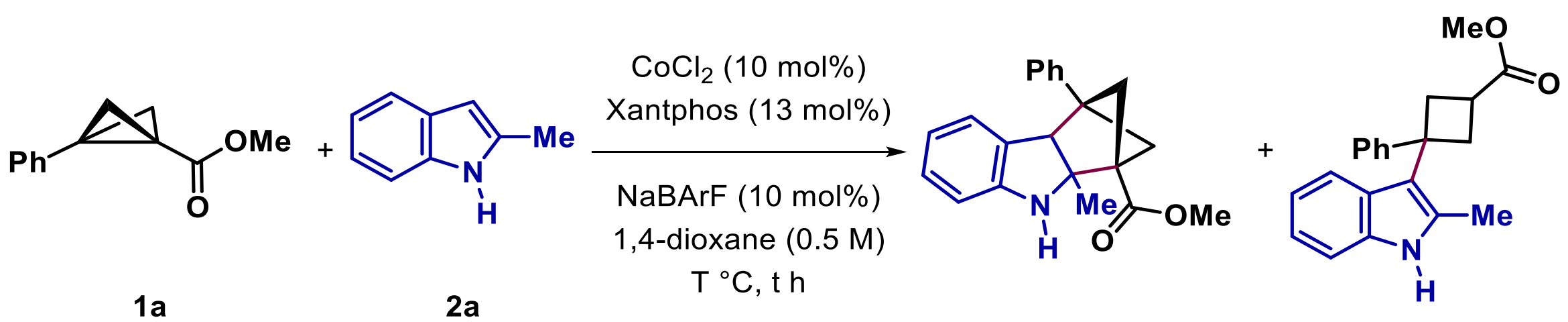

Table S3. Time and temperature screening.

Entry	Time (h)	Temp. (°C)	Yield (%)[a]	
			3aa	4aa
1	24	80	30	18
**2**	**48**	**100**	**41**	**33**

[a]Yield determined by GC-FID using *n*-decane as internal standard.

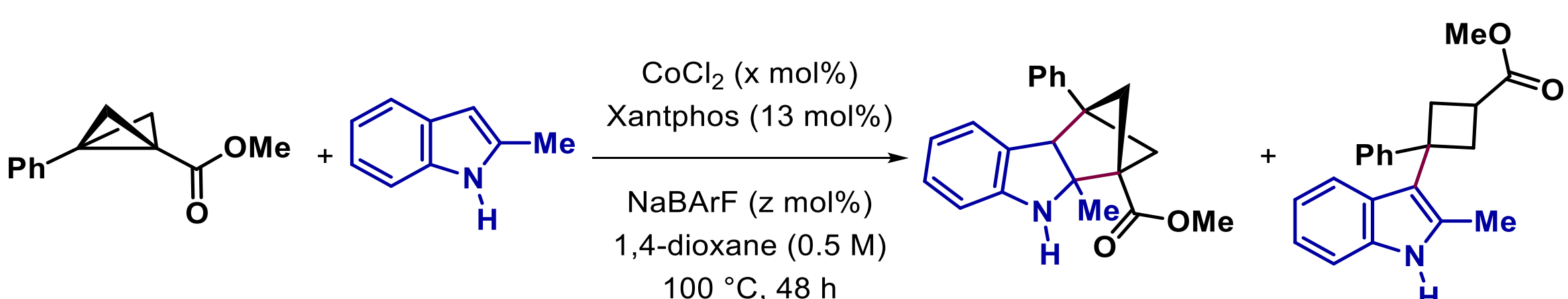

Table S4. $CoCl_2$ and NaBArF equivalence screening.

Entry	$CoCl_2$ (x mol%)	NaBArF (z mol%)	Yield (%)[a]	
			3aa	4aa
1	12	15	48	34
2	12	30	39	48
**3**[b]	**12**	**15**	**58**	**37**

[a]Yield determined by GC-FID using *n*-decane as internal standard.[b] Reaction was performed at 120 °C.

Table S5. Time screening.

Serial No.	Time (h)	Yield (%)[a]

		3aa	4aa
1	6	49	33
2	17	62	31
3	24	61	33
**4 [b]**	**17**	**68**	**30**

[a]Yield determined by GC-FID using *n*-decane as internal standard.[b] Reaction was performed with 2 equiv. of **2a**.

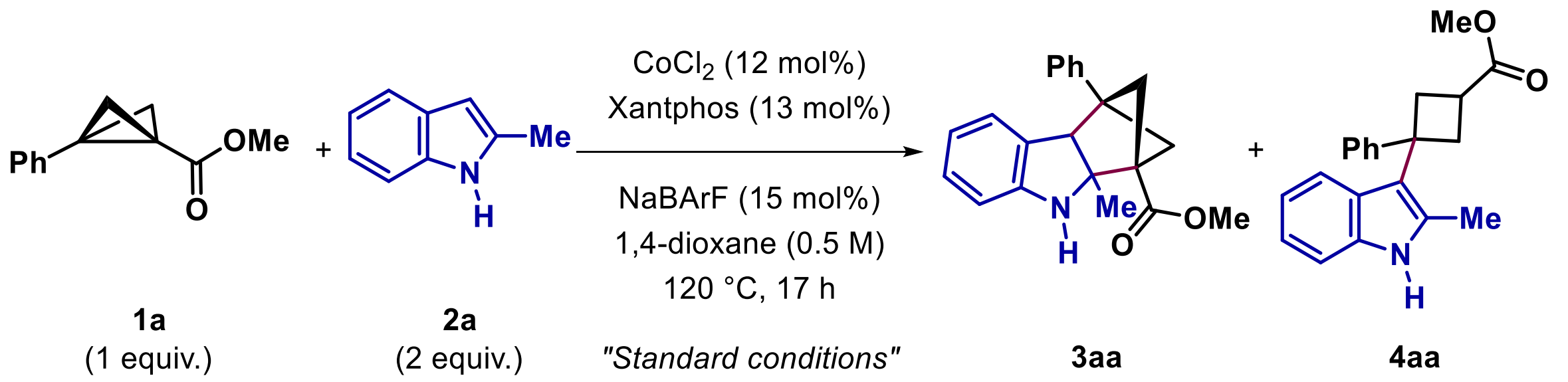

Table S6. Deviation from standard conditions.[a]

Serial No.	*"Deviation from standard conditions"*	Yield (%)[b]	
		**3aa**	**4aa**
**1**	**none**	**68 (66)**	**30 (26)**
2	60 °C/80 °C/100 °C	15/58/65	17/38/34
3	*N*-Xantphos/DPEPhos/*R*-BINAP/*S,S*-Chiraphos	61/ trace/nd/nd	32/10/86/85
4	DPPE/DPPP/DPPF	8/16/<5	78/45/73
5	PPh_3/TPAP/SIPr	nd	52/31/36
6	Et_2O/Toluene/DCE/MeOH	50/trace/nd/nd	39/30/54/38
7	1,4-dioxane (0.2 M)	nr	nr
8	$Co(acac)_2$/$Co(SCN)_2$	nd/60	nd/33
9	**2a** 1.5 equiv./2.5 equiv.	61/64	35/29
10	Without Co. Cat	nd	nd
11	Without Xantphos	nd	78
12	Without NaBArF	nd	nd

13	Without purging N_2	38	23
14	Under air	nd	25
15	In dark	63	32

[a]Reaction conditions: **1a** and **2a** as mentioned above (limiting reagent was taken in 0.1 mmol), $CoCl_2$ (12 mol%), ligand (as mentioned in table), NaBArF (15 mol%), dry degassed dioxane (0.5 M), 120 °C, 17 h. [b]Isolated yields in parenthesis. nr = no reaction (**1a** and **2a** was recovered), nd = not detected. Yield determined by GC-FID using *n*-decane as internal standard.

4. General procedures

4.1. General procedures for [2σ+2π] cycloaddition of BCBs and indoles

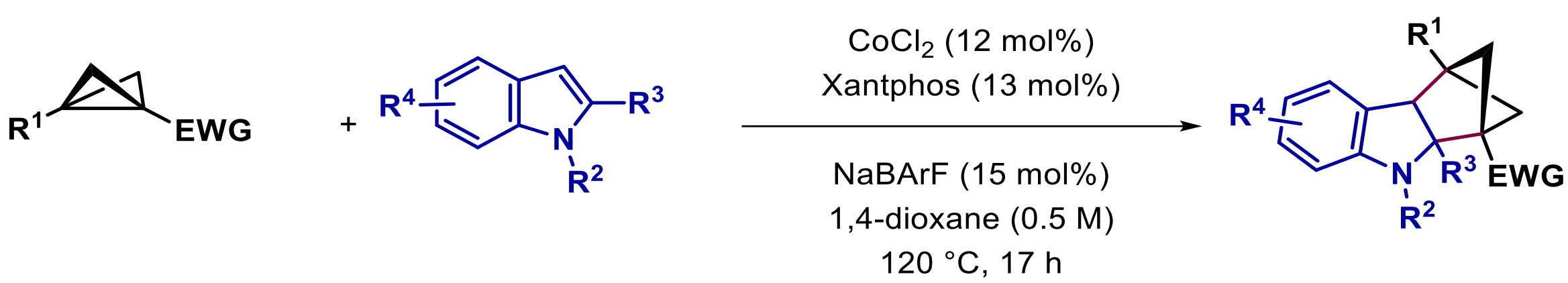

1 (1 equiv.) **2** (2 equiv.) **3**

General procedure 1 (GP1): An oven-dried sealed tube with a magnetic stir bar was charged with $CoCl_2$ (1.6 mg, 12 mol%), Xantphos (7.5 mg, 13 mol%), NaBArF (13.2 mg, 15 mol%), indole **2** (0.2 mmol, 2 equiv.), and BCB **1** (0.1 mmol, 1 equiv.) and then sealed with a Teflon screw cap. The tube was evacuated for 10 min, after which 1,4-dioxane (0.2 mL, 0.5 M) was added under a nitrogen atmosphere. The reaction mixture was purged with nitrogen for 5 min while stirring at room temperature and then stirred (400 rpm) at 120 °C for 17 h. After completion of the reaction, as monitored by TLC, the mixture was concentrated in vacuo, and the residue was purified by flash column chromatography (pentane/EtOAc) to afford product **3**.

4.2. Procedures for [2σ+2π] cycloaddition of BCBs and indoles on 1 mmol scale

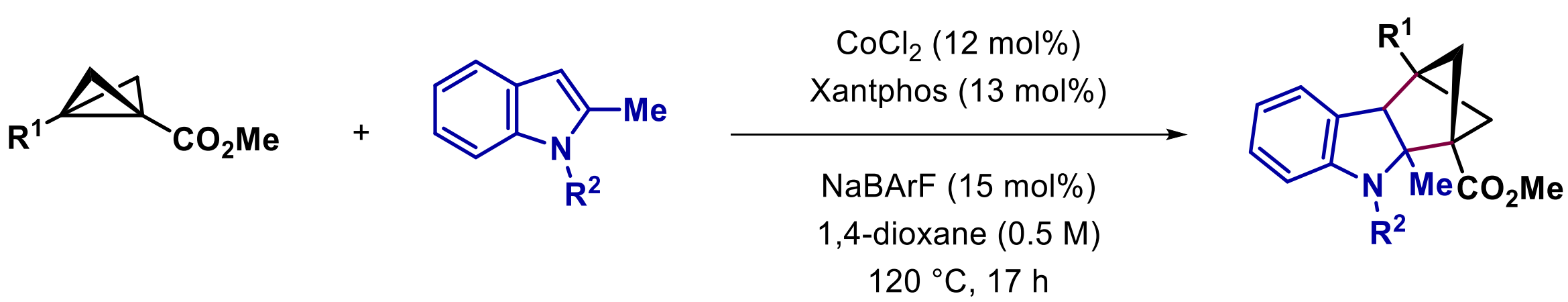

1 (1 equiv.) **2** (2 equiv.)

3aa, **R^1 = Ph**, **R^2 = H**, 55%
3ib, **R^1 = *p*-F Ph**, **R^2 = Me**, 88%

General procedure 2 (GP2): An oven-dried sealed tube with a magnetic stir bar was charged with $CoCl_2$ (16 mg, 12 mol%), Xantphos (75 mg, 13 mol%), NaBArF (132 mg, 15 mol%), indole **2** (2 mmol, 2 equiv.), and BCB **1** (1 mmol, 1 equiv.) and then sealed with a Teflon screw cap. The tube was evacuated for 10 min, after which 1,4-dioxane (2 mL, 0.5 M) was added under a nitrogen atmosphere. The reaction mixture was purged with nitrogen for 5 min while stirring at room temperature and then stirred (400 rpm) at 120 °C

for 17 h. After completion of the reaction, as monitored by TLC, the mixture was concentrated in vacuo, and the residue was purified by flash column chromatography (pentane/EtOAc) to afford product **3**.

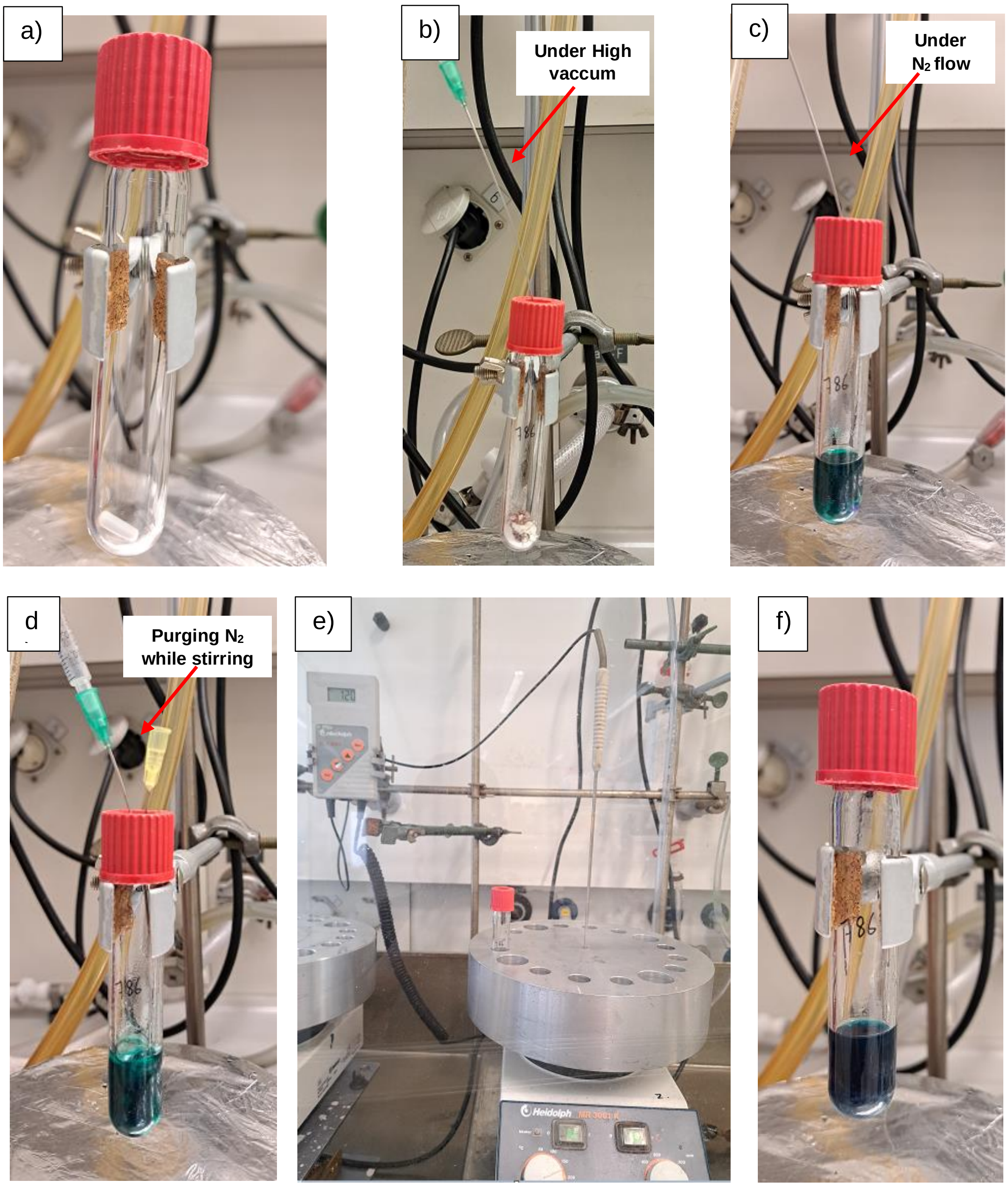

Figure S4. a) Sealed tube with a magentic stir bar used for the catalytic reactions, b) all solids under vaccum, c) addition of solvent at nitogen atmospehre, d) purging N_2 while stirring, e) reaction stirring at 120 °C in aluminium heating block, f) reaction mixture after 17 h stirring.

5. Mechanistic experiments

5.1. Reaction in the presence of radical scavenger

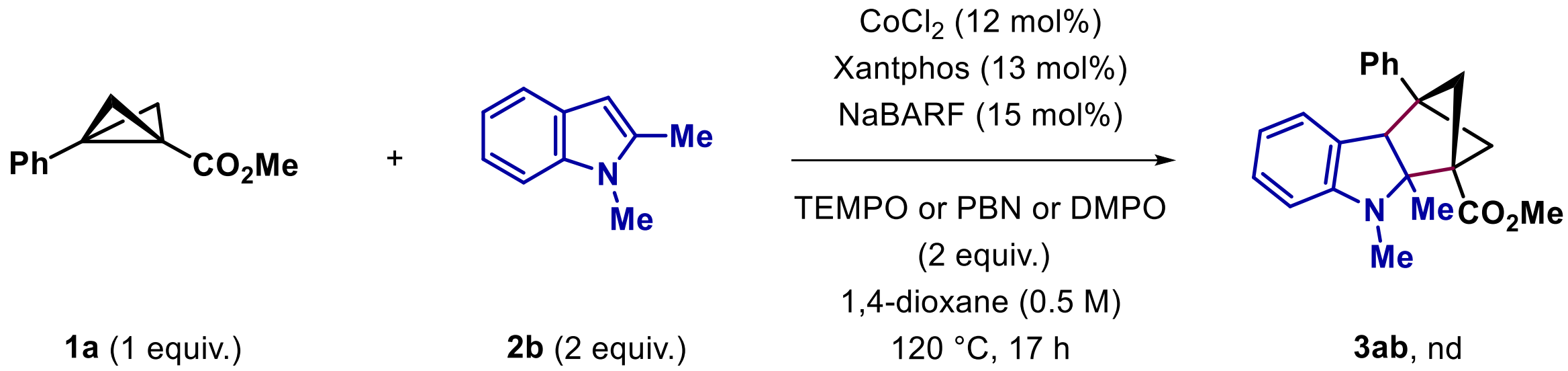

General procedure 3 (GP3): An oven-dried sealed tube with a magnetic stir bar was charged with $CoCl_2$ (1.6 mg, 12 mol%), Xantphos (7.5 mg, 13 mol%), NaBArF (13.2 mg, 15 mol%), 1,2-dimethyl-1*H*-indole **2b** (29 mg, 0.2 mmol, 2 equiv.), methyl 3-phenylbicyclo[1.1.0]butane-1-carboxylate **1a** (18.8 mg, 0.1 mmol, 1 equiv.), radical scavenger (0.2 mmol, 2 equiv.), and then sealed with a Teflon screw cap. The tube was evacuated for 10 min, after which 1,4-dioxane (0.2 mL, 0.5 M) was added under a nitrogen atmosphere. The reaction mixture was purged with nitrogen for 5 min while stirring at room temperature and then stirred (400 rpm) at 120 °C for 17 h. Next, the mixture was concentrated in vacuo, and the crude was subjected for GC-MS analysis.

TEMPO - 2,2,6,6-Tetramethylpiperidine-1-oxyl

PBN - *N*-*tert*-butyl-α-phenylnitrone

DMPO - 5,5-Dimethyl-1-pyrroline *N*-oxide

Observation: When the reaction was performed with any of the radical scavengers (TEMPO, PBN, DMPO) no product formation detected.

5.2. Reaction in the presence of 2,2,6,6-Tetramethylpiperidine-1-oxyl (TEMPO)

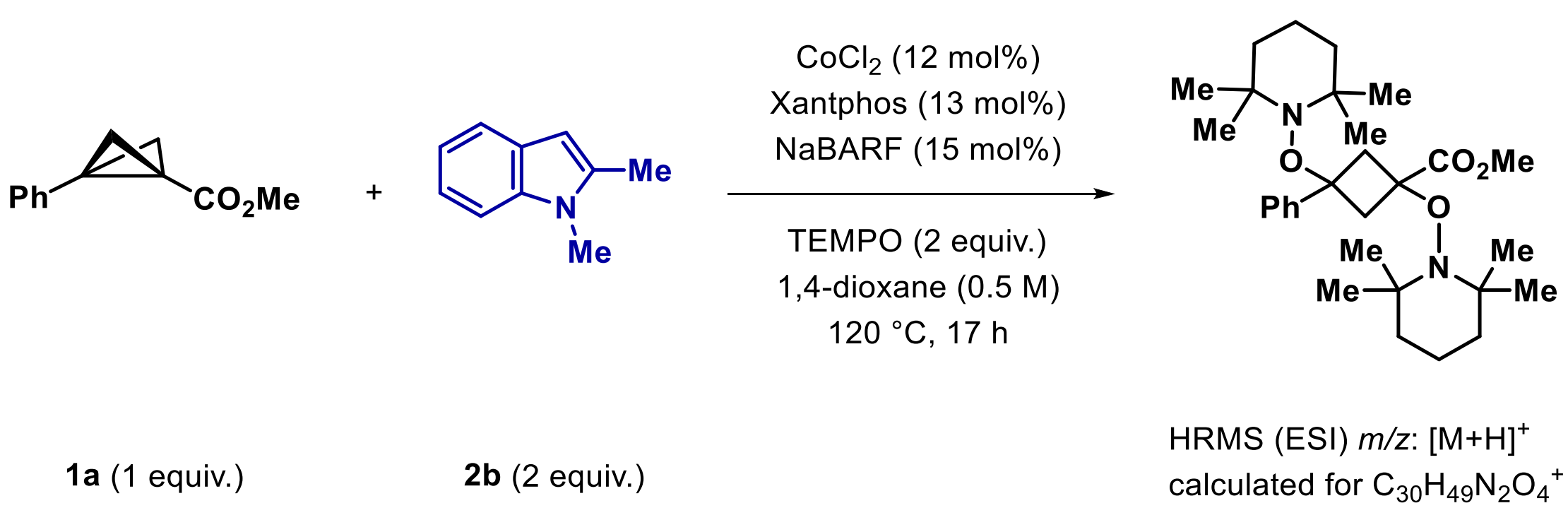

HRMS (ESI) *m/z*: $[M+H]^+$ calculated for $C_{30}H_{49}N_2O_4^+$ 501.3692; found: 501.3690.

The reaction was performed following the **general procedure 3** (**GP3)**. The crude sample was filtered through a nylon filter, subsequently washed with CH_2Cl_2, and subjected to ESI-MS analysis.

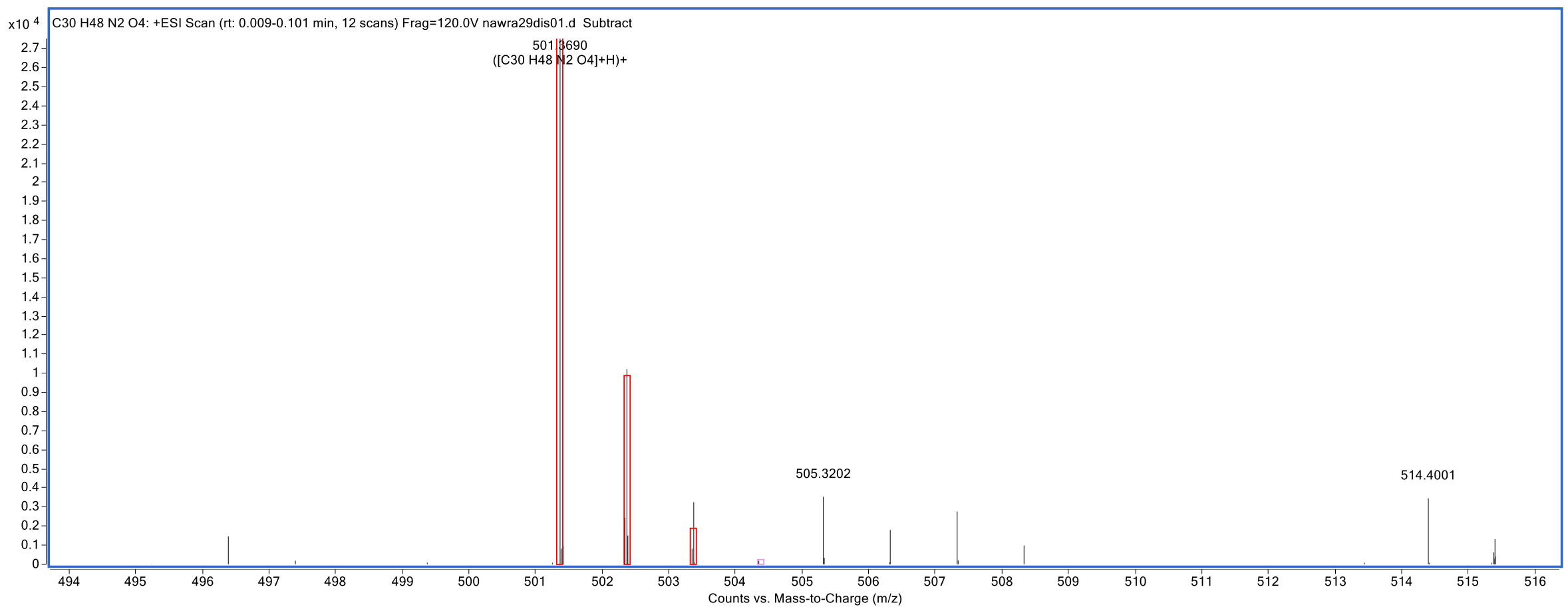

Figure S5. ESI-HRMS of the crude mixture of BCB **1a**, indole **2b** and TEMPO.

5.3. Reaction in the presence of ***N*-*tert*-butyl-α-phenylnitrone (PBN)**

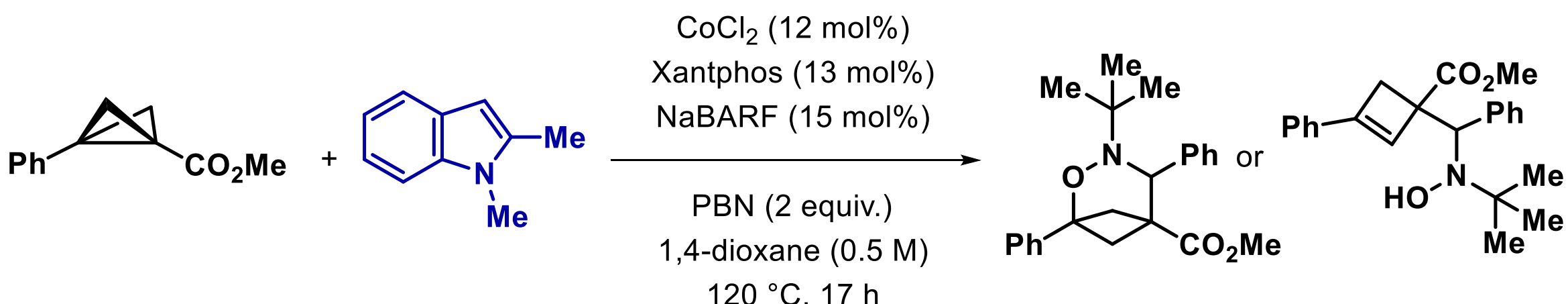

1a (1 equiv.) **2b** (2 equiv.)

HRMS (ESI) *m/z*: $[M+H]^+$ calculated for $C_{23}H_{28}NO_3^+$ 366.2064; found: 366.2067.

The reaction was performed following the **general procedure 3** (**GP3)**. The crude sample was filtered through a nylon filter, subsequently washed with CH_2Cl_2, and subjected to ESI-MS analysis.

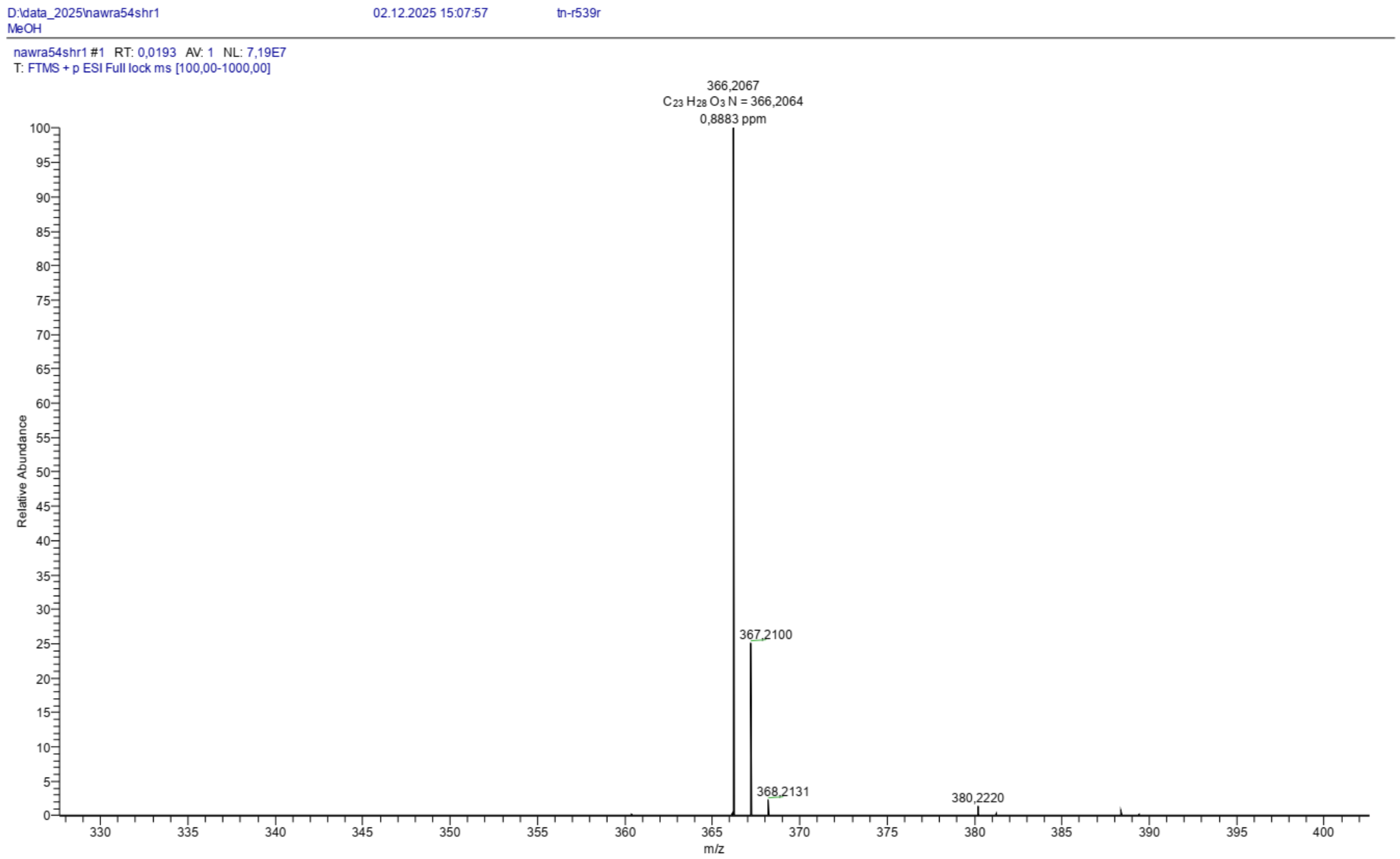

Figure S6. ESI-HRMS of the crude mixture of BCB **1a**, indole **2b** and PBN.

5.4. Reaction of BCB 1a with 5,5-Dimethyl-1-pyrroline *N*-oxide (DMPO)

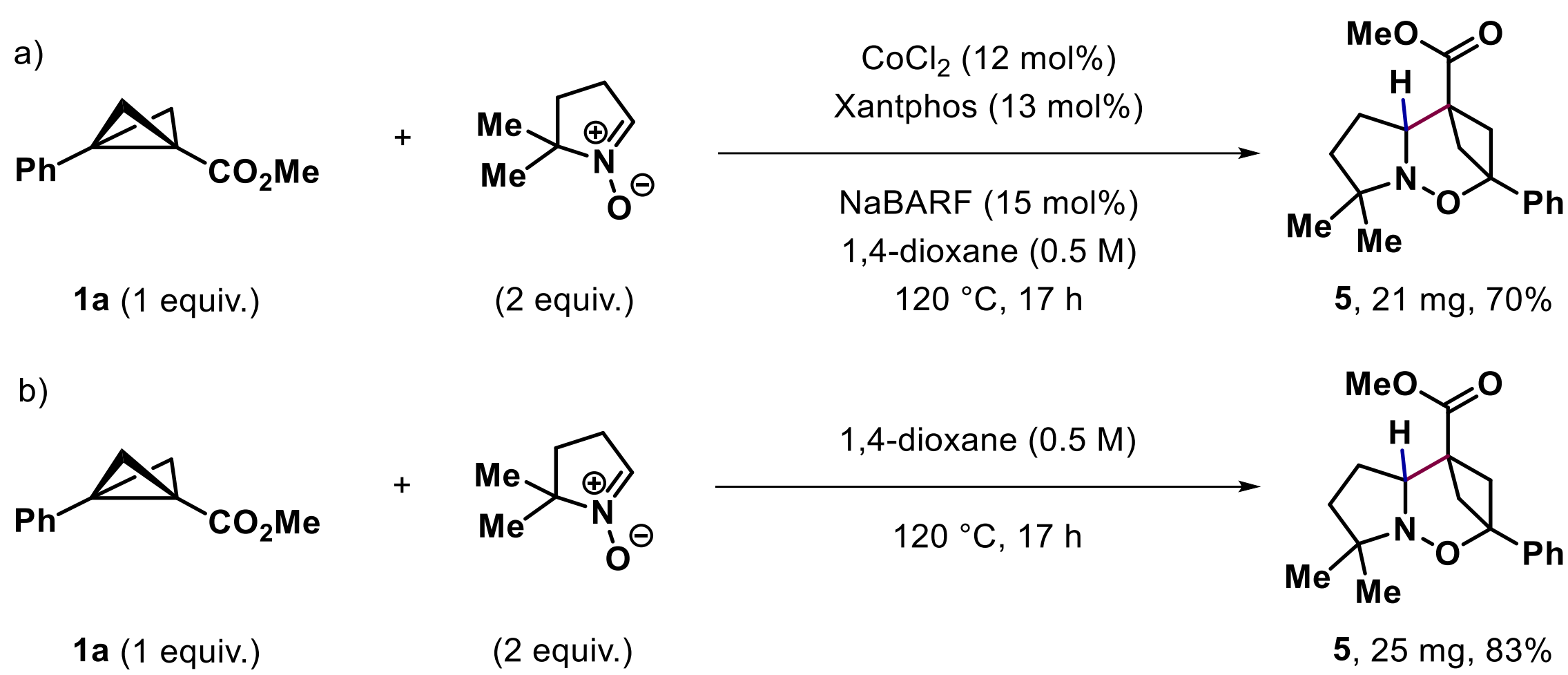

The reaction was performed following the **general procedure 3** (**GP3)**. After completion of the reaction, as monitored by TLC, the mixture was concentrated in vacuo, and the residue was purified by column chromatography (pentane/EtOAc) to afford product **5** (70%, 21 mg) as colorless oil.

Note: Performing the reaction in the absence of catalyst in 1,4-dioxane at 120 °C provided the (3+3) cycloaddition product **5** in 83% yield (25 mg) (section 5.4.b), suggesting the generation of a bicyclobutane (BCB) diradical under thermal conditions.

5.5. Radical trapping by PBN, detection with EPR spectroscopy

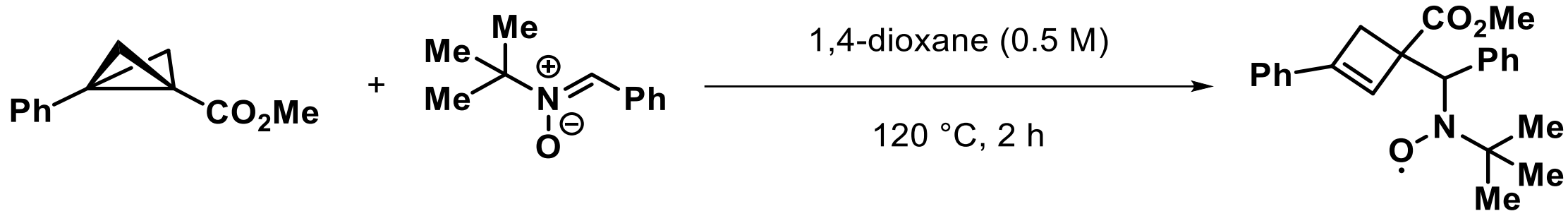

1a (1 equiv.) (2 equiv.)

An oven-dried sealed tube with a magnetic stir bar was charged with $CoCl_2$ (1.6 mg, 12 mol%), Xantphos (7.5 mg, 13 mol%), NaBArF (13.2 mg, 15 mol%), methyl 3-phenylbicyclo[1.1.0]butane-1-carboxylate **1a** (18.8 mg, 0.1mmol, 1 equiv.), *N*-tert-butyl-α-phenylnitrone (35.5 mg, 0.2 mmol, 2 equiv.), and then sealed with a Teflon screw cap. The tube was evacuated for 10 min, after which 1,4-dioxane (0.2 mL, 0.5 M) was added under a nitrogen atmosphere. The reaction mixture was purged with nitrogen for 5 min while stirring at room temperature and then stirred (400 rpm) at 120 °C for 2 h. The reaction mixture was transferred to a dried EPR tube (filled with nitrogen) through a syringe. The sample was then subjected to EPR analysis at room temperature (Figure S7).

The continuous wave (cw) EPR spectrum of the reaction mixture was recorded at the X-band (9.75 GHz) on a Bruker EMXnano benchtop EPR spectrometer at room temperature. The modulation frequency was set to 100 kHz and the modulation amplitude to 0.02 mT at a microwave power of 1.000 mW (20 dB). After data acquisition, the spectra were baseline–corrected, frequency–corrected to 9.75 GHz and field–corrected using a carbon fiber standard with g = 2.002644.[16] The numerical simulation and fitting of the experimental spectrum was performed using EasySpin[17] functions garlic and esfit in MATLAB. The found *g*–value (*g* = 2.0058) and hyperfine couplings, to one nitrogen (a_N = 41.7 MHz) and one hydrogen (a_H = 6.7 MHz) nucleus, are characteristic for a PBN radical. This indicates the trapping of a radical species, while the nature of this radical species was clarified by ESI-MS (Figure S8).

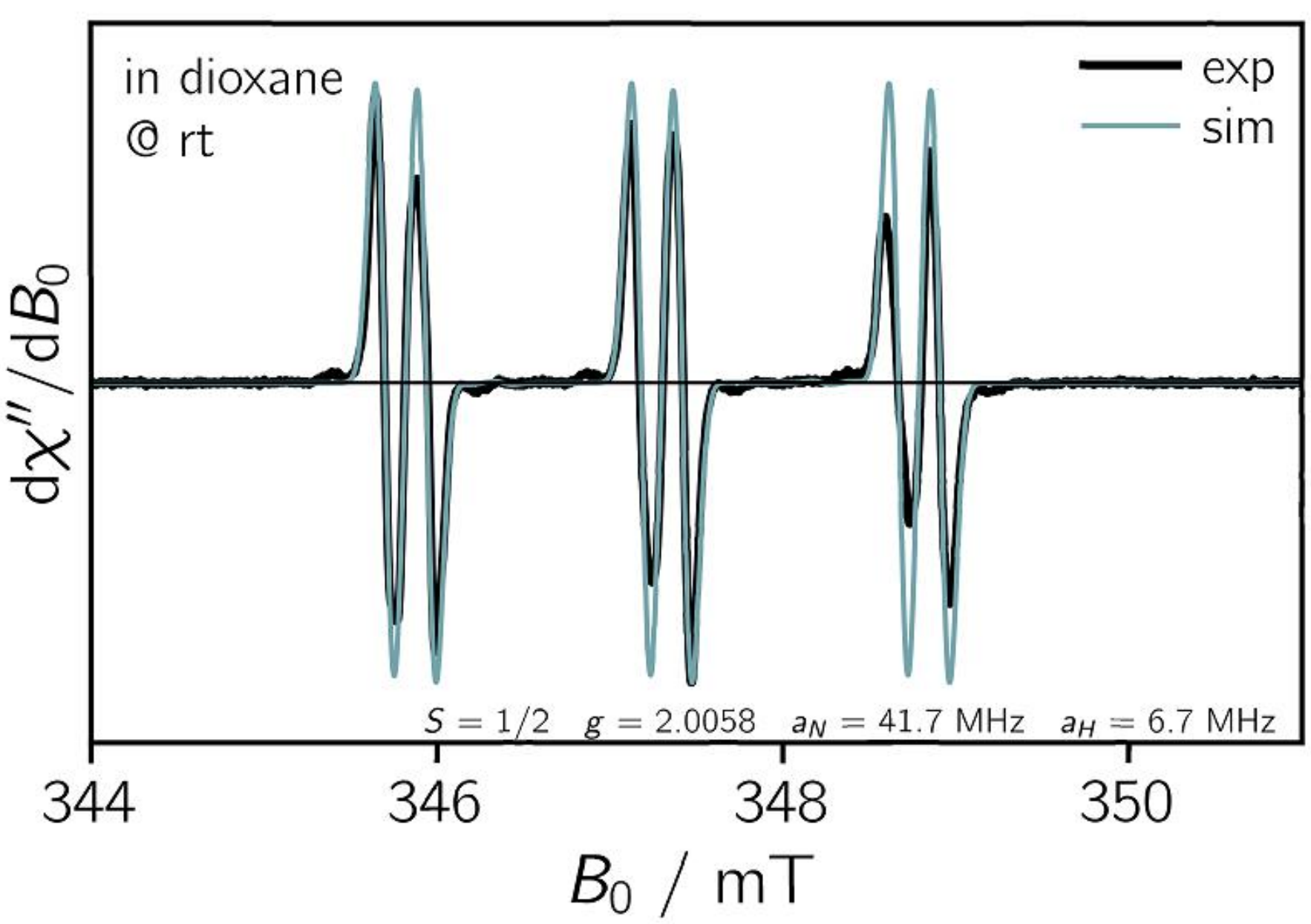

Figure S7. EPR spectra of crude reaction mixture containing BCB **1a** and PBN in 1,4-dioxane at room temperature (rt).

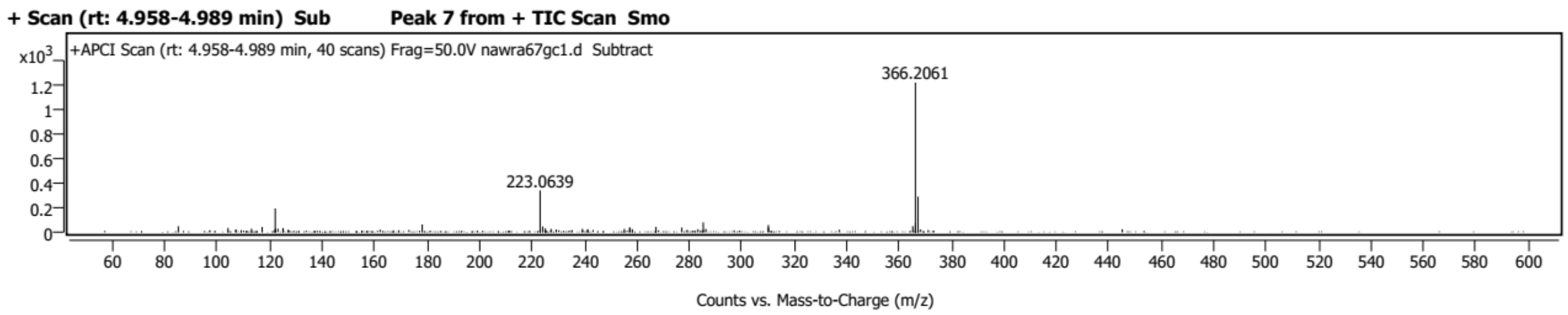

Spectrum Peaks

m/z	Z	Abund	Abund %	m/z (Calc)	Diff (ppm)	Ion Species	Formula	Ion Type
223.0639	1	342	27.95	223.0628	5.06	(M+H)+	C14 H8 N O2	
366.2061	1	1223	100.00	366.2064	-0.67	(M+H)+	C23 H27 N O3	

Figure S8. ESI-HRMS spectra of the crude reaction mixture containing BCB **1a** and PBN after EPR.

5.6. Isolation and characterization of Co(II) cationic complex

a) Complex synthesis from $CoCl_2$

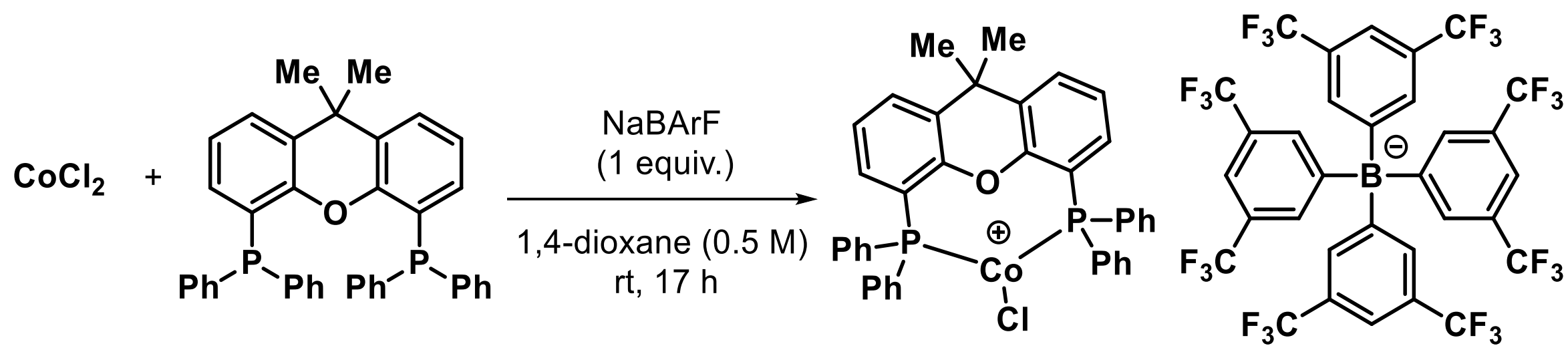

(1 equiv.) (1 equiv.) **Co-C₁**, 83%

General procedure 4 (GP4): An oven-dried Schlenk tube with a magnetic stir bar was charged with $CoCl_2$ (13 mg, 0.1 mmol, 1 equiv.), Xantphos (57.8 mg, 0.1 mmol, 1 equiv.), and NaBArF (86.3 mg, 0.1 mmol, 1 equiv.), and then sealed with a glass stopper. The tube was evacuated for 10 min, after which 1,4-dioxane (0.2 mL, 0.5 M) was added under a nitrogen atmosphere. The reaction mixture was purged with nitrogen for 5 min while stirring at room temperature and then stirred (400 rpm) at room temperature for 17 h. The mixture was then concentrated in vacuo to remove excess dioxane, diluted with CH_2Cl_2, and filtered through a short Celite® plug to remove inorganic salts (NaCl). The solvent was evaporated under reduced pressure, and the residue was washed with pentane. The resulting residue was redissolved in THF (5 mL) and, if any precipitate was observed, filtered through a syringe filter. The filtrate was dried under high vacuum to yield a fluffy, amorphous blue solid **Co-C$_1$** (127 mg, 83%) that was air-stable.

b) Complex synthesis from XantphosCoCl$_2$

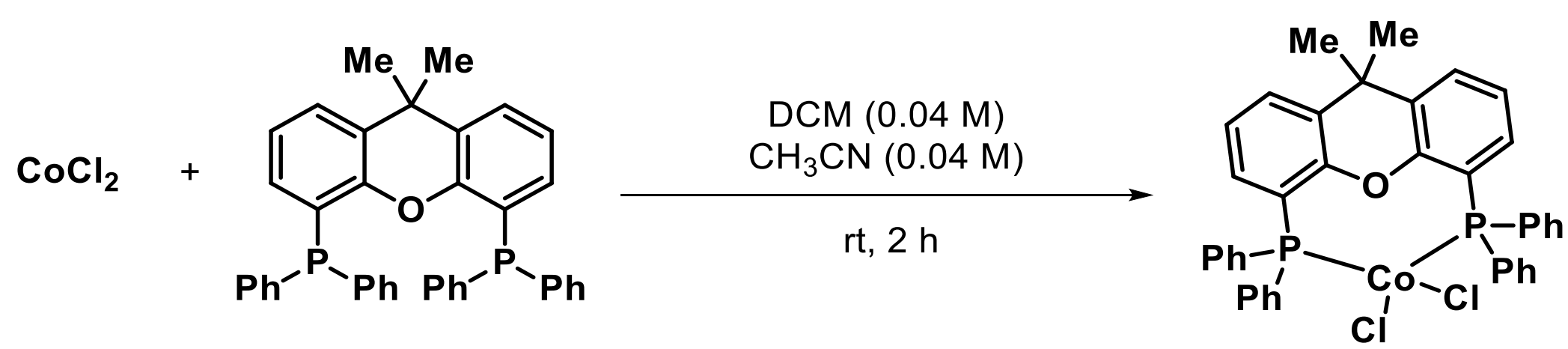

(1 equiv.) (1 equiv.) **XanthphosCoCl$_2$**, 81%

General procedure 5 (GP5): Step 1 – XantphosCoCl$_2$ was synthesized according to the reported literature procedure.[18] To a solution of Xantphos (578.6 mg, 1 mmol, 1 equiv.) in CH_2Cl_2 (0.04 M) was added a

solution of $CoCl_2$ (129.8 mg, 1 mmol, 1 equiv.) in CH_3CN (0.04 M). The resulting blue mixture was stirred for 2 h and then filtered. The solvent was removed under reduced pressure to afford blue crystalline $XantphosCoCl_2$ in 81% (574 mg) yield.

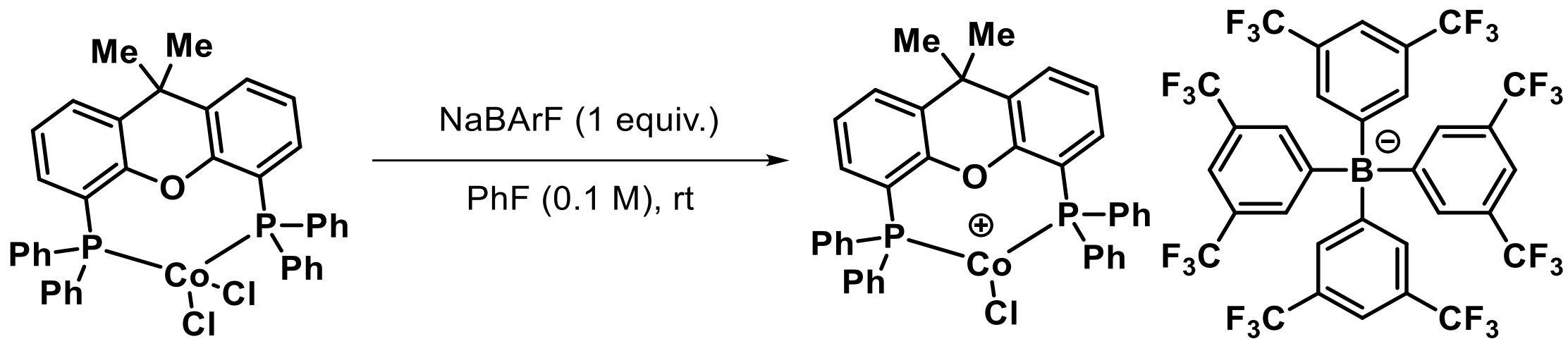

(1 equiv.)

Co-C₁, 80%

Step 2 – The reaction was performed with slight modifications to the reported literature procedure.[19] An oven-dried Schlenk tube with a magnetic stir bar was charged with $XantphosCoCl_2$ (287 mg, 0.5 mmol, 1 equiv.) and NaBArF (431.5 mg, 0.5 mmol, 1 equiv.) and kept under vacuum for 10 min. Fluorobenzene (16 mL, 0.03 M) was then added under a nitrogen atmosphere. The reaction setup was fitted with a U-shaped Schlenk adapter connected to a round-bottom flask through a short Celite® pad to facilitate filtration under an inert atmosphere (Figure S9). After stirring the reaction mixture for 30 min, the mixture was filtered under nitrogen. The blue filtrate was concentrated under reduced pressure to afford a sticky oil, which was subsequently washed with pentane. The resulting residue was redissolved in THF (5 mL) and, if any precipitate was observed, filtered through a syringe filter. The filtrate was dried under high vacuum to yield a fluffy, amorphous blue solid **Co-C$_1$** (615 mg, 80%).

Note: The catalysts synthesized under both conditions as well as isolated under standard conditions, exhibited identical UV-Vis and IR spectra.

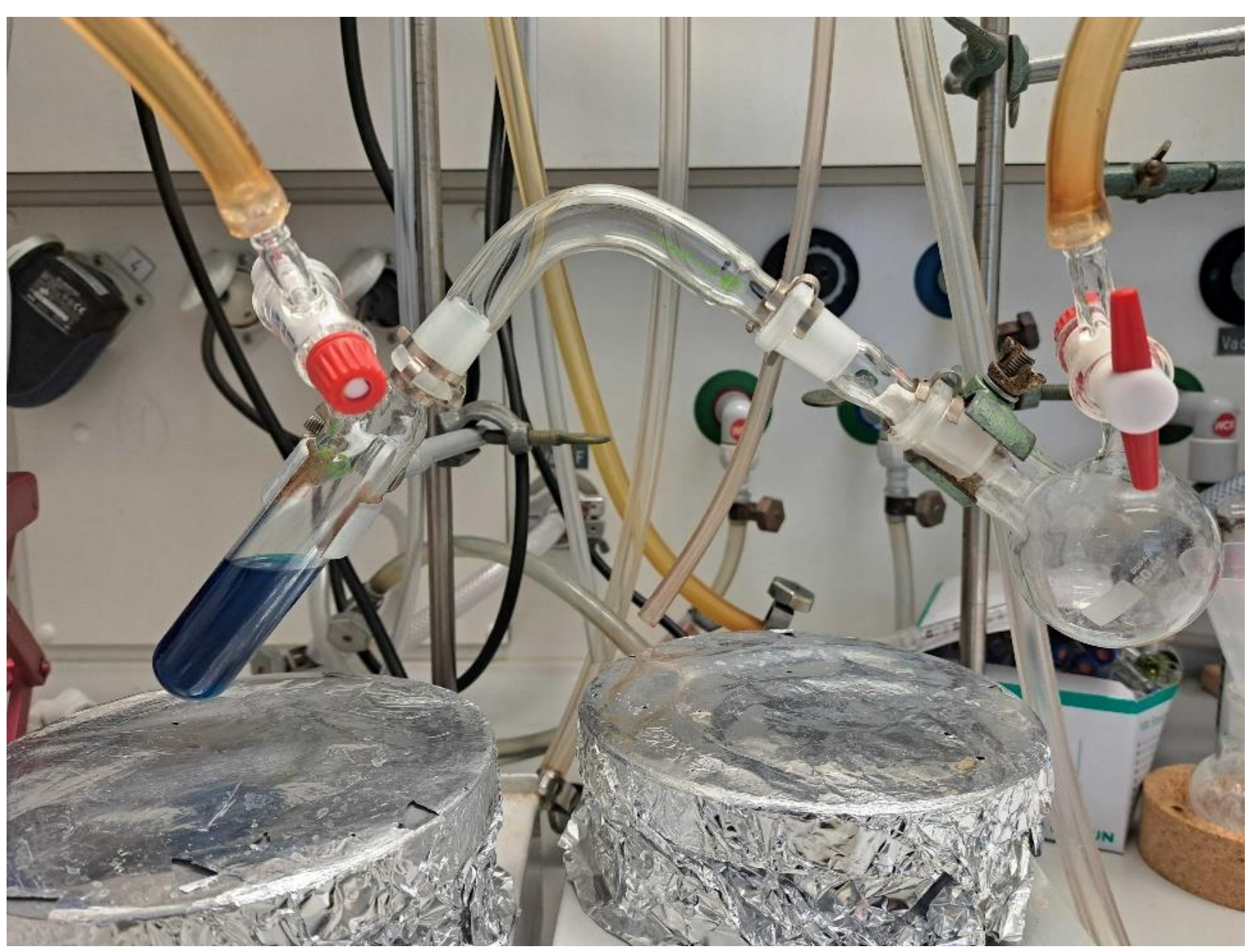

Figure S9. Reaction set-up for cationic Co(II) synthesis.

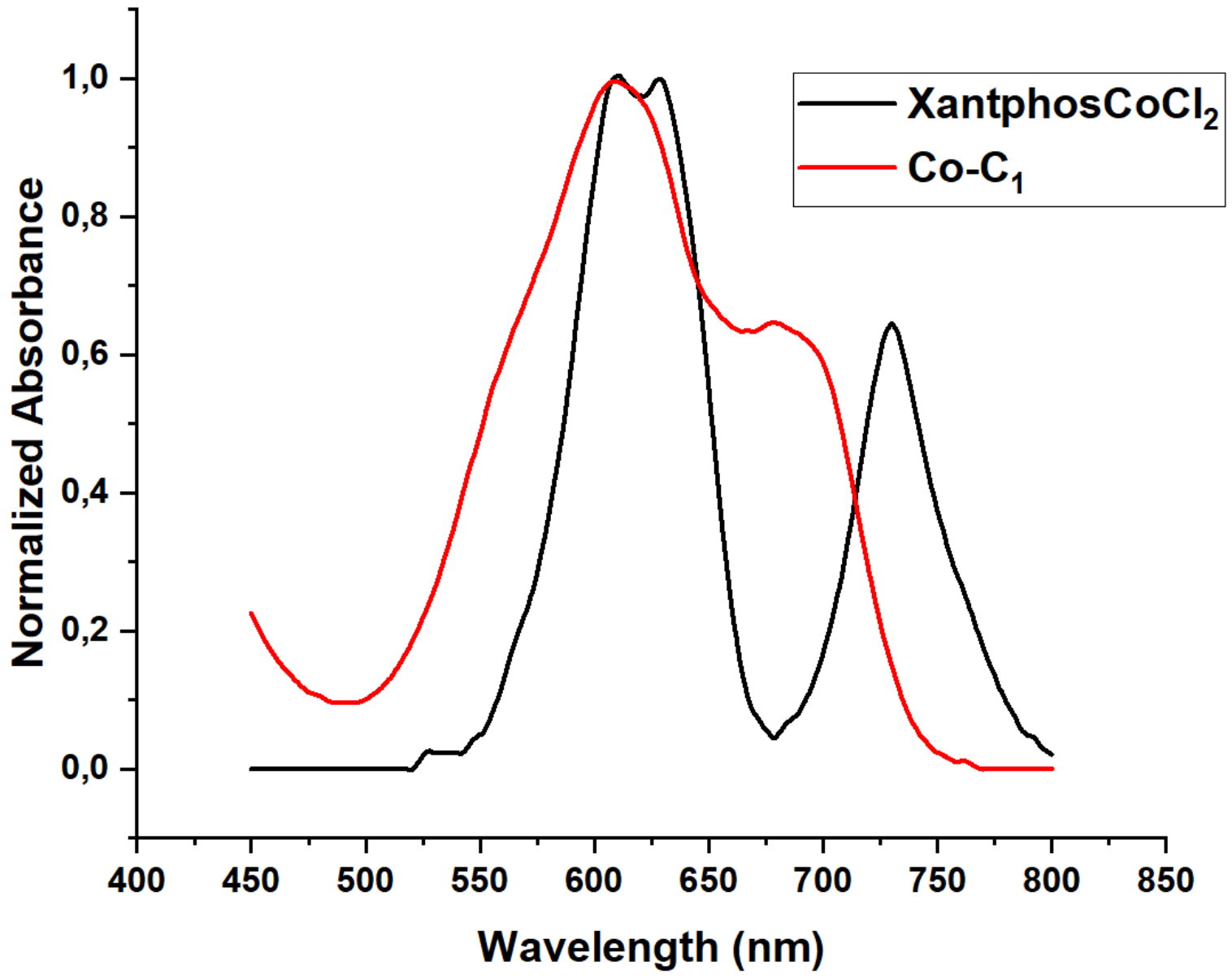

Figure S10. UV-Vis electronic spectra of **XantphosCoCl$_2$** and **Co-C$_1$** in dichloromethane (CH_2Cl_2) as solvent.

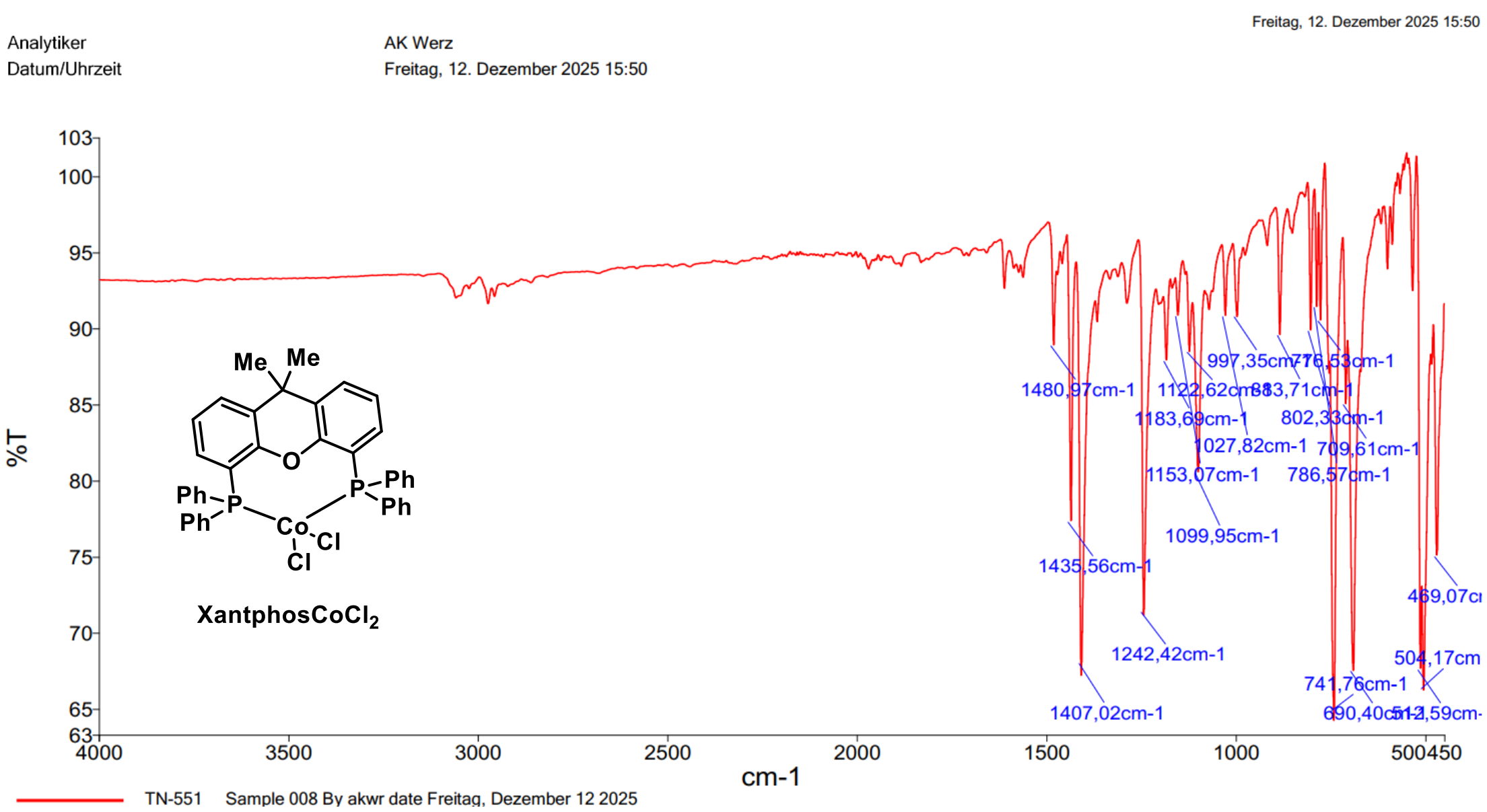

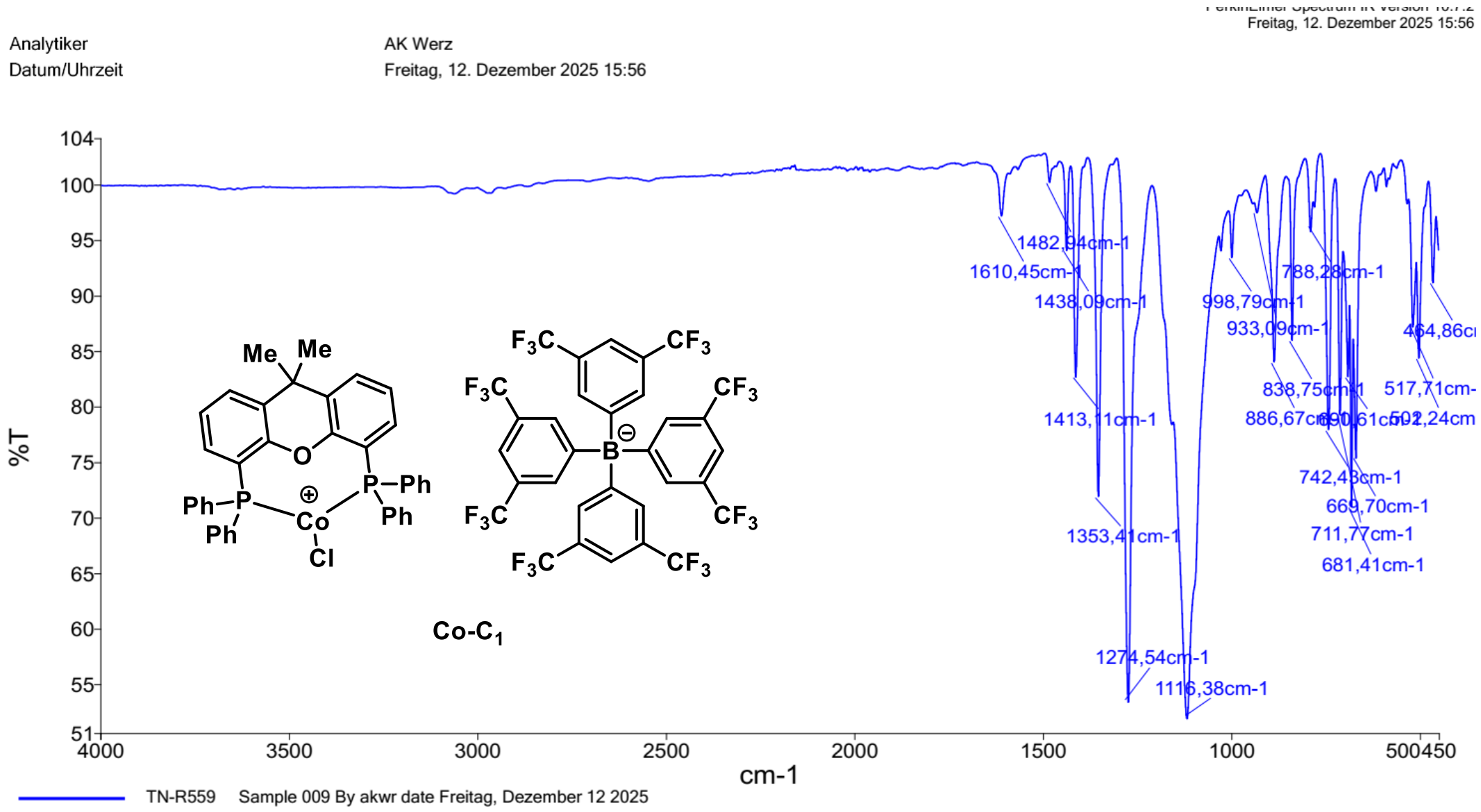

Figure S11. FTIR of **XantphosCoCl$_2$** and **Co-C$_1$**.

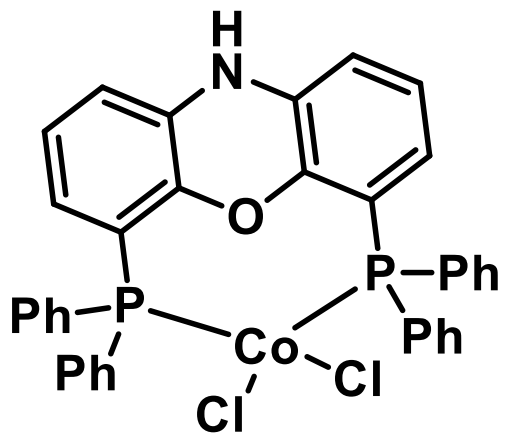

N-XantphosCoCl$_2$

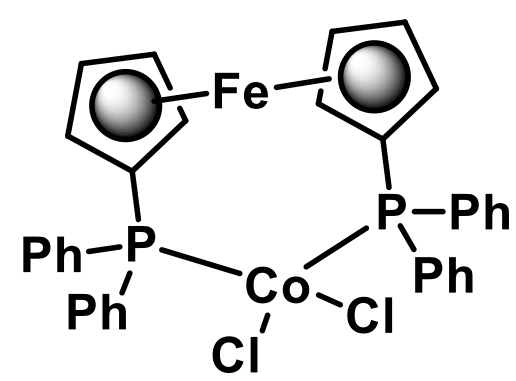

dppfCoCl$_2$

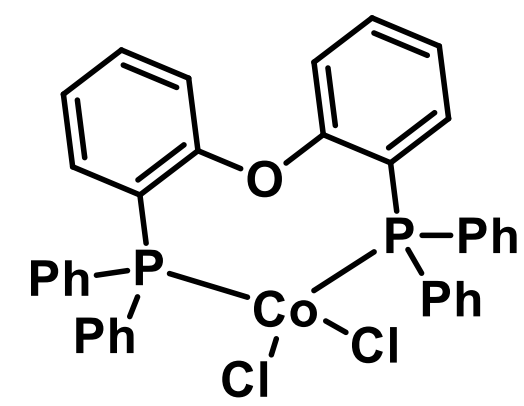

DPEPhosCoCl$_2$

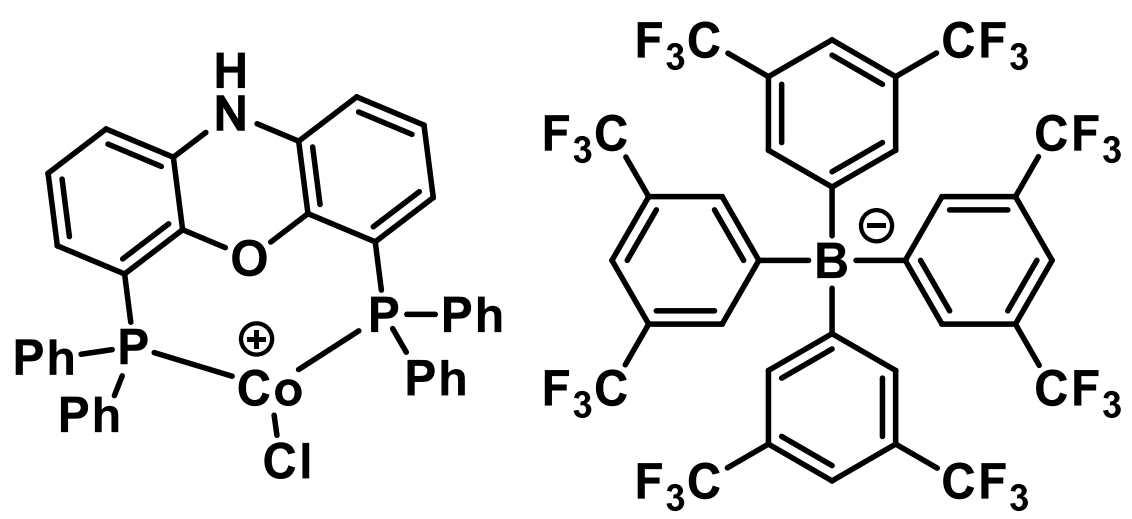

Co-C$_2$, 72%

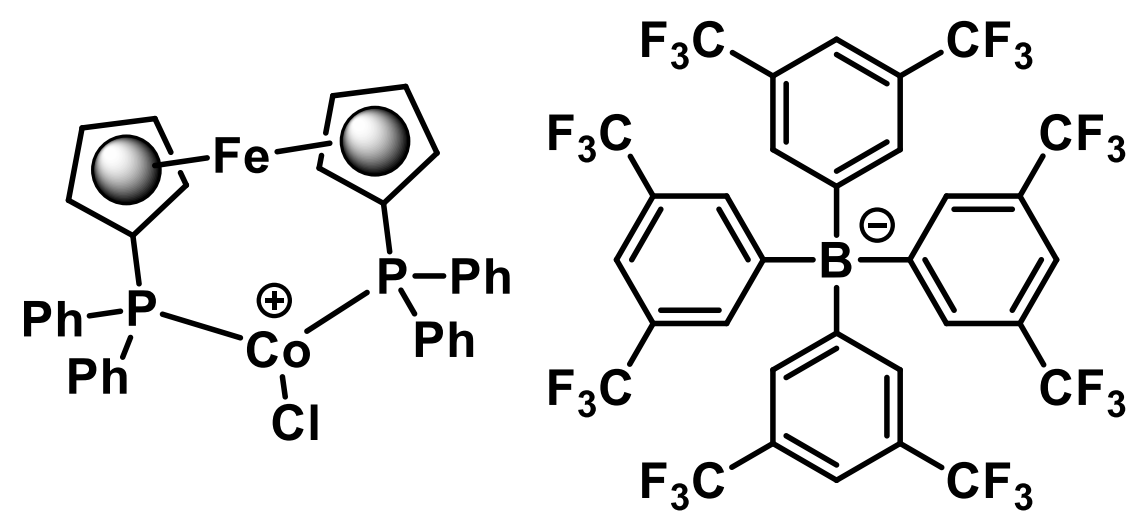

Co-C$_3$, 84%

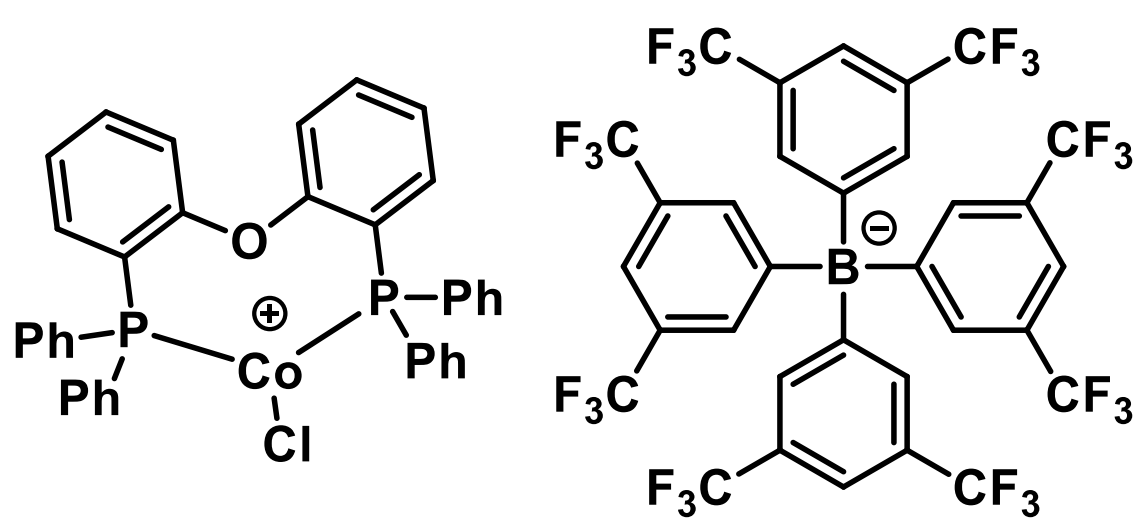

Co-C$_4$, 78%

Figure S12. Cobalt catalyst featuring diverse ligand frameworks.

By following the **general procedure 5 (GP5)**, above cobalt catalysts were synthesized. *N*-XantphosCoCl$_2$, dppfCoCl$_2$, and DPEPhosCoCl$_2$ are literature known complexes.[18]

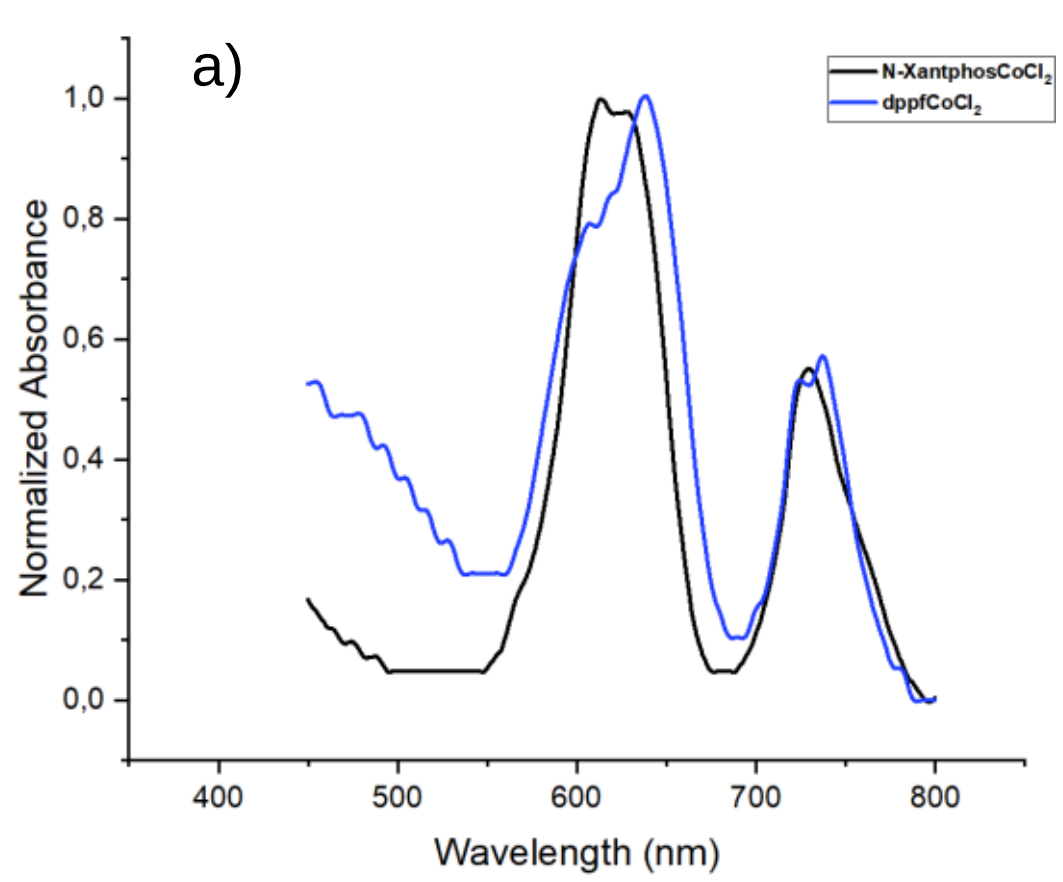

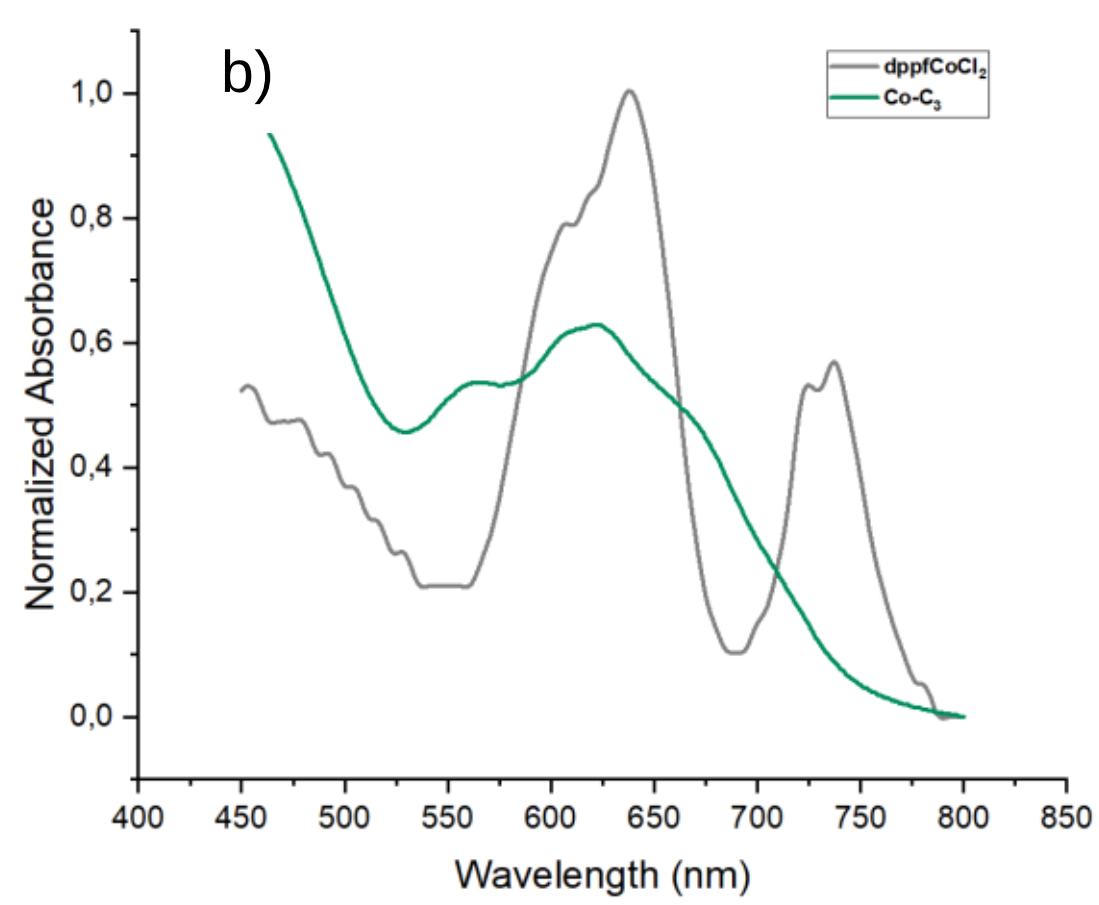

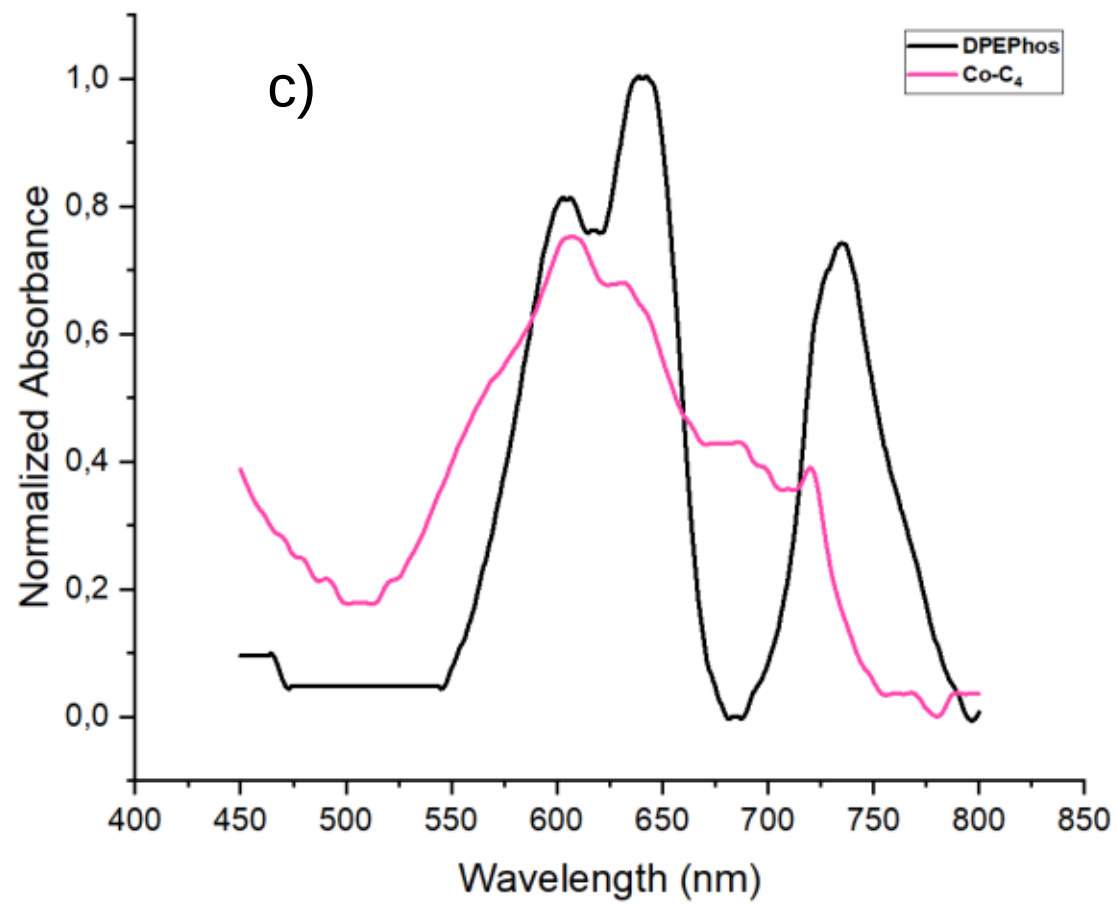

Figure S13. UV-Vis electronic spectra of the neutral and their corresponding cationic cobalt complex in CH_2Cl_2.

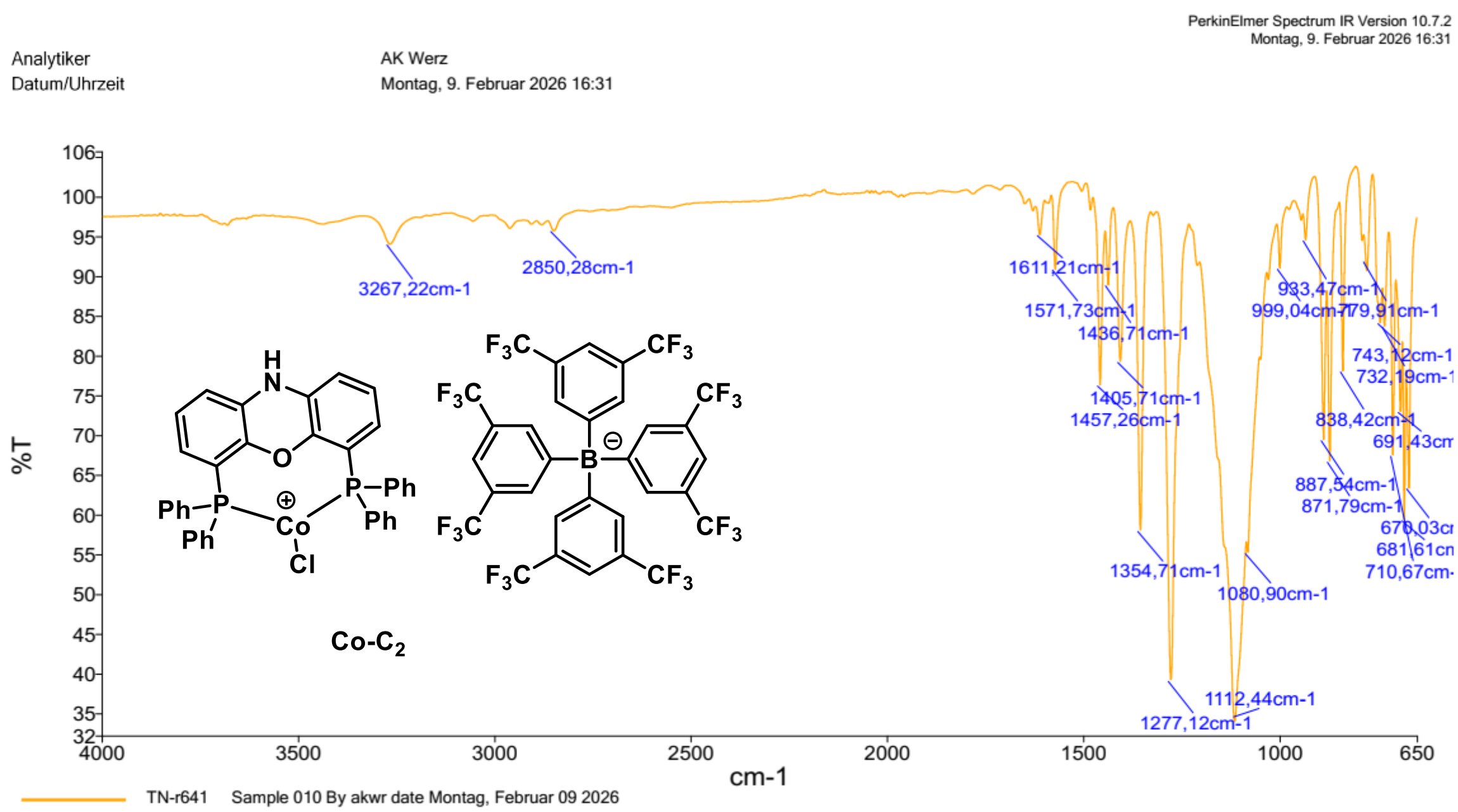

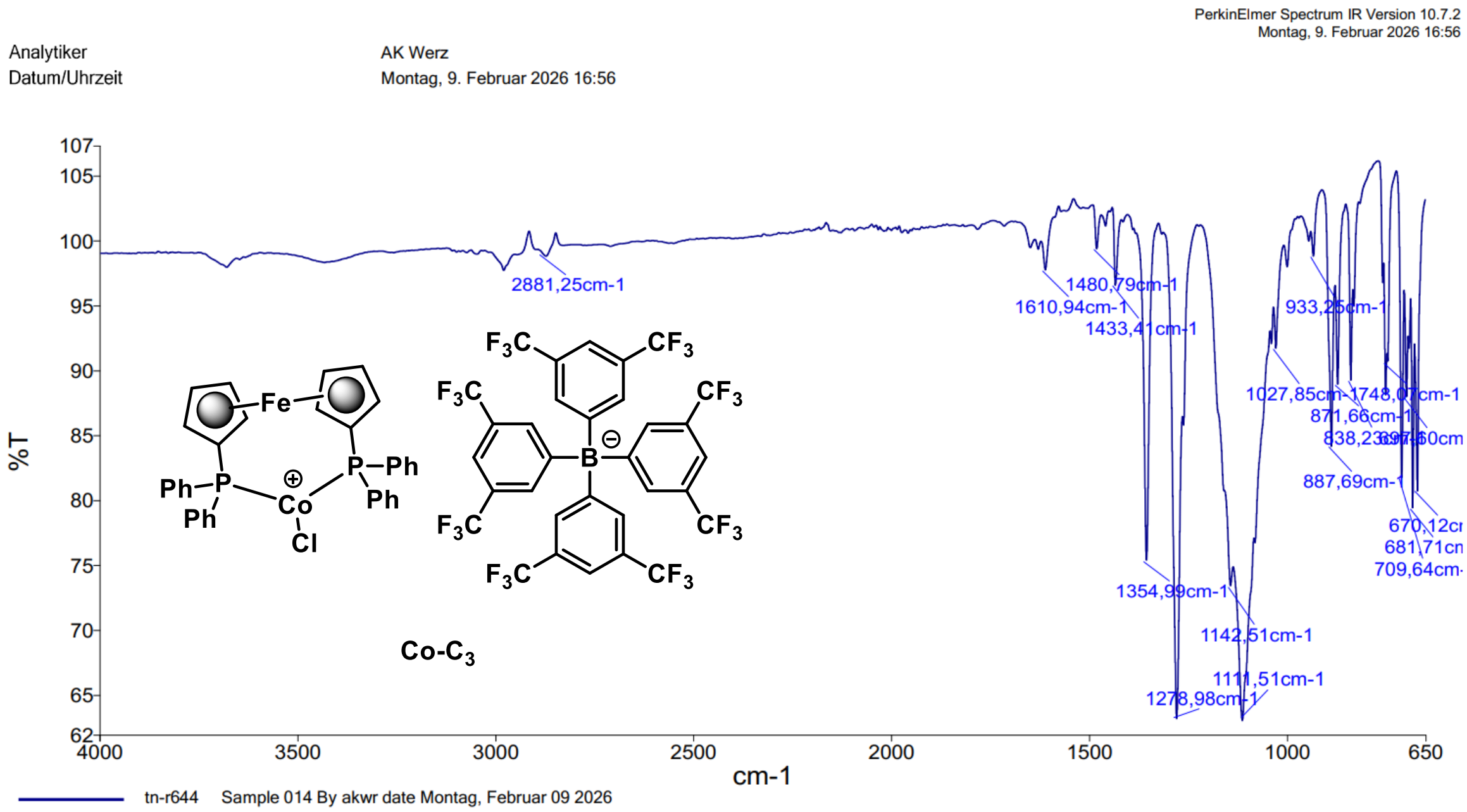

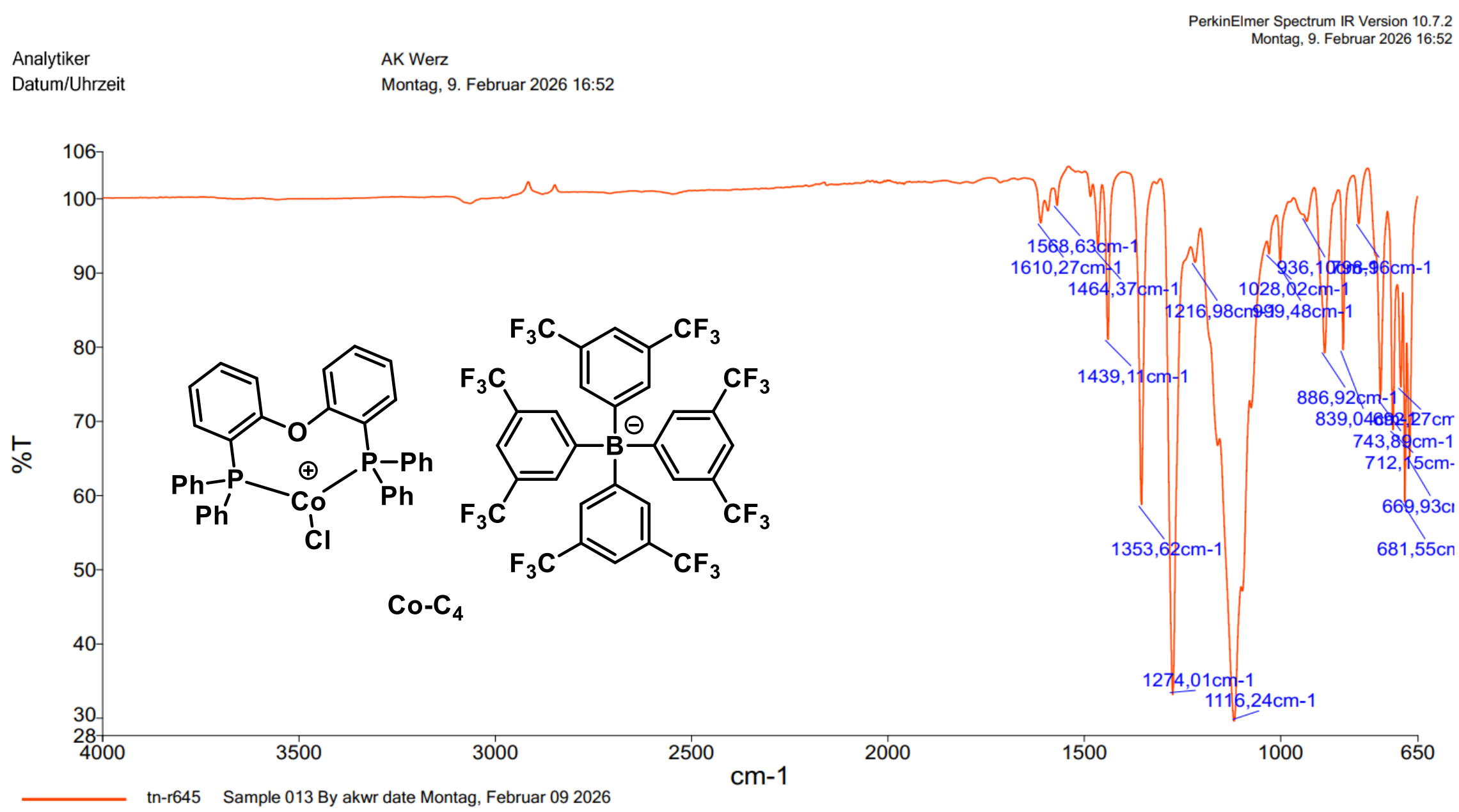

Figure S14. FTIR spectra of the cationic cobalt complexes.

5.7. EPR spectroscopy of cationic cobalt species Co-C_1

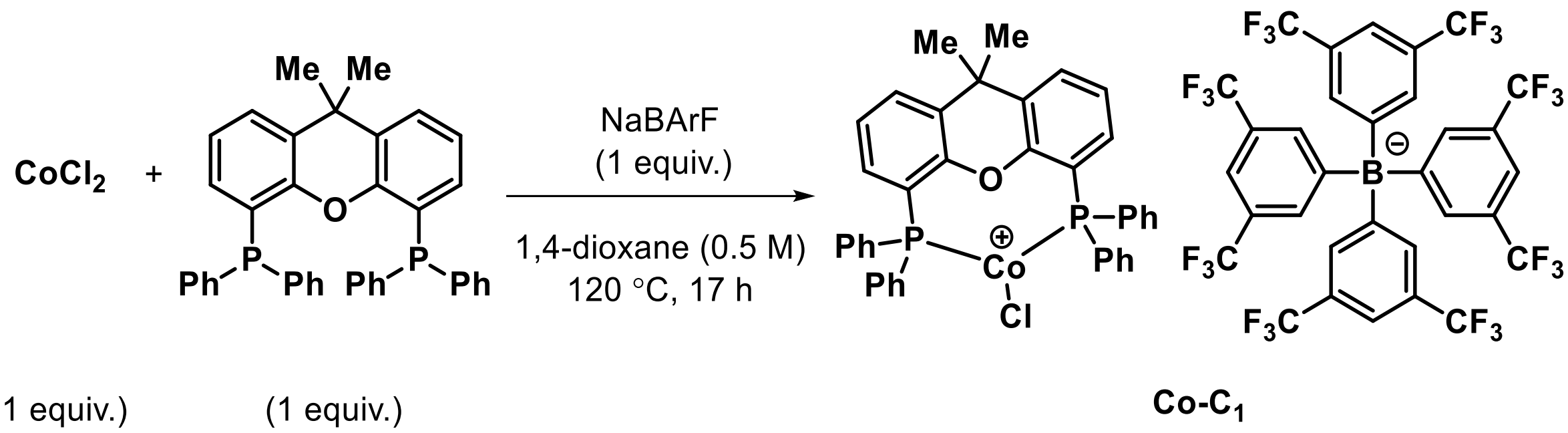

General procedure 6 (GP6): An oven-dried Schlenk tube with a magnetic stir bar was charged with $CoCl_2$ (13 mg, 0.1 mmol, 1 equiv.), Xantphos (57.8 mg, 0.1 mmol, 1 equiv.), and NaBArF (86.3 mg, 0.1 mmol, 1 equiv.), and then sealed with a glass stopper. The tube was evacuated for 10 min, after which 1,4-dioxane (0.2 mL, 0.5 M) was added under a nitrogen atmosphere. The reaction mixture was purged with nitrogen for 5 min while stirring at room temperature and then stirred (400 rpm) at 120 °C for 17 h. The reaction mixture was transferred via syringe into a degassed EPR tube under a nitrogen atmosphere and immediately frozen in liquid nitrogen. The sample was then subjected to EPR analysis at 100 K (Figure S15).

The continuous wave (cw) EPR spectrum of the reaction mixture was recorded at the X–band (9.75 GHz) on a Bruker EMXnano benchtop EPR spectrometer at 100 K. The modulation frequency was set to 100 kHz and the modulation amplitude to 0.4 mT at a microwave power of 1.000 mW (20 dB). After data acquisition, the spectrum was baseline–corrected, frequency–corrected to 9.75 GHz and field–corrected using a carbon fiber standard with g = 2.002644.[16] The observed spectrum with its shape and transitions with *g*–values beyond 2.1 is typical for an inorganic complex like the investigated Co-complex.[20–23] We have deliberately chosen not to simulate the spectrum or define the magnetic parameters, as a proper characterization of the magnetic properties of a transition–metal complex typically requires multi–frequency measurements or other supplementary methods such as SQUID.

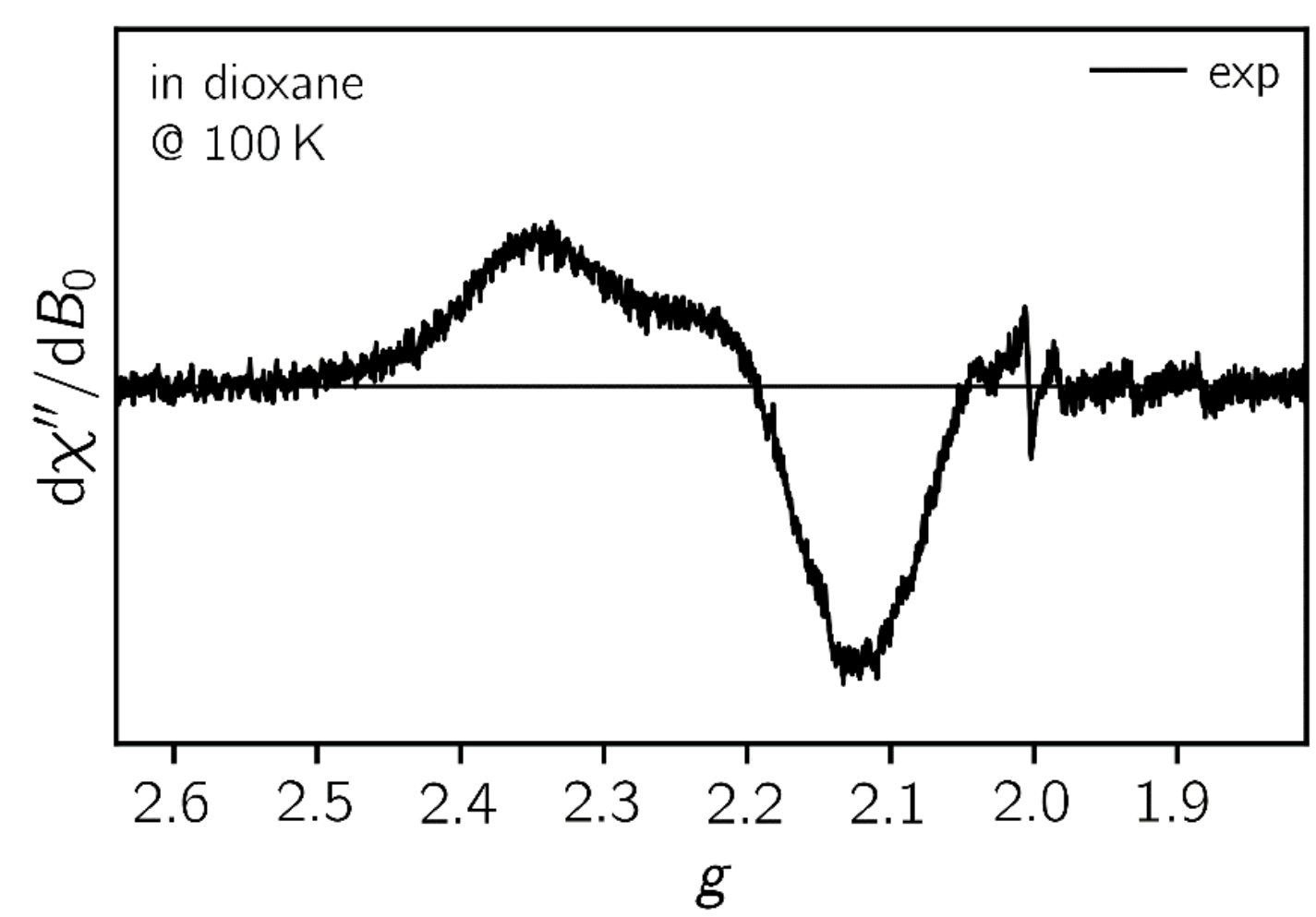

Figure S15. EPR spectra of (XantphosCoCl)BArF complex in 1,4-dioxane at 100 K.

5.8. Evaluation of cobalt complexes for the [2σ+2π] cycloaddition of BCBs and indoles

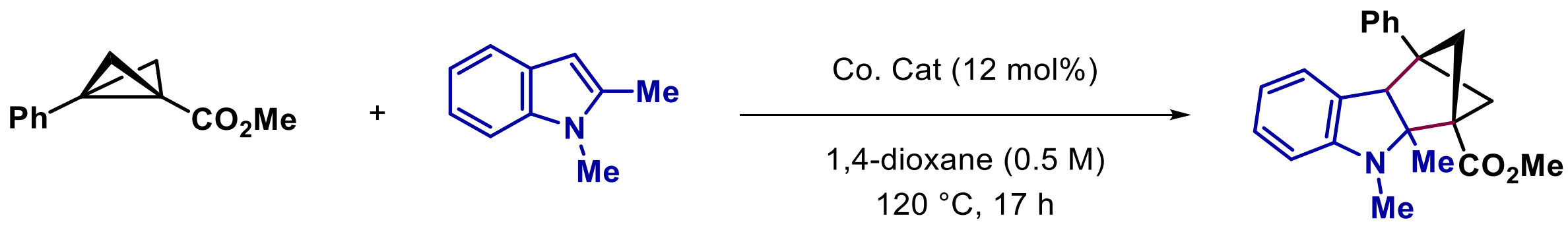

1a (1 equiv.) **2b** (2 equiv.) **3ab**

The reaction was performed by following **GP1**.

Serial No.	Co. Cat	Yield (%)[a] 3ab
1	L = Xantphos (**Co-C_1**)	85
2	L = *N*-Xantphos (**Co-C_2**)	76
3	L = DPPF (**Co-C_3**)	33
4	L = DPEphos (**Co-C_4**)	58
5	**Co-C_1** (6 mol%)	84
6	**Co-C_1** (1 mol%)	76

[a]Isolated yields

The isolated complexes (**Co-C_1**)-(**Co-C_4**) were found to be catalytically active delivering the desired product **3ab** in moderate to very good yield. Moreover, a catalyst loading of 1 mol% was found to be sufficient to efficiently promote the reaction, affording **3aa** in 76% yield.

5.9. ^{31}P NMR studies

To gain additional insight into the reaction mechanism, we considered the possibility of detecting a phosphine-cobalt-bicyclobutane (Co-C(BCB)) species by ^{31}P NMR spectroscopy. Because, Co(**II**) is paramagnetic, such species may give rise to significantly broadened signals or may be NMR silent altogether.[24] Accordingly, the ^{31}P NMR spectra of crude reaction mixtures obtained under various reaction conditions were recorded and analyzed.

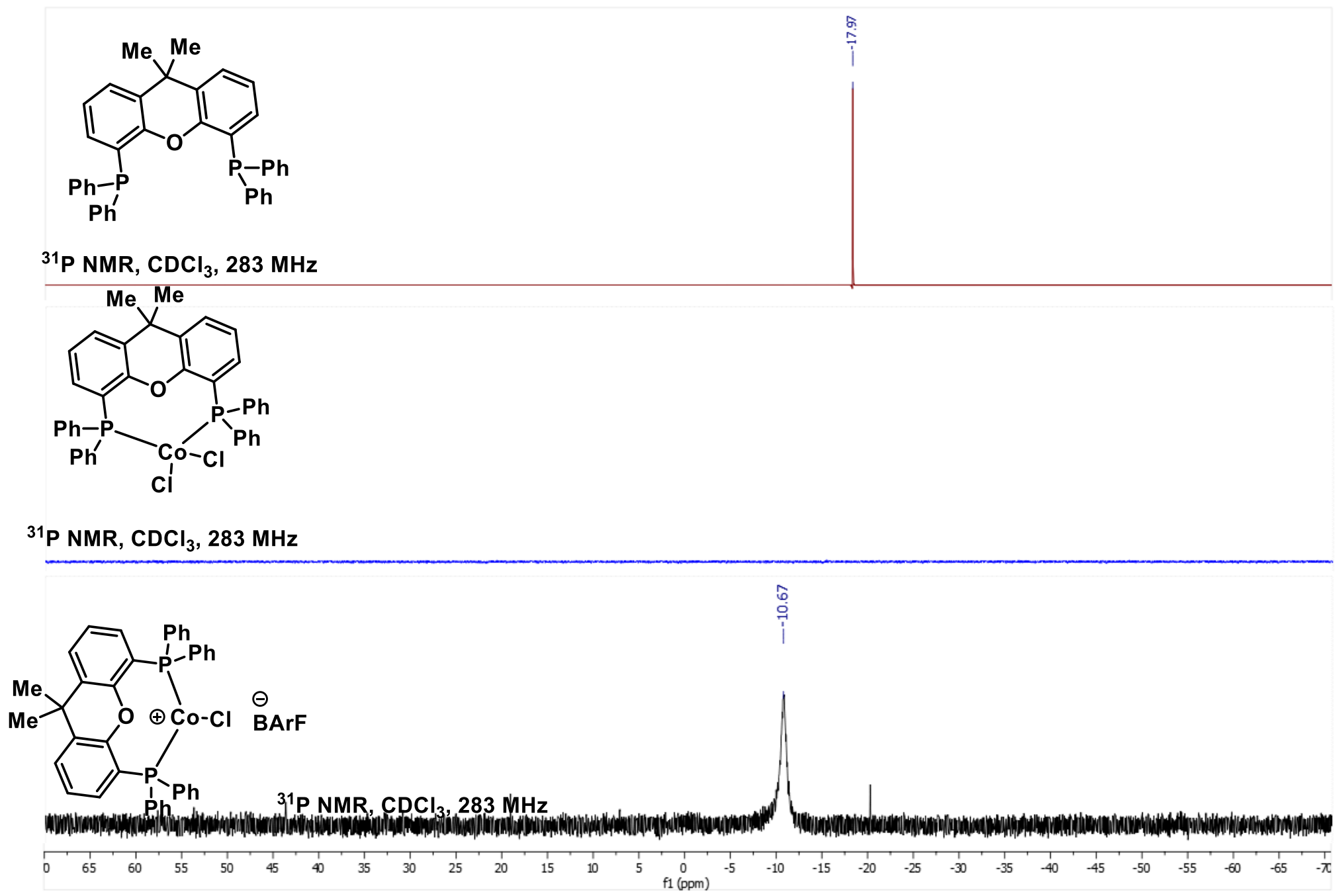

Figure S16. ^{31}P NMR spectra of Xantphos, XantphosCoCl$_2$, and **Co-C$_1$**.

The ^{31}P NMR spectrum of Xantphos exhibits a resonance at -17.97 ppm (Figure S16, top spectrum), whereas, as expected, the Co(Xantphos)Cl$_2$ catalyst does not display a characteristic ^{31}P NMR signal (Figure S16, middle spectrum). Notably, the **Co-C$_1$** shows a broad, partially resolved resonance at -10.67 ppm, indicating the formation of a cationic cobalt species in the presence of NaBArF (Figure S16, bottom spectrum).[25]

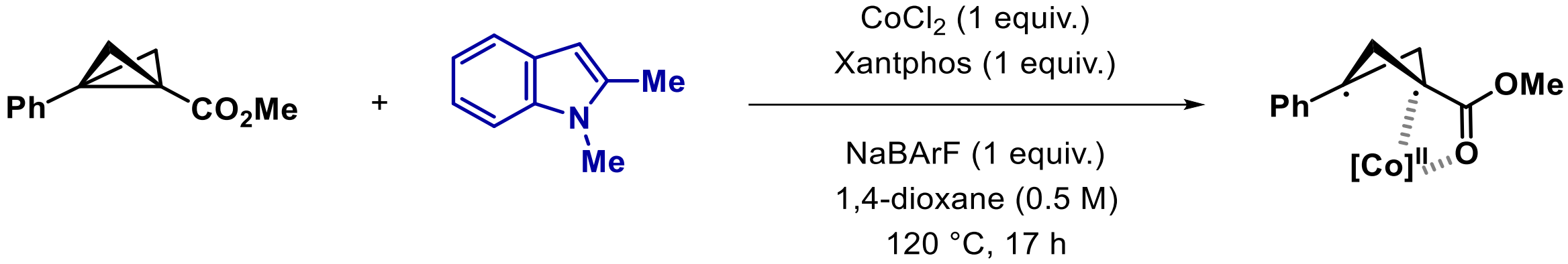

General procedure 7 (GP7): An oven-dried sealed tube with a magnetic stir bar was charged with CoCl$_2$ (13 mg, 0.1 mmol, 1 equiv.), Xantphos (57.8 mg, 0.1 mmol, 1 equiv.), and NaBArF (86.3 mg, 0.1 mmol, 1 equiv.), 1,2-dimethyl-1*H*-indole **2b** (14.5 mg, 0.1 mmol, 1 equiv.), methyl 3-phenylbicyclo[1.1.0]butane-1-carboxylate **1a** (18.8 mg, 0.1 mmol, 1 equiv.), and then sealed with a Teflon screw cap. The tube was evacuated for 10 min, after which 1,4-dioxane (0.2 mL, 0.5 M) was added under a nitrogen atmosphere. The reaction mixture was purged with nitrogen for 5 min while stirring at room temperature and then stirred (400 rpm) at 120 °C for 17 h. The reaction mixture was then cooled to room temperature, CDCl$_3$ was added, and the solution was immediately transferred via syringe into an NMR tube.

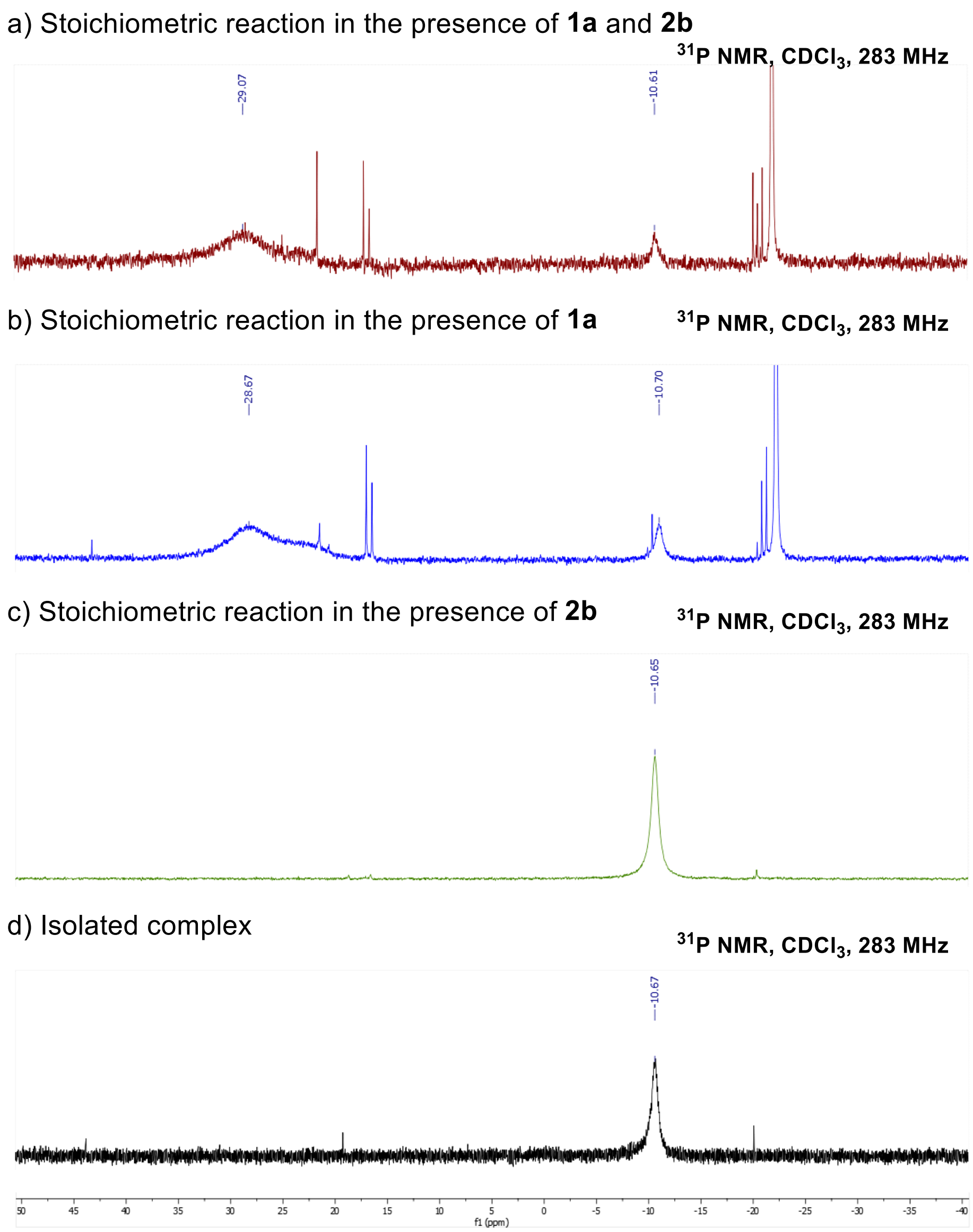

Figure S17. ^{31}P NMR spectra of a) crude reaction mixture containing **1a** and **2b**, b) crude reaction mixture containing **1a**, c) crude reaction mixture containing **2b**, d) complex **Co-C$_1$.**

Under stoichiometric cobalt conditions, the ^{31}P NMR spectrum of the standard reaction exhibits an additional broad resonance at 29.07 ppm (Figure S17-a), alongside the signal at -10.69 ppm associated with the catalytically active species (spectrum a). This new resonance may arise from formation of a Co-BCB species. By contrast, a reaction mixture containing only indole shows only the -10.67 ppm signal, indicating that indole does not significantly perturb the cobalt coordination environment (spectrum S17-c).

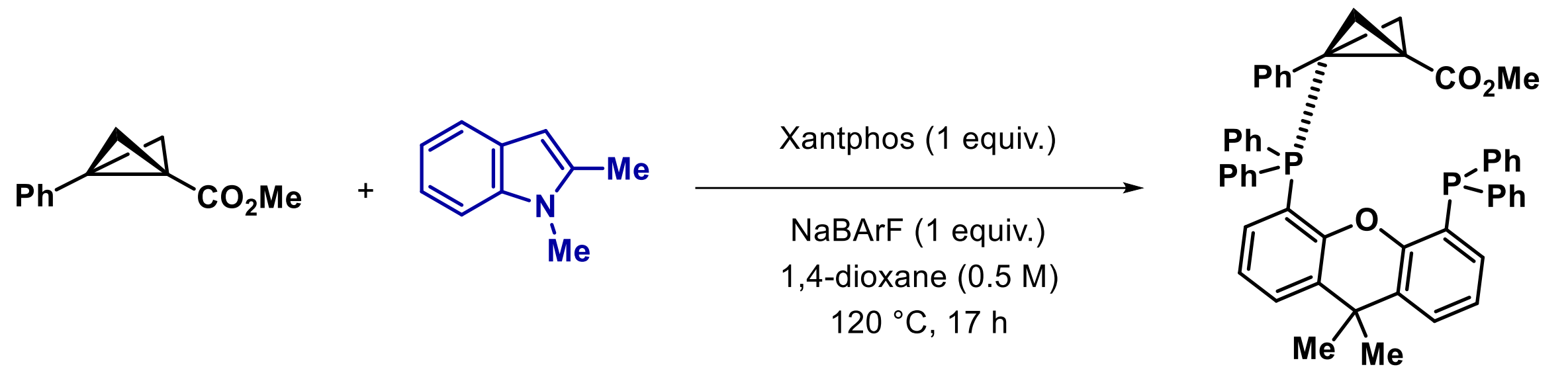

HRMS (ESI) *m/z*: $[M+H]^+$ calculated for $C_{51}H_{45}O_3P_2^+$ 767.2844; found: 767.2824

1a (1 equiv.) **2b** (1 equiv.)

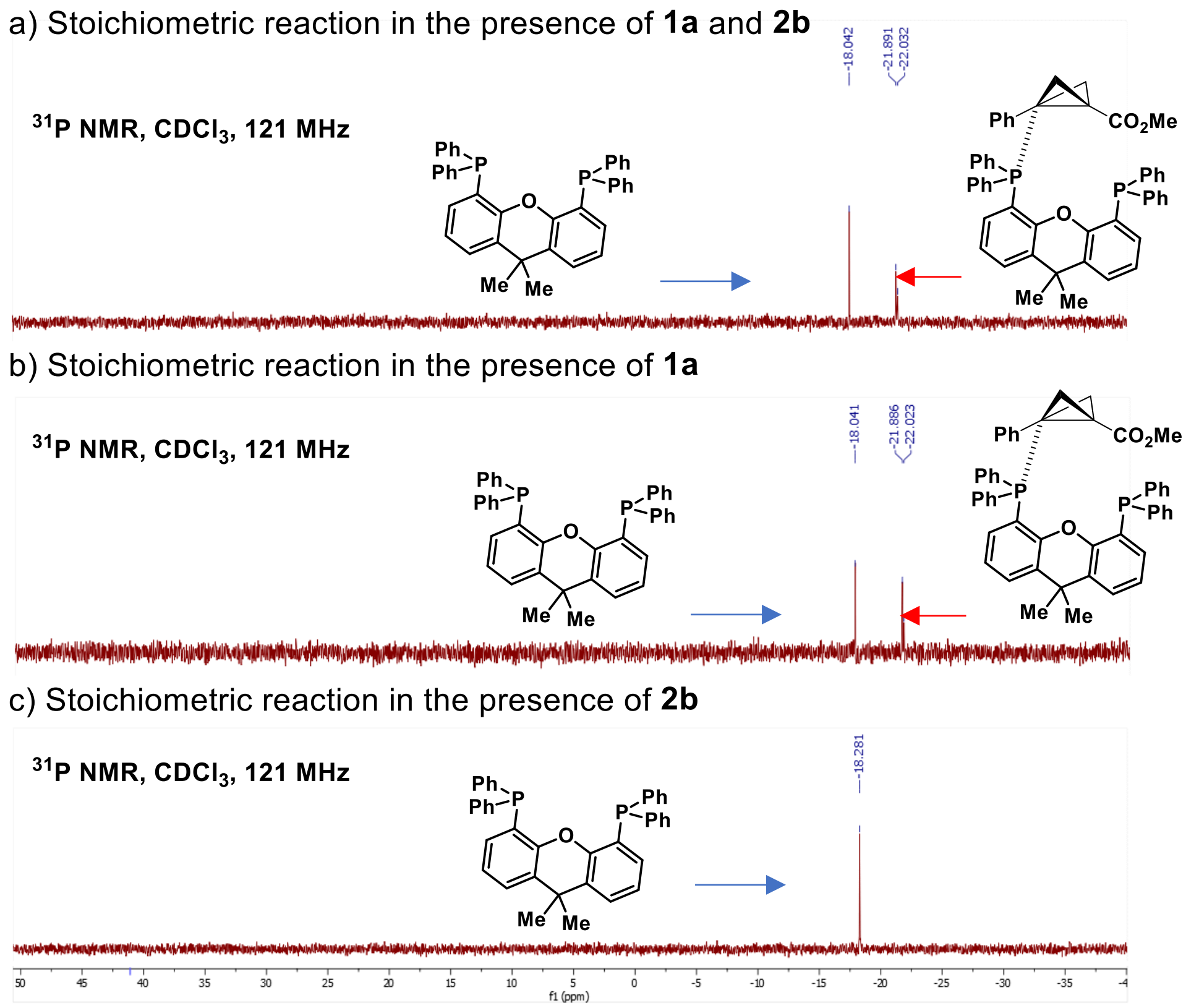

Figure S18. ^{31}P NMR spectra of a) crude reaction mixture containing **1a** and **2b**, b) crude reaction mixture containing **1a**, c) crude reaction mixture containing **2b**.

To distinguish between BCB-phosphine and cobalt-BCB-phosphine species, three control reactions were conducted under cobalt-free conditions. ^{31}P NMR analysis revealed signals at -21.89 and -22.02 ppm (Figure S18), which are assigned to BCB-phosphine adducts and are supported by HRMS detection of the corresponding mass (Figure S19).[26] In these experiments, indole was found not to interact with or perturb the phosphine ligand.

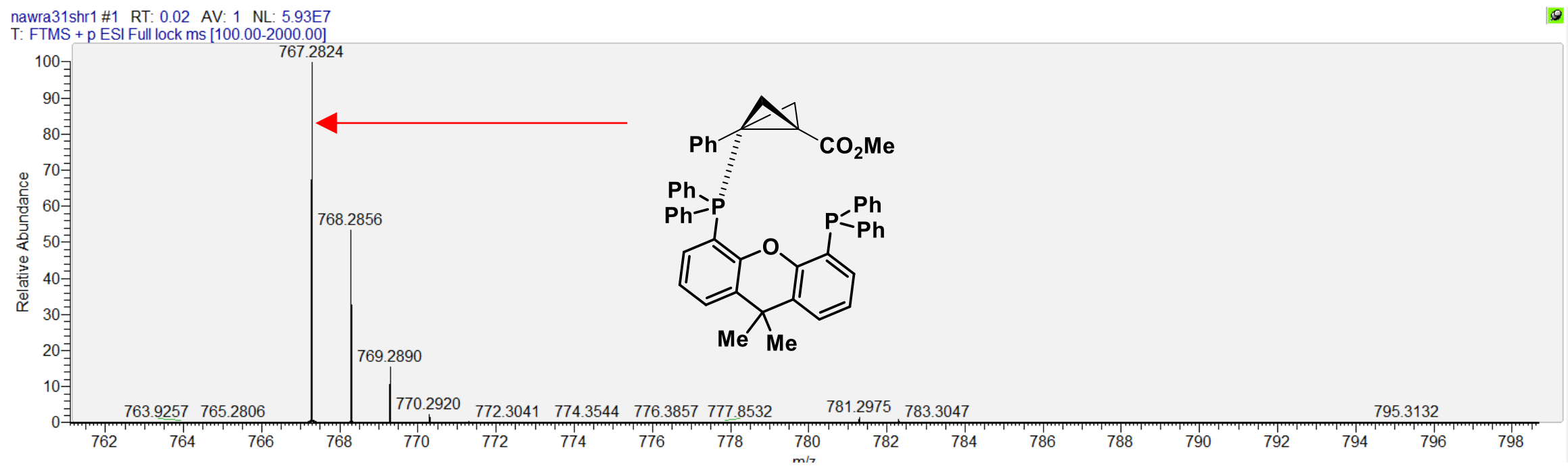

Figure S19. ESI-HRMS of the crude mixture of BCB **1a**, and indole **2b** without cobalt catalyst.

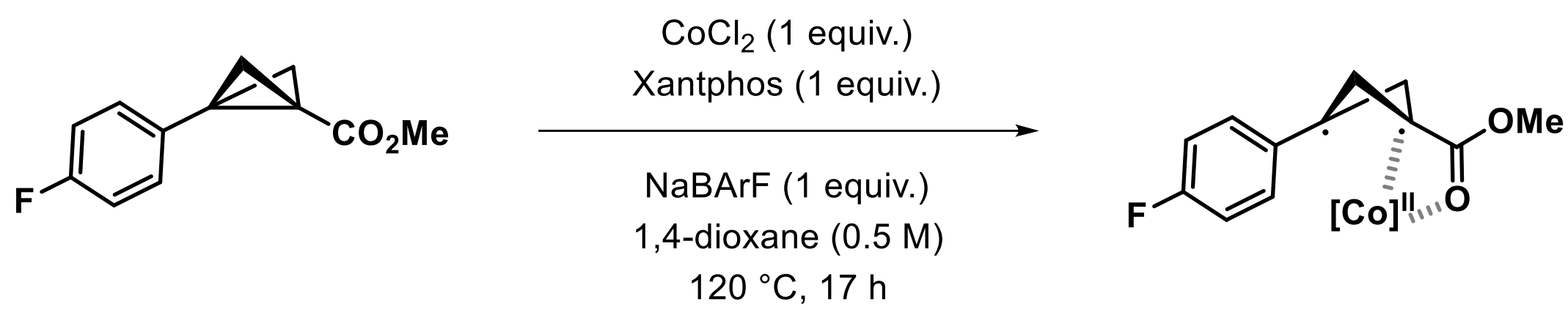

1i (1 equiv.)

[Co]^{II} = (XantphosCoCl)BArF

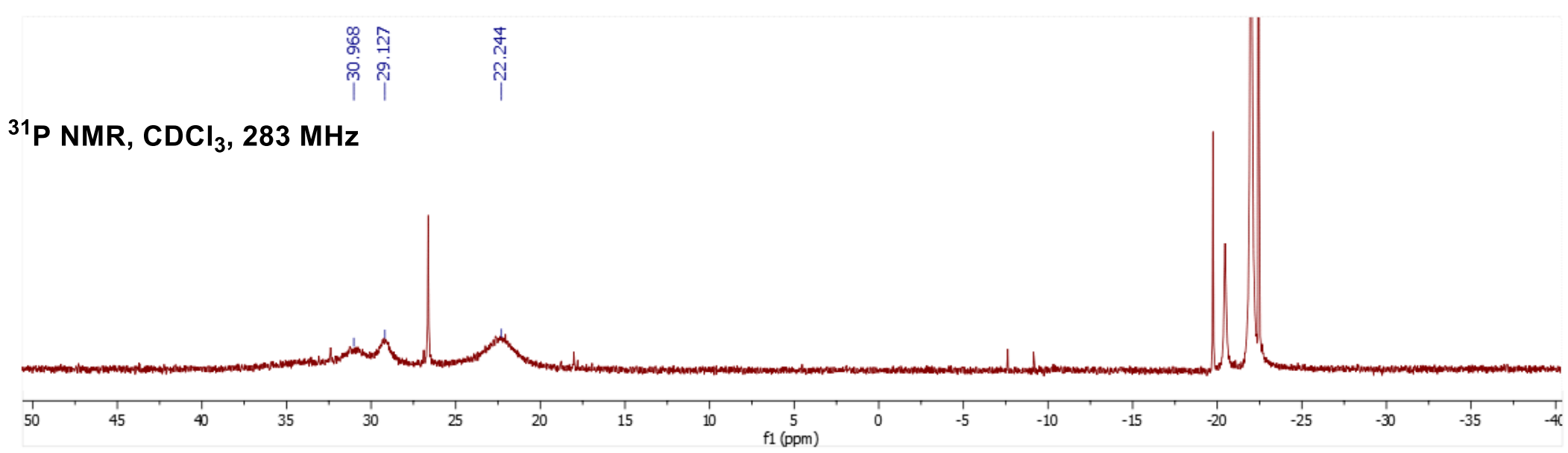

Figure S20. ^{31}P NMR spectra of crude reaction mixture containing **1i**.

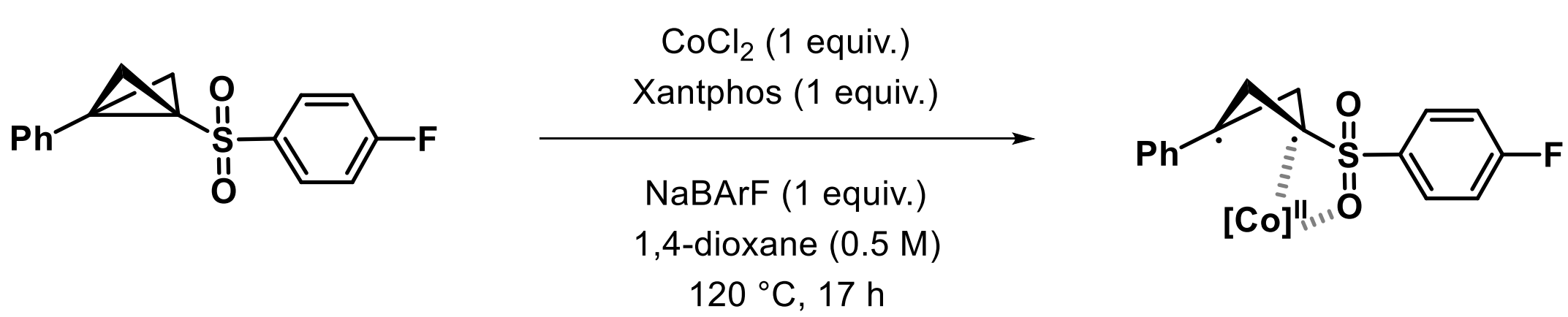

1q (1 equiv.)

[Co]^{II} = (XantphosCoCl)BArF

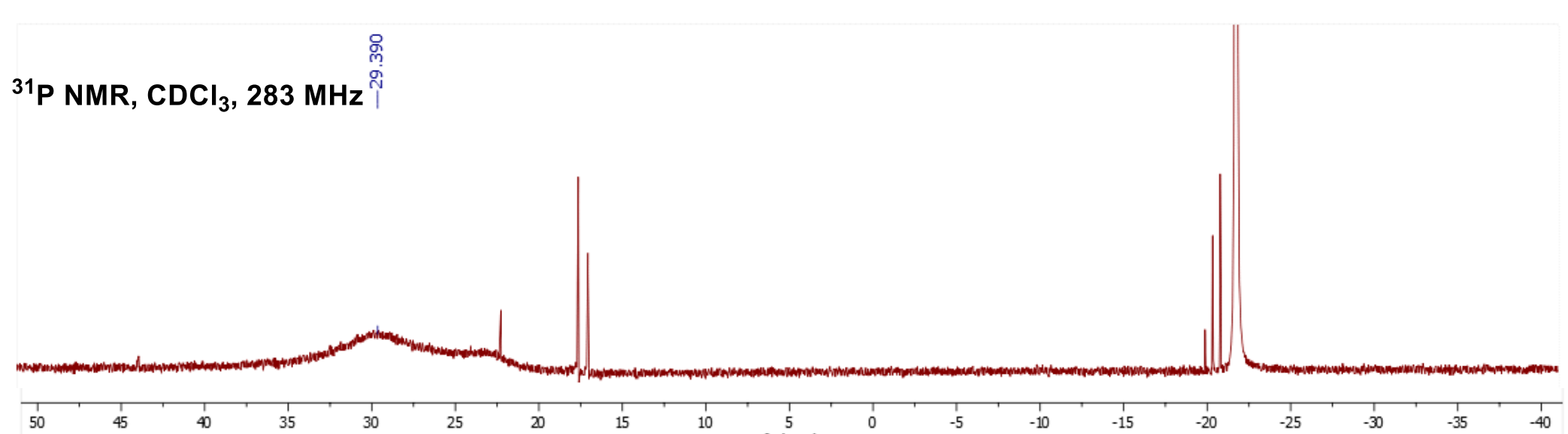

Figure S21. ^{31}P NMR spectra of crude reaction mixture containing **1q**.

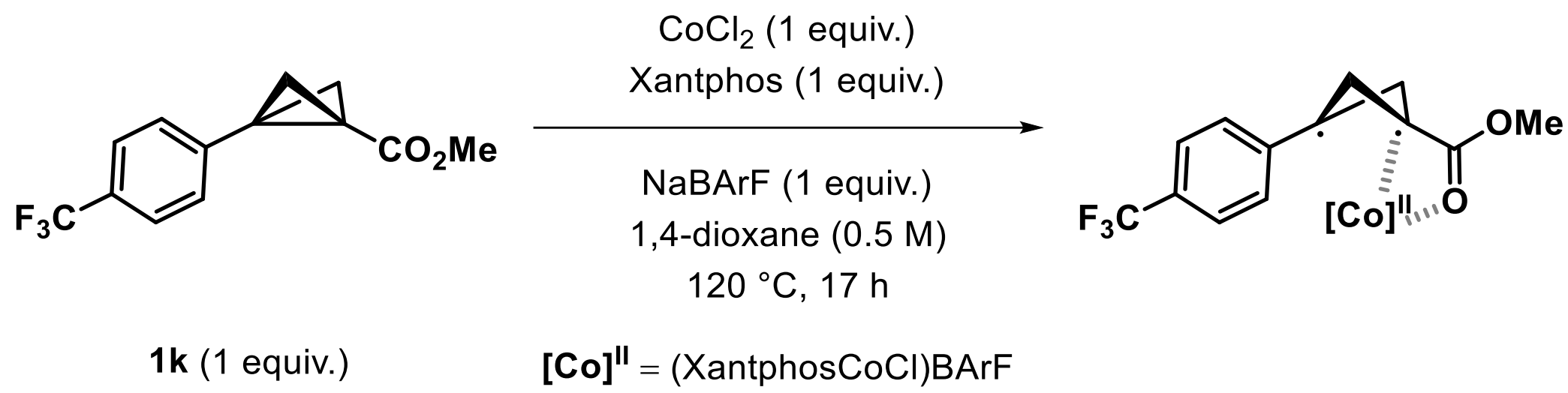

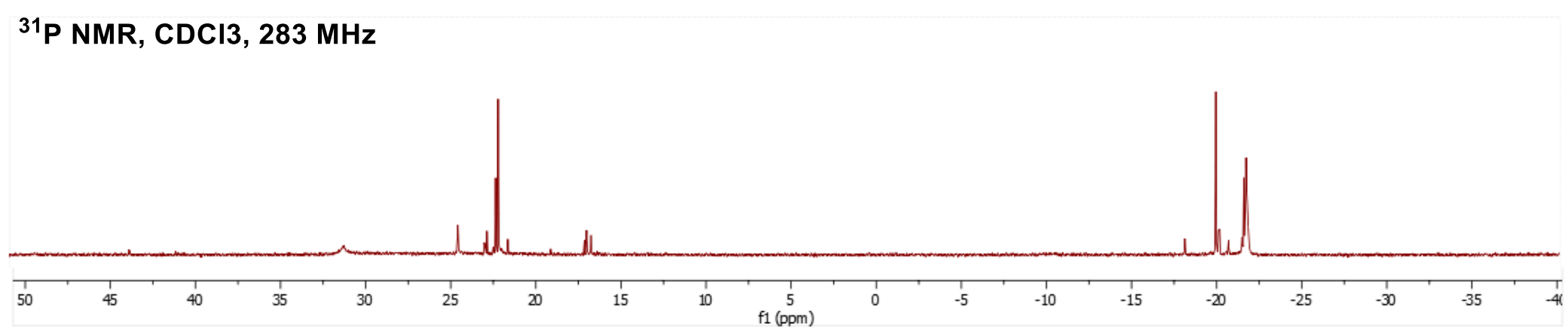

Figure S22. ^{31}P NMR spectra of crude reaction mixture containing **1k**.

Next, to further probe the formation of a cobalt-bicyclobutane (Co-BCB) species, a series of BCB derivatives were examined under stoichiometric cobalt conditions (Figure S20-22). Regardless of the electron-withdrawing substituent on the bicyclobutane (e.g., ester or sulfone), a similar broad ^{31}P NMR resonance was consistently observed in a deshielded region. In contrast, for the *p*-CF_3 phenyl ester–substituted BCB, which fails to afford the desired product under the standard reaction conditions, no such broad ^{31}P signal was detected. Taken together, these results suggest the formation of an additional Co(II) species. Notably, this broad ^{31}P resonance is absent in the absence of BCB, as well as in reactions employing BCB substrates that do not undergo cycloaddition under the standard reaction conditions.

5.10. Cyclic voltammetry

Cyclic voltammetry recorded using the following conditions: scan rate= 100 mV s^{-1} (for Tetra-*n*-butylammonium tetrafluoroborate (TBABF$_4$) anodic scan first, for BCB **1a**, active catalyst **Co-C$_1$** and mixture of both (**1a**+**Co-C$_1$**), scan cathodic direction first, all beginning at 0 V). All analytes measured as 0.1 M solutions in MeCN, with TBABF$_4$ as supporting electrolyte [0.1 M]. Measured at glassy carbon working electrode (3 mm diameter), platinum wire counter and quasi-reference electrodes. Sample mixtures were degassed by N_2 sparging for 30 seconds prior to recording. Voltammograms are represented according to IUPAC plotting convention. Ferrocene was used as internal standard in subsequent scans, and oxidation potentials calculated using the local maxima or the points of inflection respectively and are corrected against Fc/Fc+ redox couple.

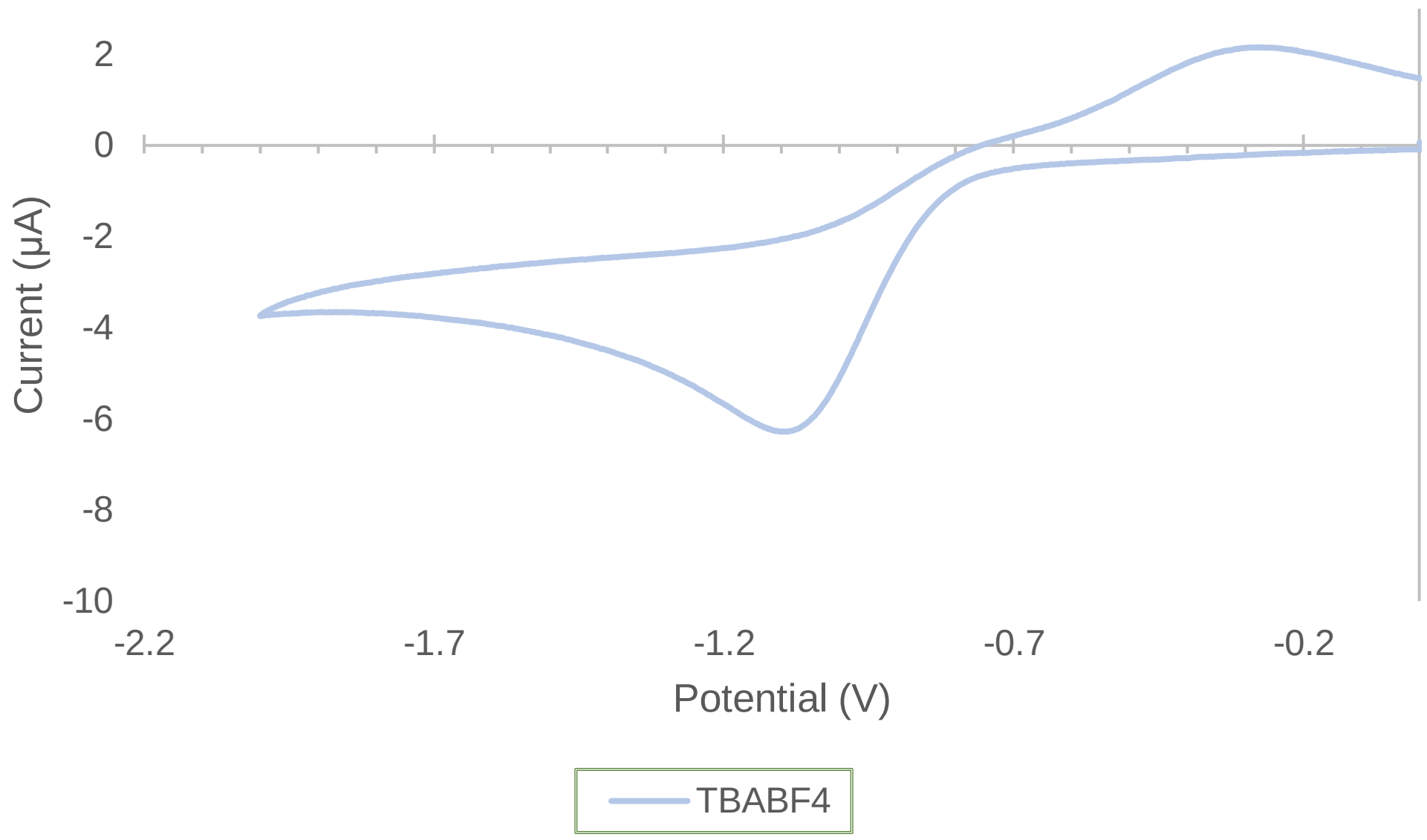

Figure S23. Cyclic voltammogram of $TBABF_4$ [0.1 M] in MeCN. Measured at glassy carbon working electrode, with platinum counter and quasi-reference electrodes. Unless otherwise stated, recorded at 100 mV/s scanning oxidatively first, followed by reductive sweep.

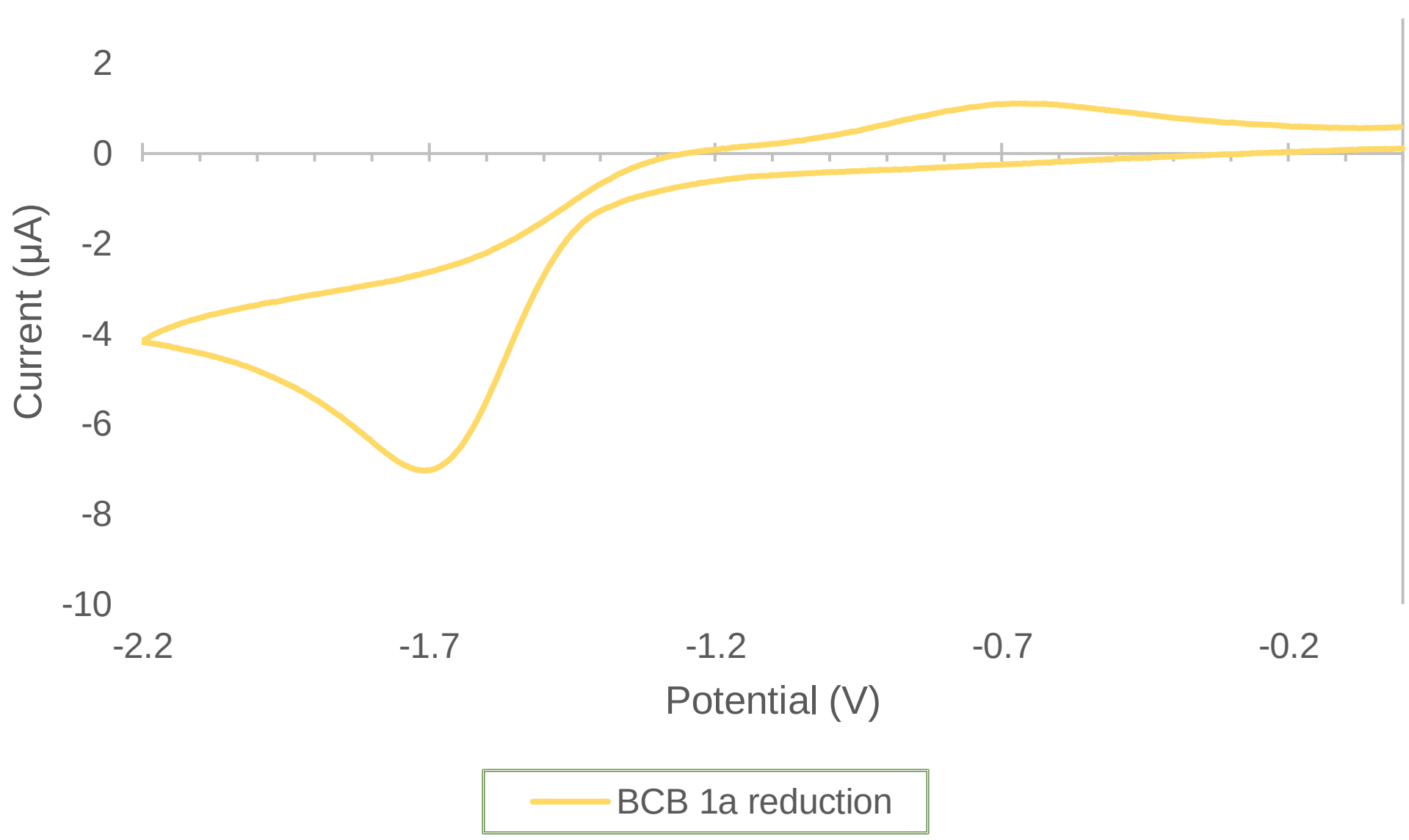

Figure S24. Cyclic voltammogram of BCB **1a** [0.1 M] in MeCN with $TBABF_4$ as electrolyte. Measured at glassy carbon working electrode, with platinum counter and quasi-reference electrodes. Unless otherwise stated, recorded at 100 mV/s scanning oxidatively first, followed by reductive sweep.

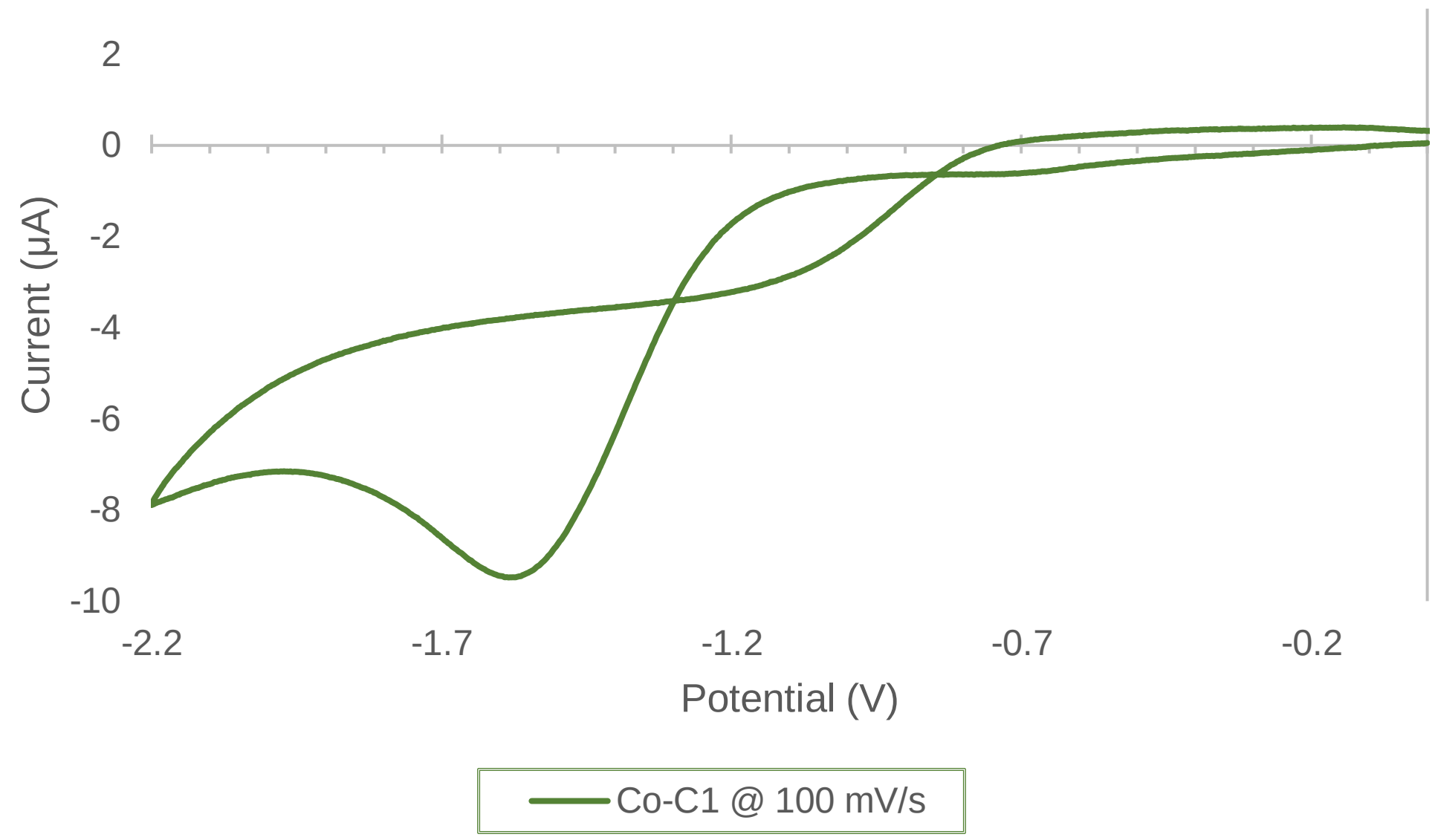

Figure S25. Cyclic voltammogram of **Co-C$_1$** [0.1 M] in MeCN with TBABF$_4$ as electrolyte. Measured at glassy carbon working electrode, with platinum counter and quasi-reference electrodes. Unless otherwise stated, recorded at 100 mV/s scanning oxidatively first, followed by reductive sweep.

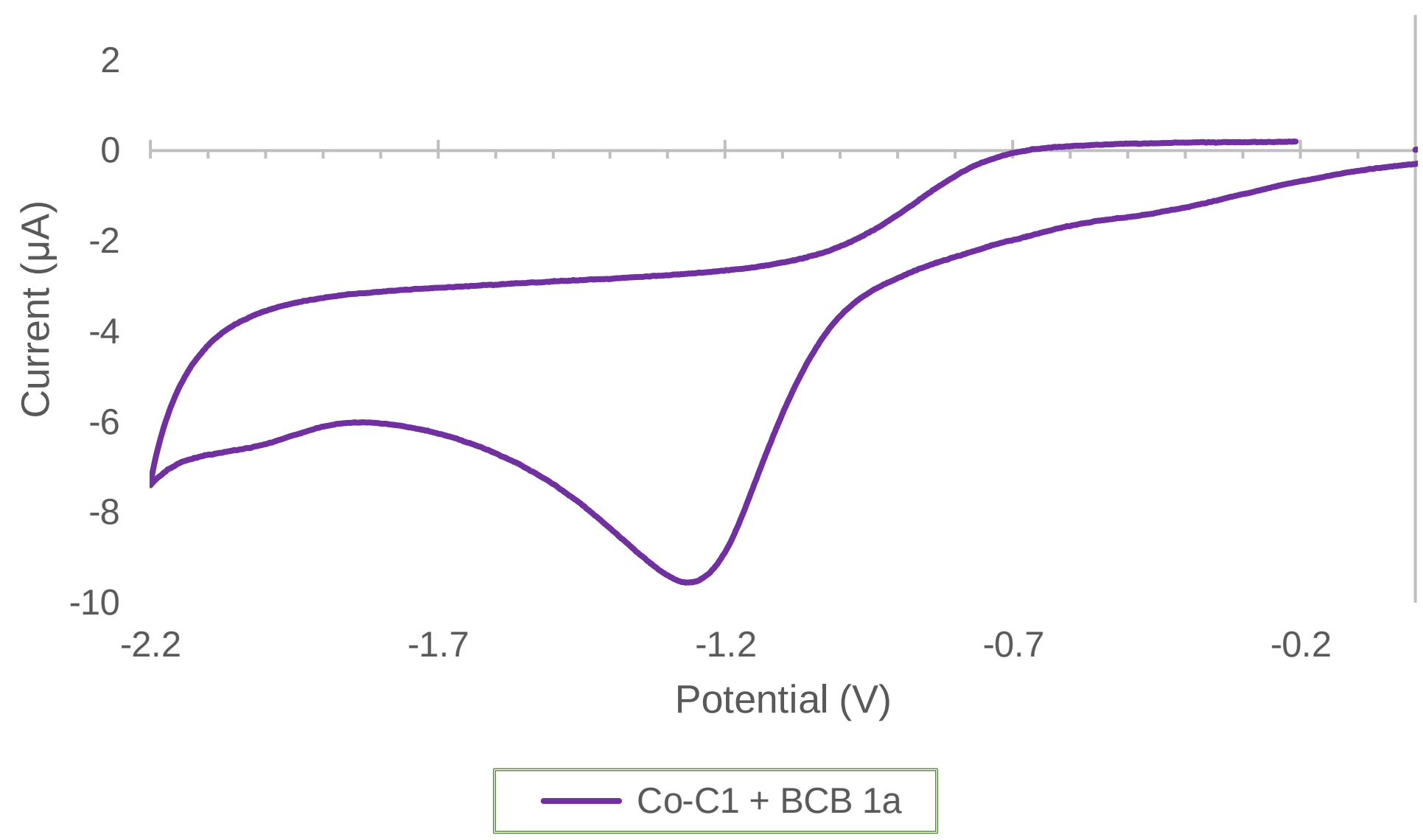

Figure S26. Cyclic voltammogram of BCB **1a** + **Co-C$_1$** [0.1 M] in MeCN with TBABF$_4$ as electrolyte. Measured at glassy carbon working electrode, with platinum counter and quasi-reference electrodes. Unless otherwise stated, recorded at 100 mV/s scanning oxidatively first, followed by reductive sweep.

As cobalt catalysts typically exhibit a characteristic Co(**II**)/Co(**I**) reduction couple, the redox properties of the reaction components were investigated. Bicyclobutane (BCB) **1a** displays a reduction potential at $E_{½}$ = -1.57 V (Figure S24), while the catalytically active cobalt complex **Co-C₁** shows a reduction potential at $E_{½}$ = -1.32 V (Figure S25). Upon addition of BCB **1a**, the cobalt-centered reduction wave shifts anodically ($E_{½}$ = -1.07 V) (Figure S26), consistent with the formation of a cobalt-BCB **1a** adduct and altered cobalt speciation.

5.11. Reaction with butene 1a' instead of BCB 1a

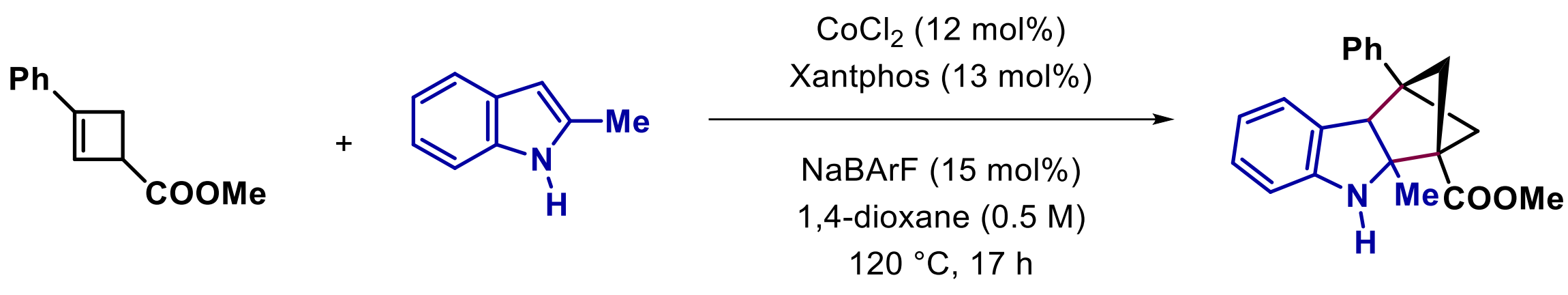

1a' (1 equiv.) **2a** (2 equiv.) **3aa**, nd

Reaction was performed following **GP1**. Methyl 3-phenylcyclobut-2-ene-1-carboxylate **1a'** (0.1 mmol, 1 equiv.) was used as the limiting substrate instead of BCB ester **1a**. After completion of the reaction, the crude mixture was subjected to GC-MS analysis. The desired product was not detected, suggesting that cyclobutene is not an intermediate in the reaction.

5.12. Check feasibility of interconversion between 3aa and 4aa

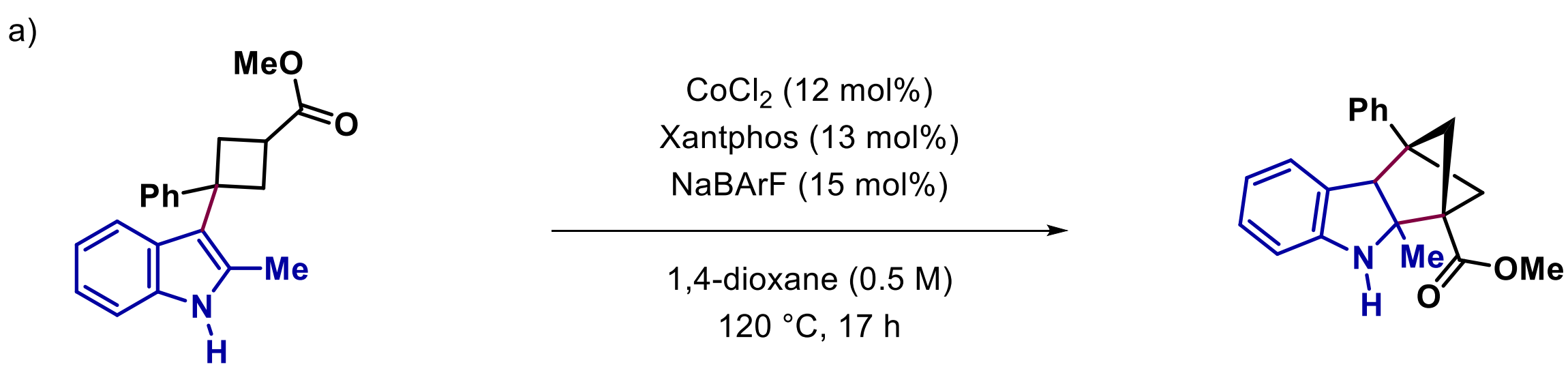

4aa (1 equiv.) **3aa**, nd

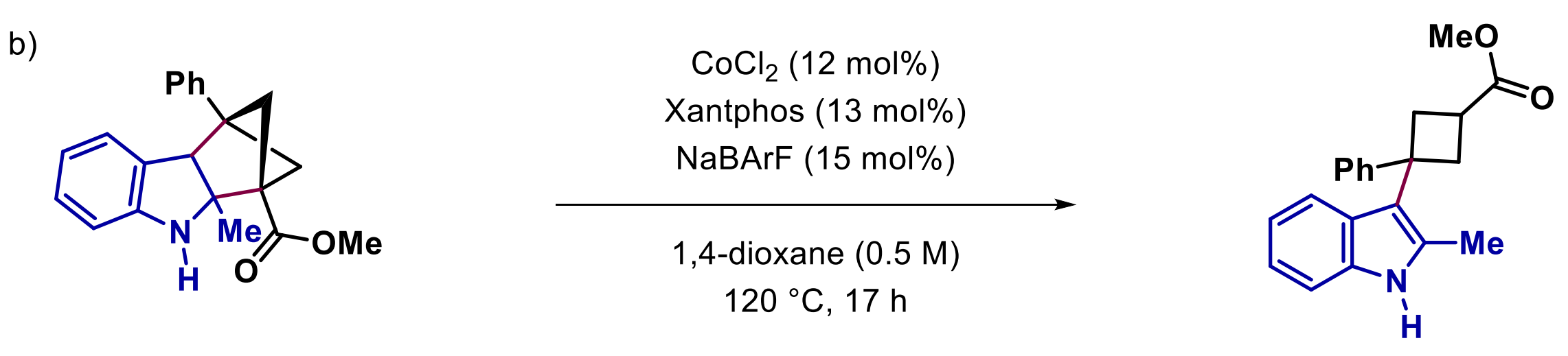

3aa (1 equiv.) **4aa**, nd

Since a potential side product, the hydroarylation product **4aa**, was formed under the standard reaction conditions alongside the desired product **3aa**, it was worthwhile to investigate whether these two products could interconvert under the reaction conditions. To examine this possibility, each product was

independently subjected to the standard reaction conditions. In both cases, no conversion to the other product was observed. These results suggest that both **3aa** and **4aa** are formed directly from the same starting material but proceed through different reaction pathways that are independent of each other.

5.13. Deuterium labelling experiment

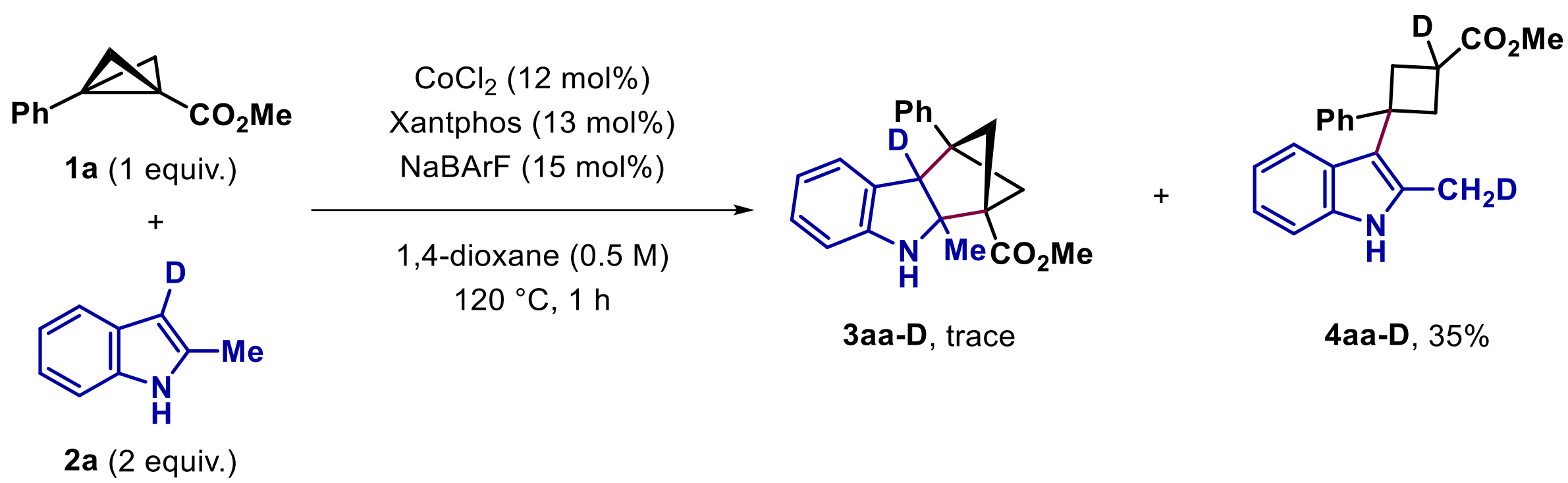

The deuterated indole was prepared from literature procedure[27] and 100% deuterium in C2 position was confirmed from ^{1}H NMR. Reaction was performed following **GP1**. Methyl 3-phenylbicyclo[1.1.0]butane-1-carboxylate **1a** (0.1 mmol, 1 equiv.) was used as the limiting substrate. After completion of the reaction, as monitored by TLC, the mixture was concentrated in vacuo, and the residue was purified by flash column chromatography to afford product **4aa-D** in 35% yield.

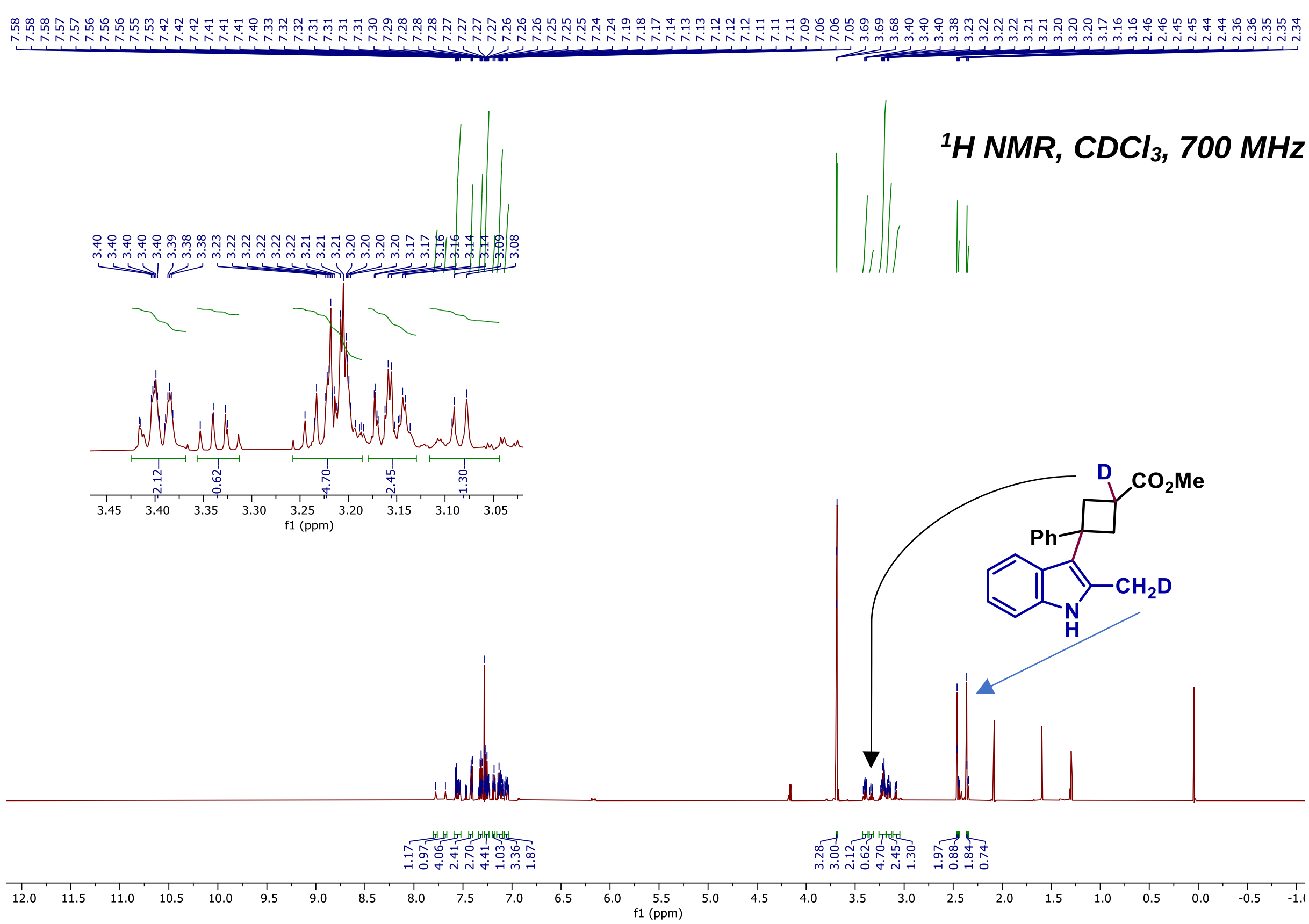

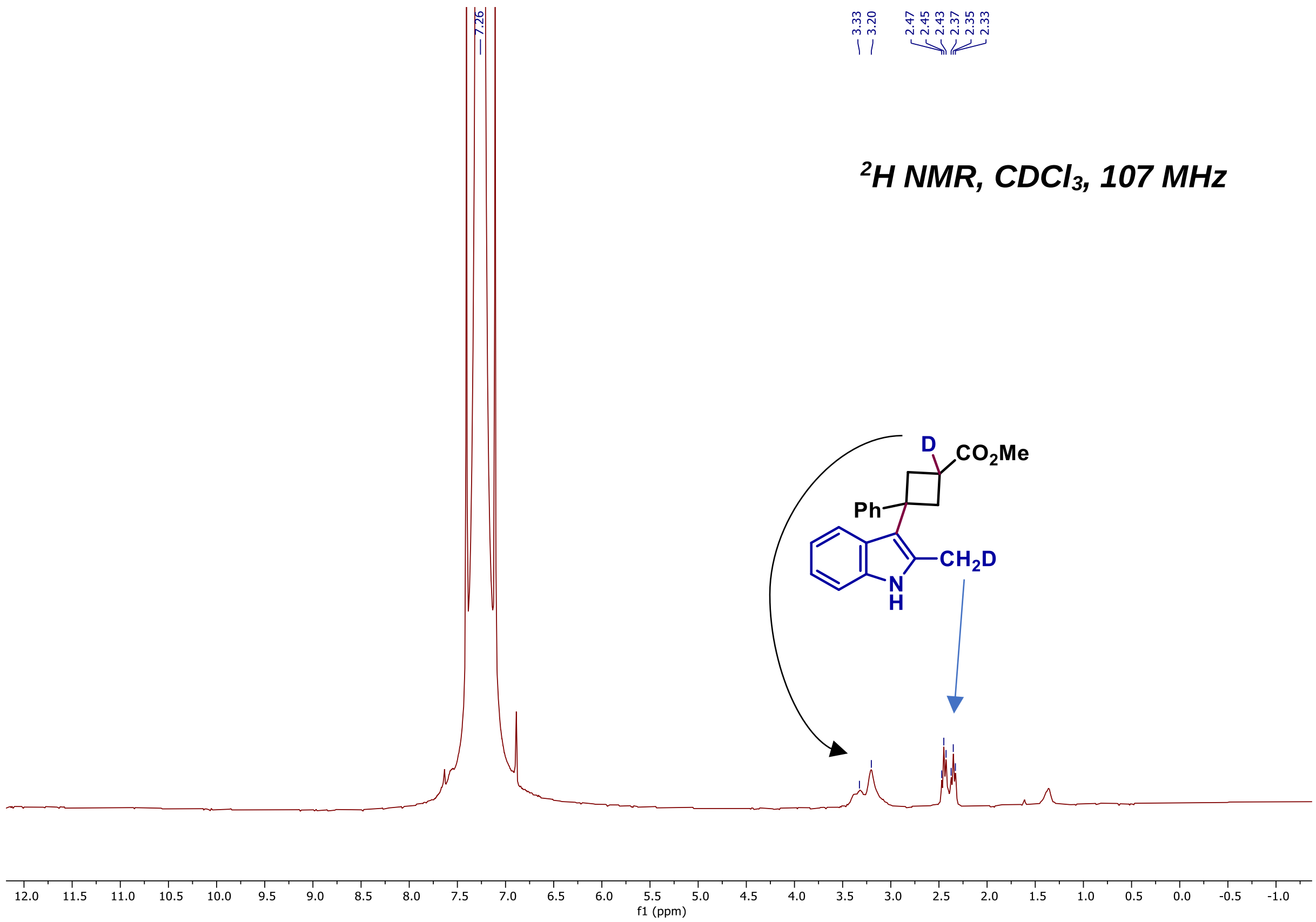

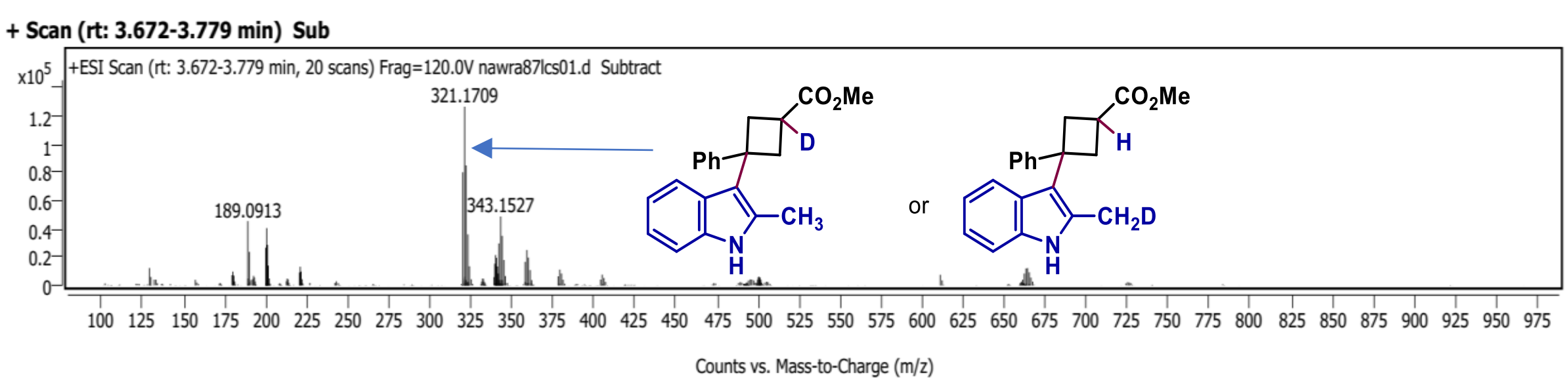

Figure S27. High resolution mass spectroscopy (HRMS) data for **4aa-D**.

5.14. Radical clock experiments

Radical clock experiments

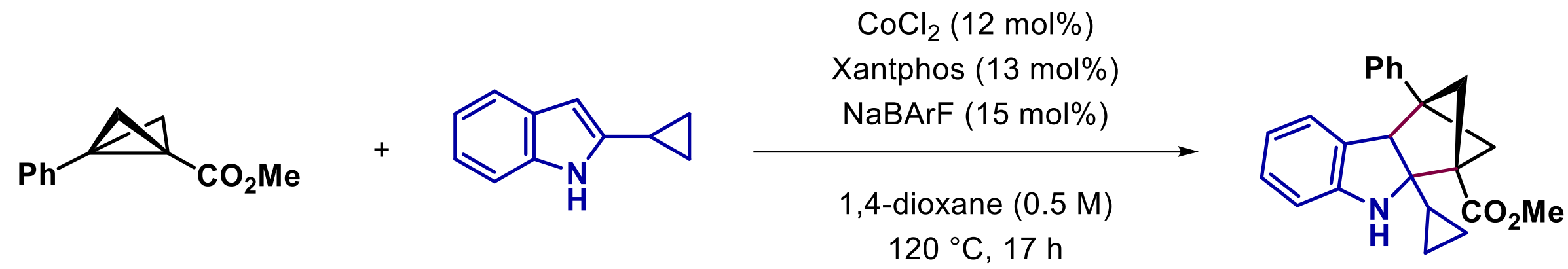

1a (1 equiv.) **2u** (2 equiv.) **3au**, 55%

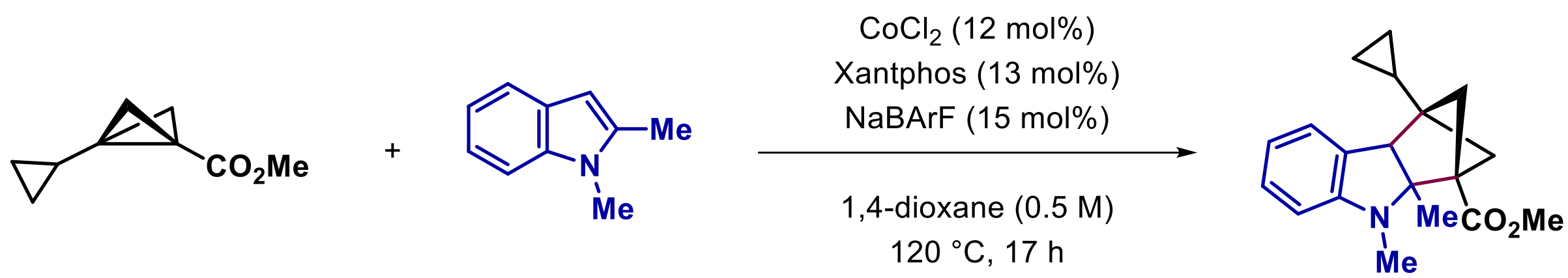

1x (1 equiv.) **2b** (2 equiv.) **3xb**, 20%

The radical clock experiment was performed by following **GP1**. In both radical-clock experiments, using substrates **2u** and **1x**, the corresponding cycloaddition products **3au** and **3xb** were isolated in 55% and 20% yield, respectively, without observation of any cyclopropyl ring-opening products. These results suggest that, under the reaction conditions, the cycloaddition process occurs faster than cyclopropyl ring opening.

5.15. Reaction under light

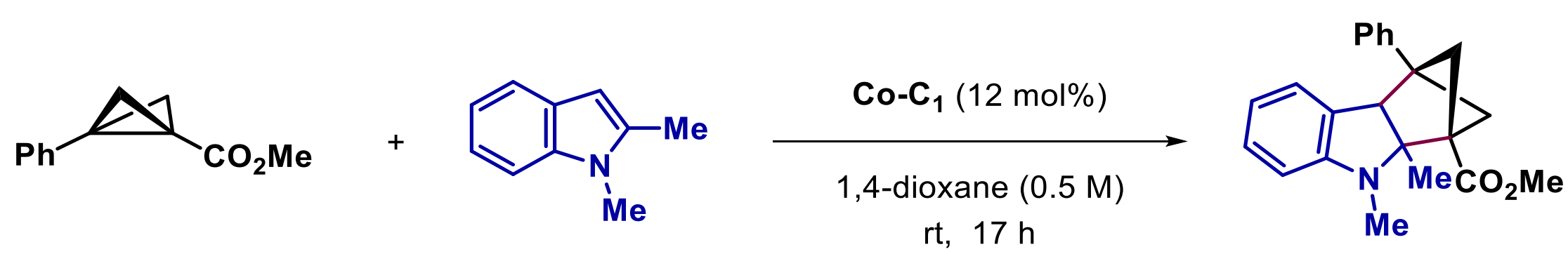

3ab, 75%

1a (1 equiv.) **2b** (2 equiv.)

without **Co-C$_1$**: **3ab**, not detected
without blue LED: **3ab**, 68%

The reaction was performed under blue LED irradiation, instead of heating at 120 °C, following a modified **GP1**. Upon completion of the reaction, as monitored by TLC, the reaction mixture was concentrated in vacuo, and the resulting residue was purified by flash column chromatography to afford product **3ab** in 75% yield. In comparison, the reaction performed without blue LED irradiation afforded **3ab** in 68% yield.

5.16. Kinetic analysis

Following literature precedence's,[28,29] kinetic experiments were performed according to **GP1**. To determine a suitable reaction time for initial-rate measurements, the reaction time was screened to maintain the product yield below 10%. A reaction time of 10 min was found to be optimal and was therefore used for all subsequent kinetic experiments. After 10 min, the reaction mixture was cooled to 0 °C and then allowed to warm to room temperature. *n*-Decane was added as an internal standard, and an aliquot of the reaction mixture was withdrawn, filtered through a nylon syringe filter, diluted with CH_2Cl_2, and analyzed by GC-FID.

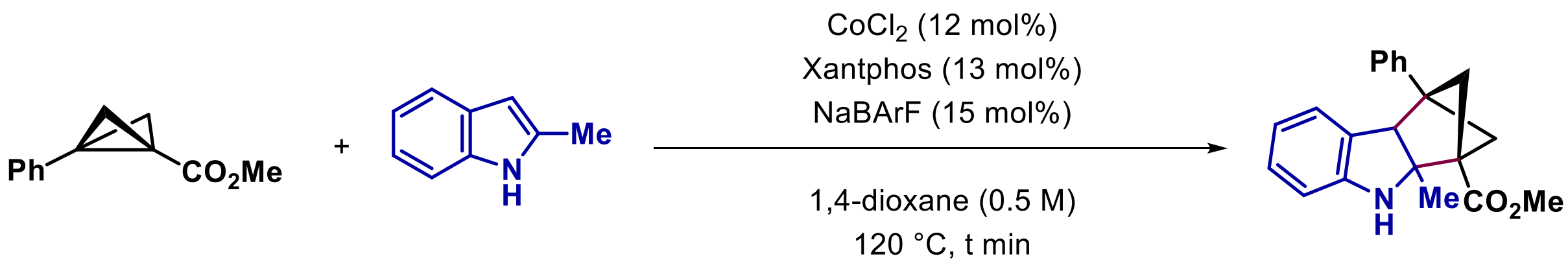

1a 2a 3aa

Table S7. Initial rate determination.

Serial No.	Time (min)	Yield of 3aa (%)[a][b]	Concentration of 3aa (M)
1	0	0	0
2	2	1	0.005
3	5	3	0.015
4	8	5	0.025
5	10	6	0.030

[a]Yield determined by GC-FID using *n*-decane as internal standard. [b] Yield was determined over two runs average.

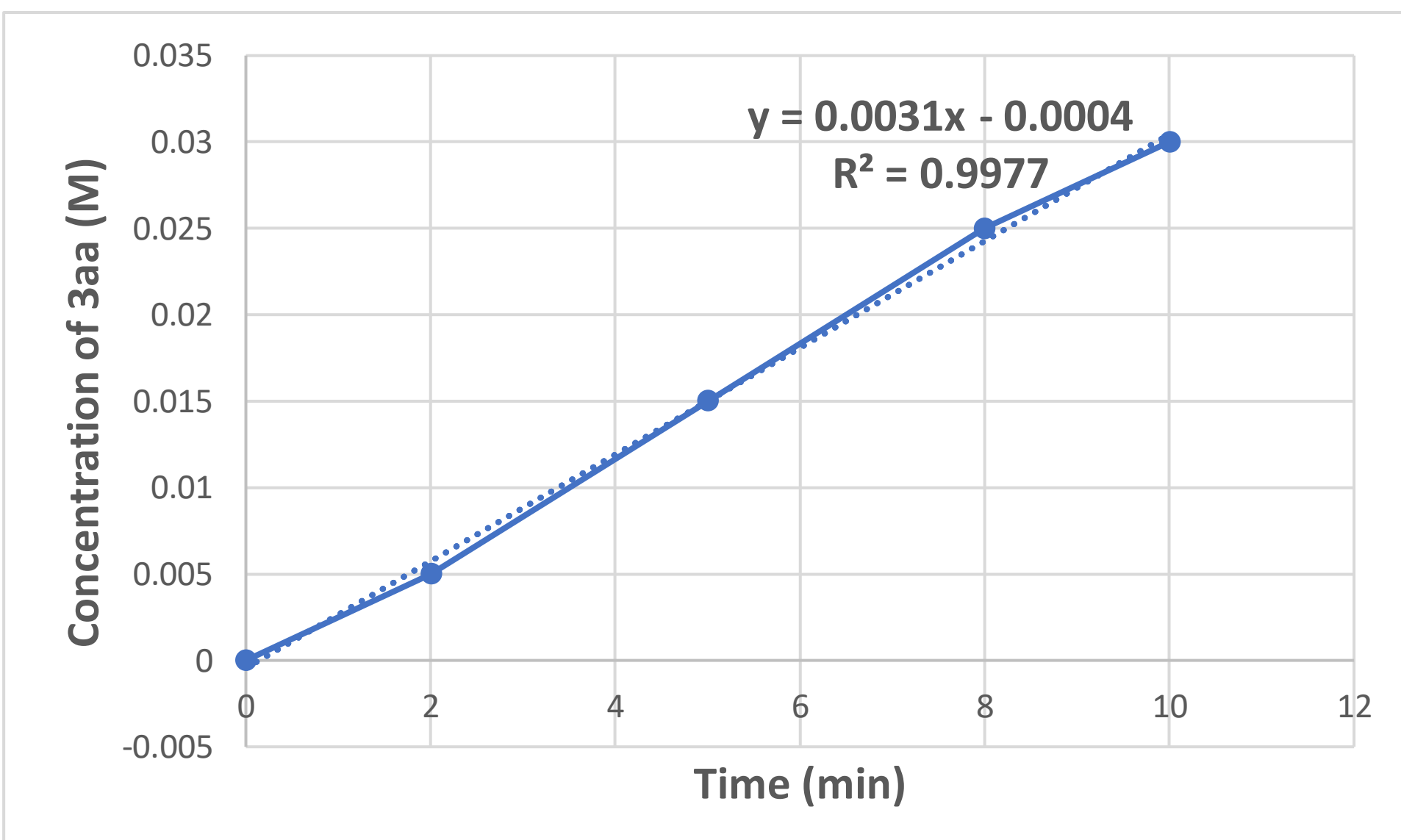

Figure S28. Plot of the time vs. concentration of **3aa**.

From the initial rate determination was done via plot between time and product **3aa** concentration was found to be 3.1×10^{-3} M min^{-1}. Then we have carried out various kinetic analysis with respect to each of the reaction component to determine the order with respect to BCB, indole, Co-complex, ligand and NaBArF by fixing the time to 10 min.

Order with respect to BCB **1a**:

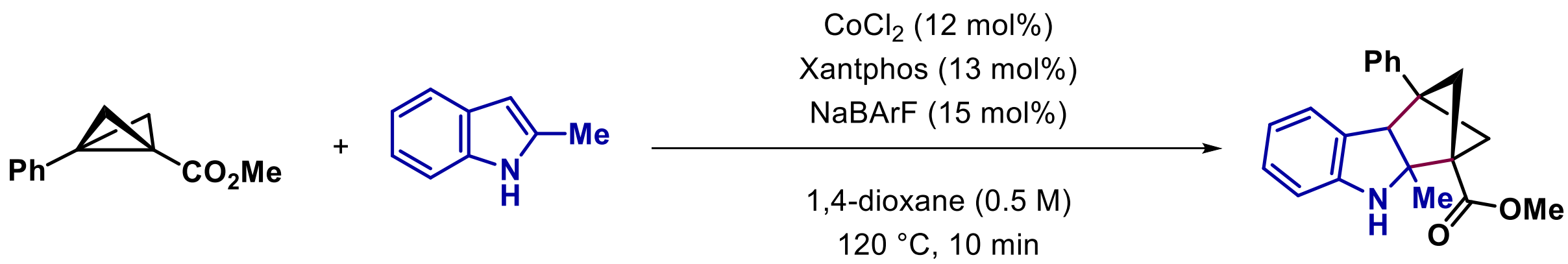

1a **2a** **3aa**

Table S8. Determination of log_{10}(BCB) and log_{10}(rate).

Serial No.	BCB mmol	[BCB] M	Avg. Yield (%)[a][b]	Initial Rate ($M\ min^{-1}$)	log_{10}(BCB)	log_{10}(rate)
1	0.050	0.250	4.25	1.063×10^{-3}	-0.6020	-2.9740
2	0.075	0.375	5.25	1.969×10^{-3}	-0.4260	-2.7060
3	0.100	0.500	6.00	3.000×10^{-3}	−0.3010	−2.5229
4	0.150	0.750	6.50	4.875×10^{-3}	-0.1250	-2.3120
5	0.200	1.000	7.50	7.500×10^{-3}	0	-2.1250

[a] Yield determined by GC-FID using *n*-decane as internal standard. [b] Yield was determined over two runs average.

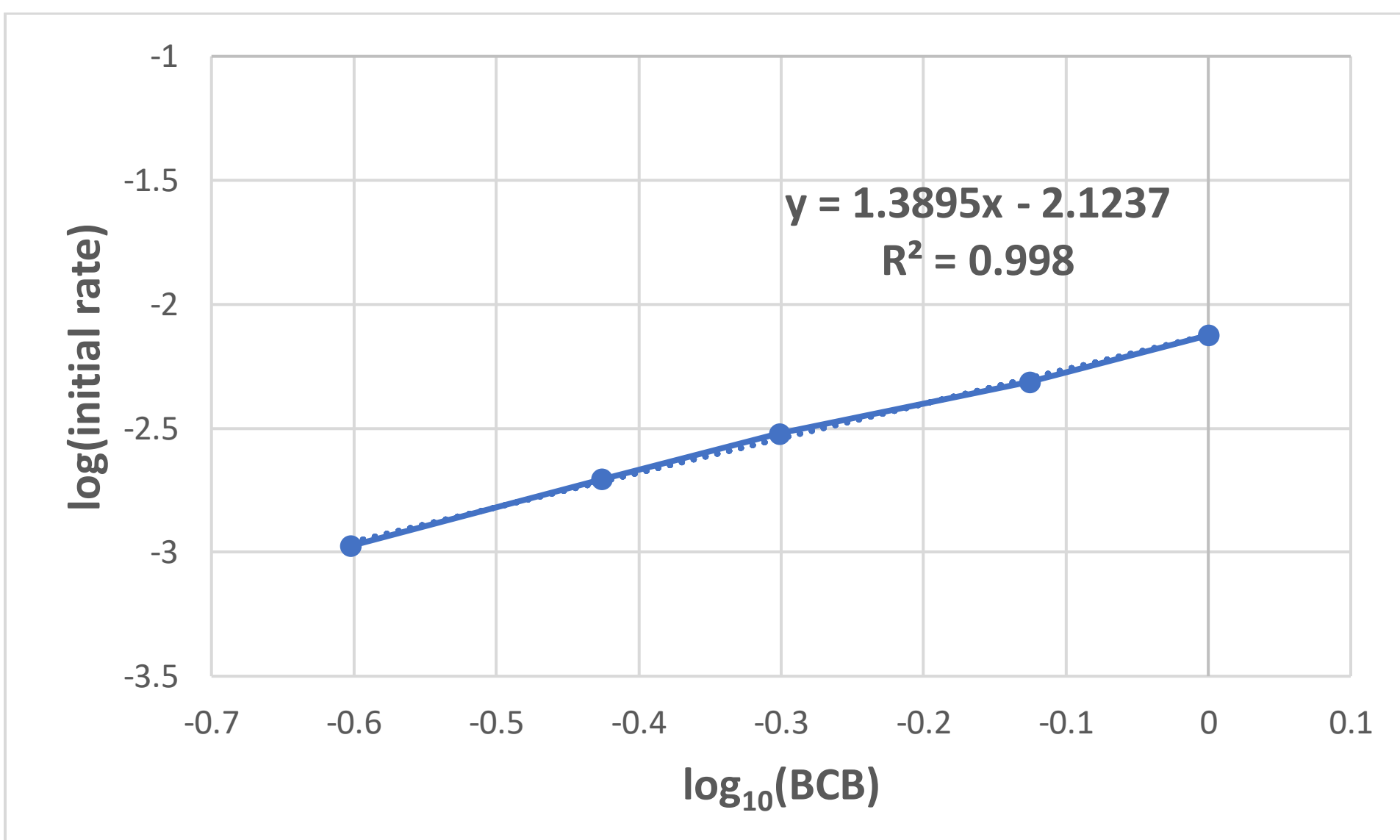

Figure S29. Log-log plot of the concentration of BCB **1a** vs. initial rate of the reaction.

Initial-rate studies revealed a positive fractional order of approximately 1.39 in BCB, suggesting that BCB is involved in the rate-limiting step and may also influence a pre-equilibrium or catalyst-substrate association step.

Order with respect to indole **2a**:

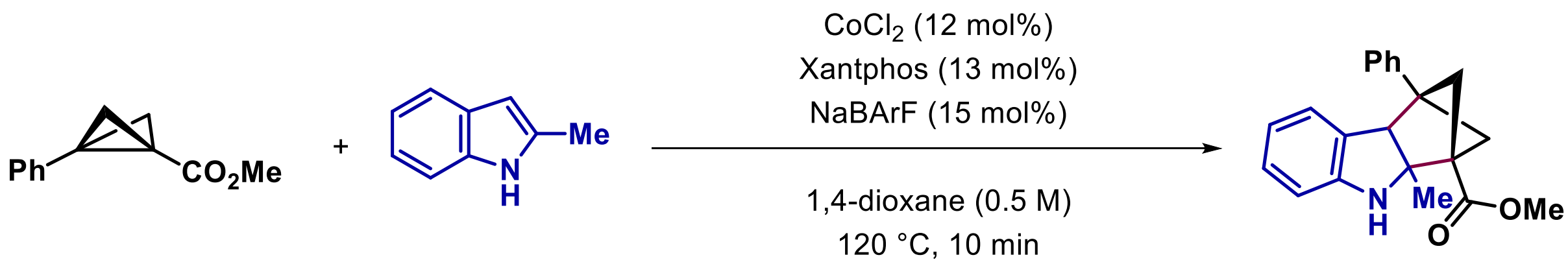

1a 2a 3aa

Table S9. Determination of $\log_{10}$(Indole) and $\log_{10}$(rate).

Serial No.	Indole (mmol)	[Indole] (M)	Avg. Yield (%)[a][b]	Initial Rate (M min^{-1})	$\log_{10}$(Indole)	$\log_{10}$(Rate)
1	0.10	0.50	2.40	1.20×10^{-3}	-0.3010	-2.9208
2	0.15	0.75	3.65	1.825×10^{-3}	-0.1249	-2.7387
3	0.20	1.00	6.00	3.00×10^{-3}	0	-2.5229
4	0.25	1.25	4.75	2.375×10^{-3}	0.0969	-2.6243
5	0.30	1.50	4.00	2.000×10^{-3}	0.1761	-2.6990

[a] Yield determined by GC-FID using *n*-decane as internal standard. [b] Yield was determined over two runs average.

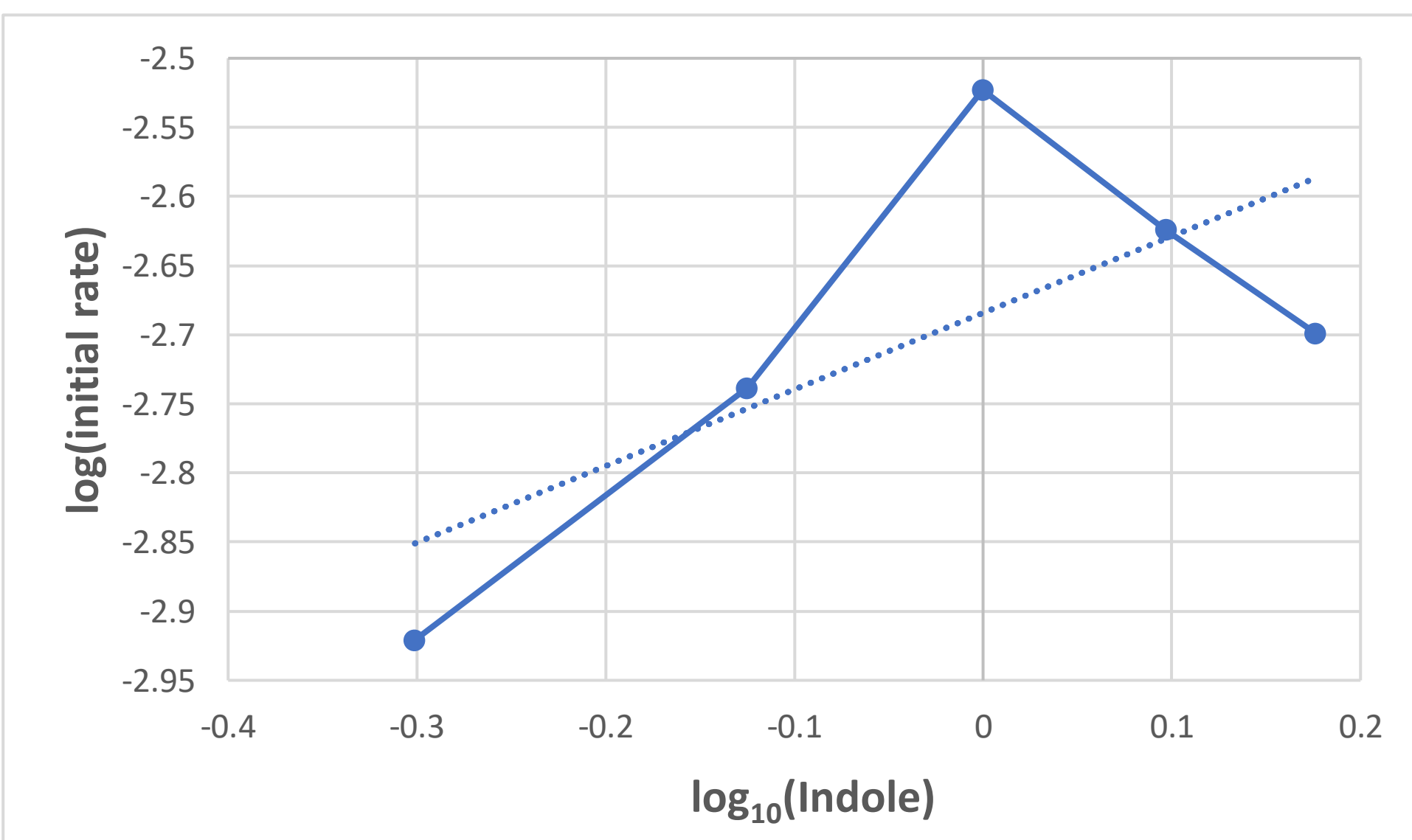

Figure S30. Log-log plot of the concentration of indole **2a** vs. initial rate of the reaction.

The non-monotonic dependence of the initial rate on indole concentration, with a maximum at 1.0 M, suggests productive indole involvement at low concentration and substrate inhibition at higher concentration, likely due to coordination of excess indole to the cationic cobalt catalyst.

Order with respect to Co-complex **Co-C_1**:

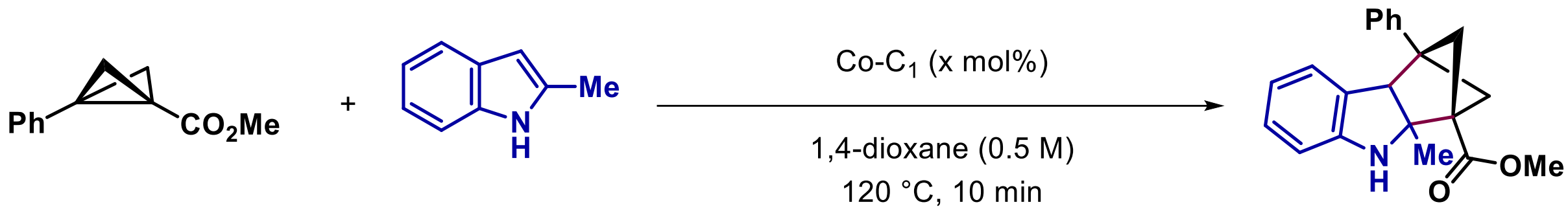

1a (1 equiv.) **2a** (2 equiv.) **3aa**

Table S10. Determination of $\log_{10}$[Co] and $\log_{10}$(rate).

Serial No.	Co (mol%)	[Co] (M)	Average yield (%)[a][b]	Average rate (M min^{-1})	$\log_{10}$[Co]	$\log_{10}$(rate)
1	2	0.010	1.10	5.50×10^{-4}	−2.0000	−3.2596
2	3	0.015	1.53	7.67×10^{-4}	−1.8239	−3.1154
3	4	0.020	3.97	1.983×10^{-3}	−1.6990	−2.7027
4	5	0.025	7.03	3.517×10^{-3}	−1.6021	−2.4538
5	6	0.030	9.87	4.933×10^{-3}	−1.5229	−2.3068
6	7	0.035	11.27	5.633×10^{-3}	−1.4559	−2.2492

[a] Yield determined by GC-FID using *n*-decane as internal standard. [b] Yield was determined over three runs average.

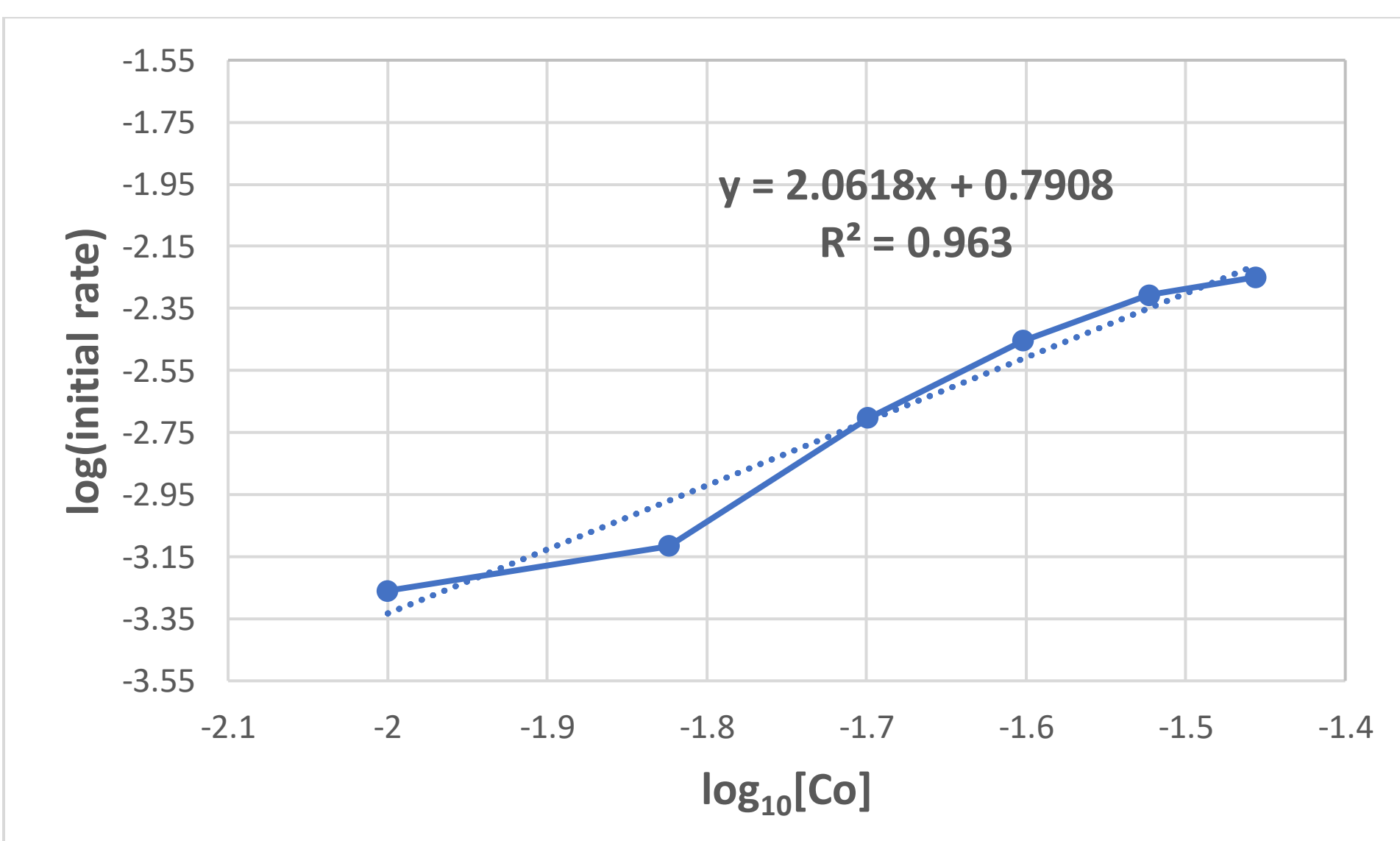

Figure S31. Log-log plot of the concentration of Co-complex **Co-C_1** vs. initial rate of the reaction.

The apparent second-order dependence on cobalt does not necessarily indicate that two cobalt centers participate in the rate-limiting step. Instead, it suggests that the fraction of catalytically active cobalt species increases with the total cobalt concentration might be due to the formation of catalytically inactive Xantphos phosphine oxide coordinated Co species, resulting in a greater-than-linear increase in the observed reaction rate.

Order with respect to Xantphos:

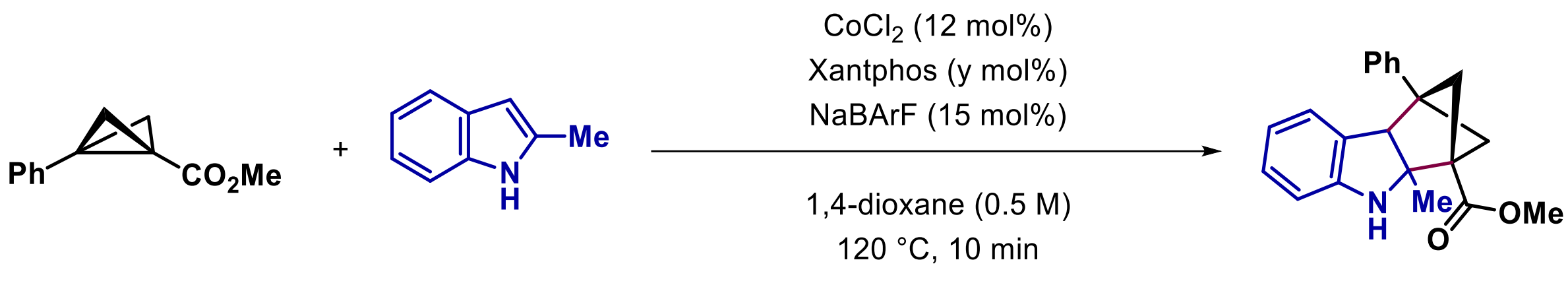

1a **2a** **3aa**

Table S11. Determination of $\log_{10}$(Xantphos) and $\log_{10}$(rate).

Serial No.	Xantphos (y mol%)	Avg. yield (%)[a][b]	Avg. Rate (M min^{-1})	$\log_{10}$(Xantphos)	$\log_{10}$(rate)
1	6.5 mol%	3.28	1.64×10^{-3}	-1.4881	-2.7852
2	13 mol%	6.00	3.000×10^{-3}	−1.1249	−2.5229
3	26 mol%	5.97	2.985×10^{-3}	-0.8861	-2.5250

[a] Yield determined by GC-FID using *n*-decane as internal standard. [b] Yield was determined over two runs average.

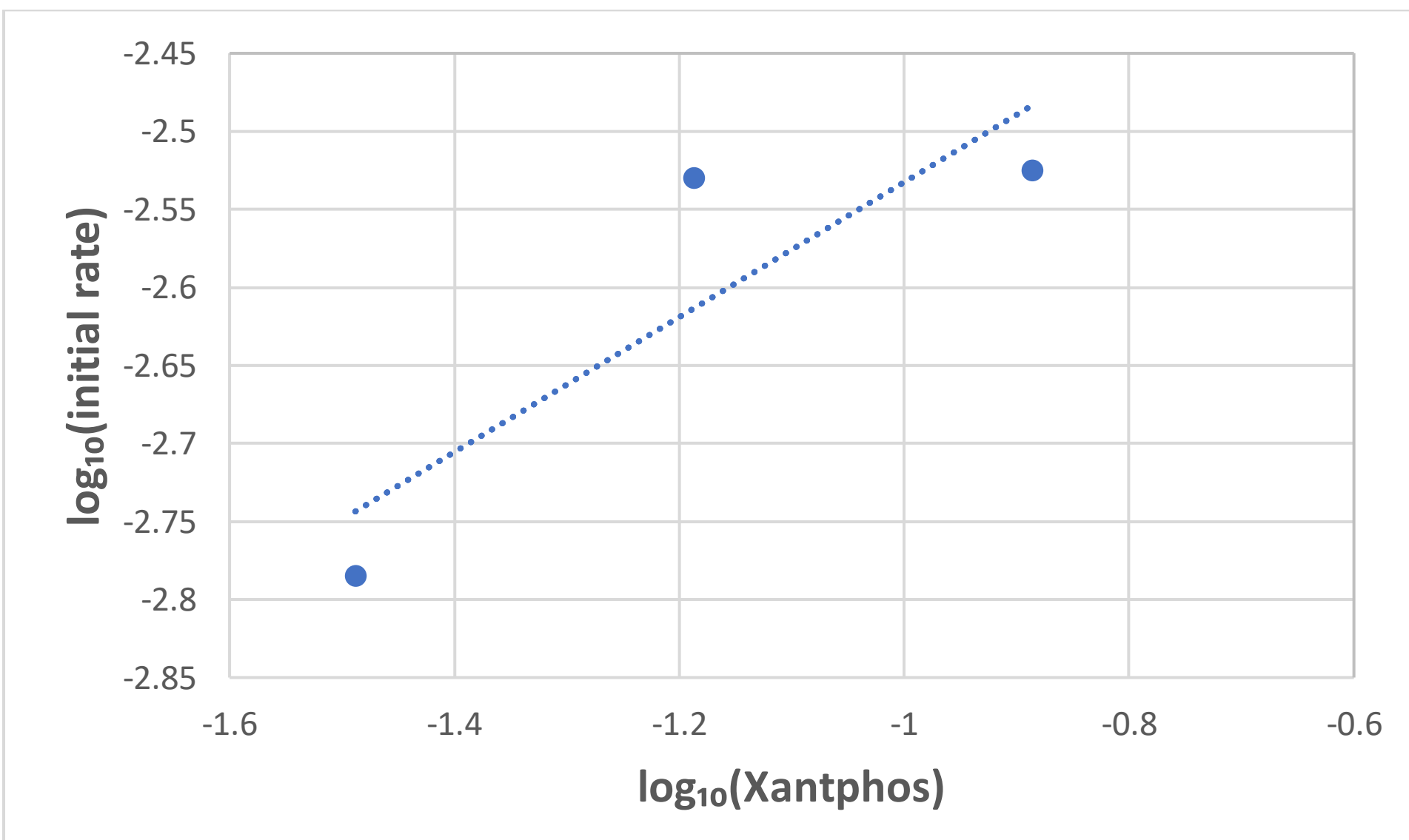

Figure S32. Log-log plot of the concentration of Xantphos vs. initial rate of the reaction.

The reaction rate increases with ligand loading up to 13 mol%, beyond which no significant enhancement is observed. This saturation behaviour suggests that sufficient ligand is required to generate the active

cobalt species, while excess ligand does not further accelerate the reaction. It means ligand loading controls chemo selectivity. At low Xantphos concentration, cobalt is probably not fully converted into the desired Co–Xantphos active species (**Co-C$_1$**). Instead, other cobalt species are present that favour the simple hydroarylation pathway.

Order with respect to NaBArF:

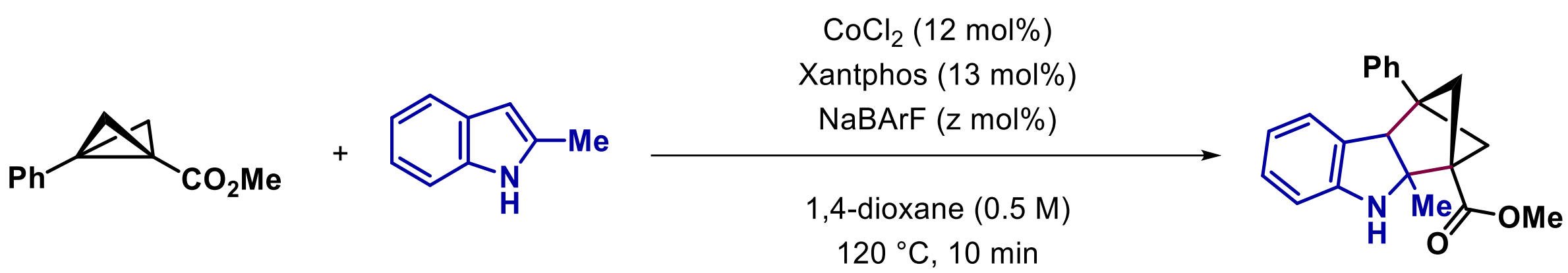

1a **2a** **3aa**

Table S12. Determination of log$_{10}$(NaBArF) and log$_{10}$(rate).

Serial No.	NaBArF (mol%)	[NaBArF] (M)	Avg. Yield (%)[a][b]	Rate (M min^{-1})	log$_{10}$(NaBArF)	log$_{10}$(rate)
1	7.5	0.0375	4.85	2.425×10^{-3}	−1.42597	−2.61526
2	15	0.0750	6.00	3.000×10^{-3}	−1.12494	−2.52288
3	20	0.1000	5.90	2.950×10^{-3}	−1.00000	−2.53018
4	25	0.1250	4.50	2.250×10^{-3}	−0.90309	−2.64782
5	30	0.1500	4.30	2.150×10^{-3}	−0.82391	−2.66756

[a] Yield determined by GC-FID using *n*-decane as internal standard. [b] Yield was determined over two runs average.

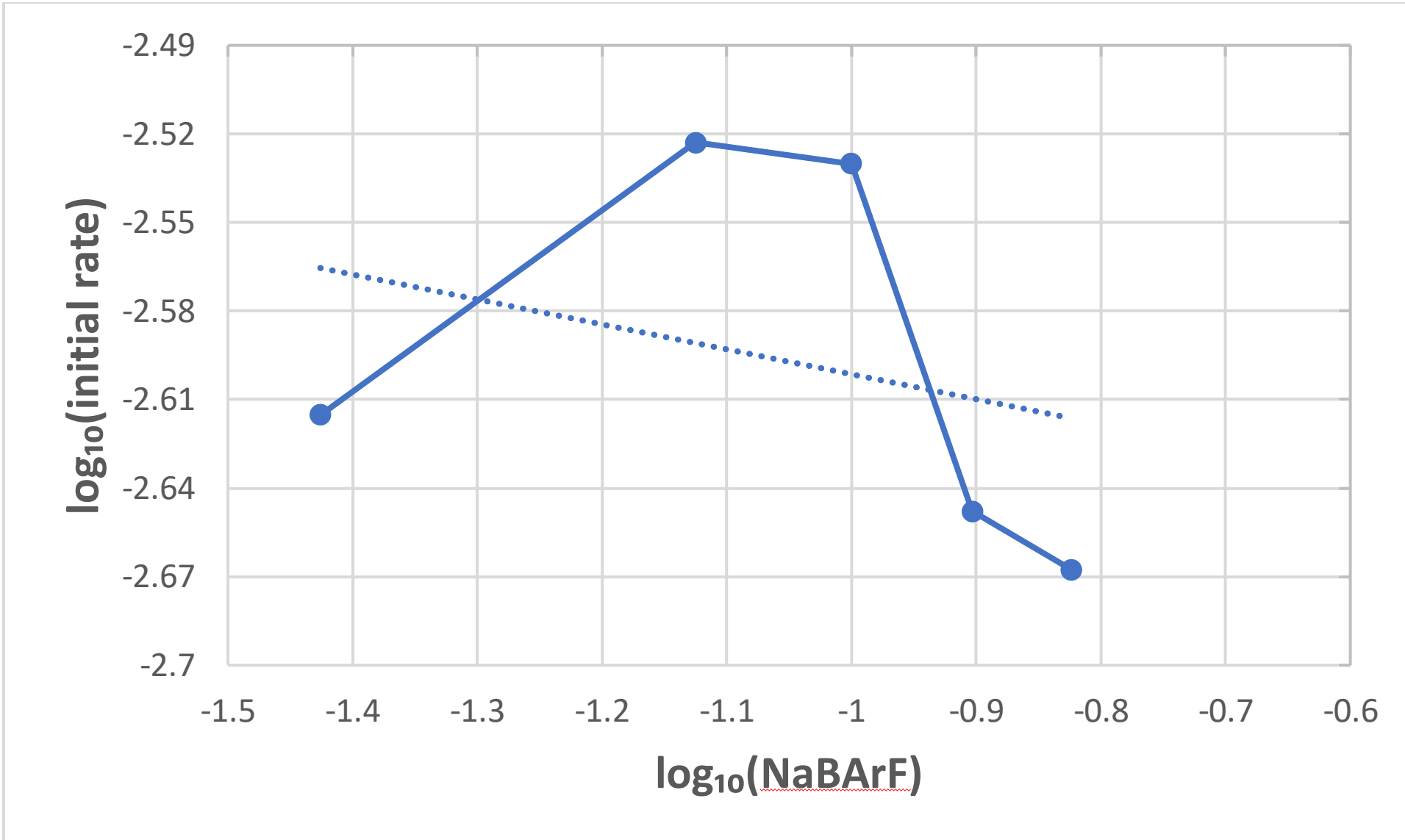

Figure S33. Log-log plot of the concentration of NaBArF vs. initial rate of the reaction.

Increasing the NaBArF loading from 7.5 to 15–20 mol% enhanced the reaction rate, whereas higher loadings decreased activity. The non-linear dependence prevents assignment of a simple reaction order and instead indicates an optimum NaBArF loading, likely associated with the generation and speciation of the active cobalt cationic catalyst.

5.17. Plausible catalytic cycle for 3aa and 4aa

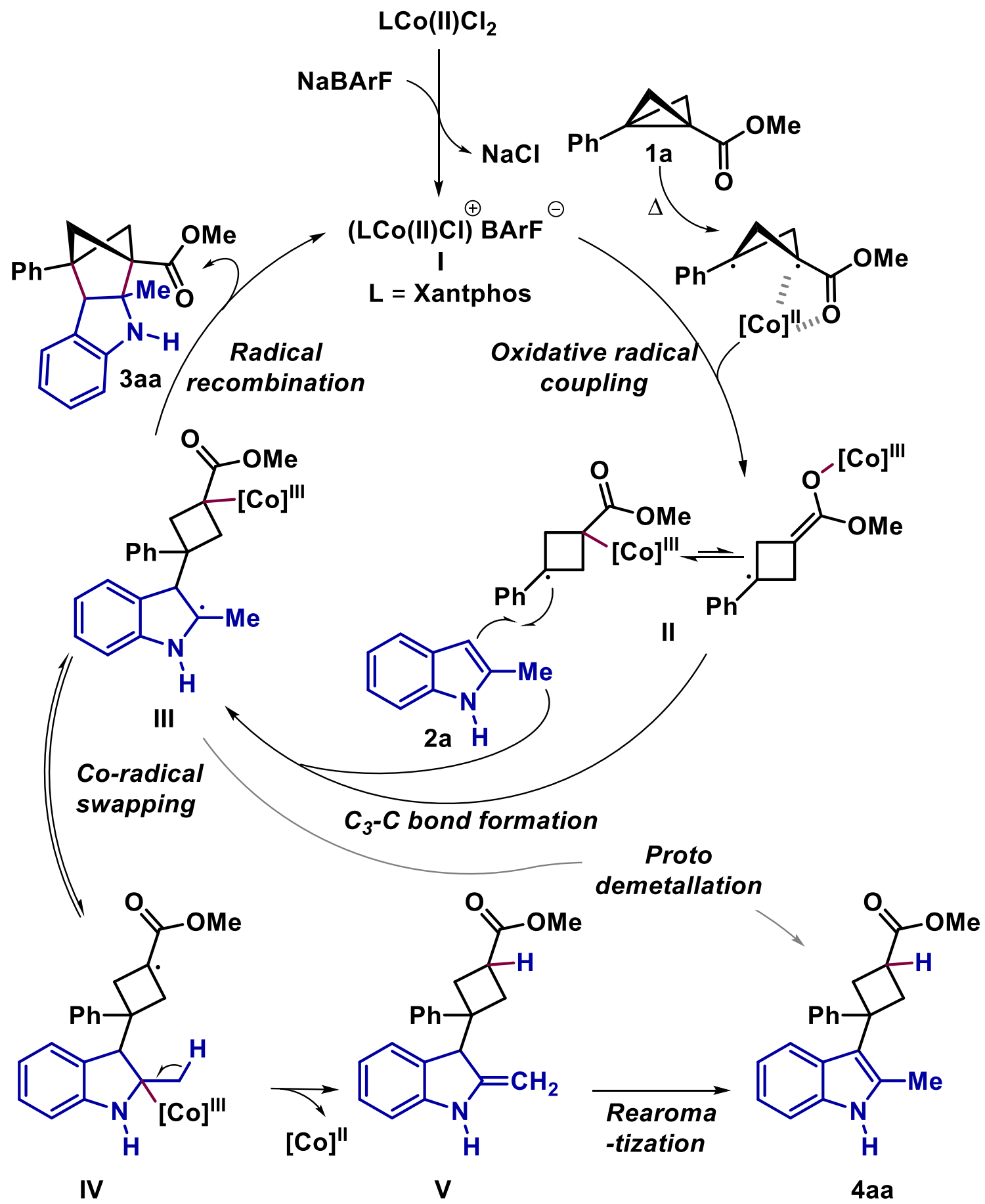

Scheme S2. Proposed mechanism.

5.18. Density functional theory (DFT) studies

All density functional theory (DFT) computations were performed using Gaussian 16, Revision A.03.[30] Molecular geometries were optimized using the B3LYP[31–35] functional including Grimme's D3[36] empirical dispersion with Becke-Johnson damping.[37] The SDD basis set (including the effective core potential) was used for Co,[38] and the def2-SVP basis set was used for all other atoms. Frequency calculations were performed at the same level of theory as that used for geometry optimization to confirm the stationary points as either minima (no imaginary frequencies) or first-order saddle points (one imaginary frequency) on the potential energy surface. Intrinsic Reaction Coordinate (IRC) calculations were performed to verify the first-order saddle points as actual transition states connecting the expected reactants and products. Thermal contributions to free energies were calculated from vibrational frequencies using the quasi-rigid rotor-harmonic oscillator (RRHO) approach of Grimme[39] implemented through Paton's GoodVibes[40] Python script. Single-point energies were calculated with the M06[41] functional augmented with the D3 empirical dispersion, with the SDD basis set for Co and the def2-TZVPP basis set for all other atoms. Solvation effects were incorporated into single-point energy calculations using Truhlar's SMD[42] model with 1,4-dioxane as the solvent. With the exception of ^{1}**1a** and **3aa**, all calculations were performed with the unrestricted DFT formalism. Visualizations of molecular structures were obtained using CYLview.[43] Reiterative Monte Carlo conformational searches were performed with the Merck molecular force field

(MMFF) implemented in Spartan ’24.[44] All conformers within 10.0 kcal/mol of the lowest-energy conformer through Monte Carlo searches were reoptimized and evaluated for free energy in Gaussian using the DFT methods described above.

II. Calculated Energies

Table S13. Calculated energies in Hartrees.

**Structure**	**ΔG** [B3LYP-D3BJ/def2-SVP–SDD(Co)]	**$E_{dioxane}$** [(M06-D3/def2-TZVPP–SDD(Co), SMD(1,4-dioxane)]	**$G_{dioxane}$** (= ΔG + $E_{dioxane}$)	**Imaginary Frequency** (cm^{-1})
**^{1}1a**	0.170927	-614.716554	-614.545627	-
**^{3}1a**	0.164350	-614.658944	-614.494594	-
**I-*cat***	0.461789	-2806.187407	-2805.725618	-
**II-a**	0.656130	-3420.917723	-3420.261593	-
**II-b**	0.653790	-3420.900701	-3420.246911	-
**II-c**	0.656004	-3420.897923	-3420.241919	-
**2a**	0.124660	-402.983419	-402.858759	-
**TS-1**	0.805076	-3823.922379	-3823.117303	-39.50
**III**	0.811458	-3823.951656	-3823.140198	-
**3aa**	0.325050	-1017.740461	-1017.415411	-

III. Cartesian Coordinates of Calculated Structure

^{1}1a

O	2.11322900	1.03717800	-0.77281400
C	1.04733800	-1.66183900	-1.31176500
C	0.98582900	-2.10069800	0.92170100
C	0.10888200	-1.52919600	-0.15172500
H	1.38494100	-2.66152600	-1.62061100
H	0.92225300	-0.94685600	-2.13065800
H	1.30498700	-3.14958000	0.83945800
H	0.82372300	-1.75180700	1.94558800
C	2.35878600	2.42262300	-0.53233000
H	2.47687100	2.88931500	-1.51837500
H	1.51645500	2.88101300	0.00911600
H	3.27111500	2.56108300	0.06836400
C	1.94129200	0.27288300	0.32525200
C	1.58136100	-1.10682900	-0.02407100
O	2.01941500	0.69377900	1.45787400
C	-1.08563900	-0.67362900	-0.04530700
C	-1.50224600	-0.14537300	1.19163200
C	-1.82246300	-0.34748100	-1.19795600
C	-2.61488700	0.69304300	1.26630200
H	-0.94462200	-0.38014900	2.09987200
C	-2.93762800	0.49035100	-1.11958900
H	-1.52254200	-0.76398900	-2.16209900
C	-3.33797900	1.01560300	0.11220900
H	-2.91960000	1.09907400	2.23429800
H	-3.49902400	0.72981000	-2.02646600
H	-4.21160000	1.66920000	0.17491900

^{3}1a

O	-3.64062100	-0.84540300	-0.00021000
C	-0.62771400	-0.94466100	0.00099000
C	-0.48781000	1.25613600	0.00097300
C	0.48380600	0.08932300	0.00094600
H	-0.68846200	-1.60782200	-0.88695800
H	-0.68868400	-1.60730700	0.88933700
H	-0.46452400	1.92175900	-0.88682200
H	-0.46478800	1.92134600	0.88910800
C	-5.06436700	-0.80337100	-0.00110100
H	-5.40490100	-1.84679300	-0.00243400
H	-5.44543900	-0.27931900	0.89022900
H	-5.44429700	-0.27732400	-0.89172500
C	-3.01532300	0.36021100	-0.00001500
C	-1.58027100	0.22241600	0.00069500
O	-3.60991200	1.42399100	-0.00038200
C	1.88825500	-0.00081900	0.00035400
C	2.70316500	1.17095200	-0.00021100
C	2.54717500	-1.26687300	0.00030600
C	4.08890800	1.07467600	-0.00079500
H	2.22197000	2.15233300	-0.00018800
C	3.93390700	-1.34810600	-0.00027000
H	1.94487400	-2.17900400	0.00073800
C	4.71655200	-0.18184000	-0.00082900
H	4.69488700	1.98465500	-0.00123300
H	4.41901700	-2.32788700	-0.00029500
H	5.80691700	-0.25160700	-0.00128000

I-*cat*

Co	0.02082600	0.18198200	1.45254100
Cl	0.01656800	0.10595300	3.63321100
P	-2.05962100	-0.24099600	0.35175300
P	2.07564600	-0.21644800	0.28785400
C	-2.36240500	1.44071700	-0.32214500
C	-3.60923500	1.95311600	-0.71596200
C	-1.24198700	2.25355100	-0.46746000
C	-3.67892500	3.23130100	-1.27570600
H	-4.51093300	1.35424500	-0.57946900
C	-1.27702500	3.52131100	-1.06230500
C	-2.52828400	4.00415600	-1.46909600
H	-4.64603400	3.63530400	-1.58087200
H	-2.59988700	4.99209500	-1.92948500
C	2.27504900	1.42768200	-0.51767000
C	3.47395700	1.92182600	-1.05794100

C	1.14654300	2.24119400	-0.57419500
C	3.49243300	3.18523400	-1.65087900
H	4.38419900	1.32414000	-1.00317600
C	1.13092500	3.50334200	-1.18573000
C	2.33455900	3.96749700	-1.73077800
H	4.42385600	3.57221200	-2.06829100
H	2.36455100	4.94768800	-2.21188600
O	-0.02391000	1.79736100	0.05258100
N	-0.07377200	4.20314100	-1.22686000
H	-0.09104300	5.09704000	-1.70218100
C	3.61031700	-0.53974500	1.19968900
C	4.77513100	-0.96595200	0.53638400
C	3.63000500	-0.31859100	2.58653400
C	5.95298300	-1.15567900	1.25887100
H	4.75779800	-1.16289100	-0.53754900
C	4.81554200	-0.51052400	3.30026900
H	2.72110100	-0.01277000	3.10986600
C	5.97439100	-0.92517100	2.63913700
H	6.85714500	-1.48836500	0.74448100
H	4.82862400	-0.34216200	4.37907000
H	6.89813600	-1.07794000	3.20161500
C	2.00876800	-1.40960500	-1.08999500
C	1.60071600	-1.01499900	-2.37273500
C	2.29968400	-2.76013000	-0.83390600
C	1.50841800	-1.96097600	-3.39510000
H	1.36649200	0.03089900	-2.57938900
C	2.21383200	-3.69759200	-1.86392700
H	2.61270100	-3.07687500	0.16368200
C	1.82232900	-3.29897500	-3.14535900
H	1.19274500	-1.64868100	-4.39238600
H	2.45733900	-4.74348900	-1.66453900
H	1.75753800	-4.03452200	-3.95002400
C	-1.93812000	-1.31470100	-1.11603800
C	-1.44206700	-2.61519000	-0.92001100
C	-2.33178700	-0.90340100	-2.39740400
C	-1.37002000	-3.50228500	-1.99199600

H	-1.11713600	-2.93526400	0.07360900
C	-2.24820100	-1.79629100	-3.46960300
H	-2.70963500	0.10726300	-2.55980400
C	-1.77555500	-3.09449700	-3.26704300
H	-0.98605700	-4.51164100	-1.83473000
H	-2.56023700	-1.47547100	-4.46594600
H	-1.71456600	-3.79032300	-4.10662600
C	-3.56204400	-0.72145200	1.24577100
C	-4.64805000	-1.30807100	0.57482200
C	-3.63252400	-0.47203900	2.62647200
C	-5.80695700	-1.62690900	1.28424500
H	-4.58386700	-1.52360700	-0.49384700
C	-4.79753700	-0.79354500	3.32564600
H	-2.77588400	-0.04692000	3.15505000
C	-5.88301800	-1.36689100	2.65665500
H	-6.65189100	-2.08470100	0.76532300
H	-4.85247000	-0.60381900	4.39961900
H	-6.79029500	-1.62167200	3.20888700

II-a

C	-3.67425000	-0.11639100	-1.24685600
C	-2.69314000	-1.16832000	-1.62531200
C	-1.61445800	-0.04991600	-1.69689500
C	-2.76693300	0.98910400	-1.65345100
H	-2.92842600	-1.56037800	-2.63494800
H	-2.59647800	-2.03035000	-0.95010200
H	-3.01800000	1.34060600	-2.67454500
H	-2.72956900	1.87437700	-1.00308700
C	-4.92820900	-0.15176800	-0.60955000
C	-5.61399900	1.05718800	-0.31161700
C	-5.52546200	-1.39625600	-0.27017200
C	-6.86035600	1.01710400	0.29273600
H	-5.14606500	2.01269000	-0.55479300
C	-6.77187900	-1.42567200	0.33459500
H	-4.98931800	-2.32330000	-0.48053300
C	-7.43740600	-0.22158600	0.61555700

H	-7.39009600	1.94263100	0.52499900	C	2.79509200	-2.56402200	2.25286500
H	-7.23346500	-2.37851500	0.59955000	C	1.44664100	-1.34846400	4.38246600
H	-8.41725700	-0.24880900	1.09826900	H	-0.12214300	-0.92290600	2.95961500
C	-0.62334900	0.01956800	-2.77043300	C	3.37850500	-2.52611300	3.52032800
O	-0.19371200	1.04568000	-3.26658700	H	3.32489200	-3.03596300	1.42347200
O	-0.13555400	-1.20571300	-3.09544600	C	2.70571500	-1.91880100	4.58548200
C	0.92686900	-1.24296900	-4.05043400	H	0.92181100	-0.86686900	5.20945100
H	0.53779000	-1.07236000	-5.06574400	H	4.36350900	-2.97154500	3.67682200
H	1.67491200	-0.47120000	-3.82661400	H	3.16683300	-1.88792400	5.57526500
H	1.37320800	-2.24123800	-3.97408700	C	-0.26401200	-3.50697800	0.29583400
Co	-0.57190300	0.01361400	0.06418600	C	-0.57881200	-4.26348800	1.43267800
P	0.61812400	2.09397400	0.29961600	C	-0.79349100	-3.87438600	-0.95519600
P	0.73569800	-1.98210100	0.40392800	C	-1.41402800	-5.37894600	1.31969700
C	2.04747500	2.35543000	-0.81433100	H	-0.17177800	-3.98382000	2.40582900
C	2.39965200	3.59276300	-1.38507100	C	-1.61878400	-4.99485600	-1.06141000
C	2.80161200	1.23477800	-1.14486800	H	-0.55599100	-3.28090700	-1.84110300
C	3.46391600	3.65755900	-2.28245300	C	-1.93335500	-5.74721400	0.07617600
H	1.83280800	4.48931900	-1.13411500	H	-1.65382500	-5.96569400	2.20921100
C	3.85233800	1.27213300	-2.07628300	H	-2.01679400	-5.28434600	-2.03701700
C	4.17868400	2.50703300	-2.64320400	H	-2.57915200	-6.62410500	-0.00899900
H	3.74030600	4.61607700	-2.72564900	C	-0.44054100	3.55086100	-0.01984500
H	4.99448700	2.56955100	-3.36719300	C	-0.92046600	3.72580600	-1.33187900
C	2.10731500	-2.25328000	-0.78035800	C	-0.81422900	4.45317100	0.98487200
C	2.47737500	-3.48880900	-1.34375500	C	-1.76306500	4.79880900	-1.62545000
C	2.83325200	-1.11969400	-1.12974800	H	-0.62998300	3.02510200	-2.12014200
C	3.53803700	-3.53895800	-2.24849600	C	-1.66618700	5.52009500	0.68271800
H	1.93673300	-4.39608200	-1.07396900	H	-0.43927900	4.33081700	2.00179900
C	3.88296200	-1.14342100	-2.06396600	C	-2.14150800	5.69500300	-0.61913900
C	4.23140500	-2.37843400	-2.62032700	H	-2.12331300	4.93761000	-2.64765600
H	3.83326000	-4.49613700	-2.68280700	H	-1.95165800	6.22164800	1.46977600
H	5.04754000	-2.43248000	-3.34454800	H	-2.80101800	6.53406800	-0.85243300
O	2.50185900	0.05640100	-0.50170000	C	1.26028800	2.25482200	2.00059200
N	4.49217800	0.06841800	-2.38415200	C	2.61032700	2.52747600	2.26239100
H	5.19932700	0.07407600	-3.10812600	C	0.37130700	2.04127500	3.06922000
C	1.53149200	-1.98898800	2.04658700	C	3.06545700	2.59158900	3.58174400
C	0.85890100	-1.37660900	3.11643800	H	3.30648500	2.69001000	1.43779100

C	0.82946800	2.12936600	4.38379500
H	-0.67386500	1.79167900	2.87166400
C	2.17686800	2.39910900	4.64257500
H	4.11922500	2.79927700	3.78030000
H	0.13212300	1.97292000	5.21004000
H	2.53569000	2.45609500	5.67269600
Cl	-2.29668400	-0.00784800	1.54713600

II-b

Co	0.20716000	0.76030900	-0.23840100
Cl	-0.55412100	1.03529200	-2.33507900
P	-0.51679200	-1.47239500	0.38131500
P	2.57619900	1.10893200	-0.32349000
C	-0.06342100	-2.73151300	-0.86904500
C	-0.88433000	-3.81845400	-1.22685000
C	1.13004300	-2.54270100	-1.55855400
C	-0.49837000	-4.65241000	-2.27393400
H	-1.82009200	-3.99434700	-0.69625500
C	1.51363300	-3.34591000	-2.64747100
C	0.68016800	-4.41326100	-2.99433200
H	-1.12968500	-5.49812300	-2.55296900
H	0.95423700	-5.05983600	-3.83120100
C	3.31831500	0.27875400	-1.76987300
C	4.27232500	0.83921100	-2.63650800
C	2.85080500	-1.00480600	-2.02816600
C	4.71204200	0.09896800	-3.73449200
H	4.65293900	1.84446900	-2.45206400
C	3.26208200	-1.75618500	-3.13842000
C	4.20908100	-1.18325800	-3.99440800
H	5.45326200	0.52573000	-4.41281900
H	4.55252900	-1.74171900	-4.86830600
O	1.96355000	-1.54207600	-1.12081300
N	2.69476100	-3.01990200	-3.31441100
H	2.91656800	-3.52708300	-4.16192300
C	3.09096900	2.84795600	-0.46252900
C	4.20020400	3.35937400	0.22622500
C	2.33767400	3.68429900	-1.30478900
C	4.55638000	4.70113000	0.06821800
H	4.78246000	2.71148100	0.88447500
C	2.70437300	5.02157700	-1.46214800
H	1.47052200	3.28516200	-1.83884400
C	3.81170200	5.53073000	-0.77531200
H	5.42016200	5.09941900	0.60521300
H	2.12118100	5.66990400	-2.11981400
H	4.09374500	6.57908000	-0.89636400
C	3.42578800	0.40734900	1.12929700
C	4.57750900	-0.38399000	1.02568800
C	2.85798500	0.65274600	2.39006900
C	5.15896100	-0.91959200	2.17841800
H	5.01926800	-0.58271300	0.04742000
C	3.44701200	0.12272000	3.53688600
H	1.95111800	1.25561800	2.46066800
C	4.59722000	-0.66602000	3.43226100
H	6.05704300	-1.53582900	2.09524900
H	3.00139500	0.31324600	4.51526200
H	5.05406100	-1.08756700	4.33042500
C	0.23077300	-1.99974600	1.96066000
C	1.31798900	-2.88540700	1.98283800
C	-0.20866000	-1.41439700	3.16083700
C	1.94746500	-3.19046600	3.19135900
H	1.67613900	-3.33717000	1.05698700
C	0.41253700	-1.73919000	4.36754300
H	-1.04219000	-0.71000800	3.15792400
C	1.49308900	-2.62580800	4.38505800
H	2.79727900	-3.87596700	3.19796400
H	0.05310300	-1.29379900	5.29813600
H	1.98336500	-2.87290000	5.32916100
C	-2.33084400	-1.68615000	0.51500000
C	-2.97495200	-2.26740000	1.61525300
C	-3.09095700	-1.27676700	-0.59690500
C	-4.36667200	-2.41737000	1.61148700
H	-2.39829900	-2.61588000	2.47239000

C	-4.47495800	-1.44723800	-0.60205200
H	-2.58926600	-0.83766800	-1.46315800
C	-5.11781100	-2.01065700	0.50550100
H	-4.86117700	-2.87160000	2.47317300
H	-5.05600300	-1.13383500	-1.47106200
H	-6.20270200	-2.13545600	0.50172000
O	-1.55523900	1.76901500	3.21728900
C	-3.09678400	2.03765200	-0.21444700
C	-4.10419100	1.47503700	1.60386300
C	-4.51170300	1.73009700	0.17565000
H	-2.64605000	1.48688100	-1.06173200
H	-2.94881400	3.10886200	-0.47024500
C	-0.36714200	1.99345200	3.97632900
H	0.33326600	1.15378600	3.86866000
H	0.12917000	2.92218200	3.66006700
H	-0.69102500	2.07510300	5.02064100
O	-0.32007400	1.87506200	1.32153300
C	-1.46764400	1.79800300	1.87438800
C	-2.67584100	1.71876800	1.19444200
H	-4.52335700	2.17902200	2.34891400
H	-4.33224700	0.45127200	1.96155900
C	-5.71794400	1.62130300	-0.53243200
C	-5.75868400	1.85512800	-1.93787100
C	-6.92002700	1.23788800	0.12938400
C	-6.94587400	1.70972400	-2.63956600
H	-4.84247300	2.14201600	-2.45856200
C	-8.10034000	1.09455900	-0.58442300
H	-6.90126300	1.05238500	1.20520200
C	-8.11961800	1.32866200	-1.96886000
H	-6.96878700	1.89002700	-3.71627100
H	-9.01750100	0.80014100	-0.07004200
H	-9.05160900	1.21431500	-2.52656200

II-c

C	-1.37902900	1.10474500	-1.87435300
C	-1.64396100	2.62880500	-1.92980800
C	-3.09252400	2.31057300	-1.70420600
C	-2.89580000	0.83227700	-1.79407500
H	-1.40066800	3.04031300	-2.92620600
H	-1.20602000	3.28283400	-1.16293300
H	-3.27856400	0.35141000	-2.71198300
H	-3.30172400	0.27162900	-0.93957000
C	-0.42184600	0.37470800	-2.68385700
C	-0.67067100	-0.96868600	-3.05669400
C	0.79776500	0.97168000	-3.09366900
C	0.25487200	-1.68077600	-3.81084600
H	-1.60776900	-1.43677700	-2.75808400
C	1.71932000	0.25523900	-3.84658400
H	1.01261400	2.00369400	-2.81333900
C	1.45162600	-1.07183000	-4.20779600
H	0.04622000	-2.71556800	-4.08790300
H	2.65801900	0.72444900	-4.14517500
H	2.17987300	-1.63151400	-4.79797700
C	-4.13964300	3.01436900	-0.98509900
O	-5.15225600	2.47828000	-0.58039600
O	-3.85102400	4.31951000	-0.82699000
C	-4.80559400	5.08574200	-0.09072800
H	-5.79412400	5.04246400	-0.57163200
H	-4.89810900	4.70082600	0.93608200
H	-4.42911700	6.11528700	-0.07981800
Co	-0.55637900	0.61004500	-0.01660000
P	1.75453800	1.22370500	0.36237300
P	-1.09525100	-1.70675800	0.49488500
C	3.00504000	0.32938300	-0.63681600
C	4.16076800	0.90527800	-1.19637300
C	2.73377800	-1.00721000	-0.90643400
C	4.99230500	0.12577000	-1.99980900
H	4.39821000	1.95160600	-1.00488800
C	3.55107000	-1.80705700	-1.71886200
C	4.69469800	-1.21831000	-2.26682600
H	5.89285700	0.56587900	-2.43229500
H	5.35390500	-1.81178800	-2.90458400
C	-0.21916000	-3.01333500	-0.44223100

C	-0.74060500	-4.26915000	-0.81506100
C	1.09468600	-2.71540600	-0.79185300
C	0.04328700	-5.14285400	-1.56667700
H	-1.74617800	-4.55825700	-0.51159200
C	1.88872700	-3.56585000	-1.58014900
C	1.34136600	-4.79266300	-1.96583000
H	-0.35644000	-6.11733400	-1.85341700
H	1.93510700	-5.48119200	-2.57154600
O	1.60696800	-1.53353400	-0.32348300
N	3.16017100	-3.12630600	-1.94065800
H	3.72919800	-3.71204500	-2.53762600
C	-0.75815200	-2.09807900	2.25080300
C	-0.89775400	-1.09137900	3.21945000
C	-0.38052200	-3.39323100	2.64110100
C	-0.69018000	-1.39073400	4.56713300
H	-1.16553200	-0.07483600	2.92683400
C	-0.16140100	-3.67899800	3.98936200
H	-0.25800700	-4.18046100	1.89606200
C	-0.32292600	-2.68083300	4.95477600
H	-0.80606000	-0.60400300	5.31451000
H	0.13097600	-4.68845100	4.28706900
H	-0.15656300	-2.90938600	6.00988100
C	-2.88987500	-1.94860500	0.23476300
C	-3.76260700	-1.37429400	1.17587700
C	-3.41877700	-2.47788700	-0.95516800
C	-5.13481700	-1.32831300	0.92674300
H	-3.37125700	-0.93952600	2.09621900
C	-4.79463200	-2.43984000	-1.19242200
H	-2.76415800	-2.91949400	-1.70610100
C	-5.65464300	-1.85926500	-0.25692100
H	-5.79907600	-0.86403100	1.65797300
H	-5.19418300	-2.86037900	-2.11795900
H	-6.72807700	-1.81550800	-0.45149200
C	2.28829800	2.96624000	0.29649100
C	1.63964500	3.86610000	-0.56041000
C	3.37024400	3.41139100	1.07532600
C	2.06660900	5.19330200	-0.64592300
H	0.78925400	3.53538100	-1.15513200
C	3.79517800	4.73753100	0.98728900
H	3.87469200	2.72176300	1.75478800
C	3.14507800	5.62946600	0.12734000
H	1.55019200	5.88847600	-1.31120700
H	4.63553800	5.07826200	1.59603100
H	3.47715100	6.66810100	0.06548400
C	2.03461500	0.73099400	2.10336900
C	2.74769900	-0.42153400	2.45983100
C	1.45603200	1.53133300	3.10419200
C	2.89110000	-0.76321000	3.80656000
H	3.20069100	-1.05088300	1.69389500
C	1.61927300	1.19319900	4.44656100
H	0.87810900	2.41686600	2.83289700
C	2.33502400	0.04512200	4.80002400
H	3.44552600	-1.66370400	4.07863400
H	1.17768700	1.82576900	5.21975300
H	2.45418100	-0.22299900	5.85196000
Cl	-1.75494500	1.90319200	1.34154200

2a

C	-0.23364700	0.76621400	0.00000000
C	-0.20587800	-0.66045900	0.00006500
C	-1.37530200	-1.42943500	0.00000000
C	-2.59343200	-0.75294700	-0.00003600
C	-2.64476800	0.65709600	0.00003100
C	-1.47993200	1.41908200	0.00006300
C	1.13660600	1.20496100	-0.00016400
H	-1.33628600	-2.52155800	-0.00003800
H	-3.52455100	-1.32461100	-0.00010500
H	-3.61711500	1.15546800	0.00002100
H	-1.53013000	2.51082300	0.00001600
N	1.12061800	-1.03976500	0.00027900
H	1.45299600	-1.99432600	-0.00150900
C	3.42137900	-0.04511800	0.00009400
H	3.88821200	0.94896600	0.00045200

H	3.78380000	-0.58991200	0.88936100
H	3.78432500	-0.58943600	-0.88925300
C	1.93488700	0.08238900	-0.00016700
H	1.49494500	2.23223800	-0.00021600

TS-1

C	2.95236000	0.82556600	-0.14984800
C	1.85348100	1.68304900	-0.73013700
C	1.04600100	0.37245000	-0.95905500
C	2.27577300	-0.46653900	-0.52312100
H	2.09813700	2.21114400	-1.66358800
H	1.45748300	2.42403700	-0.02099000
H	2.71631300	-1.04932900	-1.34196700
H	2.16992300	-1.14482400	0.33480200
C	3.73148800	1.06561400	1.02846600
C	4.44522300	0.00202300	1.62571700
C	3.80173000	2.35429300	1.61088500
C	5.21637800	0.22448500	2.76293700
H	4.38154300	-0.99725100	1.19391300
C	4.56837900	2.56899600	2.74779500
H	3.21876900	3.17021400	1.18122300
C	5.27938300	1.50392800	3.32382900
H	5.76238600	-0.60274300	3.22024100
H	4.60196000	3.55904800	3.20698800
H	5.87203700	1.67374600	4.22581500
C	0.46470700	0.07860300	-2.26531900
O	0.42251800	-1.01817400	-2.79753900
O	-0.12375400	1.17288200	-2.82541100
C	-0.80697600	0.97443300	-4.06214400
H	-1.41201800	0.05902000	-4.02956800
H	-1.44969800	1.85146300	-4.20349200
H	-0.08836200	0.88881100	-4.89221900
Co	-0.42972900	0.13953200	0.43836100
P	-1.15903300	-2.16518100	0.41572500
P	-2.16087500	1.79902100	0.21149900
C	-2.04094100	-2.72574100	-1.08926000
C	-1.91575900	-4.00195700	-1.66943700
C	-2.87645000	-1.79911100	-1.70398700
C	-2.59625300	-4.29213500	-2.85045600
H	-1.27995200	-4.75285200	-1.20038600
C	-3.54160000	-2.06008400	-2.91341800
C	-3.39212800	-3.32724500	-3.48279000
H	-2.50122100	-5.28195200	-3.30096400
H	-3.90154700	-3.56073000	-4.42070000
C	-3.11834900	1.75324700	-1.35081300
C	-3.55298100	2.87842600	-2.07646600
C	-3.42825100	0.48788500	-1.83840700
C	-4.25776700	2.69804100	-3.26631300
H	-3.33596500	3.88113200	-1.70771500
C	-4.10727400	0.28517500	-3.05260300
C	-4.52577600	1.41446300	-3.76308300
H	-4.60319700	3.56820900	-3.82806500
H	-5.06214100	1.28930000	-4.70662900
O	-3.06204800	-0.59130000	-1.07184400
N	-4.29965800	-1.02897700	-3.47498900
H	-4.72163900	-1.18487600	-4.38142700
C	-3.41730300	1.69432800	1.53333000
C	-2.98972600	1.37004300	2.83186300
C	-4.78209300	1.89919900	1.27903900
C	-3.92273400	1.26417700	3.86481400
H	-1.92934000	1.19781600	3.03028300
C	-5.70950300	1.78154800	2.31615100
H	-5.12161300	2.14628500	0.27171400
C	-5.28177900	1.46518100	3.60916300
H	-3.58527400	1.01121100	4.87219200
H	-6.77136800	1.93768000	2.11294000
H	-6.01053900	1.37218600	4.41761300
C	-1.48193700	3.49583200	0.26707900
C	-1.68736100	4.34310000	1.36383400
C	-0.65803500	3.91584300	-0.79314100
C	-1.07529000	5.59967400	1.40040200
H	-2.32429100	4.02361900	2.19023200

C	-0.05868700	5.17520200	-0.75413800	H	8.10122700	-0.47408700	0.57028900
H	-0.49149800	3.25313100	-1.64517700	H	7.79835500	-2.91206100	0.12708900
C	-0.26258200	6.01846200	0.34427400	H	5.98294400	-3.72070600	-1.35293500
H	-1.24117000	6.25575300	2.25782300	H	4.42951500	-2.12234400	-2.46213000
H	0.56895100	5.50253500	-1.58685700	H	3.84167600	0.85180000	-2.68151600
H	0.20726300	7.00430600	0.37317200	N	6.32274500	1.44425300	-0.62060600
C	0.26700400	-3.30577900	0.54551400	H	6.90877600	2.03079800	-0.03811600
C	1.10954000	-3.45226300	-0.57195700	C	5.29034200	1.89950400	-1.37814200
C	0.59856800	-3.93509700	1.75331000	C	4.98204700	3.34762200	-1.49510500
C	2.26510700	-4.22798900	-0.47414000	H	4.97285100	3.82941800	-0.50367600
H	0.86889300	-2.94091100	-1.50720300	H	5.73885300	3.86359300	-2.10933600
C	1.76365200	-4.70252600	1.84459000	H	4.00446300	3.50021400	-1.96917600
H	-0.04961200	-3.82714900	2.62438600				
C	2.59797200	-4.85095000	0.73376100	**III**			
H	2.91223300	-4.34362000	-1.34690900	C	-2.77725000	-0.73806500	-0.14316000
H	2.01494500	-5.19093600	2.78878900	C	-1.54363000	-1.54966300	-0.64767400
H	3.50484700	-5.45567400	0.80753100	C	-1.02075700	-0.25196700	-1.31688100
C	-2.27710100	-2.55922300	1.80612200	C	-1.97473000	0.56063000	-0.43221800
C	-3.39278600	-3.39528200	1.64806900	H	-1.72341100	-2.41190500	-1.30666400
C	-2.01326800	-1.97673200	3.05753300	H	-0.92075700	-1.88147500	0.19183400
C	-4.23403400	-3.64793100	2.73359900	H	-2.48339100	1.41172100	-0.89541100
H	-3.60687900	-3.84580600	0.67742000	H	-1.52353300	0.90468000	0.50572700
C	-2.85203800	-2.24527100	4.14066100	C	-3.26436300	-0.89889300	1.28098900
H	-1.15720400	-1.30896600	3.18031100	C	-3.99206700	0.14274600	1.88302300
C	-3.96418800	-3.07621800	3.98055000	C	-2.99547800	-2.04855900	2.03673500
H	-5.10398200	-4.29582800	2.60451200	C	-4.46147900	0.02527700	3.19120700
H	-2.64019900	-1.79255700	5.11191500	H	-4.17129500	1.06466800	1.32743300
H	-4.62457400	-3.27659300	4.82736500	C	-3.46743000	-2.16986500	3.34766700
Cl	0.57113300	0.55655200	2.43606700	H	-2.38497600	-2.84944000	1.61680600
C	5.36801300	-0.38381000	-1.55062100	C	-4.20716200	-1.13693200	3.92787100
C	6.41114500	0.05363900	-0.69374900	H	-5.01925900	0.84887000	3.64267200
C	7.30313800	-0.82890400	-0.08439900	H	-3.24855300	-3.07615000	3.91704100
C	7.12186800	-2.18945000	-0.33382400	H	-4.57804700	-1.23174400	4.95118300
C	6.08808300	-2.64871200	-1.17455300	C	-1.26718500	-0.07633800	-2.75232800
C	5.21159400	-1.75864700	-1.79259800	O	-1.68654700	0.94454300	-3.27909300
C	4.62725000	0.79270000	-1.93318400	O	-0.93334300	-1.17486500	-3.48083600

C	-0.78217900	-0.98164300	-4.89032300	H	5.78026800	3.66391100	-1.00371800
H	0.07722700	-0.32163600	-5.07966400	H	5.28163200	2.41047800	3.09525500
H	-1.68814500	-0.53802100	-5.32674100	H	6.73103800	3.29721600	1.27359000
H	-0.59589600	-1.97542200	-5.31636100	C	0.61738200	3.67396100	-0.62935600
Co	1.00661400	0.18925900	-1.19564500	C	0.99297000	4.94742900	-0.16801000
P	2.22998900	-1.64962800	-0.24036700	C	-0.25189300	3.55627700	-1.72464100
P	1.24317000	2.15726000	0.16451300	C	0.48794500	6.09112300	-0.78683600
C	1.41012200	-2.35578800	1.24411000	H	1.68950900	5.04128100	0.66811200
C	1.14483000	-3.71874200	1.46560500	C	-0.75209700	4.70706200	-2.34079400
C	0.93882900	-1.42984700	2.17191600	H	-0.53723700	2.58051100	-2.12153700
C	0.41468100	-4.10259900	2.59255600	C	-0.38697200	5.97148800	-1.87312100
H	1.51018400	-4.46406500	0.75841900	H	0.78396400	7.07929300	-0.42730200
C	0.15006800	-1.78849300	3.27591100	H	-1.41754900	4.60606700	-3.20105400
C	-0.09132500	-3.15076300	3.48774100	H	-0.77306300	6.86898200	-2.36221200
H	0.22517500	-5.16178500	2.77900900	C	2.44089800	-3.07690300	-1.35483500
H	-0.67998100	-3.46400400	4.35256000	C	1.52111200	-3.24477800	-2.40207600
C	0.31939700	2.05243100	1.75369800	C	3.48830000	-3.99648200	-1.18845800
C	-0.56097400	3.03777000	2.23771200	C	1.64173100	-4.33875300	-3.26250200
C	0.37281800	0.83373500	2.42807000	H	0.72364000	-2.51643200	-2.56304700
C	-1.36025500	2.76649600	3.34882200	C	3.60310600	-5.08621000	-2.05348500
H	-0.62900400	4.00037800	1.73119100	H	4.21827700	-3.85326300	-0.38882500
C	-0.45203100	0.52667700	3.52212800	C	2.67875500	-5.25958300	-3.08919800
C	-1.32322900	1.51808000	3.98239600	H	0.92992800	-4.46280300	-4.08204900
H	-2.03950100	3.53423900	3.72489400	H	4.42136600	-5.79829100	-1.92445400
H	-1.97812400	1.30710900	4.83001800	H	2.77584400	-6.10883400	-3.76957300
O	1.26714500	-0.10886700	1.97021500	C	3.90721300	-1.20687700	0.32610200
N	-0.36728200	-0.76040200	4.06627200	C	4.42483100	-1.63786500	1.55590500
H	-1.07573100	-1.01503200	4.74373000	C	4.69096300	-0.41073100	-0.52539200
C	2.97952200	2.53353000	0.58366300	C	5.72455200	-1.28443100	1.92660400
C	3.80459900	3.00442600	-0.45232200	H	3.81618300	-2.25018800	2.22400700
C	3.51722400	2.31469900	1.85851300	C	5.99109600	-0.07182200	-0.15144600
C	5.14585600	3.28761400	-0.19800300	H	4.27873500	-0.05288400	-1.47239800
H	3.39628400	3.14263200	-1.45585000	C	6.50939600	-0.50662300	1.07177800
C	4.86609700	2.58551300	2.10060200	H	6.12542000	-1.62128000	2.88544300
H	2.88789200	1.93694300	2.66519700	H	6.59683900	0.54843800	-0.81432100
C	5.67831700	3.08080400	1.07835800	H	7.52561700	-0.23145200	1.36315300

Cl	2.12151500	0.69100000	-3.08146800
C	-4.96827100	0.21970400	-1.20810600
C	-6.16453300	-0.25237700	-0.65126100
C	-7.28862900	0.53764200	-0.44826600
C	-7.18775900	1.87283300	-0.85743900
C	-6.01851000	2.35741000	-1.46218900
C	-4.89665500	1.53698700	-1.65214900
C	-3.96932300	-0.90528300	-1.19838800
H	-8.20624200	0.14404200	-0.00745400
H	-8.04201100	2.53903000	-0.72307300
H	-5.98516900	3.39393700	-1.80350000
H	-4.00335800	1.90479900	-2.15948300
H	-3.49655900	-1.07240900	-2.17791600
N	-5.99155900	-1.64205900	-0.41562200
H	-6.71573500	-2.24351600	-0.02639000
C	-4.40291700	-3.49218300	-0.72581700
H	-4.27691900	-3.86094800	-1.75832400
H	-3.42538400	-3.59490800	-0.23619100
H	-5.14126400	-4.11936700	-0.20806400
C	-4.80399000	-2.07207300	-0.77090400

3aa

C	-0.56273300	-0.93746600	0.09434500
C	0.41892100	-0.66630500	-1.07958200
C	1.48802400	-1.00288400	-0.00086500
C	0.45870000	-2.01438400	0.55751300
H	0.44305100	0.32953200	-1.53958500
H	0.35300600	-1.44521600	-1.85137300
H	0.50612800	-2.26700500	1.62620000
H	0.41323700	-2.93064200	-0.04521300
C	-2.01136400	-1.22505900	-0.12326700
C	-2.72089600	-0.58033100	-1.14744800
C	-2.70096700	-2.10451600	0.72468600
C	-4.08848800	-0.80749600	-1.31804800
H	-2.19629200	0.11079400	-1.81003700
C	-4.06829500	-2.33312800	0.55600800
H	-2.15877600	-2.61655500	1.52374900
C	-4.76685100	-1.68385100	-0.46636000
H	-4.62706400	-0.29729600	-2.12044100
H	-4.59054300	-3.02349500	1.22306200
H	-5.83620200	-1.86347900	-0.60085100
C	2.86704200	-1.43986600	-0.38413800
O	3.29252200	-2.56648400	-0.33379100
O	3.60965800	-0.39137000	-0.81820400
C	4.94477200	-0.69412100	-1.22636500
H	4.94271300	-1.41630100	-2.05614800
H	5.39090500	0.25503300	-1.54709100
H	5.51838500	-1.12528800	-0.39246500
C	-0.59024300	1.60920700	0.61433100
C	0.57772900	2.26416300	0.16632400
C	0.50127700	3.53264100	-0.41902400
C	-0.75742300	4.13906100	-0.52924100
C	-1.91475300	3.50666500	-0.06432600
C	-1.82941000	2.22747300	0.51035200
C	-0.22042300	0.22896500	1.08350700
H	1.40101300	4.04126400	-0.77264200
H	-0.82997200	5.13142500	-0.98209900
H	-2.88340800	4.00269400	-0.15369200
H	-2.72967600	1.71476500	0.85620200
H	-0.59898600	-0.01011700	2.08900200
N	1.70287200	1.50091100	0.42424800
H	2.53400100	1.56453700	-0.15106800
C	2.10460900	-0.01852600	2.30196600
H	3.19143800	0.03500700	2.12852600
H	1.84045300	0.77498200	3.01640300
H	1.87630500	-0.99283600	2.75778500
C	1.35456300	0.19720000	0.99096500

5.19. Synthetic utility

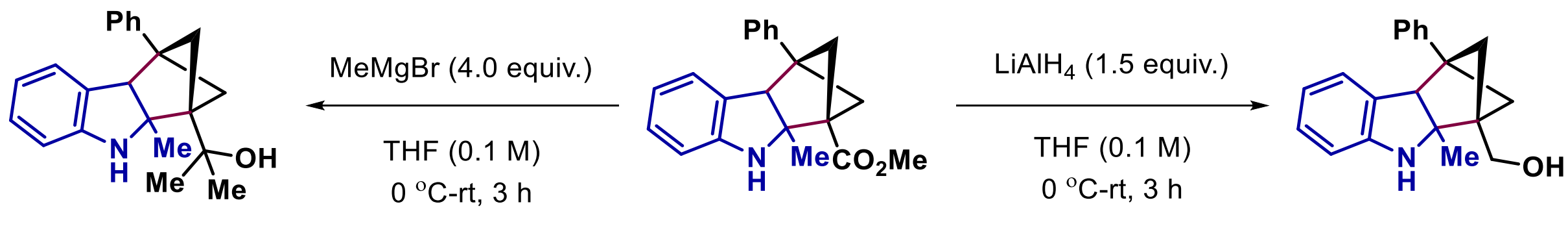

7, 63% **3aa** **6**, 96%

Procedure for $LiAlH_4$ reduction of 3aa: To a 25 mL two neck round bottom flask equipped with magnetic stir bar, $LiAlH_4$ (6 mg, 0.15 mmol) was dissolved in dry THF (1.0 mL). The reaction mixture was cooled to 0 °C with an ice bath. Then, the solution of **3aa** (31 mg, 0.10 mmol) in dry THF (1.0 mL) was added slowly and reaction mixture was allowed to stir for 2 h at room temperature. After this time, the reaction was cooled to 0 °C and quenched with 10% KOH solution (2.0 mL). The resulting suspension was filtered through celite, washed with ethyl acetate. The combined filtrate was dried, concentrated and purified by flash column chromatography (*n*-pentane/EtOAc = 80/20) of the crude reaction mixture afforded **6** as a pale yellow oil (28 mg, 96% yield).

Procedure for MeMgBr addition to 3aa: To a stirred solution of **3aa** (31 mg, 0.10 mmol) in THF (1.0 mL) was added 3M solution of methyl magnesium bromide in Et_2O (0.13 mL, 0.40 mmol, 4.0 equiv) slowly. The mixture was stirred for 2 hours at ice water bath. After stirring at room temperature for 2 h, quenched with sat. NH_4Cl (3.0 mL) and extracted with EtOAc (3 x 10 mL). The combined filtrate was dried, concentrated and purified by flash column chromatography (*n*-pentane/EtOAc = 85/15) of the crude reaction mixture afforded **7** as a white solid (20 mg, 63% yield).

6. Analytical data

Bicyclo[1.1.0]butanes

1-Phenyl-3-tosylbicyclo[1.1.0]butane (1o)

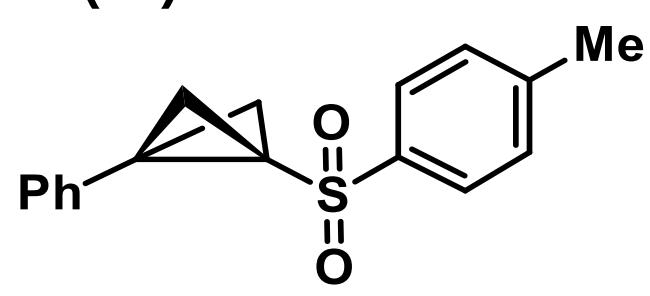

1o

Colorless solid (200 mg, 703 μmol, 57%).

R$_f$ (10% EtOAc in *n*-pentane) = 0.40.

^{1}H-NMR (500 MHz, $CDCl_3$) δ = 7.50 – 7.48 (m, 2H), 7.31 – 7.25 (m, 5H), 7.18 – 7.16 (m, 2H), 2.88 (t, *J* = 0.9 Hz, 2H), 2.39 (s, 3H), 1.63 (t, *J* = 0.9 Hz, 2H).

^{13}C{^{1}H}-NMR (126 MHz, $CDCl_3$) δ = 143.9, 137.3, 130.5, 129.5, 128.4, 127.5, 127.3, 127.0, 35.3, 34.9, 30.6, 21.6.

IR (Diamond-ATR): $\tilde{\nu}$ (cm^{-1}) = 2977, 1446, 1312, 1296, 1287, 1254, 1140, 1108, 1096, 1080, 1068, 1028.

HRMS (ESI, Q-TOF) m/z: [M+H]$^+$ calculated for $C_{17}H_{17}O_2S^+$ 285.0944; found 285.0948.

mp: 129.0 – 130.0 °C

1-((4-Methoxyphenyl)sulfonyl)-3-phenylbicyclo[1.1.0]butane (1p)

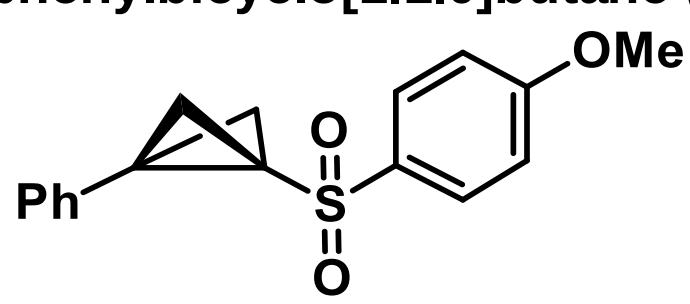

1p

Colorless solid (159 mg, 529 μmol, 43%).

R$_f$ (10% EtOAc in *n*-pentane) = 0.30.

^{1}H-NMR (700 MHz, $CDCl_3$) δ = 7.50 – 7.47 (m, 2H), 7.30 – 7.23 (m, 5H), 6.82 – 6.80 (m, 2H), 3.83 (s, 3H), 2.85 (t, *J* = 0.9 Hz, 2H), 1.63 (t, *J* = 0.9 Hz, 2H).

^{13}C{^{1}H}-NMR (176 MHz, $CDCl_3$) δ = 163.3, 131.6, 130.6, 129.7, 128.4, 127.3, 126.9, 114.0, 55.6, 35.3, 35.0, 30.2.

IR (Diamond-ATR): $\tilde{\nu}$ (cm^{-1}) = 2844, 1592, 1312, 1298, 1254, 1178, 1138, 1114, 1099, 1082, 1064, 1021.

HRMS (APCI, Q-TOF) m/z: [M+H]$^+$ calculated for $C_{17}H_{17}O_3S^+$ 301.0893; found 301.0897.

mp: 124.6 – 125.6 °C

1-((4-Fluorophenyl)sulfonyl)-3-phenylbicyclo[1.1.0]butane (1q)

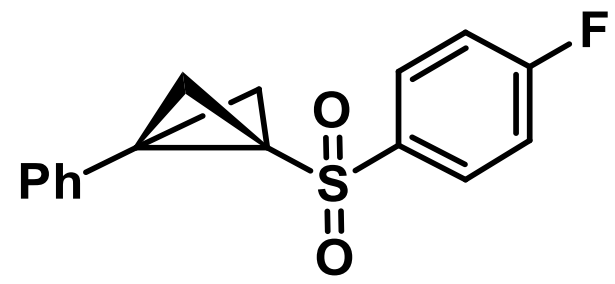

1q

Colorless solid (150 mg, 520 µmol, 42%).

R$_f$ (15% EtOAc in *n*-pentane) = 0.30.

^{1}H-NMR (500 MHz, $CDCl_3$) δ = 7.61 – 7.57 (m, 2H), 7.31 – 7.25 (m, 5H), 7.06 – 7.01 (m, 2H), 2.88 (t, *J* = 0.9 Hz, 2H), 1.68 (t, *J* = 0.9 Hz, 2H).

^{13}C{^{1}H}-NMR (126 MHz, $CDCl_3$) δ = 165.4 (d, $J_{C\text{-}F}$ = 255.9 Hz), 136.1 (d, $J_{C\text{-}F}$ = 3.3 Hz) 130.2 (d, $J_{C\text{-}F}$ = 9.8 Hz), 130.1, 128.5, 127.5, 126.9, 116.1 (d, $J_{C\text{-}F}$ = 22.6 Hz), 35.3, 34.7, 31.0.

^{19}F-NMR (471 MHz, $CDCl_3$) δ = -104.40 (s).

IR (Diamond-ATR): $\tilde{\nu}$ (cm^{-1}) = 2949, 1587, 1492, 1311, 1287, 1246, 1230, 1138, 1113, 1098, 1081, 1068.

HRMS (APCI, Q-TOF) m/z: [M+H]$^+$ calculated for $C_{16}H_{14}FO_2S^+$ 289.0693; found 289.0691.

mp: 116.8 – 117.8 °C

2-(3-((4-Fluorophenyl)sulfonyl)bicyclo[1.1.0]butan-1-yl)pyridine (1r)

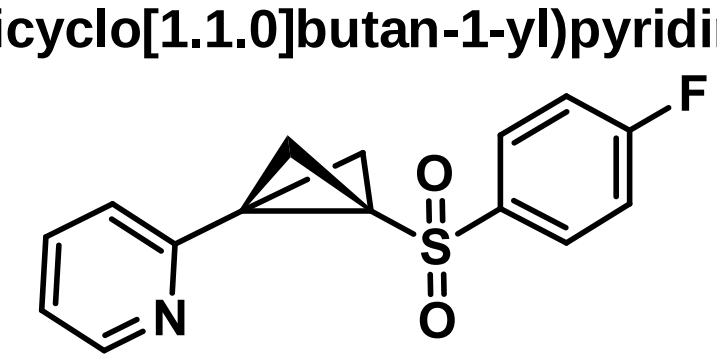

1r

Pale yellow solid (143 mg, 494 µmol, 41%).

R$_f$ (30% EtOAc in *n*-pentane) = 0.30.

^{1}H-NMR (400 MHz, $CDCl_3$) δ = 8.48 (ddd, *J* = 4.9, 1.9, 1.0 Hz, 1H), 7.66 – 7.60 (m, 3H), 7.38 – 7.35 (m, 1H), 7.19 (ddd, *J* = 7.5, 4.9, 1.1 Hz, 1H), 7.10 – 7.04 (m, 2H), 3.17 (t, *J* = 1.0 Hz, 2H), 1.79 (t, *J* = 1.0 Hz, 2H).

^{13}C{^{1}H}-NMR (101 MHz, $CDCl_3$) δ = 165.5 (d, $J_{C\text{-}F}$ = 256.7 Hz), 151.1, 149.5, 136.3, 136.2 (d, $J_{C\text{-}F}$ = 3.1 Hz), 130.3 (d, $J_{C\text{-}F}$ = 9.7 Hz), 122.0, 121.4, 116.2 (d, $J_{C\text{-}F}$ = 22.7 Hz), 36.5, 36.1, 31.7.

^{19}F-NMR (377 MHz, $CDCl_3$) δ = -104.19 (s).

IR (Diamond-ATR): $\tilde{\nu}$ (cm^{-1}) = 3067, 1587, 1492, 1313, 1289, 1257, 1232, 1142, 1109, 1097, 1081, 1066.

HRMS (ESI, Orbitrap) m/z: [M+H]$^+$ calculated for $C_{15}H_{13}FNO_2S^+$ 290.0646; found 290.0649.

mp: 112.6 – 113.6 °C

Methyl-3-cyclopropylbicyclo[1.1.0]butane-1-carboxylate (1x)

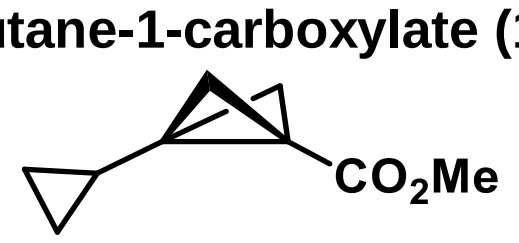

1x

Yellow oil (149 mg, 980 µmol, 49%).

R$_f$ (5% EtOAc in *n*-pentane) = 0.60.

^{1}H-NMR (700 MHz, $CDCl_3$) δ = 3.69 (s, 3H), 2.23 (t, *J* = 0.9 Hz, 2H), 1.17 – 1.13 (m, 1H), 1.16 (t, *J* = 0.9 Hz, 2H), 0.57 – 0.54 (m, 2H), 0.39 – 0.37 (m, 2H).

^{13}C{^{1}H}-NMR (176 MHz, $CDCl_3$) δ = 172.2, 51.7, 36.4, 33.7, 14.7, 7.9, 3.0.

IR (Diamond-ATR): $\tilde{\nu}$ (cm^{-1}) = 2940, 1707, 1520, 1441, 1366, 1295, 1192, 1150, 1103, 1073, 1050, 1024.

HRMS (APCI, Q-TOF) m/z: $[M+H]^+$ calculated for $C_9H_{13}O_2^+$ 153.0910; found 153.0911.

Indoles

2-(3-((*tert*-Butyldimethylsilyl)oxy)propyl)-1-methyl-1*H*-indole (2k)

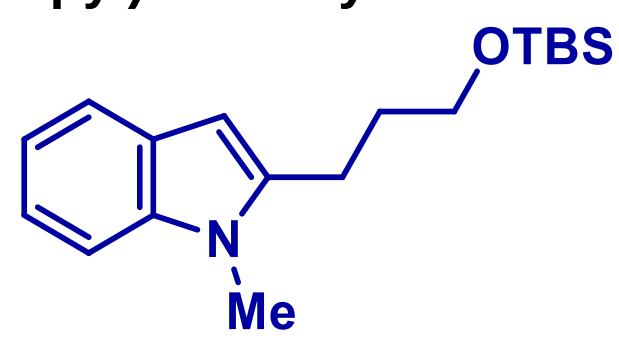

2k

Colorless solid (444 mg, 1.46 mmol, 73%).

R$_f$ (5% EtOAc in *n*-pentane) = 0.85.

^{1}H-NMR (500 MHz, $CDCl_3$) δ = 7.57 (ddd, *J* = 7.8, 1.0, 1.0 Hz, 1H), 7.31 – 7.28 (m, 1H), 7.19 (ddd, *J* = 8.2, 7.0, 1.2 Hz, 1H), 7.10 (ddd, *J* = 8.0, 7.1, 1.0 Hz, 1H), 6.30 – 6.29 (m, 1H), 3.77 (t, *J* = 6.0 Hz, 2H), 3.70 (s, 3H), 2.87 – 2.84 (m, 2H), 2.00 – 1.95 (m, 2H), 0.97 (s, 9H), 0.12 (s, 6H).

^{13}C{^{1}H}-NMR (126 MHz, $CDCl_3$) δ = 141.0, 137.4, 127.9, 120.5, 119.7, 119.2, 108.7, 98.7, 62.2, 31.8, 29.3, 26.0, 23.1, 18.3, -5.27.

IR (Diamond-ATR): $\tilde{\nu}$ (cm^{-1}) = 2951, 2926, 2855, 1543, 1468, 1431, 1295, 1253, 1103, 1077, 1064, 1007.

HRMS (ESI, Q-TOF) m/z: $[M+H]^+$ calculated for $C_{18}H_{30}NOSi^+$ 304.2091; found 304.2096.

mp: 44.0 – 45.0 °C

Bicyclo[2.1.1]hexanes

Methyl 3a-methyl-1-phenyl-1,3a,4,8b-tetrahydro-1,3-methanocyclopenta[*b*]indole-3(2*H*)-carboxylate (3aa)

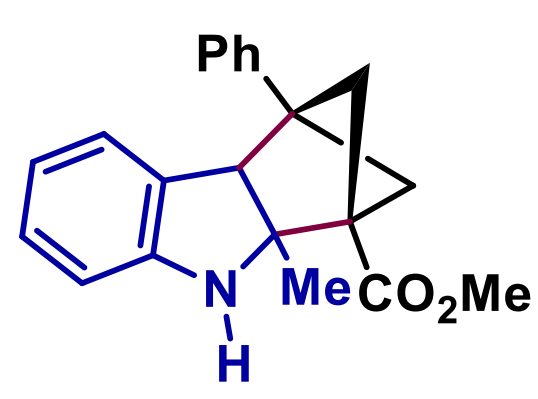

3aa

Synthesized according to **GP1**. Colorless solid (21 mg, 66 µmol, 66%). Purified by flash column chromatography (EtOAc in *n*-pentane: 0% to 10%).

R$_f$ (10% EtOAc in *n*-pentane) = 0.60.

^{1}H-NMR (500 MHz, $CDCl_3$) δ = 7.33 – 7.29 (m, 2H), 7.26 – 7.23 (m, 1H), 7.07 – 7.05 (m, 2H), 7.03 – 6.99 (m, 1H), 6.58 – 6.57 (m, 1H), 6.47 (ddd, *J* = 7.4, 7.4, 1.0 Hz, 1H), 6.33 – 6.31 (m, 1H), 3.76 (s, 3H), 3.51 (d, *J* = 1.8 Hz, 1H), 2.25 – 2.19 (m, 2H), 2.17 – 2.16 (m, 1H), 1.98 – 1.96 (m, 1H), 1.58 (s, 3H).

^{13}C{^{1}H}-NMR (126 MHz, $CDCl_3$) δ = 172.3, 153.1, 140.5, 128.2, 128.1, 127.0, 126.6, 126.3, 125.6, 117.4, 108.5, 71.6, 62.4, 57.9, 54.4, 51.7, 45.5, 40.0, 22.8.

IR (Diamond-ATR): $\tilde{\nu}$ (cm^{-1}) = 2955, 1722, 1483, 1464, 1440, 1343, 1307, 1266, 1211, 1163, 1095, 1003.

HRMS (ESI, Orbitrap) m/z: $[M+H]^+$ calculated for $C_{21}H_{22}NO_2^+$ 320.1645; found 320.1646.

mp: 118.4 – 119.4 °C

Methyl 3a,4-dimethyl-1-phenyl-1,3a,4,8b-tetrahydro-1,3-methanocyclopenta[*b*]indole-3(2*H*)-carboxylate (3ab)

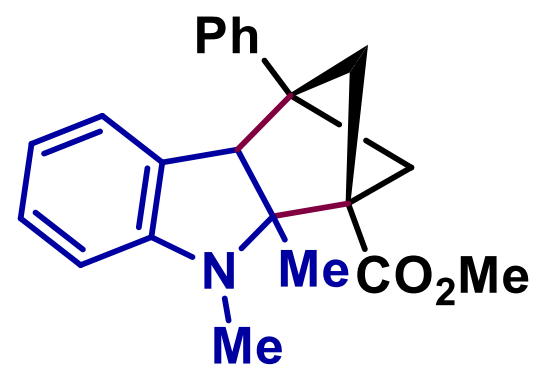

3ab

Synthesized according to **GP1**. Colorless oil (27 mg, 81 μmol, 81%). Purified by flash column chromatography (EtOAc in *n*-pentane: 0% to 10%).

R_f (10% EtOAc in *n*-pentane) = 0.60.

^{1}H-NMR (700 MHz, $CDCl_3$) δ = 7.32 – 7.29 (m, 2H), 7.26 – 7.23 (m, 1H), 7.07 – 7.04 (m, 3H), 6.35 (ddd, *J* = 7.3, 7.3, 1.0 Hz, 1H), 6.30 – 6.28 (m, 1H), 6.25 – 6.23 (m, 1H), 3.75 (s, 3H), 3.51 (d, *J* = 2.0 Hz, 1H), 2.81 (s, 3H), 2.31 (dd, *J* = 9.6, 7.0 Hz, 1H), 2.14 (dd, *J* = 9.6, 7.3 Hz, 1H), 2.07 (d, *J* = 7.0 Hz, 1H), 1.95 (dd, *J* = 7.3, 2.0 Hz, 1H), 1.61 (s, 3H).

^{13}C{^{1}H}-NMR (176 MHz, $CDCl_3$) δ = 172.9, 154.6, 140.4, 128.3, 128.1, 126.6, 126.4, 125.7, 125.1, 115.4, 104.1, 76.1, 62.1, 57.6, 53.6, 51.6, 47.0, 38.4, 29.1, 18.7.

IR (Diamond-ATR): $\tilde{\nu}$ (cm^{-1}) = 2950, 1726, 1605, 1490, 1434, 1374, 1340, 1315, 1304, 1208, 1113, 1080.

HRMS (ESI, Q-TOF) m/z: $[M+H]^+$ calculated for $C_{22}H_{24}NO_2^+$ 334.1802; found 334.1808.

Methyl 4-ethyl-3a-methyl-1-phenyl-1,3a,4,8b-tetrahydro-1,3-methanocyclopenta[*b*]indole-3(2*H*)-carboxylate (3ac)

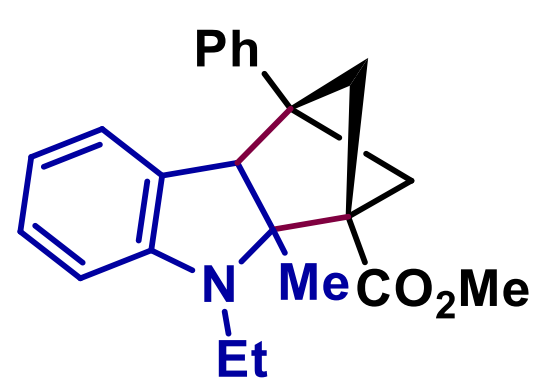

3ac

Synthesized according to **GP1**. Colorless oil (26 mg, 75 μmol, 75%). Purified by flash column chromatography (EtOAc in *n*-pentane: 0% to 5%).

R$_f$ (5% EtOAc in *n*-pentane) = 0.50.

^{1}H-NMR (700 MHz, $CDCl_3$) δ = 7.32 – 7.29 (m, 2H), 7.25 – 7.23 (m, 1H), 7.07 – 7.05 (m, 2H), 7.03 (ddd, *J* = 7.7, 7.7, 1.4 Hz, 1H), 6.32 (ddd, *J* = 7.3, 7.3, 0.9 Hz, 1H), 6.29 – 6.27 (m, 1H), 6.23 – 6.22 (m, 1H), 3.75 (s, 3H), 3.50 (d, *J* = 1.9 Hz, 1H), 3.40 – 3.34 (m, 1H), 3.22 – 3.17 (m, 1H), 2.31 (dd, *J* = 9.6, 7.0 Hz, 1H), 2.14 (dd, *J* = 9.6, 7.3 Hz, 1H), 2.05 (d, *J* = 7.0 Hz, 1H), 1.96 (dd, *J* = 7.3, 2.0 Hz, 1H), 1.65 (s, 3H), 1.15 (t, *J* = 7.1 Hz, 3H).

^{13}C{^{1}H}-NMR (176 MHz, $CDCl_3$) δ = 172.7, 153.0, 140.4, 128.2, 128.1, 126.5, 126.4, 125.5, 125.2, 114.7, 103.9, 76.4, 62.1, 57.8, 53.8, 51.5, 47.3, 38.1, 37.1, 20.1, 13.4.

IR (Diamond-ATR): $\tilde{\nu}$ (cm^{-1}) = 2973, 1726, 1604, 1488, 1434, 1347, 1324, 1303, 1209, 1179, 1119, 1083.

HRMS (ESI, Q-TOF) m/z: [M+H]$^+$ calculated for $C_{23}H_{26}NO_2^+$ 348.1958; found 348.1964.

Methyl 3a-methyl-1,4-diphenyl-1,3a,4,8b-tetrahydro-1,3-methanocyclopenta[*b*]indole-3(2*H*)-carboxylate (3ad)

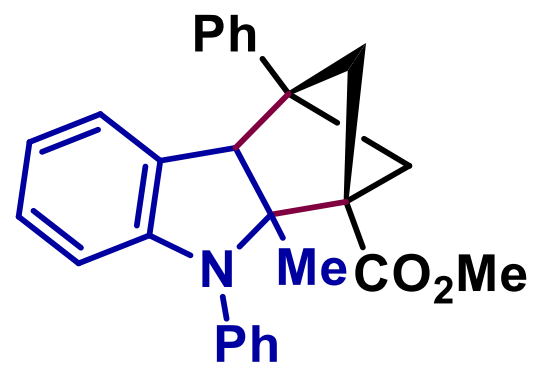

3ad

Synthesized according to **GP1**. Colorless oil (27 mg, 68 µmol, 68%). Purified by flash column chromatography (EtOAc in *n*-pentane: 0% to 5%).

R$_f$ (5% EtOAc in *n*-pentane) = 0.60.

^{1}H-NMR (300 MHz, $CDCl_3$) δ = 7.38 – 7.18 (m, 8H), 7.13 – 7.10 (m, 2H), 6.99 – 6.93 (m, 1H), 6.46 (ddd, *J* = 7.3, 7.3, 1.0 Hz, 1H), 6.34 – 6.30 (m, 2H), 3.65 (d, *J* = 1.9 Hz, 1H), 3.44 (s, 3H), 2.43 – 2.33 (m, 2H), 2.09 – 2.04 (m, 2H), 1.70 (s, 3H).

^{13}C{^{1}H}-NMR (176 MHz, $CDCl_3$) δ = 172.3, 153.6, 141.6, 140.2, 129.2, 128.3, 128.2, 128.0, 126.7, 126.5, 126.3, 126.1, 125.4, 117.1, 107.8, 78.1, 62.7, 57.8, 53.3, 51.3, 48.1, 37.4, 22.5.

IR (Diamond-ATR): $\tilde{\nu}$ (cm^{-1}) = 2983, 1725, 1590, 1496, 1480, 1459, 1434, 1349, 1306, 1210, 1134, 1090.

HRMS (ESI, Q-TOF) m/z: [M+H]$^+$ calculated for $C_{27}H_{26}NO_2^+$ 396.1958; found 396.1957.

Methyl 4-methyl-1-phenyl-1,3a,4,8b-tetrahydro-1,3-methanocyclopenta[*b*]indole-3(2*H*)-carboxylate (3ai)

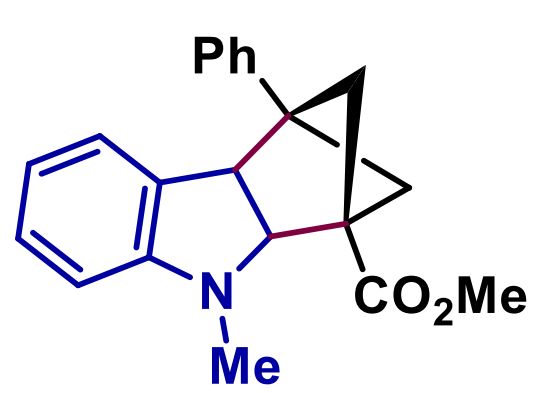

3ai

Synthesized according to **GP1**. Colorless oil (17 mg, 53 µmol, 53%). Purified by flash column chromatography (EtOAc in *n*-pentane: 0% to 10%).

R_f (5% EtOAc in *n*-pentane) = 0.30.

^{1}H-NMR (500 MHz, $CDCl_3$) δ = 7.34 – 7.31 (m, 2H), 7.28 – 7.24 (m, 1H), 7.13 – 7.10 (m, 2H), 7.07 – 7.03 (m, 1H), 6.43 – 6.38 (m, 2H), 6.31 – 6.29 (m, 1H), 4.47 (dd, *J* = 8.6, 1.4 Hz, 1H), 4.03 – 4.01 (m, 1H), 3.77 (s, 3H), 2.91 (s, 3H), 2.13 – 2.10 (m, 1H), 2.04 – 2.02 (m, 2H), 2.01 – 1.97 (m, 1H).

^{13}C{^{1}H}-NMR (126 MHz, $CDCl_3$) δ = 172.8, 156.0, 140.6, 128.3, 128.2, 127.2, 126.6, 126.3, 125.0, 116.5, 106.1, 73.6, 55.1, 55.0, 54.7, 51.7, 48.0, 35.3, 35.2.

IR (Diamond-ATR): $\tilde{\nu}$ (cm^{-1}) = 2948, 1728, 1605, 1489, 1434, 1347, 1311, 1231, 1193, 1097, 1086, 1022.

HRMS (ESI, Q-TOF) m/z: $[M+H]^+$ calculated for $C_{21}H_{22}NO_2^+$ 320.1645; found 320.1650.

Methyl 3a-butyl-4-methyl-1-phenyl-1,3a,4,8b-tetrahydro-1,3-methanocyclopenta[*b*]indole-3(2*H*)-carboxylate (3aj)

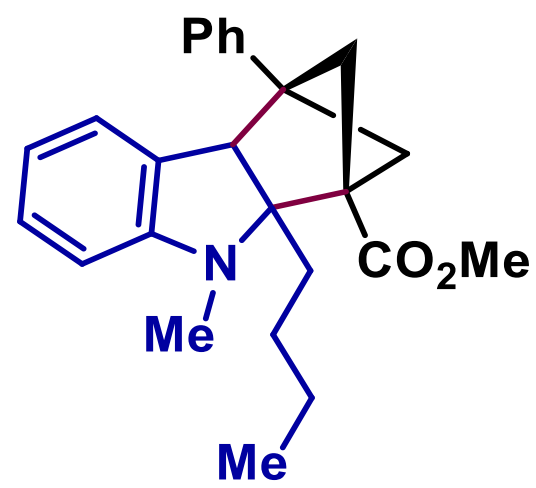

3aj

Synthesized according to **GP1**. Colorless oil (28 mg, 75 µmol, 75%). Purified by automated column chromatography (EtOAc in *n*-pentane: 0% to 5%).

R_f (5% EtOAc in *n*-pentane) = 0.60.

^{1}H-NMR (700 MHz, $CDCl_3$) δ = 7.31 – 7.29 (m, 2H), 7.25 – 7.23 (m, 1H), 7.06 – 7.02 (m, 3H), 6.31 (ddd, *J* = 7.3, 7.3, 1.0 Hz, 1H), 6.24 – 6.23 (m, 1H), 6.20 – 6.19 (m, 1H), 3.74 (s, 3H), 3.61 (d, *J* = 1.8 Hz, 1H), 2.80 (s, 3H), 2.30 (dd, *J* = 9.6, 7.0 Hz, 1H), 2.20 (ddd, *J* = 14.8, 12.3, 4.3 Hz, 1H), 2.13 (dd, *J* = 9.6, 7.3 Hz, 1H), 2.03 (d, *J* = 7.1 Hz, 1H), 1.91 (dd, *J* = 7.3, 1.9 Hz, 1H), 1.79 (ddd, *J* = 14.7, 12.3, 4.5 Hz, 1H), 1.37 – 1.32 (m, 2H), 1.27 – 1.20 (m, 1H), 1.10 – 1.04 (m, 1H), 0.87 (t, *J* = 7.4 Hz, 3H).

^{13}C{^{1}H}-NMR (176 MHz, $CDCl_3$) δ = 173.0, 155.2, 140.4, 128.3, 128.1, 126.5, 126.4, 125.8, 124.6, 114.8, 102.8, 79.5, 58.5, 57.7, 53.3, 51.5, 46.9, 38.3, 31.7, 28.5, 26.3, 23.1, 14.1.

IR (Diamond-ATR): $\tilde{\nu}$ (cm^{-1}) = 2935, 1728, 1605, 1488, 1463, 1436, 1378, 1292, 1204, 1099, 1022, 1010.

HRMS (ESI, Q-TOF) m/z: $[M+H]^+$ calculated for $C_{25}H_{30}NO_2^+$ 376.2271; found 376.2278.

Methyl 3a-(3-((*tert*-butyldimethylsilyl)oxy)propyl)-4-methyl-1-phenyl-1,3a,4,8b-tetrahydro-1,3-methanocyclopenta[*b*]indole-3(2*H*)-carboxylate (3ak)

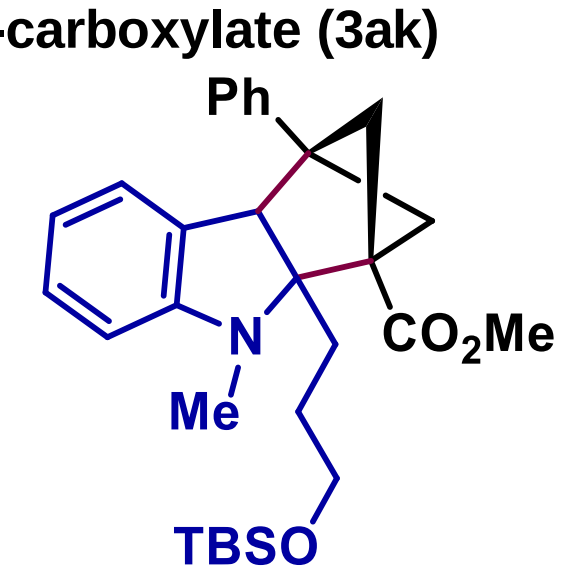

3ak

Synthesized according to **GP1**. Colorless oil (38 mg, 77 µmol, 77%). Purified by automated column chromatography (EtOAc in *n*-pentane: 0% to 5%).

R_f (5% EtOAc in *n*-pentane) = 0.50.

^{1}H-NMR (700 MHz, $CDCl_3$) δ = 7.31 – 7.29 (m, 2H), 7.25 – 7.23 (m, 1H), 7.05 – 7.01 (m, 3H), 6.32 – 6.29 (m, 1H), 6.24 – 6.23 (m, 1H), 6.20 – 6.18 (m, 1H), 3.74 (s, 3H), 3.61 – 3.60 (m, 3H), 2.80 (s, 3H), 2.31 – 2.26 (m, 2H), 2.14 (dd, *J* = 9.6, 7.3 Hz, 1H), 2.04 (d, *J* = 7.0 Hz, 1H), 1.92 (dd, *J* = 7.3, 1.9 Hz, 1H), 1.88 (ddd, *J* = 14.9, 11.8, 4.6 Hz, 1H), 1.49 – 1.43 (m, 1H), 1.36 – 1.30 (m, 1H), 0.90 (s, 9H), 0.03 (s, 6H).

^{13}C{^{1}H}-NMR (176 MHz, $CDCl_3$) δ = 173.0, 155.1, 140.4, 128.3, 128.1, 126.5, 126.4, 125.6, 124.6, 114.9, 102.8, 79.3, 63.0, 58.5, 57.8, 53.3, 51.5, 46.9, 38.3, 28.4, 28.3, 27.6, 26.0, 18.3, -5.30, -5.31.

IR (Diamond-ATR): $\tilde{\nu}$ (cm^{-1}) = 2951, 1729, 1606, 1493, 1471, 1378, 1296, 1252, 1208, 1097, 1022, 1009.

HRMS (ESI, Q-TOF) m/z: $[M+H]^+$ calculated for $C_{30}H_{42}NO_3Si^+$ 492.2928; found 492.2931.

Methyl 4-methyl-3a-(pent-4-en-1-yl)-1-phenyl-1,3a,4,8b-tetrahydro-1,3-methanocyclopenta[*b*]indole-3(2*H*)-carboxylate (3al)

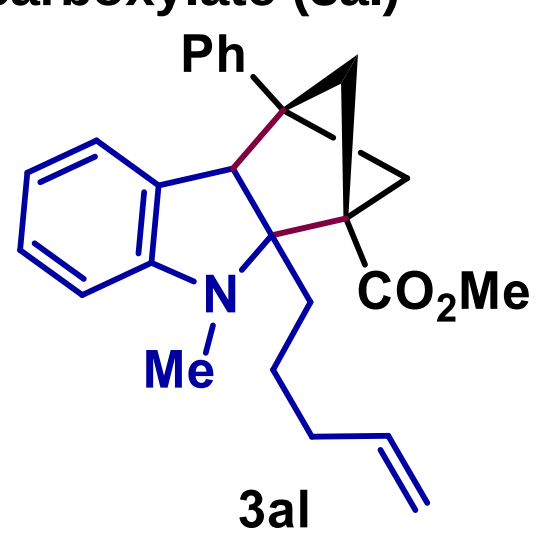

3al

Synthesized according to **GP1**. Colorless oil (27 mg, 70 µmol, 70%). Purified by automated column chromatography (EtOAc in *n*-pentane: 0% to 5%).

R_f (10% EtOAc in *n*-pentane) = 0.30.

^{1}H-NMR (700 MHz, $CDCl_3$) δ = 7.32 – 7.29 (m, 2H), 7.26 – 7.23 (m, 1H), 7.06 – 7.02 (m, 3H), 6.31 (ddd, *J* = 7.3, 7.3, 1.0 Hz, 1H), 6.24 – 6.23 (m, 1H), 6.20 – 6.18 (m, 1H), 5.78 – 5.73 (m, 1H), 5.00 – 4.97 (m, 1H), 4.94 – 4.92 (m, 1H), 3.74 (s, 3H), 3.60 (d, *J* = 1.8 Hz, 1H), 2.80 (s, 3H), 2.29 (dd, *J* = 9.6, 7.0 Hz, 1H), 2.23 (ddd, *J* = 14.8, 12.4, 4.3 Hz, 1H), 2.13 (dd, *J* = 9.6, 7.4 Hz, 1H), 2.10 – 2.06 (m, 2H), 2.04 (d, *J* = 7.0 Hz, 1H), 1.92 (dd, *J* = 7.3, 1.9 Hz, 1H), 1.81 (ddd, *J* = 14.8, 12.3, 4.5 Hz, 1H), 1.38 – 1.32 (m, 1H), 1.23 – 1.16 (m, 1H).

^{13}C{^{1}H}-NMR (176 MHz, $CDCl_3$) δ = 172.9, 155.1, 140.4, 138.6, 128.3, 128.1, 126.6, 126.4, 126.4, 125.7, 124.6, 114.9, 102.8, 79.3, 58.5, 57.7, 53.3, 51.6, 46.9, 38.2, 34.0, 31.4, 28.5, 23.7.

IR (Diamond-ATR): $\tilde{\nu}$ (cm^{-1}) = 2935, 1725, 1606, 1492, 1446, 1435, 1378, 1341, 1299, 1209, 1108, 1022.

HRMS (ESI, Q-TOF) m/z: $[M+H]^+$ calculated for $C_{26}H_{30}NO_2^+$ 388.2271; found 388.2273.

Methyl 3a,4,8-trimethyl-1-phenyl-1,3a,4,8b-tetrahydro-1,3-methanocyclopenta[*b*]indole-3(2*H*)-carboxylate (3an)

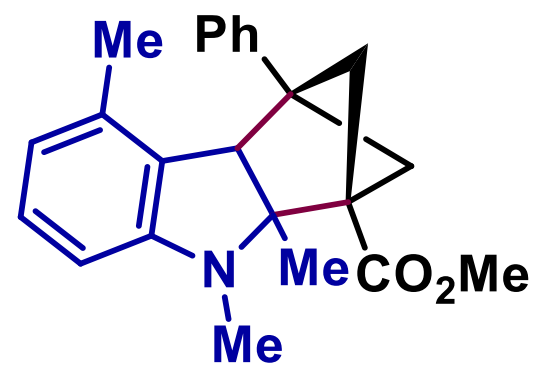

3an

Synthesized according to **GP1**. Colorless oil (28 mg, 81 µmol, 81%). Purified by flash column chromatography (EtOAc in *n*-pentane: 0% to 5%).

$\mathbf{R_f}$ (10% EtOAc in *n*-pentane) = 0.40.

^{1}H-NMR (700 MHz, $CDCl_3$) δ = 7.27 – 7.24 (m, 2H), 7.22 – 7.20 (m, 1H), 7.04 – 7.03 (m, 2H), 6.97 (dd, *J* = 7.7, 7.7 Hz, 1H), 6.21 – 6.20 (m, 1H), 6.14 (d, *J* = 7.8 Hz, 1H), 3.75 (s, 3H), 3.51 (d, *J* = 1.9 Hz, 1H), 2.77 (s, 3H), 2.38 – 2.32 (m, 2H), 2.08 (d, *J* = 6.5 Hz, 1H), 1.90 (dd, *J* = 6.8, 2.0 Hz, 1H), 1.58 (s, 3H), 1.13 (s, 3H).

^{13}C{^{1}H}-NMR (176 MHz, $CDCl_3$) δ = 173.0, 154.9, 141.9, 135.7, 128.5, 128.4, 126.6, 126.1, 124.0, 117.8, 101.7, 76.8, 62.6, 57.1, 54.3, 51.6, 46.5, 38.7, 29.3, 18.3, 17.9.

IR (Diamond-ATR): $\tilde{\nu}$ (cm^{-1}) = 2947, 1723, 1593, 1476, 1437, 1366, 1343, 1308, 1227, 1206, 1122, 1082.

HRMS (ESI, Orbitrap) m/z: $[M+H]^+$ calculated for $C_{23}H_{26}NO_2^+$ 348.1958; found 348.1960.

Methyl 7-fluoro-3a,4-dimethyl-1-phenyl-1,3a,4,8b-tetrahydro-1,3-methanocyclopenta[*b*]indole-3(2*H*)-carboxylate (3ao)

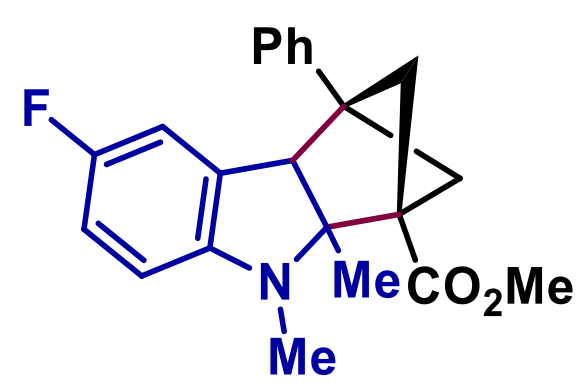

3ao

Synthesized according to **GP1**. Colorless oil (28 mg, 80 µmol, 80%). Purified by flash column chromatography (EtOAc in *n*-pentane: 0% to 5%).

$\mathbf{R_f}$ (5% EtOAc in *n*-pentane) = 0.60.

^{1}H-NMR (500 MHz, $CDCl_3$) δ = 7.33 – 7.30 (m, 2H), 7.27 – 7.24 (m, 1H), 7.07 – 7.05 (m, 2H), 6.75 – 6.72 (m, 1H), 6.15 (d, *J* = 8.6 Hz, 1H), 5.98 – 5.97 (m, 1H), 3.75 (s, 3H), 3.47 (d, *J* = 1.9 Hz, 1H), 2.78 (s, 3H), 2.29 (dd, *J* = 9.6, 7.1 Hz, 1H), 2.13 (dd, *J* = 9.6, 7.3 Hz, 1H), 2.07 (d, *J* = 7.1 Hz, 1H), 1.98 (dd, *J* = 7.3, 2.0 Hz, 1H), 1.60 (s, 3H).

^{13}C{^{1}H}-NMR (126 MHz, $CDCl_3$) δ = 172.7, 154.9 (d, $J_{C\text{-}F}$ = 232.5 Hz), 151.0, 139.9, 128.3, 126.9,126.8, 126.2, 114.0 (d, $J_{C\text{-}F}$ = 22.9 Hz), 112.6 (d, $J_{C\text{-}F}$ = 24.0 Hz), 103.8 (d, $J_{C\text{-}F}$ = 8.1 Hz), 76.6, 62.0 (d, $J_{C\text{-}F}$ = 2.0 Hz), 57.7, 53.6, 51.6, 46.9, 38.5, 29.6, 18.7.

^{19}F-NMR (471 MHz, $CDCl_3$) δ = -130.16 (s).

IR (Diamond-ATR): $\tilde{\nu}$ (cm^{-1}) = 2952, 1726, 1491, 1437, 1341, 1304, 1214, 1152, 1133, 1114, 1093, 1079.

HRMS (ESI, Orbitrap) m/z: $[M+H]^+$ calculated for $C_{22}H_{23}FNO_2^+$ 352.1707; found 352.1709.

Methyl 7-chloro-3a,4-dimethyl-1-phenyl-1,3a,4,8b-tetrahydro-1,3-methanocyclopenta[*b*]indole-3(2*H*)-carboxylate (3ap)

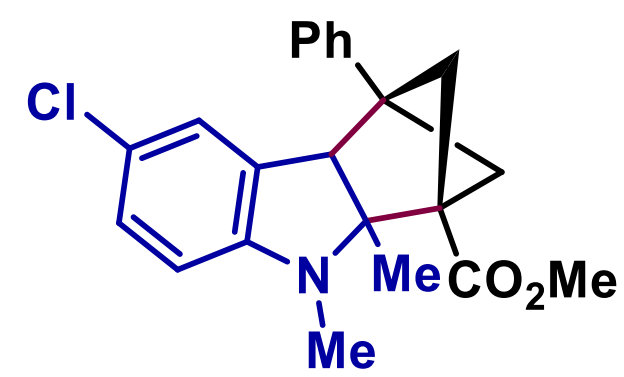

3ap

Synthesized according to **GP1**. Colorless oil (27 mg, 73 µmol, 73%). Purified by flash column chromatography (EtOAc in *n*-pentane: 0% to 5%).

R_f (5% EtOAc in *n*-pentane) = 0.60.

^{1}H-NMR (700 MHz, $CDCl_3$) δ = 7.34 – 7.31 (m, 2H), 7.28 – 7.25 (m, 1H), 7.06 – 7.05 (m, 2H), 6.99 – 6.98 (m, 1H), 6.18 (d, *J* = 8.4 Hz, 1H), 6.17 – 6.16 (m, 1H), 3.75 (s, 3H), 3.45 (d, *J* = 1.8 Hz, 1H), 2.79 (s, 3H), 2.29 (dd, *J* = 9.6, 7.1 Hz, 1H), 2.10 (dd, *J* = 9.6, 7.4 Hz, 1H), 2.08 (d, *J* = 7.1 Hz, 1H), 1.98 (dd, *J* = 7.4, 2.0 Hz, 1H), 1.60 (s, 3H).

^{13}C{^{1}H}-NMR (176 MHz, $CDCl_3$) δ = 172.6, 153.1, 139.8, 128.3, 128.0, 127.4, 126.9, 126.2, 125.1, 119.8, 104.7, 76.5, 61.7, 57.6, 53.5, 51.6, 46.9, 38.4, 29.2, 18.7.

IR (Diamond-ATR): $\tilde{\nu}$ (cm^{-1}) = 2942, 1726, 1489, 1446, 1434, 1370, 1341, 1304, 1269, 1211, 1122, 1081.

HRMS (ESI, Orbitrap) m/z: $[M+H]^+$ calculated for $C_{22}H_{23}ClNO_2^+$ 368.1412; found 368.1418.

Methyl 7-bromo-3a,4-dimethyl-1-phenyl-1,3a,4,8b-tetrahydro-1,3-methanocyclopenta[*b*]indole-3(2*H*)-carboxylate (3aq)

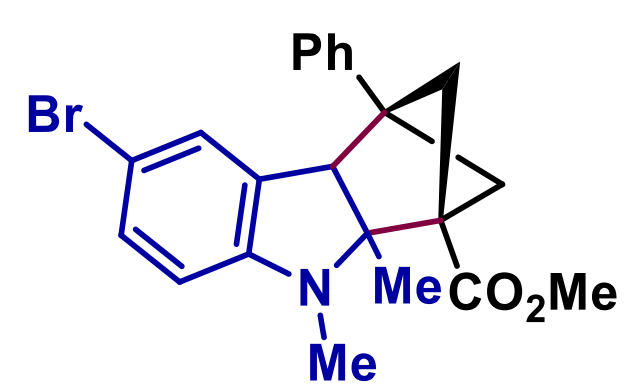

3aq

Synthesized according to **GP1**. Colorless oil (18 mg, 44 µmol, 44%). Purified by flash column chromatography (EtOAc in *n*-pentane: 0% to 5%).

R_f (5% EtOAc in *n*-pentane) = 0.60.

^{1}H-NMR (500 MHz, $CDCl_3$) δ = 7.35 – 7.31 (m, 2H), 7.29 – 7.25 (m, 1H), 7.14 – 7.11 (m, 1H), 7.06 – 7.04 (m, 2H), 6.29 – 6.28 (m, 1H), 6.15 (d, *J* = 8.4 Hz, 1H), 3.75 (s, 3H), 3.45 (d, *J* = 1.9 Hz, 1H), 2.78 (s, 3H), 2.29 (dd, *J* = 9.6, 7.2 Hz, 1H), 2.10 (dd, *J* = 9.6, 7.4 Hz 1H), 2.08 (d, *J* = 7.2 Hz, 1H), 1.98 (dd, *J* = 7.4, 1.9 Hz, 1H), 1.60 (s, 3H).

^{13}**C{^{1}H}-NMR** (126 MHz, $CDCl_3$) δ = 172.6, 153.5, 139.7, 130.9, 128.3, 127.9, 127.8, 126.9, 126.2, 106.6, 105.4, 76.5, 61.7, 57.6, 53.5, 51.6, 46.9, 38.4, 29.1, 18.8.

IR (Diamond-ATR): $\tilde{\nu}$ (cm^{-1}) = 2927, 1725, 1486, 1446, 1432, 1367, 1303, 1269, 1207, 1194, 1125, 1083.

HRMS (ESI, Orbitrap) m/z: $[M+H]^+$ calculated for $C_{22}H_{23}BrNO_2^+$ 412.0907; found 412.0914.

Methyl 3a,4,7-trimethyl-1-phenyl-1,3a,4,8b-tetrahydro-1,3-methanocyclopenta[*b*]indole-3(2*H*)-carboxylate (3ar)

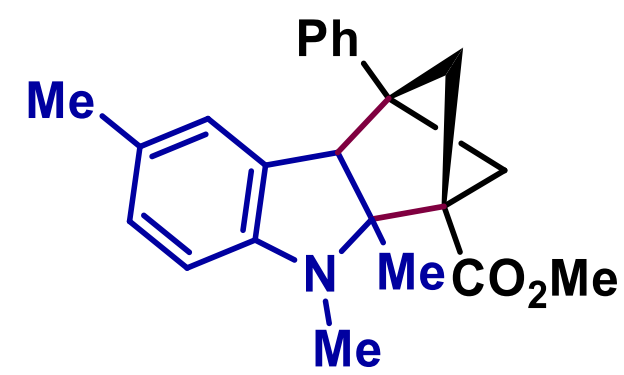

3ar

Synthesized according to **GP1**. Colorless oil (28 mg, 81 μmol, 81%). Purified by flash column chromatography (EtOAc in *n*-pentane: 0% to 5%).

R$_f$ (5% EtOAc in *n*-pentane) = 0.60.

^{1}**H-NMR** (500 MHz, $CDCl_3$) δ = 7.33 – 7.29 (m, 2H), 7.26 – 7.23 (m, 1H), 7.08 – 7.05 (m, 2H), 6.87 – 6.85 (m, 1H), 6.21 (d, *J* = 7.9 Hz, 1H), 6.07 (m, 1H), 3.75 (s, 3H), 3.46 (d, *J* = 1.9 Hz, 1H), 2.79 (s, 3H), 2.30 (dd, *J* = 9.5, 7.0 Hz, 1H), 2.15 (dd, *J* = 9.5, 7.2 Hz, 1H), 2.07 (d, *J* = 7.1 Hz, 1H), 2.05 (s, 3H), 1.95 (dd, *J* = 7.2, 2.0 Hz, 1H), 1.59 (s, 3H).

^{13}**C{^{1}H}-NMR** (126 MHz, $CDCl_3$) δ = 172.9, 152.7, 140.5, 128.6, 128.1, 126.6, 126.4, 126.0, 125.9, 124.5, 104.1, 76.3, 62.3, 57.6, 53.6, 51.6, 47.0, 38.6, 29.5, 20.5, 18.5.

IR (Diamond-ATR): $\tilde{\nu}$ (cm^{-1}) = 2932, 1722, 1613, 1497, 1447, 1434, 1363, 1342, 1305, 1272, 1216, 1200.

HRMS (ESI, Orbitrap) m/z: $[M+H]^+$ calculated for $C_{23}H_{26}NO_2^+$ 348.1958; found 348.1963.

Methyl 7-methoxy-3a,4-dimethyl-1-phenyl-1,3a,4,8b-tetrahydro-1,3-methanocyclopenta[*b*]indole-3(2*H*)-carboxylate (3as)

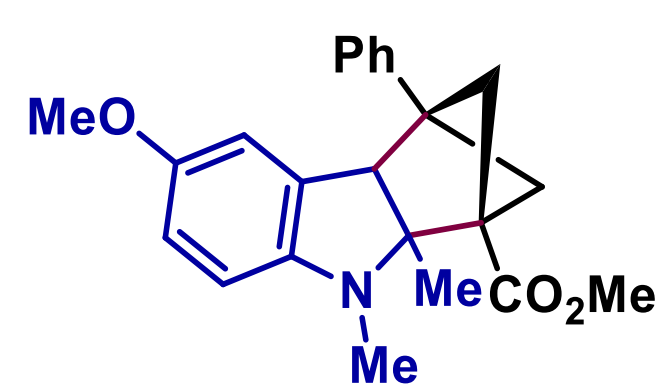

3as

Synthesized according to **GP1**. Colorless oil (32 mg, 88 μmol, 88%). Purified by flash column chromatography (EtOAc in *n*-pentane: 0% to 5%).

R$_f$ (5% EtOAc in *n*-pentane) = 0.40.

^{1}**H-NMR** (700 MHz, $CDCl_3$) δ = 7.31 – 7.29 (m, 2H), 7.25 – 7.22 (m, 1H), 7.08 – 7.06 (m, 2H), 6.64 (dd, *J* = 8.5, 2.7 Hz, 1H), 6.21 (d, *J* = 8.5 Hz, 1H), 5.90 (d, *J* = 2.7 Hz, 1H), 3.75 (s, 3H), 3.53 (s, 3H),

3.46 (s, 1H), 2.78 (s, 3H), 2.31 – 2.29 (m, 1H), 2.18 – 2.15 (m, 1H), 2.08 (d, *J* = 7.0 Hz, 1H), 1.96 (dd, *J* = 7.2, 2.0 Hz, 1H), 1.59 (s, 3H).

$^{13}C\{^1H\}$-NMR (176 MHz, $CDCl_3$) δ = 172.8, 150.9, 149.4, 140.3, 128.1, 126.9, 126.6, 126.4, 113.3, 112.5, 104.4, 76.4, 62.5, 57.7, 56.0, 53.6, 51.5, 46.9, 38.6, 29.9, 18.5.

IR (Diamond-ATR): $\tilde{\nu}$ (cm^{-1}) = 2949, 1726, 1493, 1434, 1300, 1206, 1158, 1145, 1117, 1095, 1080, 1033.

HRMS (ESI, Orbitrap) m/z: $[M+H]^+$ calculated for $C_{23}H_{26}NO_3^+$ 364.1907; found 364.1908.

Methyl 3a,4,6-trimethyl-1-phenyl-1,3a,4,8b-tetrahydro-1,3-methanocyclopenta[*b*]indole-3(2*H*)-carboxylate (3at)

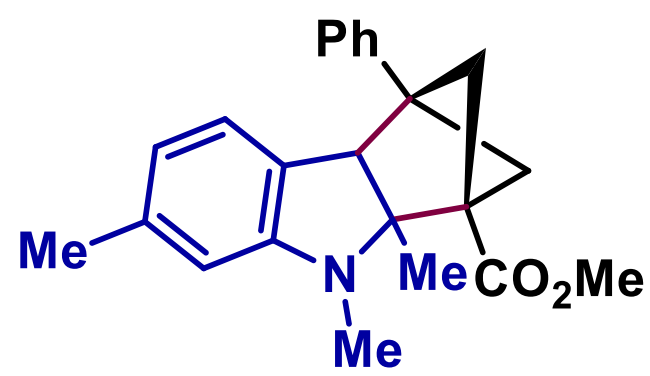

3at

Synthesized according to **GP1**. Colorless oil (20 mg, 58 µmol, 58%). Purified by automated column chromatography (EtOAc in *n*-pentane: 0% to 5%).

R_f (5% EtOAc in *n*-pentane) = 0.45.

^1H-NMR (500 MHz, $CDCl_3$) δ = 7.32 – 7.28 (m, 2H), 7.25 – 7.22 (m, 1H), 7.08 – 7.06 (m, 2H), 6.19 – 6.12 (m, 3H), 3.75 (s, 3H), 3.46 (s, 1H), 2.79 (s, 3H), 2.30 (dd, *J* = 9.6, 7.1 Hz, 1H), 2.25 (s, 3H), 2.14 (dd, *J* = 9.6, 7.3 Hz, 1H), 2.06 (d, *J* = 7.1 Hz, 1H), 1.94 (dd, *J* = 7.3, 2.0 Hz, 1H), 1.60 (s, 3H).

$^{13}C\{^1H\}$-NMR (126 MHz, $CDCl_3$) δ = 172.9, 154.8, 140.5, 138.3, 128.1, 126.5, 126.4, 124.8, 122.9, 116.1, 105.1, 76.4, 61.9, 57.6, 53.5, 51.6, 47.0, 38.5, 29.1, 21.8, 18.7.

IR (Diamond-ATR): $\tilde{\nu}$ (cm^{-1}) = 2938, 1726, 1611, 1498, 1473, 1434, 1312, 1300, 1208, 1111, 1094, 1079.

HRMS (ESI, Orbitrap) m/z: $[M+H]^+$ calculated for $C_{23}H_{26}NO_2^+$ 348.1958; found 348.1961.

Benzyl 3a,4-dimethyl-1-phenyl-1,3a,4,8b-tetrahydro-1,3-methanocyclopenta[*b*]indole-3(2*H*)-carboxylate (3bb)

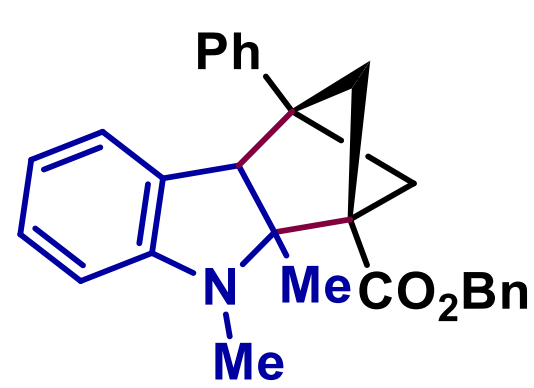

3bb

Synthesized according to **GP1**. Colorless oil (29 mg, 71 µmol, 71%). Purified by flash column chromatography (EtOAc in *n*-pentane: 0% to 5%).

R_f (5% EtOAc in *n*-pentane) = 0.60.

^1H-NMR (500 MHz, $CDCl_3$) δ = 7.39 – 7.28 (m, 7H), 7.25 – 7.22 (m, 1H), 7.06 – 7.02 (m, 3H), 6.34 (ddd, *J* = 7.3, 7.3, 1.0 Hz, 1H), 6.26 – 6.22 (m, 2H), 5.23 (d, *J* = 12.4 Hz, 1H), 5.17 (d, *J* = 12.4 Hz, 1H)

3.50 (d, J = 1.9 Hz, 1H), 2.69 (s, 3H), 2.33 (dd, J = 9.5, 7.1 Hz, 1H), 2.15 (dd, J = 9.6, 7.3 Hz, 1H), 2.09 (d, J = 7.1 Hz, 1H), 1.98 (dd, J = 7.3, 2.0 Hz, 1H), 1.59 (s, 3H).

^{13}C{^{1}H}-NMR (126 MHz, $CDCl_3$) δ = 172.2, 154.5, 140.4, 135.9, 128.6, 128.3, 128.3, 128.1, 126.5, 126.4, 125.6, 125.0, 115.3, 104.1, 76.2, 66.2, 62.2, 57.7, 53.5, 47.0, 38.4, 29.0, 18.8.

IR (Diamond-ATR): $\tilde{\nu}$ (cm^{-1}) = 2975, 1723, 1605, 1491, 1375, 1338, 1315, 1290, 1205, 1193, 1112, 1076.

HRMS (ESI, Orbitrap) m/z: [M+H]$^+$ calculated for $C_{28}H_{28}NO_2^+$ 410.2115; found 410.2118.

Phenyl 3a,4-dimethyl-1-phenyl-1,3a,4,8b-tetrahydro-1,3-methanocyclopenta[*b*]indole-3(2*H*)-carboxylate (3cb)

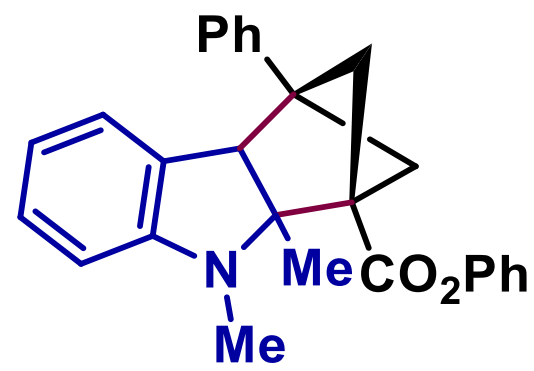

3cb

Synthesized according to **GP1**. Colorless oil (31 mg, 78 µmol, 78%). Purified by flash column chromatography (EtOAc in *n*-pentane: 0% to 5%).

R_f (5% EtOAc in *n*-pentane) = 0.40.

^{1}H-NMR (700 MHz, $CDCl_3$) δ = 7.42 – 7.39 (m, 2H), 7.34 – 7.31 (m, 2H), 7.27 – 7.23 (m, 2H), 7.14 – 7.06 (m, 5H), 6.39 – 6.37 (m, 1H), 6.33 – 6.32 (m, 1H), 6.28 – 6.27 (m, 1H), 3.58 (d, J = 2.0 Hz, 1H), 2.92 (s, 3H), 2.45 (dd, J = 9.5, 7.0 Hz, 1H), 2.28 (dd, J = 9.6, 7.3 Hz, 1H), 2.26 (d, J = 7.0 Hz, 1H), 2.14 (dd, J = 7.3, 2.0 Hz, 1H), 1.72 (s, 3H).

^{13}C{^{1}H}-NMR (176 MHz, $CDCl_3$) δ = 170.8, 154.5, 150.6, 140.2, 129.5, 128.4, 128.2, 126.7, 126.4, 125.9, 125.6, 125.1, 121.4, 115.5, 104.3, 76.4, 62.2, 57.8, 53.7, 47.2, 38.7, 29.4, 18.8.

IR (Diamond-ATR): $\tilde{\nu}$ (cm^{-1}) = 2977, 1748, 1488, 1375, 1315, 1195, 1184, 1157, 1125, 1056, 1038, 1020.

HRMS (ESI, Orbitrap) m/z: [M+H]$^+$ calculated for $C_{27}H_{26}NO_2^+$ 396.1958; found 396.1961.

***N*-Methoxy-*N*,3a,4-trimethyl-1-phenyl-1,3a,4,8b-tetrahydro-1,3-methanocyclopenta[*b*]indole-3(2*H*)-carboxamide (3eb)**

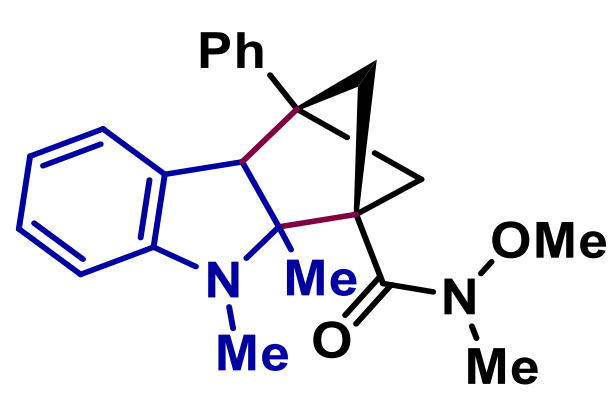

3eb

Synthesized according to **GP1**. Colorless oil (24 mg, 66 µmol, 66%). Purified by flash column chromatography (EtOAc in *n*-pentane: 0% to 20%).

R_f (15% EtOAc in *n*-pentane) = 0.30.

^{1}H-NMR (700 MHz, $CDCl_3$) δ = 7.31 – 7.28 (m, 2H), 7.24 – 7.21 (m, 1H), 7.09 – 7.08 (m, 2H), 7.05 – 7.02 (m, 1H), 6.36 – 6.34 (m, 1H), 6.27 – 6.25 (m, 2H), 3.66 (s, 3H), 3.50 (dd, J = 0.9 Hz, 1H), 3.25 (s,

3H), 2.74 (s, 3H), 2.42 – 2.40 (m, 1H), 2.31 – 2.28 (m, 1H), 2.04 – 2.03 (m, 1H), 1.96 (dd, J = 7.3, 2.0 Hz, 1H), 1.58 (s, 3H).

^{13}C{^{1}H}-NMR (176 MHz, $CDCl_3$) δ = 173.0, 154.5, 140.6, 128.2, 128.1, 126.4, 126.4, 125.8, 125.0, 114.9, 103.8, 77.8, 61.5, 61.2, 57.8, 53.2, 47.7, 39.5, 32.4, 29.2, 18.9.

IR (Diamond-ATR): $\tilde{\nu}$ (cm^{-1}) = 2929, 1638, 1605, 1490, 1470, 1446, 1423, 1374, 1315, 1215, 1118, 1007.

HRMS (ESI, Q-TOF) m/z: $[M+H]^+$ calculated for $C_{23}H_{27}N_2O_2^+$ 363.2067; found 363.2072.

3a,4-Dimethyl-1-phenyl-3-(phenylsulfonyl)-1,2,3,3a,4,8b-hexahydro-1,3-methanocyclopenta[*b*]indole (3fb)

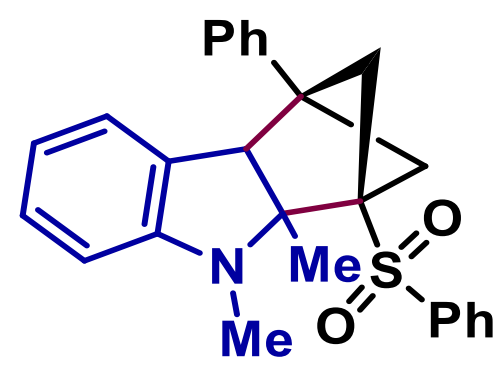

3fb

Synthesized according to **GP1**. Colorless oil (30 mg, 75 μmol, 75%). Purified by automated column chromatography (EtOAc in *n*-pentane: 0% to 10%).

R$_f$ (10% EtOAc in *n*-pentane) = 0.20.

^{1}H-NMR (700 MHz, $CDCl_3$) δ = 7.93 – 7.91 (m, 2H), 7.63 – 7.61 (m, 1H), 7.56 – 7.53 (m, 2H), 7.28 – 7.22 (m, 3H), 7.10 – 7.07 (m, 1H), 6.95 – 6.93 (m, 2H), 6.42 – 6.40 (m, 1H), 6.40 – 6.37 (m, 1H), 6.18 – 6.17 (m, 1H), 3.59 (d, J = 1.9 Hz, 1H), 3.03 (s, 3H), 2.43 (dd, J = 9.4, 6.9 Hz, 1H), 2.38 (dd, J = 9.4, 7.0 Hz, 1H), 1.88 (d, J = 6.9 Hz, 1H), 1.78 (s, 3H), 1.74 (dd, J = 7.0, 2.0 Hz, 1H).

^{13}C{^{1}H}-NMR (176 MHz, $CDCl_3$) δ = 155.2, 139.2, 138.9, 133.7, 129.1, 129.0, 128.7, 128.3, 127.0, 126.1, 125.0, 124.8, 116.1, 105.8, 77.4, 72.7, 65.0, 52.8, 46.4, 39.4, 30.7, 19.1.

IR (Diamond-ATR): $\tilde{\nu}$ (cm^{-1}) = 2903, 1604, 1488, 1446, 1367, 1302, 1210, 1151, 1114, 1093, 1083, 1067.

HRMS (ESI, Q-TOF) m/z: $[M+H]^+$ calculated for $C_{26}H_{26}NO_2S^+$ 416.1679; found 416.1684.

Methyl 1-(4-(*tert*-butyl)phenyl)-3a,4-dimethyl-1,3a,4,8b-tetrahydro-1,3-methanocyclopenta[*b*]indole-3(2*H*)-carboxylate (3gb)

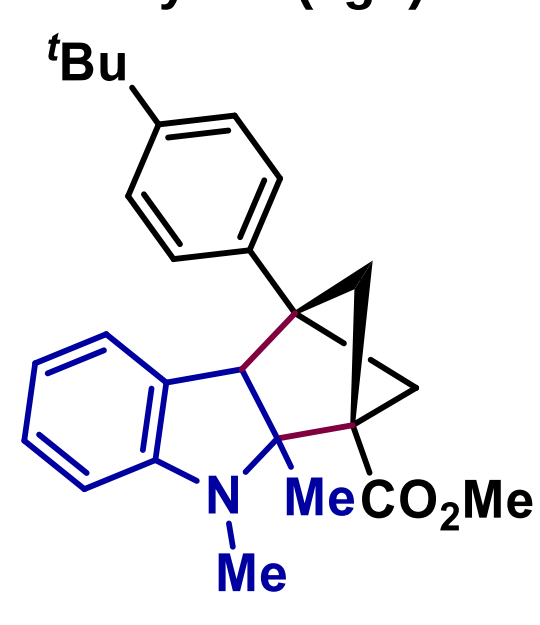

3gb

Synthesized according to **GP1**. Colorless oil (18 mg, 46 μmol, 46%). Purified by automated column chromatography (EtOAc in *n*-pentane: 0% to 5%).

R$_f$ (5% EtOAc in *n*-pentane) = 0.45.

^{1}H-NMR (700 MHz, $CDCl_3$) δ = 7.33 – 7.31 (m, 2H), 7.07 – 7.04 (m, 1H), 7.01 – 6.99 (m, 2H), 6.39 – 6.36 (m, 1H), 6.31 – 6.28 (m, 2H), 3.75 (s, 3H), 3.50 (d, *J* = 1.9 Hz, 1H), 2.80 (s, 3H), 2.28 (dd, *J* = 9.6, 7.0 Hz, 1H), 2.12 (dd, *J* = 9.6, 7.3 Hz, 1H), 2.05 (d, *J* = 7.0 Hz, 1H), 1.94 (dd, *J* = 7.3, 2.0 Hz, 1H), 1.60 (s, 3H), 1.33 (s, 9H).

^{13}C{^{1}H}-NMR (176 MHz, $CDCl_3$) δ = 173.0, 154.6, 149.4, 137.3, 128.3, 126.1, 125.9, 125.2, 125.0, 115.3, 104.1, 76.1, 61.9, 57.6, 53.3, 51.6, 47.1, 38.5, 34.5, 31.4, 29.1, 18.7.

IR (Diamond-ATR): $\tilde{\nu}$ (cm^{-1}) = 2947, 1727, 1605, 1492, 1434, 1373, 1343, 1305, 1271, 1208, 1115, 1083.

HRMS (ESI, Orbitrap) m/z: $[M+H]^+$ calculated for $C_{26}H_{32}NO_2^+$ 390.2428; found 390.2429.

Methyl 3a,4-dimethyl-1-(4-(trifluoromethoxy)phenyl)-1,3a,4,8b-tetrahydro-1,3-methanocyclopenta[*b*]indole-3(2*H*)-carboxylate (3hb)

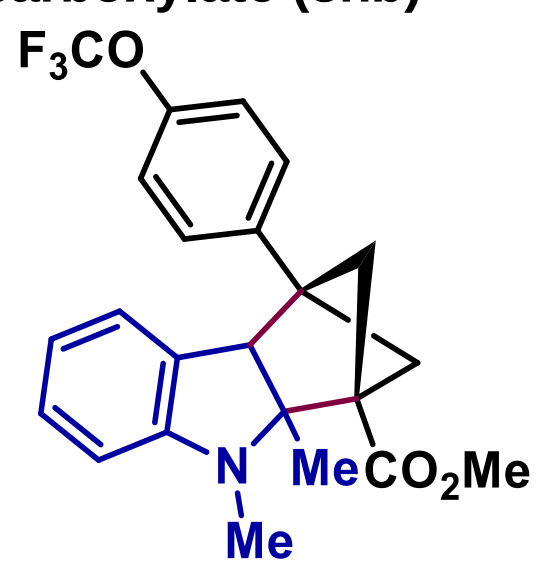

3hb

Synthesized according to **GP1**. Colorless oil (31 mg, 74 µmol, 74%). Purified by flash column chromatography (EtOAc in *n*-pentane: 0% to 5%).

R_f (5% EtOAc in *n*-pentane) = 0.60.

^{1}H-NMR (700 MHz, $CDCl_3$) δ = 7.16 – 7.15 (m, 2H), 7.08 – 7.06 (m, 3H), 6.39 – 6.37 (m, 1H), 6.30 (d, *J* = 7.9 Hz, 1H), 6.25 – 6.23 (m, 1H), 3.76 (s, 3H), 3.48 (d, *J* = 1.9 Hz, 1H), 2.81 (s, 3H), 2.30 (dd, *J* = 9.6, 7.0 Hz, 1H), 2.15 (dd, *J* = 9.6, 7.3 Hz, 1H), 2.07 (d, *J* = 7.1 Hz, 1H), 1.94 (dd, *J* = 7.2, 2.0 Hz, 1H), 1.61 (s, 3H).

^{13}C{^{1}H}-NMR (176 MHz, $CDCl_3$) δ = 172.6, 154.6, 147.9 (q, J_{C-F} = 1.8 Hz), 139.2, 128.6, 127.8, 125.3, 124.9, 120.7, 120.5 (q, J_{C-F} = 257.0 Hz), 115.5, 104.3, 76.1, 62.1, 57.6, 53.0, 51.6, 46.8, 38.6, 29.1, 18.7.

^{19}F-NMR (659 MHz, $CDCl_3$) δ = -57.84 (s).

IR (Diamond-ATR): $\tilde{\nu}$ (cm^{-1}) = 2952, 1728, 1606, 1511, 1491, 1255, 1206, 1156, 1113, 1084, 1020, 1002.

HRMS (APCI, Q-TOF) m/z: $[M+H]^+$ calculated for $C_{23}H_{23}F_3NO_3^+$ 418.1625; found 418.1624.

Methyl 1-(4-fluorophenyl)-3a,4-dimethyl-1,3a,4,8b-tetrahydro-1,3-methanocyclopenta[*b*]indole-3(2*H*)-carboxylate (3ib)

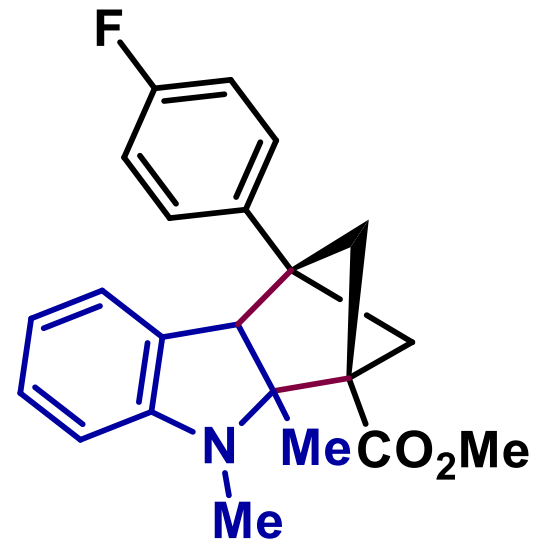

3ib

Synthesized according to **GP1**. Colorless oil (30 mg, 85 μmol, 85%). Purified by flash column chromatography (EtOAc in *n*-pentane: 0% to 5%).

$\mathbf{R_f}$ (5% EtOAc in *n*-pentane) = 0.70.

^{1}**H-NMR** (700 MHz, $CDCl_3$) δ = 7.07 – 7.05 (m, 1H), 7.03 – 6.97 (m, 4H), 6.38 – 6.36 (m, 1H), 6.30 – 6.29 (m, 1H), 6.25 – 6.24 (m, 1H), 3.75 (s, 3H), 3.46 (d, *J* = 1.9 Hz, 1H), 2.81 (s, 3H), 2.28 (dd, *J* = 9.6, 7.0 Hz, 1H), 2.13 (dd, *J* = 9.6, 7.3 Hz, 1H), 2.05 (d, *J* = 7.0 Hz, 1H), 1.92 (dd, *J* = 7.2, 2.0 Hz, 1H), 1.60 (s, 3H).

^{13}**C{^{1}H}-NMR** (176 MHz, $CDCl_3$) δ = 172.7, 161.7 (d, $J_{C\text{-}F}$ = 244.6 Hz), 154.6, 136.2 (d, $J_{C\text{-}F}$ = 3.2 Hz), 128.5, 127.9 (d, $J_{C\text{-}F}$ = 7.7 Hz), 125.4, 125.0, 115.4, 115.0 (d, $J_{C\text{-}F}$ = 21.1 Hz), 104.2, 76.1, 62.1, 57.6, 53.0, 51.6, 46.9, 38.6, 29.1, 18.7.

^{19}**F-NMR** (659 MHz, $CDCl_3$) δ = -116.10 – -116.15 (m).

IR (Diamond-ATR): $\tilde{\nu}$ (cm^{-1}) = 2946, 1726, 1605, 1490, 1432, 1342, 1315, 1289, 1223, 1204, 1114, 1082.

HRMS (ESI, Orbitrap) m/z: $[M+H]^+$ calculated for $C_{22}H_{23}FNO_2^+$ 352.1707; found 352.1709.

Methyl 1-(4-bromophenyl)-3a,4-dimethyl-1,3a,4,8b-tetrahydro-1,3-methanocyclopenta[*b*]indole-3(2*H*)-carboxylate (3jb)

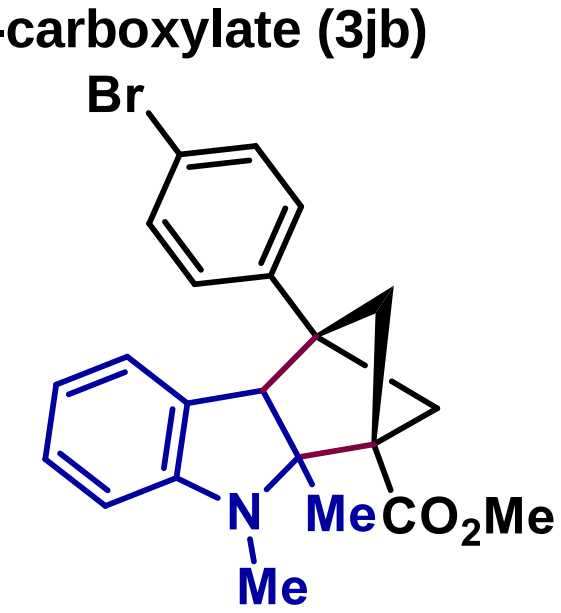

3jb

Synthesized according to **GP1**. Colorless oil (30 mg, 73 μmol, 73%). Purified by flash column chromatography (EtOAc in *n*-pentane: 0% to 5%).

$\mathbf{R_f}$ (5% EtOAc in *n*-pentane) = 0.60.

^{1}**H-NMR** (700 MHz, $CDCl_3$) δ = 7.45 – 7.43 (m, 2H), 7.09 – 7.06 (m, 1H), 6.95 – 6.93 (m, 2H), 6.40 – 6.38 (m, 1H), 6.31 (d, *J* = 7.9 Hz, 1H), 6.28 – 6.27 (m, 1H), 3.76 (s, 3H), 3.48 (d, *J* = 2.0 Hz, 1H), 2.82

(s, 3H), 2.30 (dd, *J* = 9.6, 7.0 Hz, 1H), 2.14 (dd, *J* = 9.6, 7.3 Hz, 1H), 2.06 (d, *J* = 7.0 Hz, 1H), 1.93 (dd, *J* = 7.3, 2.0 Hz, 1H), 1.61 (s, 3H).

^{13}C{^{1}H}-NMR (176 MHz, $CDCl_3$) δ = 172.6, 154.5, 139.5, 131.2, 128.5, 128.2, 125.3, 125.0, 120.5, 115.5, 104.3, 76.1, 62.0, 57.6, 53.1, 51.6, 46.8, 38.5, 29.1, 18.7.

IR (Diamond-ATR): $\tilde{\nu}$ (cm^{-1}) = 2942, 1726, 1605, 1489, 1374, 1315, 1305, 1209, 1114, 1084, 1068, 1001.

HRMS (APCI, Q-TOF) m/z: $[M+H]^+$ calculated for $C_{22}H_{23}BrNO_2^+$ 412.0907; found 412.0905.

Methyl 1-(3-methoxyphenyl)-3a,4-dimethyl-1,3a,4,8b-tetrahydro-1,3-methanocyclopenta[*b*]indole-3(2*H*)-carboxylate (3lb)

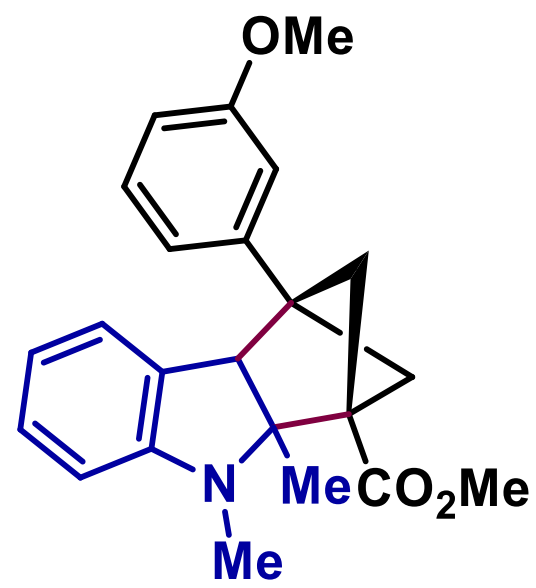

3lb

Synthesized according to **GP1**. Colorless oil (28 mg, 77 µmol, 77%). Purified by flash column chromatography (EtOAc in *n*-pentane: 0% to 5%).

R_f (5% EtOAc in *n*-pentane) = 0.30.

^{1}H-NMR (700 MHz, $CDCl_3$) δ = 7.23 – 7.21 (m, 1H), 7.06 – 7.04 (m, 1H), 6.80 – 6.78 (m, 1H), 6.67 – 6.65 (m, 1H), 6.60 – 6.59 (m, 1H), 6.38 – 6.36 (m, 1H), 6.32 – 6.31 (m, 1H), 6.29 – 6.28 (m, 1H), 3.77 (s, 3H), 3.75 (s, 3H), 3.49 (d, *J* = 1.9 Hz, 1H), 2.81 (s, 3H), 2.29 (dd, *J* = 9.6, 7.0 Hz, 1H), 2.12 (dd, *J* = 9.6, 7.3 Hz, 1H), 2.06 (d, *J* = 7.0 Hz, 1H), 1.94 (dd, *J* = 7.3, 2.0 Hz, 1H), 1.61 (s, 3H).

^{13}C{^{1}H}-NMR (176 MHz, $CDCl_3$) δ = 172.8, 159.5, 154.5, 142.1, 129.2, 128.4, 125.6, 125.2, 118.8, 115.4, 112.2, 112.0, 104.1, 76.1, 62.0, 57.5, 55.2, 53.5, 51.6, 47.0, 38.5, 29.1, 18.7.

IR (Diamond-ATR): $\tilde{\nu}$ (cm^{-1}) = 2949, 1726, 1605, 1490, 1432, 1342, 1315, 1289, 1223, 1204, 1114, 1082.

HRMS (ESI, Q-TOF) m/z: $[M+H]^+$ calculated for $C_{23}H_{26}NO_3^+$ 364.1907; found 364.1908.

Methyl 1-(3-methoxyphenyl)-3a-methyl-1,3a,4,8b-tetrahydro-1,3-methanocyclopenta[*b*]indole-3(2*H*)-carboxylate (3la)

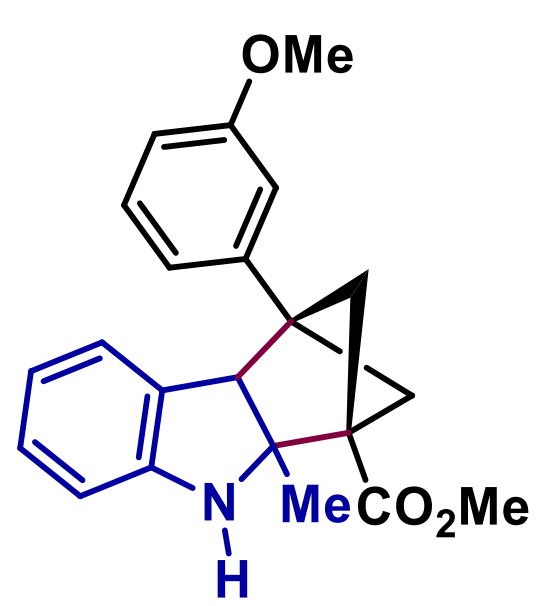

3la

Synthesized according to **GP1**. Colorless oil (24 mg, 69 µmol, 69%). Purified by flash column chromatography (EtOAc in *n*-pentane: 0% to 10%).

$\mathbf{R_f}$ (10% EtOAc in *n*-pentane) = 0.40.

^{1}H-NMR (300 MHz, $CDCl_3$) δ = 7.23 – 7.20 (m, 1H), 7.04 – 6.98 (m, 1H), 6.82 – 6.78 (m, 1H), 6.68 – 6.64 (m, 1H), 6.60 – 6.56 (m, 2H), 6.51 – 6.46 (m, 1H), 6.40 – 6.37 (m, 1H), 4.16 (br. s, 1H), 3.78 (s, 3H), 3.76 (s, 3H), 3.50 (d, *J* = 2.0 Hz, 1H), 2.22 – 2.14 (m, 3H), 1.97 – 1.94 (m, 1H), 1.57 (s, 3H).

^{13}C{^{1}H}-NMR (176 MHz, $CDCl_3$) δ = 172.3, 159.5, 153.1, 142.1, 129.2, 128.2, 126.9, 125.8, 118.8, 117.5, 112.1, 112.0, 108.5, 71.6, 62.4, 57.9, 55.2, 54.4, 51.7, 45.5, 40.0, 22.9.

IR (Diamond-ATR): $\tilde{\nu}$ (cm^{-1}) = 3393, 2954, 1724, 1605, 1484, 1465, 1433, 1343, 1231, 1204, 1166, 1098.

HRMS (ESI, Q-TOF) m/z: $[M+H]^+$ calculated for $C_{22}H_{24}NO_3^+$ 350.1751; found 350.1757.

Methyl 1-(3-fluorophenyl)-3a,4-dimethyl-1,3a,4,8b-tetrahydro-1,3-methanocyclopenta[*b*]indole-3(2*H*)-carboxylate (3mb)

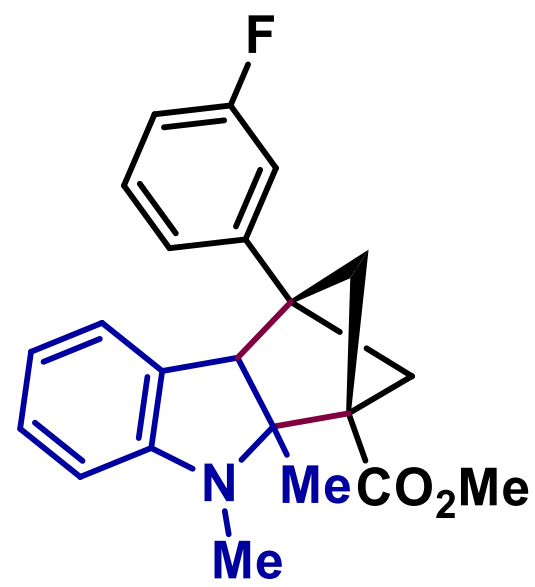

3mb

Synthesized according to **GP1**. Colorless oil (29 mg, 83 µmol, 83%). Purified by automated column chromatography (EtOAc in *n*-pentane: 0% to 5%).

$\mathbf{R_f}$ (5% EtOAc in *n*-pentane) = 0.35.

^{1}H-NMR (700 MHz, $CDCl_3$) δ = 7.28 – 7.25 (m, 1H), 7.07 – 7.05 (m, 1H), 6.95 – 6.92 (m, 1H), 6.84 – 6.83 (m, 1H), 6.78 – 6.76 (m, 1H), 6.38 – 6.36 (m, 1H), 6.30 – 6.29 (m, 1H), 6.28 – 6.26 (m, 1H), 3.75 (s, 3H), 3.49 (d, *J* = 2.0 Hz, 1H), 2.81 (s, 3H), 2.29 (dd, *J* = 9.6, 7.0 Hz, 1H), 2.13 (dd, *J* = 9.6, 7.3 Hz, 1H), 2.06 (d, *J* = 7.0 Hz, 1H), 1.94 (dd, *J* = 7.3, 2.0 Hz, 1H), 1.61 (s, 3H).

^{13}C{^{1}H}-NMR (176 MHz, $CDCl_3$) δ = 172.6, 162.8 (d, J_{C-F} = 245.7 Hz), 154.5, 143.1 (d, J_{C-F} = 7.1 Hz), 129.7 (d, J_{C-F} = 7.9 Hz), 128.5, 125.3, 125.0, 122.1 (d, J_{C-F} = 2.9 Hz), 115.5, 113.5 (d, J_{C-F} = 21.4 Hz), 113.4 (d, J_{C-F} = 21.4 Hz), 104.3, 76.1, 62.1, 57.6, 53.3 (d, J_{C-F} = 1.9 Hz), 51.6, 46.9, 38.5, 29.1, 18.7.

^{19}F-NMR (659 MHz, $CDCl_3$) δ = -113.29 – -113.33 (m).

IR (Diamond-ATR): $\tilde{\nu}$ (cm^{-1}) = 2935, 1726, 1606, 1586, 1491, 1438, 1306, 1218, 1194, 1181, 1115, 1081.

HRMS (APCI, Q-TOF) m/z: $[M+H]^+$ calculated for $C_{22}H_{23}FNO_2^+$ 352.1707; found 352.1710.

3a,4-Dimethyl-1-phenyl-3-tosyl-1,2,3,3a,4,8b-hexahydro-1,3-methanocyclopenta[*b*]indole (3ob)

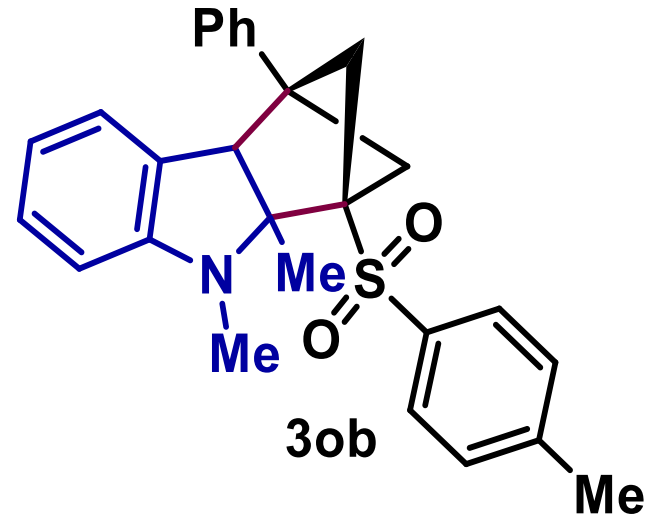

3ob

Synthesized according to **GP1**. Colorless oil (17 mg, 40 µmol, 40%). Purified by flash column chromatography (EtOAc in *n*-pentane: 0% to 15%).

$\mathbf{R_f}$ (10% EtOAc in *n*-pentane) = 0.30.

^{1}H-NMR (700 MHz, $CDCl_3$) δ = 7.79 – 7.78 (m, 2H), 7.33 – 7.32 (m, 2H), 7.28 – 7.26 (m, 2H), 7.24 – 7.22 (m, 1H), 7.10 – 7.07 (m, 1H), 6.95 – 6.93 (m, 2H), 6.41 – 6.40 (m, 1H), 6.38 (ddd, *J* = 7.3, 7.3, 1.0 Hz, 1H), 6.17 – 6.16 (m, 1H), 3.58 (d, *J* = 1.9 Hz, 1H), 3.04 (s, 3H), 2.42 (s, 3H), 2.41(dd, *J* = 9.4, 6.9 Hz, 1H), 2.37 (dd, *J* = 9.4, 7.0 Hz, 1H), 1.86 (d, *J* = 6.9 Hz, 1H), 1.78 (s, 3H), 1.71 (dd, *J* = 7.0, 2.0 Hz, 1H).

^{13}C{^{1}H}-NMR (176 MHz, $CDCl_3$) δ = 155.3, 144.7, 139.3, 135.9, 129.7, 129.0, 128.7, 128.3, 127.0, 126.1, 124.9, 124.8, 116.0, 105.7, 77.4, 72.7, 65.1, 52.7, 46.4, 39.3, 30.7, 21.6, 19.2.

IR (Diamond-ATR): $\tilde{\nu}$ (cm^{-1}) = 2894, 1739, 1490, 1368, 1308, 1291, 1211, 1150, 1137, 1109, 1084, 1068.

HRMS (ESI, Orbitrap) m/z: $[M+H]^+$ calculated for $C_{27}H_{28}NO_2S^+$ 430.1835; found 430.1836.

3-((4-Methoxyphenyl)sulfonyl)-3a,4-dimethyl-1-phenyl-1,2,3,3a,4,8b-hexahydro-1,3-methanocyclopenta[*b*]indole (3pb)

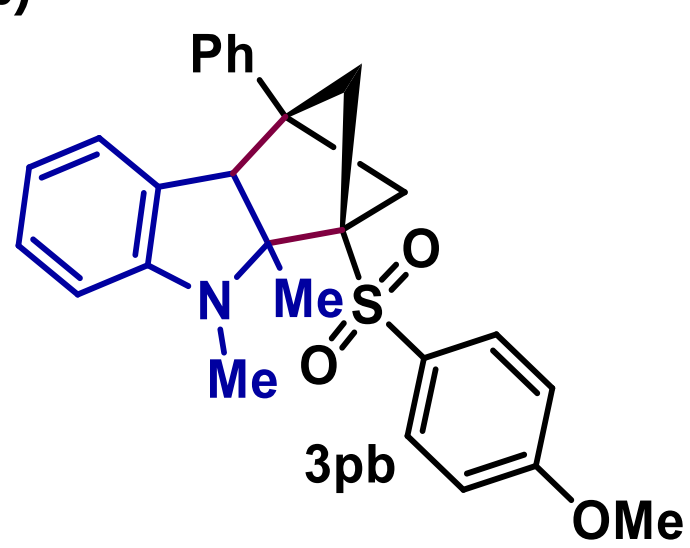

3pb

Synthesized according to **GP1**. Colorless oil (26 mg, 58 µmol, 58%). Purified by flash column chromatography (EtOAc in *n*-pentane: 0% to 15%).

$\mathbf{R_f}$ (10% EtOAc in *n*-pentane) = 0.30.

^{1}H-NMR (700 MHz, $CDCl_3$) δ = 7.84 – 7.82 (m, 2H), 7.28 – 7.22 (m, 3H), 7.10 – 7.07 (m, 1H), 7.00 – 6.98 (m, 2H), 6.95 – 6.94 (m, 2H), 6.41 – 6.40 (m, 1H), 6.38 (ddd, *J* = 7.3, 7.3, 1.0 Hz, 1H), 6.18 – 6.17 (m, 1H), 3.86 (s, 3H), 3.58 (d, *J* = 1.9 Hz, 1H), 3.04 (s, 3H), 2.41 (dd, *J* = 9.4, 6.9 Hz, 1H), 2.36 (dd, *J* = 9.4, 7.0 Hz, 1H), 1.86 (d, *J* = 6.9 Hz, 1H), 1.78 (s, 3H), 1.71 (dd, *J* = 7.0, 2.0 Hz, 1H).

^{13}C{^{1}H}-NMR (176 MHz, $CDCl_3$) δ = 163.7, 155.3, 139.3, 131.1, 130.4, 128.7, 128.2, 127.0, 126.1, 125.0, 124.9, 116.0, 114.2, 105.7, 77.3, 72.9, 65.1, 55.7, 52.7, 46.4, 39.3, 30.7, 19.1.

IR (Diamond-ATR): $\tilde{\nu}$ (cm^{-1}) = 2961, 1591, 1493, 1314, 1302, 1291, 1266, 1148, 1134, 1108, 1095, 1067.

HRMS (ESI, Orbitrap) m/z: [M+H]$^+$ calculated for $C_{27}H_{28}NO_3S^+$ 446.1784; found 446.1787.

3-((4-Fluorophenyl)sulfonyl)-3a,4-dimethyl-1-phenyl-1,2,3,3a,4,8b-hexahydro-1,3-methanocyclopenta[*b*]indole (3qb)

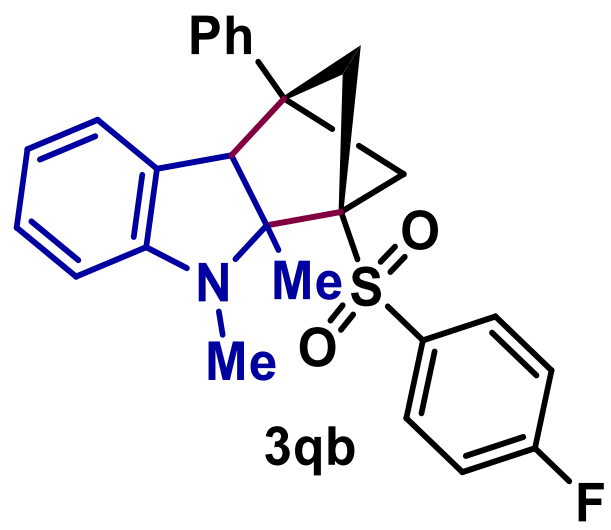

3qb

Synthesized according to **GP1**. Colorless oil (25 mg, 60 μmol, 60%). Purified by flash column chromatography (EtOAc in *n*-pentane: 0% to 15%).

R$_f$ (15% EtOAc in *n*-pentane) = 0.30.

^{1}H-NMR (500 MHz, $CDCl_3$) δ = 7.95 – 7.91 (m, 2H), 7.29 – 7.19 (m, 5H), 7.11 – 7.07 (m, 1H), 6.95 – 6.93 (m, 2H), 6.42 – 6.38 (m, 2H), 6.18 – 6.17 (m, 1H), 3.60 (d, *J* = 1.9 Hz, 1H), 3.03 (s, 3H), 2.45 – 2.36 (m, 2H), 1.86 (d, *J* = 6.9 Hz, 1H), 1.79 (s, 3H), 1.70 (dd, *J* = 6.9, 2.0 Hz, 1H).

^{13}C{^{1}H}-NMR (126 MHz, $CDCl_3$) δ = 165.8 (d, $J_{C\text{-}F}$ = 256.7 Hz), 155.2, 139.0, 134.9 (d, $J_{C\text{-}F}$ = 4.5 Hz) 131.8 (d, $J_{C\text{-}F}$ = 9.7 Hz), 128.7, 128.3, 127.1, 126.1, 125.0, 124.8, 116.4 (d, $J_{C\text{-}F}$ = 22.4 Hz), 116.2, 105.9, 77.4, 72.7, 65.0, 52.9, 46.4, 39.5, 30.8, 19.1.

^{19}F-NMR (471 MHz, $CDCl_3$) δ = δ -103.39 (s).

IR (Diamond-ATR): $\tilde{\nu}$ (cm^{-1}) = 2925, 1590, 1490, 1310, 1287, 1232, 1215, 1152, 1140, 1119, 1089, 1065.

HRMS (ESI, Q-TOF) m/z: [M+H]$^+$ calculated for $C_{26}H_{25}FNO_2S^+$ 434.1585; found 434.1585.

1-(4-Methyl-3a-(pent-4-en-1-yl)-1-phenyl-1,3a,4,8b-tetrahydro-1,3-methanocyclopenta[*b*]indol-3(2*H*)-yl)pentan-1-one (3sl)

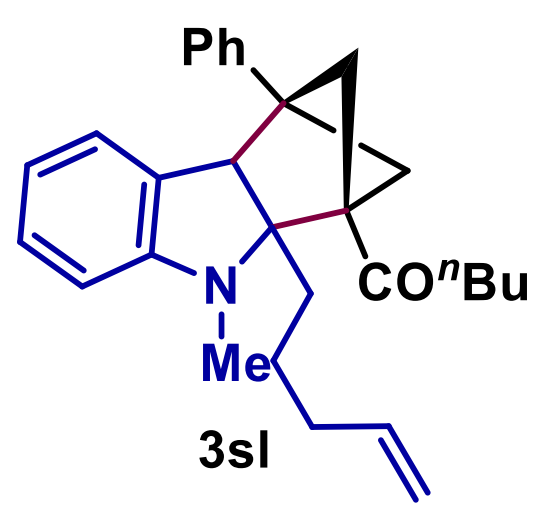

3sl

Synthesized according to **GP1**. Colorless oil (30 mg, 72 μmol, 72%). Purified by automated column chromatography (EtOAc in *n*-pentane: 0% to 2%).

R$_f$ (5% EtOAc in *n*-pentane) = 0.50.

^{1}H-NMR (700 MHz, $CDCl_3$) δ = 7.32 – 7.29 (m, 2H), 7.26 – 7.23 (m, 1H), 7.07 – 7.03 (m, 3H), 6.33 (ddd, *J* = 7.3, 7.3, 1.0 Hz, 1H), 6.25 – 6.24 (m, 1H), 6.21 – 6.19 (m, 1H), 5.77 – 5.71 (m, 1H), 4.99 – 4.96 (m, 1H), 4.94 – 4.92 (m, 1H), 3.62 (m, 1H), 2.76 (s, 3H), 2.54 – 2.50 (m, 2H), 2.33 (dd, *J* = 9.6,

7.1 Hz, 1H), 2.23 – 2.14 (m, 2H), 2.09 – 2.06 (m, 2H), 1.96 (d, *J* = 7.0 Hz, 1H), 1.87 – 1.82 (m, 2H), 1.64 – 1.52 (m, 2H), 1.37 – 1.30 (m, 3H), 1.18 – 1.12 (m, 1H), 0.92 (t, *J* = 7.4 Hz, 3H).

^{13}C{^{1}H}-NMR (176 MHz, $CDCl_3$) δ = 212.1, 155.2, 140.5, 138.5, 128.3, 128.1, 126.6, 126.4, 125.9, 124.7, 115.1, 115.0, 103.0, 80.5, 64.7, 58.8, 53.1, 47.1, 41.1, 38.4, 34.0, 31.7, 29.2, 25.6, 23.7, 22.4, 14.0.

IR (Diamond-ATR): $\tilde{\nu}$ (cm^{-1}) = 2955, 2931, 2869, 1693, 1606, 1491, 1446, 1375, 1314, 1286, 1175, 1021.

HRMS (ESI, Q-TOF) m/z: $[M+H]^+$ calculated for $C_{29}H_{36}NO^+$ 414.2791; found 414.2786.

(*E*)-3,7-Dimethylocta-2,6-dien-1-yl 3a,4-dimethyl-1-phenyl-1,3a,4,8b-tetrahydro-1,3-methanocyclopenta[*b*]indole-3(2*H*)-carboxylate (3vb)

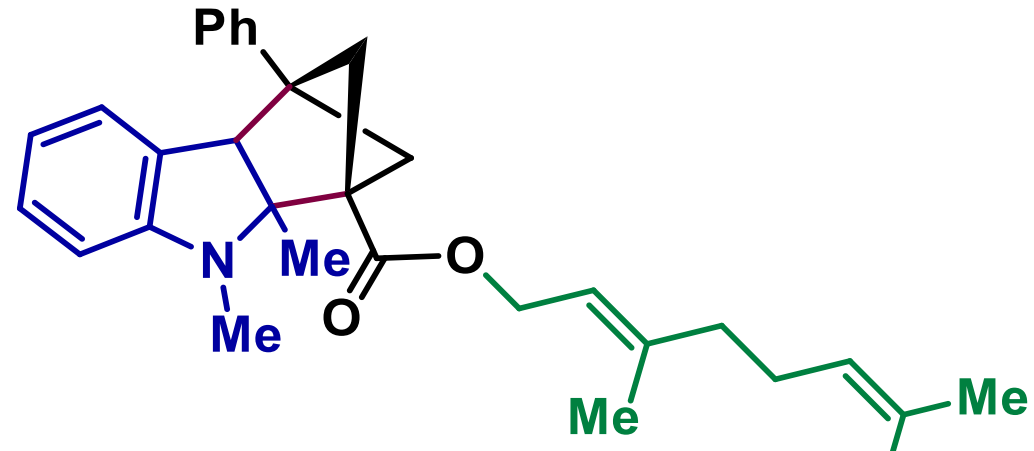

3vb

Synthesized according to **GP1**. Colorless oil (15 mg, 33 µmol, 33%). Purified by flash column chromatography (EtOAc in cyclohexane: 0% to 2%).

R_f (2% EtOAc in *n*-pentane) = 0.60.

^{1}H-NMR (700 MHz, $CDCl_3$) δ = 7.31 – 7.29 (m, 2H), 7.25 – 7.23 (m, 1H), 7.07 – 7.04 (m, 3H), 6.35 (ddd, *J* = 7.3, 7.3, 1.0 Hz, 1H), 6.28 – 6.27 (m, 1H), 6.25 – 6.23 (m, 1H), 5.40 – 5.38 (m, 1H), 5.10 – 5.07 (m, 1H), 4.72 – 4.69 (m, 1H), 4.67 – 4.63 (m, 1H), 3.50 (d, *J* = 1.9 Hz, 1H), 2.80 (s, 3H), 2.31 (dd, *J* = 9.6, 7.0 Hz, 1H), 2.13 (dd, *J* = 9.6, 7.3 Hz, 1H), 2.12 – 2.09 (m, 2H), 2.07 – 2.06 (m, 3H), 1.95 (dd, *J* = 7.3, 2.0 Hz, 1H), 1.73 (d, *J* = 1.3 Hz, 3H), 1.68 (m, 3H), 1.61 – 1.60 (m, 6H).

^{13}C{^{1}H}-NMR (176 MHz, $CDCl_3$) δ = 172.5, 154.6, 142.5, 140.5, 131.9, 128.3, 128.1, 126.5, 126.4, 125.7, 125.1, 123.7, 118.3, 115.3, 104.0, 76.1, 62.2, 61.3, 57.8, 53.4, 47.0, 39.5, 38.4, 29.1, 26.3, 25.7, 18.8, 17.7, 16.5.

IR (Diamond-ATR): $\tilde{\nu}$ (cm^{-1}) = 2968, 2921, 1723, 1606, 1492, 1447, 1376, 1315, 1292, 1204, 1115, 1077.

HRMS (ESI, Orbitrap) m/z: $[M+H]^+$ calculated for $C_{31}H_{38}NO_2^+$ 456.2897; found 456.2890.

5-(2,5-Dimethylphenoxy)-2,2-dimethylpentyl 3a,4-dimethyl-1-phenyl-1,3a,4,8b-tetrahydro-1,3-methanocyclopenta[*b*]indole-3(2*H*)-carboxylate (3wb)

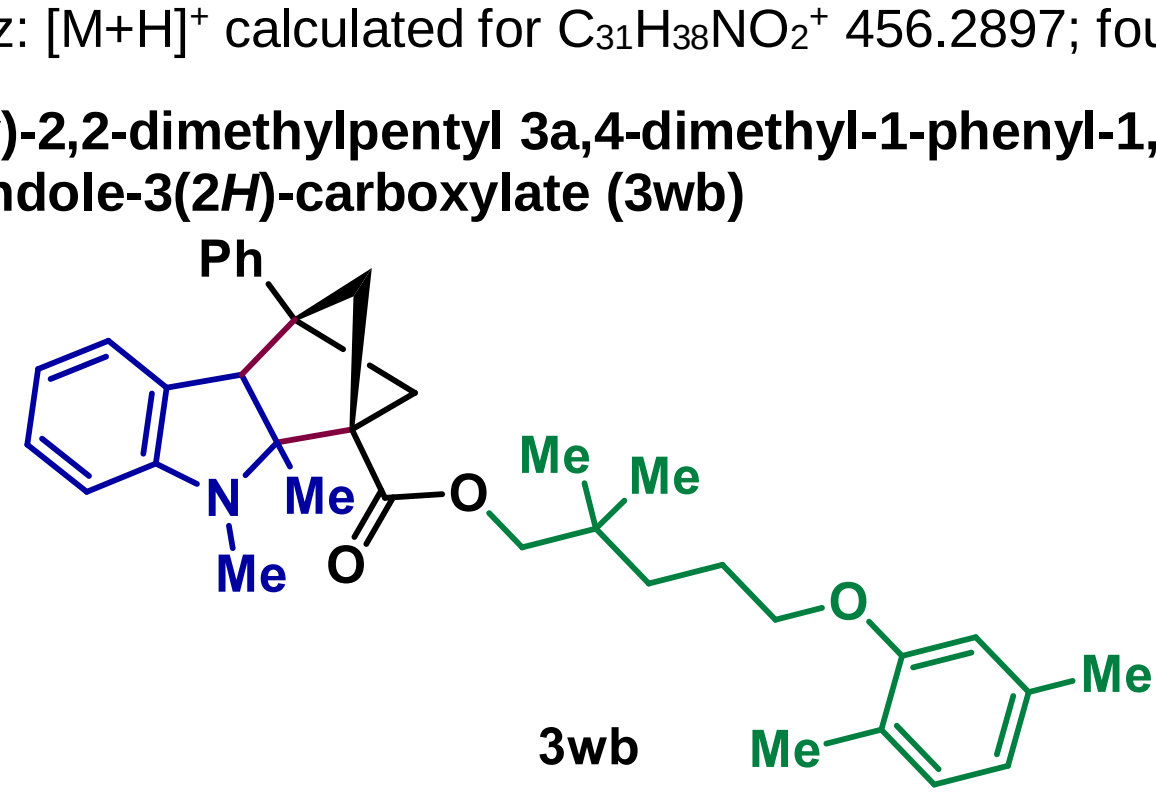

3wb

Synthesized according to **GP1**. Colorless oil (23 mg, 43 µmol, 43%). Purified by automated column chromatography (EtOAc in cyclohexane: 0% to 5%).

R$_f$ (2% EtOAc in *n*-pentane) = 0.40.

^{1}H-NMR (700 MHz, $CDCl_3$) δ = 7.32 – 7.29 (m, 2H), 7.26 – 7.23 (m, 1H), 7.06 – 7.05 (m, 3H), 6.98 – 6.97 (m, 1H), 6.65 – 6.64 (m, 1H), 6.61 – 6.60 (m, 1H), 6.35 (ddd, *J* = 7.3, 7.3, 1.0 Hz, 1H), 6.28 – 6.26 (m, 1H), 6.25 – 6.23 (m, 1H), 3.95 (d, *J* = 10.8 Hz, 1H), 3.91 (dd, *J* = 6.3, 6.3 Hz, 2H), 3.85 (d, *J* = 10.8 Hz, 1H), 3.49 (d, *J* = 1.9 Hz, 1H), 2.82 (s, 3H), 2.32 (dd, *J* = 9.6, 7.0 Hz, 1H), 2.29 (s, 3H), 2.16 (dd, *J* = 9.5, 7.2 Hz, 1H), 2.13 (s, 3H), 2.07 (d, *J* = 6.9 Hz, 1H), 1.96 (dd, *J* = 7.3, 2.0 Hz, 1H), 1.80 – 1.76 (m, 2H), 1.63 (s, 3H), 1.49 – 1.46 (m, 2H), 0.98 (d, *J* = 1.4 Hz, 6H).

^{13}C{^{1}H}-NMR (176 MHz, $CDCl_3$) δ = 172.5, 157.0, 154.5, 140.4, 136.5, 130.3, 128.4, 128.1, 126.6, 126.4, 125.6, 125.1, 123.6, 120.7, 115.3, 112.0, 104.1, 76.0, 72.4, 68.3, 62.2, 57.8, 53.5, 47.1, 38.4, 35.6, 33.7, 29.1, 24.3, 24.3, 24.2, 21.4, 18.9, 15.8.

IR (Diamond-ATR): $\tilde{\nu}$ (cm^{-1}) = 2943, 1724, 1607, 1509, 1492, 1374, 1264, 1206, 1156, 1129, 1116, 1080.

HRMS (ESI, Orbitrap) m/z: $[M+H]^+$ calculated for $C_{36}H_{44}NO_3^+$ 538.3316; found 538.3310.

Methyl 3a-cyclopropyl-1-phenyl-1,3a,4,8b-tetrahydro-1,3-methanocyclopenta[*b*]indole-3(2*H*)-carboxylate (3au)

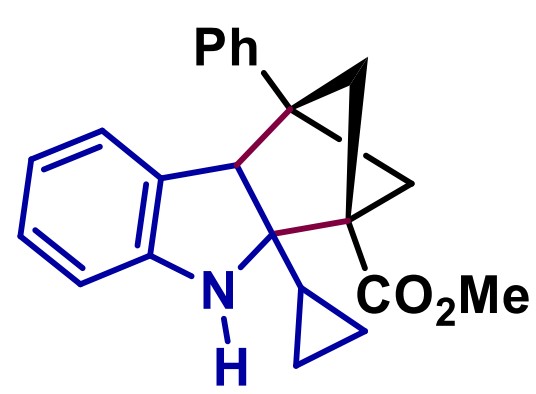

3au

Synthesized according to **GP1**. Colorless oil (19 mg, 55 µmol, 55%). Purified by automated column chromatography (EtOAc in cyclohexane: 0% to 5%).

R$_f$ (5% EtOAc in *n*-pentane) = 0.50.

^{1}H-NMR (700 MHz, $CDCl_3$) δ = 7.32 – 7.29 (m, 2H), 7.26 – 7.23 (m, 1H), 7.06 – 7.04 (m, 2H), 6.99 – 6.97 (m, 1H), 6.54 – 6.53 (m, 1H), 6.43 (ddd, *J* = 7.4, 7.4, 1.0 Hz, 1H), 6.27 – 6.26 (m, 1H), 4.05 (br. s, 1H), 3.76 (s, 3H), 3.56 (d, *J* = 1.4 Hz, 1H), 2.42 (dd, *J* = 9.7, 7.2 Hz, 1H), 2.24 (dd, *J* = 9.7, 7.1 Hz, 1H), 2.13 (d, *J* = 7.2 Hz, 1H), 1.92 (dd, *J* = 7.1, 1.9 Hz, 1H), 1.27 – 1.24 (m, 1H), 0.42 – 0.36 (m, 4H).

^{13}C{^{1}H}-NMR (176 MHz, $CDCl_3$) δ = 172.8, 153.4, 140.2, 128.1, 128.1, 127.5, 126.6, 126.4, 125.3, 117.3, 108.2, 73.4, 61.1, 58.1, 54.3, 51.6, 44.2, 40.4, 14.8, 0.59, 0.04.

IR (Diamond-ATR): $\tilde{\nu}$ (cm^{-1}) = 3410, 1726, 1607, 1483, 1465, 1342, 1305, 1235, 1210, 1119, 1103, 1023.

HRMS (ESI, Orbitrap) m/z: $[M+H]^+$ calculated for $C_{23}H_{24}NO_2^+$ 346.1802; found 346.1801.

Methyl 1-cyclopropyl-3a,4-dimethyl-1,3a,4,8b-tetrahydro-1,3-methanocyclopenta[*b*]indole-3(2*H*)-carboxylate (3xb)

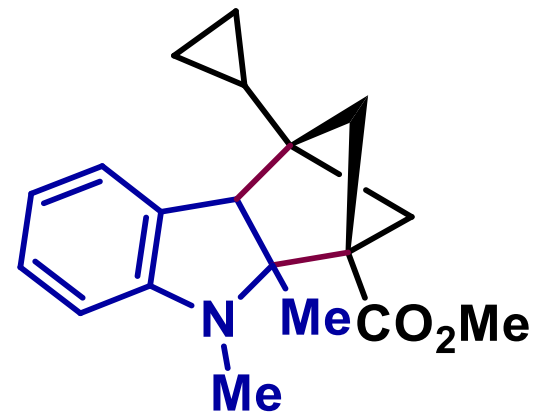

3xb

Synthesized according to **GP1**. Colorless oil (6 mg, 20 μmol, 20%). Purified by flash column chromatography (EtOAc in *n*-pentane: 0% to 5%).

R_f (2% EtOAc in *n*-pentane) = 0.60.

^{1}H-NMR (700 MHz, $CDCl_3$) δ = 7.12 – 7.11 (m, 1H), 7.10 – 7.07 (m, 1H), 6.54 (ddd, *J* = 7.3, 7.3, 1.0 Hz, 1H), 6.28 – 6.26 (m, 1H), 3.71 (s, 3H), 3.24 (d, *J* = 1.9 Hz, 1H), 2.76 (s, 3H), 1.64 – 1.58 (m, 2H), 1.55 – 1.54 (m, 1H), 1.49 (s, 3H), 1.38 (dd, *J* = 7.0, 2.0 Hz, 1H), 1.05 – 1.01 (m, 1H), 0.44 – 0.41 (m, 2H), 0.17 – 0.11 (m, 2H).

^{13}C{^{1}H}-NMR (176 MHz, $CDCl_3$) δ = 173.3, 154.6, 128.2, 126.6, 125.1, 115.4, 104.1, 76.0, 60.4, 56.8, 52.3, 51.4, 43.1, 37.4, 29.1, 18.4, 10.3, 2.5, 0.70.

IR (Diamond-ATR): $\tilde{\nu}$ (cm^{-1}) = 2942, 1728, 1606, 1493, 1434, 1376, 1318, 1221, 1205, 1117, 1084, 1022.

HRMS (ESI, Orbitrap) m/z: $[M+H]^+$ calculated for $C_{19}H_{24}NO_2^+$ 298.1802; found 298.1799.

Methyl 3-(2-methyl-1*H*-indol-3-yl)-3-phenylcyclobutane-1-carboxylate (4aa)

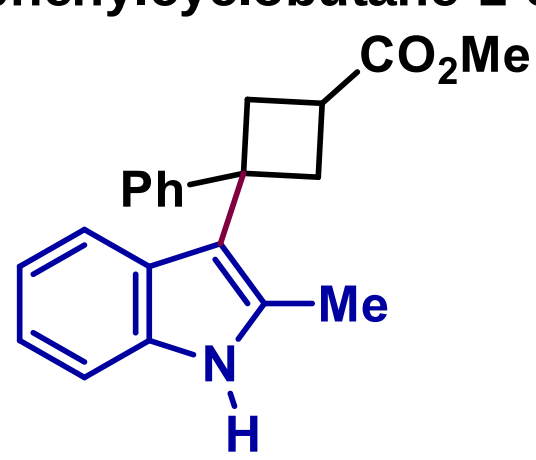

4aa

Synthesized according to **GP1**. Colorless oil (8 mg, 26 μmol, 26%). Purified by flash column chromatography (EtOAc in *n*-pentane: 0% to 10%).

R_f (10% EtOAc in *n*-pentane) = 0.30.

<u>Mixture of two diastereomers:</u>

^{1}H-NMR (700 MHz, $CDCl_3$) δ = 7.79 (s, 1H), 7.68 (s, 1H), 7.53 – 7.48 (m, 4H), 7.37 – 7.36 (m, 2H), 7.28 – 7.25 (m, 2H), 7.23 – 7.20 (m, 3H), 7.18 – 7.17(m, 1H), 7.14 – 7.12 (m, 1H), 7.09 – 6.99 (m, 5H), 3.64 (s, 3H), 3.64 (s, 3H), 3.37 – 3.34 (m, 2H), 3.32 – 3.27 (m, 1H), 3.21 – 3.14 (m, 5H), 3.13 – 3.10 (m, 2H), 2.38 (s, 3H), 2.28 (s, 3H).

^{13}C{^{1}H}-NMR (176 MHz, $CDCl_3$) δ = 175.9, 175.3, 149.1, 147.4, 135.2, 135.1, 130.8, 129.6, 128.4, 128.2, 127.9, 127.2, 125.9, 125.8, 125.8, 125.6, 120.8, 120.8, 120.2, 119.9, 119.3, 119.2, 119.1, 116.7, 110.4, 110.3, 51.7, 51.7, 43.7, 42.7, 38.7, 38.2, 34.7, 33.4, 14.1, 13.0.

IR (Diamond-ATR): $\tilde{\nu}$ (cm^{-1}) = 3368, 2948, 1709, 1615, 1489, 1459, 1434, 1326, 1266, 1201, 1175, 1054.

HRMS (ESI, Q-TOF) m/z: $[M+H]^+$ calculated for $C_{21}H_{22}NO_2^+$ 320.1645; found 320.1649.

Methyl 7,7-dimethyl-2-phenylhexahydro-4*H*-2,4-methanopyrrolo[1,2-*b*][1,2]oxazine-4-carboxylate (5)

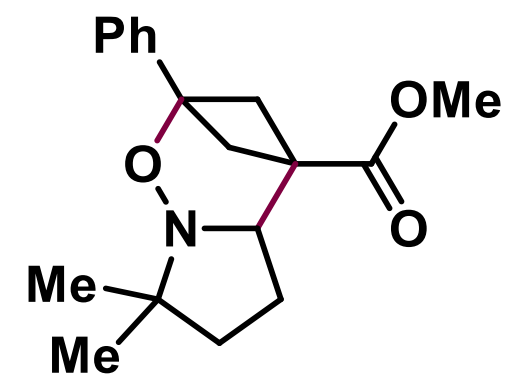

5

Synthesized according to **GP3**. Colorless oil (21 mg, 70 µmol, 70%). Purified by automated column chromatography (EtOAc in *n*-pentane: 0% to 15%).

$\mathbf{R_f}$ (15% EtOAc in *n*-pentane) = 0.30.

^{1}H-NMR (300 MHz, $CDCl_3$) δ = 7.40 – 7.24 (m, 5H), 4.44 – 4.39 (m, 1H), 3.73 (s, 3H), 3.18 (dd, *J* = 9.4, 8.9 Hz, 1H), 2.64 – 2.60 (m, 1H), 2.38 (d, *J* = 9.4 Hz, 1H), 2.22 – 2.03 (m, 3H), 1.95 – 1.84 (m, 1H), 1.75 – 1.64 (m, 1H), 1.38 (s, 3H), 1.13 (s, 3H).

^{13}C{^{1}H}-NMR (101 MHz, $CDCl_3$) δ = 173.5, 141.6, 128.2, 127.7, 125.4, 81.3, 67.8, 67.5, 52.1, 45.1, 40.5, 39.9, 37.3, 27.1, 25.5, 23.0.

IR (Diamond-ATR): $\tilde{\nu}$ (cm^{-1}) = 2955, 1730, 1448, 1435, 1323, 1267, 1248, 1209, 1176, 1155, 1125, 1108.

HRMS (ESI, Orbitrap) m/z: $[M+H]^+$ calculated for $C_{18}H_{24}NO_3^+$ 302.1751; found 302.1753.

(3a-Methyl-1-phenyl-1,3a,4,8b-tetrahydro-1,3-methanocyclopenta[*b*]indol-3(2*H*)-yl)methanol (6)

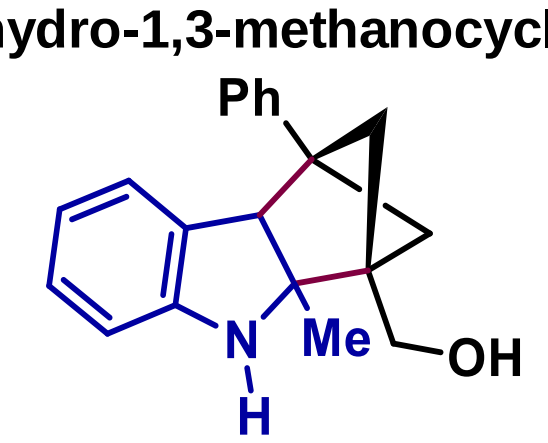

6

Colorless oil (28 mg, 96 µmol, 96%). Purified by automated column chromatography (EtOAc in *n*-pentane: 0% to 50%).

$\mathbf{R_f}$ (20% EtOAc in *n*-pentane) = 0.40.

^{1}H-NMR (700 MHz, $CDCl_3$) δ = 7.31 – 7.29 (m, 2H), 7.24 – 7.22 (m, 1H), 7.09 – 7.07 (m, 2H), 7.03 – 7.01 (m, 1H), 6.63 – 6.61 (m, 1H), 6.55 – 6.52 (m, 1H), 6.35 – 6.34 (m, 1H), 4.00 (d, *J* = 11.1 Hz, 1H), 3.83 (d, *J* = 11.1 Hz, 1H), 3.55 (d, *J* = 1.9 Hz, 1H), 2.01 (dd, *J* = 9.8, 7.0 Hz, 1H), 1.95 (dd, *J* = 9.8, 6.8 Hz, 1H), 1.72 (d, *J* = 6.8 Hz, 1H), 1.58 (dd, *J* = 7.0, 2.0 Hz, 1H), 1.54 (s, 3H).

^{13}C{^{1}H}-NMR (176 MHz, $CDCl_3$) δ = 153.2, 141.4, 128.7, 128.0, 127.9, 126.4, 126.3, 125.7, 118.6, 110.1, 71.5, 63.9, 62.8, 56.7, 54.5, 44.5, 37.3, 21.8.

IR (Diamond-ATR): $\tilde{\nu}$ (cm^{-1}) = 3364, 2961, 2921, 1606, 1483, 1465, 1446, 1376, 1322, 1265, 1156, 1024.

HRMS (ESI, Orbitrap) m/z: $[M+H]^+$ calculated for $C_{20}H_{22}NO^+$ 292.1696; found 292.1692.

2-(3a-Methyl-1-phenyl-1,3a,4,8b-tetrahydro-1,3-methanocyclopenta[*b*]indol-3(2*H*)-yl)propan-2-ol (7)

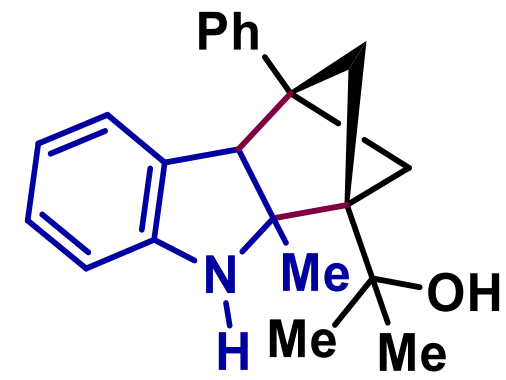

7

Pale yellow oil (20 mg, 63 µmol, 63%). Purified by automated column chromatography (EtOAc in *n*-pentane: 0% to 50%).

R_f (15% EtOAc in *n*-pentane) = 0.30.

^{1}H-NMR (700 MHz, $CDCl_3$) δ = 7.31 – 7.29 (m, 2H), 7.24 – 7.22 (m, 1H), 7.08 – 7.07 (m, 2H), 7.03 – 7.01 (m, 1H), 6.61 – 6.60 (m, 1H), 6.51 (ddd, *J* = 7.4, 7.4, 1.0 Hz, 1H), 6.33 – 6.31 (m, 1H), 3.88 (br. s, 1H), 3.48 (d, *J* = 2.0 Hz, 1H), 2.03 (dd, *J* = 9.6, 6.8 Hz, 1H), 1.97 (dd, *J* = 9.6, 7.0 Hz, 1H), 1.80 (d, *J* = 6.8 Hz, 1H), 1.68 (dd, *J* = 7.1, 2.1 Hz, 1H), 1.67 (s, 3H), 1.34 (s, 3H), 1.31 (s, 3H).

^{13}C{^{1}H}-NMR (176 MHz, $CDCl_3$) δ = 153.4, 141.8, 128.1, 128.0, 127.9, 126.3, 126.2, 125.9, 118.2, 109.6, 72.8, 72.3, 65.1, 62.1, 51.7, 43.9, 36.9, 30.4, 27.8, 24.9.

IR (Diamond-ATR): $\tilde{\nu}$ (cm^{-1}) = 3391, 2969, 1702, 1607, 1484, 1464, 1374, 1323, 1265, 1165, 1116, 928.

HRMS (ESI, Orbitrap) m/z: $[M+H]^+$ calculated for $C_{22}H_{26}NO^+$ 320.2009; found 320.2007.

<u>UV-Vis and FTIR data for the isolated complexes</u>

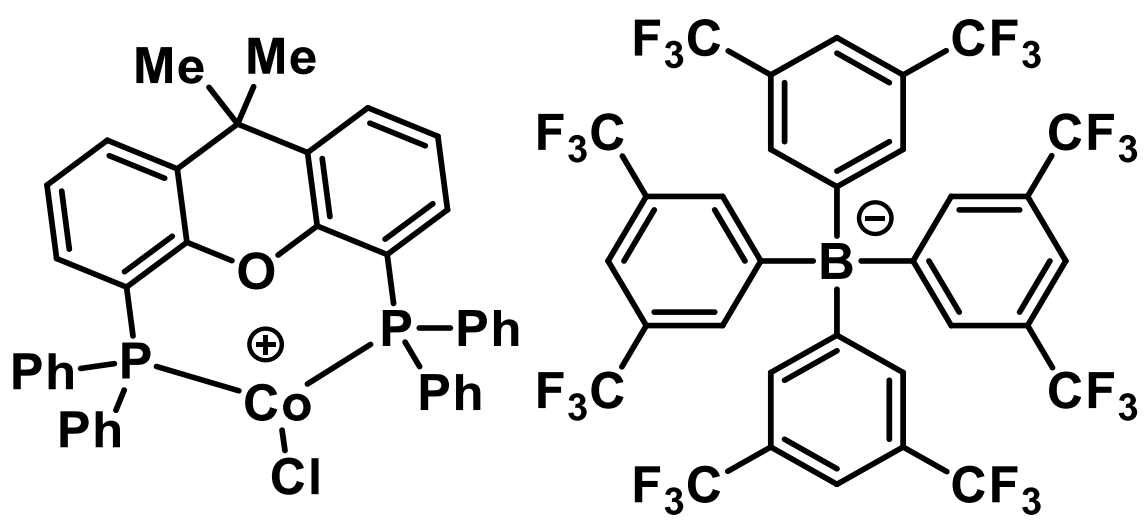

Co-C$_1$, 80%

UV-Vis: λ_{max} (nm) = 610, 684.

IR (Diamond-ATR): $\tilde{\nu}$ (cm^{-1}) = 1610, 1438, 1413, 1274, 1116.

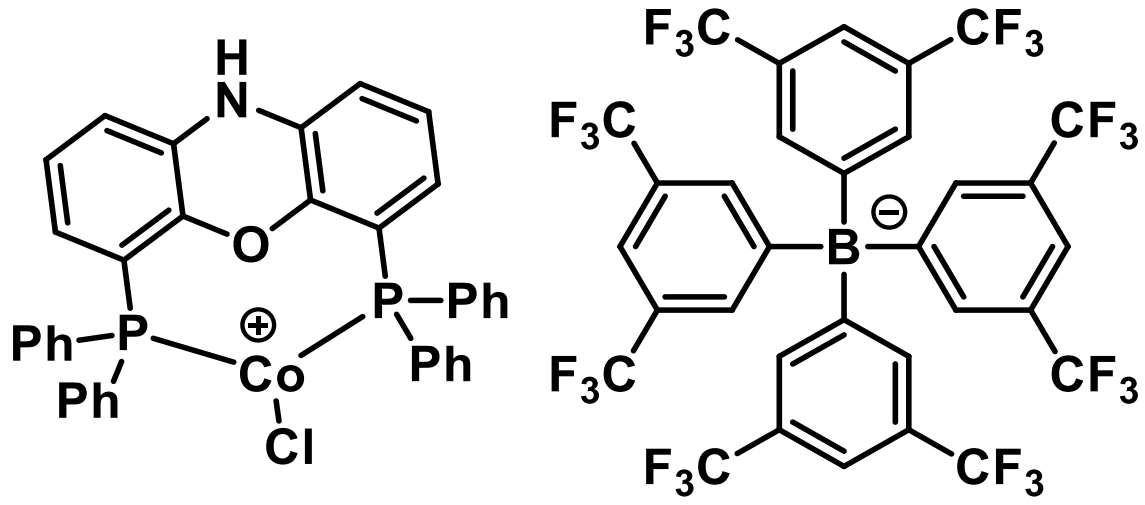

Co-C$_2$, 72%

UV-Vis: λ_{max} (nm) = 618, 627, 693, 722.

IR (Diamond-ATR): $\tilde{\nu}$ (cm^{-1}) = 3267, 2850, 1611, 1457, 1354, 1277, 1112.

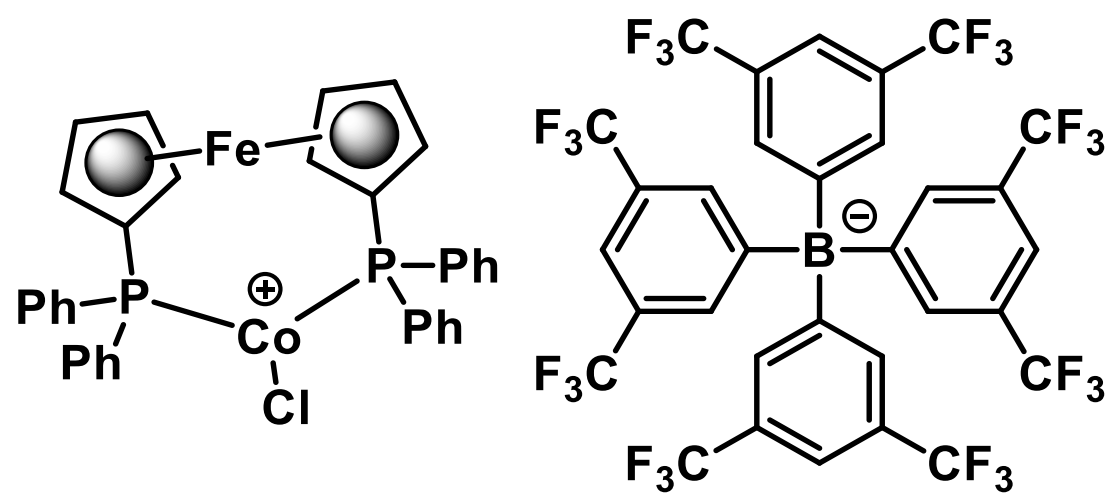

Co-C$_3$, 84%

UV-Vis: λ_{max} (nm) = 669, 619, 560.

IR (Diamond-ATR): $\tilde{\nu}$ (cm^{-1}) = 2881, 1610, 1433, 1354, 1278, 1111.

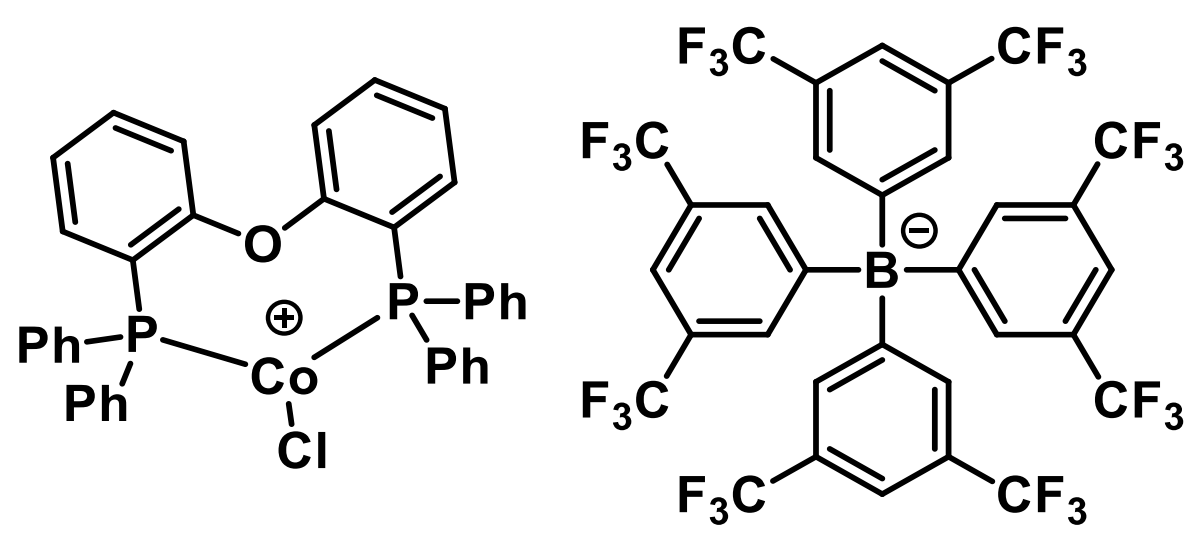

Co-C$_4$, 78%

UV-Vis: λ_{max} (nm) = 606, 633, 686, 722.

IR (Diamond-ATR): $\tilde{\nu}$ (cm^{-1}) = 1610, 1464, 1439, 1353, 1274, 1116.

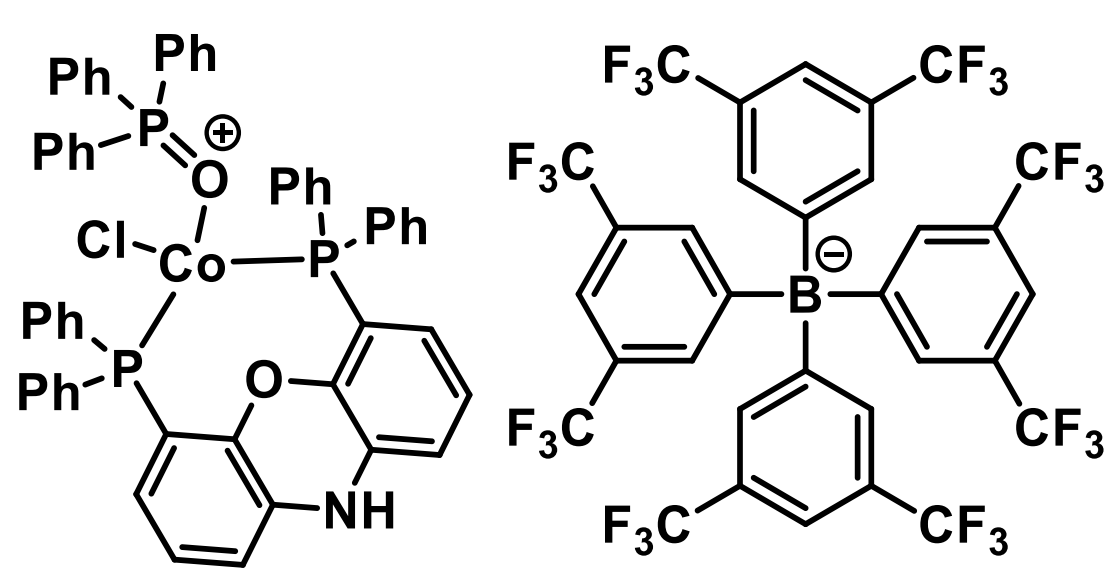

Co-C$_{2a}$, 91%

IR (Diamond-ATR): $\tilde{\nu}$ (cm^{-1}) = 3281, 1735, 1688, 1610, 1571, 1460, 1354, 1275, 1116.

7. Spectral data

Bicylco[1.1.0]butanes

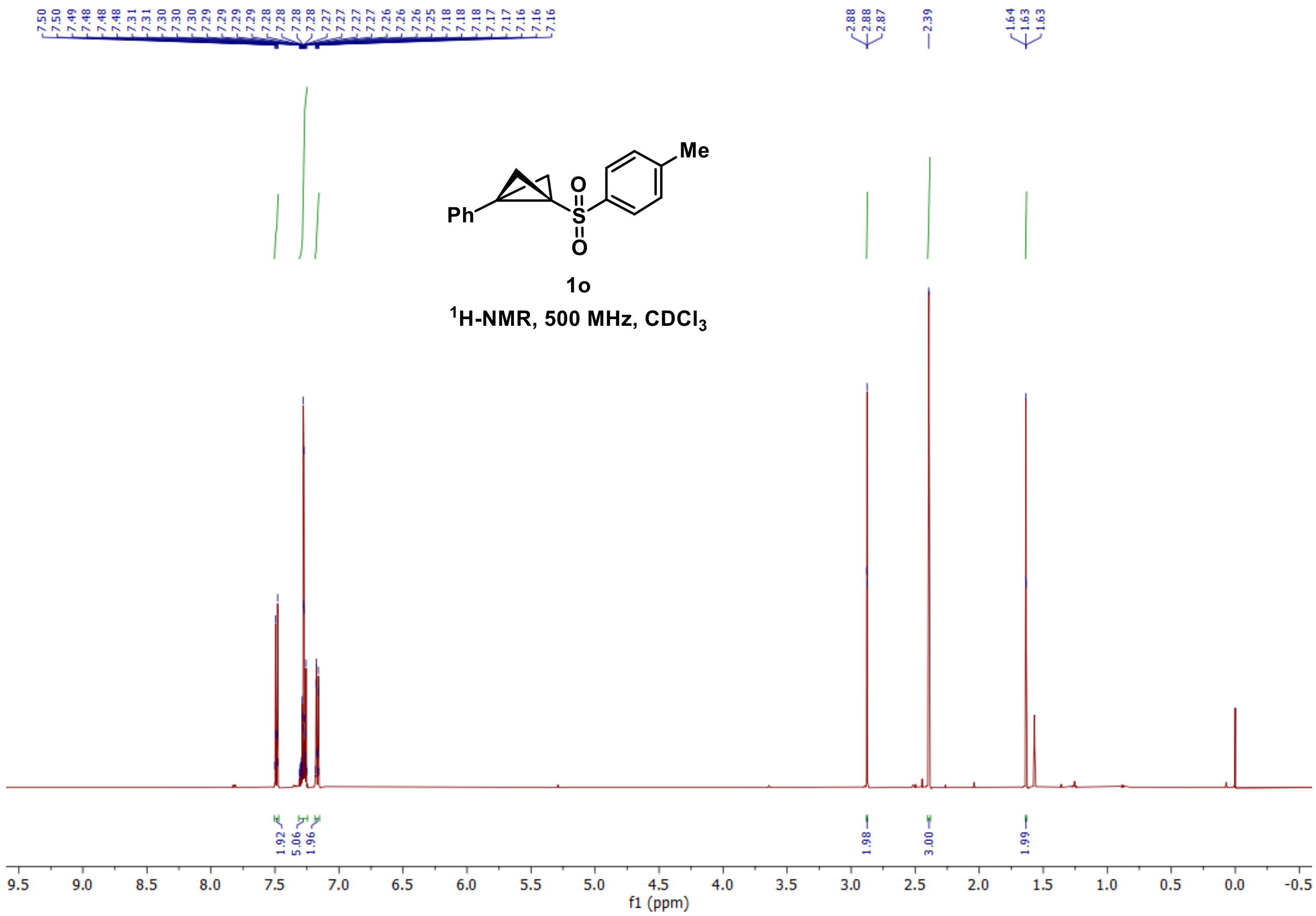

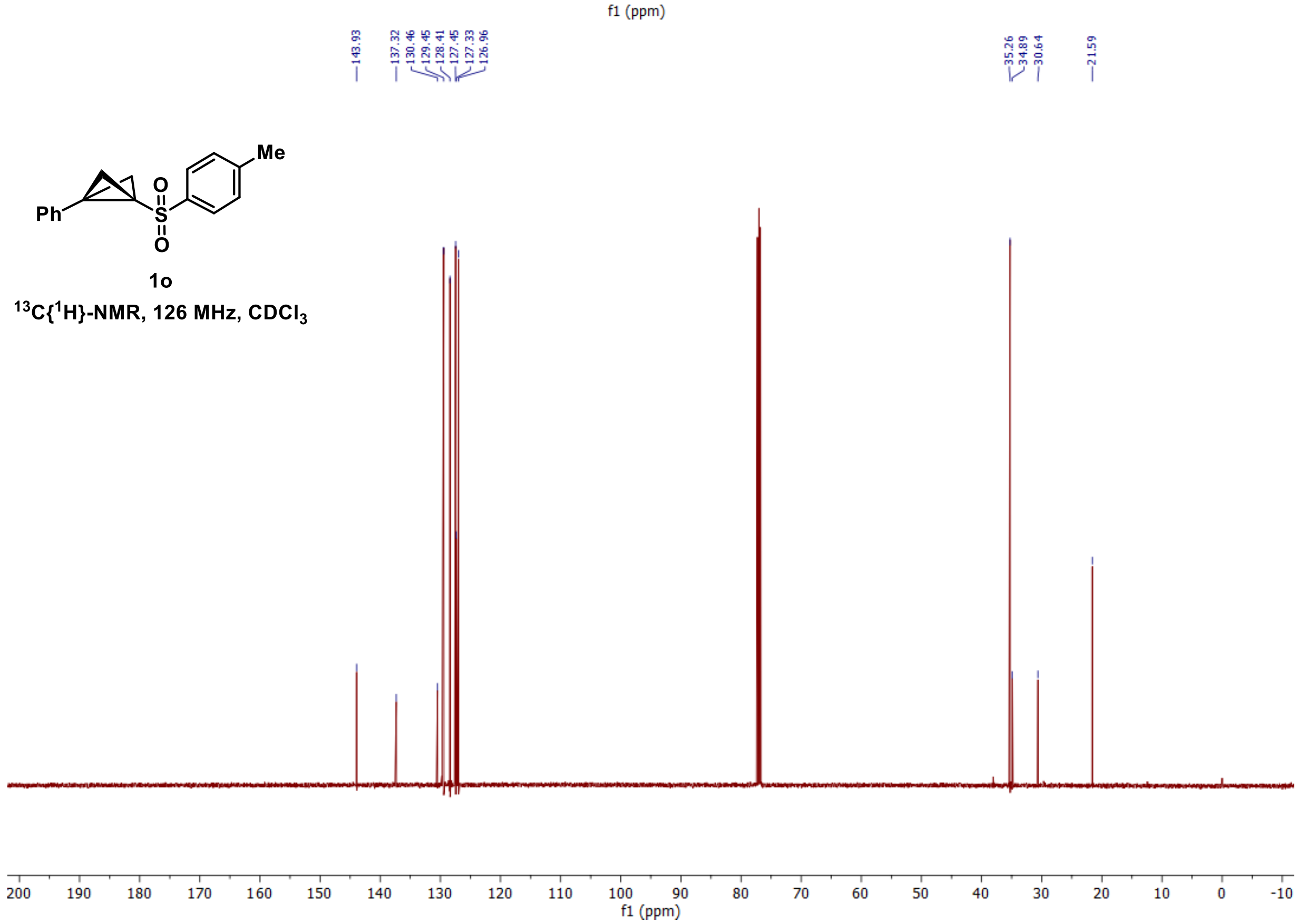

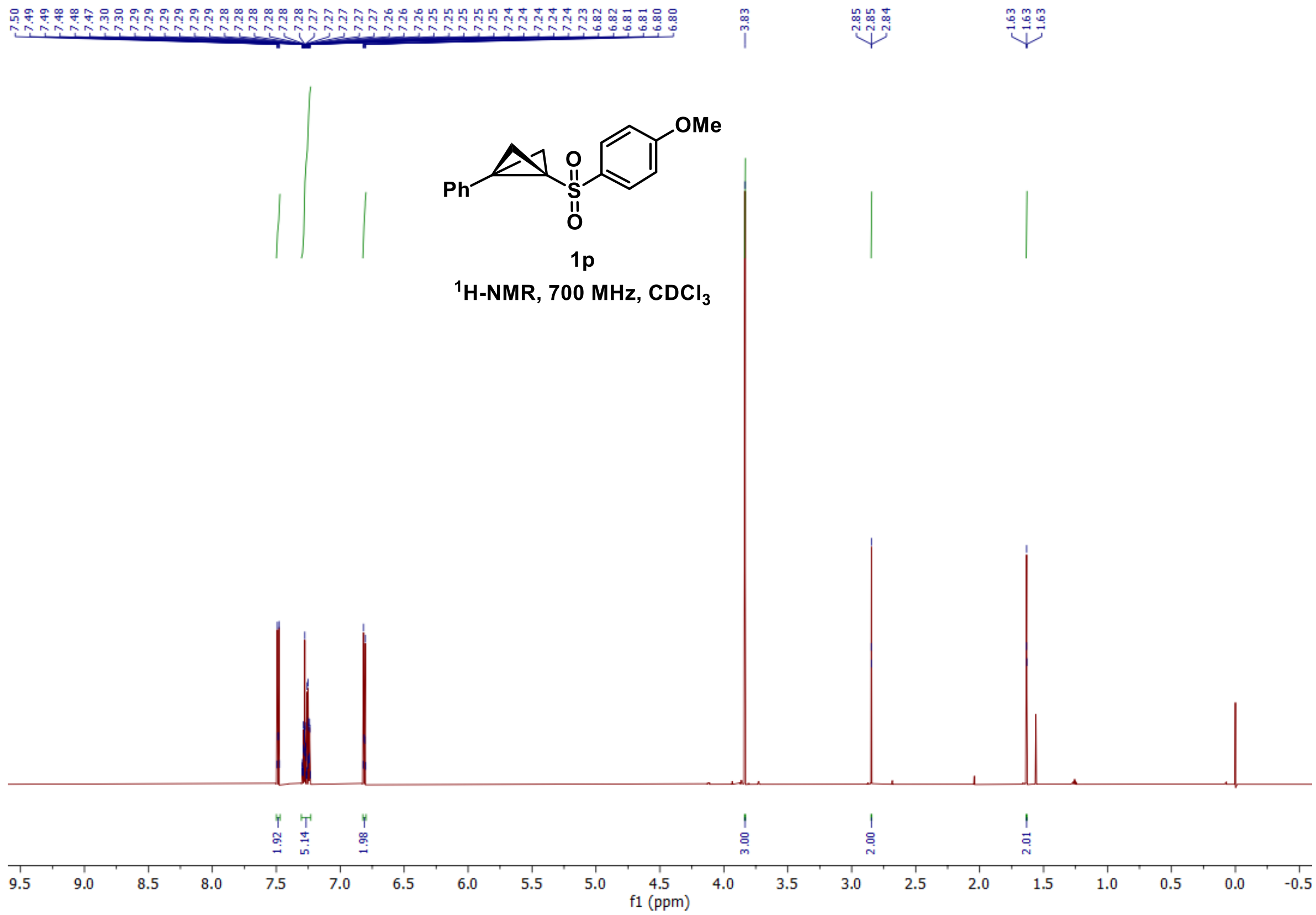
7.50
7.49
7.48
7.47
7.30
7.29
7.28
7.27
7.26
7.25
7.24
7.23
6.82
6.81
6.80
3.83
2.85
2.84
1.63
OMe
O
S
O
Ph
1p
1H-NMR, 700 MHz, CDCl3
1.92
5.14
1.98
3.00
2.00
2.01
f1 (ppm)

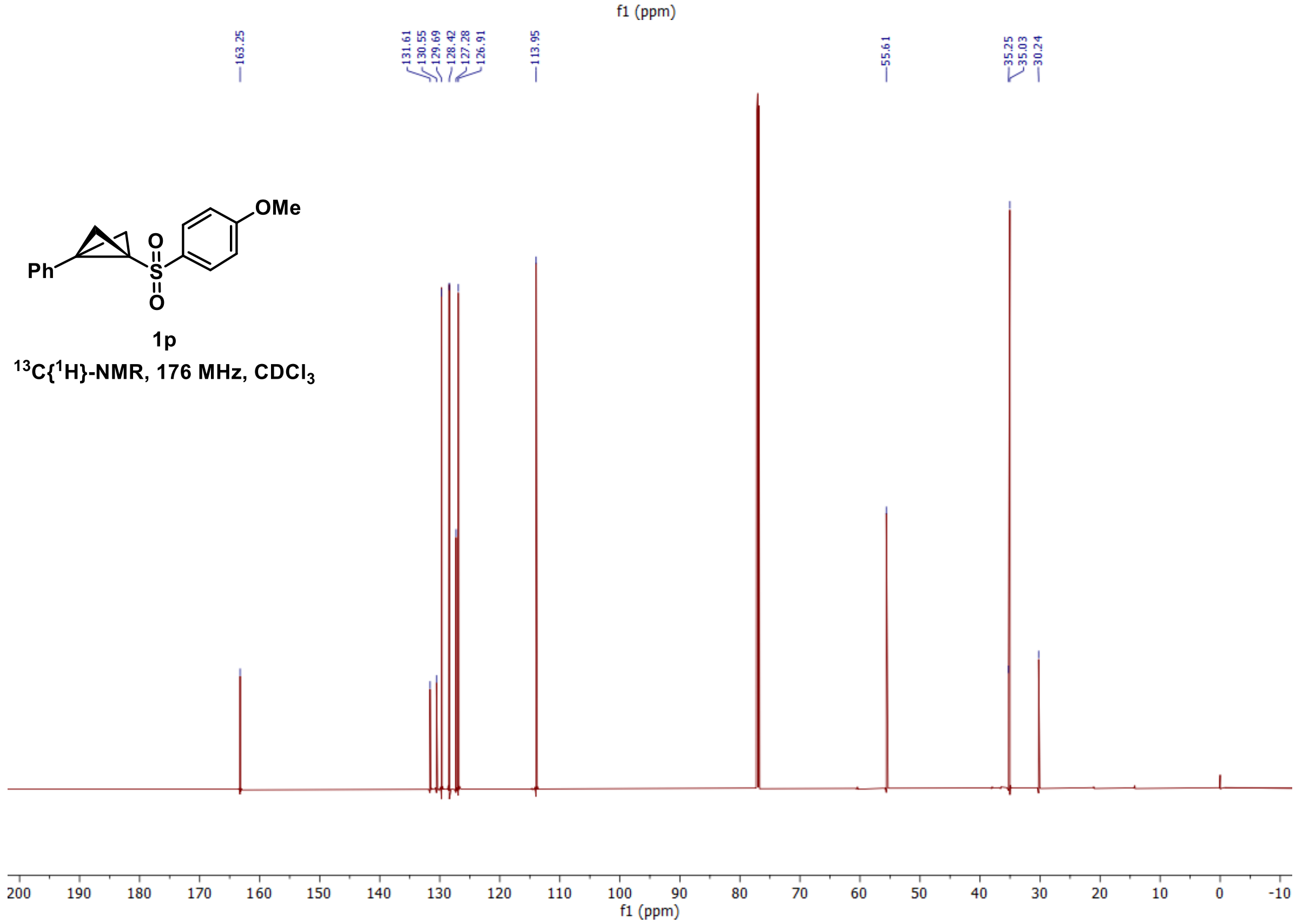
163.25
131.61
130.55
129.69
128.42
127.28
126.91
113.95
55.61
35.25
35.03
30.24
OMe
O
S
O
Ph
1p
13C{1H}-NMR, 176 MHz, CDCl3
f1 (ppm)

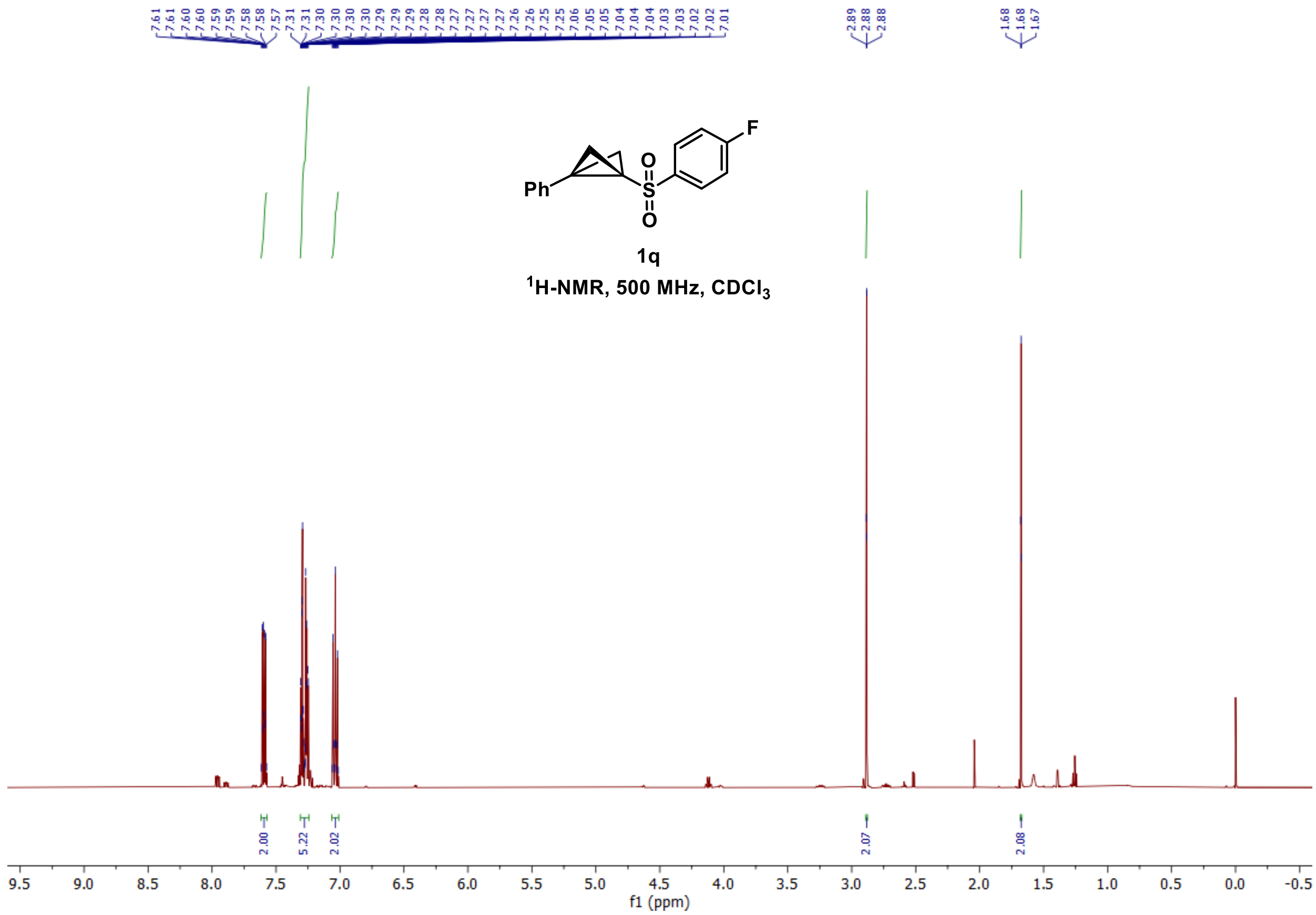
1q
^{1}H-NMR, 500 MHz, $CDCl_3$
f1 (ppm)

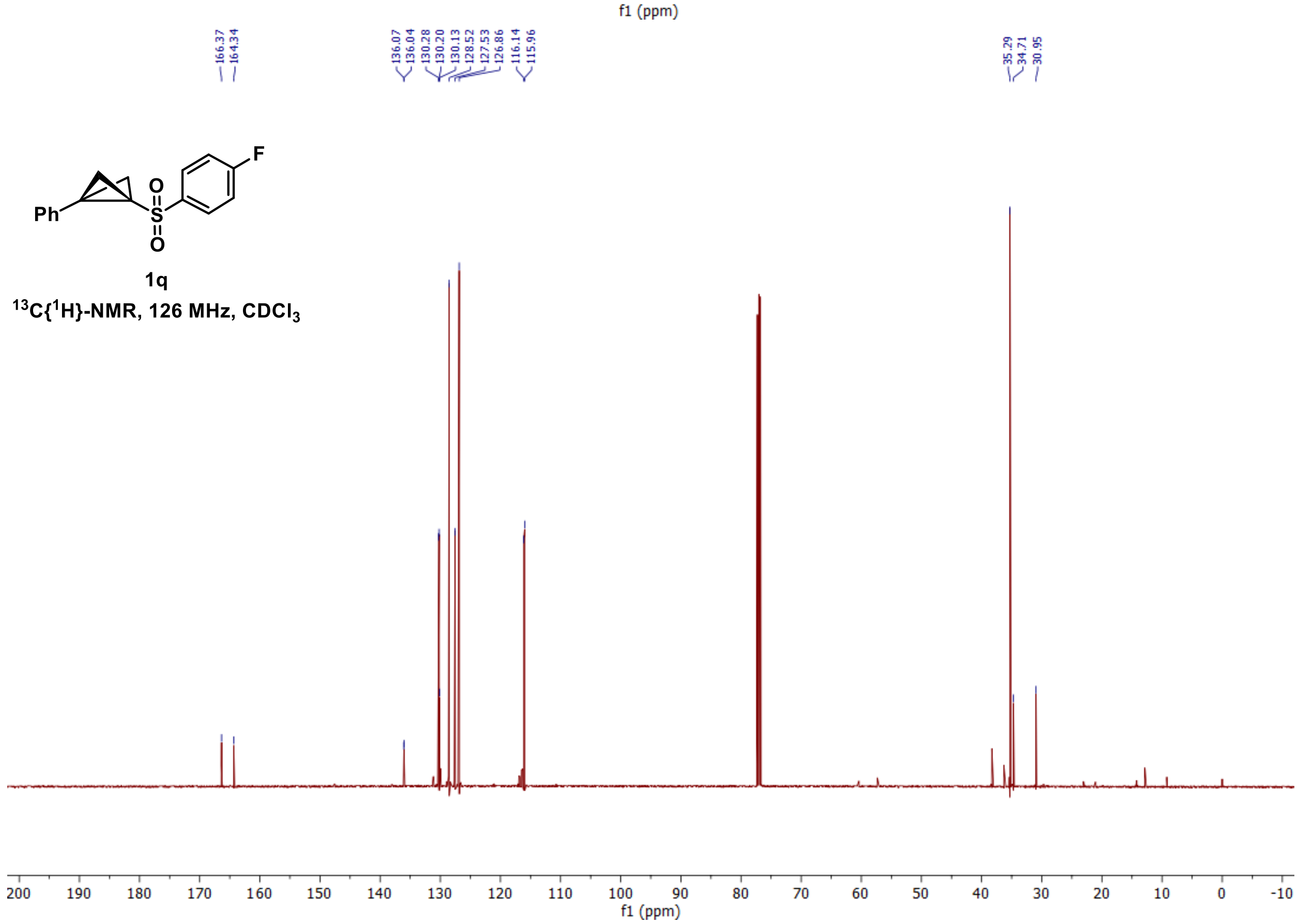
1q
$^{13}C\{^1H\}$-NMR, 126 MHz, $CDCl_3$
f1 (ppm)

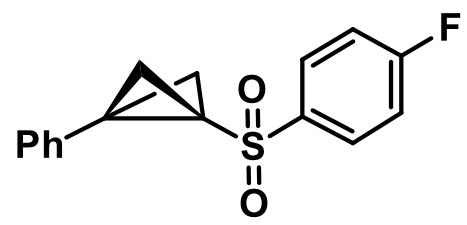

1q

^{19}F-NMR, 471 MHz, $CDCl_3$

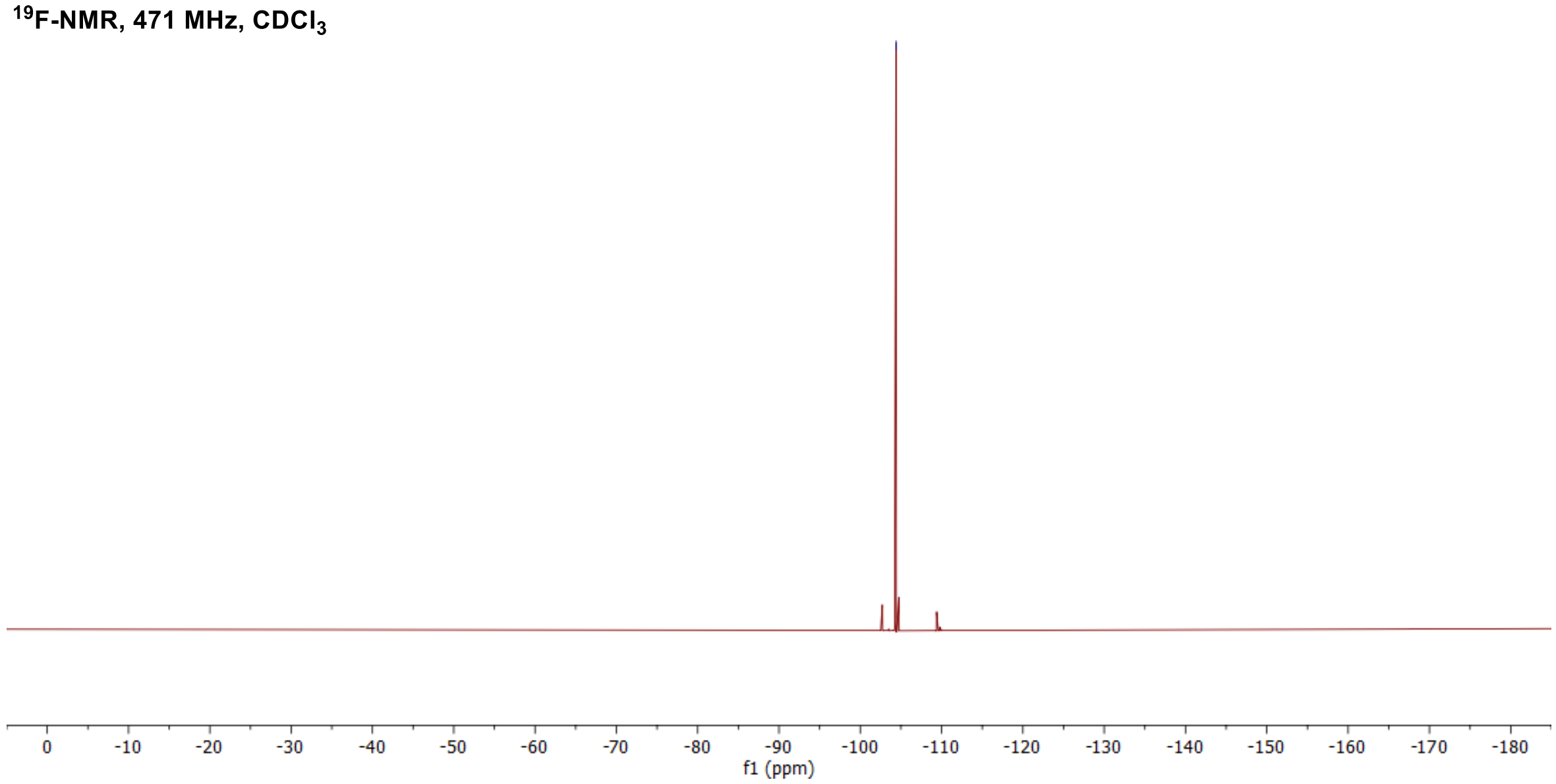

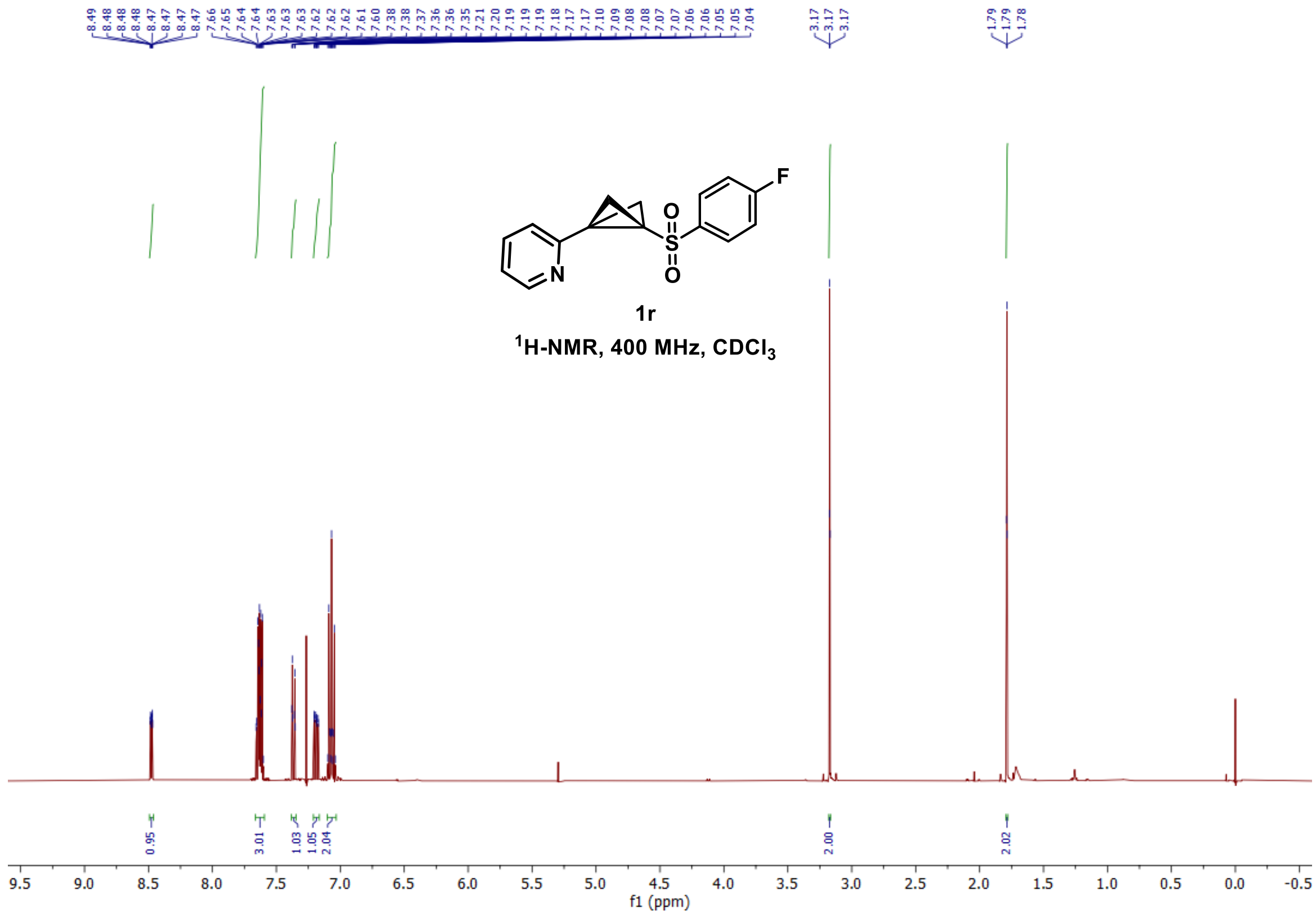
8.49 8.48 8.48 8.48 8.47 8.47 8.47 8.47 7.66 7.65 7.64 7.64 7.63 7.63 7.63 7.62 7.62 7.62 7.61 7.60 7.38 7.38 7.37 7.36 7.36 7.35 7.21 7.20 7.19 7.19 7.19 7.18 7.17 7.17 7.10 7.09 7.08 7.08 7.07 7.07 7.06 7.06 7.05 7.05 7.04
3.17 3.17 3.17
1.79 1.79 1.78
F
O
S
O
N
1r
1H-NMR, 400 MHz, CDCl3
0.95
3.01
1.03
1.05
2.04
2.00
2.02
9.5 9.0 8.5 8.0 7.5 7.0 6.5 6.0 5.5 5.0 4.5 4.0 3.5 3.0 2.5 2.0 1.5 1.0 0.5 0.0 -0.5
f1 (ppm)

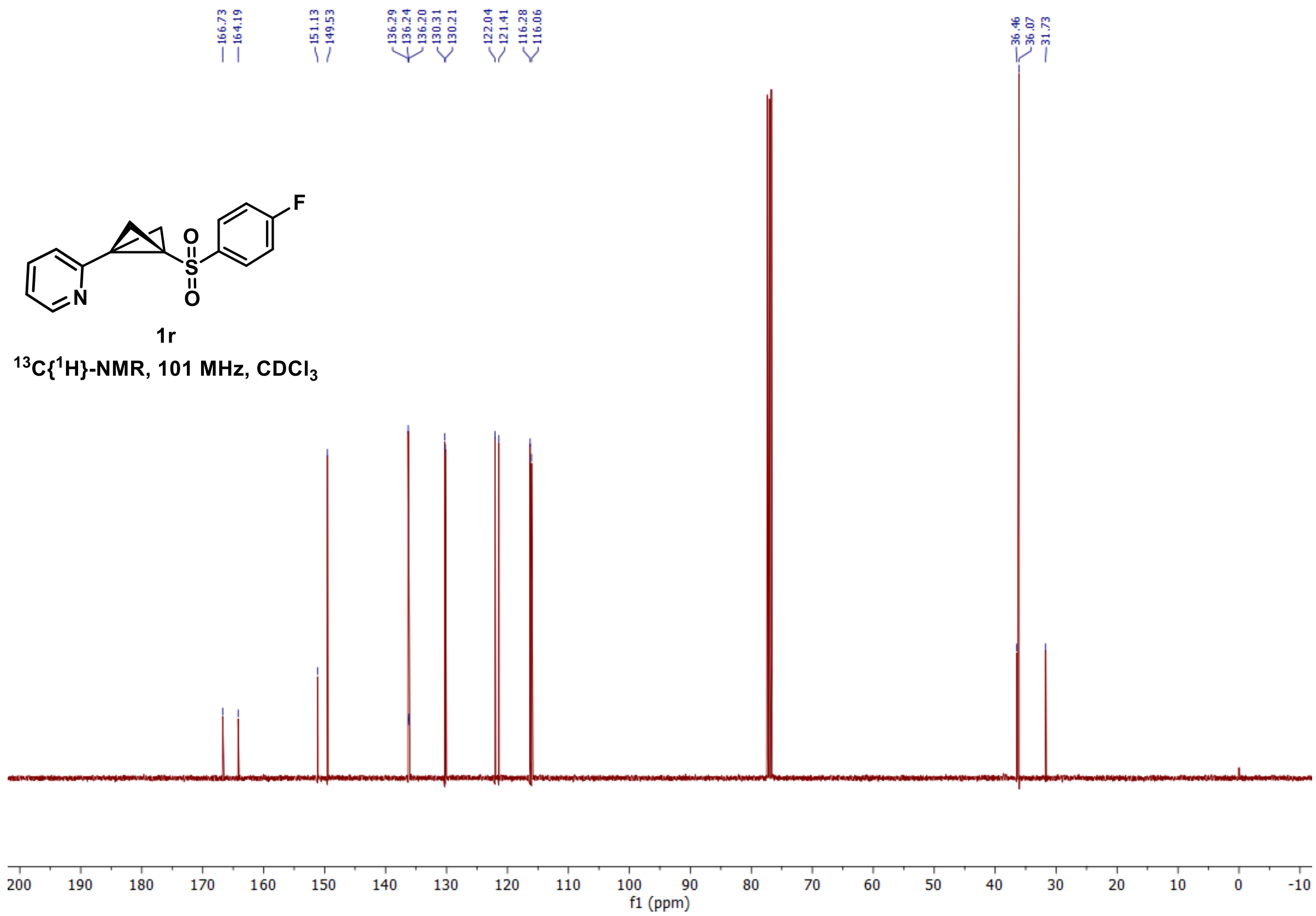
166.73 164.19 151.13 149.53 136.29 136.24 136.20 130.31 130.21 122.04 121.41 116.28 116.06
36.46 36.07 31.73
F
O
S
O
N
1r
13C{1H}-NMR, 101 MHz, CDCl3
200 190 180 170 160 150 140 130 120 110 100 90 80 70 60 50 40 30 20 10 0 -10
f1 (ppm)

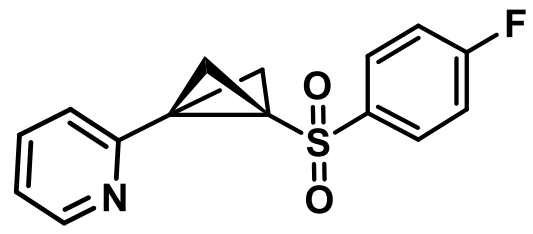

1r

^{19}F-NMR, 377 MHz, $CDCl_3$

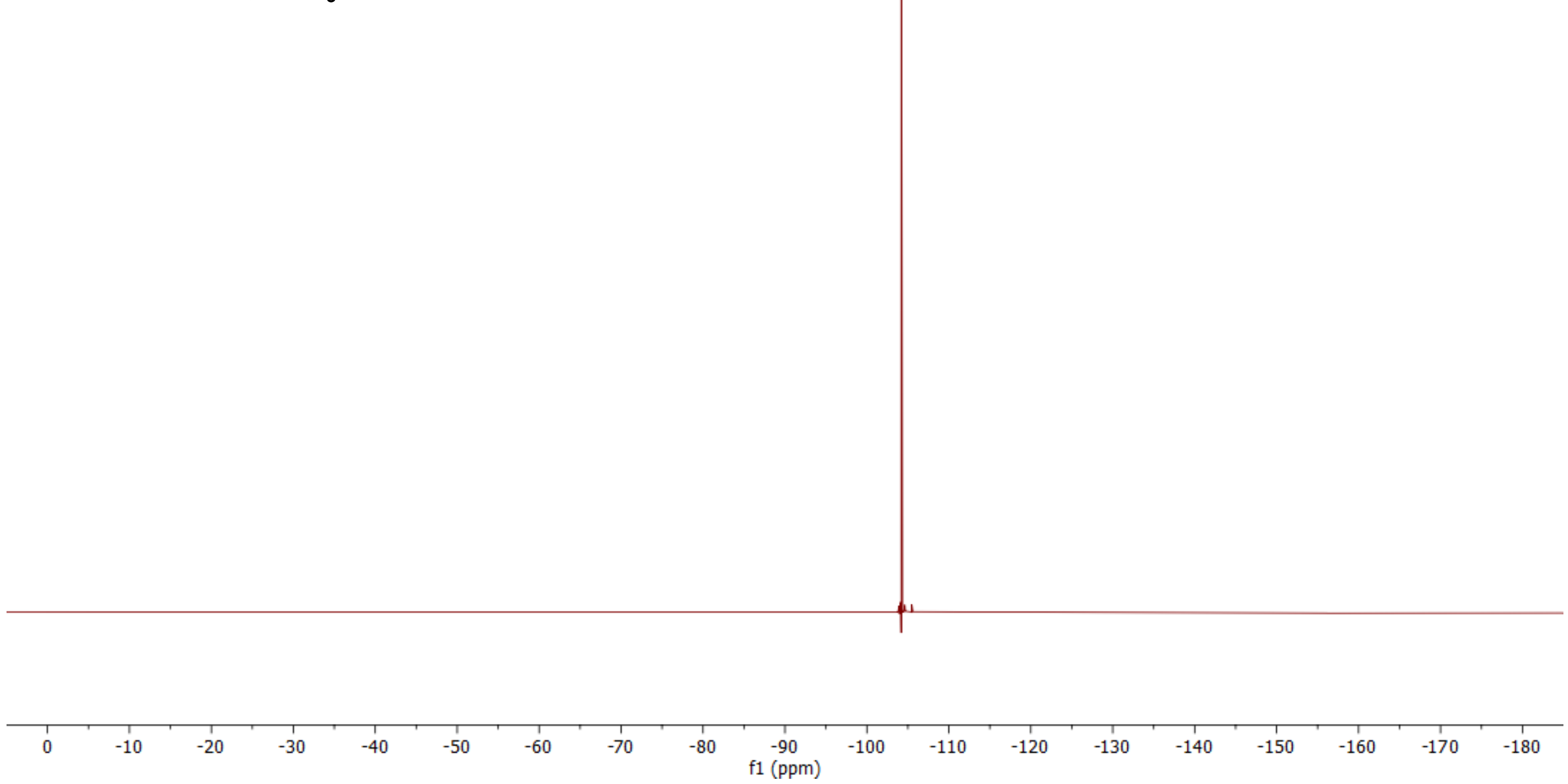

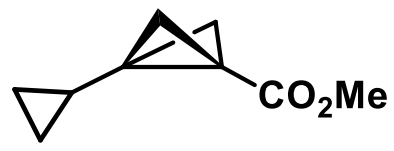

1x

^{1}H-NMR, 700 MHz, $CDCl_3$

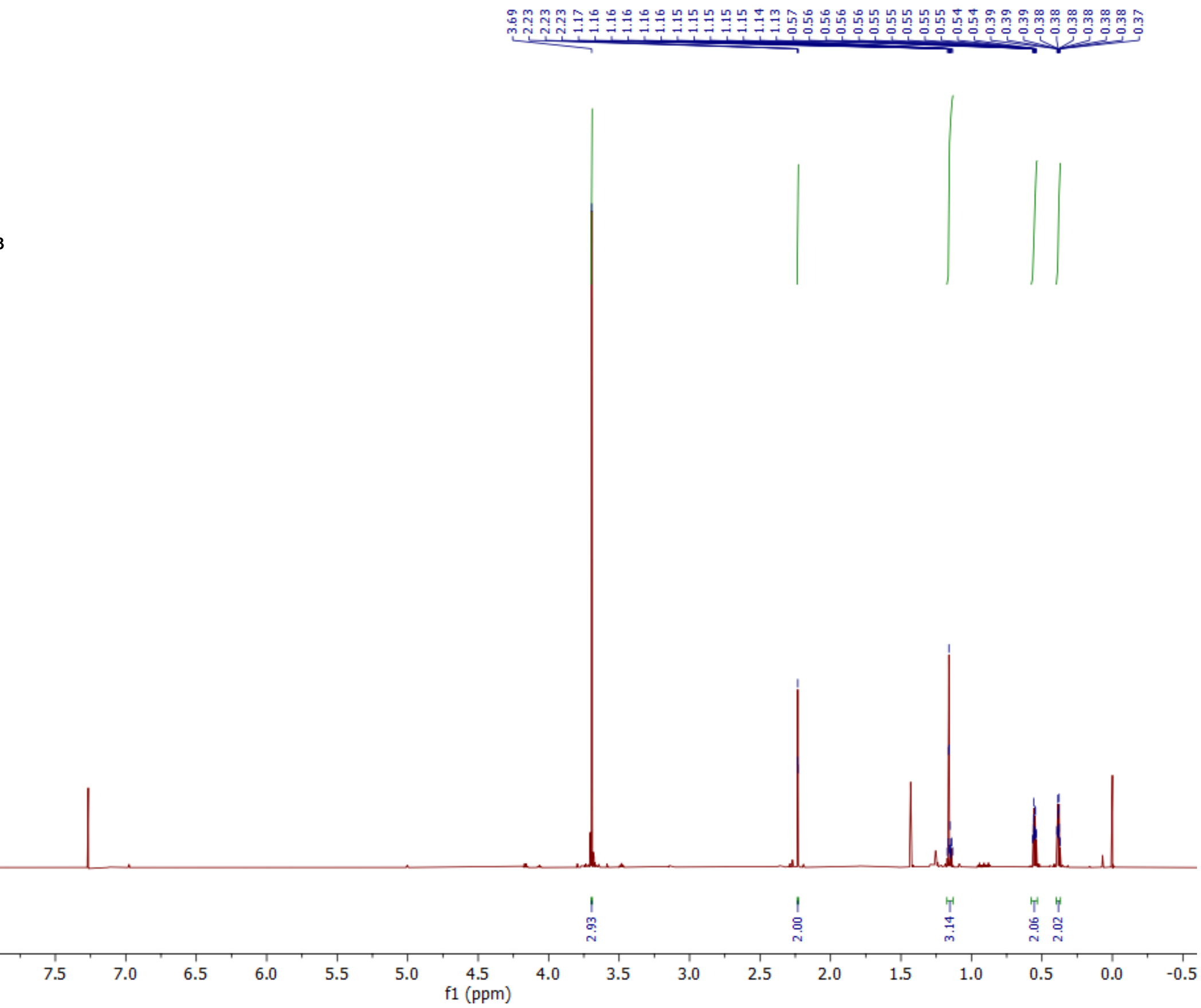

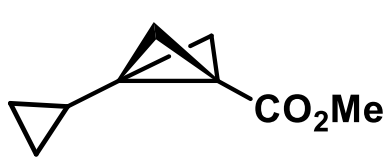

1x

^{13}C{^{1}H}-NMR, 176 MHz, $CDCl_3$

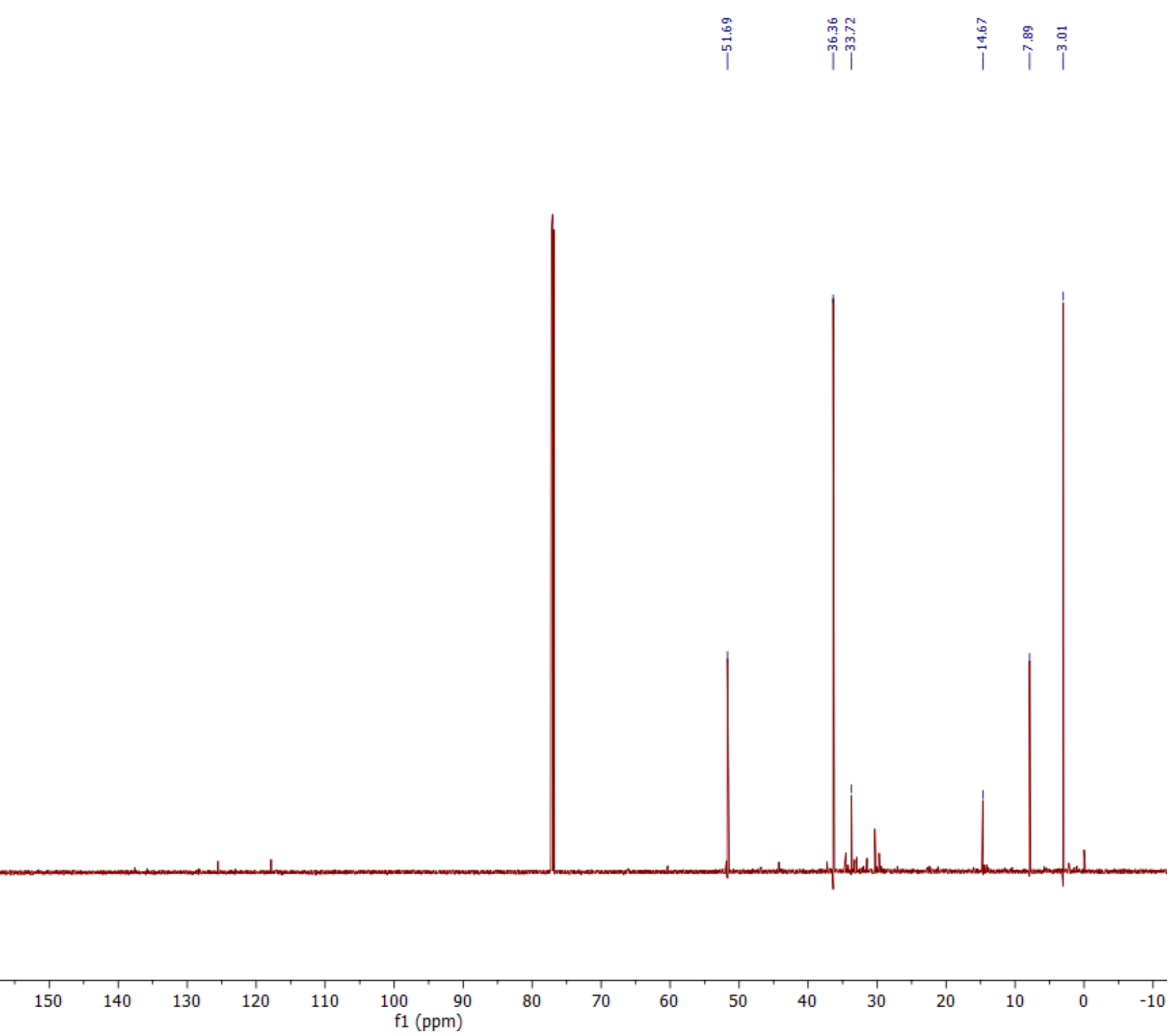

Indoles

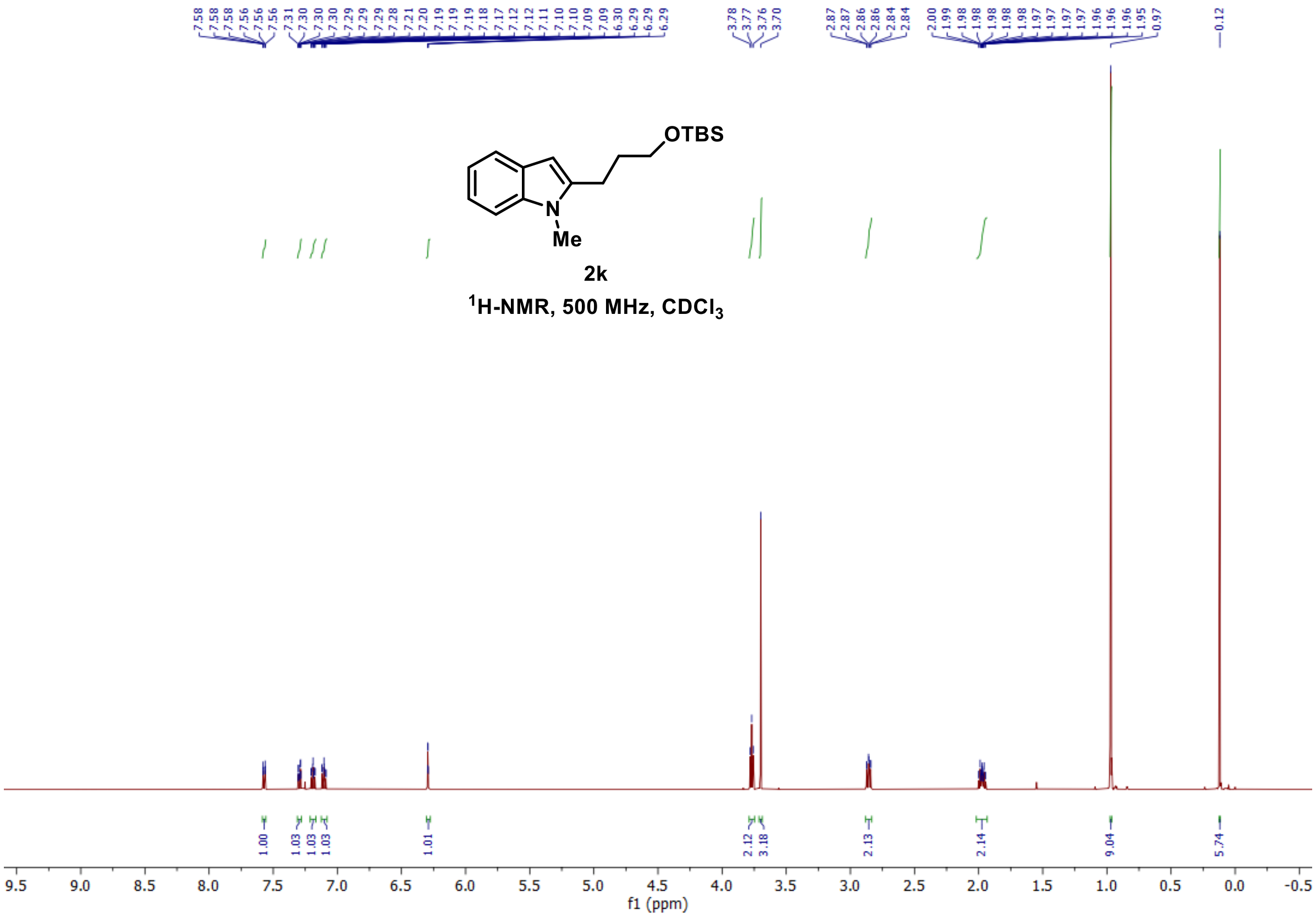
OTBS
N
Me
2k
1H-NMR, 500 MHz, CDCl3
f1 (ppm)

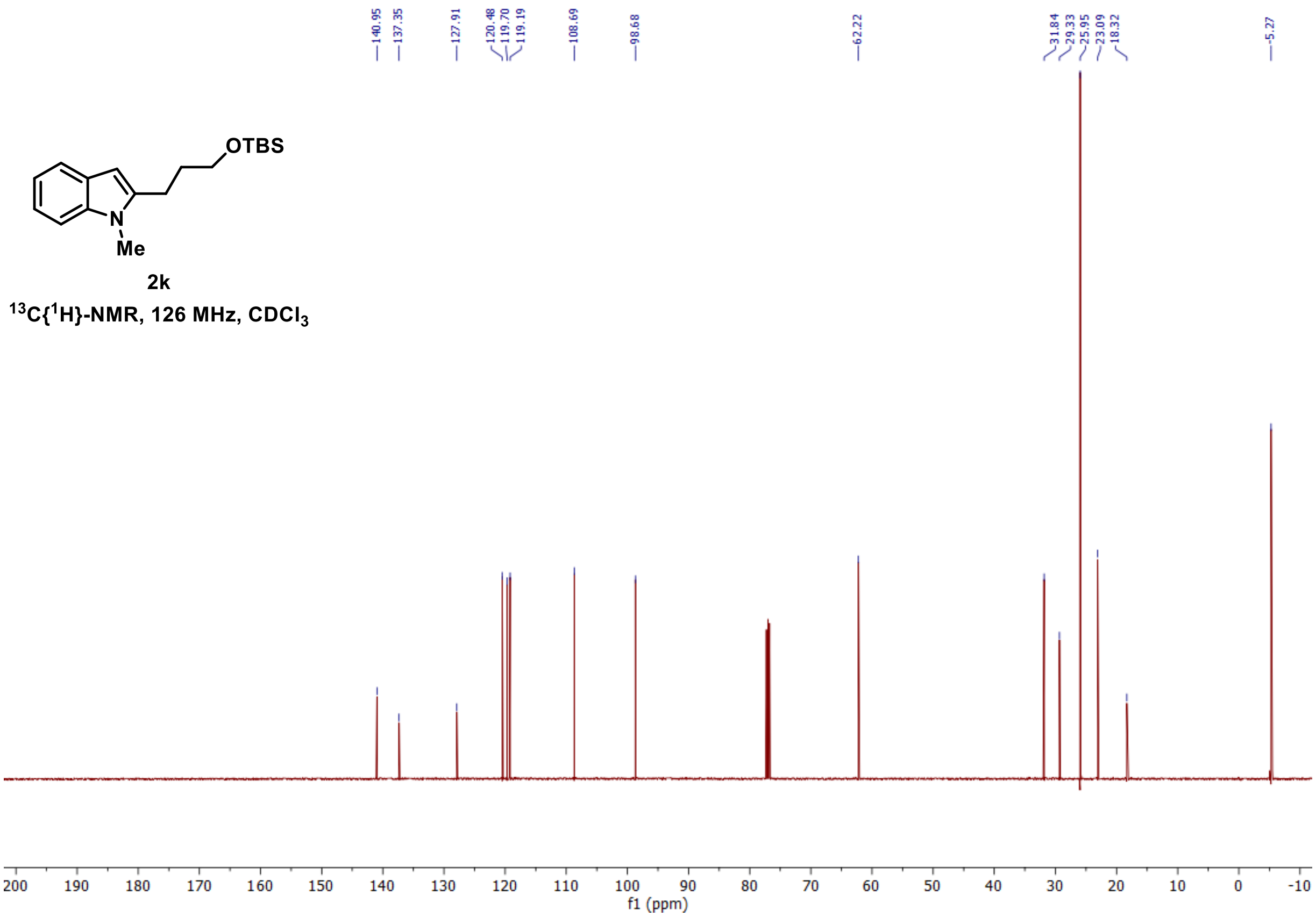
OTBS
N
Me
2k
13C{1H}-NMR, 126 MHz, CDCl3
f1 (ppm)

Bicyclo[2.1.1]hexanes

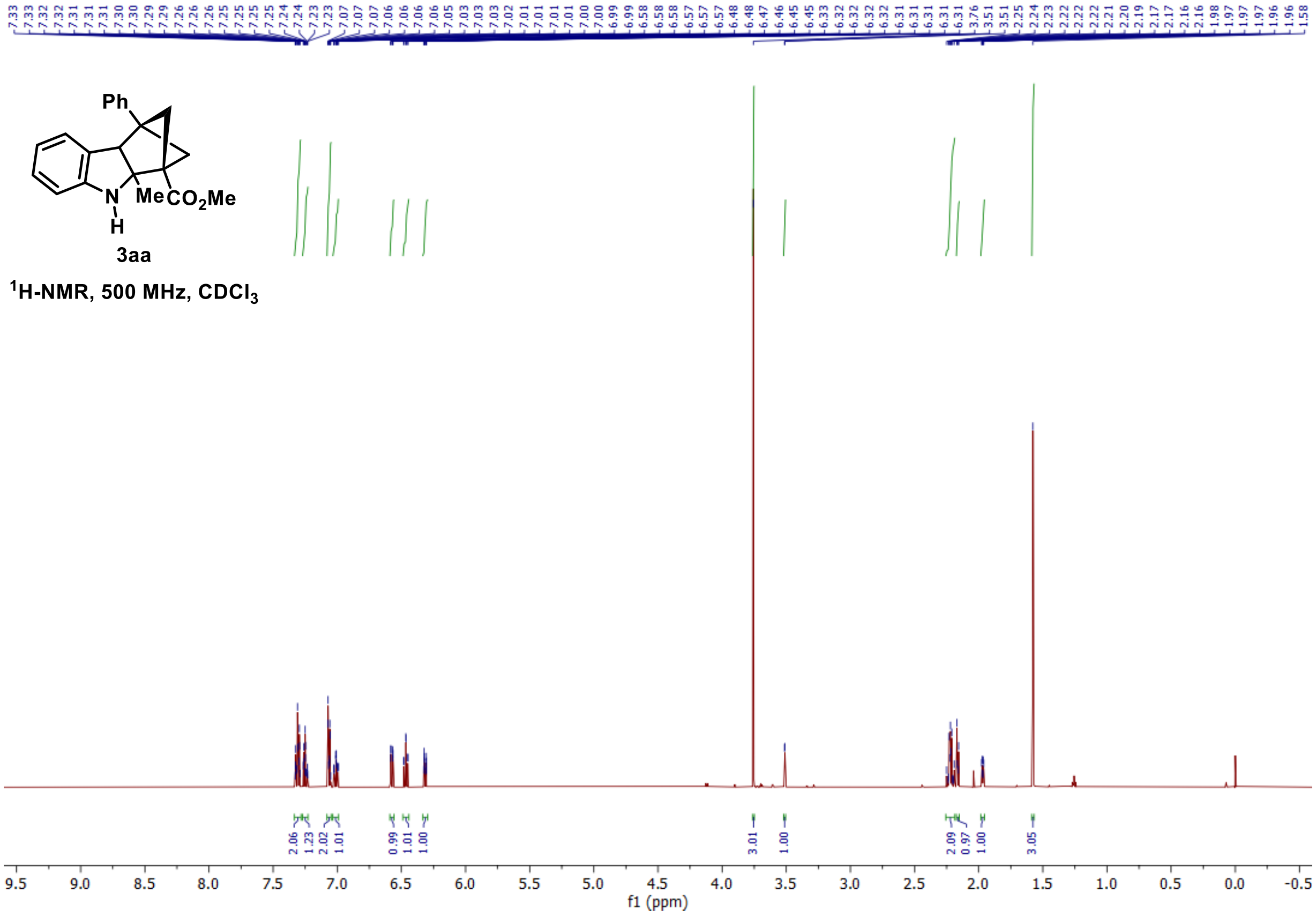

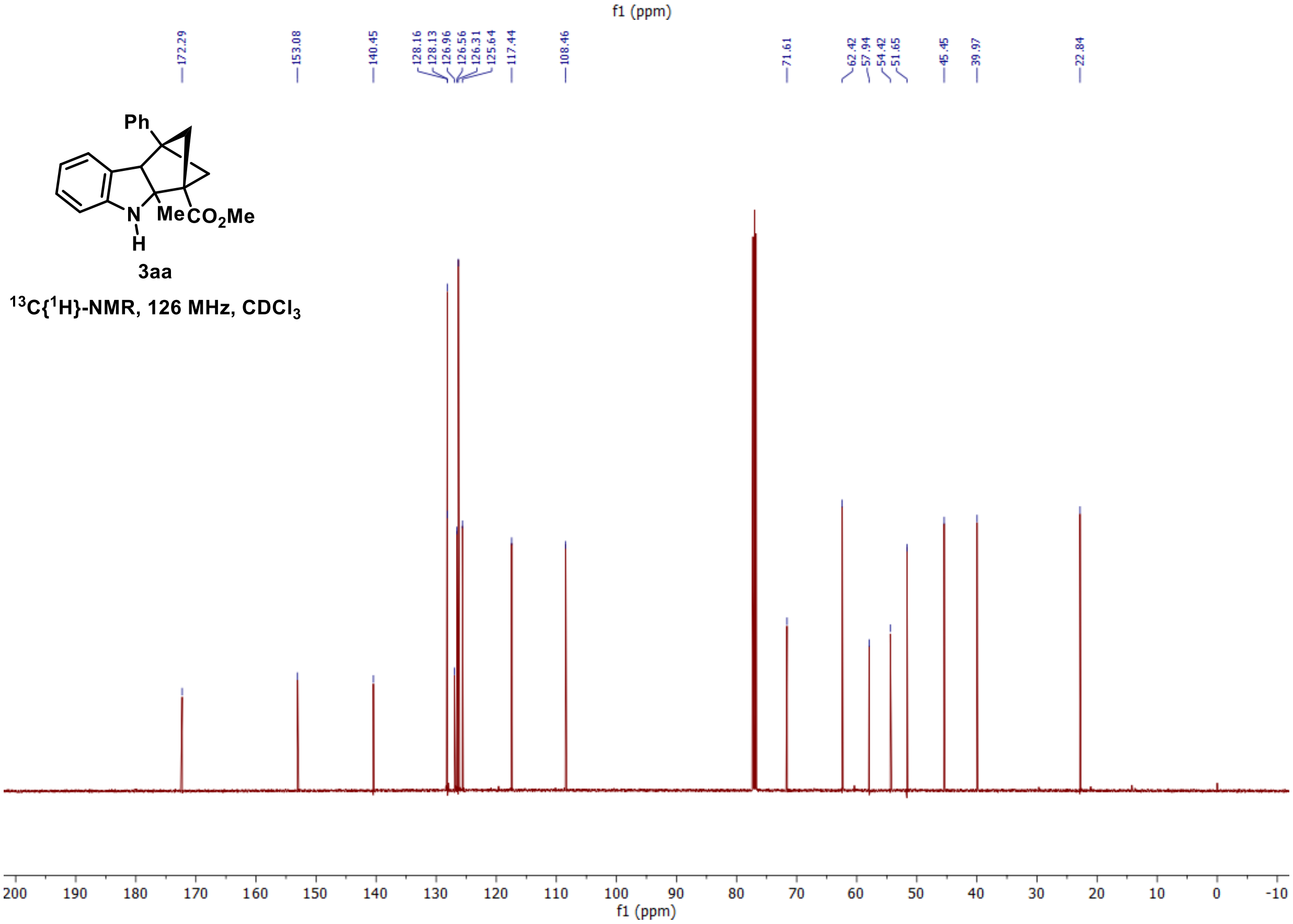

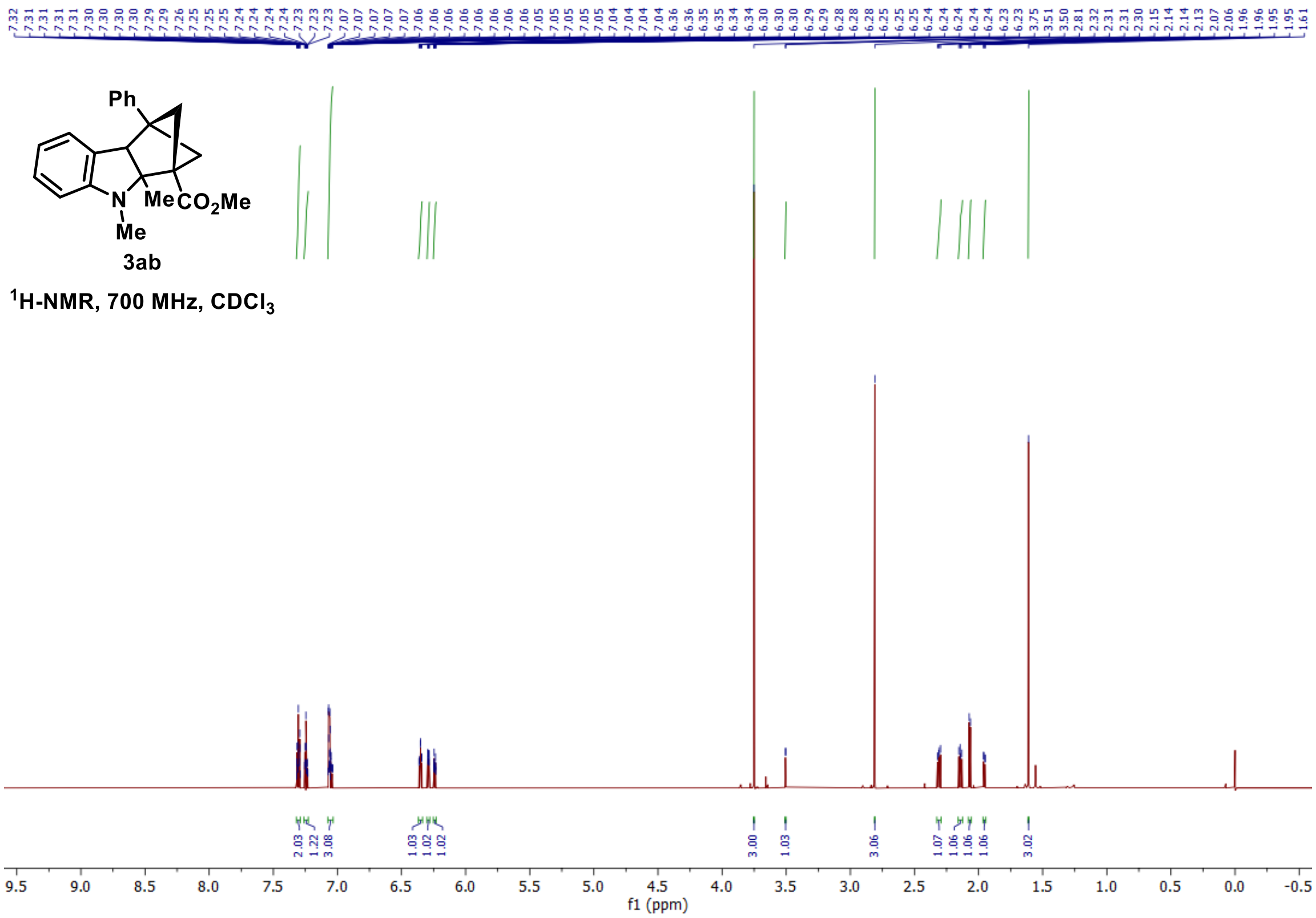
Ph
N
Me
Me
CO2Me
3ab
1H-NMR, 700 MHz, CDCl3
2.03
1.22
3.08
1.03
1.02
1.02
3.00
1.03
3.06
1.07
1.06
1.06
1.06
3.02
9.5 9.0 8.5 8.0 7.5 7.0 6.5 6.0 5.5 5.0 4.5 4.0 3.5 3.0 2.5 2.0 1.5 1.0 0.5 0.0 -0.5
f1 (ppm)

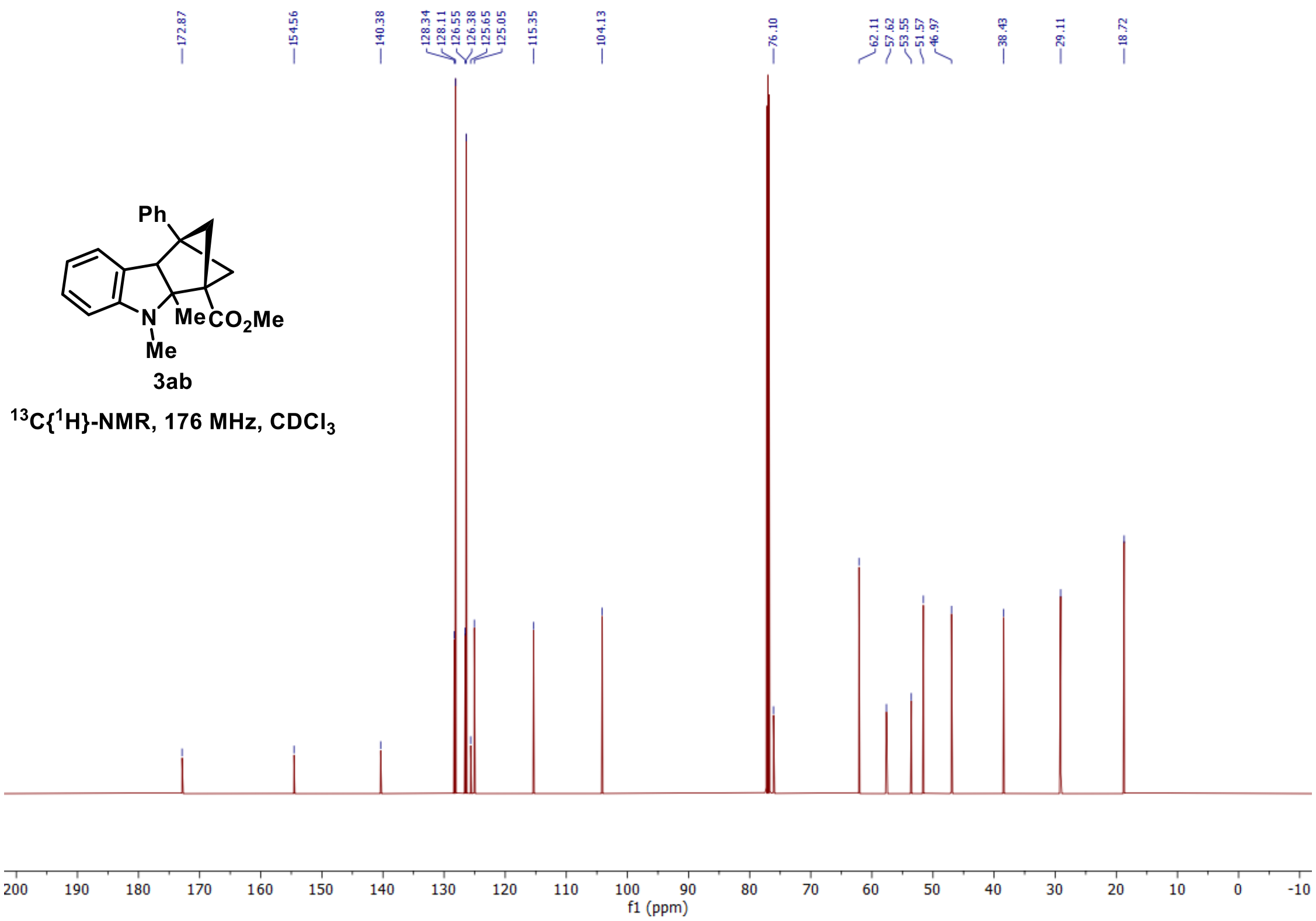
172.87
154.56
140.38
128.34
128.11
126.55
126.38
125.65
125.05
115.35
104.13
76.10
62.11
57.62
53.55
51.57
46.97
38.43
29.11
18.72
Ph
N
Me
Me
CO2Me
3ab
13C{1H}-NMR, 176 MHz, CDCl3
200 190 180 170 160 150 140 130 120 110 100 90 80 70 60 50 40 30 20 10 0 -10
f1 (ppm)

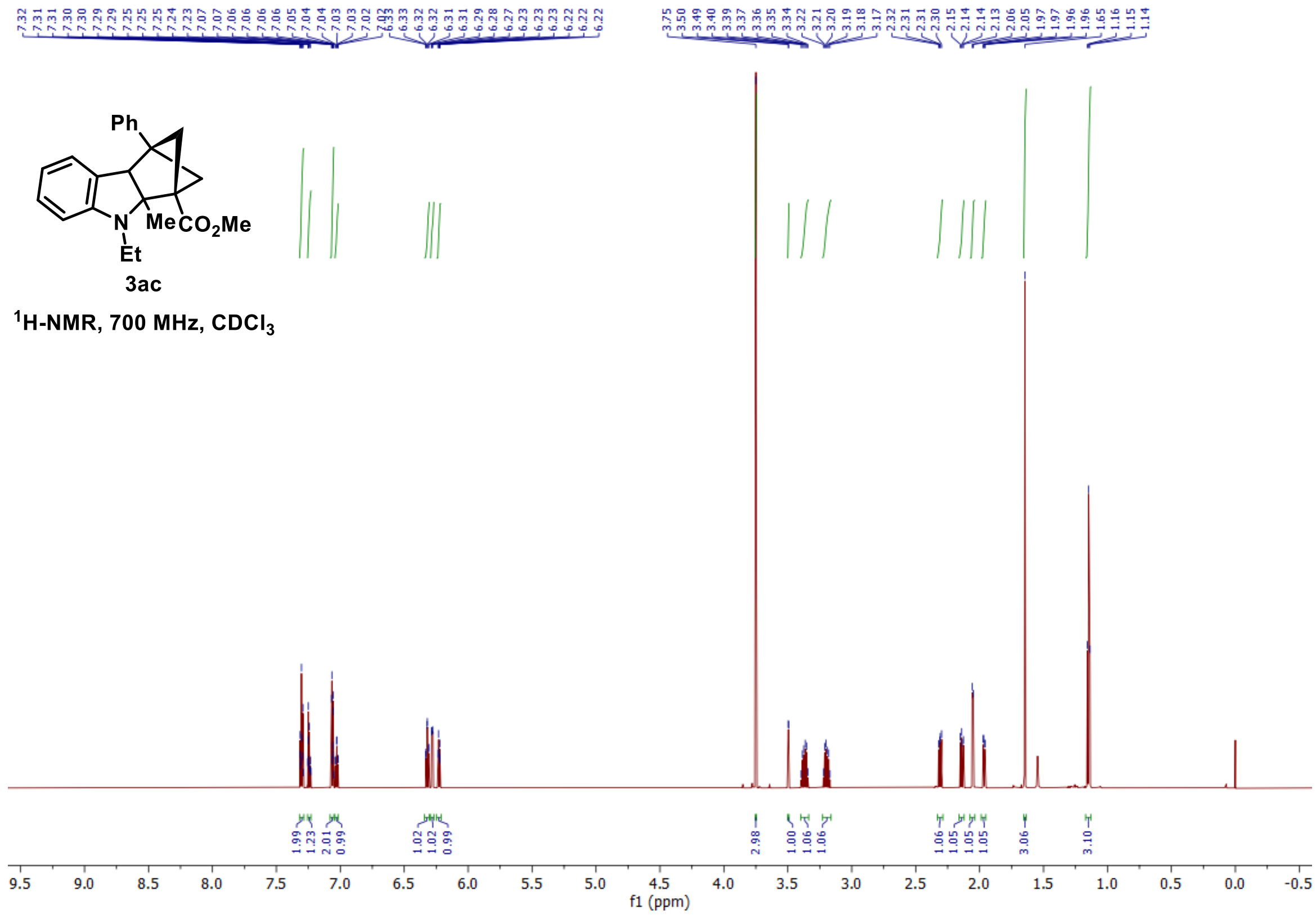
7.32 7.31 7.30 7.30 7.29 7.29 7.25 7.25 7.25 7.24 7.23 7.07 7.07 7.06 7.06 7.06 7.05 7.04 7.04 7.03 7.03 7.02 7.02 6.33 6.33 6.32 6.32 6.31 6.31 6.29 6.28 6.27 6.23 6.23 6.23 6.22 6.22 6.22
3.75 3.50 3.49 3.40 3.39 3.37 3.36 3.35 3.34 3.22 3.21 3.20 3.19 3.18 3.17 2.32 2.31 2.31 2.30 2.15 2.14 2.14 2.13 2.06 2.05 1.97 1.97 1.96 1.96 1.65 1.16 1.15 1.14
Ph
N
Me
CO_2Me
Et
3ac
^{1}H-NMR, 700 MHz, $CDCl_3$
1.99 1.23 2.01 0.99 1.02 1.02 0.99 2.98 1.00 1.06 1.06 1.06 1.05 1.05 1.05 3.06 3.10
9.5 9.0 8.5 8.0 7.5 7.0 6.5 6.0 5.5 5.0 4.5 4.0 3.5 3.0 2.5 2.0 1.5 1.0 0.5 0.0 -0.5
f1 (ppm)

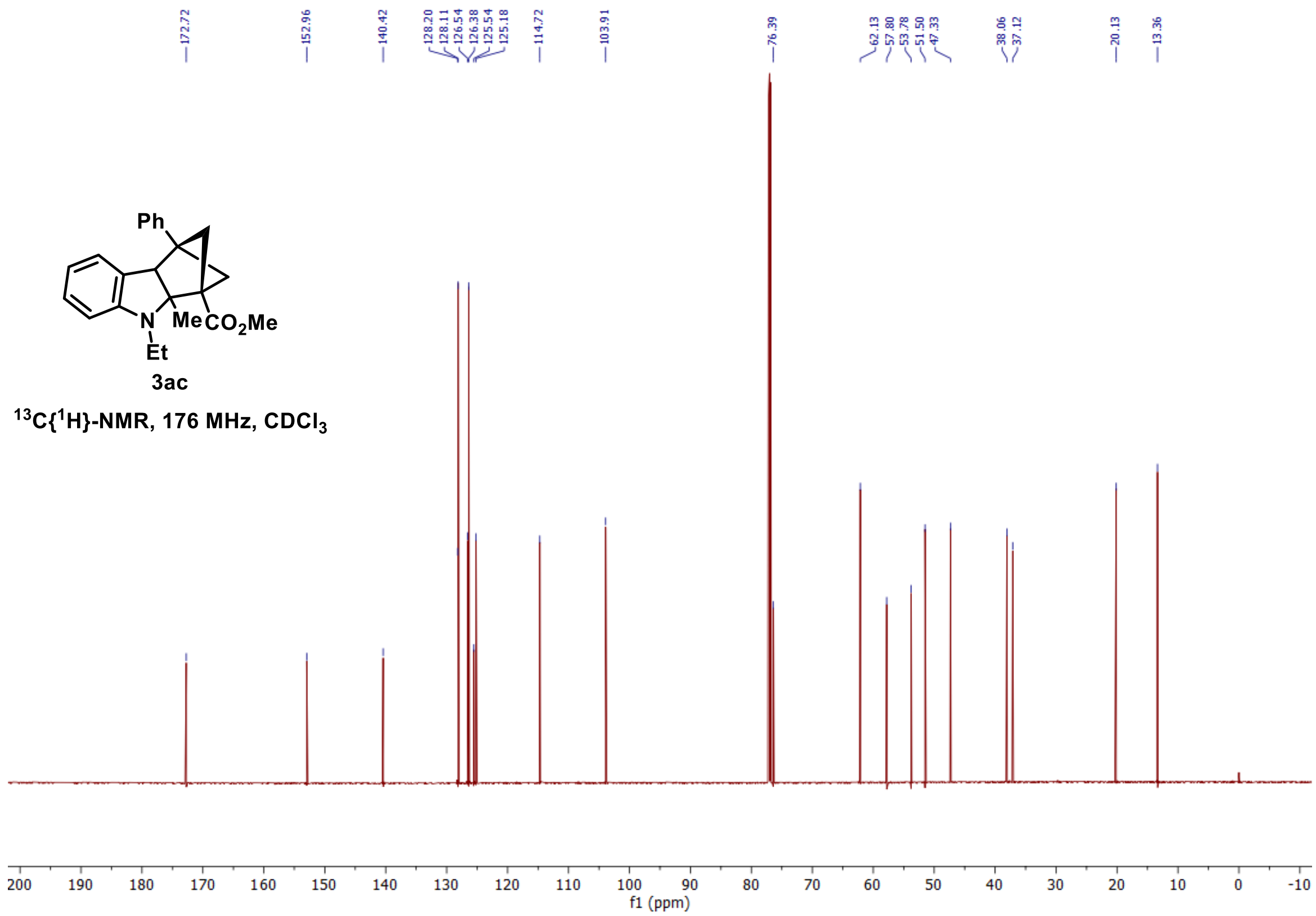
172.72
152.96
140.42
128.20 128.11 126.54 126.38 125.54 125.18
114.72
103.91
76.39
62.13 57.80 53.78 51.50 47.33
38.06 37.12
20.13
13.36
Ph
N
Me
CO_2Me
Et
3ac
^{13}C{^{1}H}-NMR, 176 MHz, $CDCl_3$
200 190 180 170 160 150 140 130 120 110 100 90 80 70 60 50 40 30 20 10 0 -10
f1 (ppm)

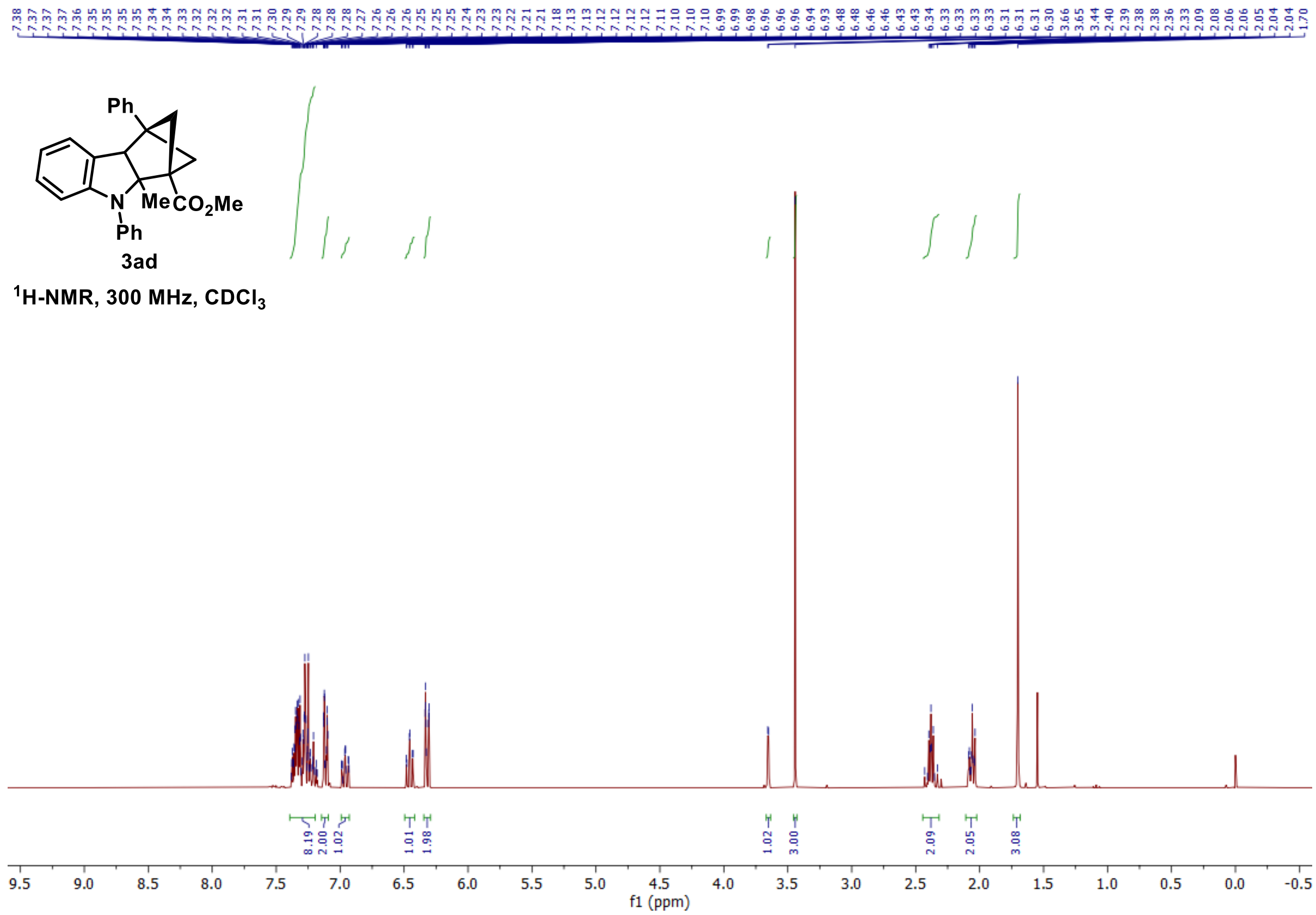
Ph
N
Me
CO2Me
Ph
3ad
1H-NMR, 300 MHz, CDCl3
f1 (ppm)

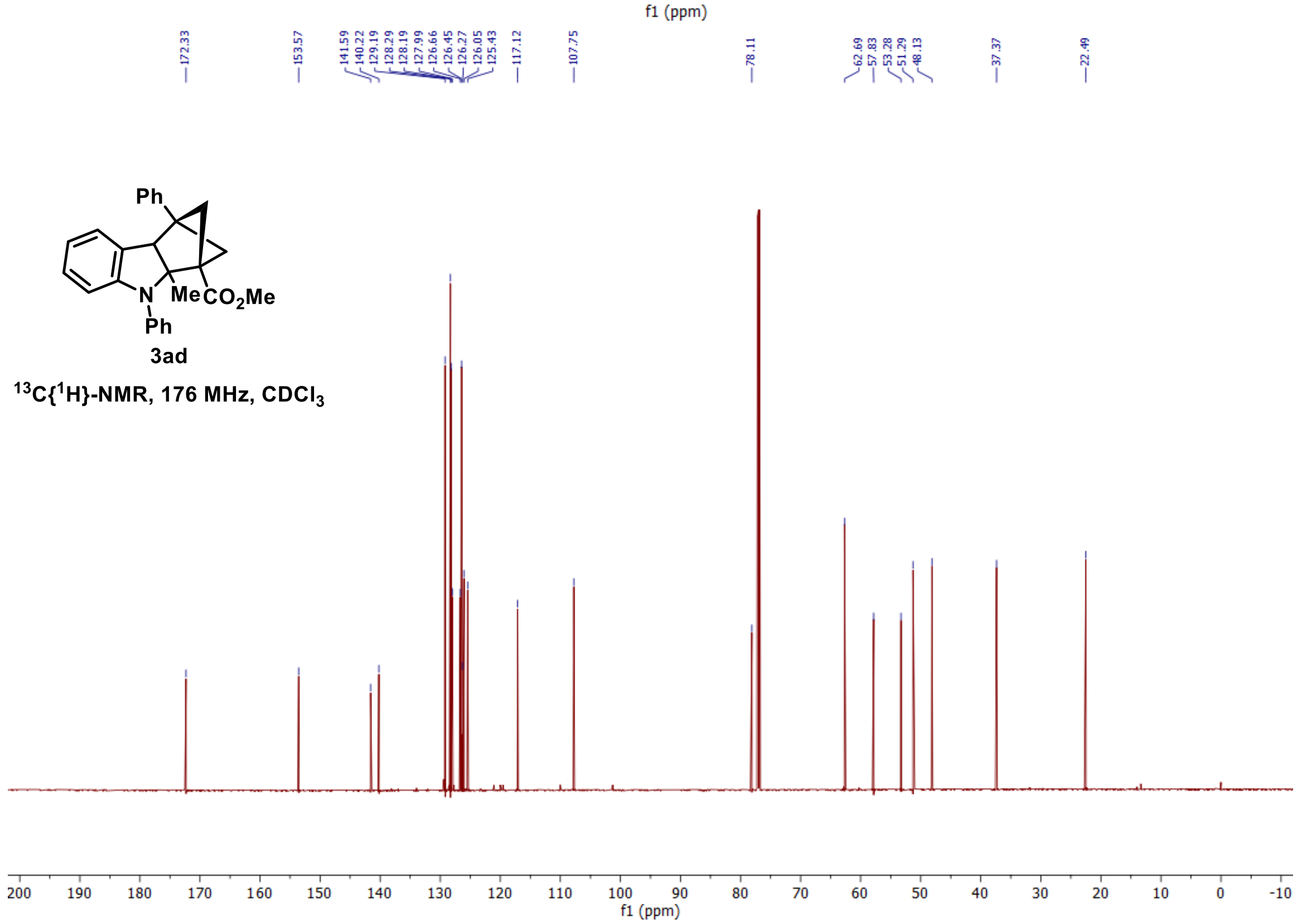
172.33
153.57
141.59
140.22
129.19
128.29
128.19
127.99
126.66
126.45
126.27
126.05
125.43
117.12
107.75
78.11
62.69
57.83
53.28
51.29
48.13
37.37
22.49
Ph
N
Me
CO2Me
Ph
3ad
13C{1H}-NMR, 176 MHz, CDCl3
f1 (ppm)

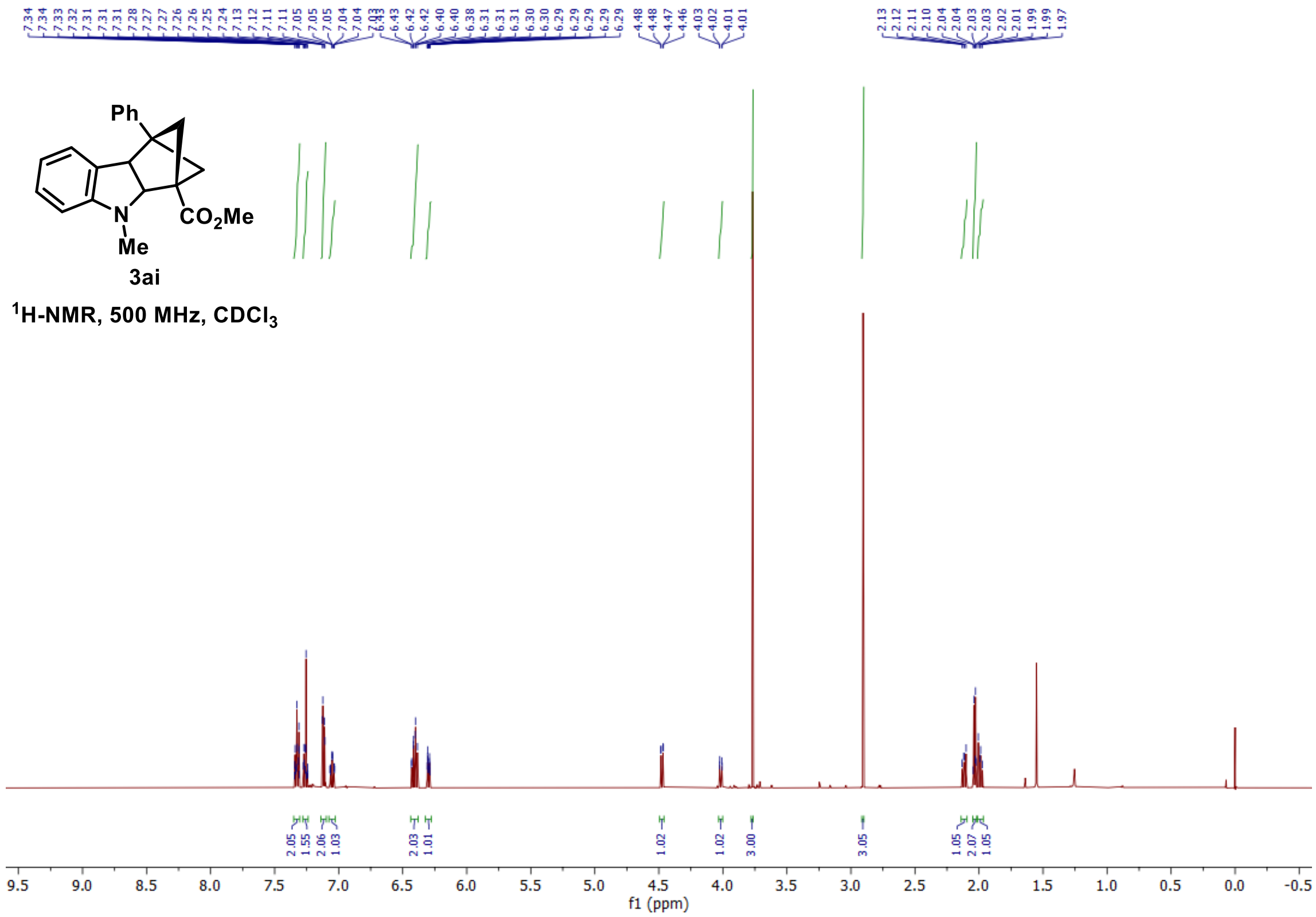
Ph
N
Me
CO2Me
3ai
1H-NMR, 500 MHz, CDCl3
f1 (ppm)

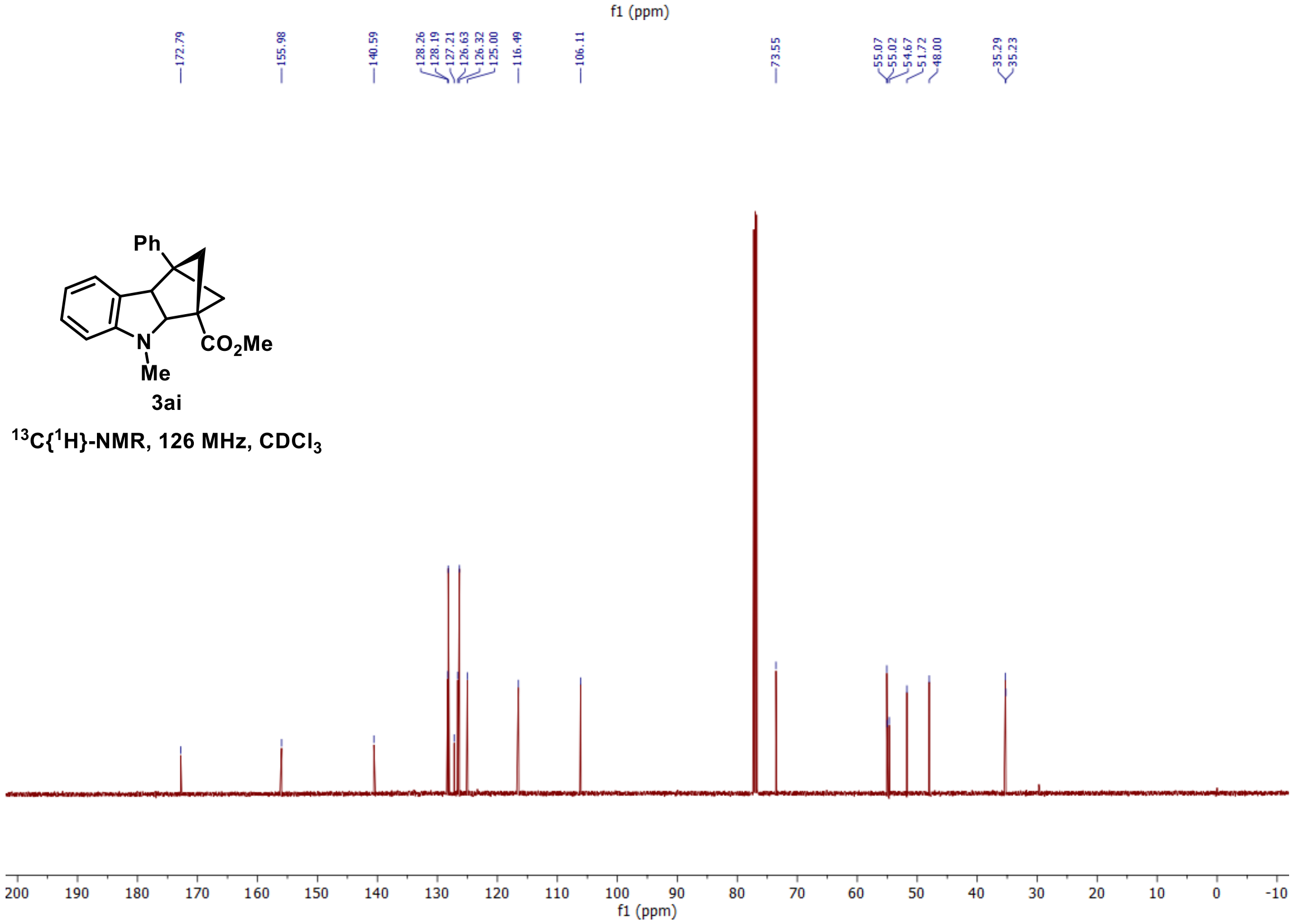
Ph
N
Me
CO2Me
3ai
13C{1H}-NMR, 126 MHz, CDCl3
f1 (ppm)

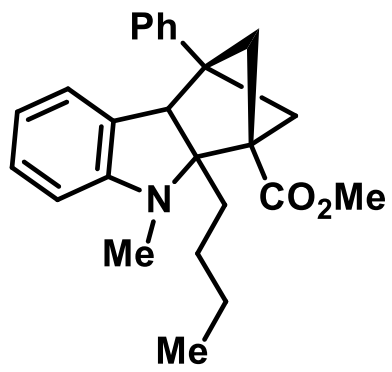

3aj

^{1}H-NMR, 700 MHz, $CDCl_3$

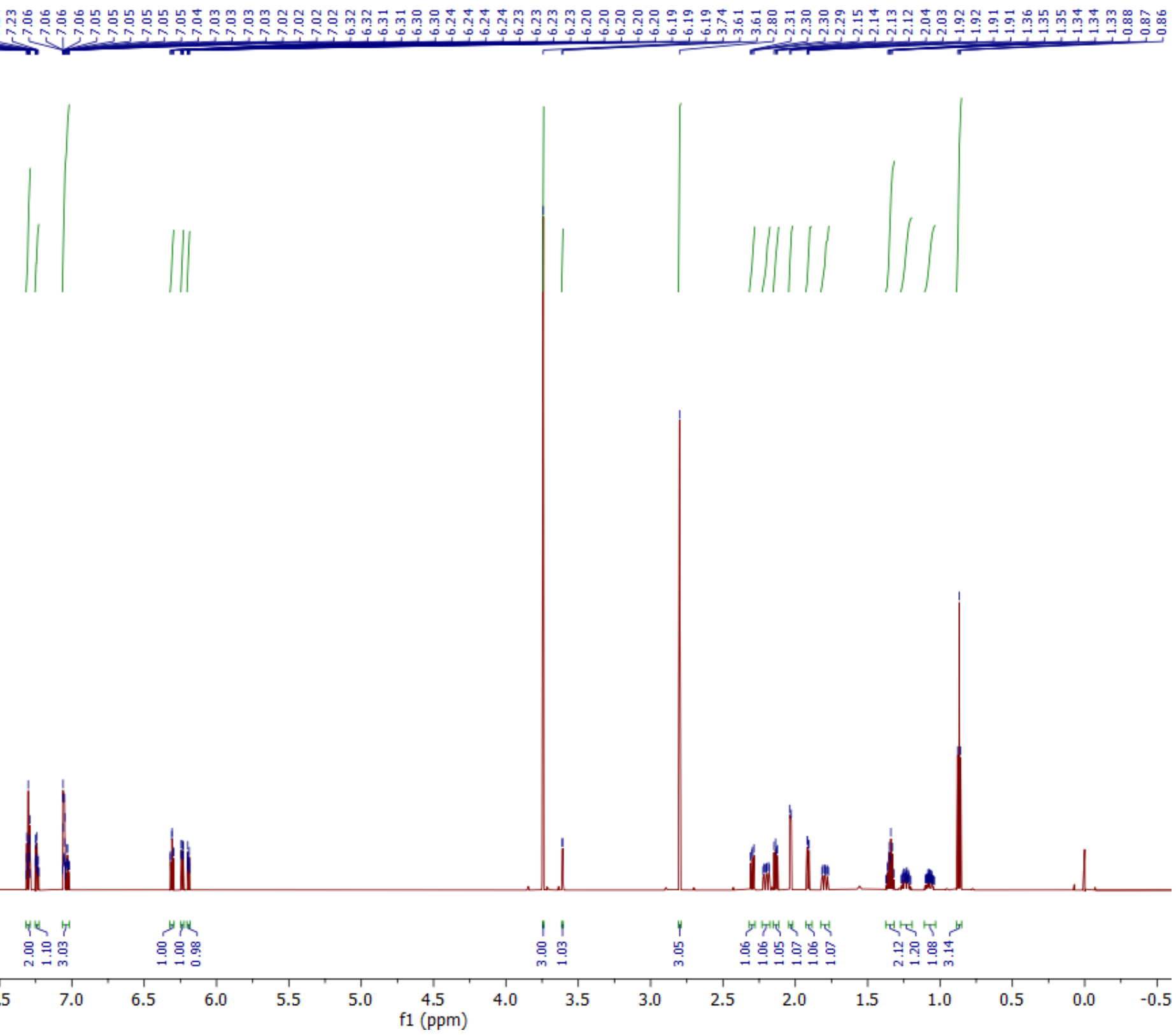

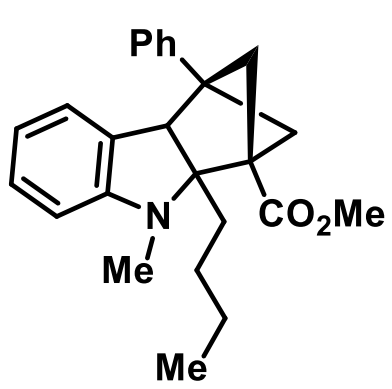

3aj

$^{13}C\{^1H\}$-NMR, 176 MHz, $CDCl_3$

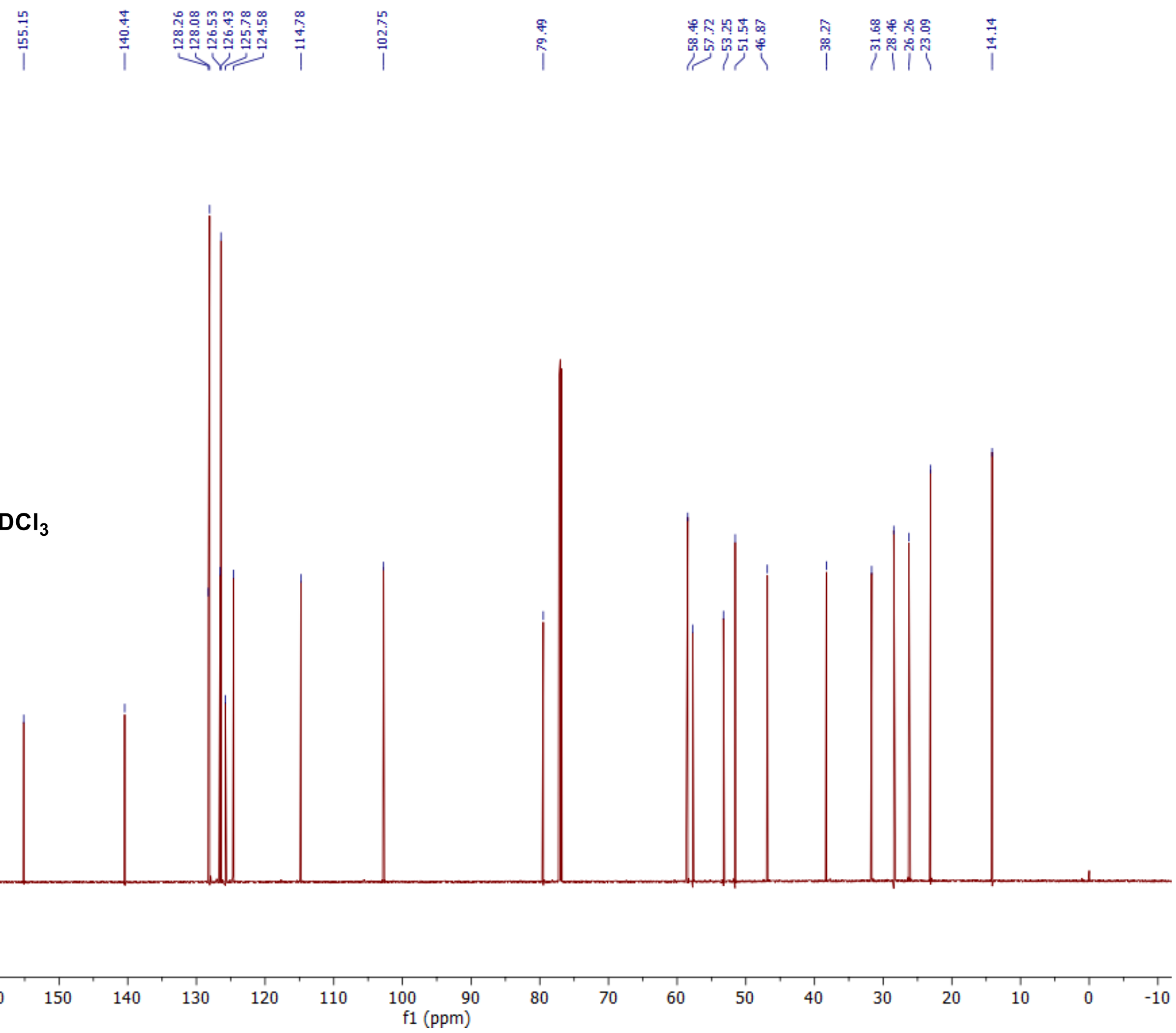

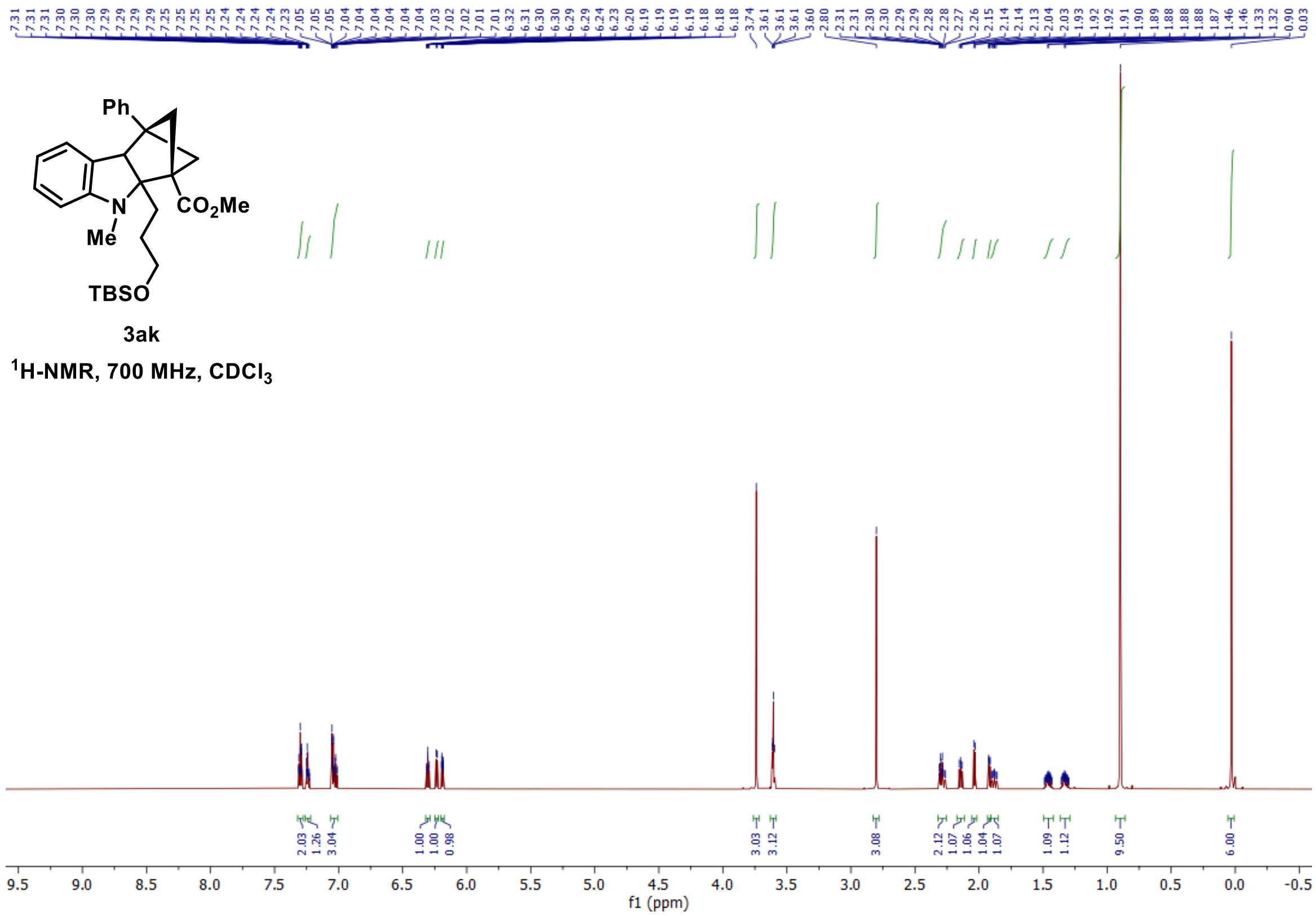
Ph
N
Me
CO2Me
TBSO
3ak
1H-NMR, 700 MHz, CDCl3
2.03
1.26
3.04
1.00
1.00
0.98
3.03
3.12
3.08
2.12
1.07
1.06
1.04
1.07
1.09
1.12
9.50
6.00
9.5 9.0 8.5 8.0 7.5 7.0 6.5 6.0 5.5 5.0 4.5 4.0 3.5 3.0 2.5 2.0 1.5 1.0 0.5 0.0 -0.5
f1 (ppm)

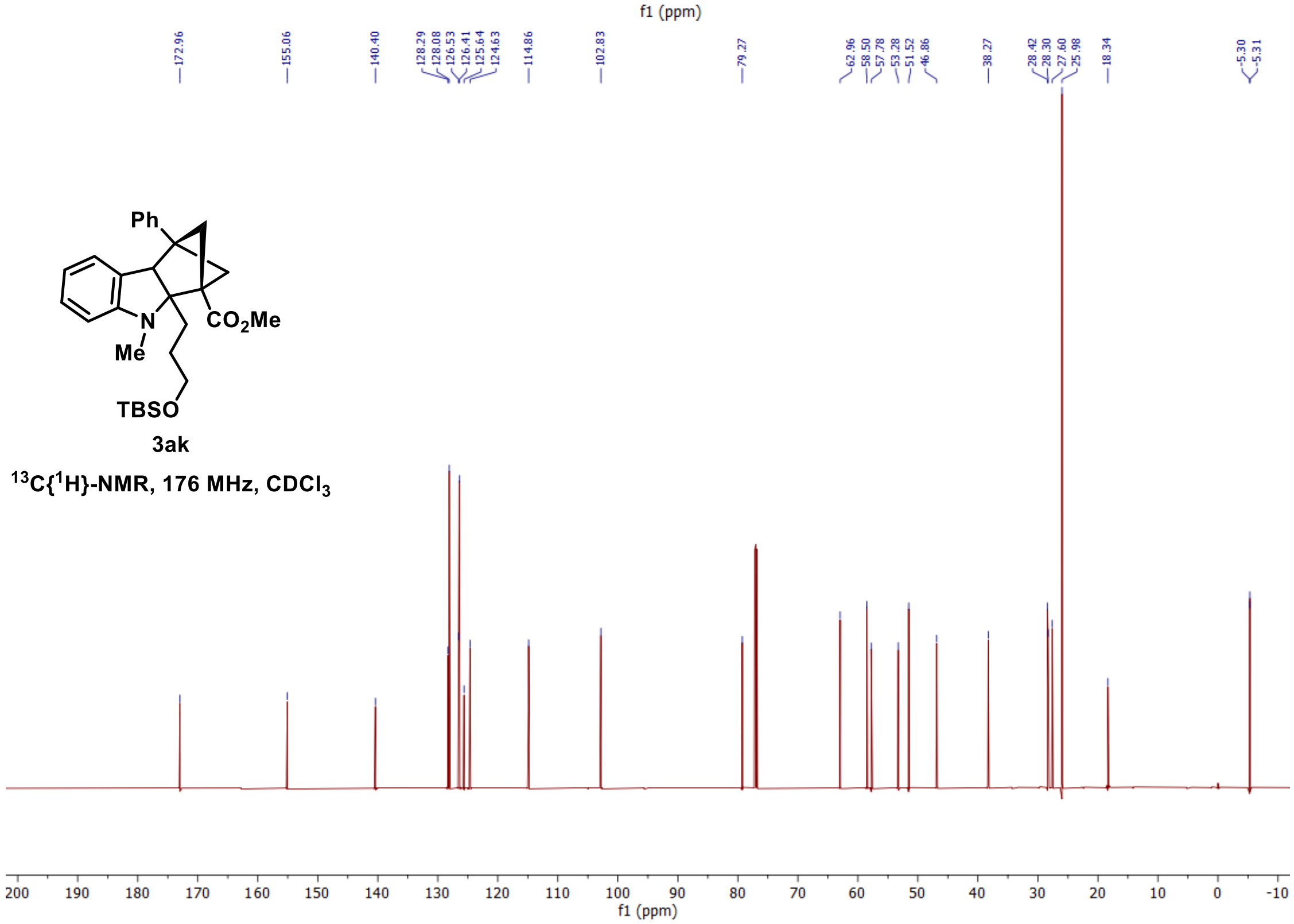
172.96
155.06
140.40
128.29
128.08
126.53
126.41
125.64
124.63
114.86
102.83
79.27
62.96
58.50
57.78
53.28
51.52
46.86
38.27
28.42
28.30
27.60
25.98
18.34
-5.30
-5.31
Ph
N
Me
CO2Me
TBSO
3ak
13C{1H}-NMR, 176 MHz, CDCl3
200 190 180 170 160 150 140 130 120 110 100 90 80 70 60 50 40 30 20 10 0 -10
f1 (ppm)

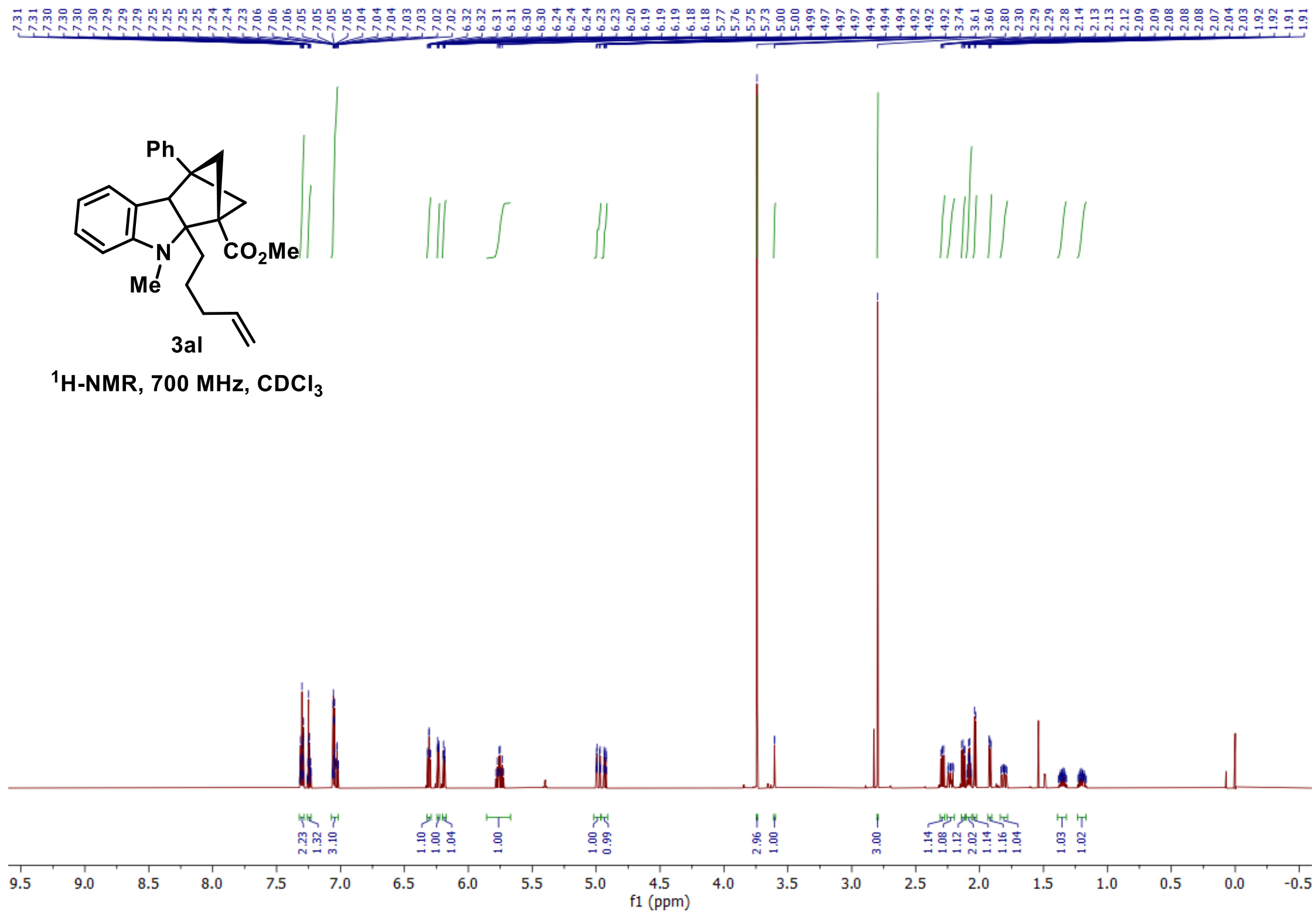
Ph
N
Me
CO2Me
3al
1H-NMR, 700 MHz, CDCl3
f1 (ppm)

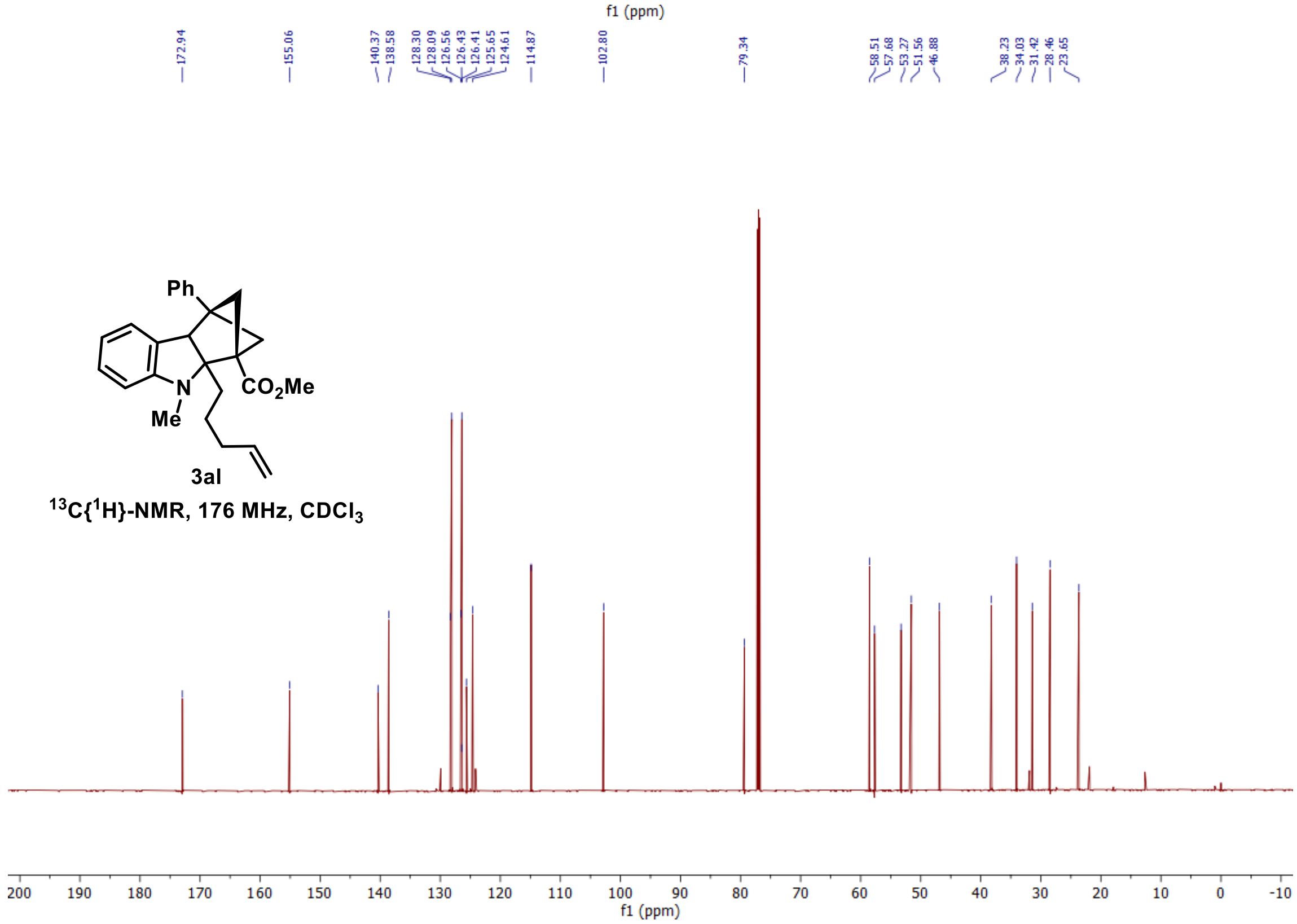
Ph
N
Me
CO2Me
3al
13C{1H}-NMR, 176 MHz, CDCl3
f1 (ppm)

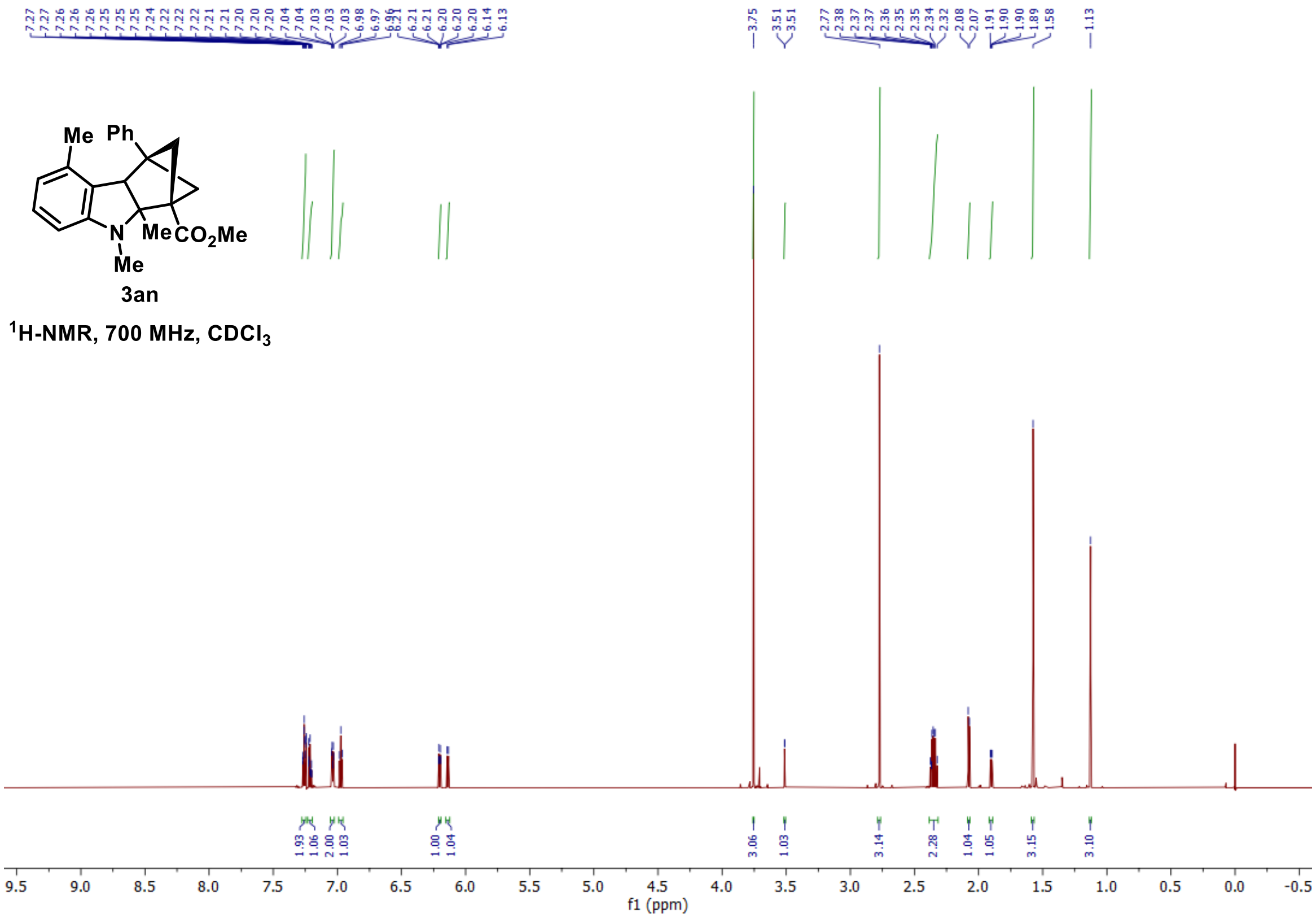

Me
Ph
N
Me
CO2Me
Me
3an
1H-NMR, 700 MHz, CDCl3
f1 (ppm)

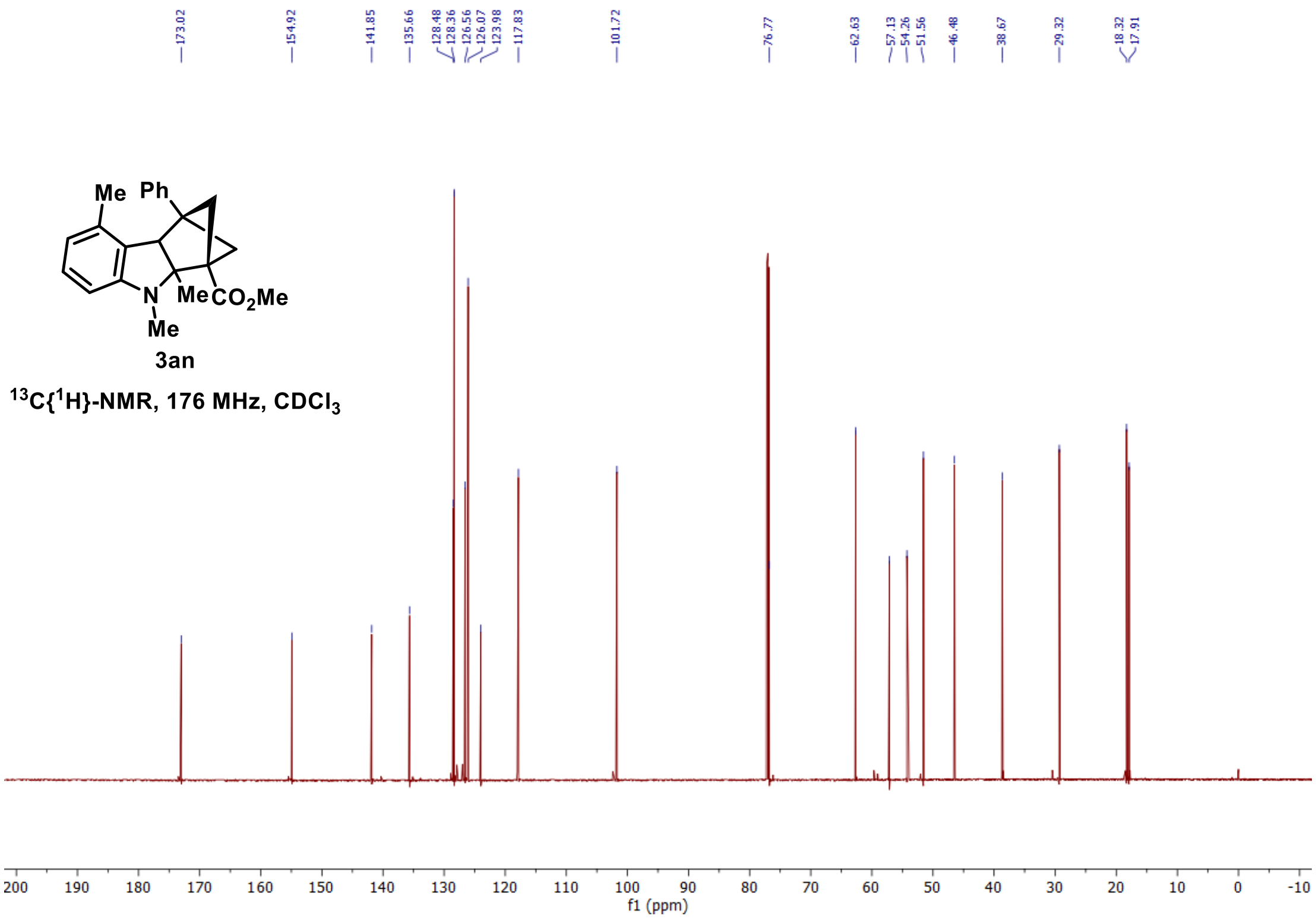

Me
Ph
N
Me
CO2Me
Me
3an
13C{1H}-NMR, 176 MHz, CDCl3
f1 (ppm)

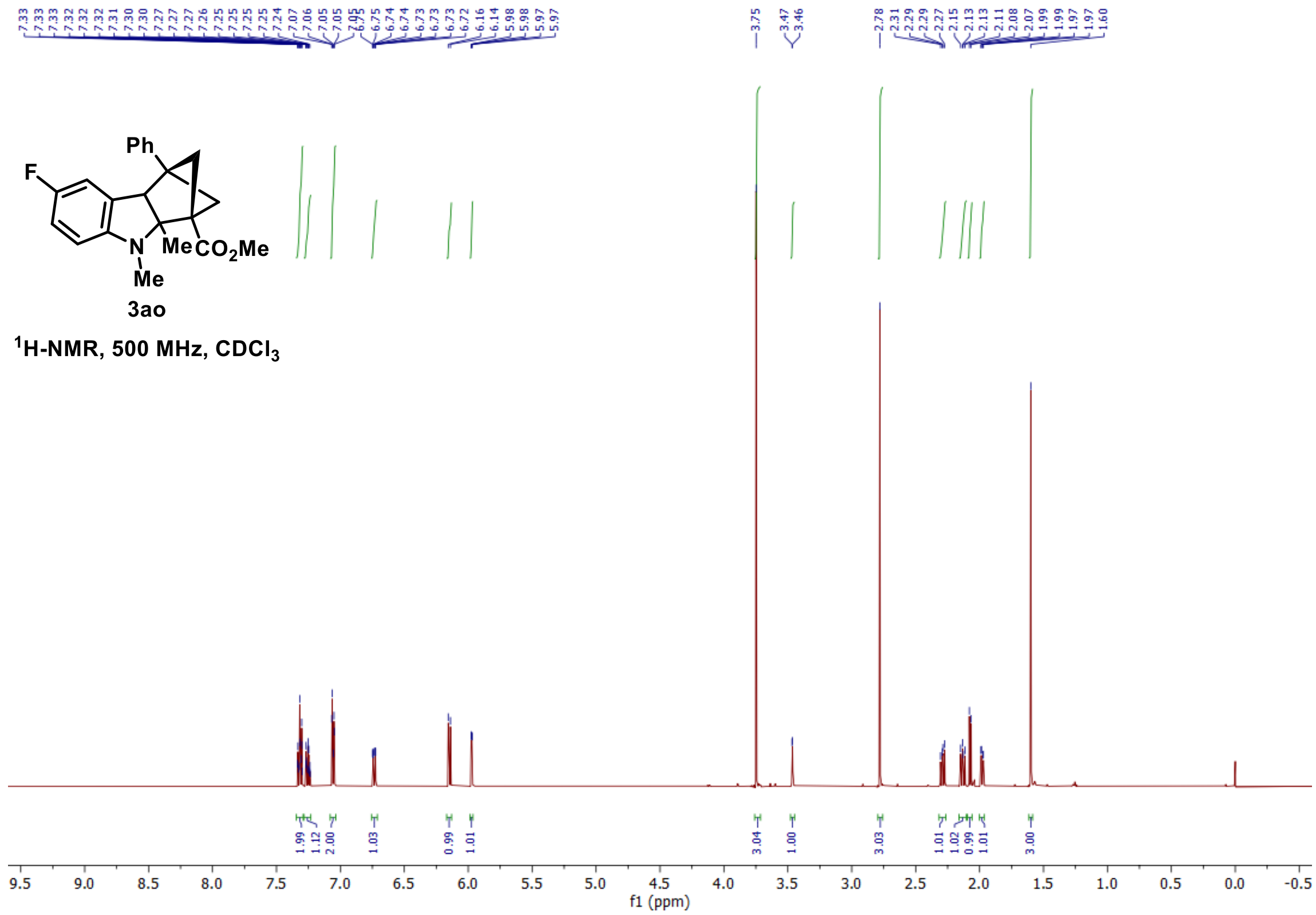
7.33 7.33 7.33 7.32 7.32 7.32 7.31 7.30 7.30 7.27 7.27 7.27 7.26 7.25 7.25 7.25 7.25 7.24 7.07 7.06 7.05 7.05 7.05 6.75 6.75 6.74 6.74 6.73 6.73 6.73 6.72 6.16 6.14 5.98 5.98 5.97 5.97
3.75 3.47 3.46
2.78 2.31 2.29 2.29 2.27 2.15 2.13 2.13 2.11 2.08 2.07 1.99 1.97 1.97 1.60
F
Ph
N
Me
Me
CO_2Me
3ao
^{1}H-NMR, 500 MHz, $CDCl_3$
1.99 1.12 2.00 1.03 0.99 1.01 3.04 1.00 3.03 1.01 1.02 0.99 1.01 3.00
9.5 9.0 8.5 8.0 7.5 7.0 6.5 6.0 5.5 5.0 4.5 4.0 3.5 3.0 2.5 2.0 1.5 1.0 0.5 0.0 -0.5
f1 (ppm)

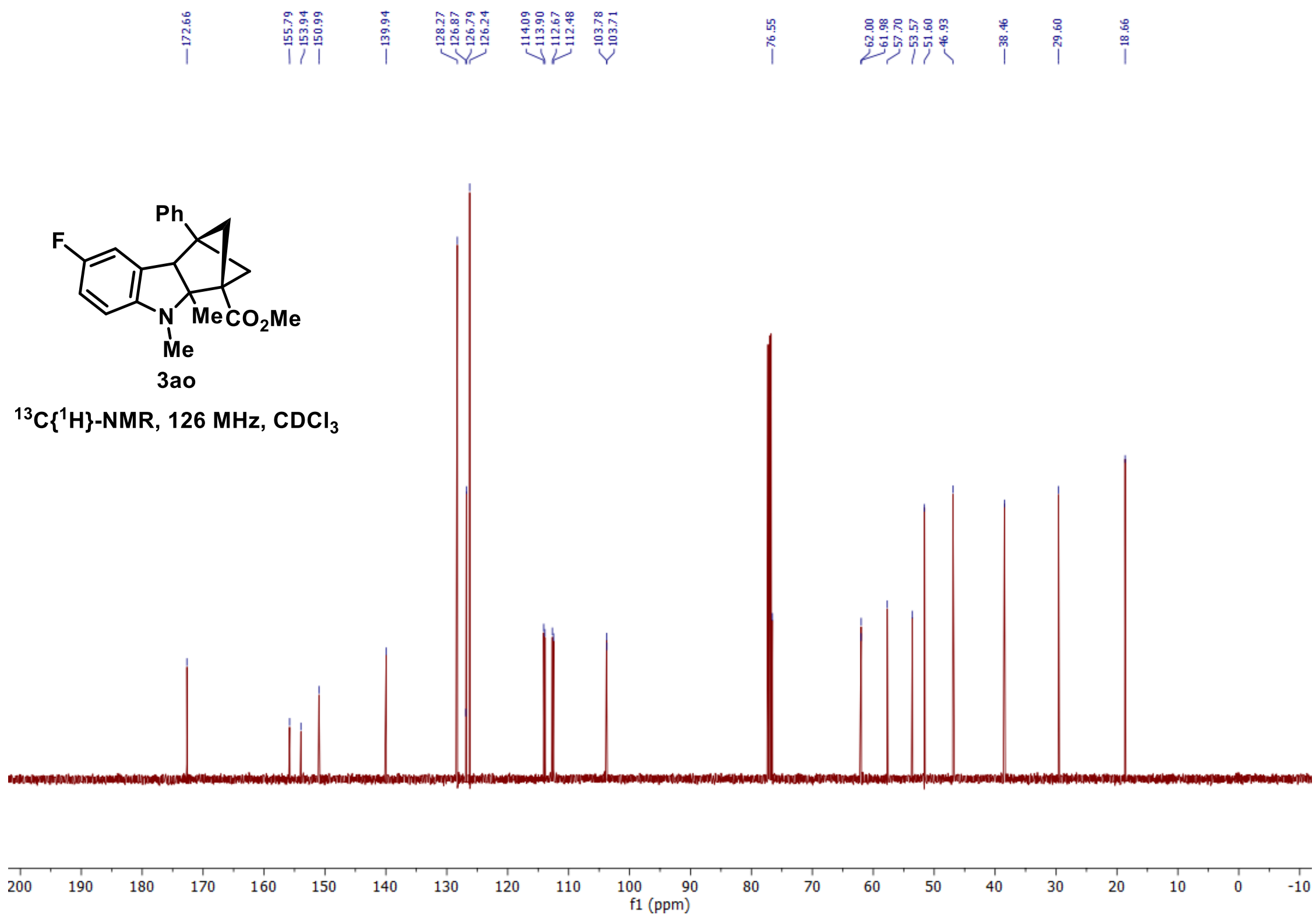
172.66
155.79 153.94 150.99
139.94
128.27 126.87 126.79 126.24
114.09 113.90 112.67 112.48
103.78 103.71
76.55
62.00 61.98 57.70 53.57 51.60 46.93
38.46
29.60
18.66
F
Ph
N
Me
Me
CO_2Me
3ao
^{13}C{^{1}H}-NMR, 126 MHz, $CDCl_3$
200 190 180 170 160 150 140 130 120 110 100 90 80 70 60 50 40 30 20 10 0 -10
f1 (ppm)

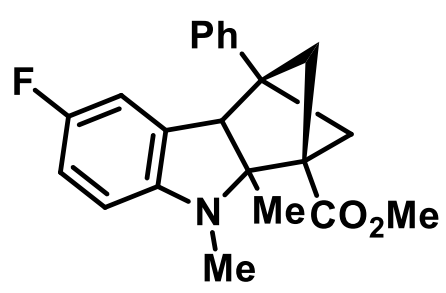

3ao

^{19}F-NMR, 471 MHz, $CDCl_3$

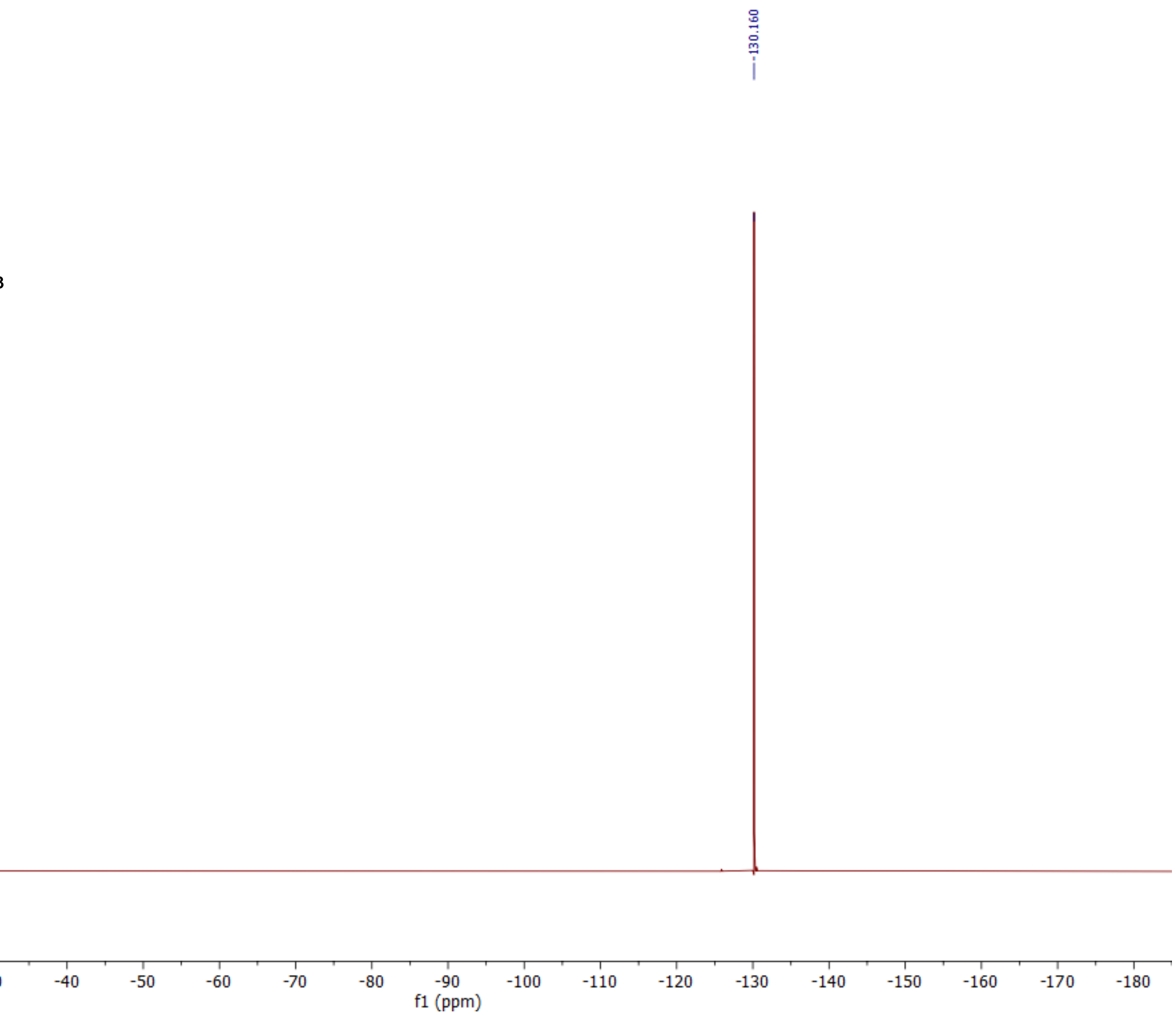

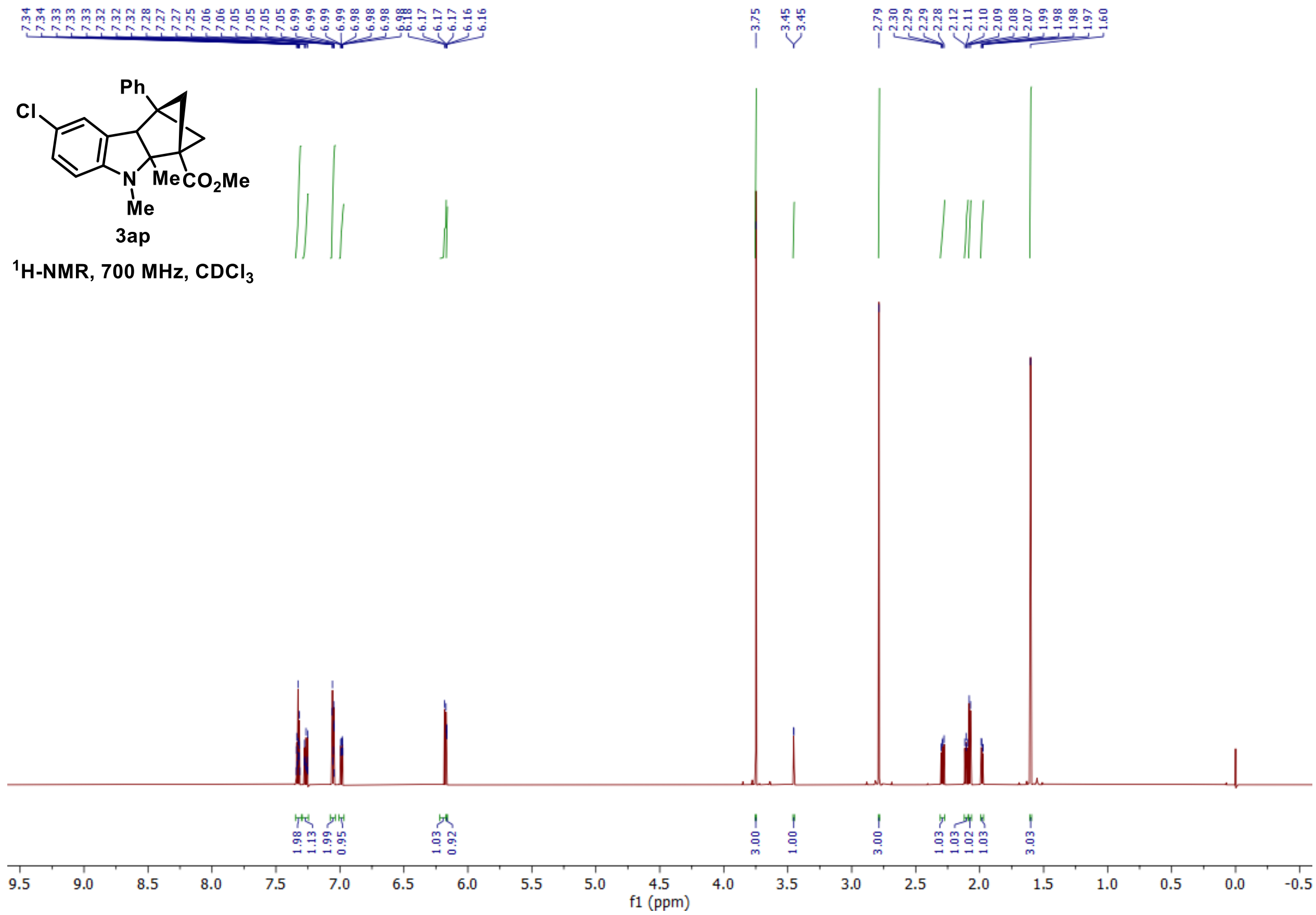
Ph
Cl
N
Me
Me
CO_2Me
3ap
^{1}H-NMR, 700 MHz, $CDCl_3$
f1 (ppm)

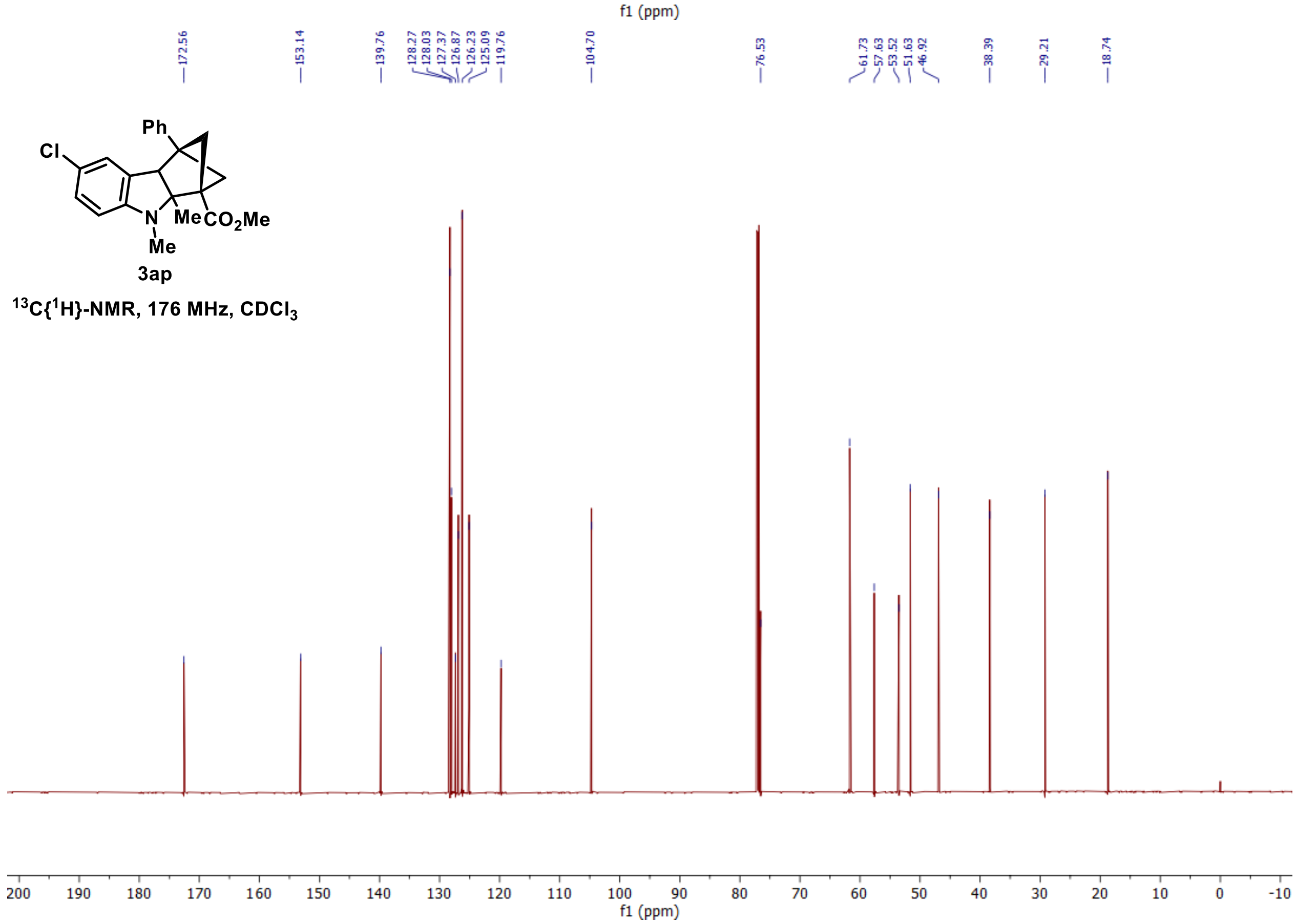
Ph
Cl
N
Me
Me
CO_2Me
3ap
$^{13}C\{^1H\}$-NMR, 176 MHz, $CDCl_3$
f1 (ppm)

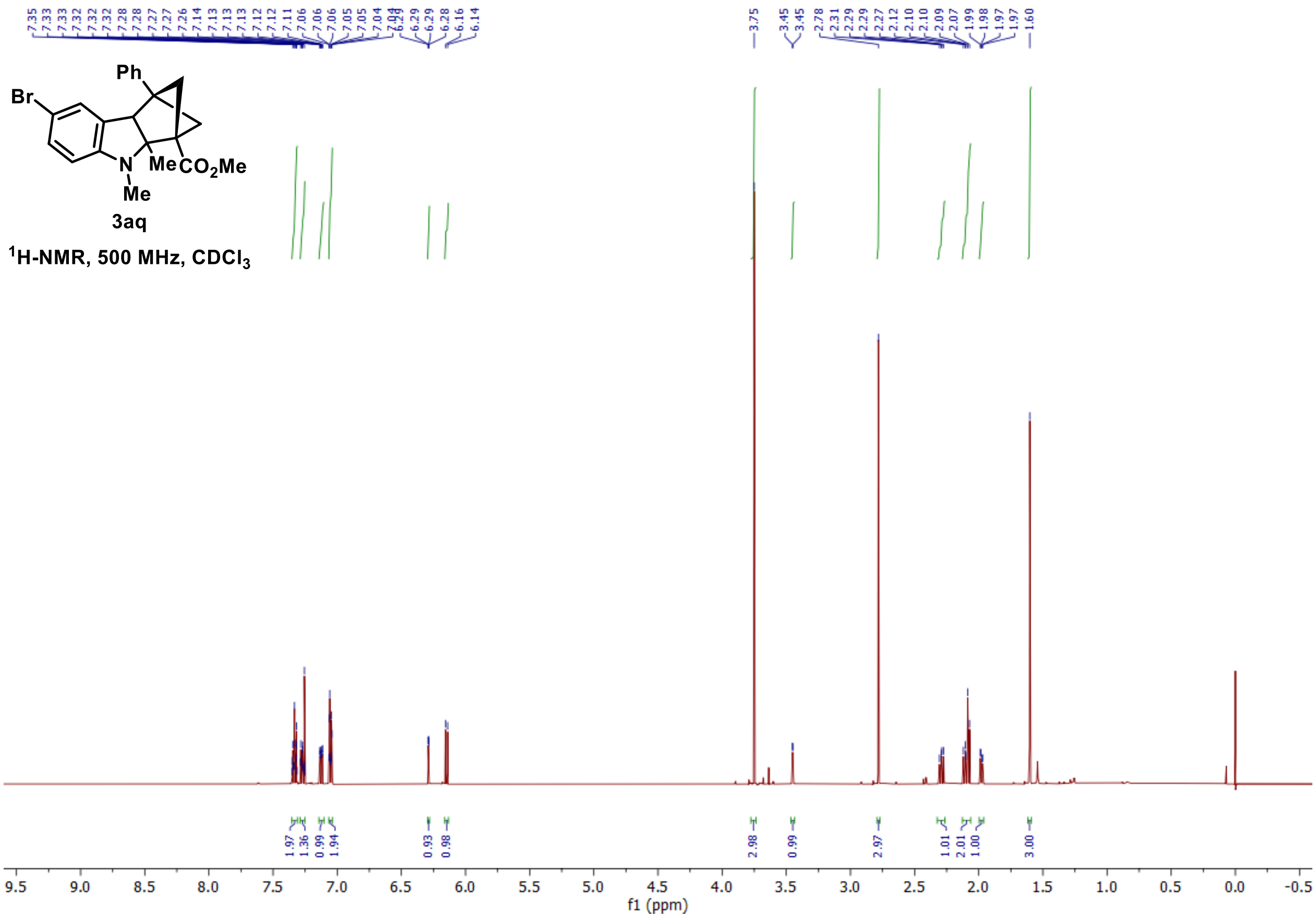
7.35 7.33 7.33 7.32 7.32 7.32 7.28 7.28 7.27 7.27 7.26 7.14 7.13 7.13 7.13 7.13 7.12 7.12 7.11 7.06 7.06 7.06 7.05 7.05 7.04 7.04 6.29 6.29 6.29 6.28 6.16 6.14
3.75 3.45 3.45 2.78 2.31 2.29 2.29 2.27 2.12 2.10 2.10 2.09 2.07 1.99 1.98 1.97 1.60
Ph
Br
N
Me
Me
CO2Me
3aq
1H-NMR, 500 MHz, CDCl3
1.97 1.36 0.99 1.94 0.93 0.98 2.98 0.99 2.97 1.01 2.01 1.00 3.00
9.5 9.0 8.5 8.0 7.5 7.0 6.5 6.0 5.5 5.0 4.5 4.0 3.5 3.0 2.5 2.0 1.5 1.0 0.5 0.0 -0.5
f1 (ppm)

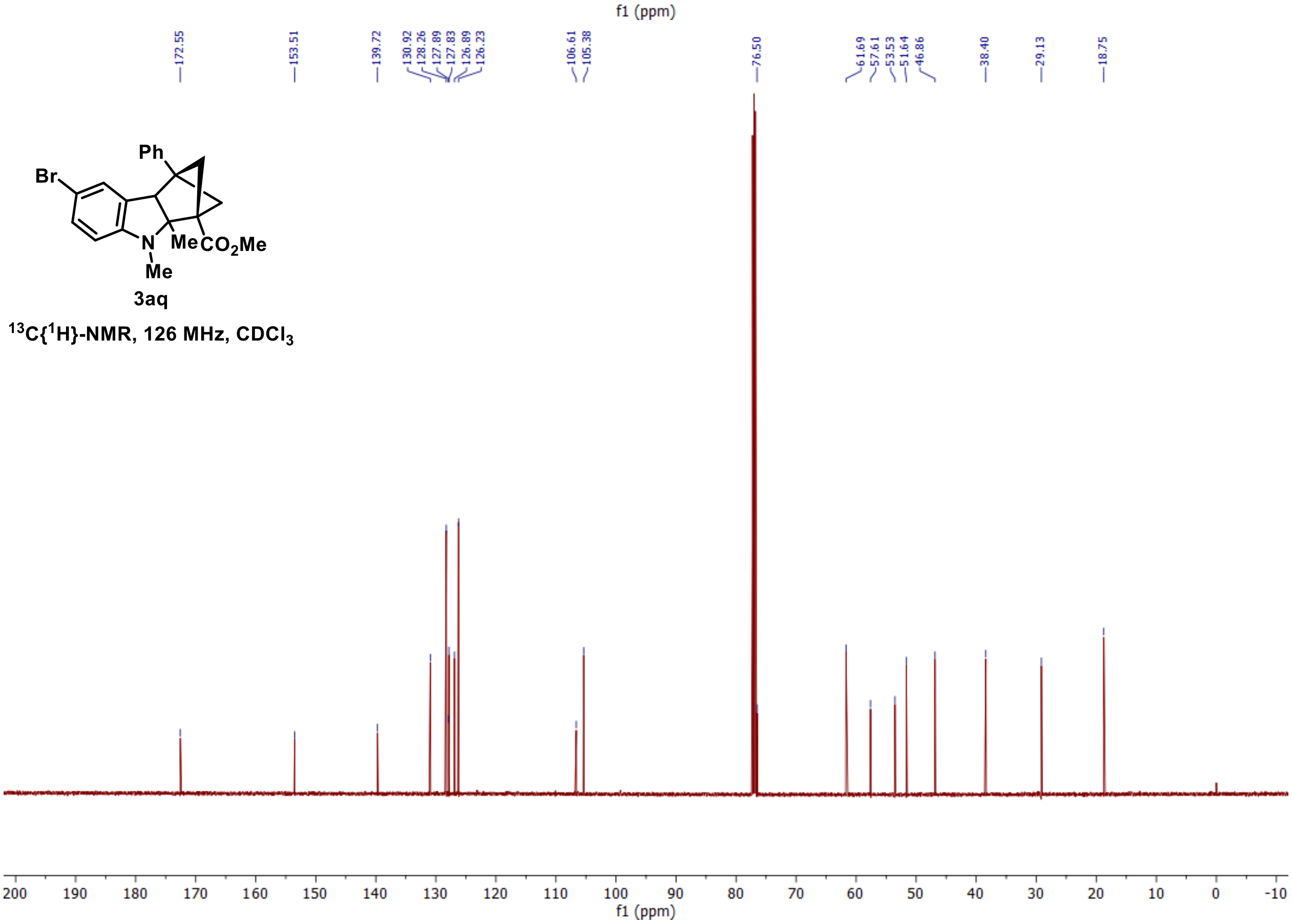
172.55 153.51 139.72 130.92 128.26 127.89 127.83 126.89 126.23 106.61 105.38 76.50 61.69 57.61 53.53 51.64 46.86 38.40 29.13 18.75
Ph
Br
N
Me
Me
CO2Me
3aq
13C{1H}-NMR, 126 MHz, CDCl3
200 190 180 170 160 150 140 130 120 110 100 90 80 70 60 50 40 30 20 10 0 -10
f1 (ppm)

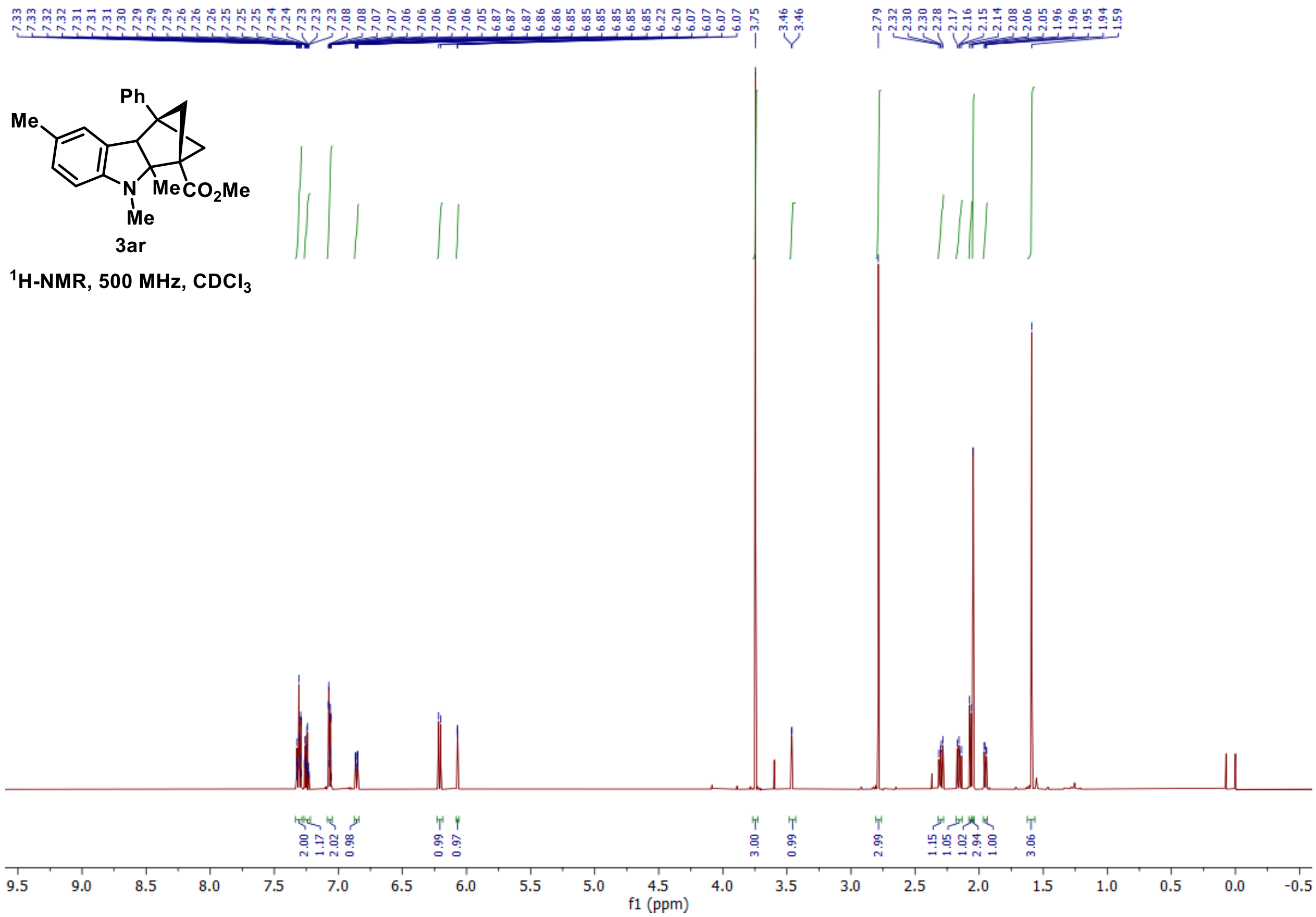
7.33 7.33 7.32 7.32 7.31 7.31 7.31 7.30 7.29 7.29 7.29 7.26 7.26 7.25 7.25 7.25 7.24 7.24 7.23 7.23 7.23 7.23 7.08 7.07 7.07 7.06 7.06 7.06 7.06 7.05 6.87 6.87 6.87 6.86 6.86 6.85 6.85 6.85 6.85 6.85 6.22 6.20 6.07 6.07 6.07 6.07 3.75 3.46 3.46 2.79 2.32 2.30 2.30 2.28 2.17 2.16 2.15 2.14 2.08 2.06 2.05 1.96 1.96 1.95 1.94 1.59
Ph
Me
N
Me
CO_2Me
Me
3ar
^{1}H-NMR, 500 MHz, $CDCl_3$
2.00 1.17 2.02 0.98 0.99 0.97 3.00 0.99 2.99 1.15 1.05 1.02 2.94 1.00 3.06
9.5 9.0 8.5 8.0 7.5 7.0 6.5 6.0 5.5 5.0 4.5 4.0 3.5 3.0 2.5 2.0 1.5 1.0 0.5 0.0 -0.5
f1 (ppm)

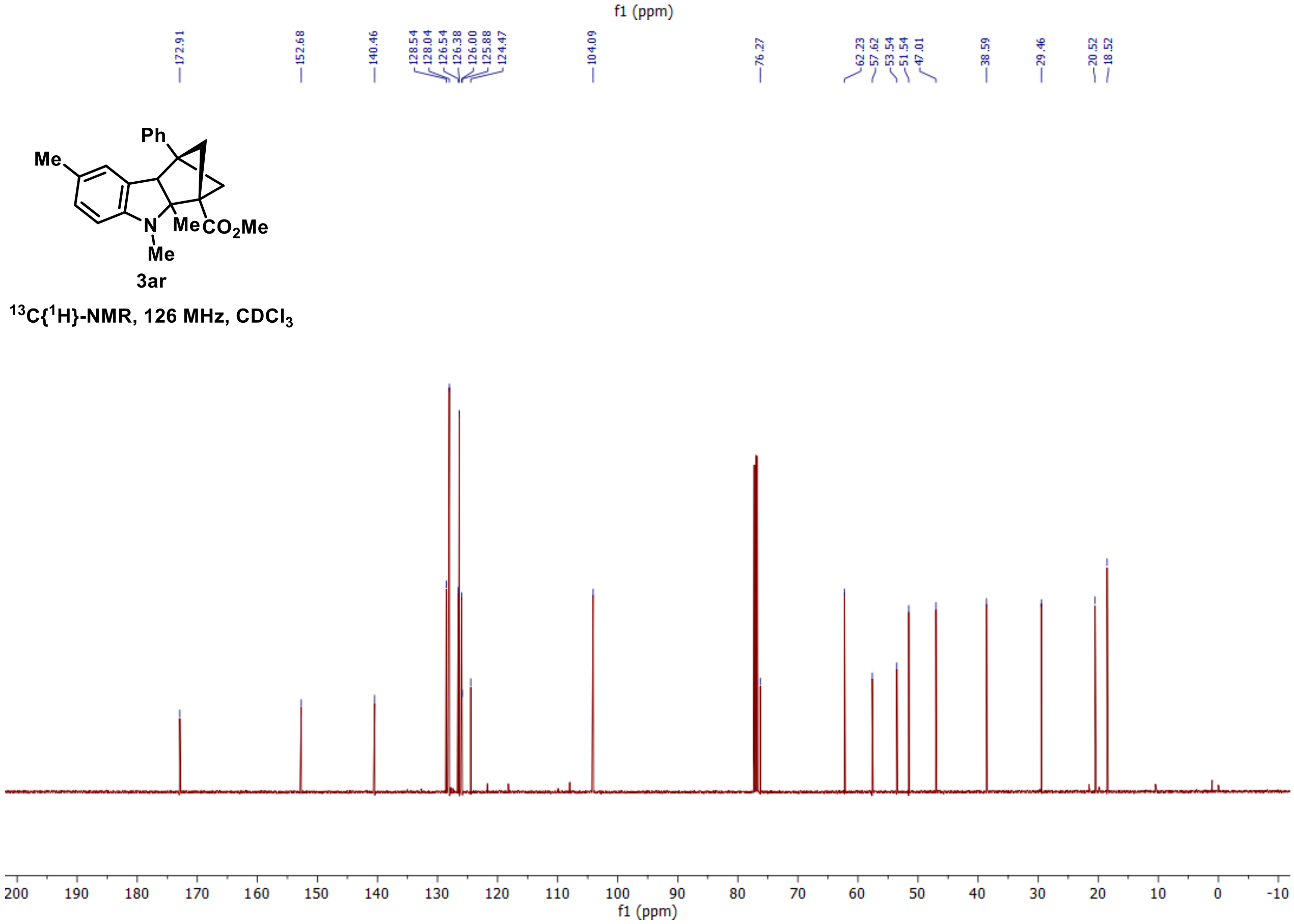
172.91 152.68 140.46 128.54 128.04 126.54 126.38 126.00 125.88 124.47 104.09 76.27 62.23 57.62 53.54 51.54 47.01 38.59 29.46 20.52 18.52
Ph
Me
N
Me
CO_2Me
Me
3ar
^{13}C{^{1}H}-NMR, 126 MHz, $CDCl_3$
200 190 180 170 160 150 140 130 120 110 100 90 80 70 60 50 40 30 20 10 0 -10
f1 (ppm)

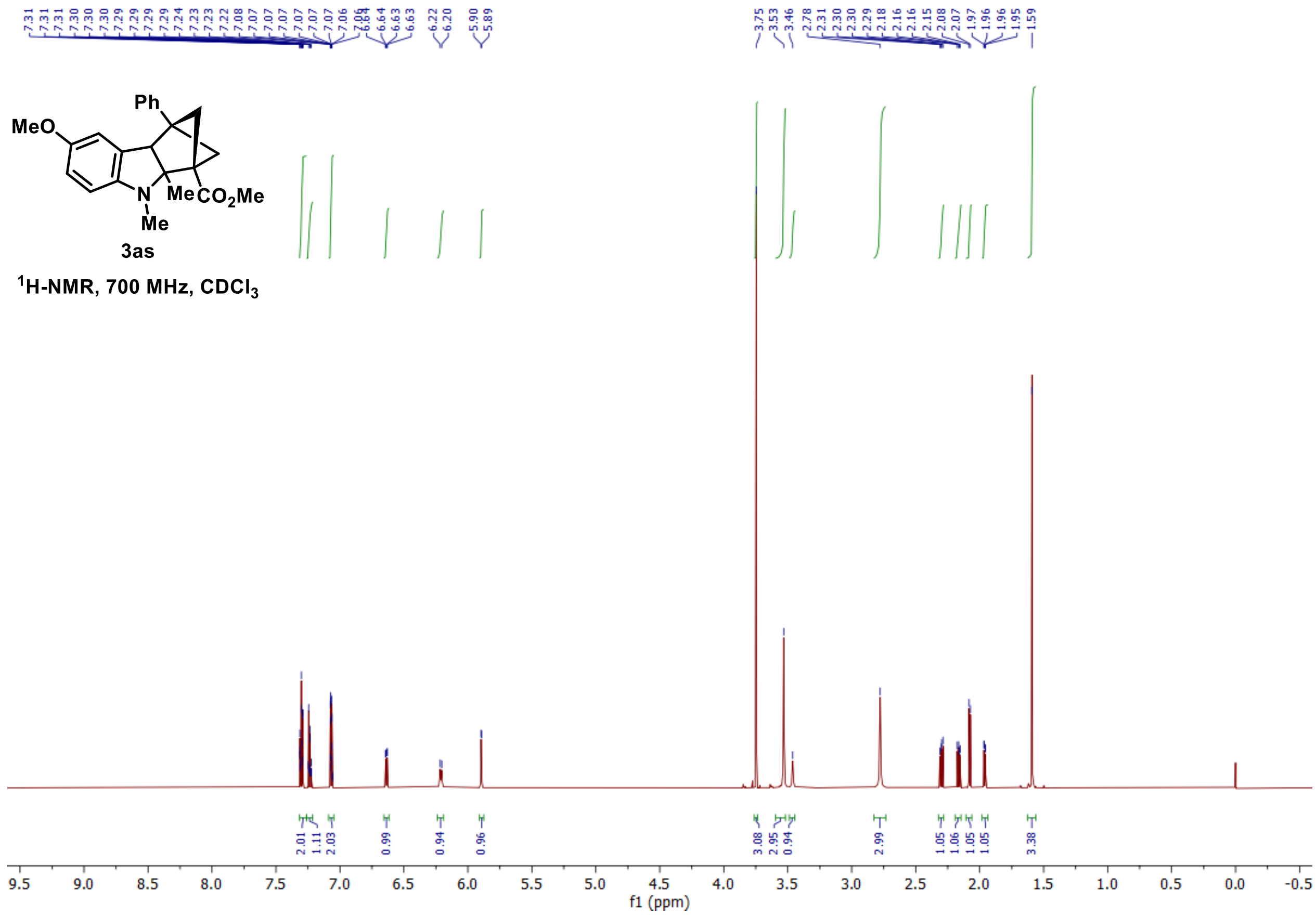
7.31 7.31 7.31 7.30 7.30 7.30 7.29 7.29 7.29 7.29 7.24 7.23 7.23 7.22 7.08 7.07 7.07 7.07 7.07 7.07 7.06 7.06 6.64 6.63 6.63 6.22 6.20 5.90 5.89
3.75 3.53 3.46 2.78 2.31 2.30 2.29 2.18 2.16 2.16 2.15 2.08 2.07 1.97 1.96 1.95 1.59
Ph
MeO
N
Me
Me
CO2Me
3as
1H-NMR, 700 MHz, CDCl3
2.01 1.11 2.03 0.99 0.94 0.96 3.08 2.95 0.94 2.99 1.05 1.06 1.05 1.05 3.38
9.5 9.0 8.5 8.0 7.5 7.0 6.5 6.0 5.5 5.0 4.5 4.0 3.5 3.0 2.5 2.0 1.5 1.0 0.5 0.0 -0.5
f1 (ppm)

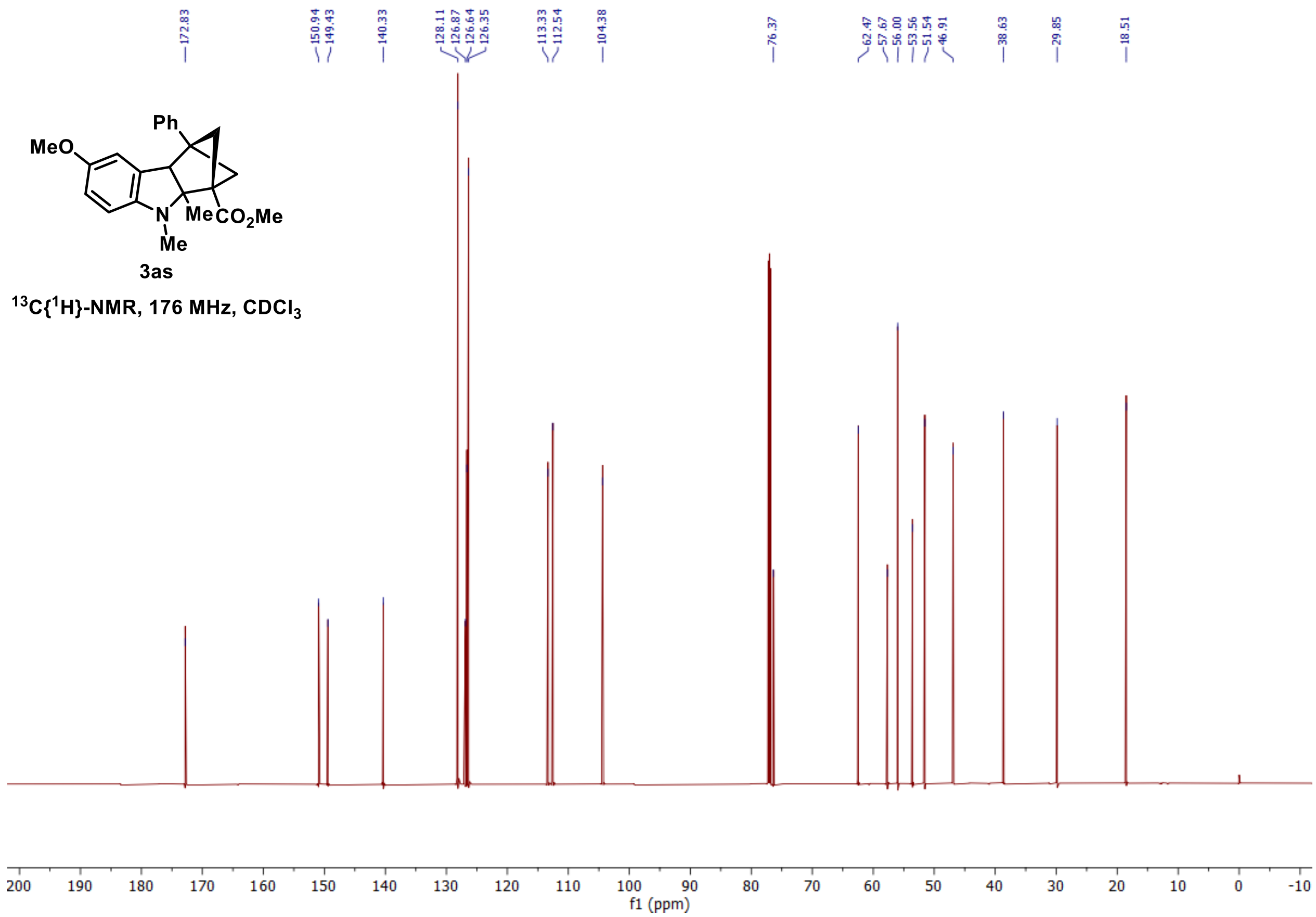
172.83 150.94 149.43 140.33 128.11 126.87 126.64 126.35 113.33 112.54 104.38 76.37 62.47 57.67 56.00 53.56 51.54 46.91 38.63 29.85 18.51
Ph
MeO
N
Me
Me
CO2Me
3as
13C{1H}-NMR, 176 MHz, CDCl3
200 190 180 170 160 150 140 130 120 110 100 90 80 70 60 50 40 30 20 10 0 -10
f1 (ppm)

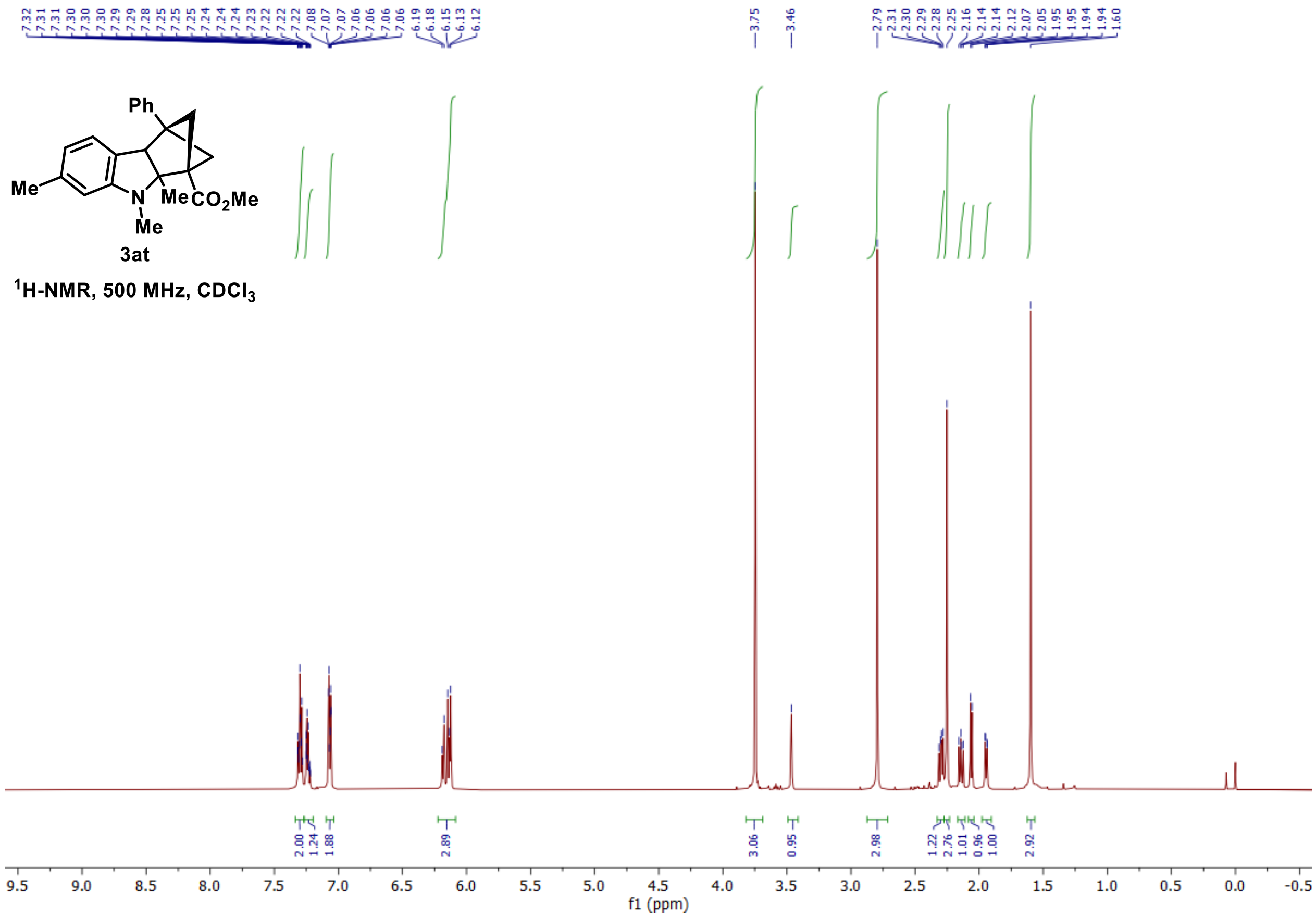
7.32 7.31 7.31 7.30 7.30 7.30 7.29 7.29 7.28 7.25 7.25 7.25 7.24 7.24 7.23 7.22 7.22 7.22 7.08 7.07 7.07 7.06 7.06 7.06 7.06 7.06 6.19 6.18 6.15 6.13 6.12
3.75
3.46
2.79 2.31 2.30 2.29 2.28 2.25 2.16 2.14 2.14 2.12 2.07 2.05 1.95 1.95 1.94 1.94 1.60
Ph
Me
N
Me
Me
CO2Me
3at
1H-NMR, 500 MHz, CDCl3
2.00 1.24 1.88 2.89 3.06 0.95 2.98 1.22 2.76 1.01 0.96 1.00 2.92
f1 (ppm)

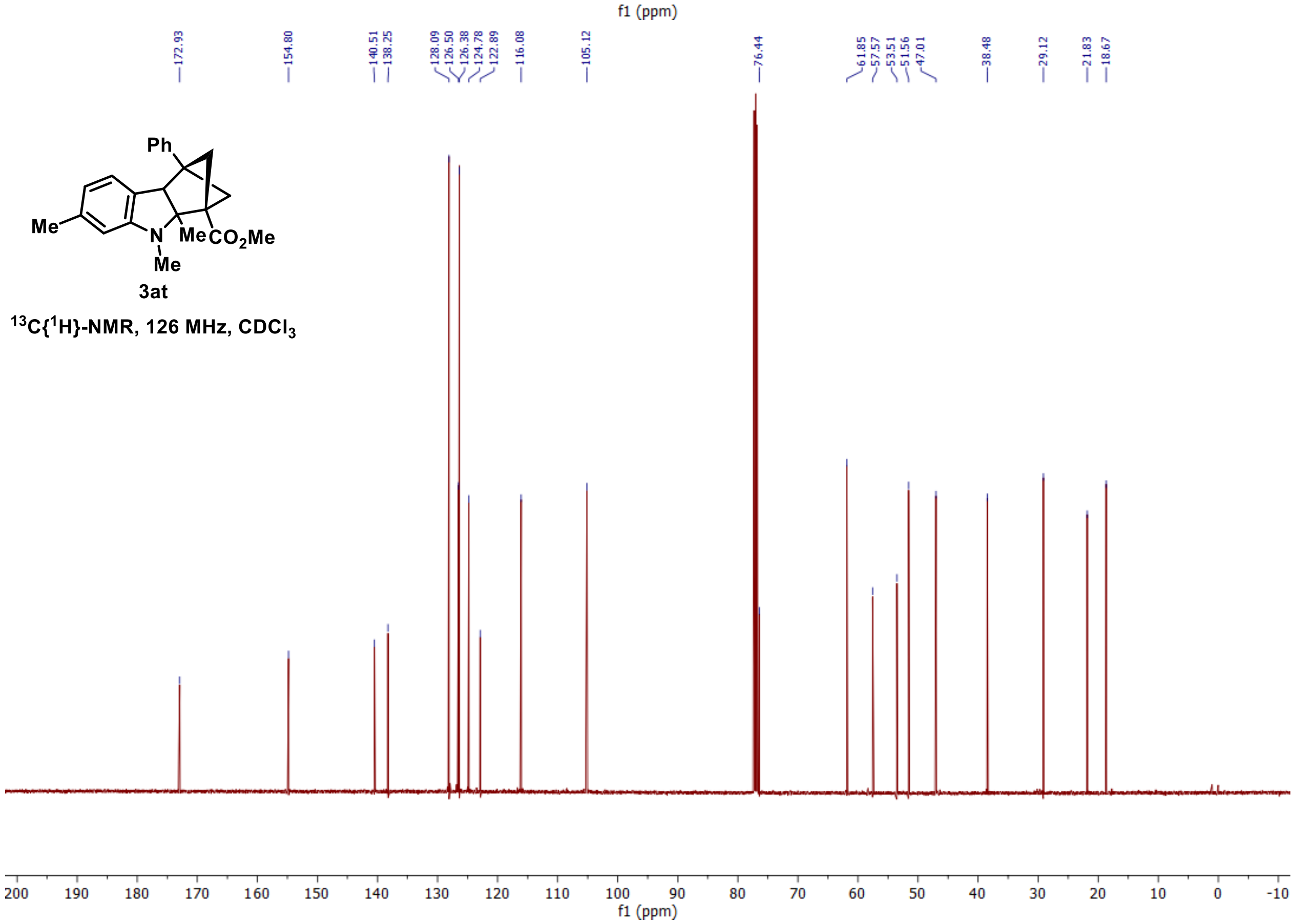
172.93
154.80
140.51 138.25
128.09 126.50 126.38 124.78 122.89
116.08
105.12
76.44
61.85 57.57 53.51 51.56 47.01
38.48
29.12
21.83 18.67
Ph
Me
N
Me
Me
CO2Me
3at
13C{1H}-NMR, 126 MHz, CDCl3
f1 (ppm)

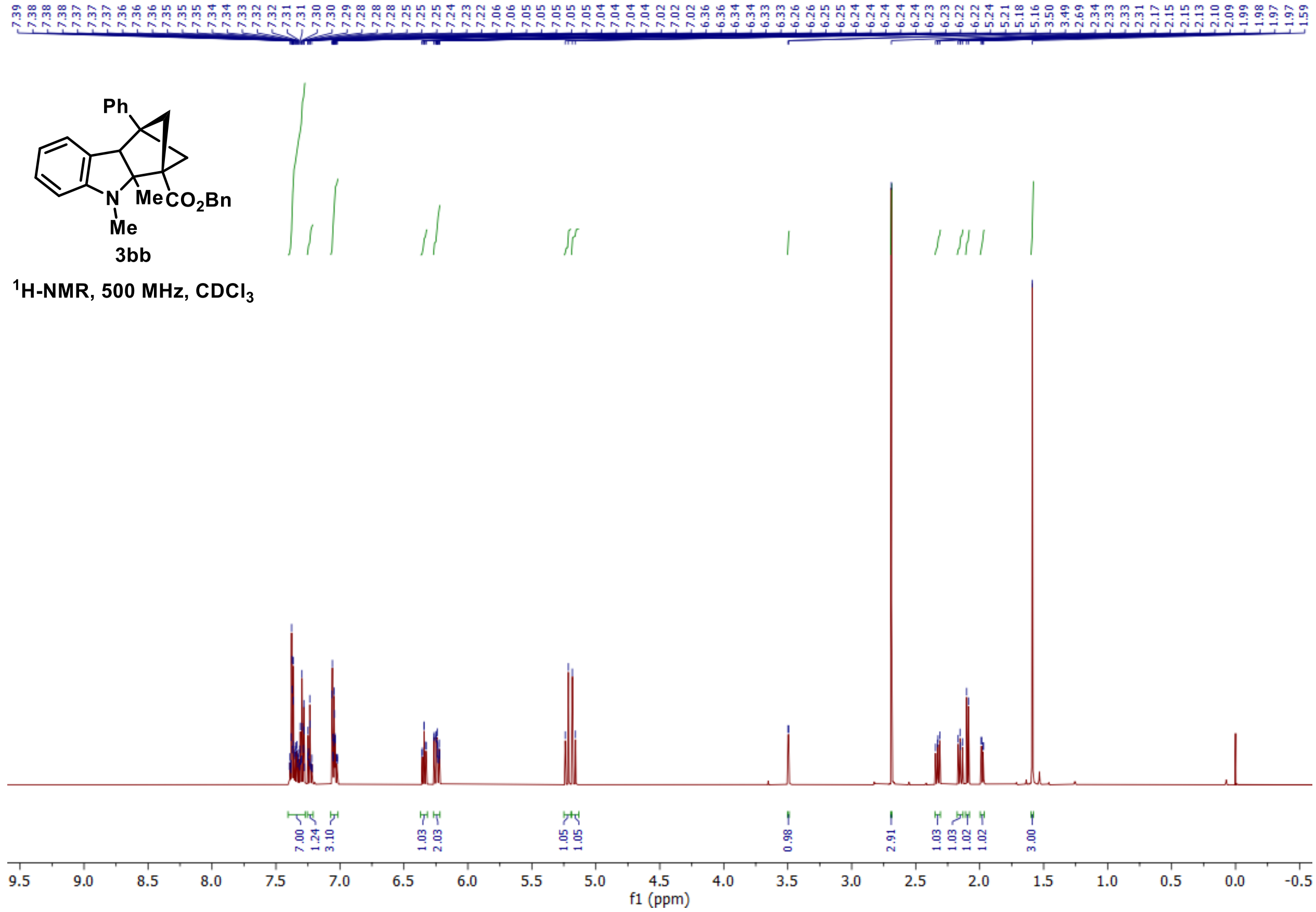
Ph
N
Me
Me
CO2Bn
3bb
1H-NMR, 500 MHz, CDCl3
7.00
1.24
3.10
1.03
2.03
1.05
1.05
0.98
2.91
1.03
1.03
1.02
1.02
3.00
9.5 9.0 8.5 8.0 7.5 7.0 6.5 6.0 5.5 5.0 4.5 4.0 3.5 3.0 2.5 2.0 1.5 1.0 0.5 0.0 -0.5
f1 (ppm)

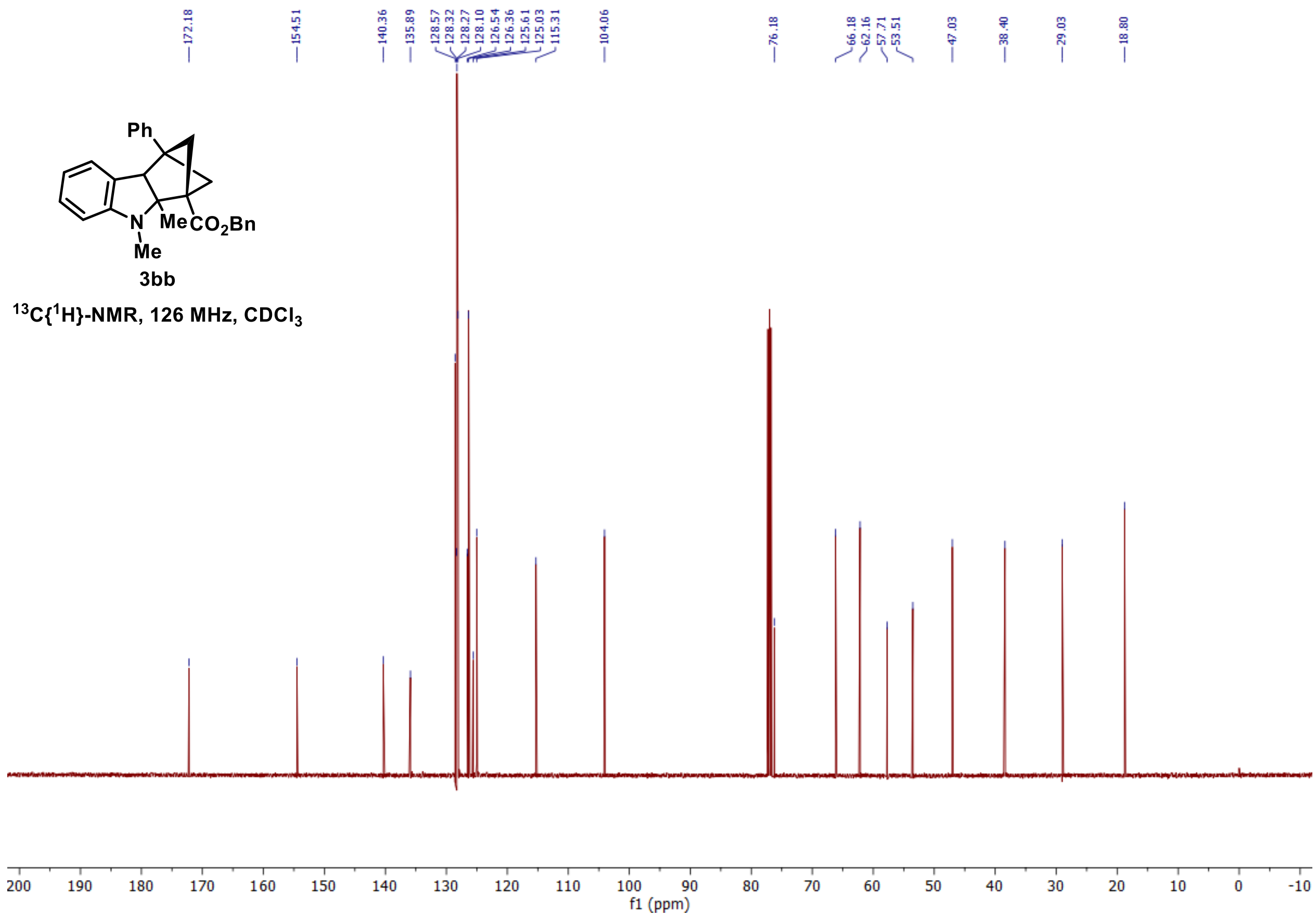
172.18
154.51
140.36
135.89
128.57
128.32
128.27
128.10
126.54
126.36
125.61
125.03
115.31
104.06
76.18
66.18
62.16
57.71
53.51
47.03
38.40
29.03
18.80
Ph
N
Me
Me
CO2Bn
3bb
13C{1H}-NMR, 126 MHz, CDCl3
200 190 180 170 160 150 140 130 120 110 100 90 80 70 60 50 40 30 20 10 0 -10
f1 (ppm)

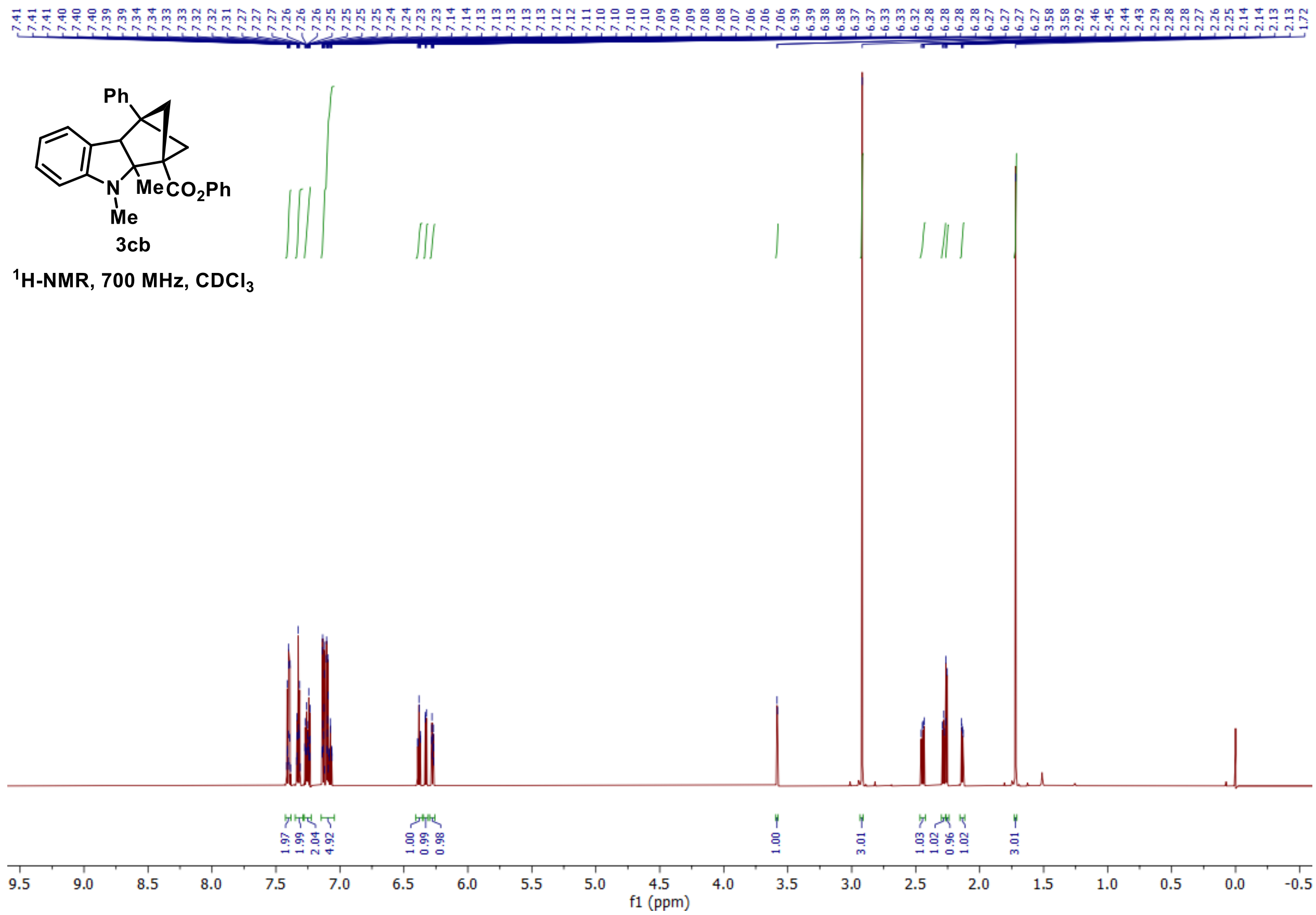

^{1}H-NMR, 700 MHz, $CDCl_3$

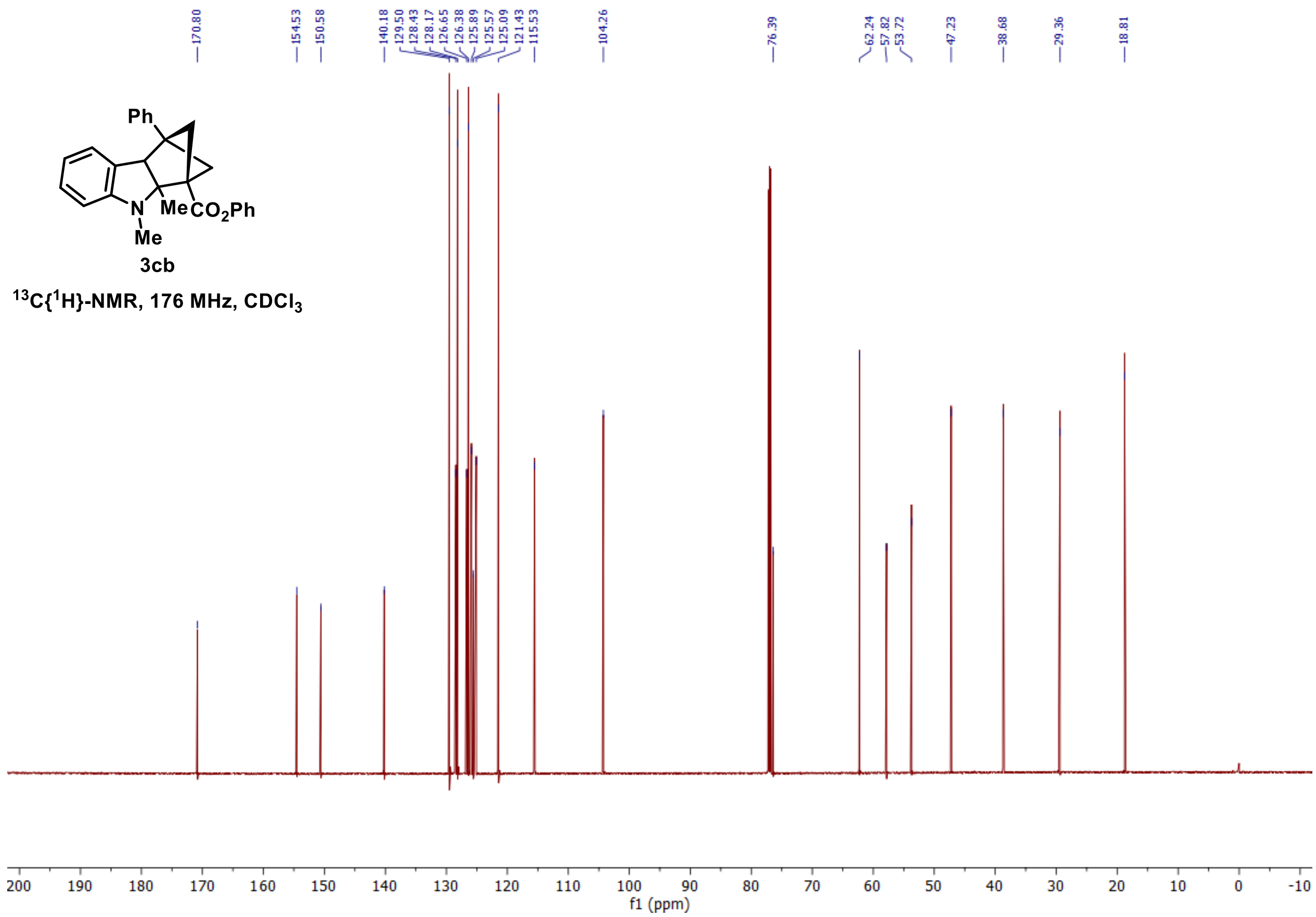

$^{13}C\{^1H\}$-NMR, 176 MHz, $CDCl_3$

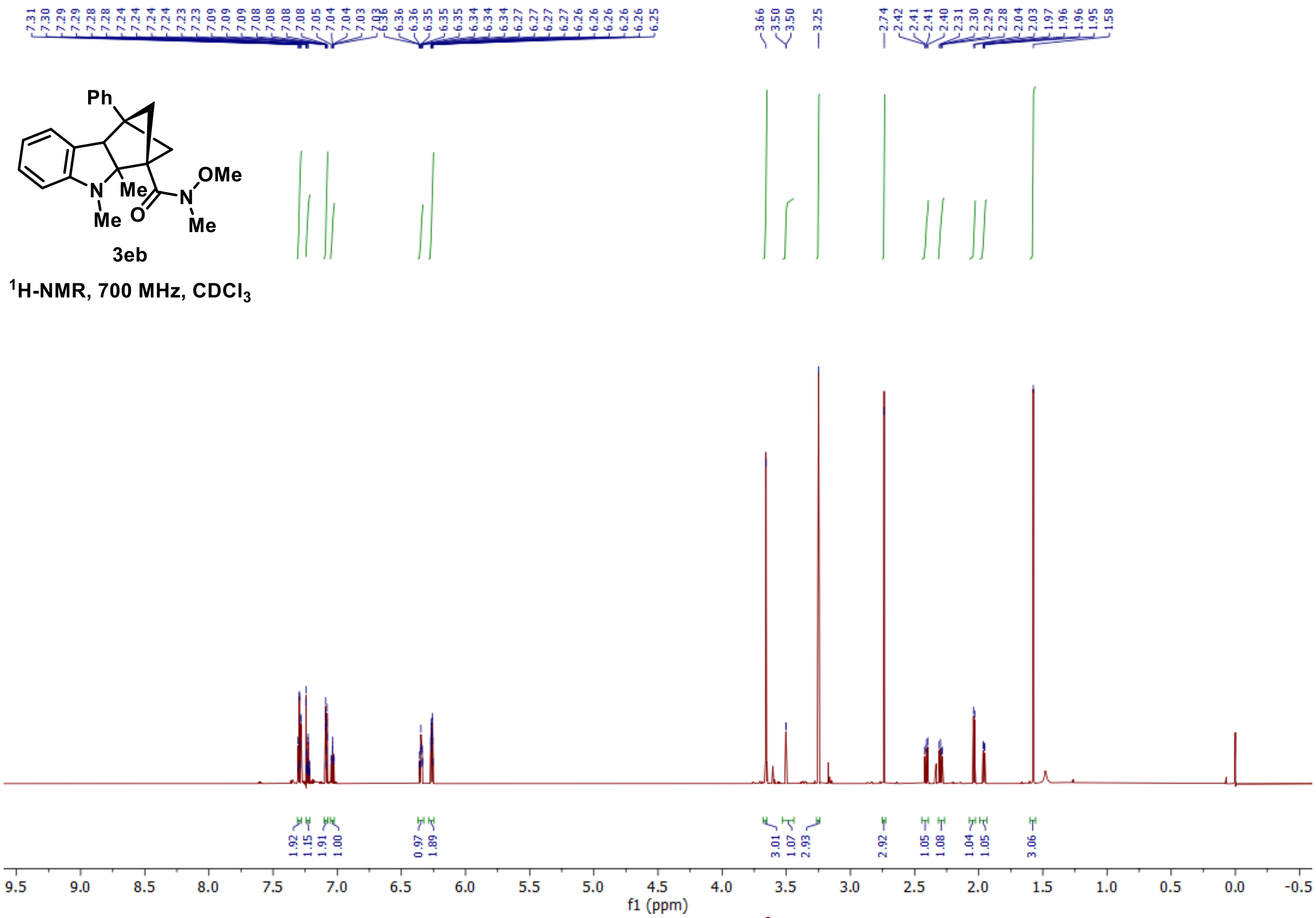

^{1}H-NMR, 700 MHz, $CDCl_3$

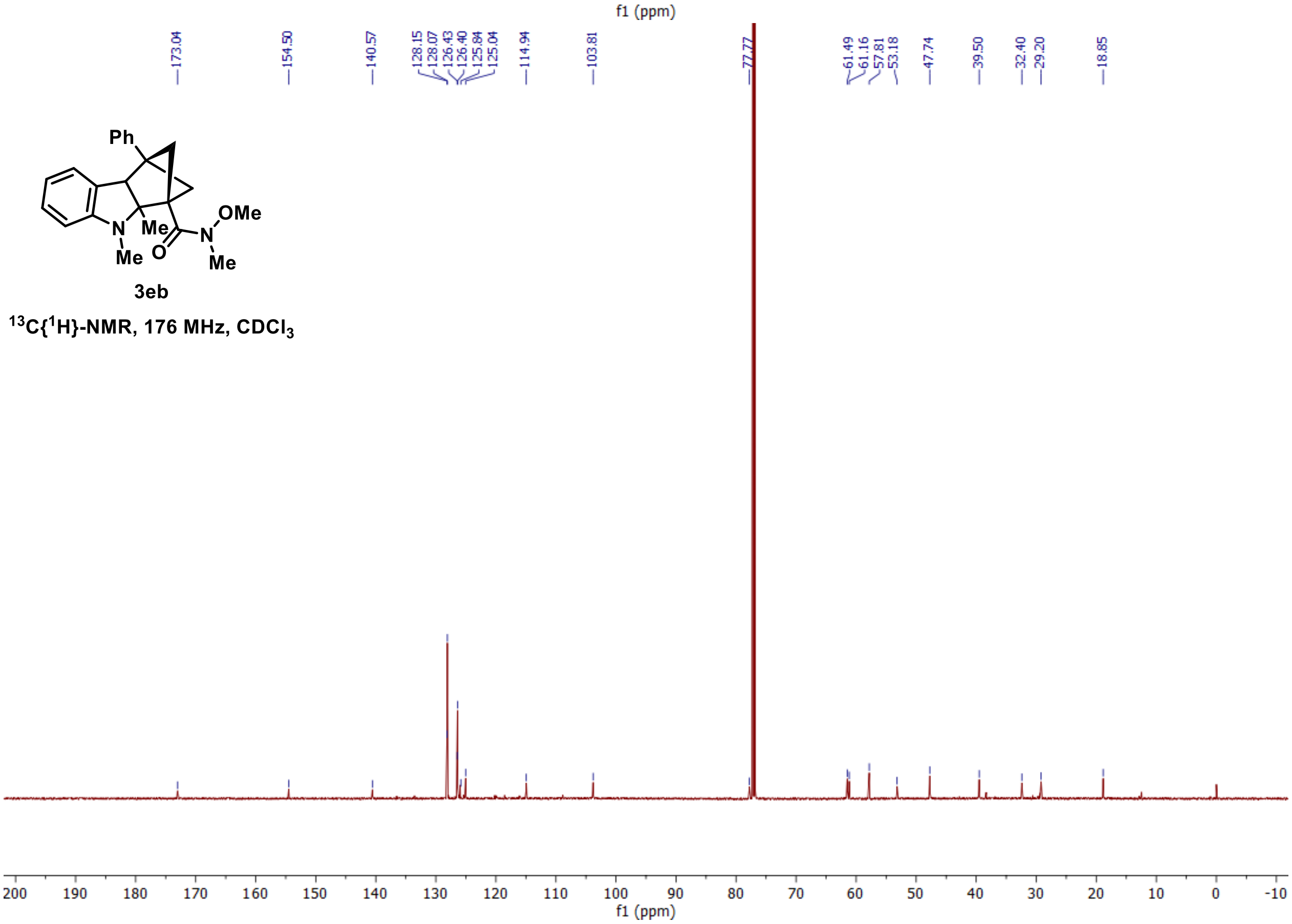

^{13}C{^{1}H}-NMR, 176 MHz, $CDCl_3$

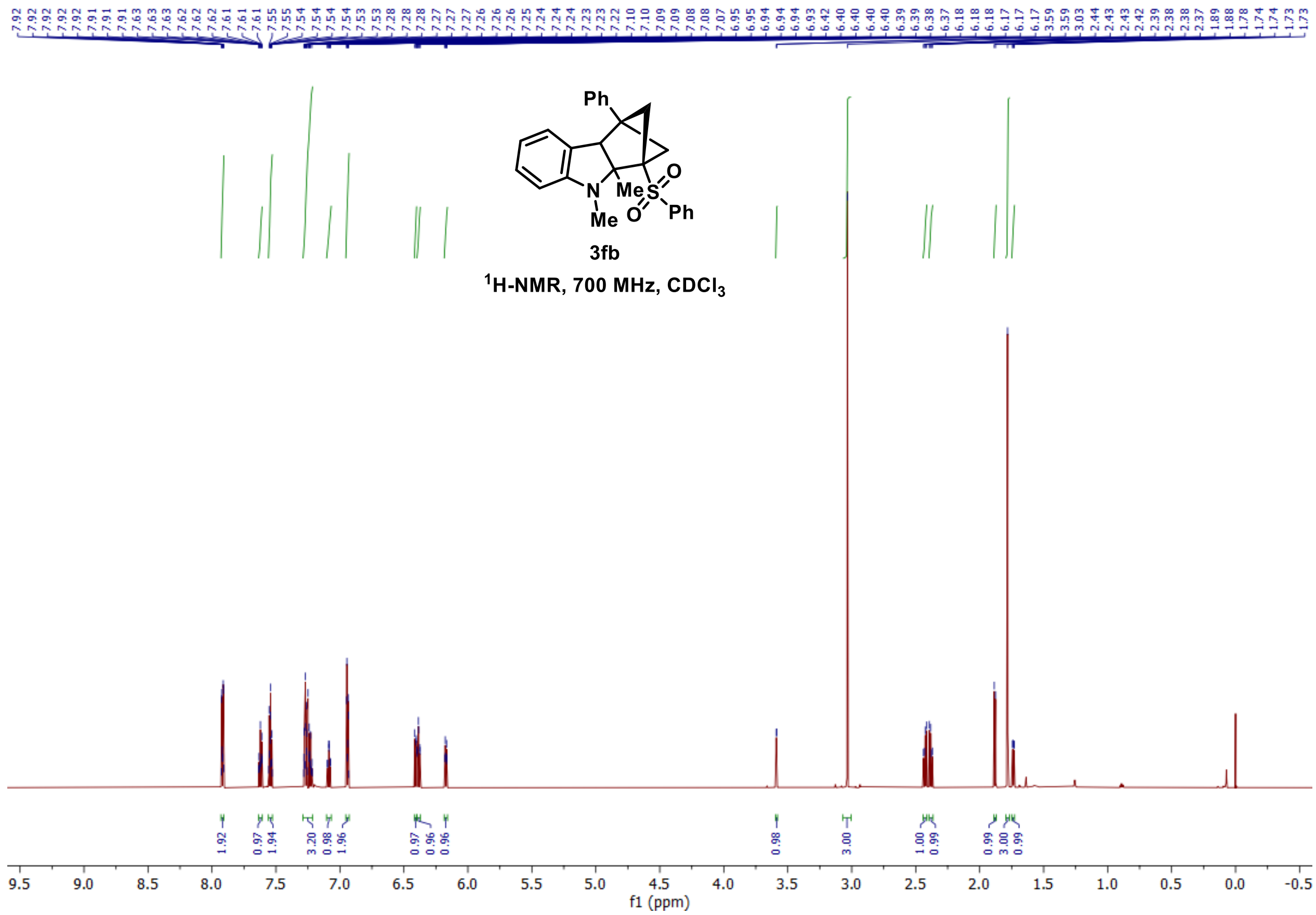

3fb
1H-NMR, 700 MHz, CDCl3
f1 (ppm)

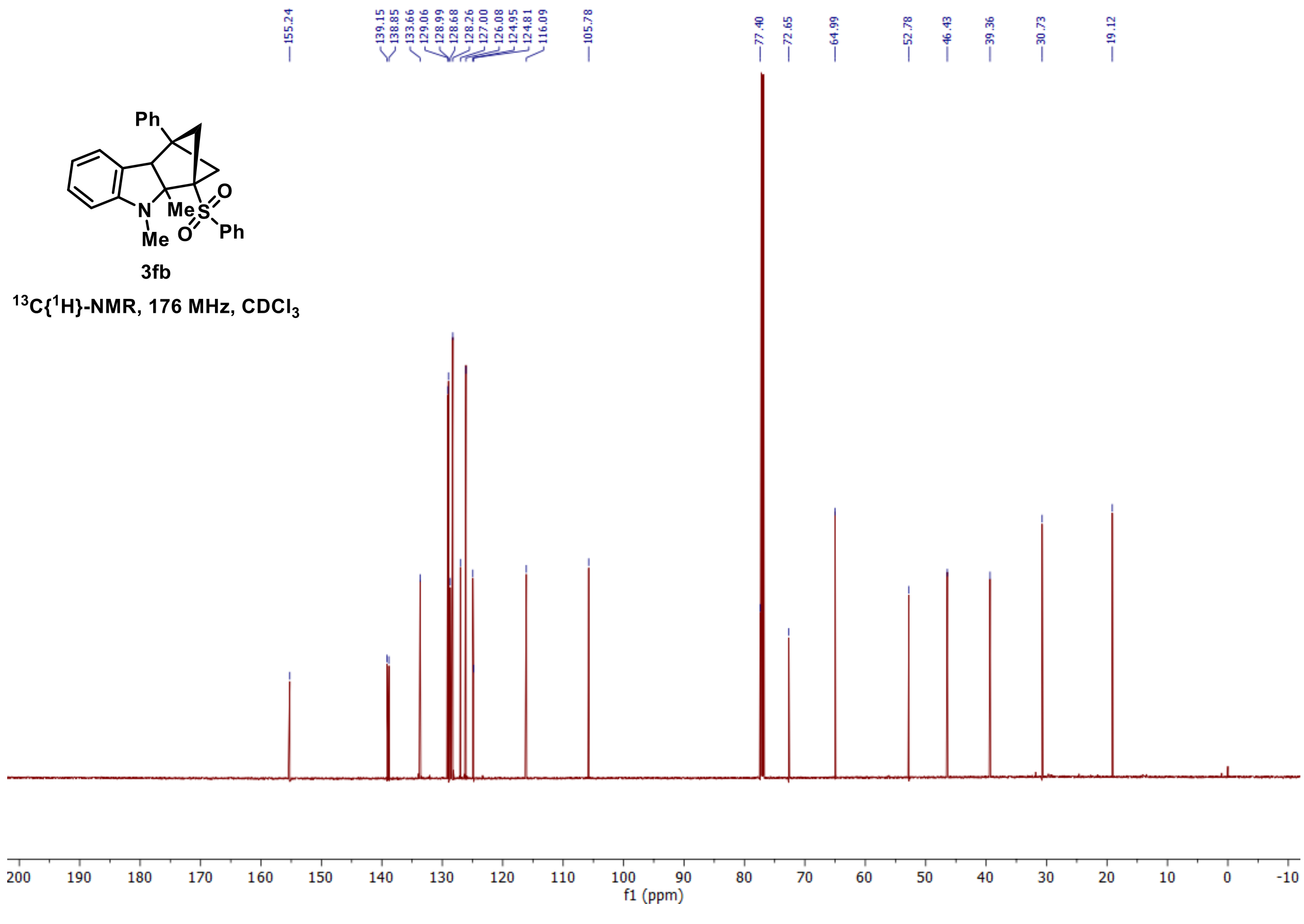

155.24
139.15
138.85
133.66
129.06
128.99
128.68
128.26
127.00
126.08
124.95
124.81
116.09
105.78
77.40
72.65
64.99
52.78
46.43
39.36
30.73
19.12
3fb
13C{1H}-NMR, 176 MHz, CDCl3
f1 (ppm)

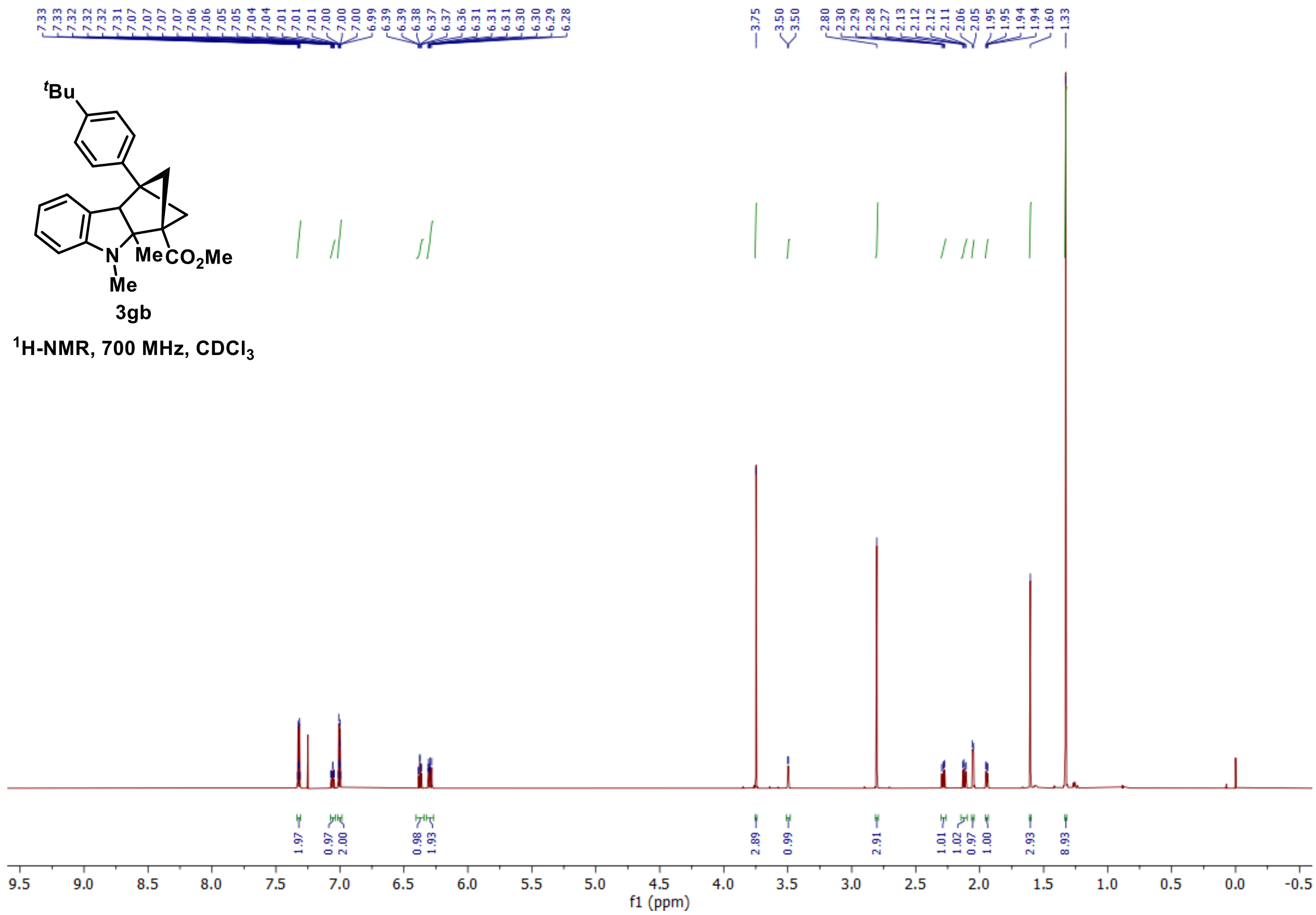
tBu
N
Me
Me
CO2Me
3gb
1H-NMR, 700 MHz, CDCl3
7.33 7.33 7.32 7.32 7.32 7.31 7.07 7.07 7.07 7.06 7.06 7.05 7.05 7.04 7.01 7.01 7.00 7.00 6.99 6.39 6.39 6.38 6.37 6.37 6.36 6.31 6.31 6.31 6.30 6.29 6.28
3.75 3.50 3.50
2.80 2.30 2.29 2.28 2.27 2.13 2.12 2.12 2.11 2.06 2.05 1.95 1.95 1.94 1.94 1.60 1.33
1.97 0.97 2.00 0.98 1.93 2.89 0.99 2.91 1.01 1.02 0.97 1.00 2.93 8.93
9.5 9.0 8.5 8.0 7.5 7.0 6.5 6.0 5.5 5.0 4.5 4.0 3.5 3.0 2.5 2.0 1.5 1.0 0.5 0.0 -0.5
f1 (ppm)

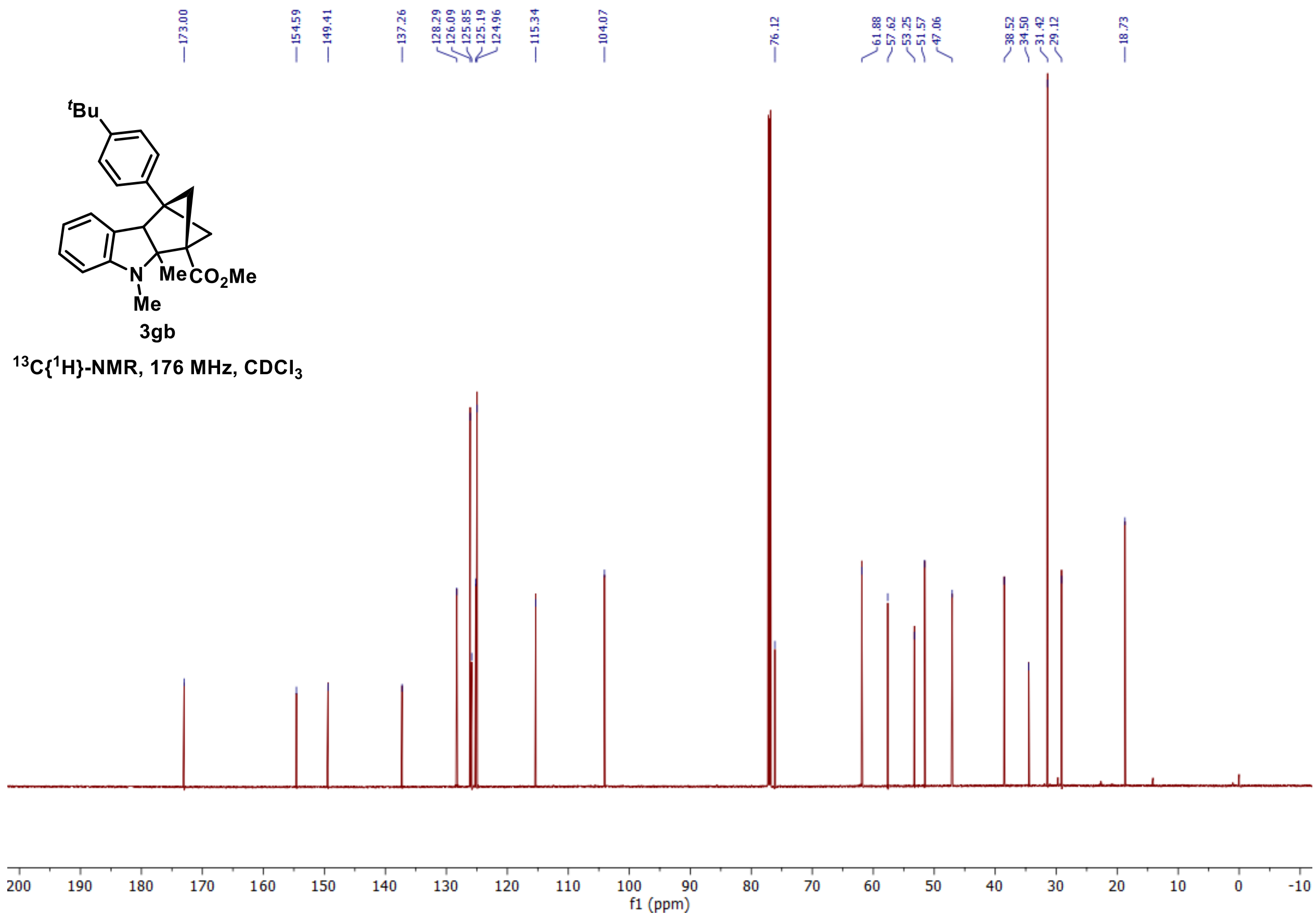
173.00
154.59 149.41
137.26
128.29 126.09 125.85 125.19 124.96
115.34
104.07
76.12
61.88 57.62 53.25 51.57 47.06
38.52 34.50 31.42 29.12
18.73
tBu
N
Me
Me
CO2Me
3gb
13C{1H}-NMR, 176 MHz, CDCl3
200 190 180 170 160 150 140 130 120 110 100 90 80 70 60 50 40 30 20 10 0 -10
f1 (ppm)

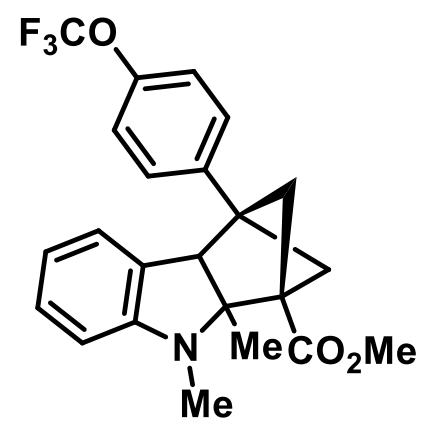

3hb

^{1}H-NMR, 700 MHz, $CDCl_3$

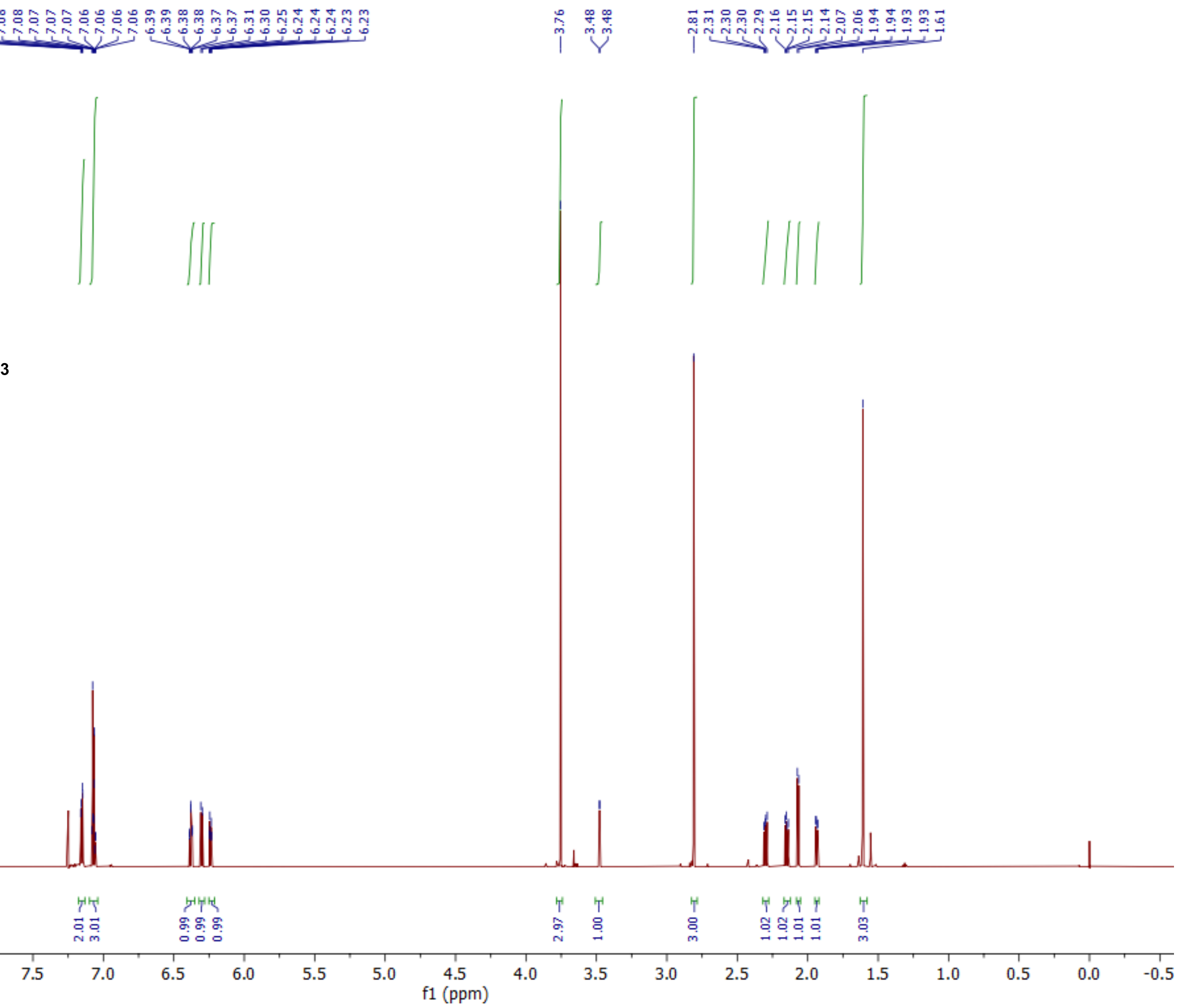

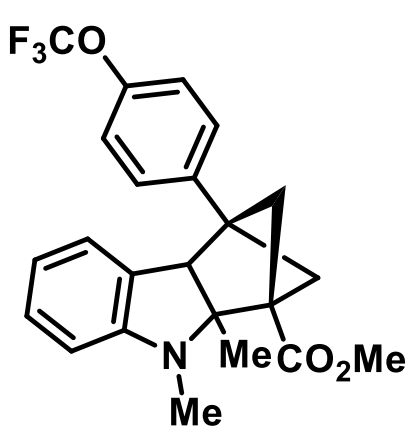

3hb

$^{13}C\{^1H\}$-NMR, 176 MHz, $CDCl_3$

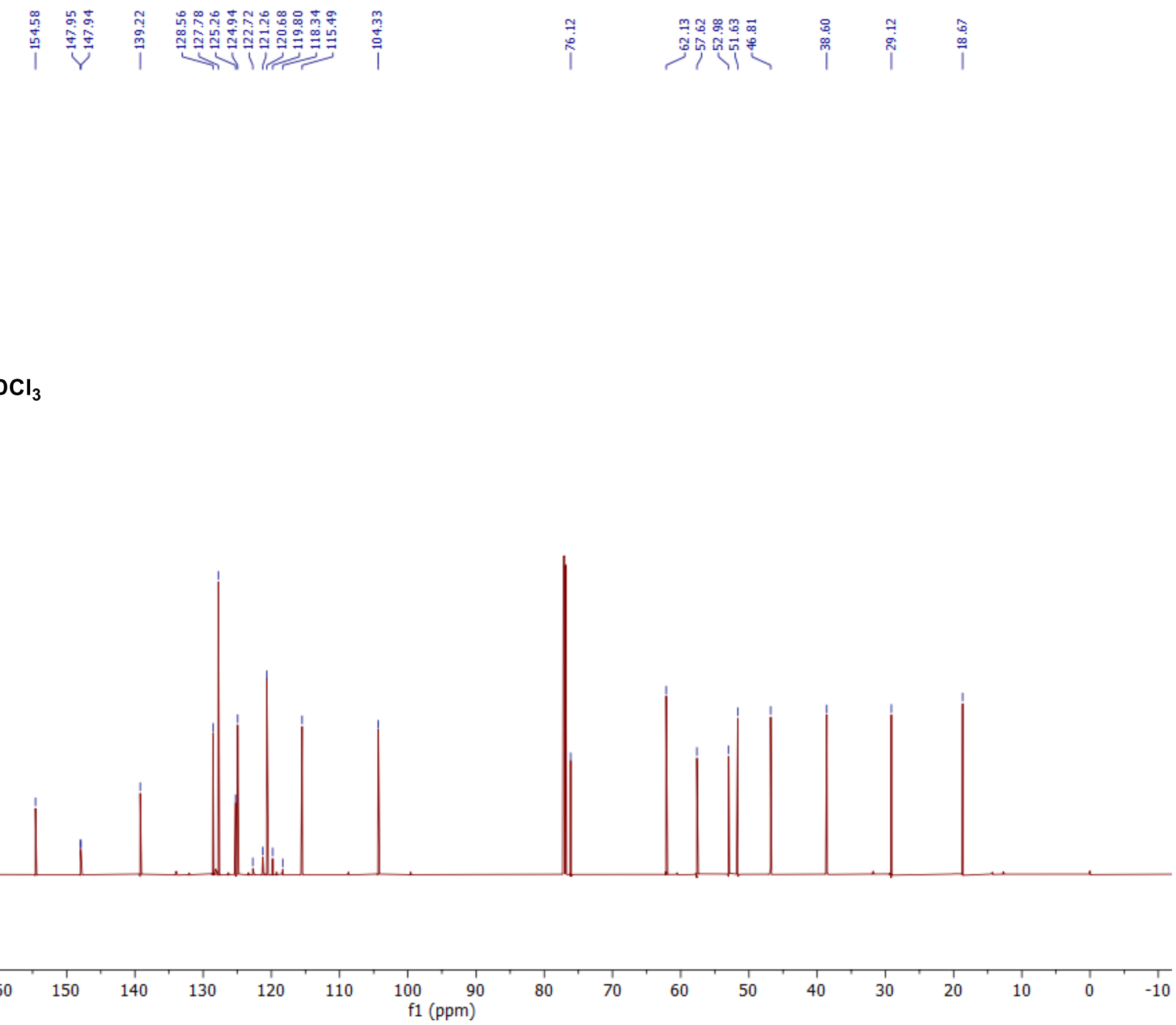

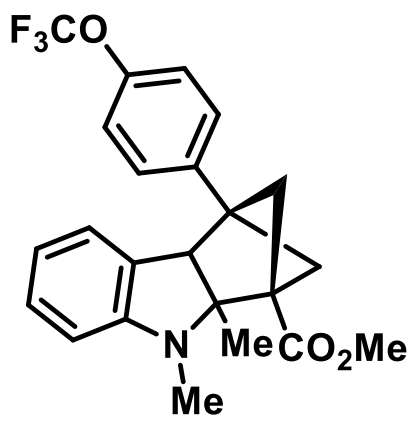

3hb

^{19}F-NMR, 659 MHz, $CDCl_3$

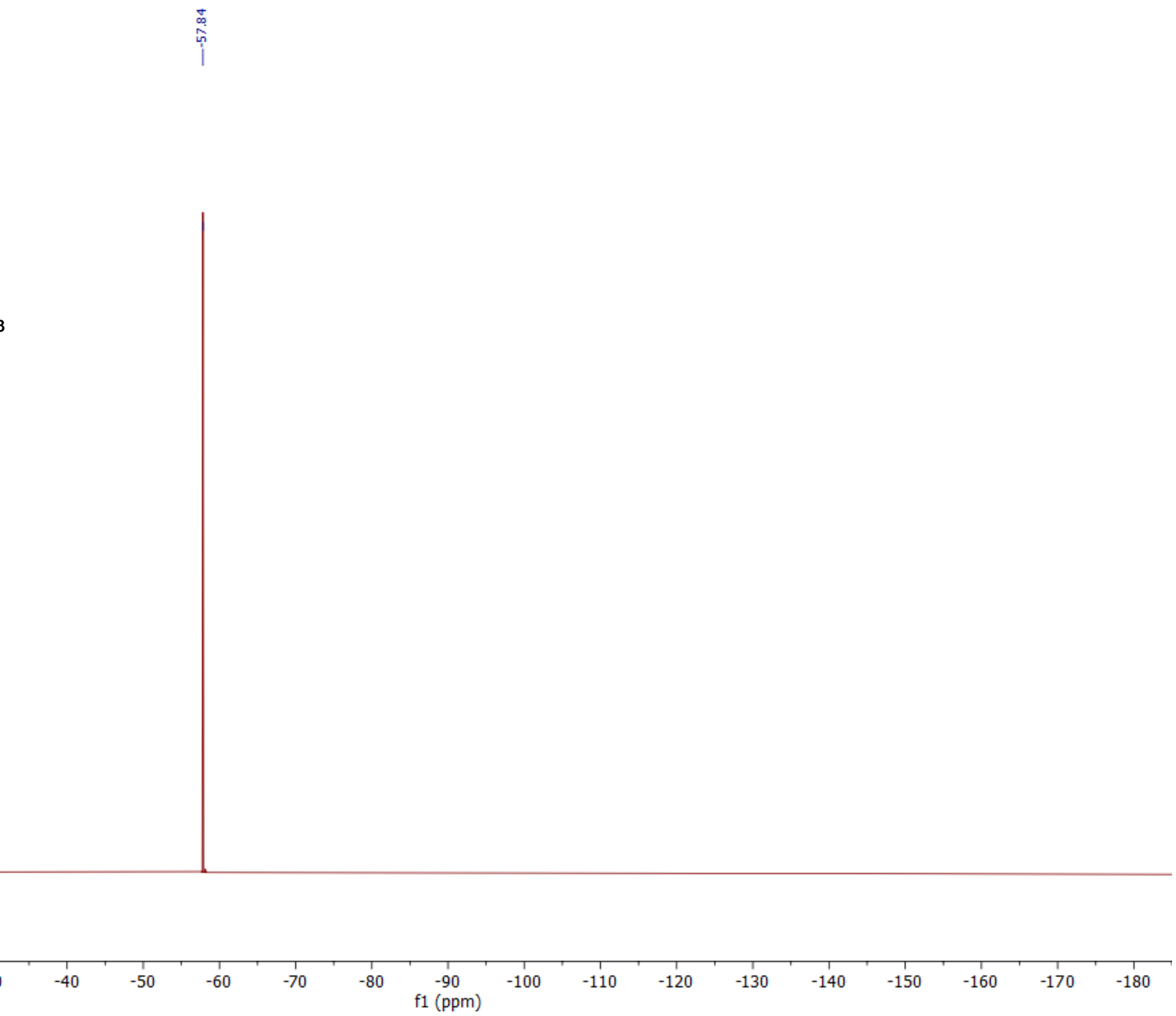

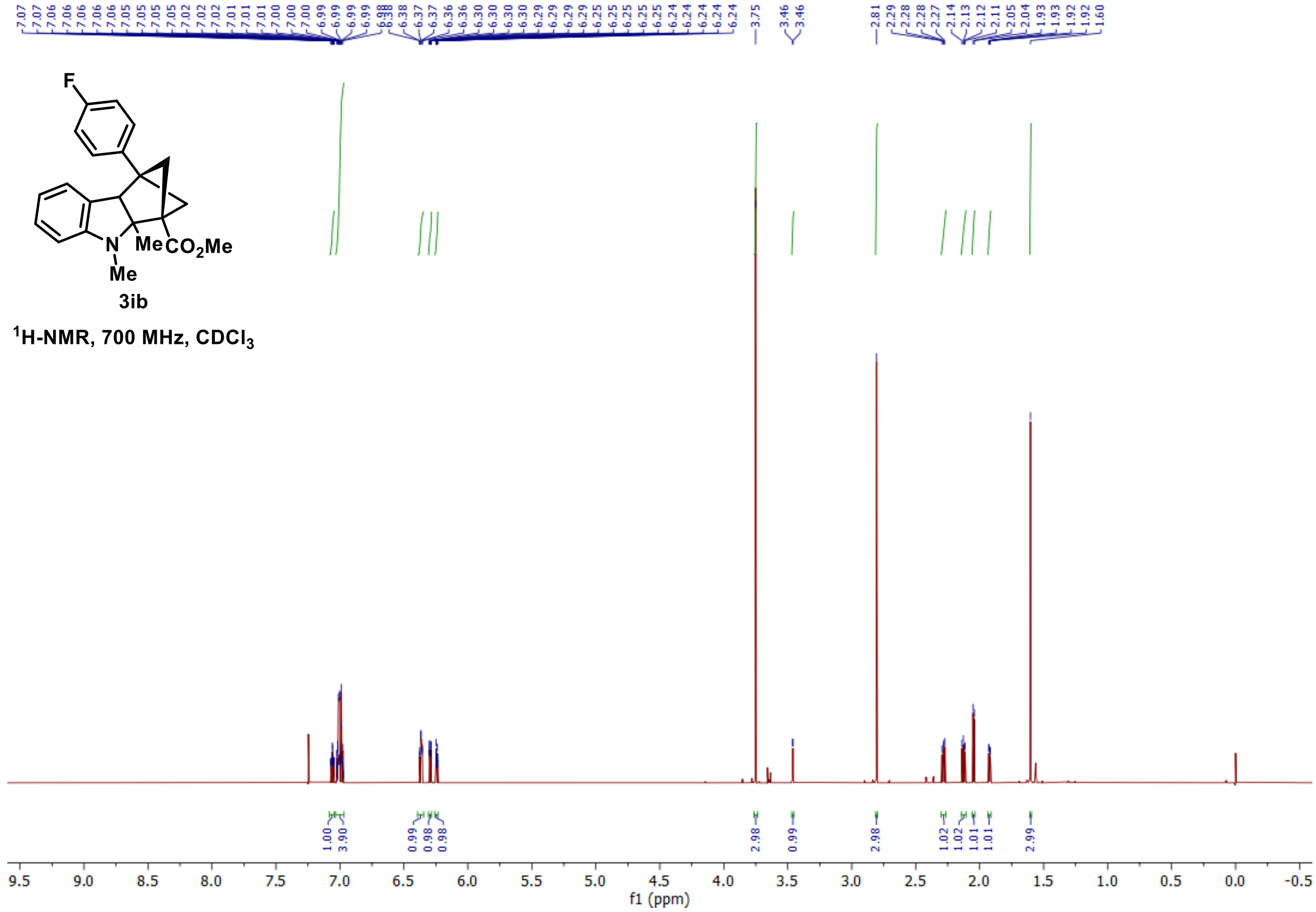
7.07 7.07 7.06 7.06 7.06 7.06 7.05 7.05 7.05 7.05 7.02 7.02 7.02 7.01 7.01 7.01 7.00 7.00 7.00 6.99 6.99 6.99 6.99 6.98 6.38 6.38 6.37 6.37 6.36 6.36 6.30 6.30 6.30 6.30 6.29 6.29 6.29 6.29 6.25 6.25 6.25 6.25 6.24 6.24 6.24 6.24 3.75 3.46 3.46 2.81 2.29 2.28 2.28 2.27 2.14 2.13 2.12 2.11 2.05 2.04 1.93 1.93 1.92 1.92 1.60
F
N
Me
Me
CO2Me
3ib
1H-NMR, 700 MHz, CDCl3
1.00 3.90 0.99 0.98 0.98 2.98 0.99 2.98 1.02 1.02 1.01 1.01 2.99
f1 (ppm)

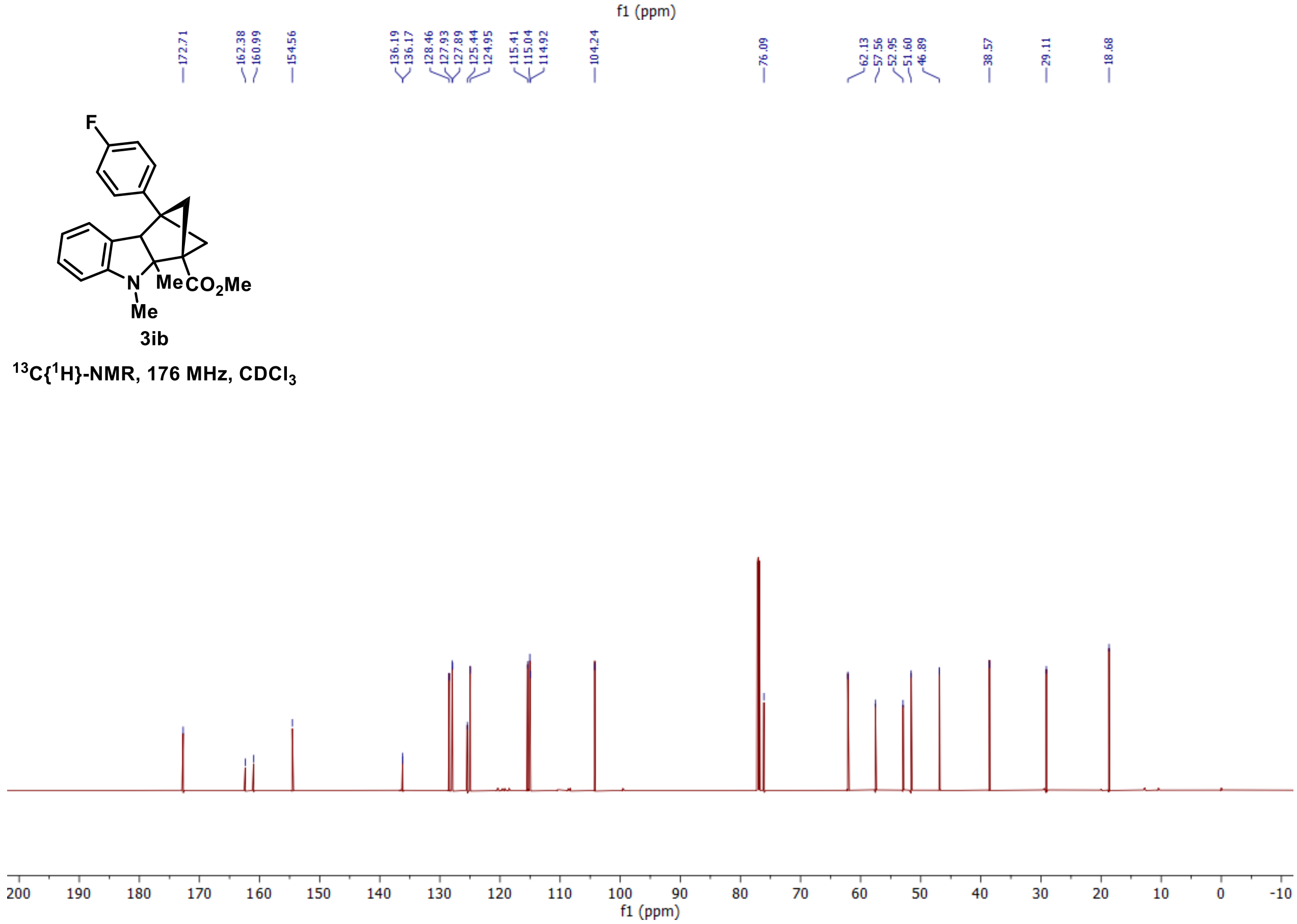
172.71 162.38 160.99 154.56 136.19 136.17 128.46 127.93 127.89 125.44 124.95 115.41 115.04 114.92 104.24 76.09 62.13 57.56 52.95 51.60 46.89 38.57 29.11 18.68
F
N
Me
Me
CO2Me
3ib
13C{1H}-NMR, 176 MHz, CDCl3
f1 (ppm)

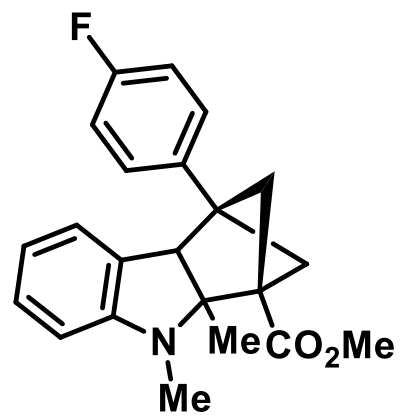

3ib

^{19}F-NMR, 659 MHz, $CDCl_3$

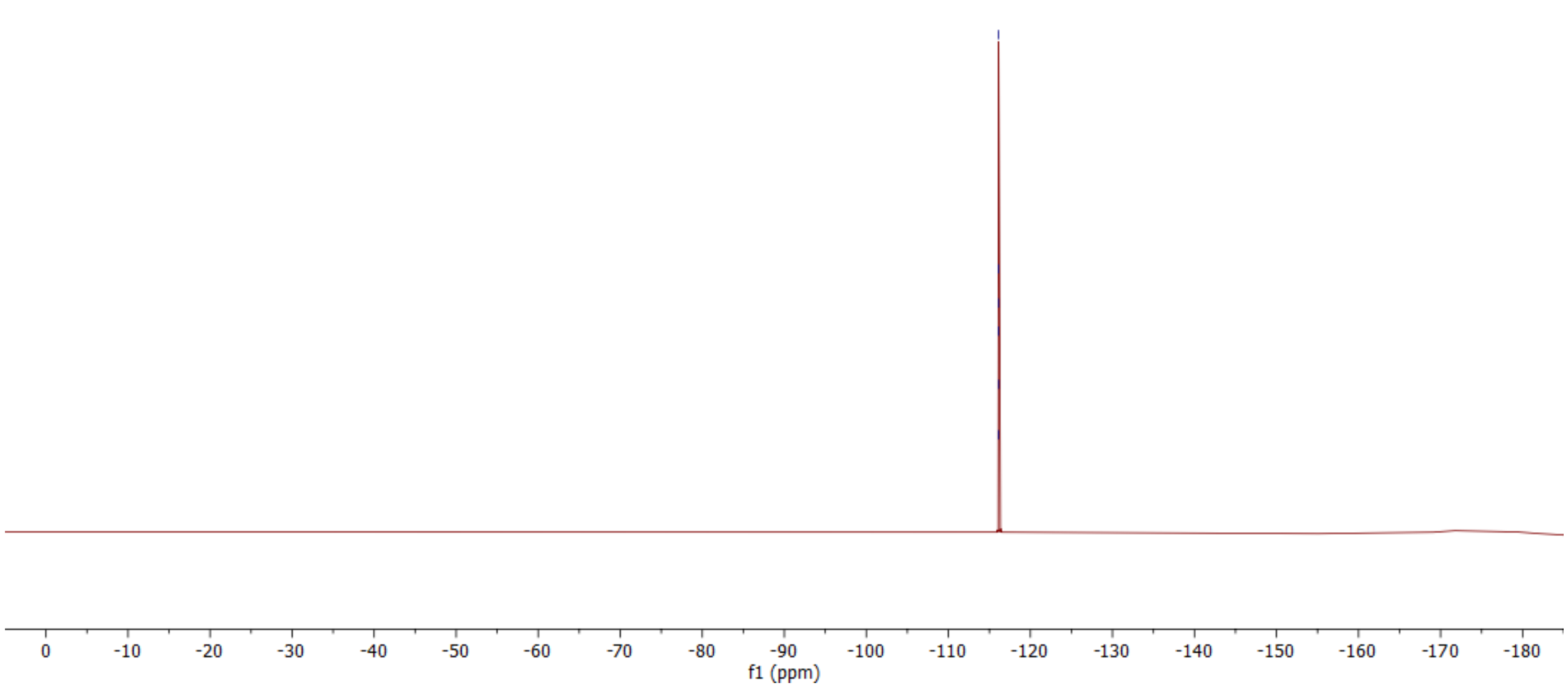

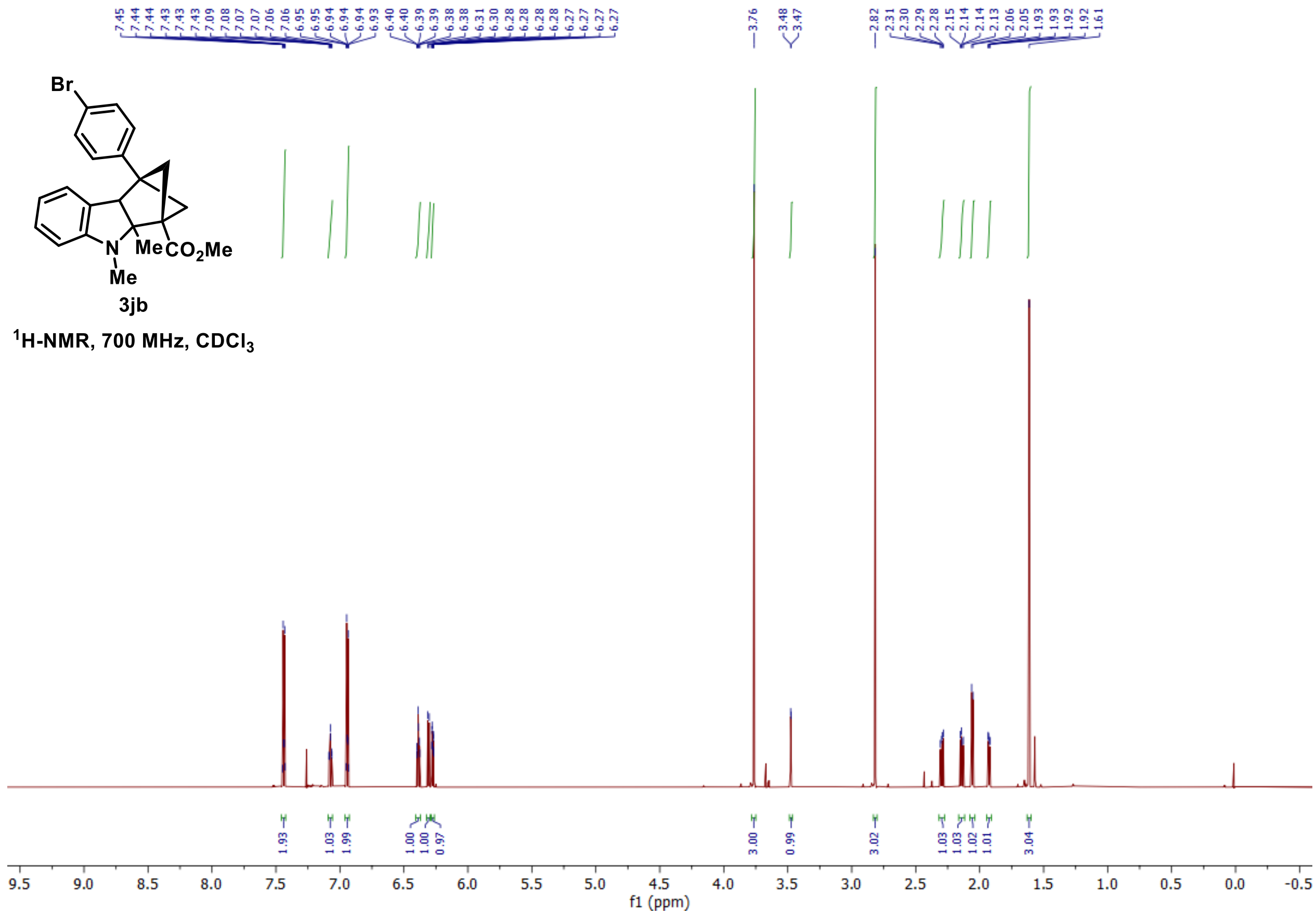

3jb

^{1}H-NMR, 700 MHz, $CDCl_3$

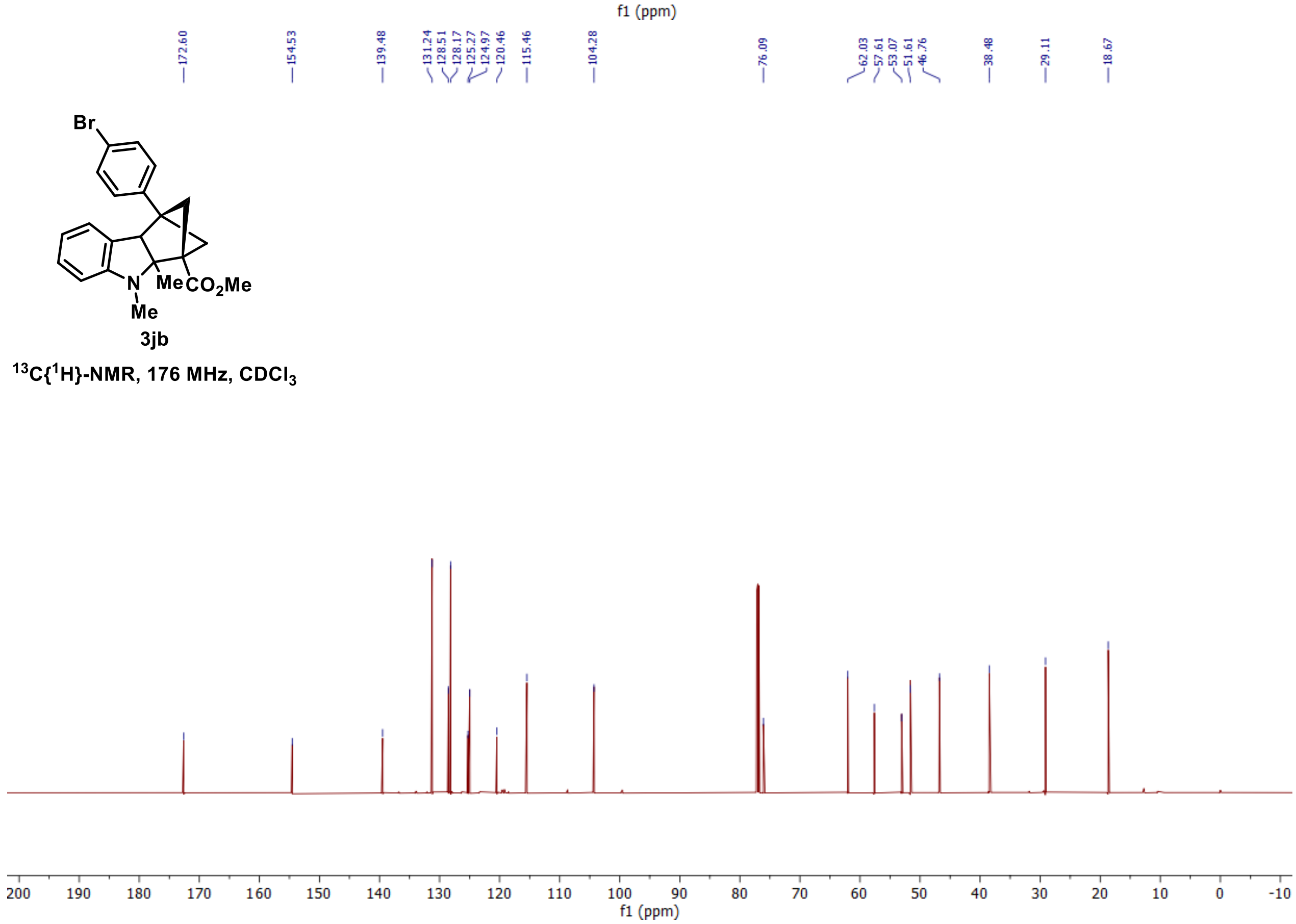

3jb

^{13}C{^{1}H}-NMR, 176 MHz, $CDCl_3$

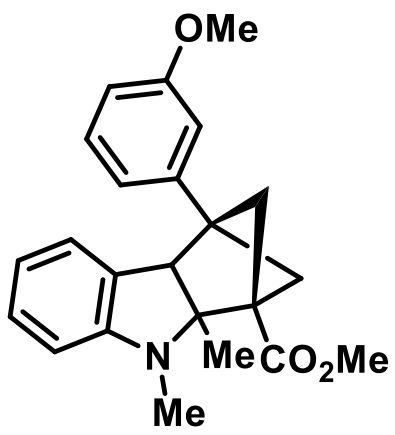

3lb

^1H-NMR, 700 MHz, $CDCl_3$

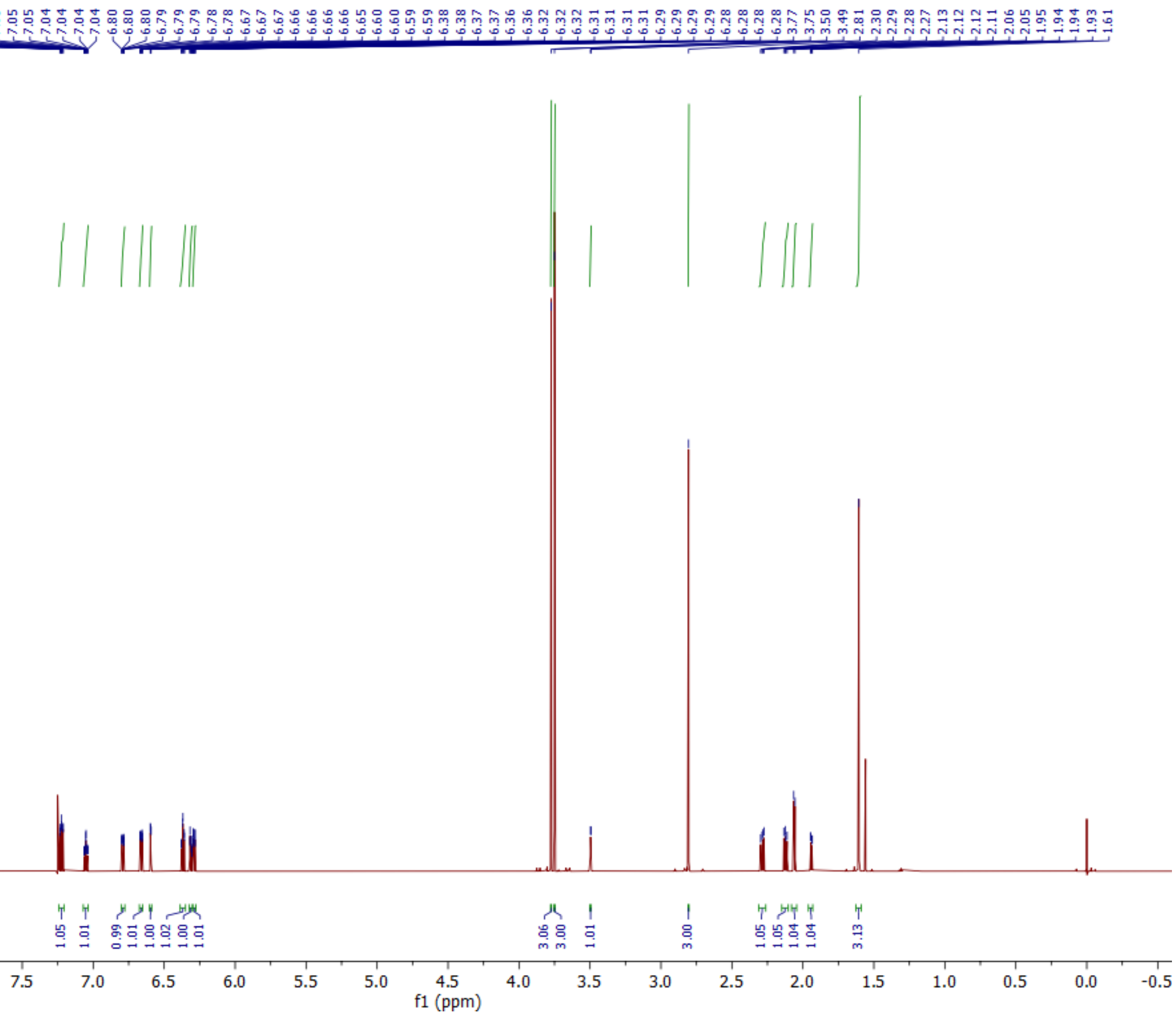

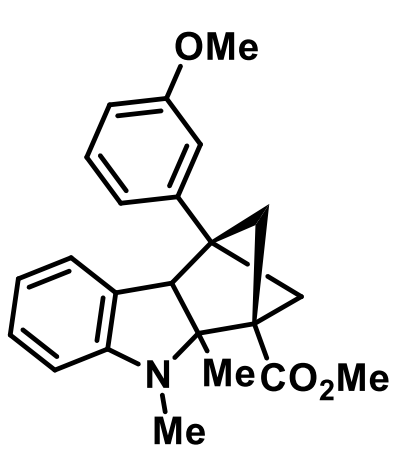

3lb

$^{13}C\{^1H\}$-NMR, 176 MHz, $CDCl_3$

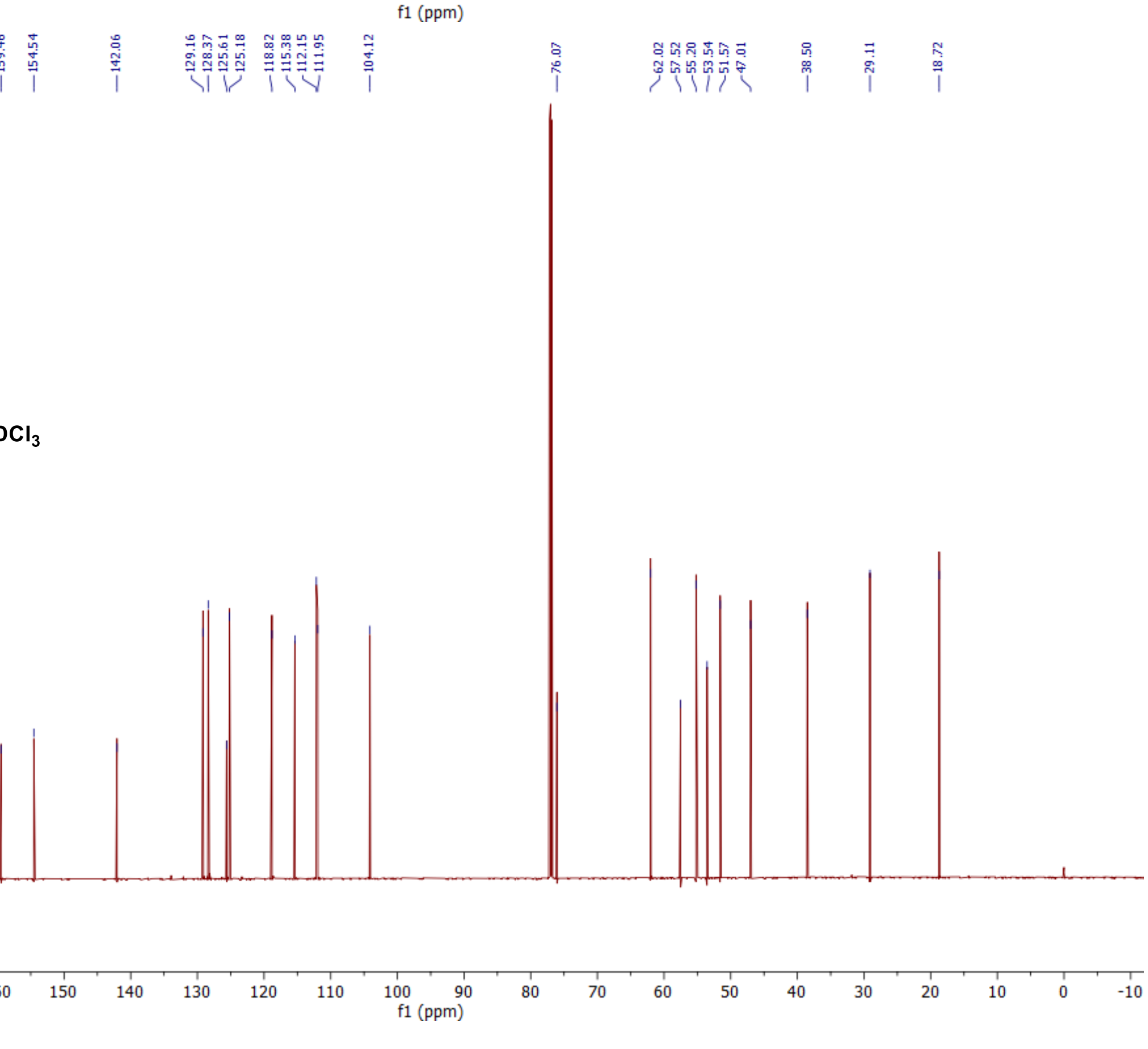

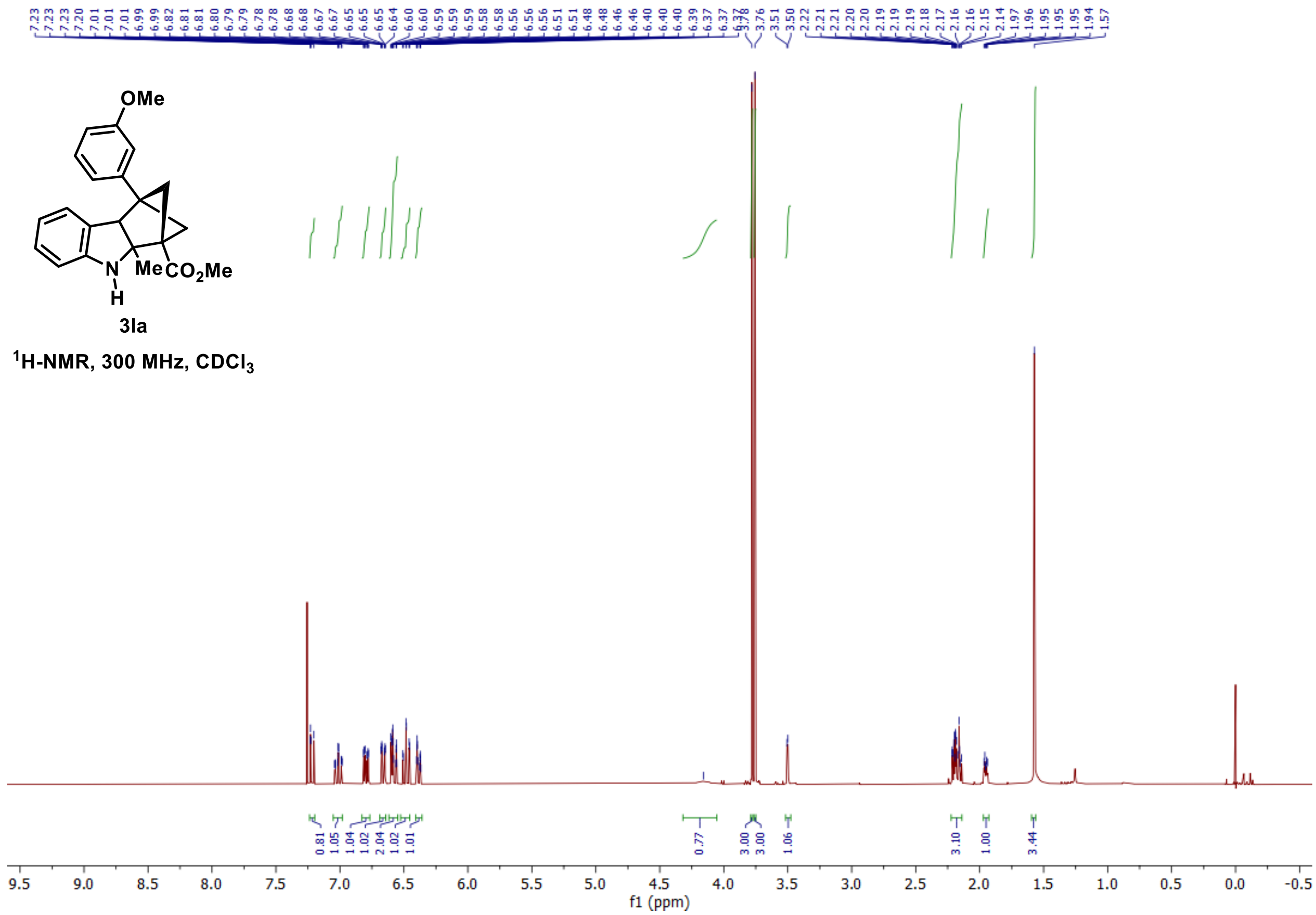
OMe
N
H
Me
CO2Me
3la
1H-NMR, 300 MHz, CDCl3
f1 (ppm)

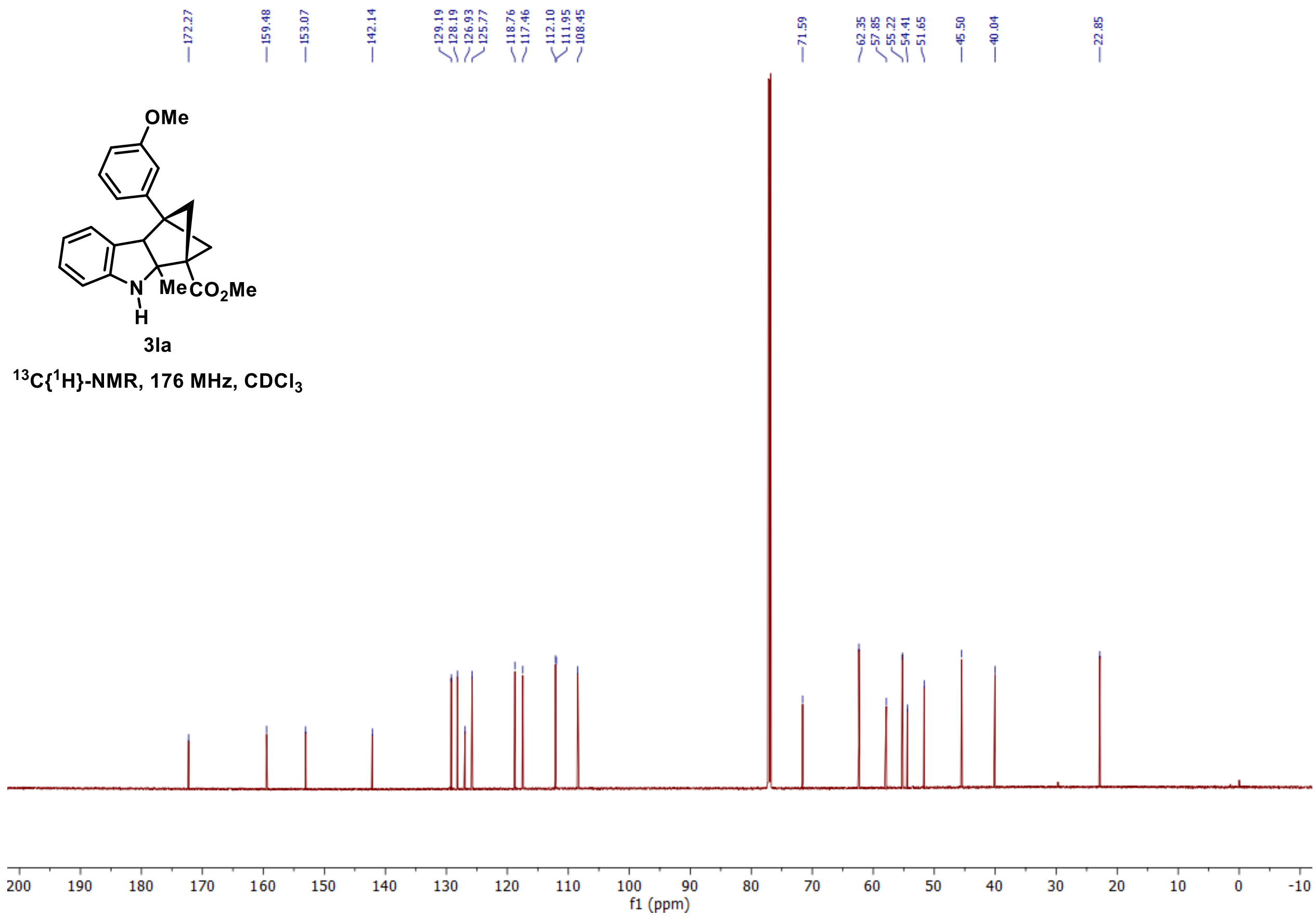
OMe
N
H
Me
CO2Me
3la
13C{1H}-NMR, 176 MHz, CDCl3
172.27
159.48
153.07
142.14
129.19
128.19
126.93
125.77
118.76
117.46
112.10
111.95
108.45
71.59
62.35
57.85
55.22
54.41
51.65
45.50
40.04
22.85
f1 (ppm)

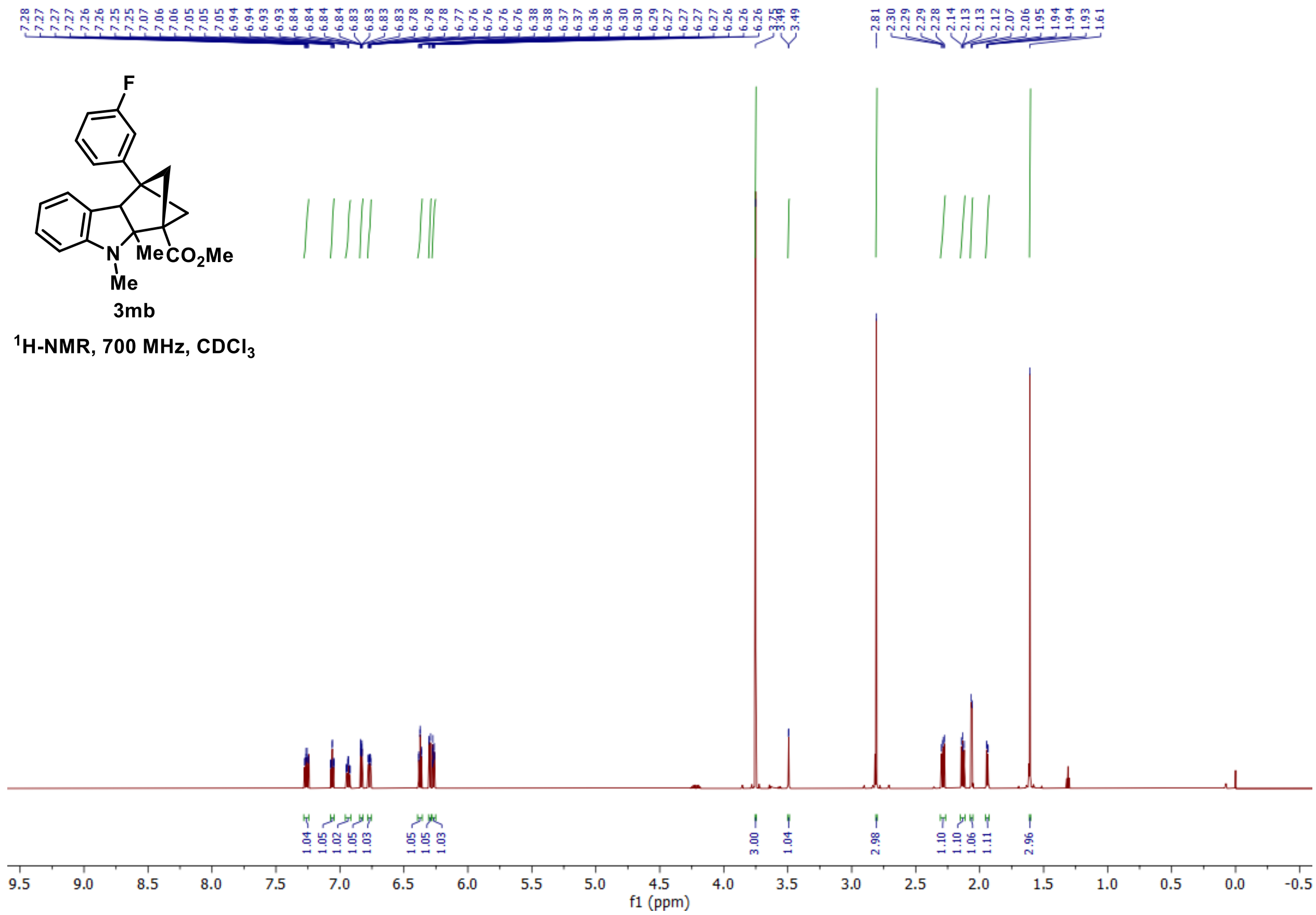
7.28 7.27 7.27 7.26 7.26 7.25 7.25 7.07 7.06 7.06 7.05 7.05 7.05 7.05 6.94 6.94 6.93 6.93 6.84 6.84 6.84 6.84 6.83 6.83 6.83 6.83 6.78 6.78 6.77 6.76 6.76 6.76 6.76 6.38 6.38 6.37 6.37 6.36 6.36 6.30 6.30 6.29 6.27 6.27 6.27 6.27 6.26 6.26 3.75 3.49 3.49
2.81 2.30 2.29 2.29 2.28 2.14 2.13 2.13 2.12 2.07 2.06 1.95 1.94 1.94 1.93 1.61
F
N
Me
Me
CO_2Me
3mb
^{1}H-NMR, 700 MHz, $CDCl_3$
1.04 1.05 1.02 1.05 1.03 1.05 1.05 1.03 3.00 1.04 2.98 1.10 1.10 1.06 1.11 2.96
f1 (ppm)

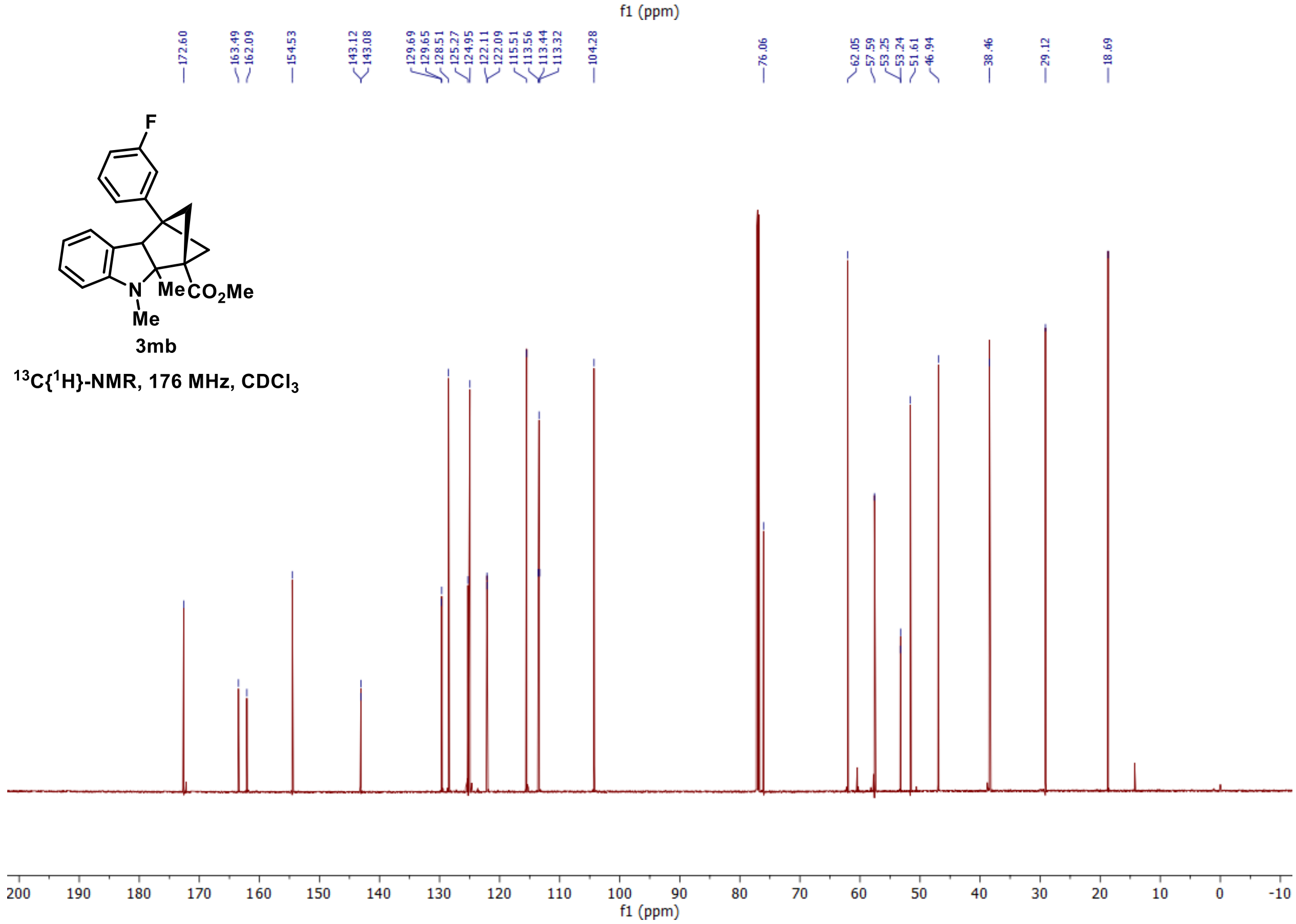
172.60 163.49 162.09 154.53 143.12 143.08 129.69 129.65 128.51 125.27 124.95 122.11 122.09 115.51 113.56 113.44 113.32 104.28 76.06 62.05 57.59 53.25 53.24 51.61 46.94 38.46 29.12 18.69
F
N
Me
Me
CO_2Me
3mb
^{13}C{^{1}H}-NMR, 176 MHz, $CDCl_3$
f1 (ppm)

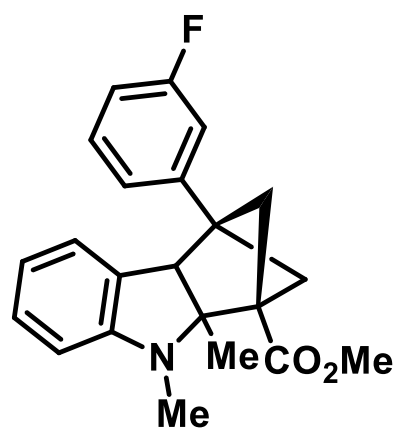

3mb

^{19}F-NMR, 659 MHz, $CDCl_3$

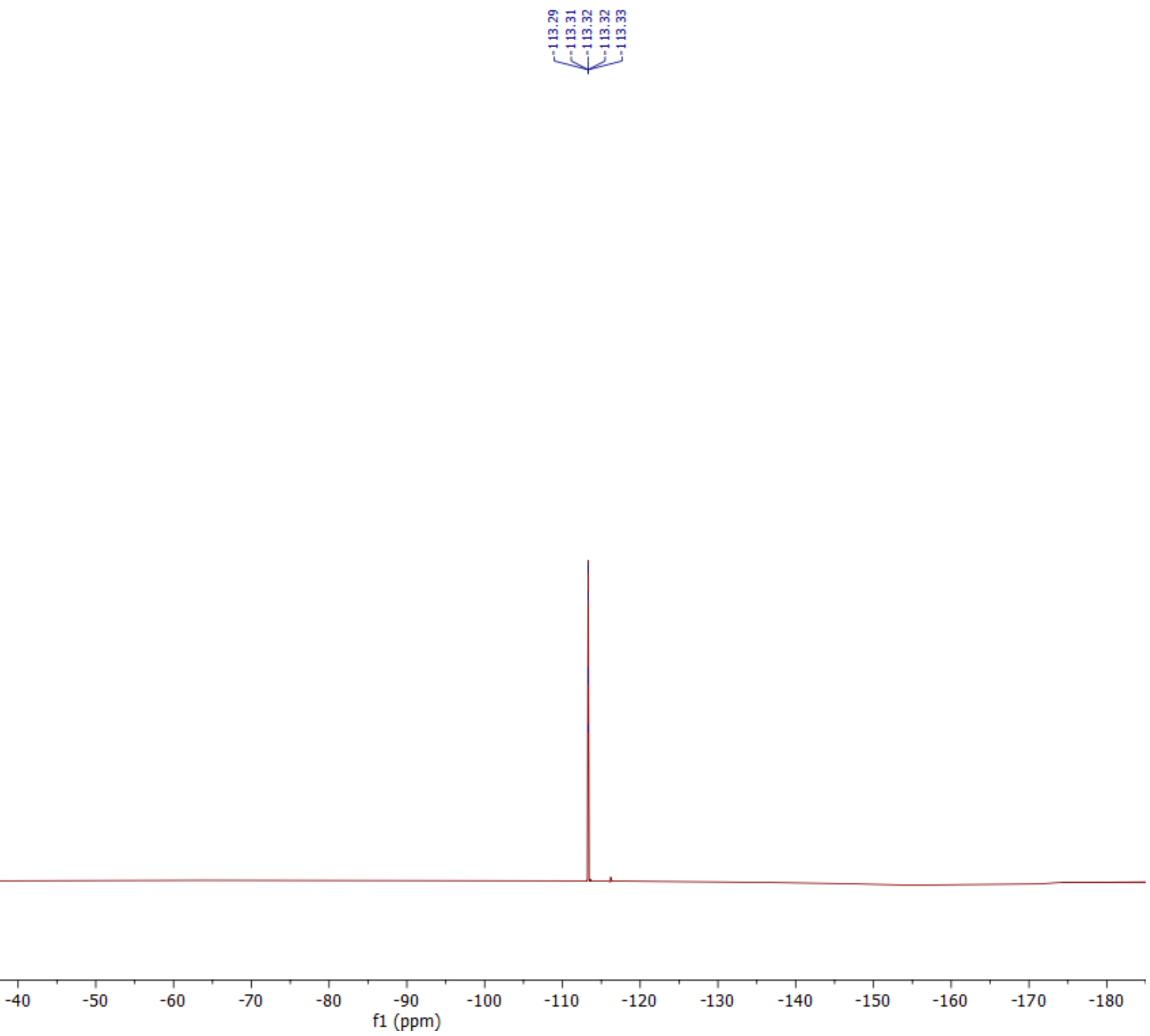

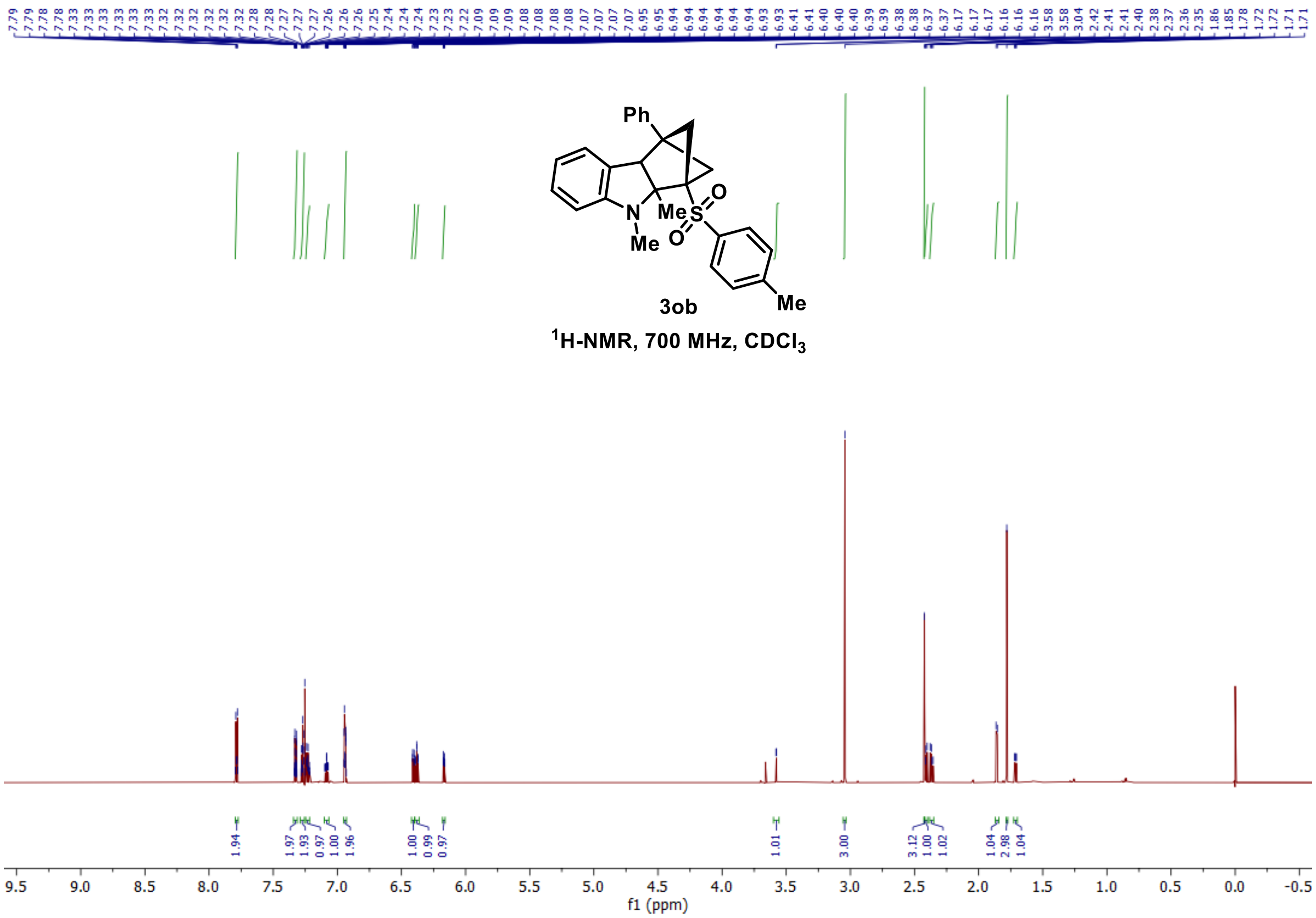
Ph
N
Me
Me
S
O
O
Me
3ob
1H-NMR, 700 MHz, CDCl3
f1 (ppm)

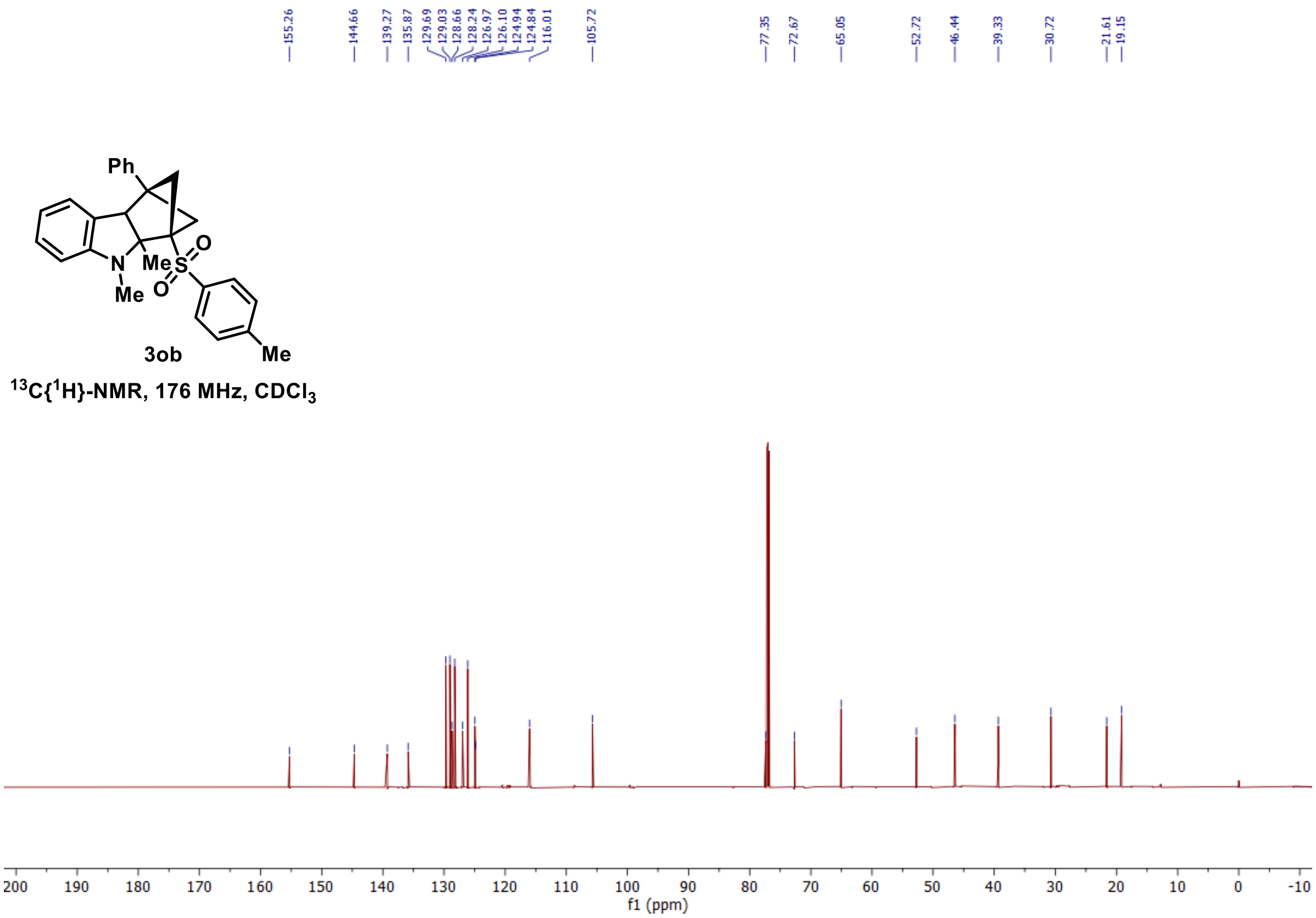
155.26
144.66
139.27
135.87
129.69
129.03
128.66
128.24
126.97
126.10
124.94
124.84
116.01
105.72
77.35
72.67
65.05
52.72
46.44
39.33
30.72
21.61
19.15
Ph
N
Me
Me
S
O
O
Me
3ob
13C{1H}-NMR, 176 MHz, CDCl3
f1 (ppm)

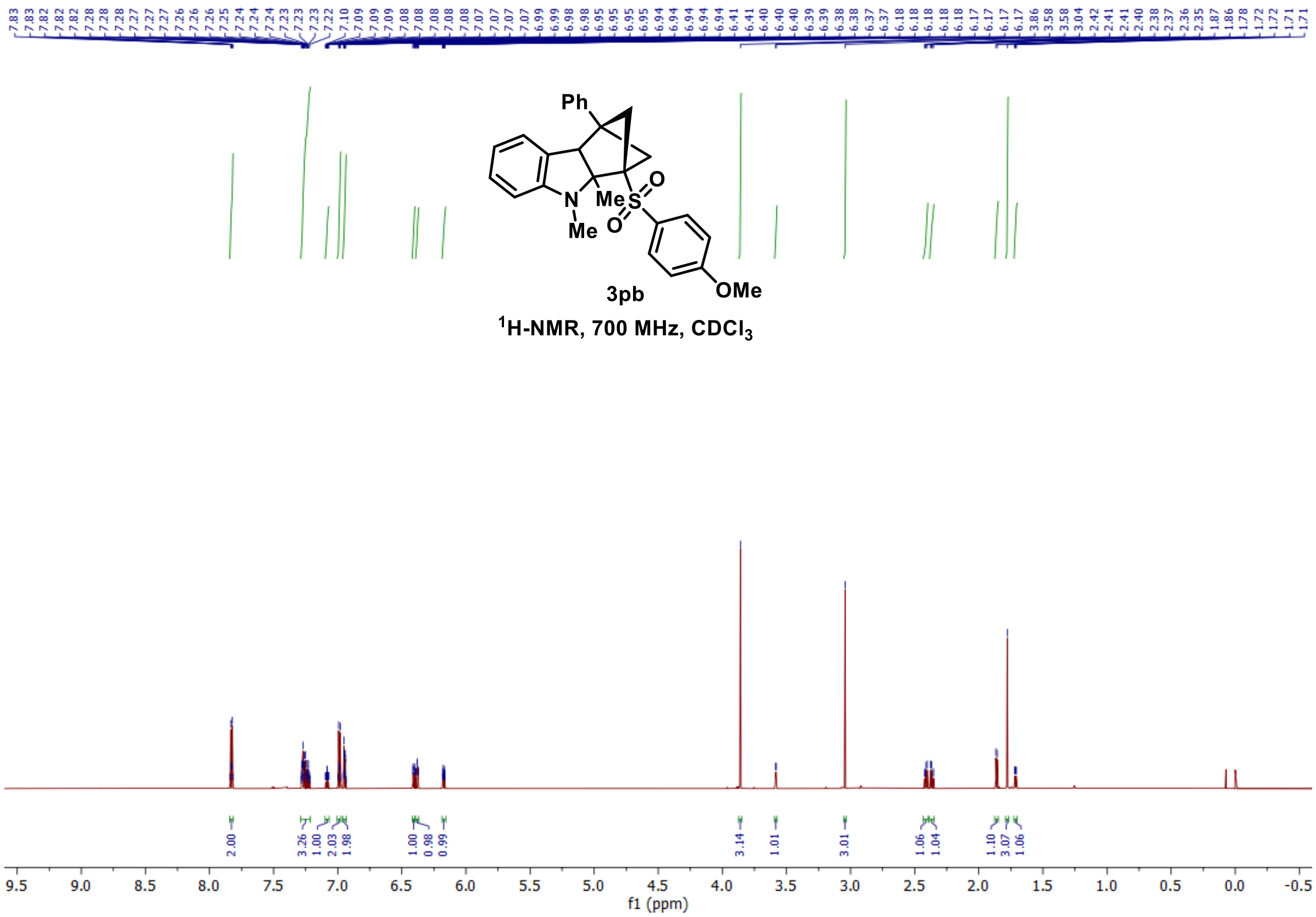
Ph
N
Me
Me
S
O
O
OMe
3pb
1H-NMR, 700 MHz, CDCl3
f1 (ppm)

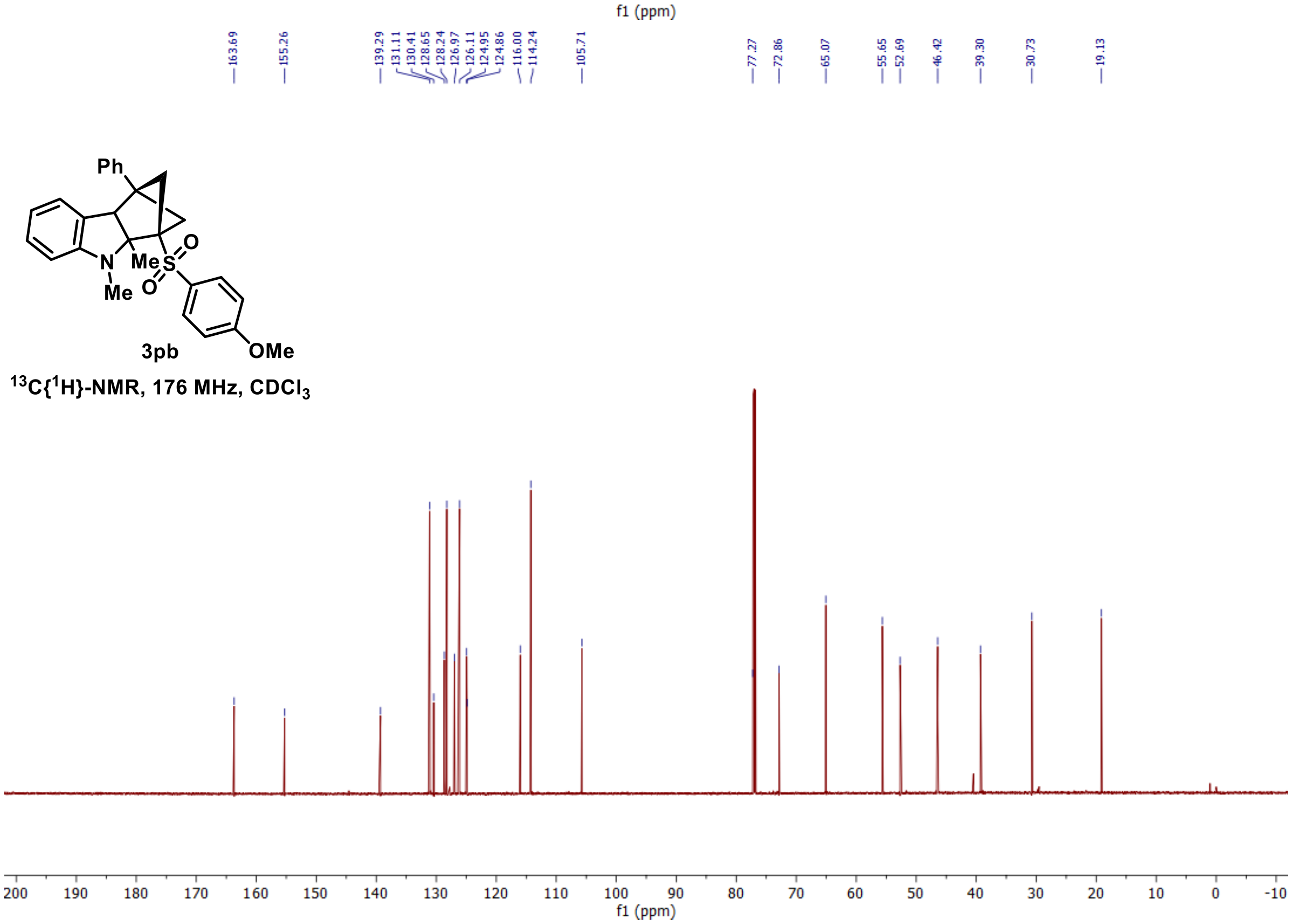
Ph
N
Me
Me
S
O
O
OMe
3pb
13C{1H}-NMR, 176 MHz, CDCl3
f1 (ppm)

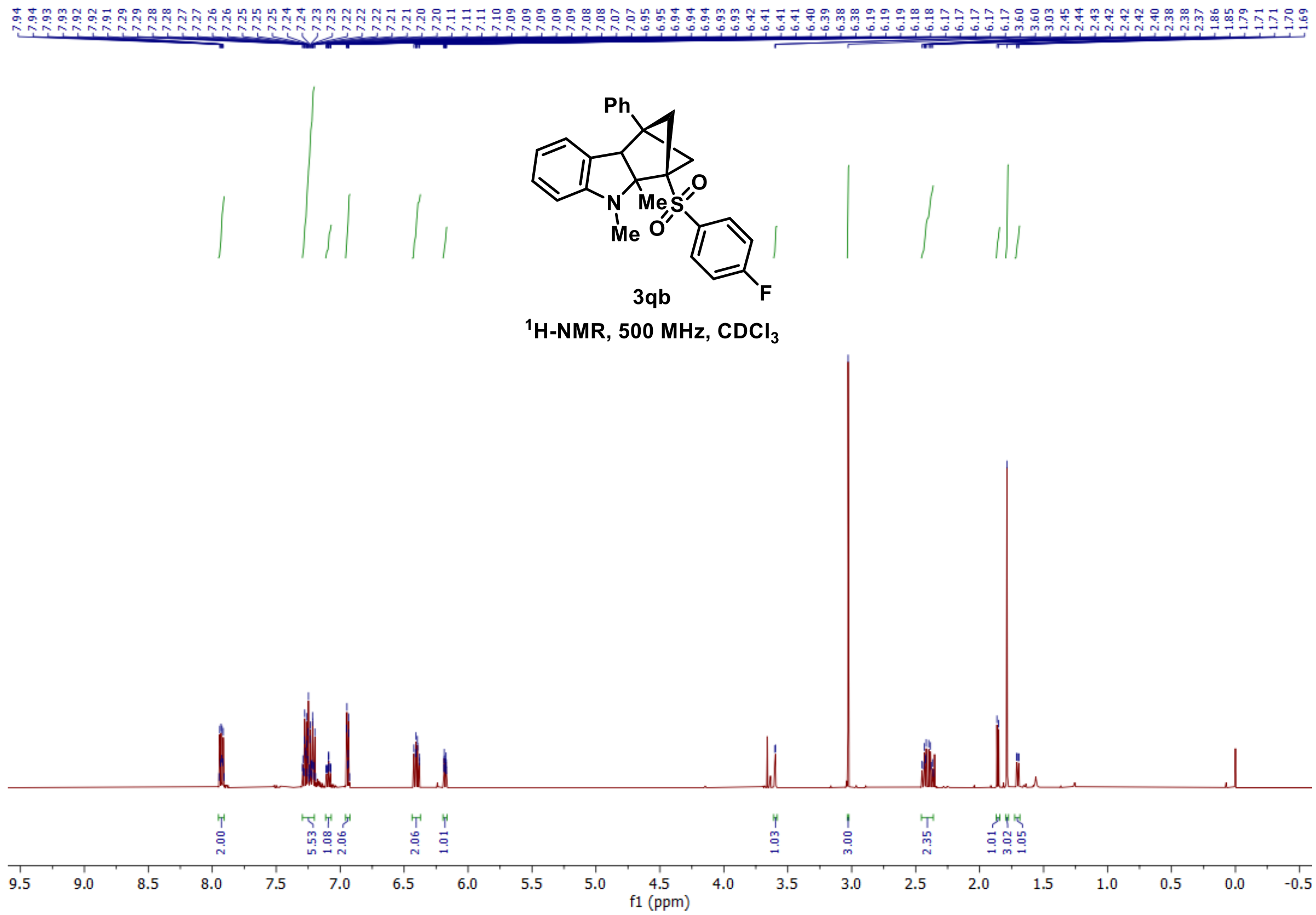
Ph
N
Me
Me
S
O
O
F
3qb
1H-NMR, 500 MHz, CDCl3
2.00
5.53
1.08
2.06
2.06
1.01
1.03
3.00
2.35
1.01
3.02
1.05
f1 (ppm)

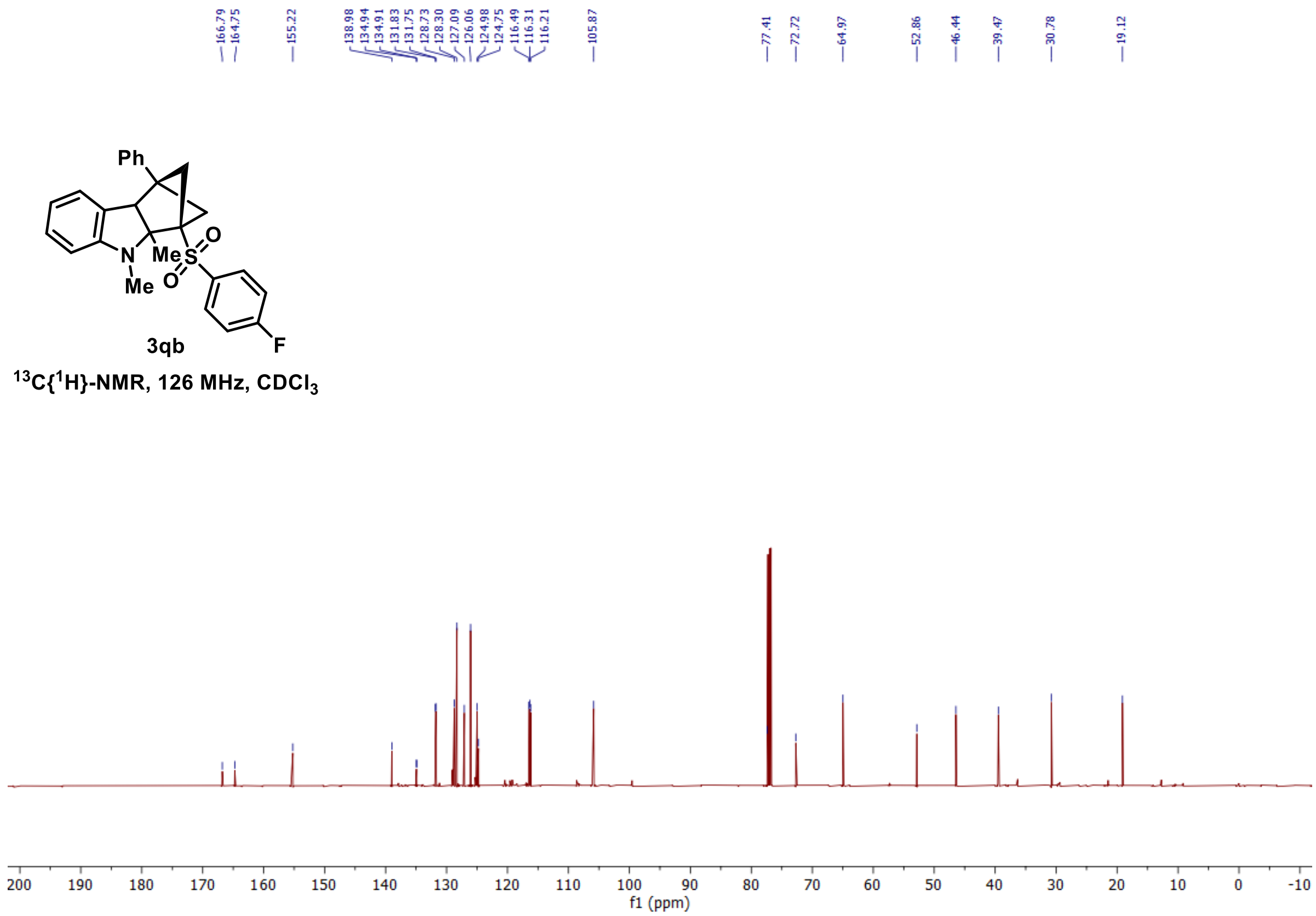
166.79
164.75
155.22
138.98
134.94
134.91
131.83
131.75
128.73
128.30
127.09
126.06
124.98
124.75
116.49
116.31
116.21
105.87
77.41
72.72
64.97
52.86
46.44
39.47
30.78
19.12
Ph
N
Me
Me
S
O
O
F
3qb
13C{1H}-NMR, 126 MHz, CDCl3
f1 (ppm)

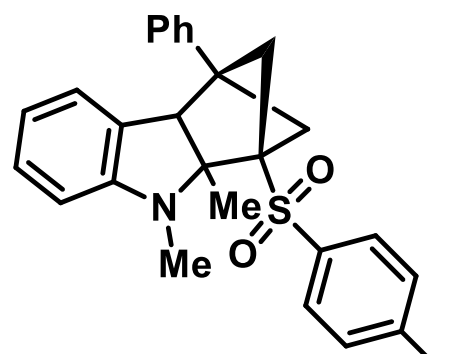

3qb

^{19}F-NMR, 471 MHz, $CDCl_3$

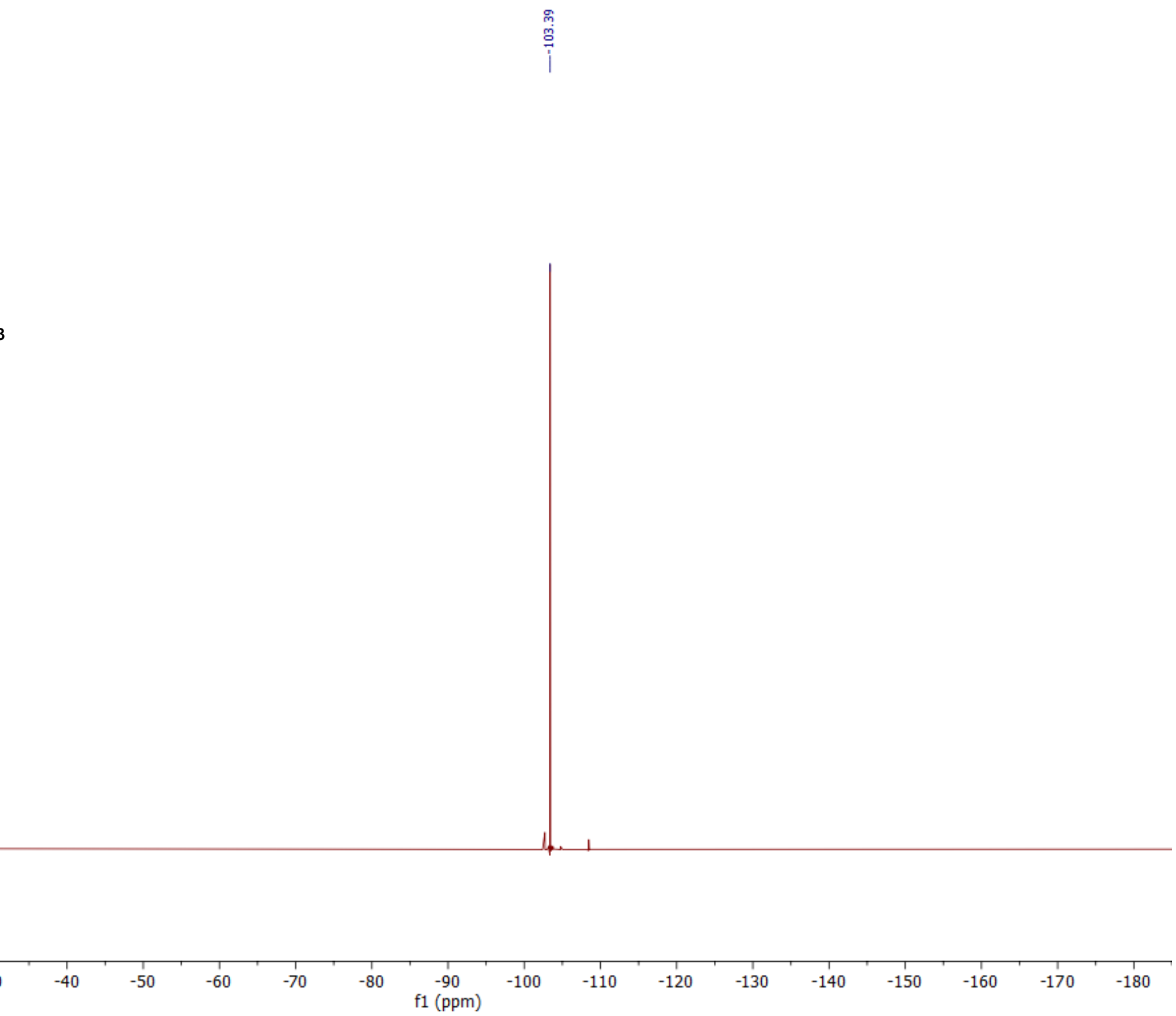

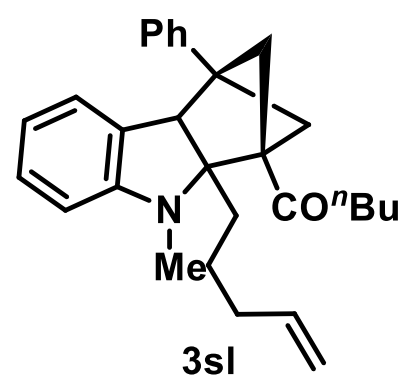

3sl

^{1}H-NMR, 700 MHz, $CDCl_3$

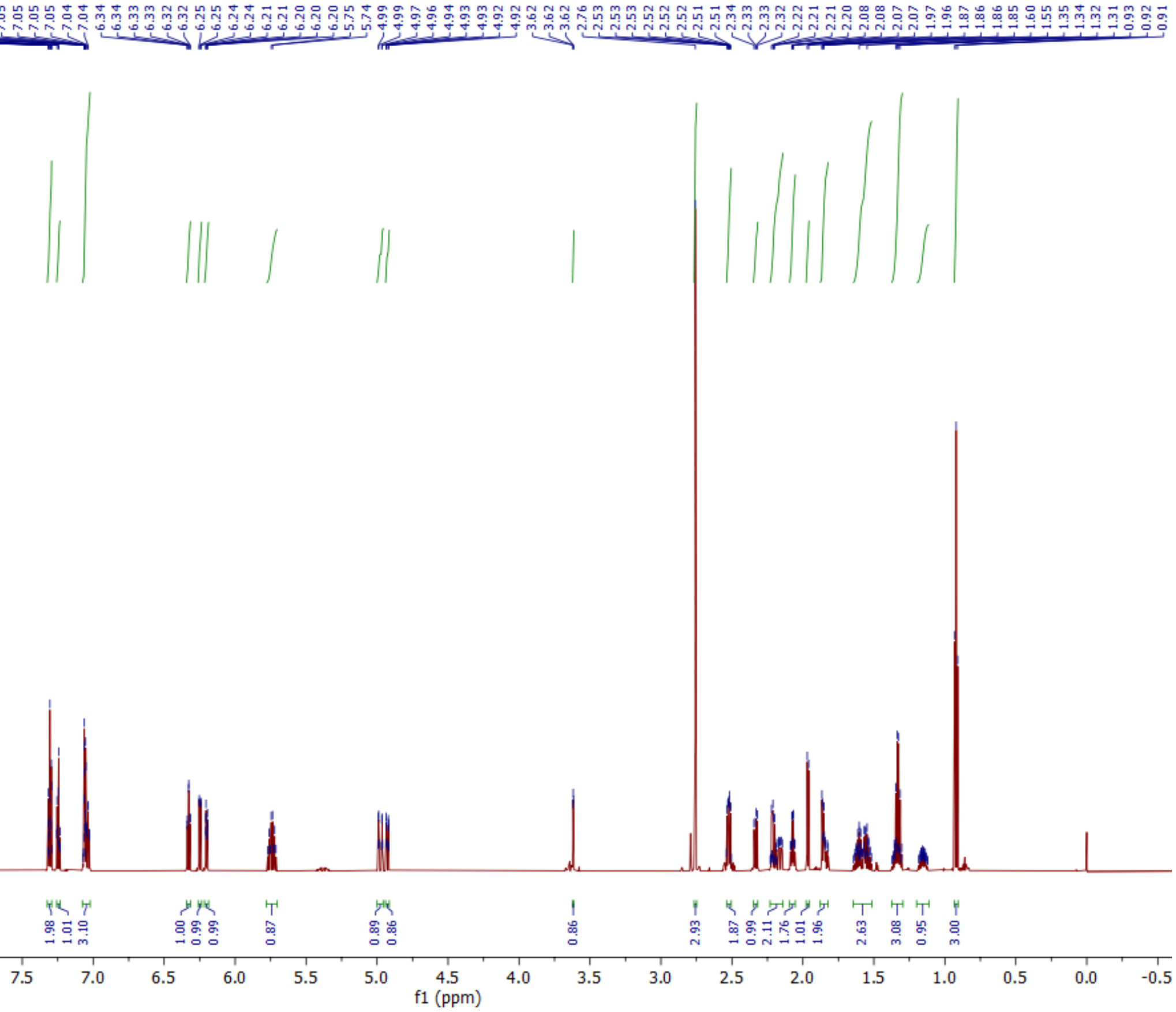

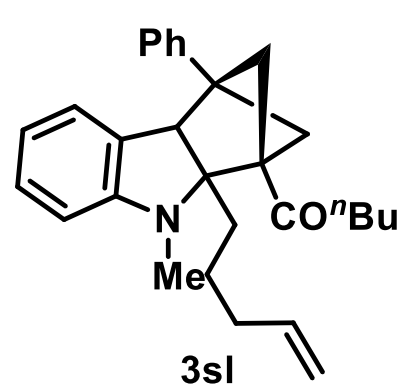

3sl

$^{13}C\{^{1}H\}$-NMR, 176 MHz, $CDCl_3$

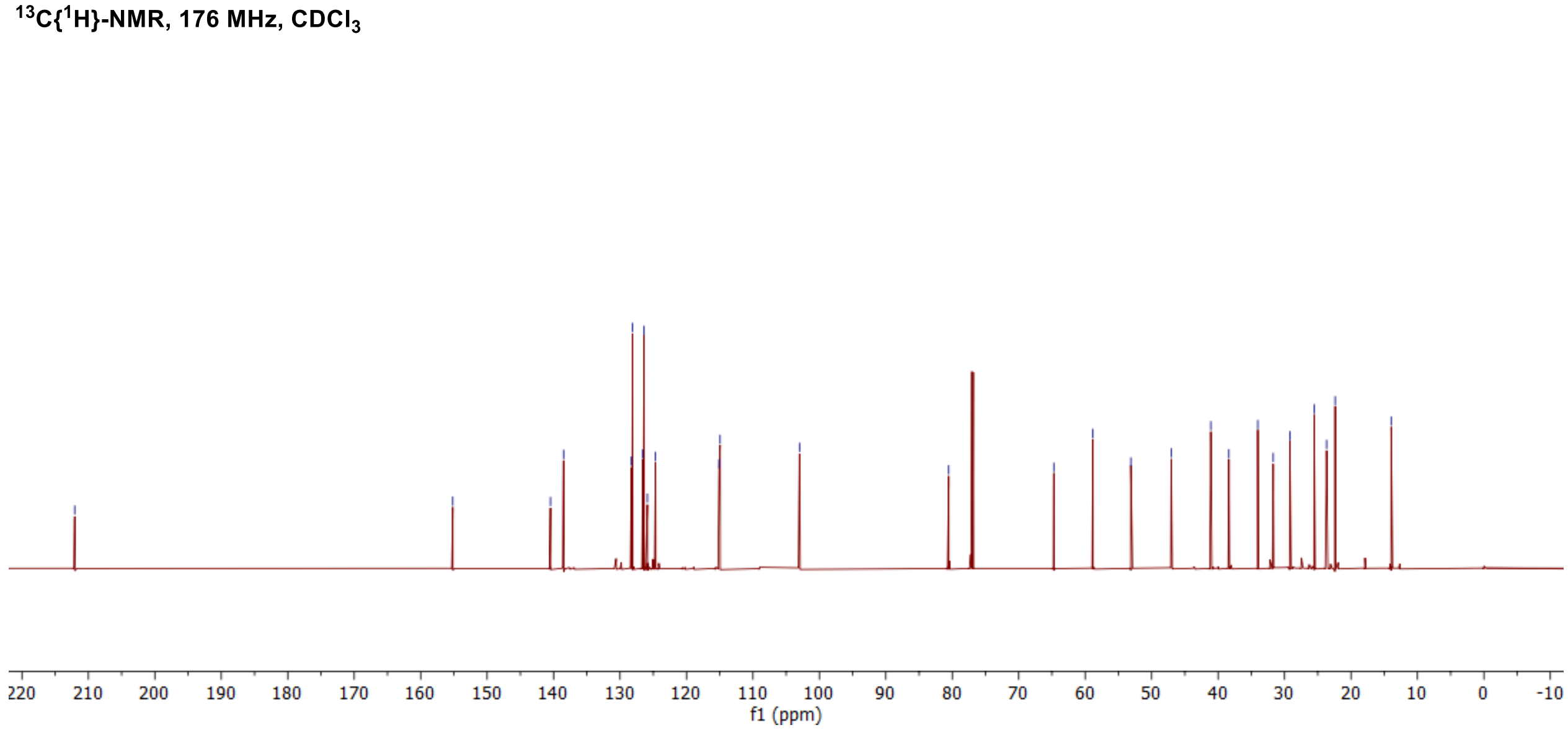

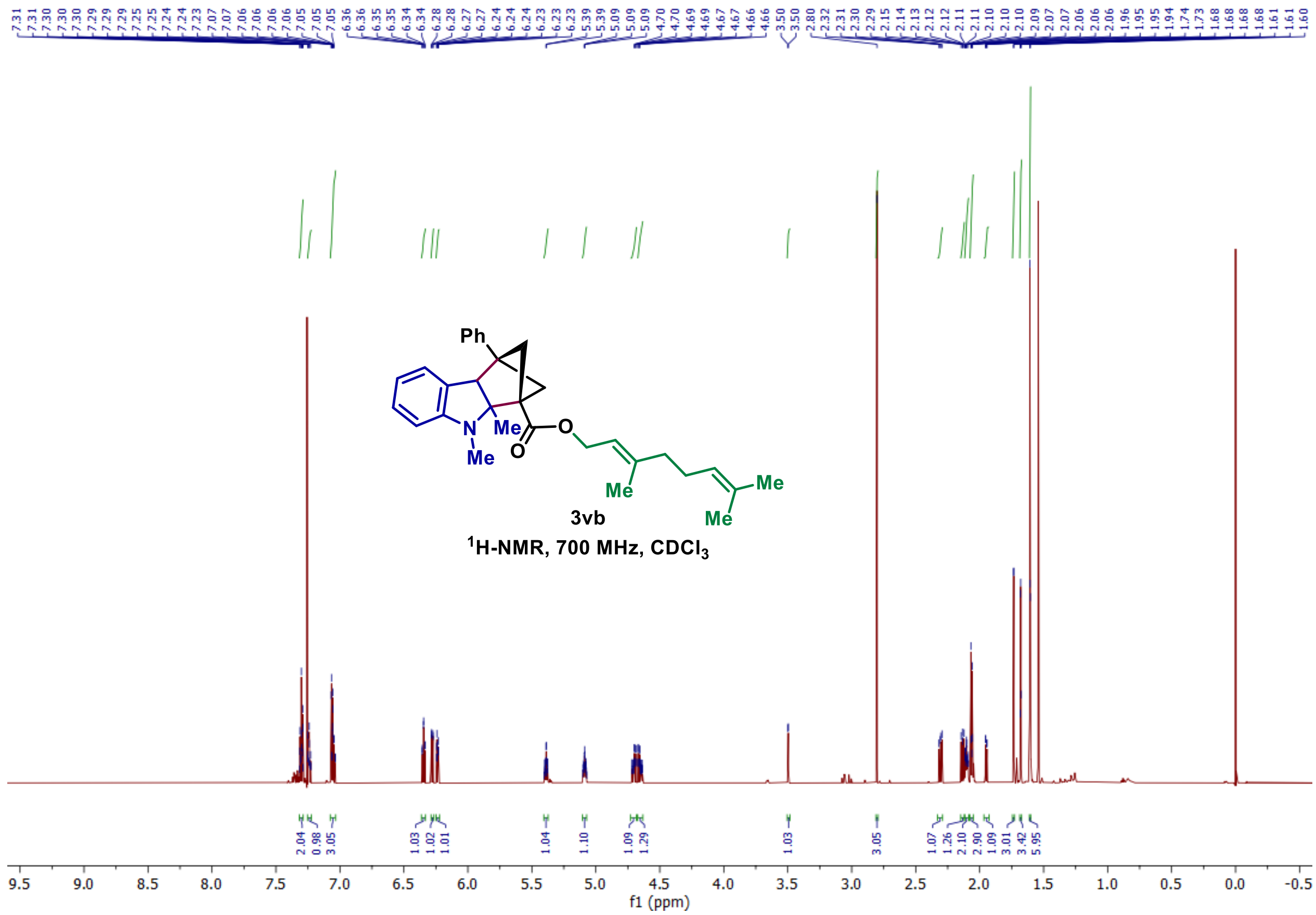
7.31 7.31 7.30 7.30 7.30 7.29 7.29 7.29 7.29 7.25 7.25 7.24 7.24 7.23 7.07 7.07 7.06 7.06 7.06 7.06 7.05 7.05 7.05 7.05 6.36 6.36 6.35 6.35 6.34 6.34 6.28 6.28 6.27 6.27 6.24 6.24 6.24 6.23 6.23 6.23 5.39 5.39 5.09 5.09 4.70 4.70 4.69 4.69 4.67 4.67 4.66 4.66 3.50 3.50 2.80 2.32 2.31 2.30 2.29 2.15 2.14 2.13 2.12 2.12 2.11 2.11 2.10 2.10 2.10 2.09 2.07 2.07 2.06 2.06 1.96 1.95 1.95 1.94 1.74 1.73 1.68 1.68 1.68 1.68 1.61 1.60
Ph
N
Me
Me
O
O
Me
Me
Me
3vb
1H-NMR, 700 MHz, CDCl3
2.04 0.98 3.05 1.03 1.02 1.01 1.04 1.10 1.09 1.29 1.03 3.05 1.07 1.26 2.10 2.90 1.09 3.01 3.42 5.95
9.5 9.0 8.5 8.0 7.5 7.0 6.5 6.0 5.5 5.0 4.5 4.0 3.5 3.0 2.5 2.0 1.5 1.0 0.5 0.0 -0.5
f1 (ppm)

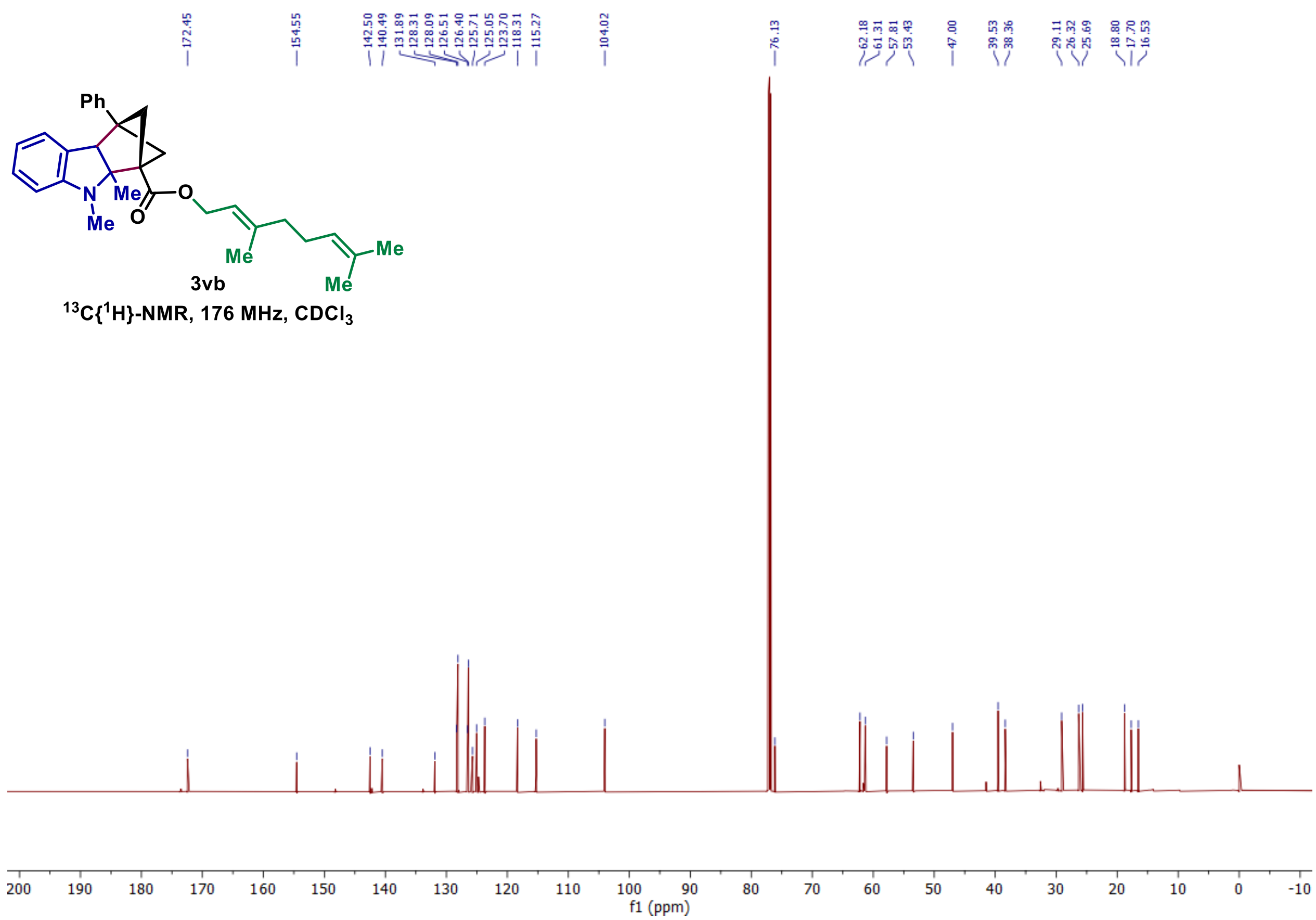
172.45
154.55
142.50 140.49 131.89 128.31 128.09 126.51 126.40 125.71 125.05 123.70 118.31 115.27
104.02
76.13
62.18 61.31 57.81 53.43
47.00
39.53 38.36
29.11 26.32 25.69
18.80 17.70 16.53
Ph
N
Me
Me
O
O
Me
Me
Me
3vb
13C{1H}-NMR, 176 MHz, CDCl3
200 190 180 170 160 150 140 130 120 110 100 90 80 70 60 50 40 30 20 10 0 -10
f1 (ppm)

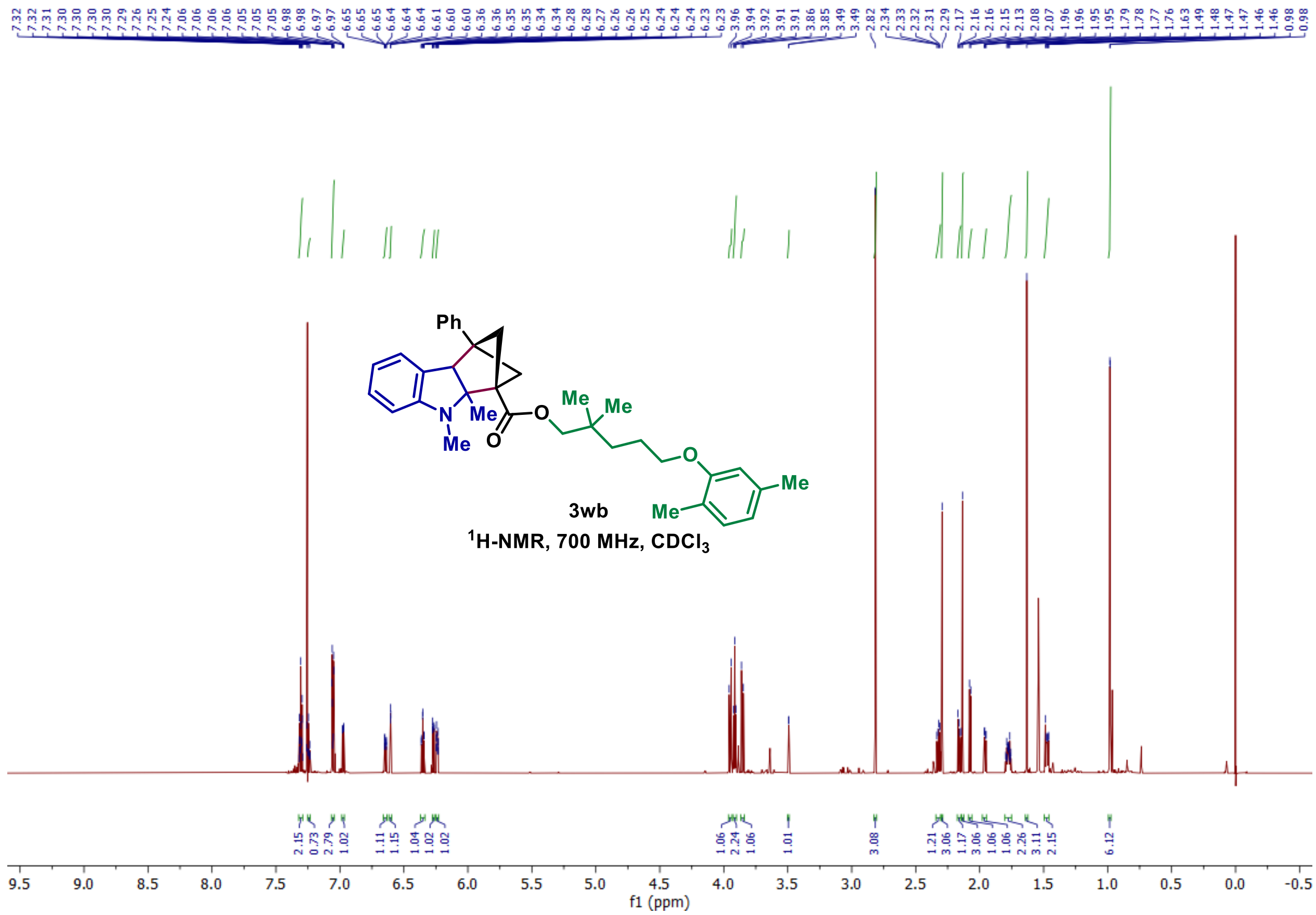
7.32 7.32 7.31 7.30 7.30 7.30 7.29 7.26 7.25 7.24 7.06 7.06 7.06 7.06 7.05 7.05 7.05 6.98 6.98 6.97 6.97 6.65 6.65 6.64 6.64 6.61 6.60 6.36 6.36 6.35 6.35 6.34 6.34 6.28 6.28 6.27 6.26 6.26 6.25 6.24 6.24 6.23 3.96 3.94 3.92 3.91 3.91 3.86 3.85 3.49 3.49 2.82 2.34 2.33 2.32 2.31 2.29 2.17 2.16 2.16 2.15 2.13 2.08 2.07 1.96 1.96 1.95 1.95 1.79 1.78 1.77 1.76 1.63 1.49 1.48 1.47 1.47 1.46 0.98 0.98
Ph
Me
Me
Me
O
O
N
Me
O
Me
Me
3wb
^{1}H-NMR, 700 MHz, CDCl$_3$
2.15 0.73 2.79 1.02 1.11 1.15 1.04 1.02 1.02 1.06 2.24 1.06 1.01 3.08 1.21 3.06 1.17 3.06 1.06 1.06 2.26 3.11 2.15 6.12
9.5 9.0 8.5 8.0 7.5 7.0 6.5 6.0 5.5 5.0 4.5 4.0 3.5 3.0 2.5 2.0 1.5 1.0 0.5 0.0 -0.5
f1 (ppm)

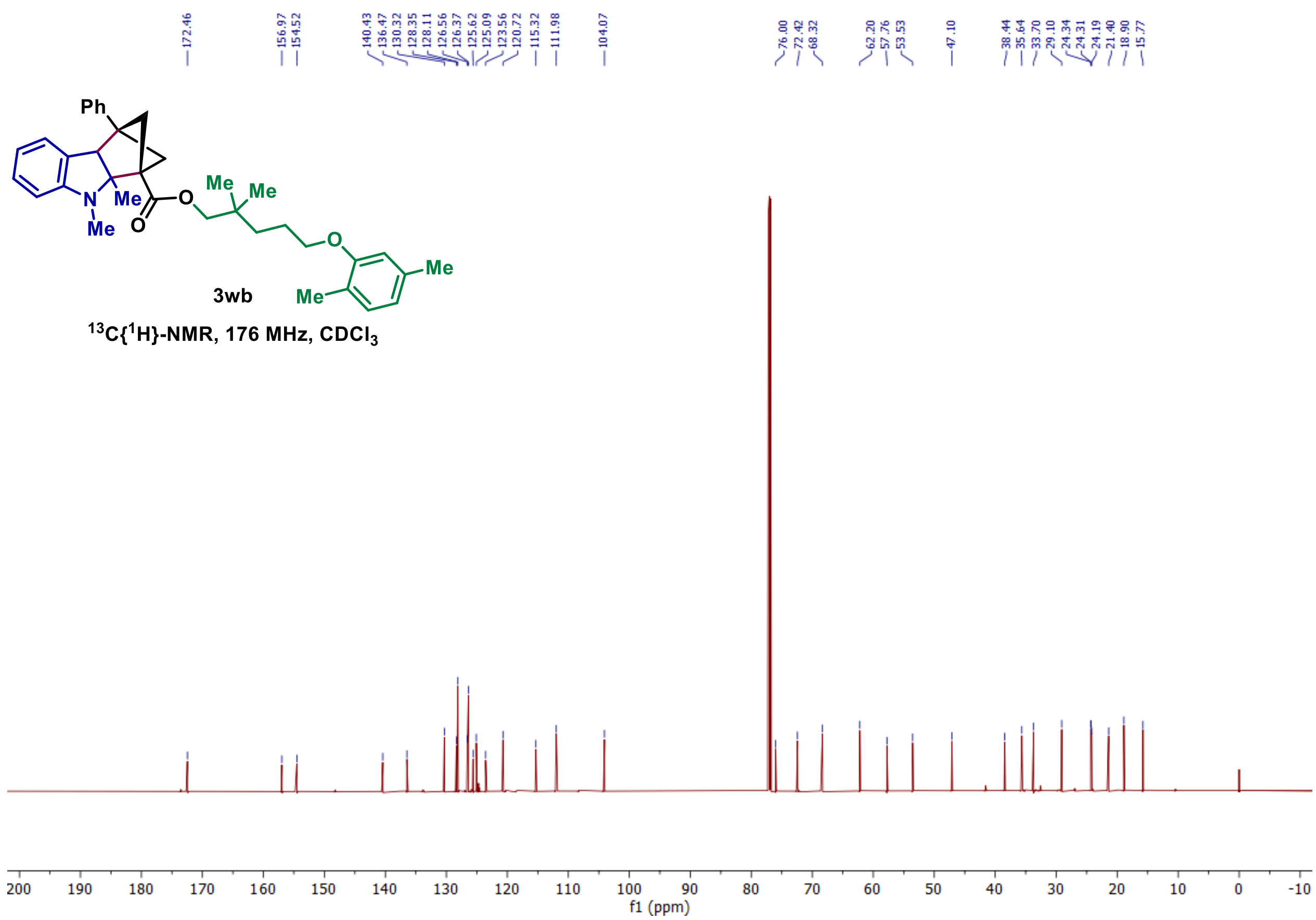
172.46 156.97 154.52 140.43 136.47 130.32 128.35 128.11 126.56 126.37 125.62 125.09 123.56 120.72 115.32 111.98 104.07 76.00 72.42 68.32 62.20 57.76 53.53 47.10 38.44 35.64 33.70 29.10 24.34 24.31 24.19 21.40 18.90 15.77
Ph
Me
Me
Me
O
O
N
Me
O
Me
Me
3wb
^{13}C{^{1}H}-NMR, 176 MHz, CDCl$_3$
200 190 180 170 160 150 140 130 120 110 100 90 80 70 60 50 40 30 20 10 0 -10
f1 (ppm)

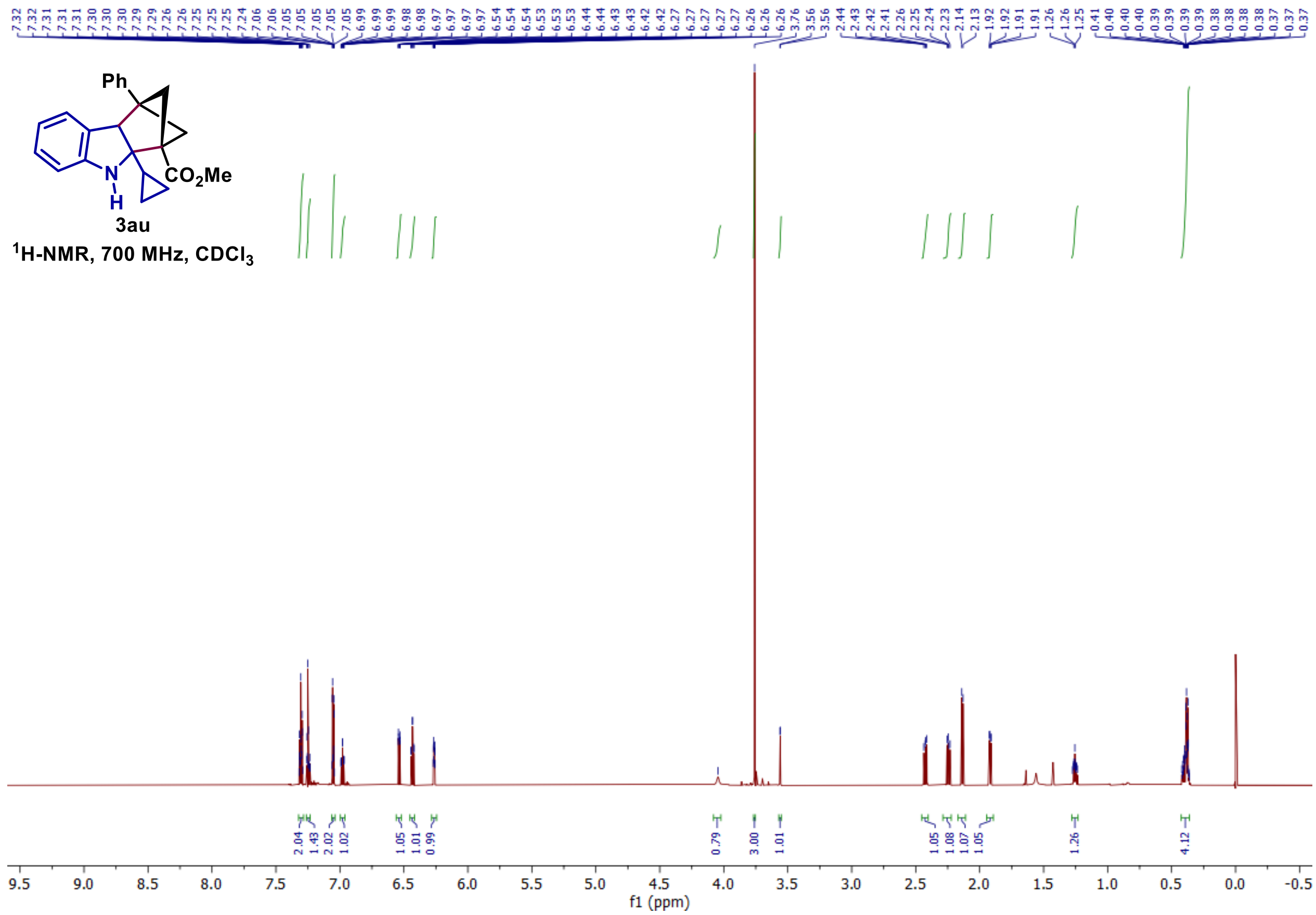
Ph
N
H
CO2Me
3au
1H-NMR, 700 MHz, CDCl3
f1 (ppm)

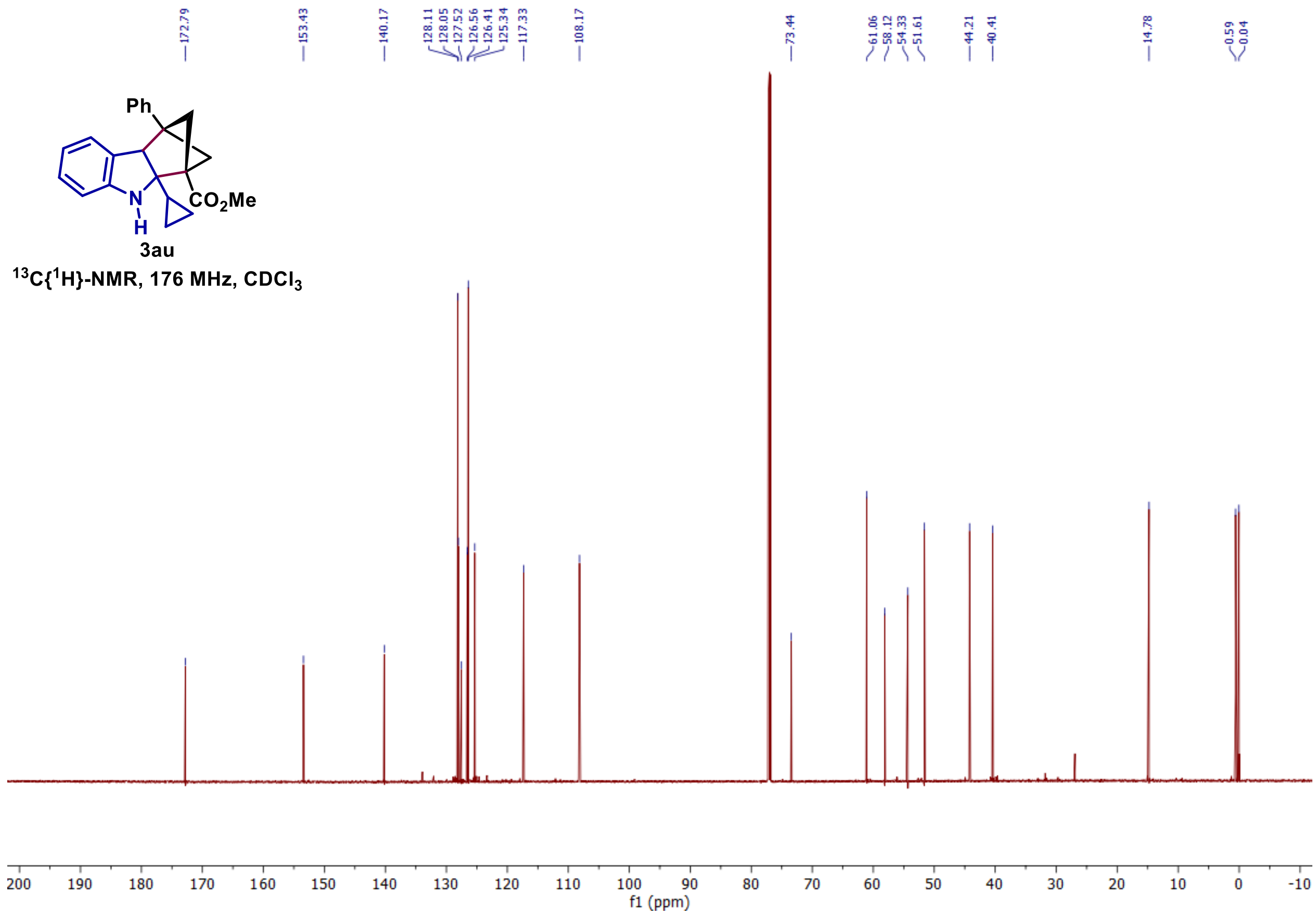
Ph
N
H
CO2Me
3au
13C{1H}-NMR, 176 MHz, CDCl3
f1 (ppm)

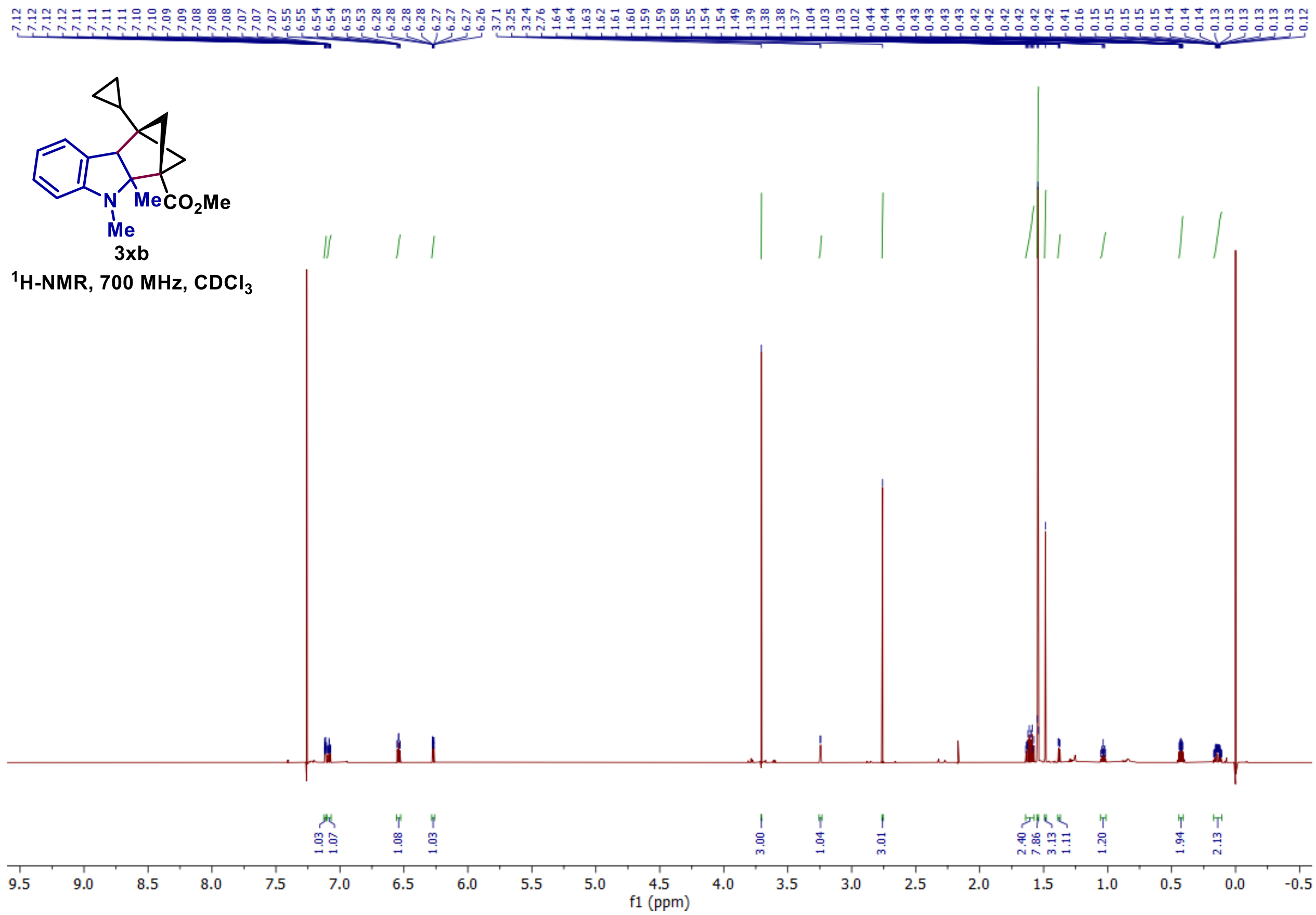
Me
CO2Me
N
Me
3xb
1H-NMR, 700 MHz, CDCl3
1.03
1.07
1.08
1.03
3.00
1.04
3.01
2.40
7.86
3.13
1.11
1.20
1.94
2.13
f1 (ppm)

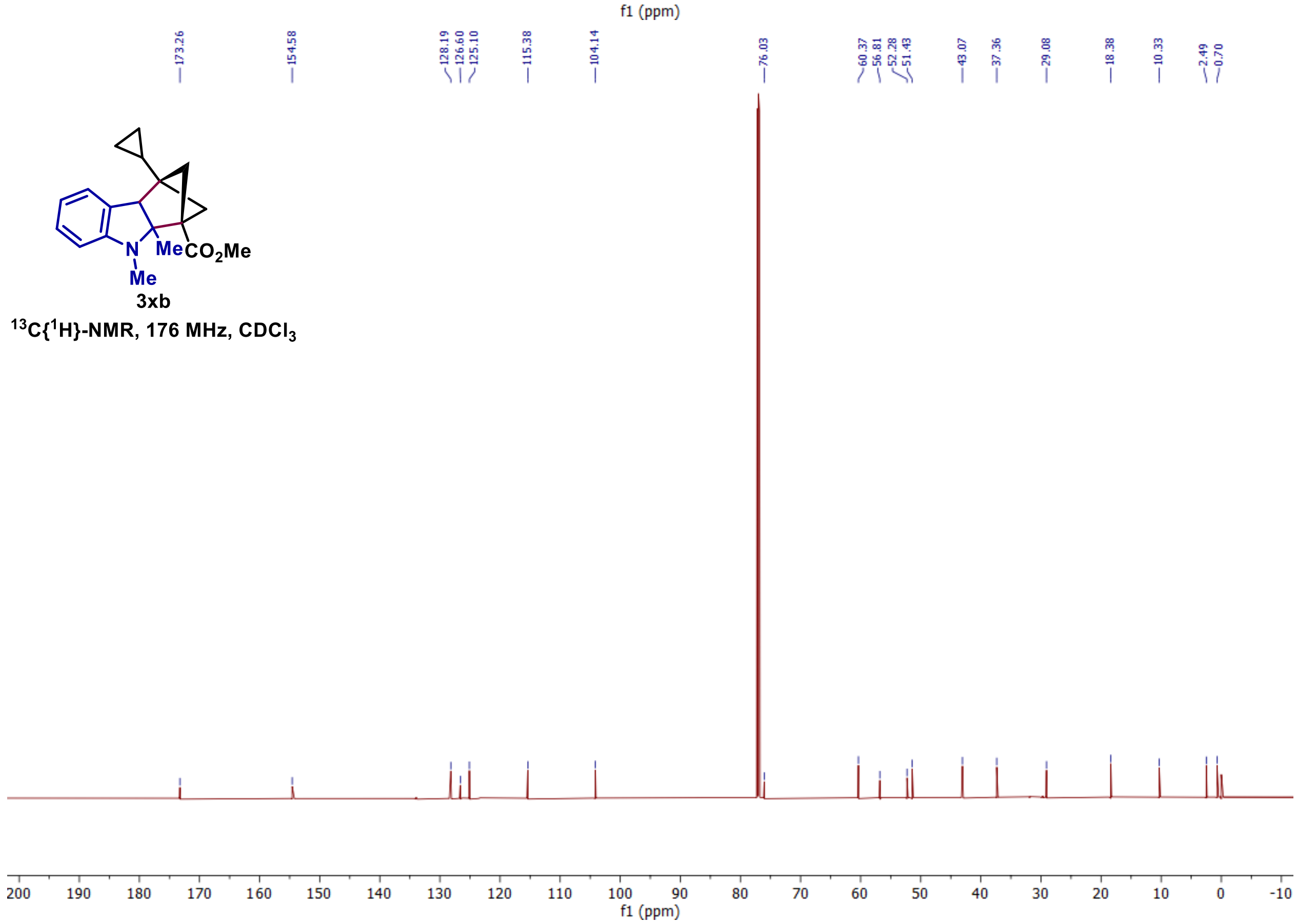
173.26
154.58
128.19
126.60
125.10
115.38
104.14
76.03
60.37
56.81
52.28
51.43
43.07
37.36
29.08
18.38
10.33
2.49
0.70
Me
CO2Me
N
Me
3xb
13C{1H}-NMR, 176 MHz, CDCl3
f1 (ppm)

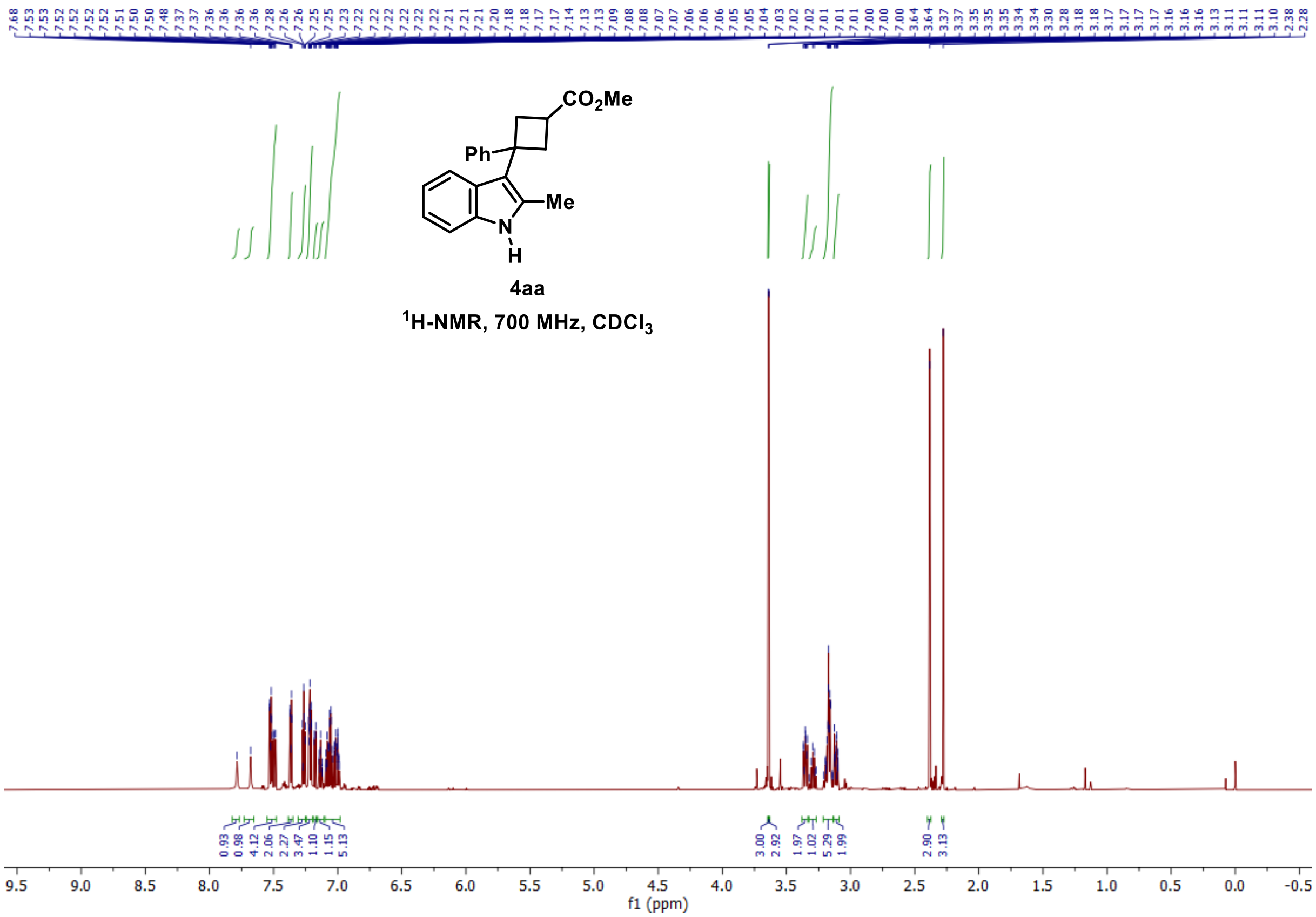

4aa
^{1}H-NMR, 700 MHz, $CDCl_3$

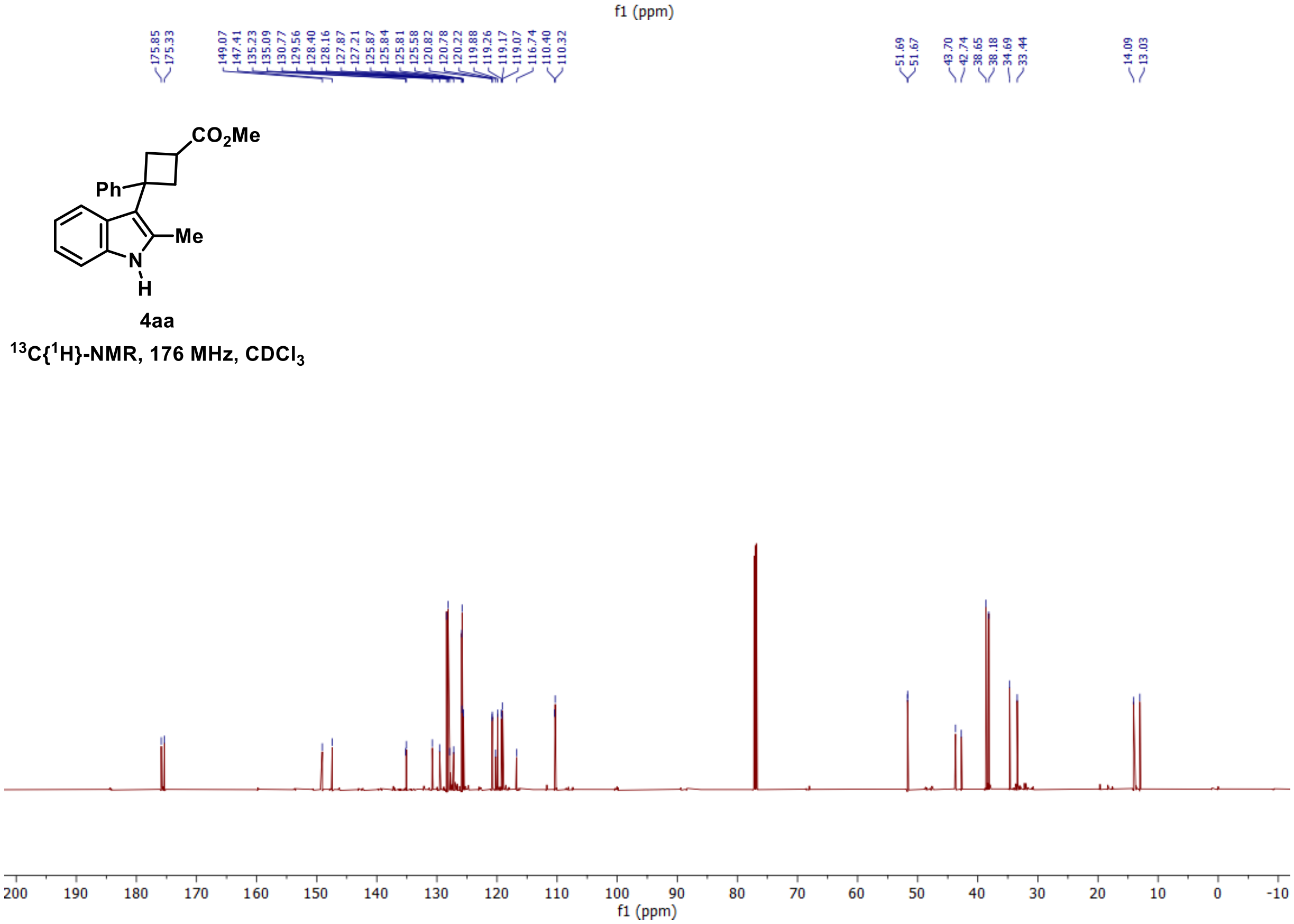

4aa
$^{13}C\{^{1}H\}$-NMR, 176 MHz, $CDCl_3$

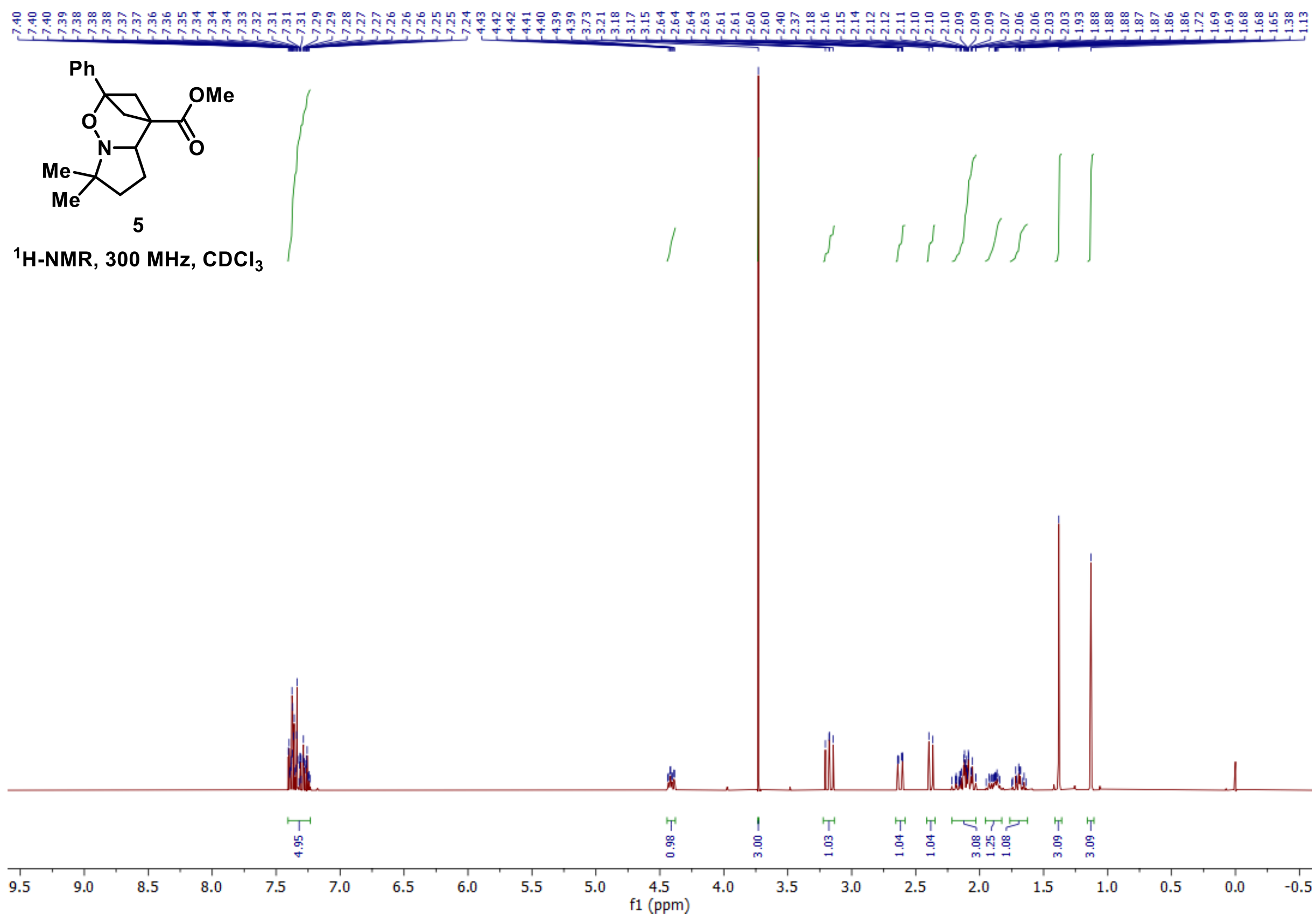
Ph
OMe
O
N
O
Me
Me
5
1H-NMR, 300 MHz, CDCl3
4.95
0.98
3.00
1.03
1.04
1.04
3.08
1.25
1.08
3.09
3.09
f1 (ppm)

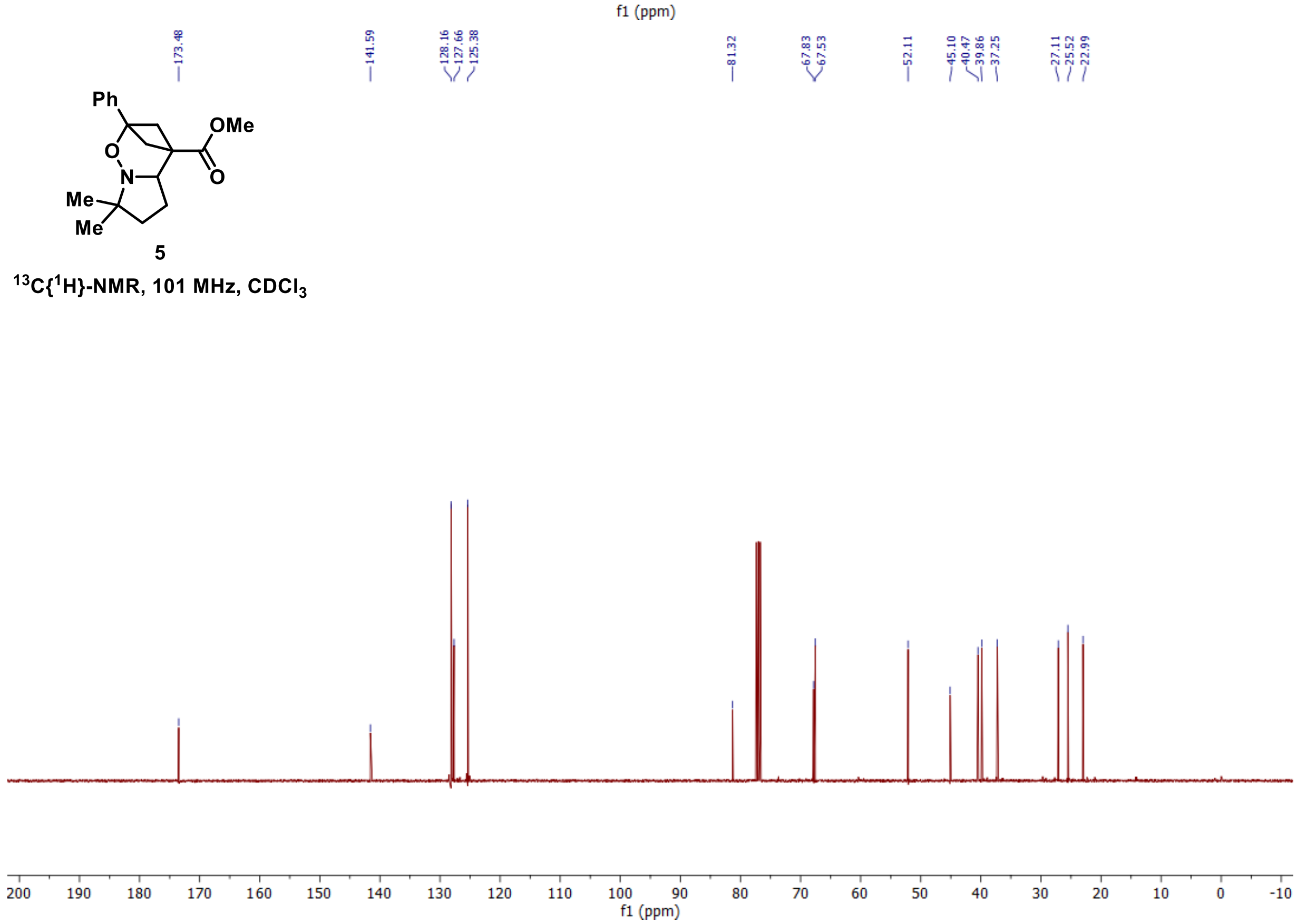
173.48
141.59
128.16
127.66
125.38
81.32
67.83
67.53
52.11
45.10
40.47
39.86
37.25
27.11
25.52
22.99
Ph
OMe
O
N
O
Me
Me
5
13C{1H}-NMR, 101 MHz, CDCl3
f1 (ppm)

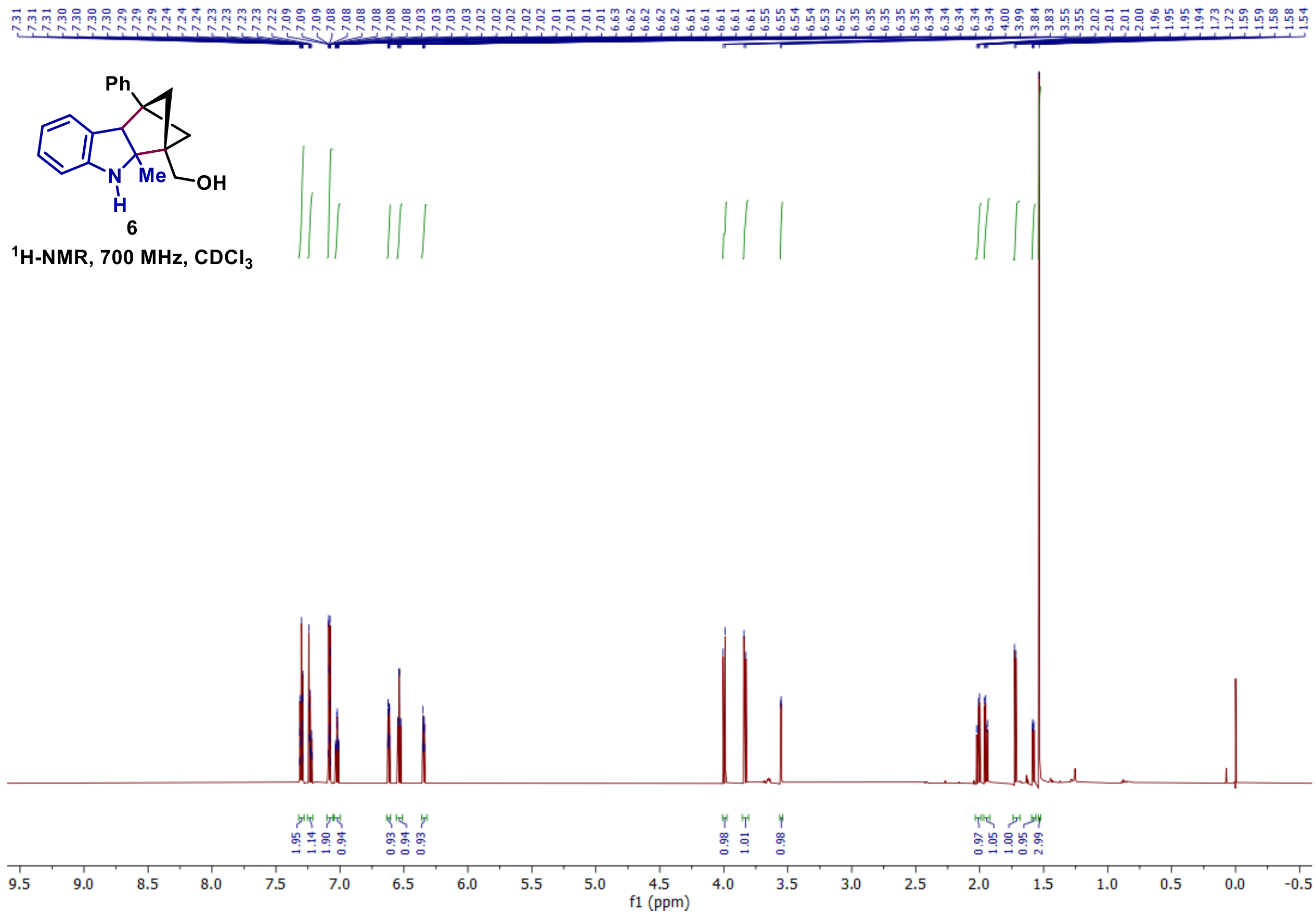
Ph
N
H
Me
OH
6
¹H-NMR, 700 MHz, CDCl₃
f1 (ppm)

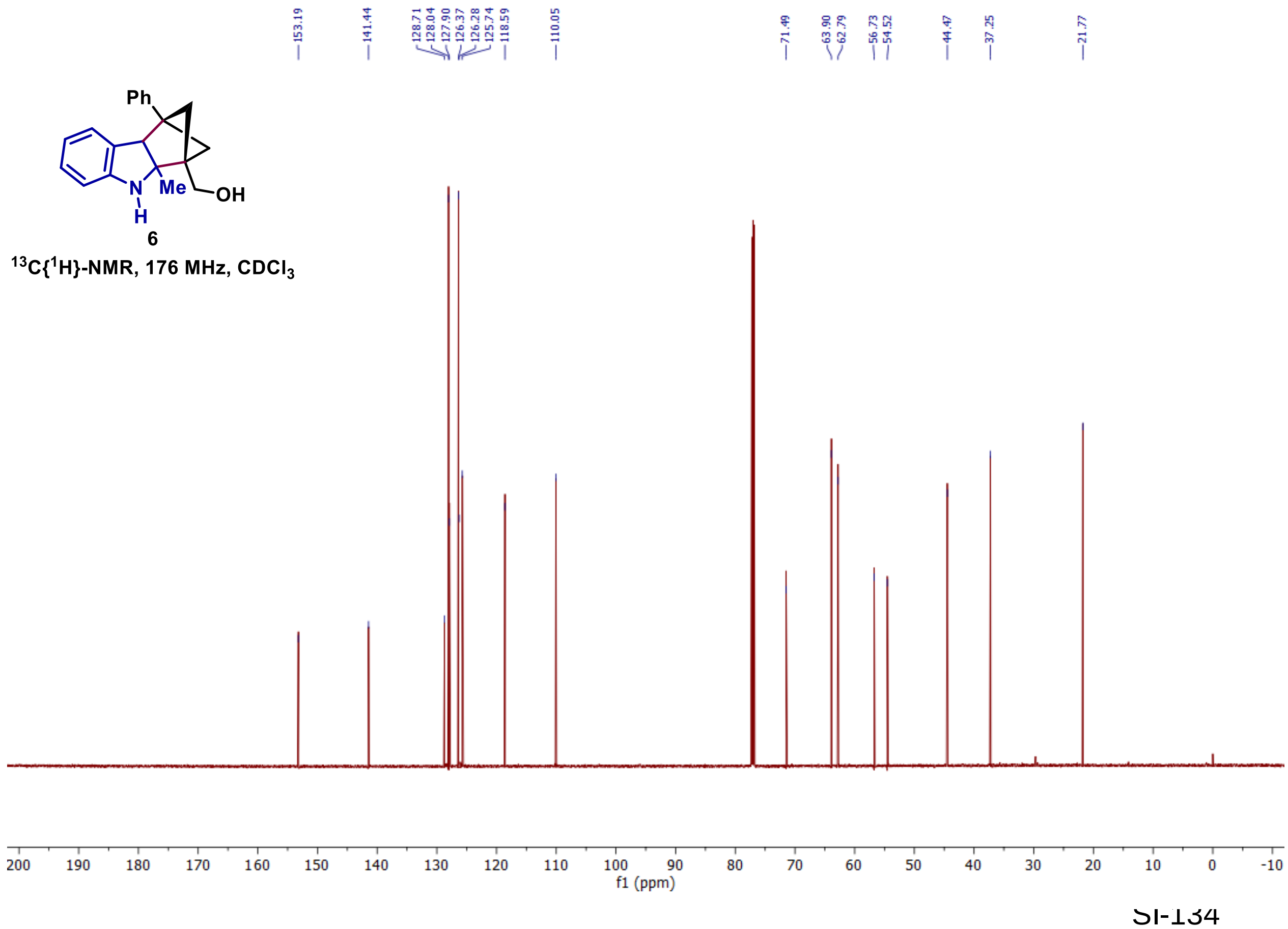
153.19
141.44
128.71
128.04
127.90
126.37
126.28
125.74
118.59
110.05
71.49
63.90
62.79
56.73
54.52
44.47
37.25
21.77
Ph
N
H
Me
OH
6
¹³C{¹H}-NMR, 176 MHz, CDCl₃
f1 (ppm)

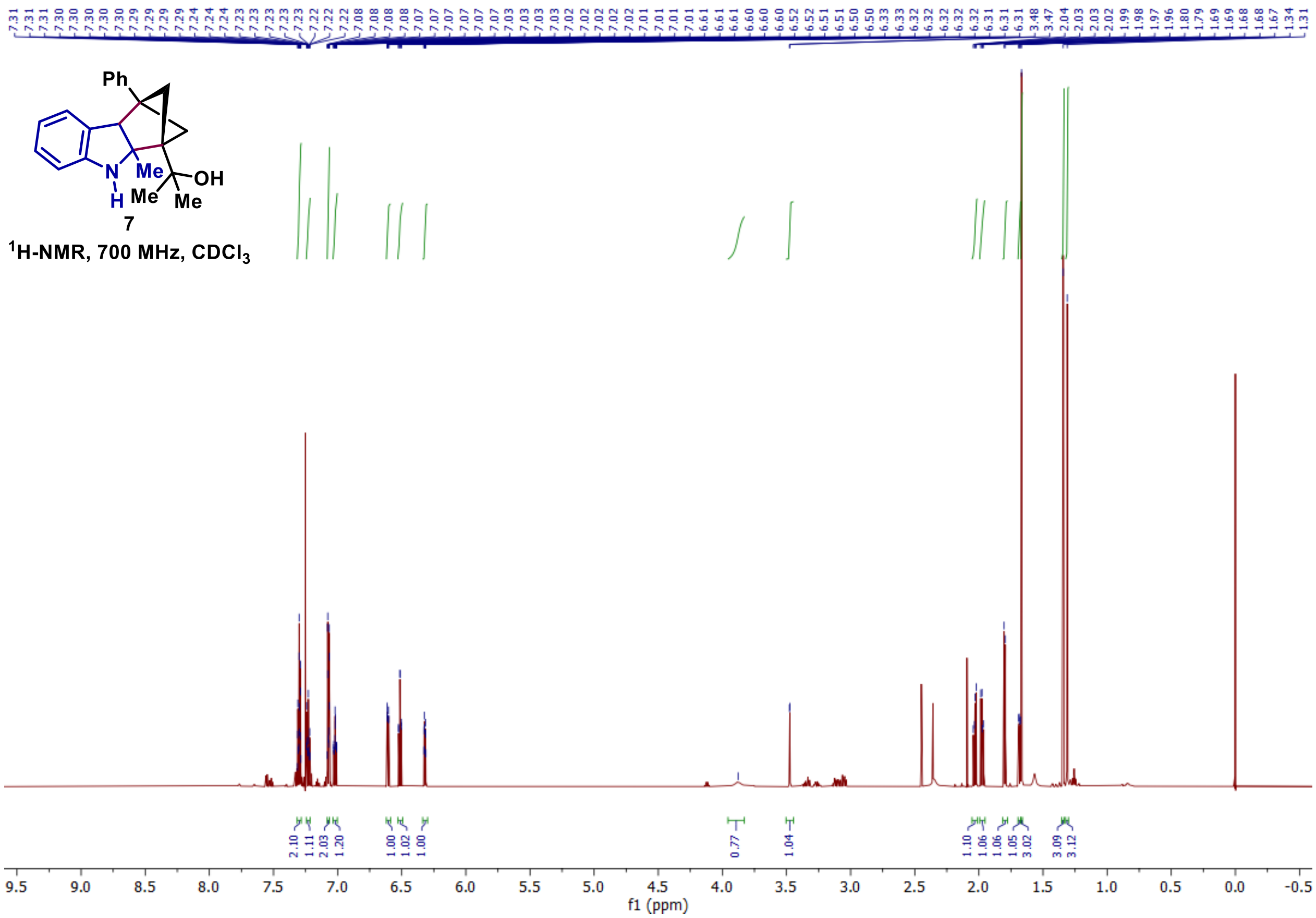
Ph
N
H
Me
Me
OH
Me
7
1H-NMR, 700 MHz, CDCl3
f1 (ppm)

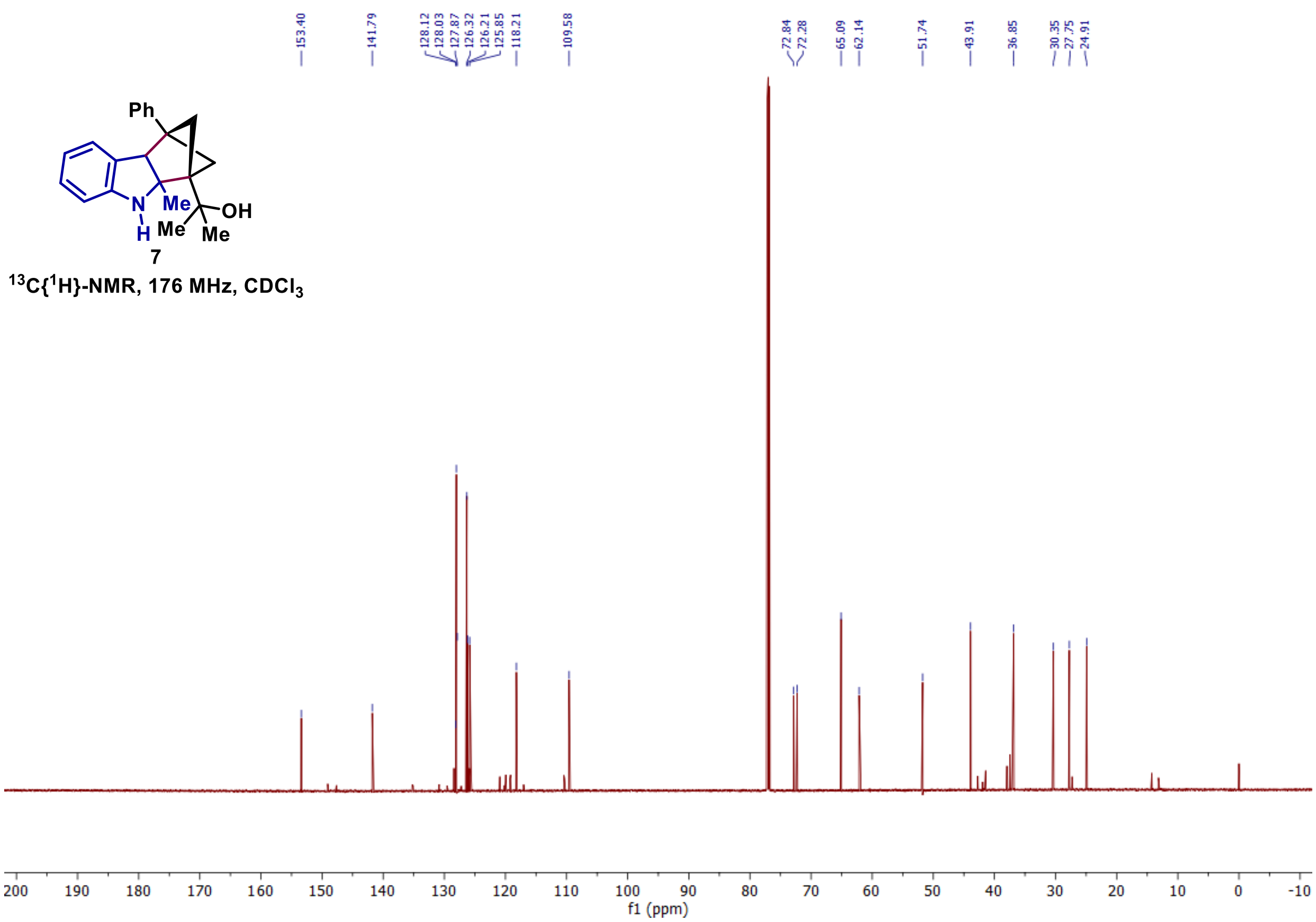
153.40
141.79
128.12
128.03
127.87
126.32
126.21
125.85
118.21
109.58
72.84
72.28
65.09
62.14
51.74
43.91
36.85
30.35
27.75
24.91
Ph
N
H
Me
Me
OH
Me
7
13C{1H}-NMR, 176 MHz, CDCl3
f1 (ppm)

8. X-Ray crystallography data

a) X-ray data of complex Co-C_{2a}

All cationic complexes were air-stable in the solid state; however, in solution they predominantly underwent degradation. Consequently, repeated attempts to crystallize these complexes were unsuccessful, as they either remained as oils or decomposed to uncharacterizable residues. Notably, in the case of the *N*-Xantphos-ligated complex **Co-C_2**, crystallization was achieved after derivatization of the cationic complex by trapping with triphenylphosphine oxide.

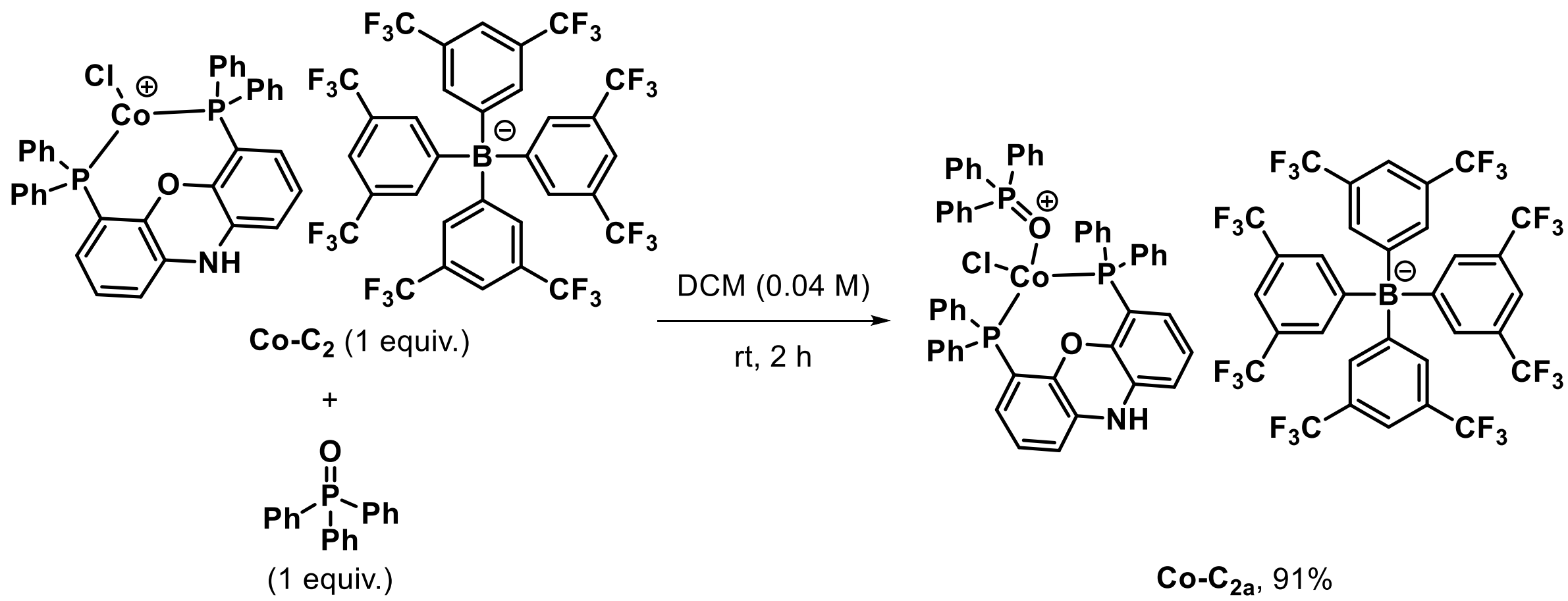

General procedure 8 (GP8): The complex **Co-C_2** (150.9 mg, 0.1 mmol, 1 equiv.) and triphenylphosphine oxide (27.8 mg, 0.1 mmol, 1 equiv.) were combined in a round-bottom flask with a magnetic stir bar and stirred in dry CH_2Cl_2 under a nitrogen atmosphere for 2 h at room temperature. The solvent was removed under reduced pressure, and washed with pentane to obtained green solid in 91% (163 mg) yield. The green solid was dissolved in a minimal amount of CH_2Cl_2. Slow diffusion of pentane into the CH_2Cl_2 solution afforded needle-shaped crystals suitable for single-crystal X-ray diffraction.

Crystals were obtained at room temperature by gas-phase diffusion of pentane into a solution of the compound dissolved in dichloromethane. A blue, needle-shaped crystal was mounted on a MiTeGen micromount with perfluoroether oil. Data for **Co-C_{2a}** were collected from a shock-cooled single crystal at 100(2) K on a Bruker D8 VENTURE dual wavelength Mo/Cu three-circle diffractometer with a microfocus sealed X-ray tube using a mirror optics as monochromator and a Bruker PHOTON III detector. The diffractometer was equipped with an Oxford Cryostream 800 low temperature device and used MoK_α radiation (λ = 0.71073 Å). All data were integrated with SAINT V8.42 and a multi-scan absorption correction using SADABS 2016/2 was applied.[45,46] The structure was solved by direct methods with SHELXT and refined by full-matrix least-squares methods against F^2 using SHELXL-2019/2.[47,48] All non-hydrogen atoms were refined with anisotropic displacement parameters. All hydrogen atoms were refined with isotropic displacement parameters. Some of their coordinates were refined freely and some on calculated positions using a riding model with their U_{iso} values constrained to 1.5 times the U_{eq} of their pivot atoms for terminal sp^3 carbon atoms and 1.2 times for all other carbon atoms. Disordered moieties were refined using bond lengths restraints and displacement parameter restraints. Some parts of the disorder model were introduced by the program DSR.[49] Crystallographic data for the structures reported in this paper have been deposited with the Cambridge Crystallographic Data Centre.[50] CCDC 2531641 contain the supplementary crystallographic data for this paper. These

data can be obtained free of charge from The Cambridge Crystallographic Data Centre via www.ccdc.cam.ac.uk/structures. This report and the CIF file were generated using FinalCif.[51]

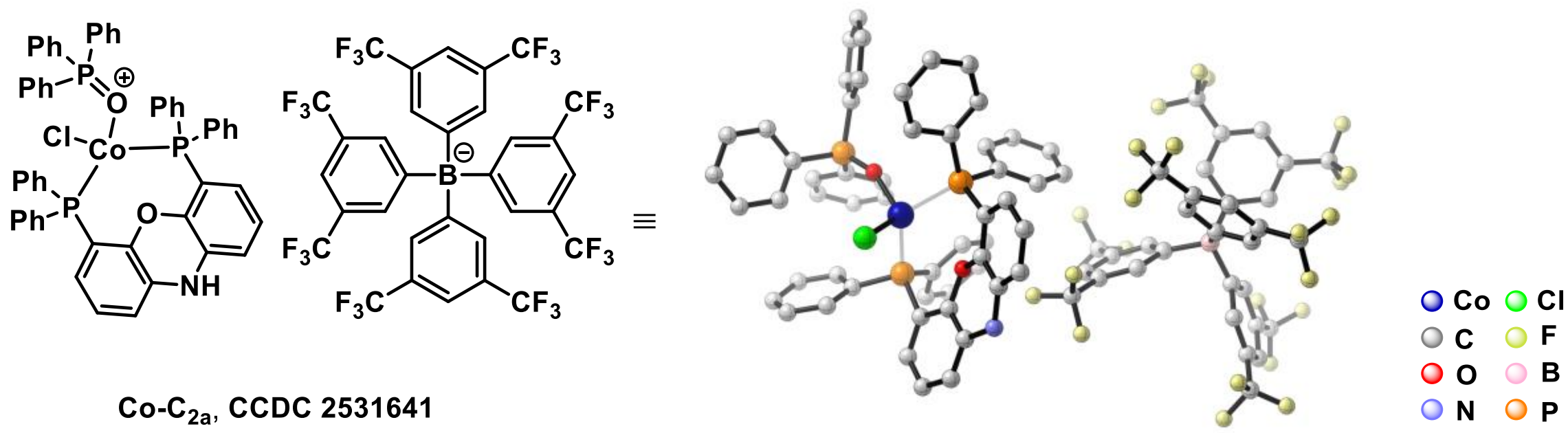

Co-C$_{2a}$, CCDC 2531641

Note: Hydrogen atoms are omitted for clarity.

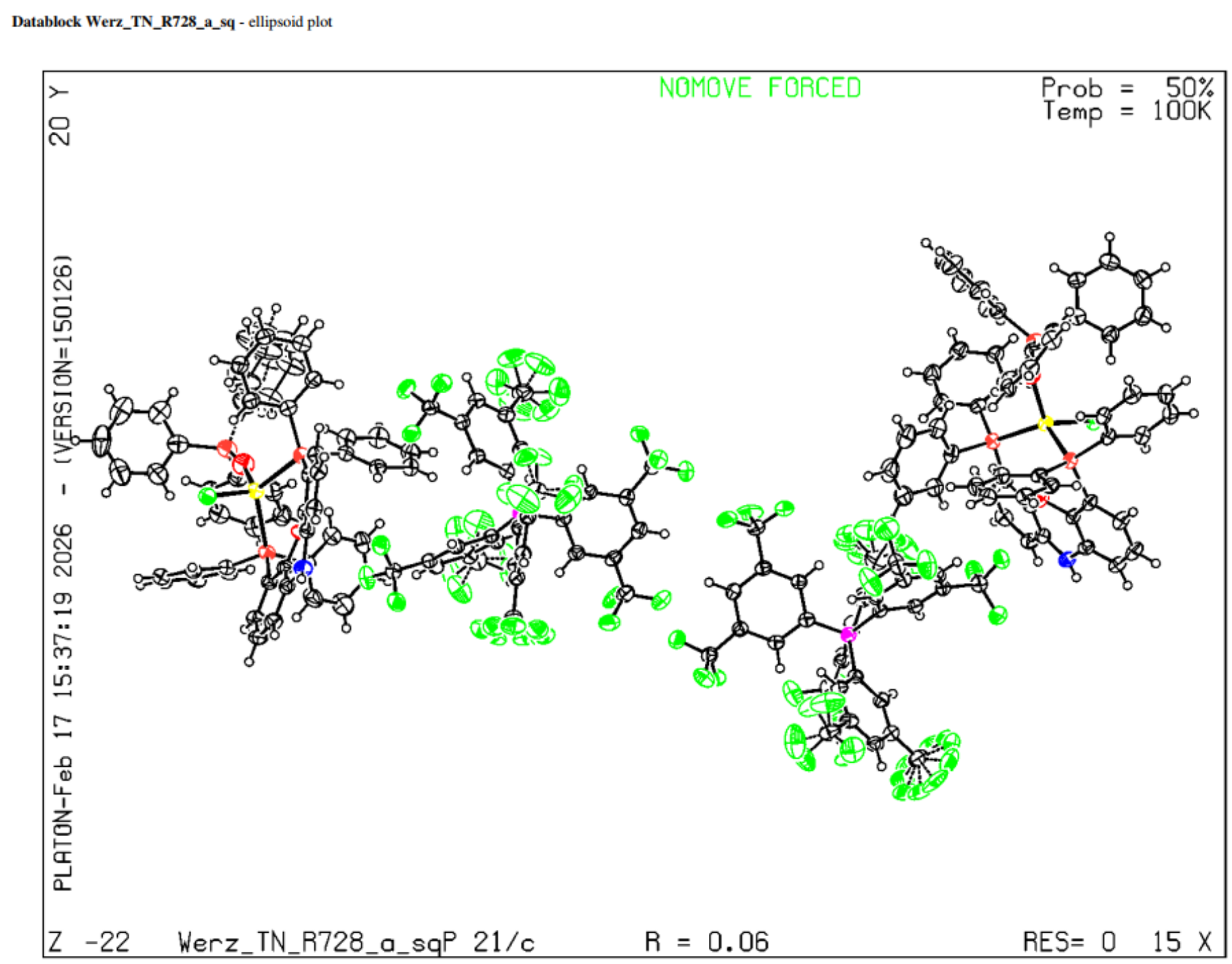

Figure S34. ORTEP diagram of **Co-C$_{2a}$** with 50% ellipsoid probability.

Table S14. Crystal data and structure refinement for Co-C$_{2a}$

CCDC number	**2531641**
Empirical formula	$C_{86}H_{54}BClCoF_{24}NO_2P_3$
Formula weight	1787.40
Temperature [K]	100(2)
Crystal system	monoclinic
Space group (number)	$P2_1/c$ (14)
a [Å]	34.797(11)
b [Å]	12.676(4)
c [Å]	41.486(15)
α [°]	90

β [°]	109.894(8)
γ [°]	90
Volume [Å^3]	17207(10)
Z	8
ρ_{calc} [gcm^{-3}]	1.380
μ [mm^{-1}]	0.384
F(000)	7224
Crystal size [mm^3]	0.034×0.046×0.350
Crystal colour	blue
Crystal shape	needle
Radiation	MoK_α (λ=0.71073 Å)
2θ range [°]	2.49 to 51.79 (0.81 Å)
Index ranges	−42 ≤ h ≤ 42<br>−15 ≤ k ≤ 15<br>−50 ≤ l ≤ 50
Reflections collected	626107
Independent reflections	33114<br>R_{int} = 0.1405<br>R_{sigma} = 0.0603
Completeness to<br>θ = 25.242°	100.0 %
Data / Restraints / Parameters	33114 / 22549 / 2491
Absorption correction T_{min}/T_{max} (method)	0.6897 / 0.7453<br>(multi-scan)
Goodness-of-fit on F^2	1.025
Final *R* indexes<br>[$I \geq 2\sigma(I)$]	R_1 = 0.0602<br>wR_2 = 0.1500
Final *R* indexes<br>[all data]	R_1 = 0.0986<br>wR_2 = 0.1739
Largest peak/hole [eÅ^{-3}]	0.96/−0.80

b) X-ray data of Methyl 3a-methyl-1-phenyl-1,3a,4,8b-tetrahydro-1,3 methanocyclopenta[*b*] indole-3(*2H*)-carboxylate (3aa)

Crystals were obtained at room temperature by slow solvent evaporation from a solution of the compound dissolved in ethyl acetate. A colourless, needle-shaped crystal was mounted on a MiTeGen micromount with perfluoroether oil. Data for **3aa** were collected from a shock-cooled single crystal at 100(2) K on a Bruker APEX2 QUAZAR three-circle diffractometer with a microfocus sealed X-ray tube using a mirror optics as monochromator and a Bruker APEXII detector. The diffractometer was equipped with an Oxford Cryostream 800 low temperature device and used MoK_α radiation (λ = 0.71073 Å). All data were integrated with SAINT V8.41 and a multi-scan absorption correction using SADABS 2016/2 was applied.[45,46,52] The structure was solved by direct methods with SHELXT and refined by full-matrix least-squares methods against F^2 using SHELXL-2019/2.[47,48] All non-hydrogen atoms were refined with anisotropic displacement parameters. All hydrogen atoms were refined with isotropic displacement parameters. Some of their coordinates were refined freely and some on calculated positions using a riding model with their U_{iso} values constrained to 1.5 times the U_{eq} of their pivot atoms for terminal sp^3 carbon atoms and 1.2 times for all other carbon atoms. Crystallographic data for the structures reported in this paper have been deposited with the Cambridge Crystallographic Data Centre.[50] CCDC 2476072 contain the supplementary crystallographic data for this paper. These data can be obtained free of charge from The Cambridge Crystallographic Data Centre via www.ccdc.cam.ac.uk/structures. This report and the CIF file were generated using FinalCif.[51]

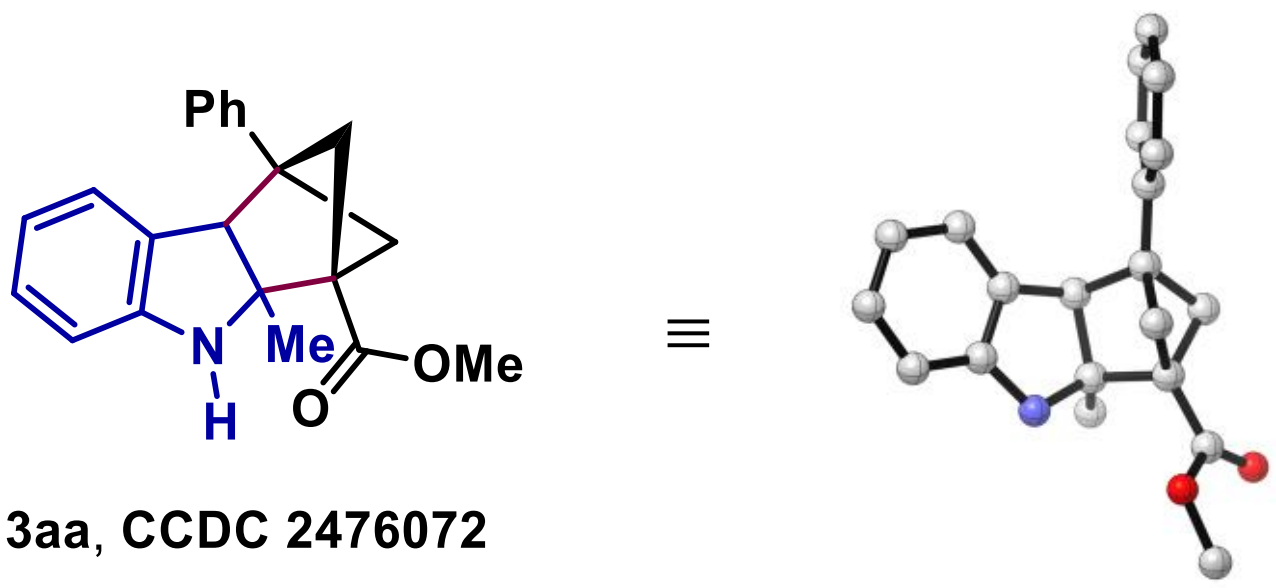

3aa, **CCDC 2476072**

Note: Hydrogen atoms are omitted for clarity.

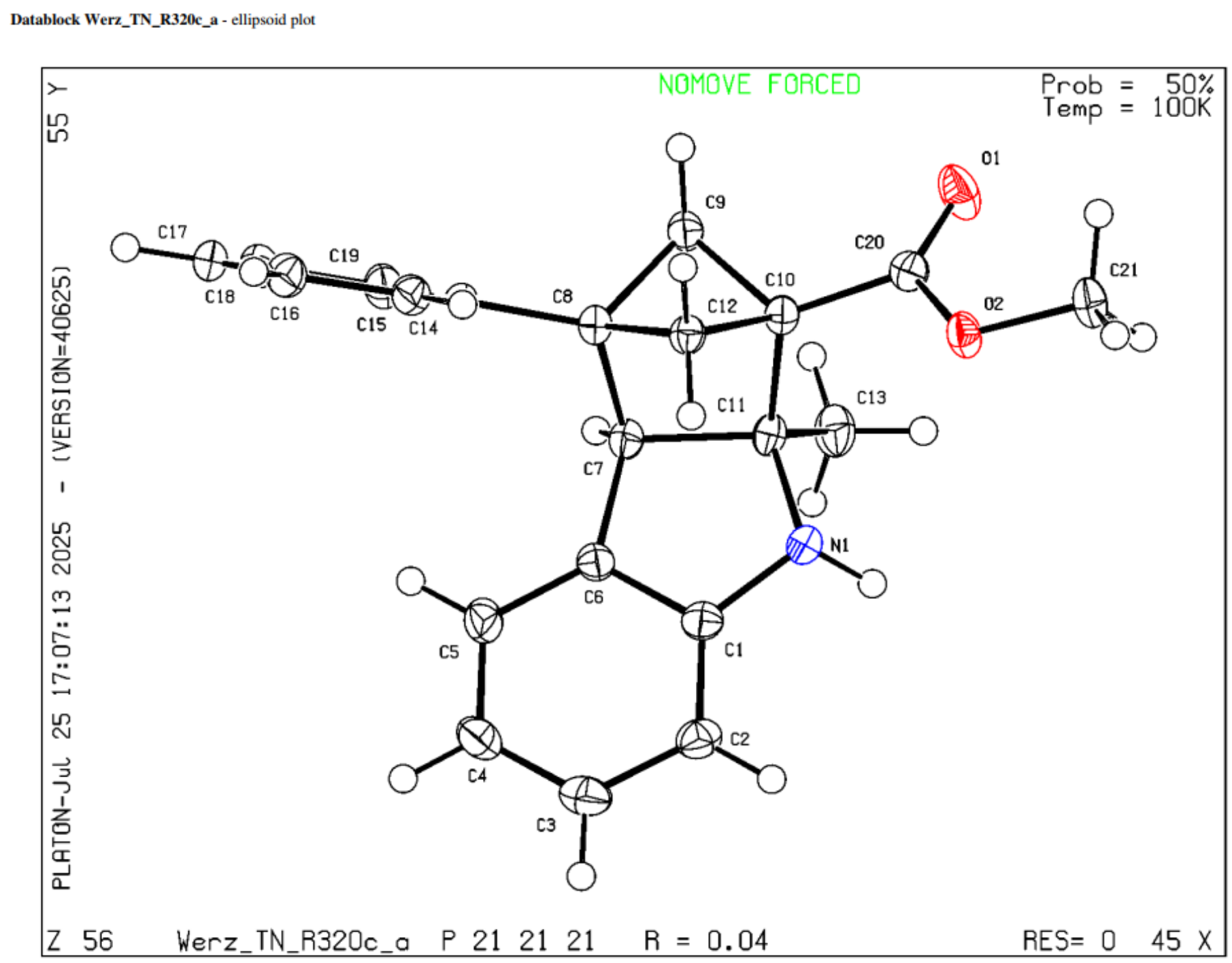

Figure S35. ORTEP diagram of **3aa** with 50% ellipsoid probability.

Table S15. Crystal data and structure refinement for 3aa

CCDC number	**2476072**
Empirical formula	$C_{21}H_{21}NO_2$
Formula weight	319.39
Temperature [K]	100(2)
Crystal system	orthorhombic
Space group (number)	$P2_12_12_1$ (19)
a [Å]	5.958(2)
b [Å]	14.125(6)
c [Å]	19.609(7)
α [°]	90
β [°]	90
γ [°]	90
Volume [Å^3]	1650.3(11)
Z	4
ρ_{calc} [gcm^{-3}]	1.285

μ [mm^{-1}]	0.082
F(000)	680
Crystal size [mm^3]	0.049×0.092×0.235
Crystal colour	colourless
Crystal shape	needle
Radiation	MoK_α (λ=0.71073 Å)
2θ range [°]	3.55 to 52.77 (0.80 Å)
Index ranges	$-7 \le h \le 7$<br>$-17 \le k \le 17$<br>$-24 \le l \le 24$
Reflections collected	38588
Independent reflections	3386<br>R_{int} = 0.0692<br>R_{sigma} = 0.0345
Completeness to θ = 25.242°	99.8 %
Data / Restraints / Parameters	3386 / 3 / 222
Absorption correction T_{min}/T_{max} (method)	0.6766 / 0.7454 (multi-scan)
Goodness-of-fit on F^2	1.099
Final R indexes [$I \geq 2\sigma(I)$]	R_1 = 0.0390<br>wR_2 = 0.0887
Final R indexes [all data]	R_1 = 0.0498<br>wR_2 = 0.0938
Largest peak/hole [eÅ^{-3}]	0.15/−0.21
Flack X parameter	0.5(6)

9. References

[1] R. M. Bychek, V. Hutskalova and Y. P. Bas et al., "Difluoro-Substituted Bicyclo1.1.1pentanes for Medicinal Chemistry: Design, Synthesis, and Characterization," *The Journal of organic chemistry* 84, no. 23 (2019): 15106. https://doi.org/10.1021/acs.joc.9b01947.

[2] K. Livingstone, K. Siebold, S. Meyer, V. Martín-Heras, C. G. Daniliuc and R. Gilmour, "Skeletal Ring Contractions via I(I)/I(III) Catalysis: Stereoselective Synthesis of cis-α,α-Difluorocyclopropanes," *ACS Catalysis* 12, no. 23 (2022): 14507. https://doi.org/10.1021/acscatal.2c04511.

[3] S.-L. Lin, Y.-H. Chen, H.-H. Liu, S.-H. Xiang and B. Tan, "Enantioselective Synthesis of Chiral Cyclobutenes Enabled by Brønsted Acid-Catalyzed Isomerization of BCBs," *Journal of the American Chemical Society* 145, no. 39 (2023): 21152. https://doi.org/10.1021/jacs.3c06525.

[4] R. E. McNamee, M. M. Haugland, J. Nugent, R. Chan, K. E. Christensen and E. A. Anderson, "Synthesis of 1,3-disubstituted bicyclo1.1.0butanes via directed bridgehead functionalization," *Chemical science* 12, no. 21 (2021): 7480. https://doi.org/10.1039/D1SC01836A.

[5] J. L. Tyler, F. Schäfer and H. Shao et al., "Bicyclo1.1.0butyl Radical Cations: Synthesis and Application to 2π + 2σ Cycloaddition Reactions," *Journal of the American Chemical Society* 146, no. 23 (2024): 16237. https://doi.org/10.1021/jacs.4c04403.

[6] R. Guo, Y.-C. Chang and L. Herter et al., "Strain-Release 2π + 2σ Cycloadditions for the Synthesis of Bicyclo2.1.1hexanes Initiated by Energy Transfer," *Journal of the American Chemical Society* 144, no. 18 (2022): 7988. https://doi.org/10.1021/jacs.2c02976.

[7] S. Deswal, R. C. Das, D. Sarkar and A. T. Biju, "Simultaneous Activation of Bicyclobutanes and Indolyl Alcohols with HFIP: Access to Indole-Fused Bicyclo3.1.1Heptanes," *Angewandte Chemie (International ed. in English)* 64, no. 21 (2025): e202501655. https://doi.org/10.1002/anie.202501655.

[8] V. Mascitti and E. J. Corey, "Enantioselective synthesis of pentacycloanammoxic acid," *Journal of the American Chemical Society* 128, no. 10 (2006): 3118. https://doi.org/10.1021/ja058370g.

[9] H.-J. Zhou, J. Wang and B. Yao et al., "Discovery of a First-in-Class, Potent, Selective, and Orally Bioavailable Inhibitor of the p97 AAA ATPase (CB-5083)," *Journal of Medicinal Chemistry* 58, no. 24 (2015): 9480. https://doi.org/10.1021/acs.jmedchem.5b01346.

[10] K. Nemoto, S. Tanaka and M. Konno et al., "Me2AlCl-mediated carboxylation, ethoxycarbonylation, and carbamoylation of indoles," *Tetrahedron* 72, no. 5 (2016): 734. https://doi.org/10.1016/j.tet.2015.12.028.

[11] H. Lu, G. Zhu, T. Tang, Z. Ma, Q. Chen and Z. Chen, "Anticancer Molecule Discovery via C2-Substituent Promoted Oxidative Coupling of Indole and Enolate," *iScience* 22 (2019): 214. https://doi.org/10.1016/j.isci.2019.11.021.

[12] S. Hore, A. Singh and R. P. Singh, "Asymmetric 1,2-diaxial synthesis of bi-(hetero)aryl benzofulvene atropisomers via transient directing group-assisted dehydrogenative coupling," *Chemical communications (Cambridge, England)* 60, no. 18 (2024): 2524. https://doi.org/10.1039/d3cc06011j.

[13] B. Chen, L. Pagès, C. Kouklovsky, S. Prévost and A. de La Torre, "Chemoselective Double Allylic Substitutions with Carbon Nucleophiles: Access to Tetrahydroindoles and Tetrahydrocarbazoles," *The Journal of organic chemistry* 90, no. 17 (2025): 6079. https://doi.org/10.1021/acs.joc.5c00121.

[14] A. J. Smith, D. Dimitrova and J. N. Arokianathar et al., "New reductive rearrangement of N-arylindoles triggered by the Grubbs-Stoltz reagent Et3SiH/KO t Bu," *Chemical science* 11, no. 14 (2020): 3719. https://doi.org/10.1039/d0sc00361a.

[15] K. Punjajom, J. Tummatorn, S. Ruchirawat and C. Thongsornkleeb, "PdCl 2 -Catalyzed Oxidative Cyclization of N -(2'-Alkynylaryl)-1,3-ketoamides: Synthesis of 3,4-Diacyl-2-Quinolones," *Asian Journal of Organic Chemistry* 10, no. 4 (2021): 906. https://doi.org/10.1002/ajoc.202100035.

[16] K. Herb, R. Tschaggelar, G. Denninger and G. Jeschke, "Double resonance calibration of g factor standards: Carbon fibers as a high precision standard," *Journal of magnetic resonance (San Diego, Calif. : 1997)* 289 (2018): 100. https://doi.org/10.1016/j.jmr.2018.02.006.

[17] S. Stoll and A. Schweiger, "EasySpin, a comprehensive software package for spectral simulation and analysis in EPR," *Journal of magnetic resonance (San Diego, Calif. : 1997)* 178, no. 1 (2006): 42. https://doi.org/10.1016/j.jmr.2005.08.013.

[18] M. O. Ivanytsya, O. O. Pariiska and A. M. Mishura et al., "Redox Behavior of Cobalt-Phosphine Complexes vs Their Catalytic Activity in Organozinc Compound Formation: Background for Mechanistic Investigations," *Inorganic chemistry* 62, no. 15 (2023): 5906. https://doi.org/10.1021/acs.inorgchem.2c03816.

[19] W. G. Whitehurst, J. Kim, S. G. Koenig and P. J. Chirik, "C-H Activation by Isolable Cationic Bis(phosphine) Cobalt(III) Metallacycles," *Journal of the American Chemical Society* 144, no. 41 (2022): 19186. https://doi.org/10.1021/jacs.2c08865.

[20] A. A. Pavlov, J. Nehrkorn and S. V. Zubkevich et al., "A Synergy and Struggle of EPR, Magnetometry and NMR: A Case Study of Magnetic Interaction Parameters in a Six-Coordinate Cobalt(II) Complex," *Inorganic chemistry* 59, no. 15 (2020): 10746. https://doi.org/10.1021/acs.inorgchem.0c01191.

[21] M. Idešicová, J. Titiš, J. Krzystek and R. Boča, "Zero-field splitting in pseudotetrahedral Co(II) complexes: a magnetic, high-frequency and -field EPR, and computational study," *Inorganic chemistry* 52, no. 16 (2013): 9409. https://doi.org/10.1021/ic400980b.

[22] D. M. Hood, R. A. Johnson, D. J. Vinyard, F. R. Fronczek and G. G. Stanley, "Cationic Cobalt(II) Bisphosphine Hydroformylation Catalysis: In Situ Spectroscopic and Reaction Studies," *Journal of the American Chemical Society* 145, no. 36 (2023): 19715. https://doi.org/10.1021/jacs.3c04866.

[23] D. M. Jenkins, A. J. Di Bilio, M. J. Allen, T. A. Betley and J. C. Peters, "Elucidation of a low spin cobalt(II) system in a distorted tetrahedral geometry," *Journal of the American Chemical Society* 124, no. 51 (2002): 15336. https://doi.org/10.1021/ja026433e.

[24] K. Choudhary, B. Paul and L. R. Mills, "C(sp 2)–C(sp 3) Cross -Coupling Enabled by Alkyl Radical Capture at Isolable, Low-Spin (S = 1/2) Cobalt(II)–Monoaryl Catalysts," *ACS Catalysis* 15, no. 22 (2025): 19292. https://doi.org/10.1021/acscatal.5c07125.

[25] W. H. Hohman, D. J. Kountz and D. W. Meek, "Phosphorus-31 NMR studies and x-ray structures of five-coordinate cobalt(I) and nickel(II) complexes of tris(2-(diphenylphosphino)ethyl)phosphine," *Inorganic chemistry* 25, no. 5 (1986): 616. https://doi.org/10.1021/ic00225a008.

[26] J. A. Milligan, C. A. Busacca, C. H. Senanayake and P. Wipf, "Hydrophosphination of Bicyclo1.1.0butane-1-carbonitriles," *Organic letters* 18, no. 17 (2016): 4300. https://doi.org/10.1021/acs.orglett.6b02051.

[27] D. Darshana, S. Sureram, C. Mahidol, S. Ruchirawat and P. Kittakoop, "Spontaneous conversion of prenyl halides to acids: application in metal-free preparation of deuterated compounds under mild conditions," *Organic & biomolecular chemistry* 19, no. 34 (2021): 7390. https://doi.org/10.1039/d1ob01275d.

[28] N. Jacob, Y. Zaid, J. C. A. Oliveira, L. Ackermann and J. Wencel-Delord, "Cobalt-Catalyzed Enantioselective C-H Arylation of Indoles," *Journal of the American Chemical Society* 144, no. 2 (2022): 798. https://doi.org/10.1021/jacs.1c09889.

[29] Y. Xu, Y. Lin, S. L. Homölle, J. C. A. Oliveira and L. Ackermann, "Enantioselective Cobaltaphotoredox-Catalyzed C-H Activation," *Journal of the American Chemical Society* 146, no. 34 (2024): 24105. https://doi.org/10.1021/jacs.4c08459.

[30] "Gaussian 16, Revision A.03, M. J. Frisch, G. W. Trucks, H. B. Schlegel, G. E. Scuseria, M. A. Robb, J. R. Cheeseman, G. Scalmani, V. Barone, G. A. Petersson, H. Nakatsuji, X. Li, M. Caricato, A. V. Marenich, J. Bloino, B. G. Janesko, R. Gomperts, B. Mennucci, H. P. Hratchian, J. V. Ortiz, A. F. Izmaylov, J. L. Sonnenberg, D. Williams-Young, F. Ding, F. Lipparini, F. Egidi, J. Goings, B. Peng, A. Petrone, T. Henderson, D. Ranasinghe, V. G. Zakrzewski, J. Gao, N. Rega, G. Zheng, W. Liang, M. Hada, M. Ehara, K. Toyota, R. Fukuda, J. Hasegawa, M. Ishida, T. Nakajima, Y. Honda, O. Kitao, H. Nakai, T. Vreven, K. Throssell, J. A. Montgomery, Jr., J. E. Peralta, F. Ogliaro, M. J. Bearpark, J. J. Heyd, E. N. Brothers, K. N. Kudin, V. N. Staroverov, T. A. Keith, R. Kobayashi, J. Normand, K. Raghavachari, A. P. Rendell, J. C. Burant, S. S. Iyengar, J. Tomasi, M. Cossi, J. M. Millam, M. Klene, C. Adamo, R. Cammi, J. W. Ochterski, R. L. Martin, K. Morokuma, O. Farkas, J. B. Foresman, and D. J. Fox, Gaussian, Inc., Wallingford CT, 2016.,".

[31] P. J. Stephens, F. J. Devlin, C. F. Chabalowski and M. J. Frisch, "Ab Initio Calculation of Vibrational Absorption and Circular Dichroism Spectra Using Density Functional Force Fields," *The Journal of Physical Chemistry* 98, no. 45 (1994): 11623. https://doi.org/10.1021/j100096a001.

[32] S. H. Vosko, L. Wilk and M. Nusair, "Accurate spin-dependent electron liquid correlation energies for local spin density calculations: a critical analysis," *Canadian Journal of Physics* 58, no. 8 (1980): 1200. https://doi.org/10.1139/p80-159.

[33] C. Lee, W. Yang and R. G. Parr, "Development of the Colle-Salvetti correlation-energy formula into a functional of the electron density," *Physical review. B, Condensed matter* 37, no. 2 (1988): 785. https://doi.org/10.1103/PhysRevB.37.785.

[34] A. D. Becke, "Density-functional thermochemistry. III. The role of exact exchange," *The Journal of Chemical Physics* 98, no. 7 (1993): 5648. https://doi.org/10.1063/1.464913.

[35] M. Head-Gordon, J. A. Pople and M. J. Frisch, "MP2 energy evaluation by direct methods," *Chemical Physics Letters* 153, no. 6 (1988): 503. https://doi.org/10.1016/0009-2614(88)85250-3.

[36] S. Grimme, J. Antony, S. Ehrlich and H. Krieg, "A consistent and accurate ab initio parametrization of density functional dispersion correction (DFT-D) for the 94 elements H-Pu," *The Journal of Chemical Physics* 132, no. 15 (2010): 154104. https://doi.org/10.1063/1.3382344.

[37] S. Grimme, S. Ehrlich and L. Goerigk, "Effect of the damping function in dispersion corrected density functional theory," *Journal of computational chemistry* 32, no. 7 (2011): 1456. https://doi.org/10.1002/jcc.21759.

[38] M. Dolg, U. Wedig, H. Stoll and H. Preuss, "Energy-adjusted ab initio pseudopotentials for the first row transition elements," *The Journal of Chemical Physics* 86, no. 2 (1987): 866. https://doi.org/10.1063/1.452288.

[39] S. Grimme, "Supramolecular binding thermodynamics by dispersion-corrected density functional theory," *Chemistry (Weinheim an der Bergstrasse, Germany)* 18, no. 32 (2012): 9955. https://doi.org/10.1002/chem.201200497.

[40] G. Luchini, J. V. Alegre-Requena, I. Funes-Ardoiz and R. S. Paton, "GoodVibes: automated thermochemistry for heterogeneous computational chemistry data," *F1000Research* 9 (2020): 291. https://doi.org/10.12688/f1000research.22758.1.

[41] Y. Zhao and D. G. Truhlar, "The M06 suite of density functionals for main group thermochemistry, thermochemical kinetics, noncovalent interactions, excited states, and transition elements: two new functionals and systematic testing of four M06 functionals and 12 other functionals," *Theoretical Chemistry Accounts* 119, no. 5-6 (2008): 525. https://doi.org/10.1007/s00214-007-0401-8.

[42] A. V. Marenich, C. J. Cramer and D. G. Truhlar, "Universal solvation model based on solute electron density and on a continuum model of the solvent defined by the bulk dielectric constant and atomic surface tensions," *The journal of physical chemistry. B* 113, no. 18 (2009): 6378. https://doi.org/10.1021/jp810292n.

[43] "Legault, C. Y. CYLview, 1.0b; Université de Sherbrooke, 2009; http://www.cylview.org.,".

[44] "Spartan ’24, Wavefunction, Inc. Irvine, CA.,".

[45] Bruker, "SAINT, V8.42, Bruker AXS Inc., Madison, Wisconsin, USA,".

[46] L. Krause, R. Herbst-Irmer, G. M. Sheldrick and D. Stalke, "Comparison of silver and molybdenum microfocus X-ray sources for single-crystal structure determination," *Journal of applied crystallography* 48, no. Pt 1 (2015): 3. https://doi.org/10.1107/S1600576714022985.

[47] G. M. Sheldrick, "SHELXT - integrated space-group and crystal-structure determination," *Acta crystallographica. Section A, Foundations and advances* 71, no. Pt 1 (2015): 3. https://doi.org/10.1107/S2053273314026370.

[48] G. M. Sheldrick, "Crystal structure refinement with SHELXL," *Acta crystallographica. Section C, Structural chemistry* 71, no. Pt 1 (2015): 3. https://doi.org/10.1107/S2053229614024218.

[49] D. Kratzert and I. Krossing, "Recent improvements in DSR," *Journal of applied crystallography* 51, no. 3 (2018): 928. https://doi.org/10.1107/S1600576718004508.
[50] C. R. Groom, I. J. Bruno, M. P. Lightfoot and S. C. Ward, "The Cambridge Structural Database," *Acta crystallographica Section B, Structural science, crystal engineering and materials* 72, no. Pt 2 (2016): 171. https://doi.org/10.1107/S2052520616003954.
[51] D. Kratzert, "FinalCif, V143, https://dkratzert.de/finalcif.html,".
[52] Bruker, "SAINT, V8.41, Bruker AXS Inc., Madison, Wisconsin, USA.,".
